# AAV-mediated gene therapy demonstrates phenotypic rescue in a mouse model of Cockayne syndrome

**DOI:** 10.1172/JCI196689

**Published:** 2026-07-30

**Authors:** Ana Rita Batista, Aine C. Scholand, William S. Callahan, McKenna K. Watson, Cassandra M. Sion, Tyler Mola, Kennedy O’Hara, Simon A. Wentworth, William S. Sena-Esteves, Oliver D. King, Robert M. King, Miguel Sena-Esteves

**Affiliations:** 1 Department of Genetic and Cellular Medicine,; 2 Department of Neurology,; 3 MD/PhD program, Morningside Graduate School of Biomedical Sciences, T.H. Chan School of Medicine, and; 4 Department of Radiology, University of Massachusetts Chan Medical School, Worcester, Massachusetts, USA.

**Keywords:** Genetics, Neuroscience, DNA repair, Gene therapy, Genetic diseases

## Abstract

Cockayne syndrome (CS) is an autosomal recessive, progressive developmental and neurodegenerative disease. Approximately 30% of cases are caused by mutations in the *ERCC8/CSA* gene. Patients with CS present with cutaneous photosensitivity, growth failure, shorter life span, and a progressive degeneration of the central nervous system. Loss-of-function mutations in CSA result in deficiencies in transcription-coupled nucleotide excision repair. Currently, no therapies are available for these patients. Adeno-associated virus–mediated (AAV-mediated) gene therapy offers an opportunity to address this unmet need. We designed an AAV vector encoding human CSA under a ubiquitous promoter. We tested the therapeutic efficacy of this AAV9-CSA vector by neonatal intracerebroventricular injection in the *Csa^–/–^ Xpa^–/–^* mouse model. Treatment with AAV9-CSA resulted in a significant increase in life span, and broad distribution of human CSA in the brain and heart, without evidence of vector-related toxicity. Despite clear therapeutic benefit, we also observed neuroradiological abnormalities, and neuropathologic alterations, including hypomyelination, astrocytosis, and microgliosis, as well as likely life-limiting transcriptomic alterations in liver at endpoint. Nonetheless, the success of these experiments paves the way for clinical translation of an AAV gene therapy for patients with CS into humans.

## Abstract 2

For the first time, gene therapy successfully treats Cockayne Syndrome in mice, extending lifespan safely. This milestone opens the door to human clinical trials for this rare disease.

## Graphical abstract

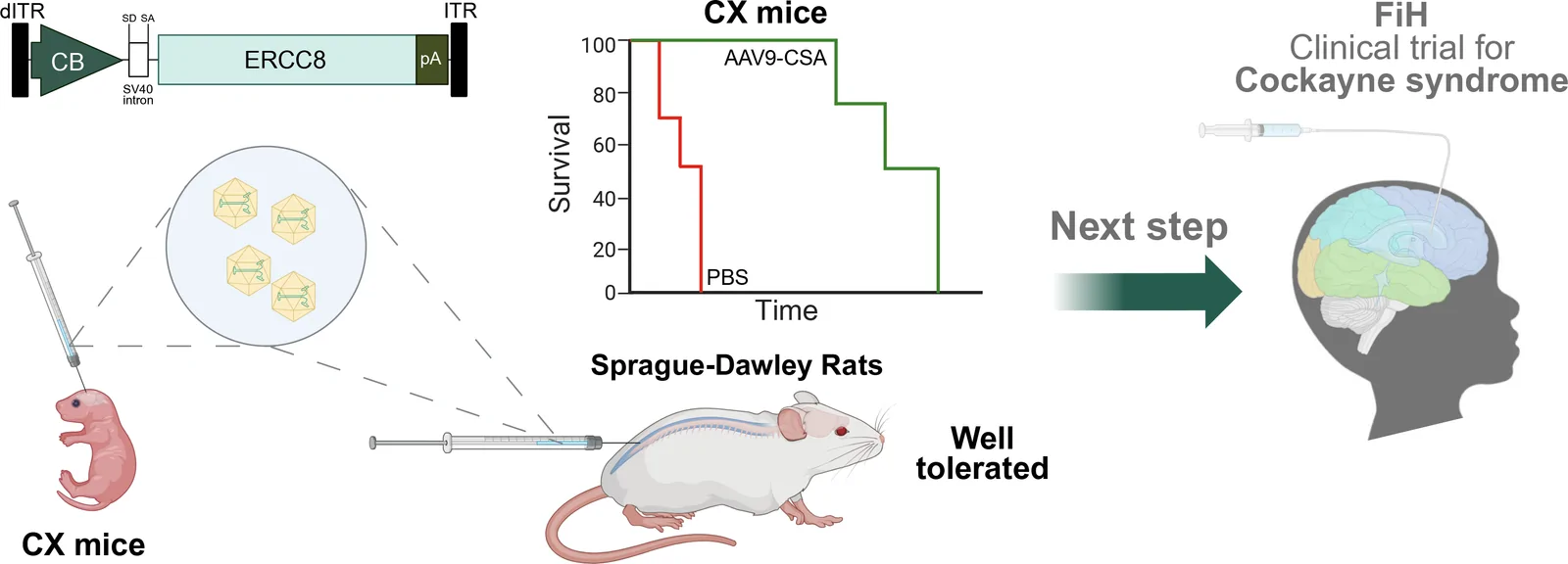

## Introduction

Cockayne syndrome (CS) is a multisystem, autosomal recessive, progressive developmental and neurodegenerative disorder, caused primarily by mutations in either the *ERCC8* (Cockayne syndrome A or *CSA*) or *ERCC6* (Cockayne syndrome B or *CSB*) gene. It is very rare, devastating, and almost universally fatal in children. The incidence of CS is 1 per 250,000 live births, but it is frequently undiagnosed (1–4). The hallmark signs of disease are microcephaly, growth failure, and developmental delay. Other common features include short stature, cataracts, sunken eyes, hearing loss, cutaneous photosensitivity, liver dysfunction, dental caries, and morphological abnormalities of the teeth (5–8). The central nervous system (CNS) is the most severely affected by CS, and its pathology contributes substantially to morbidity. However, the most common cause of death is pneumonia and recurrent respiratory infection (6, 9).

*ERCC8* (*CSA*) and *ERCC6* (*CSB*) mutations account for approximately 30% and 70% of CS cases, respectively (6). Mutations in these genes result in defects in transcription-coupled nucleotide excision repair (TC-NER), leading to gene misregulation and cytotoxicity because of RNA polymerase II (RNA pol II) stalling in nondividing tissues (10, 11). Additionally, CSA and CSB proteins are involved in repairing oxidative DNA damage (12, 13), and in mitochondrial metabolism (14), both of which contribute to the growth failure and neurodegeneration observed in children. Our research focuses on the rarest form of CS, caused by mutations in the *ERCC8/CSA* gene.

CSA is a 44 kDa protein containing 7 WD40 repeats (15). CSA was originally described as a key player in the TC-NER pathway (15–18), which repairs bulky DNA lesions, such as those caused by UV light and/or redox damage. The triggering event for TC-NER is the arrest of RNA pol II caused by lesions in the actively transcribed strand of a gene. Upon stalling of RNA pol II, TC-NER is initiated by CSB, which recruits CSA as part of the CRL4-based E3 ubiquitin ligase complex. The complex then binds ELOF1 and recruits UVSSA, which in turn recruits TFIIH to RNA pol II. This complex recruits downstream factors, such as Xpa, and initiates the repair process once RNA pol II is degraded. In the absence of CSA, these repair factors are not recruited, and RNA pol II arrest persists, leading to p53 activation and cell death (11, 16, 19–24).

However, TC-NER defects alone do not explain the severe neurological disease observed in CS (25, 26). In addition to proteins in the TC-NER complex, CSA interacts with several other proteins involved in transcription, ribosomal biogenesis, and mitochondrial homeostasis, but the exact molecular mechanisms remain unclear (27).

Several animal models of CS have been developed over the years to understand the disease mechanism. The first *Csa^–/–^* mouse model presents with a minimal phenotype, exhibiting pronounced skin cancer only after chronic UV exposure and marked photoreceptor loss, but otherwise has no other apparent phenotypes or pathological alterations and has a normal life span (28). This mouse model also fails to reproduce the myriad somatic and neurologic deficits of CS, except for clusters of activated microglia observed in close association with mature oligodendrocytes, which resemble the patchy demyelination observed in patients (25, 29). To generate more aggressive models of CS, double-KO mice have been generated by deleting either *Xpa* (a component of the TC-NER complex) or *Adh5* (a formaldehyde-detoxifying enzyme), in addition to *Csa* or *Csb* (30, 31). Although these double-KO mice are genetically artificial models, they show that increasing the levels of endogenous DNA damage is required to faithfully recapitulate the neurological features of CS, and therefore, they are strong surrogates for testing new therapies.

There are currently no disease-modifying treatments to halt or slow the progression of CS, and clinical management is limited to supportive care aimed at alleviating disease manifestations. Because CS is a monogenic disorder caused by loss-of-function mutations, it is well suited for gene replacement therapy. Adeno-associated virus (AAV) vectors are a powerful and clinically validated platform for gene delivery, with multiple FDA-approved therapies, including those for CNS indications, and a substantial number of ongoing clinical trials (32).

Among available AAV serotypes, adeno-associated virus serotype 9 (AAV9) has emerged as a leading vector for CNS-directed gene therapy because of its unique ability to cross the blood-brain barrier following intravenous administration, enabling widespread transduction of neurons and astrocytes throughout the brain and spinal cord (33, 34). In particular, delivery into the cerebrospinal fluid via the cisterna magna or the intrathecal space achieves enhanced CNS biodistribution at lower vector doses than systemic injection, further positioning AAV9-based gene replacement therapy as an attractive approach for a broad range of monogenic CNS disorders (35).

Here, we report, to our knowledge, the first preclinical development of an AAV9-based gene therapy for CS, demonstrating significant phenotypic rescue in an aggressive mouse model of CS.

## Results

**Figure 1:**
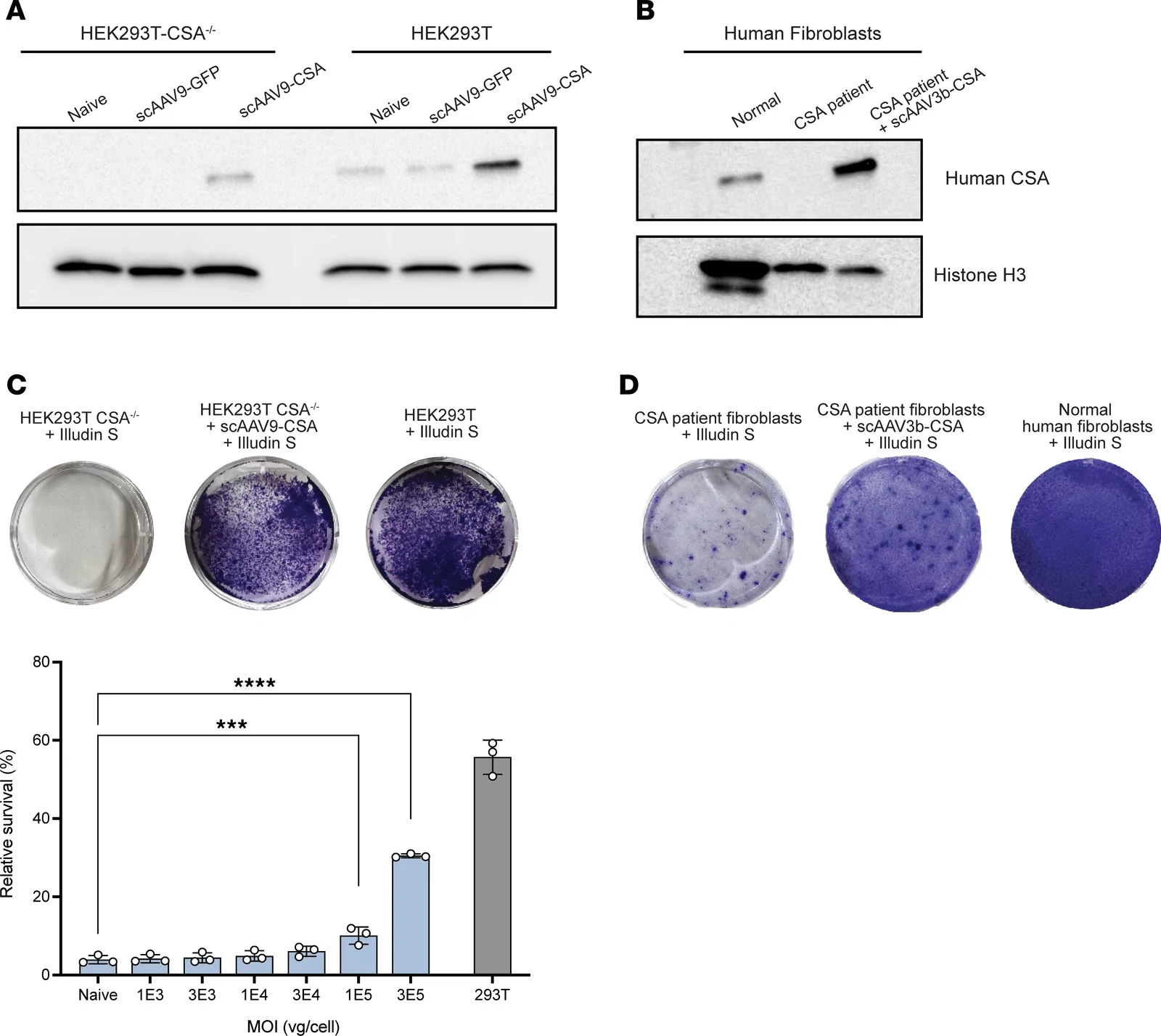
Validation of AAV-CBA-CSA in cell culture. (**A**) Representative Western blot showing CSA expression in HEK293T and HEK293T-CSA^–/–^ cells following infection with scAAV9-GFP or scAAV9-CSA, with naive cells as controls. (**B**) Representative Western blot of human fibroblasts from a normal control, CSA patient, and CSA patient following transduction with scAAV3b-CSA. Histone H3 was used as a loading control. (**C**) Representative images of Illudin S killing assay in HEK293T-CSA^–/–^ (top panel) and quantification of percentage survival for dose response (bottom panel). HEK293T cells were included as a positive control. Data are represented as mean ± SD with individual data points shown. Statistical significance versus naive cells was determined by 1-way ANOVA with Dunnett’s post hoc test; ***P < 0.001, ****P < 0.0001. (**D**) Representative images of Illudin S killing assay in human fibroblasts.

We designed an scAAV9-CSA vector encoding wild-type (WT) human *ERCC8* driven by the ubiquitous chicken β-actin (CBA) promoter. To test the efficacy of this new vector in vitro, we generated a CSA-KO HEK293T cell line (HEK293T-CSA^–/–^) by CRISPR editing (see Supplemental Figure 1; supplemental material available online with this article; https://doi.org/10.1172/JCI196689DS1). Upon infection of HEK293T-CSA^–/–^ cells with AAV9-CSA vector (1 × 10^5^ vector genomes/cell), CSA protein expression was equivalent to normal levels in HEK293T cells (Figure 1A). Infection of CS patient fibroblasts with an AAV3b-CSA vector at a dose of 1 × 10^5^ vg/cell resulted in CSA protein expression levels higher than in normal human fibroblasts (Figure 1B). We also used a functional assay to demonstrate the biological activity of the CSA protein by exposing cells to the chemotherapeutic drug Illudin S, which is a potent DNA-alkylating agent. DNA lesions caused by this genotoxic drug are repaired exclusively by TC-NER (36), leading to CSA-deficient cell death (22). Transduction of HEK293T-CSA^–/–^ with AAV9-CSA vector promoted cell survival in a dose-dependent manner (Figure 1C), as measured in a cell survival assay. Similarly, AAV treatment of CS patient-derived fibroblasts exposed to Illudin S showed increased survival when compared with untreated controls (Figure 1D), further supporting the functionality of vector-encoded CSA protein.

**Figure 2:**
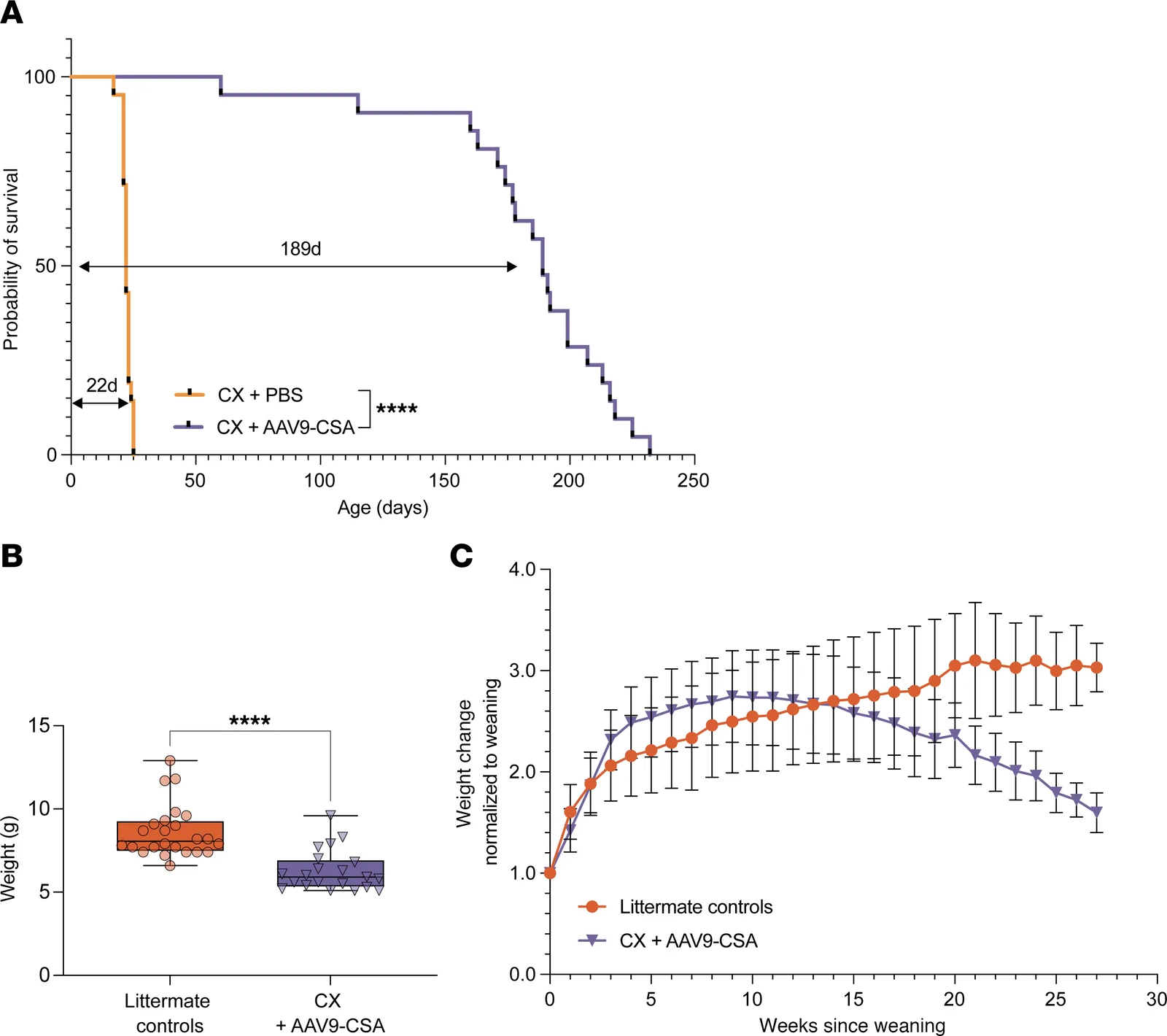
AAV9-CSA treatment increases life span and temporarily stabilizes the growth rate of CX mice. (**A**) Kaplan-Meier survival analysis of AAV-treated mice (*n* = 21) compared with untreated CX mice (*n* = 20). Statistical significance was assessed using the log-rank (Mantel-Cox) test, ****P < 0.0001. (**B**) Body weight at weaning (postnatal day 21) in AAV-treated CX mice (*n* = 21) and littermate controls (*n* = 24). Data are presented as median and interquartile range. Statistical analysis was performed using a 2-tailed Mann-Whitney test (****P < 0.0001). (**C**) Growth curves of AAV-treated mice showing weekly body weights normalized to individual weaning weights. Data are represented as mean ± SD.

To assess our vector in vivo, we used the severe double-KO mouse model for *Csa* and *Xpa* (*Csa^–/–^ Xpa^–/–^*; referred to here as CX mice). CX mice are indistinguishable from controls at birth, but their smaller size becomes apparent by postnatal day 5. By day 12, CS disease symptoms are evident, with a median survival of 22 days. Signs of CS disease include kyphosis, abnormal gait consistent with ataxia, uncoordinated hind limb movement, dystonia, and eventual paralysis (30). Neonatal (P0–P1) bilateral intracerebroventricular (ICV) administration of 1 × 10^11^ vg AAV9-CSA significantly increased (*P* < 0.0001) the median survival of CX mice from 22 days (PBS-injected controls) to 189 days, an 8.5-fold increase (Figure 2A). At weaning age (21 days), the body weight of AAV-treated CX mice was significantly lower than their littermate controls (Figure 2B). However, their growth rate was comparable to that of normal littermate controls until ~8 weeks of age (5 weeks postweaning), when they stopped gaining weight. The inflection point occurred at ~13 weeks of age, and body weight declined thereafter until the humane endpoint (Figure 2C). As there were no apparent differences in survival or growth rate between males and females, all animals enrolled in the study were combined into a single group for statistical analyses. Because the treatment was performed at P0–P1, all animals within a single litter were injected without prior knowledge of their genotypes. All treated littermates (*Csa^–/–^ Xpa^+/+^* or *Csa^–/–^ Xpa^+/–^*) were euthanized at the same time points as AAV9-CSA–treated CX mice, with no apparent adverse events.

**Figure 3:**
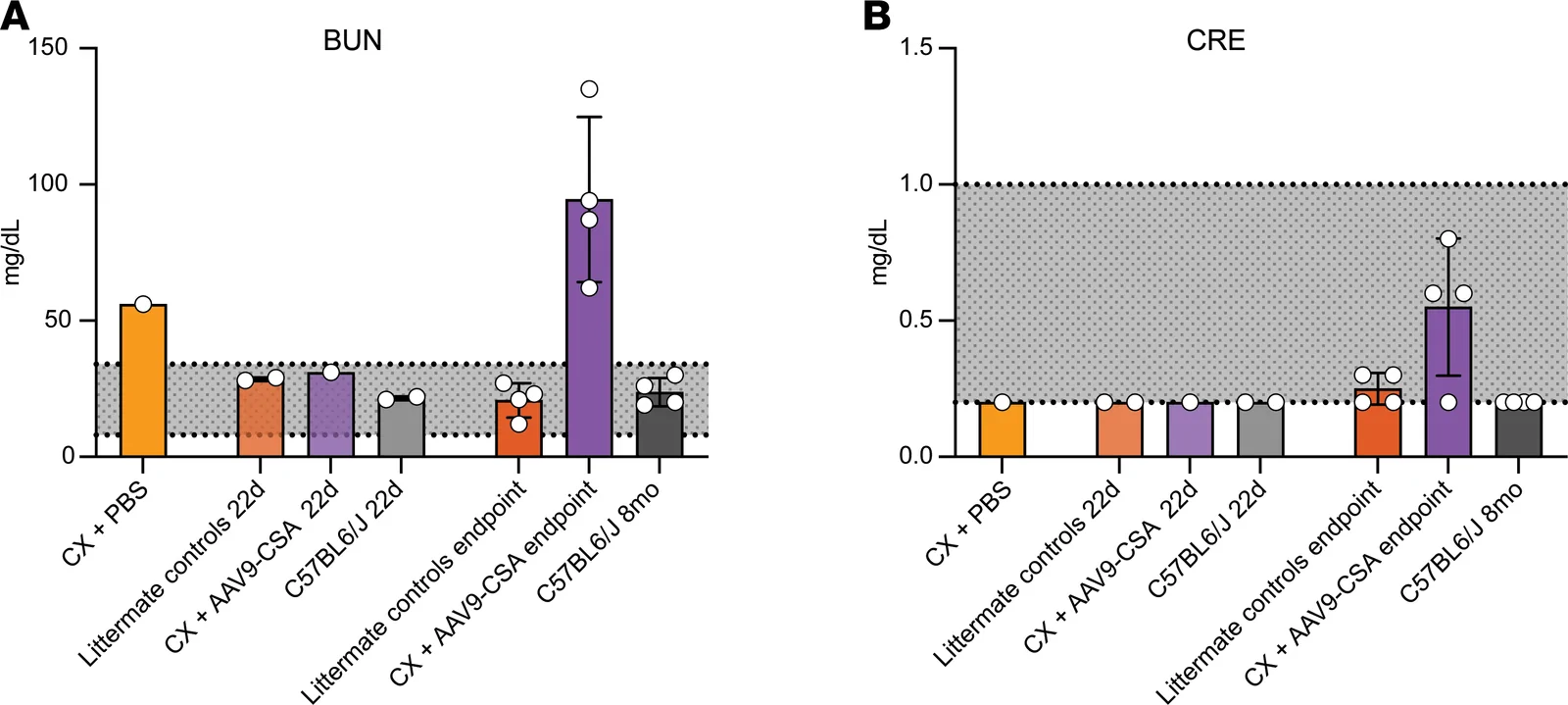
Serum markers of renal function. Blood urea nitrogen (BUN) (**A**) and creatinine (CRE) (**B**) were measured in CX-treated and untreated mice, littermate controls, and WT C57BL/6J mice at early (22 days) and endpoint time points. Dashed lines and shaded areas denote normal reference ranges for each analyte. Data are presented as mean ± SD with individual data points shown. Each individual data point represents a pooled serum sample from 2 animals within the same group.

To further evaluate the therapeutic impact of AAV treatment, we analyzed serum chemistry markers. Blood urea nitrogen (BUN) levels were notably elevated in PBS-injected CX mice at the endpoint, consistent with renal dysfunction, whereas they remained within the normal range at 21–22 days of age in AAV-treated CX mice (Figure 3A), indicating preserved renal function at this early time point. However, by endpoint, AAV-treated CX mice developed severe renal dysfunction, as evidenced by dramatically elevated BUN and creatinine (CRE) levels (Figure 3, A and B). In comparison, BUN and CRE levels in AAV-treated littermate controls remained within the normal range at both time points and were comparable to those observed in WT controls.

**Figure 4:**
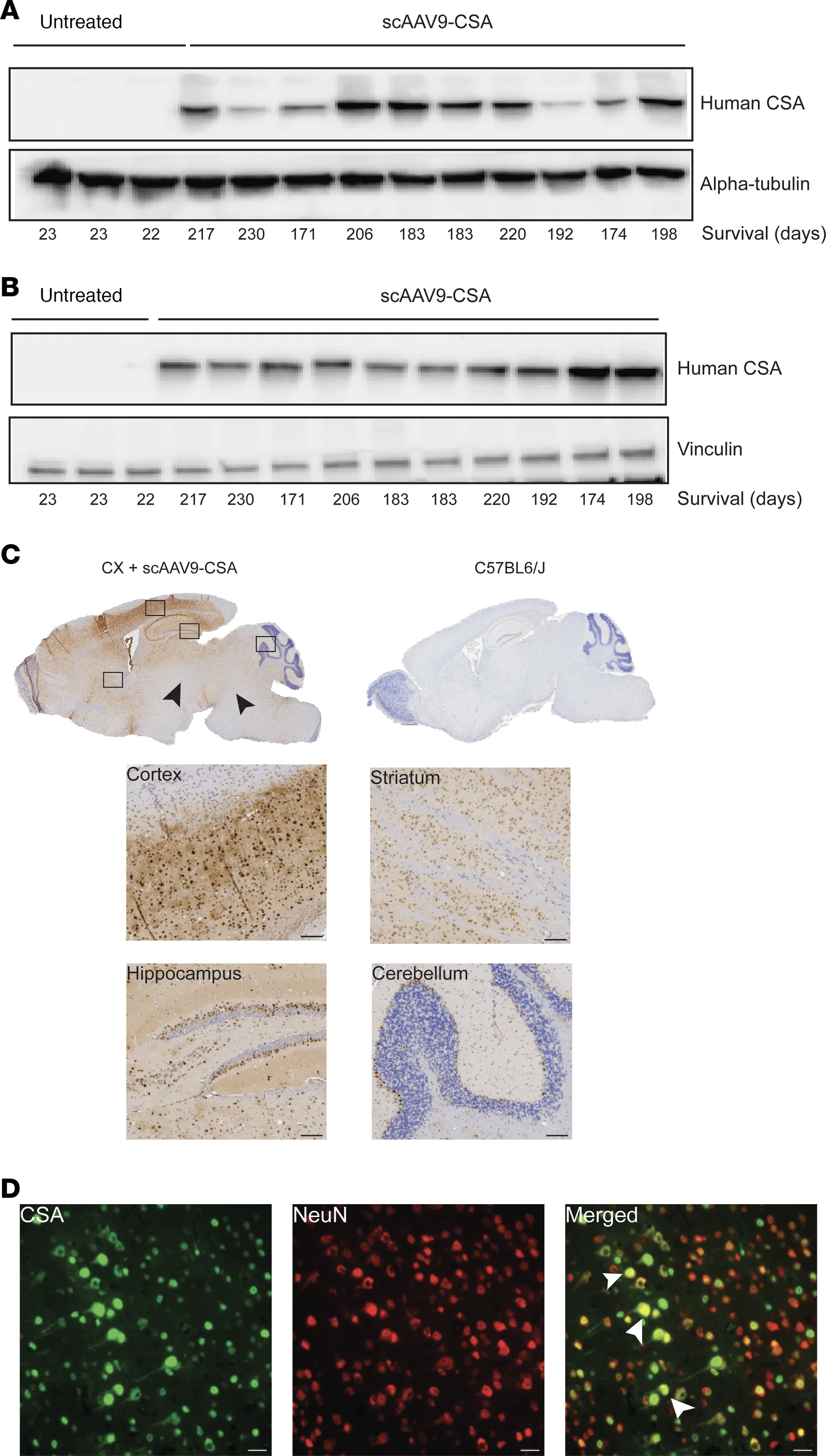
A single neonatal ICV injection of AAV9-CSA results in widespread CSA expression in the brain. CSA protein expression was assessed by Western blot in (**A**) brain and (**B**) heart. vinculin or α-tubulin was used as loading control. Survival age (in days) for each animal is indicated below the blots. (**C**) Representative CSA immunohistochemistry in CX-treated mice. Insets show high-magnification images of the indicated brain regions. Black arrows denote areas of low transduction efficiency. Scale bar: 100 μm. (**D**) Representative immunofluorescence staining for CSA and NeuN in the cortex of AAV-treated CX mice. Colocalization of CSA and NeuN is shown in yellow in the merged image (white arrowheads). Images were acquired as *z*-stacks and displayed as average-intensity projections. Scale bar: 25 μm.

CSA protein expression in the brains and hearts of AAV-treated and control CX mice at endpoint was evaluated by Western blot. We observed variable levels of CSA expression in the brains of AAV-treated CX mice (Figure 4A), which we attribute to variability in ICV injection. Interestingly, the differences in CSA expression levels did not correlate with survival. However, CSA expression in the heart was more consistent across animals, suggesting equivalent leakage of the AAV vector from cerebrospinal fluid (CSF) to the periphery (Figure 4B). Of note, CSA protein expression was not detectable in the kidneys of AAV-treated CX mice of any age (Supplemental Figure 2A). Immunostaining of AAV-treated CX mouse brains showed widespread distribution of CSA-positive cells (Figure 4C), with a higher number of human CSA-expressing cells in the cerebral cortex, hippocampus, striatum, and Purkinje cell layer in the cerebellum, and lower expression in the midbrain and upper cortical layers. Our data also show a predominant neuronal identity of CSA-positive cells in the cortex (Figure 4D).

**Figure 5:**
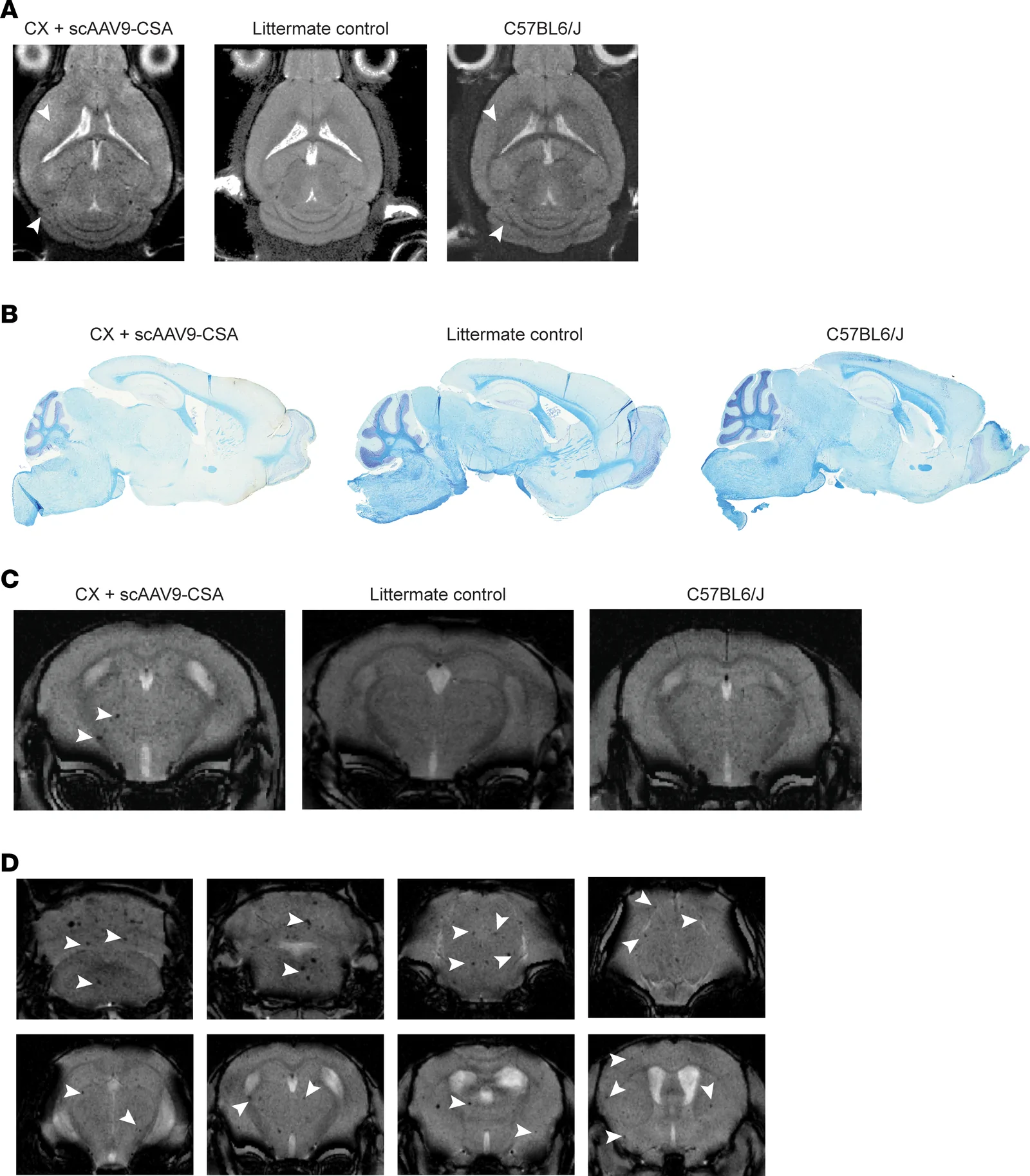
Multimodal brain imaging. (**A**) Representative T2-weighted MRI images of AAV-treated CX mice, age-matched littermate controls, and C57BL6/J mice, demonstrating reduced gray/white matter differentiation in the CX mice. White arrowheads point at regions where white matter is normally visible. (**B**) Luxol Fast blue staining of the same animals shows clear differences in myelination status. (**C**) Susceptibility-weighted imaging (SWI) showing magnetic susceptibility artifacts (MSAs; white arrowheads), suggestive of calcifications or microbleeds. (**D**) SWI images from a single AAV-treated CX mouse showing MSAs (white arrowheads) in multiple regions throughout the brain.

Hallmarks of CS disease in patients include hypomyelination, calcifications, and neuroinflammation in the brain, as well as cranial abnormalities (6, 8, 9, 29, 37). T2-weighted MRI of AAV-treated CX mice at endpoint revealed considerable hypomyelination compared with age-matched controls (Figure 5A). This MRI finding was confirmed by Luxol Fast blue staining of brain sections, showing lower myelin content in AAV-treated CX mice at humane endpoint, compared with littermates and age-matched normal controls (Figure 5B). Additionally, susceptibility-weighted imaging (SWI) revealed magnetic susceptibility artifacts (MSAs) throughout the brain (Figure 5, C and D), consistent with either calcifications or potentially microbleeds from vascular disease (38). However, histological analysis for either bleeds (Prussian blue staining) or calcium (von Kossa staining) proved inconclusive, possibly because of the small size of neuropathological alterations or the challenge of sampling the exact same brain region. Microbleeds and small calcifications in the brain are known to have an outsized effect on SWI, making this technique extremely sensitive for the detection of small local events (38). Also consistent with disease presentation in patients with CS, CT imaging revealed profound kyphosis in untreated CX mice that was not resolved by AAV administration (Supplemental Figure 3, A–D). Kyphosis was a criterion for humane endpoint because of its severity. Additionally, we found evidence of skeletal abnormalities in the form of skull surface pitting in AAV-treated CX mice at humane endpoint (Supplemental Figure 3, E and F).

**Figure 6:**
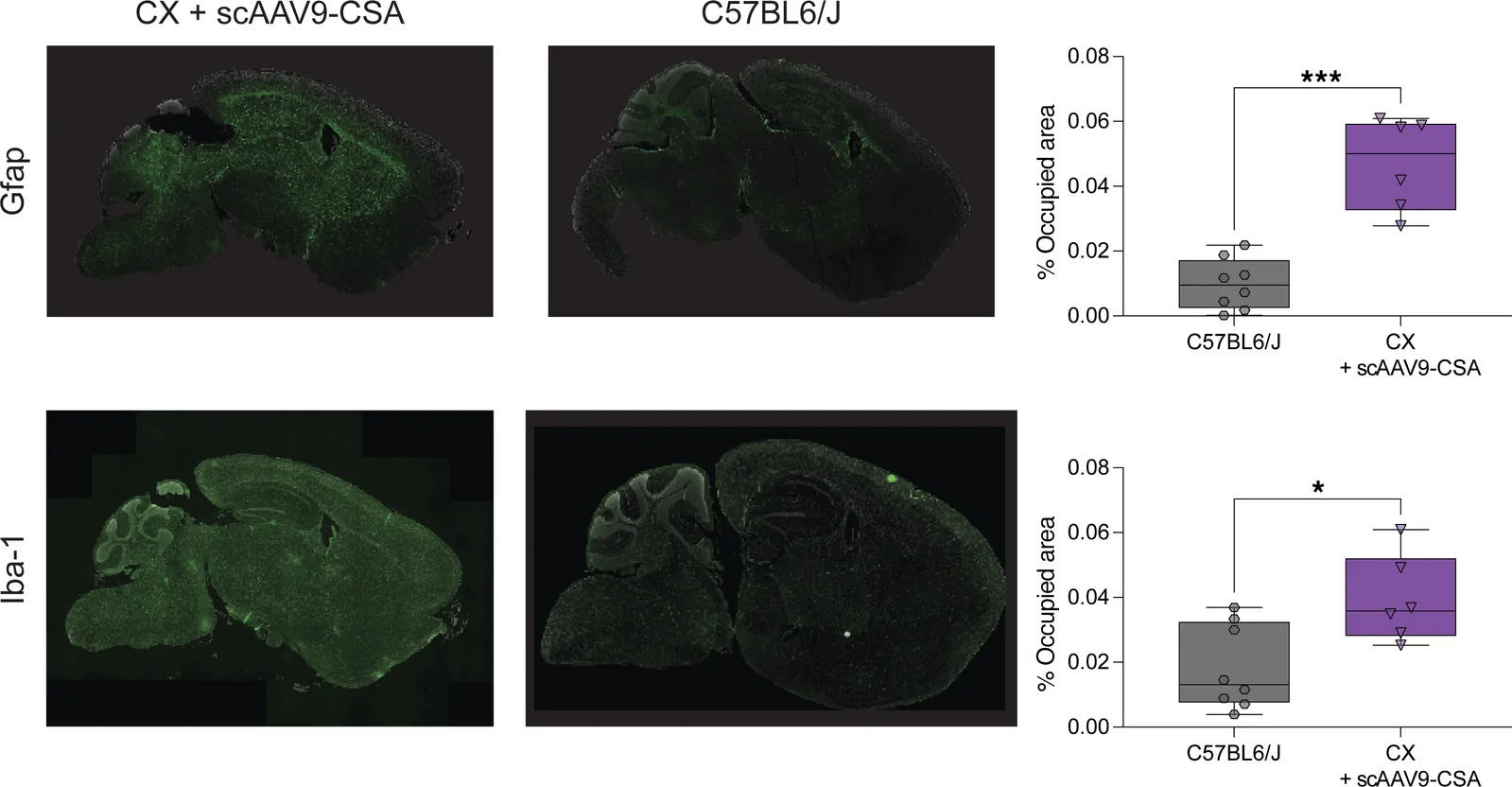
Markers of neuroinflammatory processes. Quantification of immunostaining for glial fibrillary protein (GFAP, top panel) and activated microglia (Iba-1, bottom panel) in AAV-treated CX mice and age-matched C57BL6/J controls. Data are represented as median and interquartile range. Statistical analysis was performed using a 2-tailed unpaired *t* test with Welch’s correction. For GFAP, ***P = 0.0006; for Iba-1, *P = 0.0136.

We also evaluated neuroinflammation by immunofluorescence staining of brain sections with antibodies against markers of reactive astrocytes (glial fibrillary acidic protein, GFAP) and activated microglia (ionized calcium binding adaptor molecule 1, IBA-1). We found both markers to be significantly elevated in AAV-treated CX mice compared with age-matched WT controls (Figure 6).

**Figure 7:**
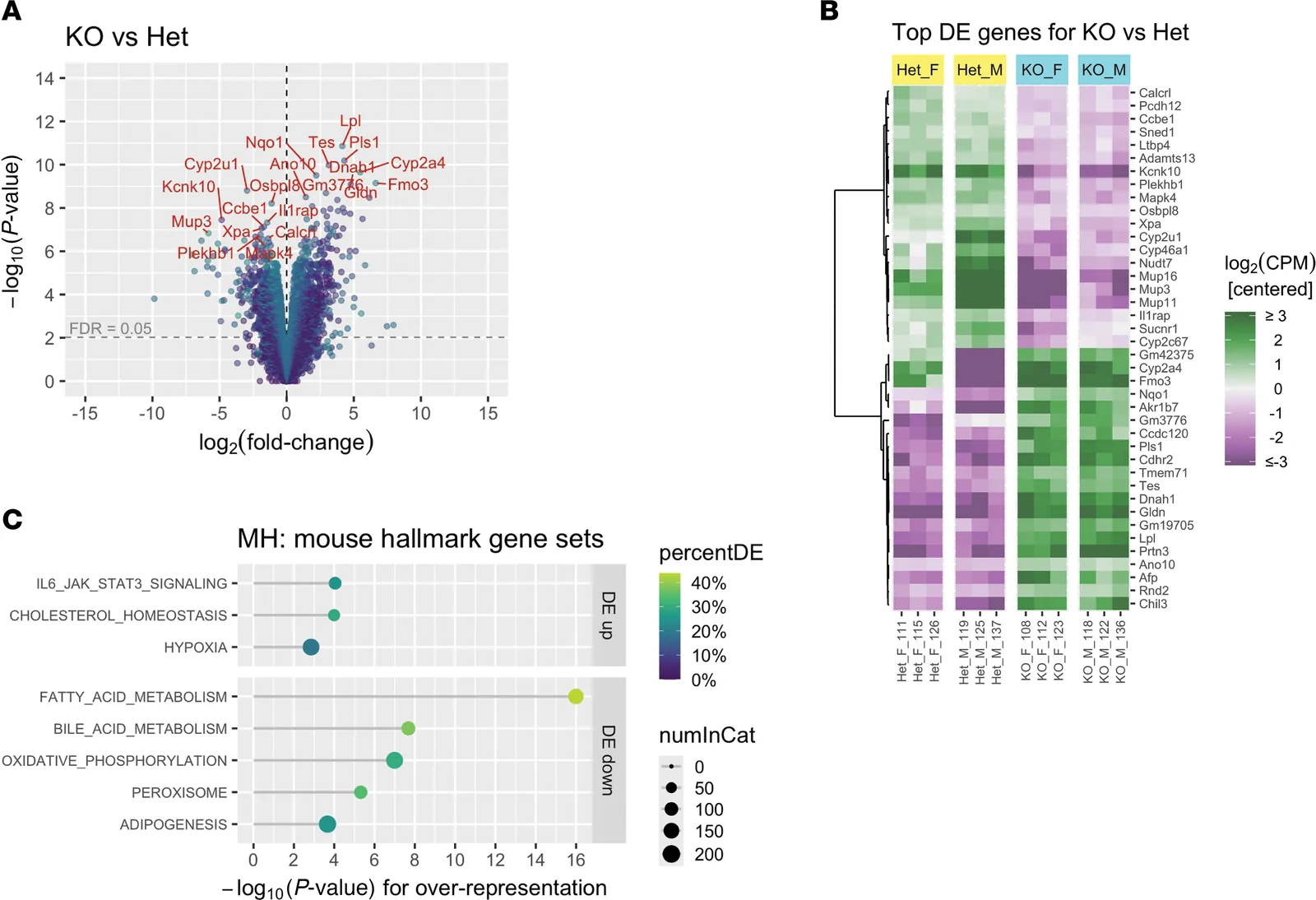
RNA-seq data from mouse liver. (**A**) Volcano plot showing log_2_(fold-change) versus –log_10_(*P* value) for differential gene expression in comparison of KO versus Het mice — the main effect of genotype in a model with factors for genotype and sex and their interaction. Color indicates average log_2_(counts per million [CPM]) across all samples. Dashed horizontal and vertical lines indicate false discovery rate (FDR) = 0.05. (**B**) Heatmap showing the 20 genes in each direction with the smallest *P* values in the KO versus Het comparison from **A**. Expression levels are on a log_2_ scale, centered to have mean 0 for each gene, and capped at ±3 (i.e., 8-fold above or below mean for the gene). (**C**) Categories from the MSigDB mouse Hallmark gene set collection most significantly enriched among the differentially expressed (DE) genes (genes with FDR < 0.05) from the KO versus Het comparison in **A**, separately for DE genes up and down in KO versus Het. Nominal *P* values for enrichment are shown. Size of points indicates the number of genes in the category, and color indicates the percentage of these genes that were DE in the specified direction.

Beyond the neurological manifestations, patients with CS also present with liver dysfunction as indicated by elevation in serum transaminases or cholestatic enzymes (6, 39, 40). We hypothesized that liver dysfunction may have contributed to growth stalling by ~8 weeks of age and to the eventual demise of AAV-treated CX mice due to loss of AAV vector genomes in rapidly dividing tissues, such as the liver, after AAV delivery to neonatal mice (41). To explore this hypothesis, we performed bulk RNA-seq on AAV-treated CX mouse livers at the humane endpoint and their age-matched AAV-treated littermate controls, which showed no signs of disease and had a normal life span (30). In a 2-way experimental design with factors for genotype and sex, there were 3,702 genes differentially expressed between genotypes at a cutoff of FDR < 0.05 (Figure 7A, with heatmap of top hits in Figure 7B). There were 167 genes differentially expressed between sexes at the cutoff FDR < 0.05, with the top 5 hits all consistent with expected sex-related differences: higher levels of 4 genes on chromosome Y (*Kdm5d*, *Uty*, *Ddx3y*, *Eif2s3y*) in male versus female mice and lower levels of *Xist* in male versus female mice. There was little evidence of sex-specific expression differences related to *Xpa* genotype, with only 9 genes having FDR < 0.05 in the test for a genotype × sex interaction (Supplemental Figure 4). The differentially expressed genes that were upregulated in KO versus heterozygous mice were overrepresented in Molecular Signatures Database (MSigDB) Hallmark pathways (42) for IL6/JAK/STAT3 signaling, cholesterol homeostasis, and hypoxia, while those downregulated were overrepresented in metabolic pathways including fatty acid metabolism, bile acid metabolism, oxidative phosphorylation, peroxisome, and adipogenesis (Figure 7C).

**Figure 8:**
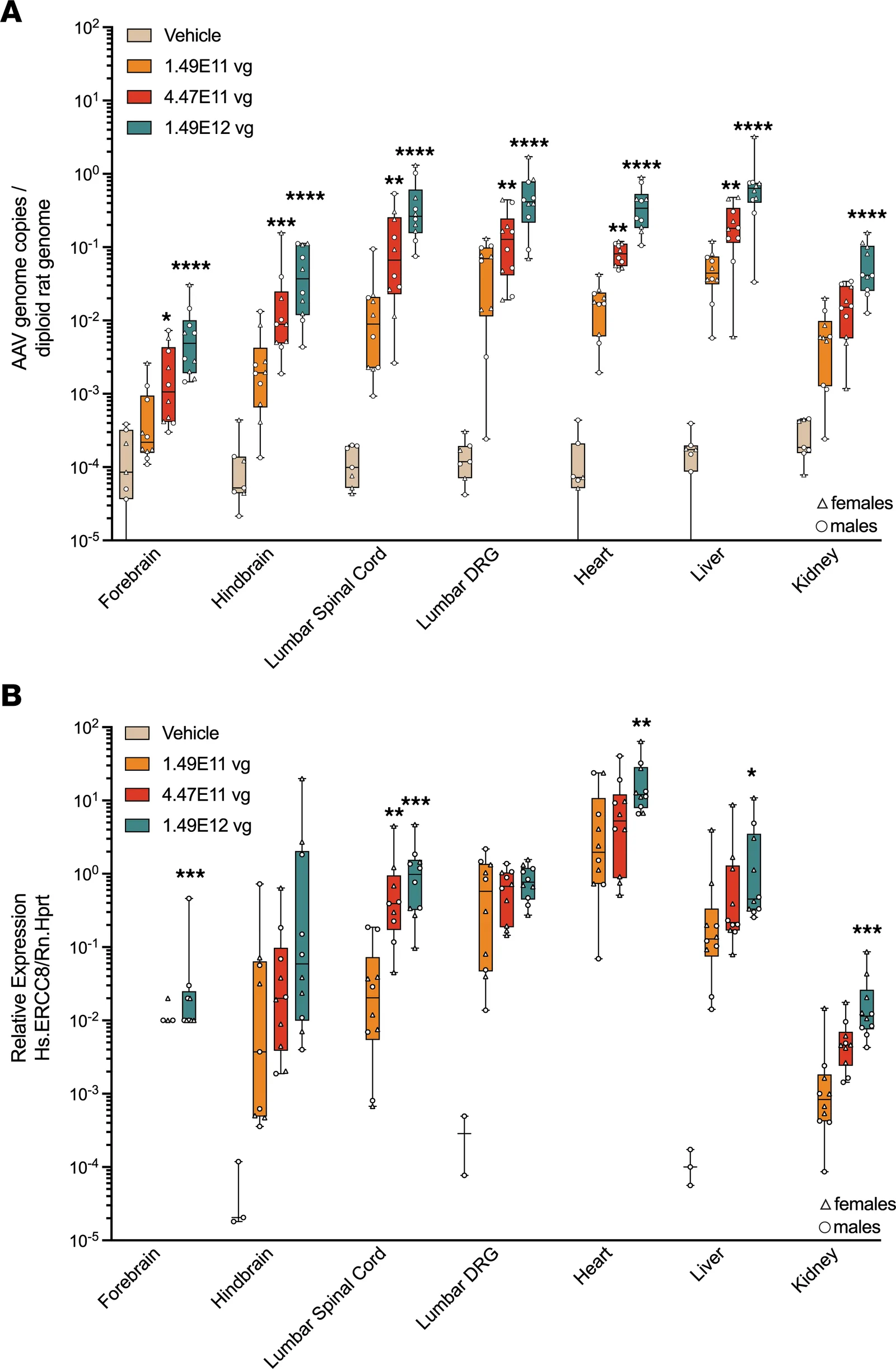
AAV vector biodistribution and transgene expression following intrathecal administration in Sprague-Dawley rats. (**A**) AAV genome copies per diploid rat genome in forebrain, hindbrain, lumbar spinal cord, lumbar dorsal root ganglia (DRG), heart, liver, and kidney analyzed 91 ± 2 days after intrathecal injection of vehicle or AAV9-CSA. (**B**) Relative expression of the human transgene *ERCC8* in the same tissues. Data are presented as box-and-whisker plots (whiskers indicate minimum to maximum values) with individual data points overlaid for female (Δ) and male (○) rats. Statistical significance versus vehicle (biodistribution) or low dose (gene expression) was determined by 1-way ANOVA with Dunn’s (nonparametric) or Dunnett’s (parametric) post hoc tests; *P < 0.05, **P < 0.01, ***P < 0.001, ****P < 0.0001.

In preparation for an *N* = 1 single-patient clinical trial, a good laboratory practice-compliant (GLP-compliant) toxicology study was conducted in Sprague-Dawley rats. Following lumbar intrathecal (IT) administration at different doses, AAV9-CSA vector genomes were detected broadly across tissues in a dose-dependent manner, with no sex-related differences observed (Figure 8A). In the brain, AAV genome distribution showed a caudal-to-rostral gradient, with higher levels in the hindbrain than in the forebrain, consistent with CSF-mediated dissemination from the lumbar IT injection site.

At the highest dose (1.49 × 10^12^ vg), mean brain levels reached approximately 0.03 vg per diploid genome. As expected for IT delivery, the lumbar DRG and lumbar spinal cord exhibited the highest copy numbers across all dose groups, ranging from ~10- to 20-fold higher than brain levels and ~1- to 3-fold higher than those observed in the liver. Vector DNA was also detected in peripheral tissues, with levels comparable to those previously reported for AAV9 following IT administration in rodent and nonhuman primate studies (43).

Concordant with the biodistribution findings, CSA transgene expression was detected in most tissues analyzed in a dose-dependent manner (Figure 8B). Notable exceptions included the forebrain and lumbar DRG. CSA expression in the forebrain was observed only at the highest dose tested, consistent with the caudal-to-rostral CSF flow gradient reflected by vector genome distribution. In contrast, lumbar DRG exhibited relatively uniform CSA expression across all 3 dose levels, suggesting that DRG transduction reached saturation at the site of injection within the dosing range. Collectively, these data demonstrate broad and sustained CSA expression for at least 91 days following IT delivery of AAV9-CSA in rats.

IT administration of AAV9-CSA was well tolerated in male and female rats at all dose levels tested, with no surgery-related complications, mortality, behavioral effects, clinical pathology abnormalities, macroscopic findings, or changes in organ weights. Dose- and time-dependent microscopic findings were observed primarily in the DRG, sciatic nerve, and spinal cord and consisted of minimal to mild neuronal and nerve fiber degeneration, occasional mononuclear infiltration, and glial cell aggregates. These findings were consistent with known, class-associated effects of AAV vectors and were not attributable to transgene-specific toxicity (44, 45). No neuronal degeneration or necrosis was observed at the lowest dose tested (1.49 × 10^11^ vg), which was therefore considered nonadverse and established as the no-observed-adverse-effect level for AAV9-CSA (Supplemental Data 1, report from Charles River Laboratories, 32025165).

## Discussion

In this study, we demonstrate that neonatal CSF administration of AAV9-CSA to an aggressive mouse model of CSA-associated CS results in a marked therapeutic benefit, extending survival by approximately 8.5-fold. To our knowledge, this is the first gene therapy approach for CS and the most substantial therapeutic effect reported to date, highlighting the therapeutic potential for CSA gene replacement.

AAV9-CSA treatment was well tolerated in vivo. No adverse effects were observed in AAV-treated non-CX littermates, and a GLP toxicology study in WT rats identified no major toxicities beyond the expected, low-grade microscopic findings associated with IT AAV9 administration (44, 45) (Supplemental Data 1).

Despite the pronounced survival benefit and widespread CSA expression, AAV-treated CX mice continued to exhibit multiple disease manifestations at endpoint (30, 46). The precise cause of death in AAV-treated CX mice remains unknown but is unlikely to be treatment-related toxicity and instead likely reflects incomplete functional correction in tissues critical for long-term survival.

Longitudinal serum chemistry analyses (Figure 3 and Supplemental Figure 5) revealed progressive multiorgan involvement in the CX mouse model. Renal dysfunction is the dominant endpoint finding in AAV-treated CX mice, likely driven by NAD^+^ depletion via the ATF3/QPRT axis (47). However, analysis of this pathway showed inconclusive results (Supplemental Figure 2, B and C) and therefore invalidated its potential use as a biomarker for preclinical studies using this model.

The natural history of CS in patients includes profound myelination deficits (6, 8). Although there is still debate on whether CS is associated with demyelination or dysmyelination, some studies have characterized it as primarily a hypomyelinating disorder (29). Based on our neuroradiological imaging and histological findings, we were unable to correct myelination with AAV treatment. We believe this is because injection of AAV vectors into P0–P1 mice occurs during a period of rapid oligodendrocyte precursor cell (OPC) proliferation, and oligodendrocyte (OL) differentiation reaches peak levels only at P10–P14. In other words, there would be few mature OLs to transduce at the time of AAV injection, and AAV vector genomes in transduced OPCs would likely be lost through their rapid proliferation between P0 and P7 and thus not transmitted to the mature OLs that differentiate from OPCs at a later stage of development (48). Additionally, there is evidence that the CBA promoter, used in our vector, is inactive in OLs (49). Therefore, correcting the hypomyelination component of CS may require using a different promoter that is functional in OLs. Cell-specific promoters derived from the myelin-associated glycoprotein (*MAG*) (50) or myelin basic protein (*MBP*) (51, 52) genes are effective for AAV-mediated gene expression in both oligodendrocytes and Schwann cells. These promoters are being used in the development of AAV gene therapies for other nervous system diseases (53). However, restoring CSA expression in only a subset of cell types is unlikely to drive a marked therapeutic benefit, as this is a ubiquitously expressed protein fundamental for DNA repair through the TC-NER pathway in any cell type. Combining the use of different promoters with systemic delivery at P10, when OLs are mature, could bypass some of the limitations of our study.

Other neuropathological findings in patients with CS include calcifications, vascular abnormalities (“string vessels”), reactive astrogliosis, and microgliosis (37). These are all features we observed in our AAV-treated CX mice at endpoint. Interestingly, there appears to be a degree of correlation between areas of high astrogliosis (Figure 6) and areas of poor CSA expression (Figure 4C), suggesting that we need to improve the distribution of CSA expression throughout the brain to fully correct the neurological aspects of CS.

Based on our data, it is likely that disease-related physiological dysfunction in other organs contributed to the partial rescue. Some patients with CS may exhibit hepatic dysfunction, as indicated by elevated transaminases, cholestasis, and lipodystrophy (6, 8). The transcriptomic changes documented in the livers of endpoint AAV-treated CX mice showed upregulation of genes in the IL6/JAK/STAT pathway, suggesting an ongoing inflammatory process, which is normally accompanied by increased serum transaminases (Supplemental Figure 5). Interestingly, downregulated genes were in fatty acid and bile acid metabolism pathways as well as adipogenesis pathways. These changes, together with impaired weight gain, suggest that systemic metabolic dysfunction, potentially driven by hepatic stress, contributes substantially to the reduced life span of AAV-treated animals. Evaluation of hepatic therapeutic efficacy following neonatal AAV administration is complicated by hepatocellular turnover, which leads to progressive loss of vector genomes in the liver (41, 54). While this is a limitation of AAV gene therapy studies in neonatal mice, it is less likely to constrain clinical translation.

Modeling the complexity of CS in small rodents has proven challenging, as animal models often fail to reproduce disease phenotypes (28). The CX model used in this study is a double mutant for *Csa* and *Xpa*, both of which are involved in the TC-NER pathway. While this is a severe model that enables faster testing of therapies, we are presently unable to exclude the possible contribution of *Xpa* mutations to the lingering phenotypes observed in AAV-treated CX mice, despite the *Xpa^–/–^* mouse model having no overt phenotype (55, 56). Furthermore, CSA operates as part of a multiprotein machinery requiring coordinated interactions to mediate appropriate stress responses (23, 57). It is therefore possible that expression of the human protein in mouse cells does not fully support proper assembly of repair complexes, which may contribute to the incomplete correction of phenotypes observed in CX mice.

The profound neurological and peripheral symptoms in CS indicate that a transformative AAV gene therapy will require new capsids that target the CNS with higher efficiency than AAV9 while retaining its broad peripheral tissue tropism. The new generation of AAV capsids identified by in vivo or in vitro screening of capsid libraries are orders of magnitude more potent than AAV9 for CNS gene transfer (58–64). Importantly, these new AAV9 variants largely retain the broad peripheral tissue tropism with the added advantage that liver tropism is often tuned down but not altogether eliminated. This is an important consideration when selecting an AAV capsid for the development of a gene therapy for neurological diseases like CS with CNS and peripheral pathology, as exemplified by the apparent liver disease in CX mice.

The second component of a successful gene therapy for broadly expressed genes, such as CSA, is to use promoters with broad functionality that express the therapeutic protein at normal or nearly normal physiological levels. Development of transgene expression cassettes with broad functionality can be achieved using combinations of cell-specific promoters (49) or new promoters with endogenous-like expression (54). In recent years, bioinformatic analysis of single-cell transcriptomic and epigenomic data from mouse and human has been used to develop new enhancer elements with exquisite specificity for different cell populations in CNS (65–69). This same approach may also be applicable to developing small gene-specific promoters capable of expressing the respective therapeutic protein at near-physiological levels, or AAV-compatible promoters derived from housekeeping genes likely to function in most cells. The combination of potent new AAV capsids for CNS gene delivery with the emerging principles of promoter engineering based on single-cell multiomics will be the basis for the development of a second-generation AAV gene therapy with the potential to achieve transformative therapeutic outcomes for CS.

In conclusion, our data support translation of this AAV9-CSA vector into first-in-human trials for the treatment of CSA-linked Cockayne syndrome, given the broad CNS gene transfer and the significant extension in life span, especially in the context of a disease without alternative treatments. Our study also identified targets for improvement that will guide the development of the next generation of AAV-CSA vectors necessary to deliver transformative clinical outcomes for patients with CS.

## Methods

### Sex as a biological variable

All our study groups include male and female animals. Similar findings are reported for both sexes, with no differences in survival or growth rate or transcriptome analysis, between males and females. Therefore, both sexes were combined into a single group for statistical analyses.

### AAV vector design and preparation

The self-complementary AAV vector used in these studies carries a transgene cassette with the WT human *ERCC8/CSA* reference cDNA (NM_000082.4), which encodes the longest isoform of CSA, driven by a CBA promoter composed of the CMV immediate-early gene enhancer upstream of the CBA promoter and followed by an SV-40 intron, and it uses a bovine growth hormone (BGH) polyA signal for polyadenylation. The transgene cassette is flanked by AAV2 inverted terminal repeats (ITRs). The ITR near the CMV enhancer carries a deletion of the terminal resolution sequence for production of self-complementary AAV genomes during packaging.

AAV9 vector was produced, purified, and its titer determined as previously described (70). Throughout this study, several independent batches of vector were required. All titers were normalized to the value of the first batch to ensure consistency of dosing.

### Cell lines and in vitro assays

We generated CSA-deficient HEK293T cells (HEK293T-CSA^–/–^) using CRISPR/Cas9 to disrupt exons 2 and 7 of the *ERCC8* gene. The efficacy of disruption was validated by Western blot of cell lysates, as described below (Supplemental Figure 1). HEK293T (ATCC) and HEK293T-CSA^–/–^ cells were cultured in DMEM (12430-047, Gibco) with 10% FBS (Sigma-Aldrich), 1% GlutaMAX-I (Gibco), and 100 U/mL penicillin/streptomycin (Gibco). Normal human fibroblasts (AG08498) and CSA patient fibroblasts (GM28257) were acquired from the Coriell Institute for Medical Research. Both cell lines were cultured in EMEM (Gibco) supplemented with 15% FBS (Sigma-Aldrich), 1% GlutaMAX-I (Gibco), 1% (v/v) nonessential amino acids (Gibco), and 100 U/mL penicillin/streptomycin (Gibco). All cell lines were routinely propagated as monolayers at 37°C in 5% CO_2_ humidified incubators.

#### Assessing CSA protein expression.

Cells were plated in 12-well plates at a density of 300,000 cells per well (HEK293T) or in 6-well plates at 1,000,000 cells per well (fibroblasts), and 24 hours later they were infected with AAV-CSA or AAV-eGFP (as control) at an MOI of 100,000 vg/cell. Experiments in HEK293T were performed with AAV9 vectors, whereas experiments in fibroblasts used AAV3b vectors. At 72 hours postinfection, cells were assessed for eGFP expression by fluorescence microscopy, lysed in RIPA buffer (50 mM Tris pH 7.4, 140 mM NaCl, 5 mM EDTA, 0.1% SDS, 0.5% sodium deoxycholate, 1% NP-40) with protease inhibitors (cOmplete Mini, EDTA-free, Sigma-Aldrich), and processed for Western blot analysis as described below.

#### Illudin S cell survival assays.

HEK293T-CSA^–/–^ cells were plated in 12-well plates at a density of 300,000 cells per well and transduced 24 hours later with increasing doses (1 × 10^3^ to 3 × 10^5^ vg/cell) of AAV9-CSA, with naive HEK293T-CSA^–/–^ and WT HEK293T cells serving as negative and positive controls, respectively. At 72 hours after transduction, cells were harvested and replated into white 96-well plates at 1,000 cells per well; after an additional 24 hours, cells were treated with Illudin S (4 ng/mL) or vehicle and incubated for 72 hours. Cell viability was quantified using a luminescence-based ATP assay (CellTiter-Glo 2.0, Promega), and survival was calculated by normalizing Illudin S–treated wells to untreated controls.

#### Illudin S killing assays.

All cell lines were plated in 12-well plates (300,000 cells per well) and infected as above. At 36 hours postinfection, cells were trypsinized and replated in 6-well plates at a density of 100,000 cells per well. At 24 hours later, Illudin S (17451, Cayman Chemical) was added to the cells at a concentration of 4 ng/mL (HEK293T) or 2 ng/mL (fibroblasts). Cells were fixed in formalin and stained with crystal violet at 72 hours after adding Illudin S.

### Mouse procedures

*Csa^–/–^ Xpa^+/–^* embryos were donated by Michael MacArthur and Sarah Mitchell (Jay Mitchell group, ETH Zurich, Zurich, Switzerland) and recovered at the UMass Transgenic Mouse Core. Seven-month-old C57BL/6J mice were purchased from The Jackson Laboratory (stock no. 00064). Animals were maintained at 21 ± 1°C under a 12-hour light/dark cycle with water provided ad libitum. Breeding pairs were fed a 10% kcal fat diet (D12450Bi, Research Diets), while all study animals after weaning were provided with regular irradiated laboratory chow.

#### ICV delivery.

P0–P1 pups were anesthetized using isoflurane (drop method) and injected with 1 × 10^11^ vg of AAV9-CSA freehand in the lateral ventricles (2 μL + 2 μL) using a 10 μL glass Hamilton syringe fitted with a 32 G beveled needle. Animals were returned to home cage with their mom and monitored until full recovery.

All animals in the same litter were injected regardless of genotype. Mice were genotyped at weaning. CX mice (*Csa^–/–^ Xpa^–/–^*) were followed until humane endpoint, defined by severe hunched posture and body tremors, at which point they were euthanized with an overdose of ketamine (375 mg/kg) and xylazine (37.5 mg/kg), and whole blood and multiple organs were collected and bisected, with one portion immediately frozen (for molecular analysis) and the other fixed in 10% neutral buffered formalin and embedded for paraffin processing. All non-CX littermates (*Csa^–/–^ Xpa^+/–^* or *Csa^–/–^ Xpa^+/+^*) were used as controls and euthanized in the same manner.

#### Serum chemistries.

Whole blood collected from the vena cava was allowed to coagulate at room temperature for 1–3 hours and then centrifuged at 1,500*g* for 30 minutes. Serum was collected and frozen at –80°C until further analysis. Prior to analysis, sera were thawed, kept refrigerated, and pooled such that each sample represented a combination of 2 animals from the same treatment group. Samples were analyzed using a VetScan Comprehensive Diagnostic Profile reagent rotor (Zoetis).

#### Radiological imaging.

A subset of AAV-treated CX mice (*n* = 5) were subjected to radiological imaging at endpoint before euthanasia, as were age-matched littermate controls (*n* = 8) and normal C57BL6/J mice (*n* = 6). Animals were subjected to both 7 T MRI (Bruker Biospec 70/30) and microCT (VECTor CT, MiLabs). A standard imaging protocol was used for each animal including qualitative imaging in the form of coronal T2 weighted (repetition time [TR]/echo time [TE] 2,500/33 ms, FoV: 15 × 15 mm, matrix: 256 × 256, thickness: 0.5 mm, number of signal averages [NSA]: 4) and SWI (TR/TE: 425/10 ms, FA: 30°, FoV: 20 × 15 mm, matrix: 280 × 210, thickness: 0.5 mm, NSA: 4). Quantitative diffusion imaging in the form of diffusion tensor imaging (TR/TE: 2,500/21 ms, FoV: 18 × 15 mm, matrix: 108 × 90, thickness: 0.8 mm, NSA: 2, directions: 30) was also acquired. After the MRI protocol was completed, the animals were transferred to the microCT system for whole-body computed tomography (20 μm isotropic resolution) to assess bone health and qualitative spinal curvature. For both imaging modalities animals were anesthetized with isoflurane gas (1%–3%) and allowed to spontaneously breathe air with 1.5% isoflurane.

### Histology and image processing

#### Immunohistochemistry.

Four-micrometer-thick sections were deparaffinized in xylene and hydrated in a descendent alcohol series. Heat-induced antigen retrieval was performed using a microwave and sodium citrate buffer (10 mM sodium citrate, pH 6.0). Tissue slides were incubated with blocking buffer containing 5% fetal bovine serum and 5% normal goat serum in phosphate-buffered saline for 1 hour at room temperature and then incubated overnight at 4°C with primary antibodies diluted in blocking buffer. Primary antibody used was rabbit recombinant monoclonal anti-ERCC8 antibody (1:100; ab137033 [EPR9237], Abcam). Endogenous peroxidase activity was quenched with 3% hydrogen peroxide in phosphate-buffered saline for 20 min. Antigen visualization was performed using VECTASTAIN Elite ABC Reagent and DAB Substrate Kit (both from Vector Labs), according to the manufacturer’s instructions. Sections were counterstained with hematoxylin, dehydrated in an ascending alcohol series, and coverslipped using Permount (Thermo Fisher Scientific). Sections were visualized under a Leica Thunder Imager DMi8 microscope equipped with a DMC4500 digital camera.

#### Immunofluorescence.

Four-micrometer-thick sections were deparaffinized in xylene and hydrated in a descendent alcohol series. Heat-induced antigen retrieval was performed using a microwave and sodium citrate buffer (10 mM sodium citrate, pH 6.0). Tissue slides were incubated with blocking buffer containing 5% fetal bovine serum, 5% normal donkey serum, and 0.3 M glycine in phosphate-buffered saline for 1 hour at room temperature. Primary antibodies were diluted in blocking buffer without glycine and then incubated overnight at 4°C. Primary antibodies used were rabbit recombinant monoclonal anti-ERCC8 (1:100; ab137033 [EPR9237], Abcam), rat monoclonal anti-NeuN (1:100; ab177487 [EPR12763], Abcam), rabbit polyclonal anti-GFAP (1:1,000; Z0334, Agilent), and rabbit polyclonal anti-Iba1 (1:1,000; CTJ0605, Wako). Sections were then washed and incubated with secondary antibodies Alexa Fluor 555 anti-rabbit IgG or Alexa Fluor 647 anti-rat IgG (1:1,000; A31572, A21247, Invitrogen) for 1 hour at room temperature. Slides were mounted in VECTASHIELD containing DAPI (Vector Labs) and visualized under a Leica Thunder Imager DMi8 microscope equipped with a Leica K5 digital camera and using the same exposure conditions across groups.

For both staining techniques, parallel sections were incubated with blocking buffer in lieu of primary antibody as staining controls, to assess antibody specificity. Staining was absent under these conditions.

#### Image processing.

All images were processed using either Fiji software (71) or in-house–developed MathWorks MATLAB algorithms, and changes to brightness or contrast or gamma were kept consistent across groups. Tissue sections immunostained for GFAP and IBA-1 expression were analyzed using the same algorithm. The image quantification algorithm is a 2-step automated process: First the images were processed through a Gaussian filter to smooth the noise. These filtered images were then thresholded, and a marching squares algorithm (72) was applied to segment the outer boundary of the brain. The area inside this segmentation defines the brain area. Once the brain area was defined, the original unfiltered image was subdivided into smaller regions for parallel processing. Each subsection was thresholded to detect only the stained cells (GFAP or IBA-1) using an adaptive method that accounts for the local background intensity variations. The total area of all the stained cells was then summed across all subsections. The final result is presented as the percentage area of the brain that is occupied by the stained cells.

### Western blot

For protein expression analysis, tissues were lysed in RIPA buffer containing protease inhibitors, as above. Total protein in the lysates was determined by Bradford assay (Bio-Rad) using serial dilutions of bovine serum albumin as protein standard; 40 μg of total protein was separated in a 4%–20% polyacrylamide SDS-PAGE gel (Bio-Rad) and then transferred to a 0.2 μm pore nitrocellulose membrane (Amersham, GE Healthcare). Primary antibodies were rabbit recombinant monoclonal anti-ERCC8 (1:100; ab137033 [EPR9237], Abcam), mouse monoclonal anti–α-tubulin (1:1,000; T6199, Sigma-Aldrich), or rabbit monoclonal anti-vinculin (1:1,000; ab129002 [EPR8185], Abcam) for normalization. Detection was performed by chemiluminescence using Clarity Western ECL Substrate (Bio-Rad), and images were acquired using a ChemiDoc system (Bio-Rad).

### RNA-seq

Total RNA was isolated using TRIzol (Invitrogen) according to the manufacturer’s instructions. Libraries were prepared with rRNA depletion (TruSeq Stranded Total RNA Ribo-Zero H/M/R Gold kit; Illumina) and sequenced on the NovaSeq X platform by Psomagen with 50 million–70 million 2 × 151 paired-end reads per sample (with 12 samples total: livers from 3 mice per sex per genotype).

The nf-core/rnaseq (v3.14.0) (73) pipeline from the nf-core collection (74) of Nextflow workflows (75) was used for data quality control and preprocessing, for mapping reads with the STAR aligner (2.7.9a) (76) to the mouse GRCm39/mm39 reference genome with GENCODE M34/Ensembl 111 annotations, and for computing estimated read counts per gene per sample with Salmon (1.10.1) (77). The R (v.4.3.2) package edgeR (v.4.0.16) (78) was used to perform quasi-likelihood tests (79) for differential gene expression in a generalized linear model with factors for genotype (KO = *Csa^–/–^ Xpa^+/+^* and Het = *Csa^–/–^ Xpa^+/–^*), sex (M and F), and their interaction (with nondefault parameters prior.count=2 and robust=T for glmQLFit). Genes that did not have at least 5 reads in at least 3 samples (after adjusting for differences in library sizes while keeping median library size fixed) were filtered out prior to edgeR analysis. FDR (80) was used to control for multiple-hypothesis testing. The R package goseq (v.1.54.0) was used for gene set analysis, testing for enrichment of categories from the MSigDB (v2023.2.Mm) (81) among differentially expressed genes (FDR < 0.05), using log_2_(CPM) as a covariate to adjust for potential biases due to gene expression level (82).

### GLP toxicity study in rats

A GLP toxicology study was conducted in normal rats at Charles River Laboratories (Mattawan, Michigan, USA; Testing Facility Study No. 32025165). Male and female Sprague-Dawley rats were randomized into 4 groups (*n* = 10/sex/group) according to the experimental design outlined in Supplemental Table 1. Animals received vehicle or AAV9-CSA by IT administration at 8–10 weeks of age at doses of 1.49 × 10^11^, 4.47 × 10^11^, or 1.49 × 10^12^ vg/rat. Half of the animals (*n* = 5/sex) in the vehicle and highest-dose groups were euthanized 29 ± 1 days postinjection, and all remaining animals were euthanized 91 ± 2 days postinjection. Tissues were collected for toxicological evaluation, biodistribution, and assessment of CSA transgene expression. Complete study details are provided in Supplemental Data 1.

#### Biodistribution.

Genomic DNA was isolated from tissues collected at day 91 (±2) using the DNeasy Blood & Tissue Kit (QIAGEN), according to the manufacturer’s instructions. AAV vector genomes were quantified by qPCR using a primer/probe mix targeting the BGH polyA in the vector (forward primer: CCTCGACTGTGCCTTCTAG; reverse primer: TGCGATGCAATTTCCTCAT; probe:56-FAM/TGCCAGCCA/ZEN/TCTGTTGTTTGCC/3IABkFQ). Vector genome copy numbers were normalized to rat diploid genomic equivalents.

#### Gene expression.

Total RNA was extracted from tissues collected at day 91 (±2) using a combined TRIzol/column purification method. Briefly, samples were homogenized in TRIzol according to the manufacturer’s instructions. Following phase separation with chloroform, the aqueous phase was mixed with an equal volume of 70% ethanol and loaded onto an RNeasy Mini column (QIAGEN). RNA was then purified according to the manufacturer’s protocol, including on-column DNase I digestion, and eluted in RNase-free water. cDNA was synthesized using a mixture of random hexamers and oligo(dT) with the High-Capacity RNA to cDNA Kit (Applied Biosystems). qPCR was performed using PrimeTime Gene Expression Master Mix (IDT) with assays targeting *ERCC8* (Hs.PT.58.1362799, IDT) and *Hprt* (Rn.PT.39a.22214832, IDT). Relative expression was calculated using the ΔΔCt method (83).

### Statistics

All statistical analyses were performed using GraphPad Prism v11 for macOS, aside from RNA-seq analysis described above. Statistical tests on survival experiments were performed using Kaplan-Meier analysis and log-rank (Mantel-Cox) tests. For all other tests, data are plotted as median and interquartile range. Prior to statistical analysis, normality testing was performed. Comparisons between groups that did not fail tests for normality were analyzed with parametric tests. Otherwise, nonparametric tests were used. Significance level (alpha) was set at 0.05 for all analysis. Details on specific analysis are detailed in the figure legends.

### Study approval

Mouse studies were conducted as approved by the UMass Chan Medical School Institutional Animal Care and Use Committee (IACUC) under docket PROT202100108. Rat studies were approved by the Charles River Laboratories (Mattawan, Michigan, USA) IACUC under protocol 32025165.

### Data availability

The RNA-seq data discussed in this publication have been deposited in NCBI’s Gene Expression Omnibus (GEO) (84) and are accessible through GEO Series accession number GSE336535. All other raw data and MATLAB scripts are available upon request. Values for all data points in graphs are reported in the Supporting Data Values file.

## Author contributions

ARB and MSE conceived the study and designed the experimental plan. ACS, MKW, TM, and KOH performed animal procedures. ARB, ACS, WSC, MKW, CMS, SAW, and WSSE processed samples and collected data. ARB analyzed data. ODK analyzed RNA-seq data. RMK performed radiological imaging and staining quantification. ARB and MSE wrote the manuscript. MSE supervised the project.

## Conflict of interest

The authors have declared that no conflict of interest exists.

## Funding support

- Grant from the Riaan Research Initiative (RRI-1212 to ARB).

## Supplementary Material

Supplemental data

Unedited blot and gel images

Supporting data values

## Version 1 07/30/2026

In-Press Preview

## Version 2 09/15/2026

Electronic publication
