# Supplementary material for: AAV-mediated gene therapy demonstrates phenotypic rescue in a mouse model of Cockayne syndrome
Source: J Clin Invest. 2026 Jul 30;136(18):e196689. doi: 10.1172/JCI196689 (PMC13574149; doi:10.1172/JCI196689)
Supplement: Supplementary file 1 [file jci-136-196689-s024.pdf]

Supplemental Figure 1. Generation of HEK293T $CSA^{-/-}$ cell line. HEK293T cells were co-transfected with plasmids expressing Cas9, guide RNAs (gRNAs), and a puromycin resistance cassette to generate CSA knockout clones. (**A**) gRNA sequences used, with exon targeting indicated in the construct ID (e.g., E3 targets exon 3). (**B**) Western blot analysis of CSA expression in puromycin-selected clones. The table shows all the clones and gRNA combinations tested. Clone 6 (highlighted) was selected as the CSA KO line for subsequent experiments. Only 1 of 17 clones tested (clone 2) retained CSA expression.

A

ID	Sequence
E2A	GATGTTGAAAGAATCCACGG
E2B	GGTGTTAATTCCACCGCCG
E3	TGTGTAATAAGATTGTCTGC
E4	CAGTGGTATCCTCATGACAC
E7	GCCAAGATATAGTCATAACG

B

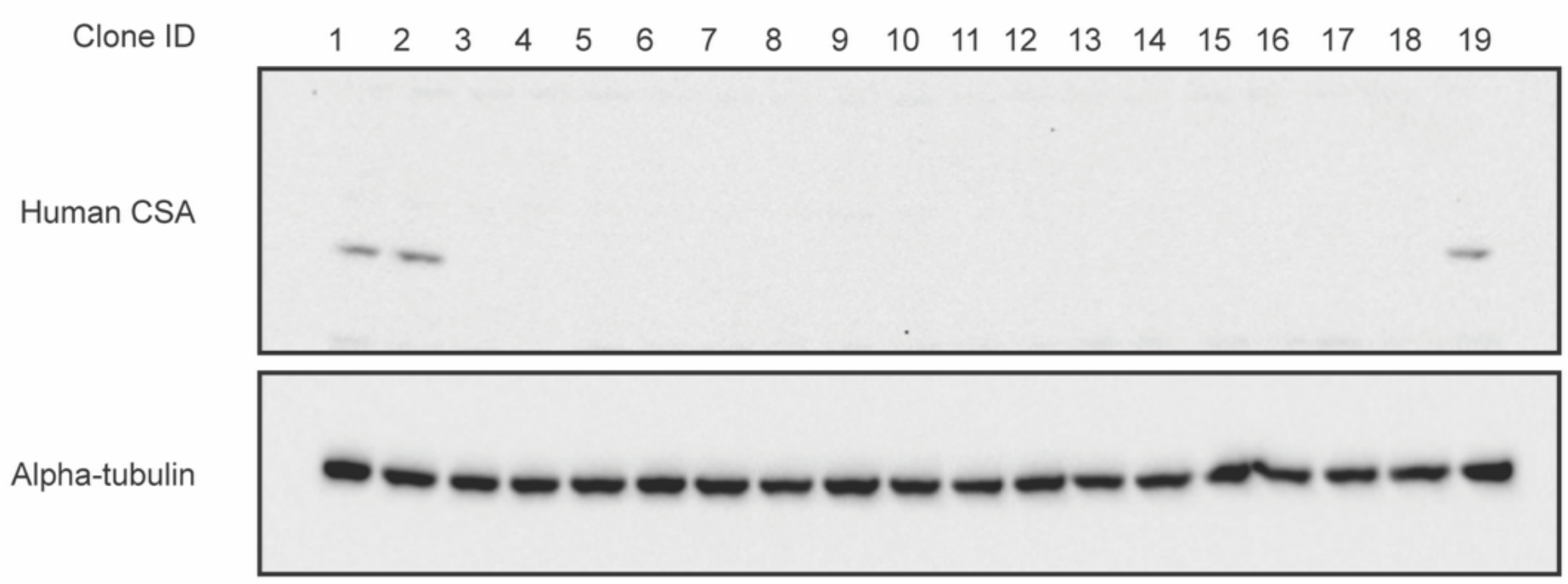

Clone ID	
1	Naïve HEK293T
2	E2A+ E4 B2
3	E2A+E4 C2
4	E2A + E4 C3
5	E2A+E4 D2
6	E2A+E7 A1
7	E2A+ E7 A3
8	E2A + E7 A5
9	E2A+ E7 A6
10	E2A+E7 B1
11	E2A + E7 B2
12	E2A+E7 +B5
13	E2A+E7 C2
14	E2A + E7 C3
15	E2A+E7 C4
16	E2A+E7 C5
17	E2A+E7 D1
18	E2A + E7 D6
19	Naïve HEK293T

Supplementary Figure 2. Assessment of kidney dysfunction. Western blot analysis of protein expression of **(A)** CSA, **(B)** Atf3 and **(C)** Qprt. Vinculin was used as loading control. Samples shown are kidney protein lysates from CX mice (n=4), littermate controls at 22 days of age (n=2), CX + AAV9-CSA at 22 days of age (n=2), CX + AAV9-CSA at endpoint (n=4), littermate controls at endpoint age (n=4), C57BL6/J at 22 days of age (n=4), and C57BL6/J at 8 months of age (n=3 for CSA, n=4 for Atf3 and Qprt). Positive control in (A) is a brain sample from AAV-CX treated mouse previously used in Figure 4.

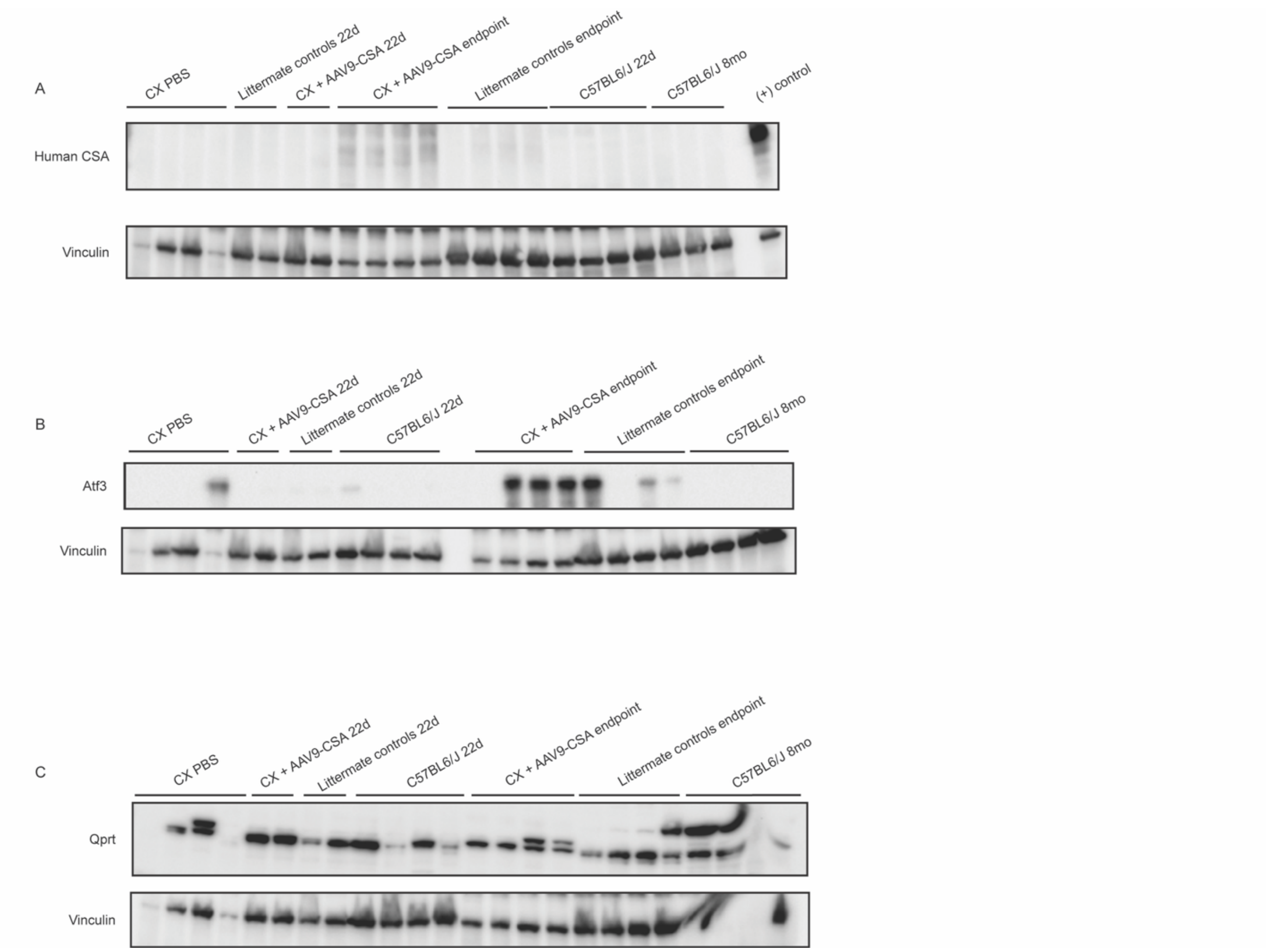

Supplementary Figure 3. <u>CT imaging.</u> Representative maximum intensity projection (MIP) microCT reconstruction of whole-body scans from (**A**) untreated CX mice at endpoint (22 days); (**B**) AAV-treated CX mouse at endpoint (approximately 7 months); (**C**) age-matched littermate controls and (**D**) C57BL6/J controls. Severe kyphosis is evident in (A) and (B). Skull MIP images reveal pronounced surface pitting in (**E**) AAV-treated CX mice at endpoint compared to (**F**) age-matched littermate controls and (**G**) C57BL6/J controls.

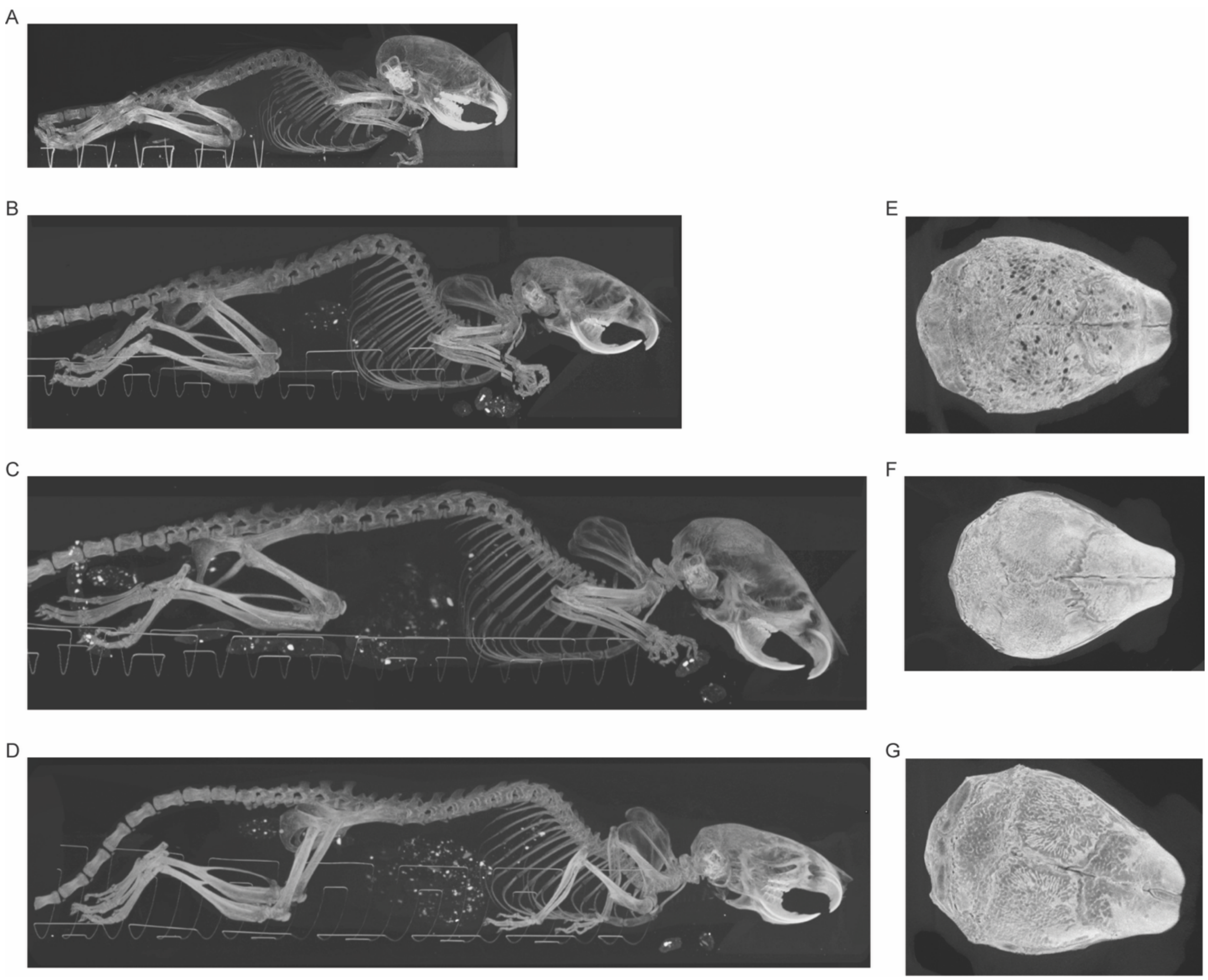

Supplemental Figure 4. RNA-seq data from mouse liver. Volcano plots showing $\log_2$(fold-change) vs $-\log_{10}$(p-value) for differential gene expression in (**A**) male vs female mice, and (**B**) the difference in KO vs Het for male vs female mice (i.e., Xpa genotype x sex interaction). Dashed horizontal lines indicate false discovery rate (FDR) = 0.05; at this cutoff there were 167 in (A), and 9 in (B), out of 19440 genes tested after filtering out genes with very low expression. Note that the top hits for (A) are *Xist* (down in male vs female) and four genes on the Y-chromosome (up in male vs female), consistent with expected sex-related differences, and that despite widespread expression differences associated with *Xpa* genotype there is little evidence for sex-specificity of these differences in (B). (**C**) Heatmap for all the genes in the “hallmark fatty acid metabolism” gene set, the most sig hit from pathway analysis. Expression levels are on a $\log_2$ scale, centered to have mean zero for each gene, and capped at +/-3 (i.e., 8-fold above or below mean for the gene).

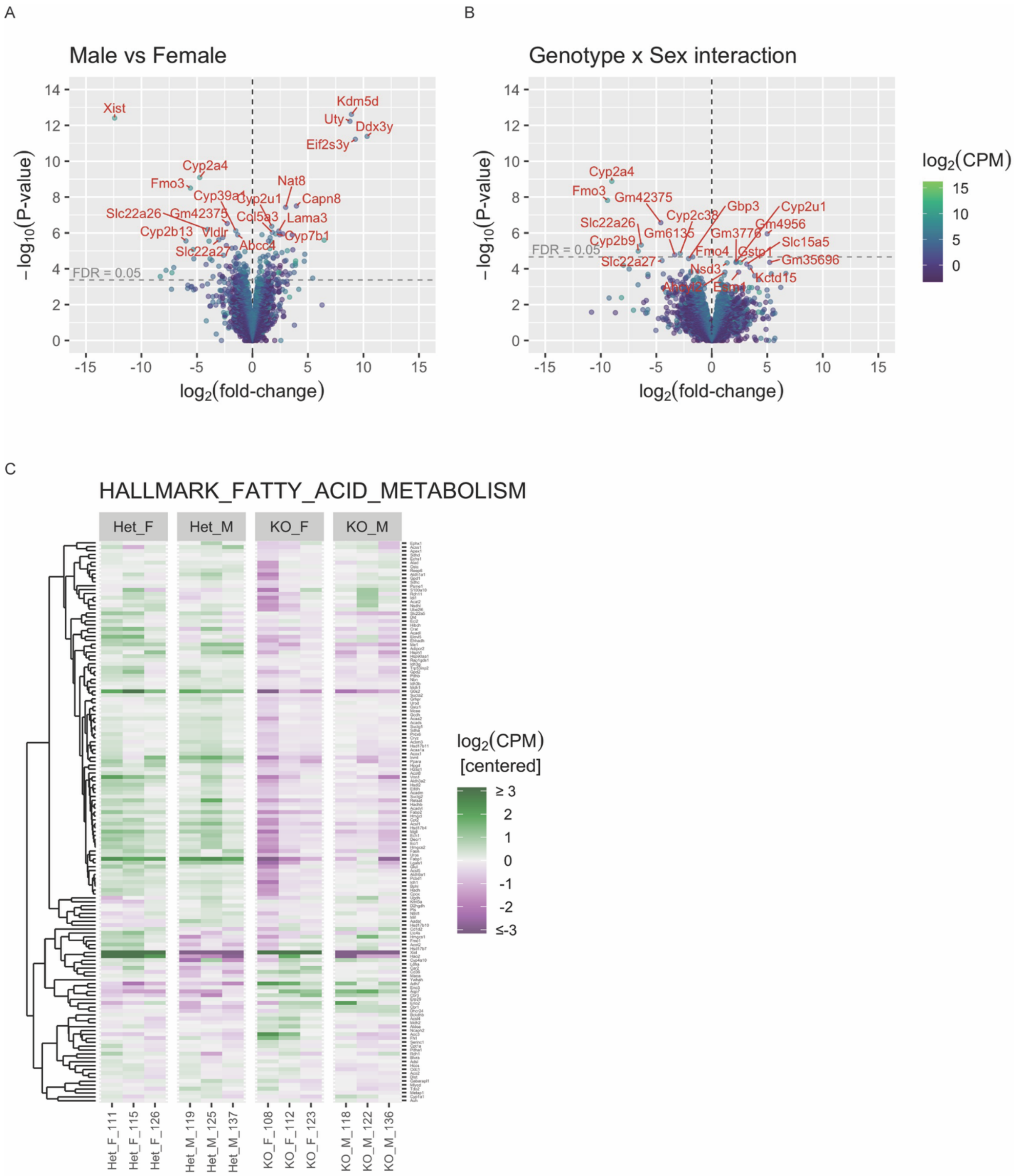
A
Male vs Female
Xist
Kdm5d
Uty
Ddx3y
Eif2s3y
Cyp2a4
Fmo3
Nat8
Cyp39a1
Cyp2u1
Capn8
Slc22a26
Gm42375
Ccl5a3
Lama3
Cyp2b13
Vldlr
Cyp7b1
Abcc4
Slc22a27
FDR = 0.05
−log10(P-value)
log2(fold-change)
B
Genotype x Sex interaction
Cyp2a4
Fmo3
Gm42375
Gbp3
Cyp2u1
Cyp2c38
Slc22a26
Gm4956
Gm6135
Gm3776
Cyp2b9
Slc15a5
Fmo4
Gstp1
Slc22a27
Gm35696
Nsd3
Kctd15
log2(CPM)
C
HALLMARK_FATTY_ACID_METABOLISM
Het_F
Het_M
KO_F
KO_M
log2(CPM) [centered]
≥ 3
≤-3
Het_F_111
Het_F_115
Het_F_126
Het_M_119
Het_M_125
Het_M_137
KO_F_108
KO_F_112
KO_F_123
KO_M_118
KO_M_122
KO_M_136

Supplementary Figure 5. Serum markers of hepatic function. Alanine aminotransferase (ALT) **(A)** and alkaline phosphatase (ALP) **(B)** were measured in CX-treated and untreated mice, littermate controls and wild-type C57BL/6J mice at early (22 days) and endpoint timepoints. Dashed lines and shaded areas denote normal reference ranges for each analyte. Data are presented as mean ± SD with individual data points shown. Each individual data point represents a pooled serum sample from two animals within the same group.

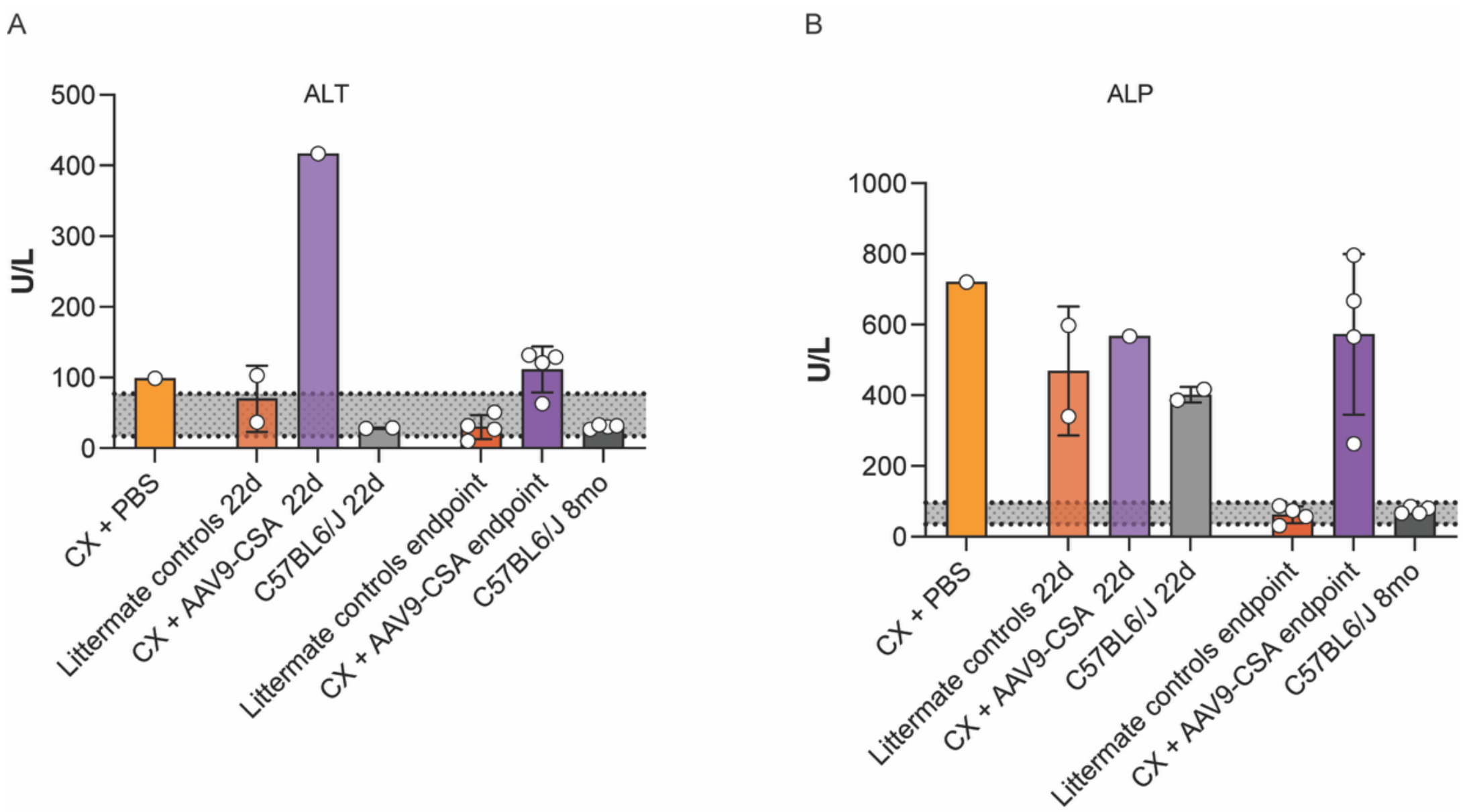

Supplementary Table 1. Study design for the GLP toxicology study in rats.

Group No.	Test Article	Dose Level (vg)	Dose Volume (mL)	Dose Concentration (vg/mL)	Main Study			
					Necropsy Day 29 (±1)		Necropsy Day 91 (±2)	
					No. of Males	No. of Females	No. of Males	No. of Females
1	Vehicle Control	0	0.1	0	5	5	5	5
2	scAAV9-CSA	1.49×10^{11}	0.1	1.49×10^{12}	-	-	10	10
3	scAAV9-CSA	4.47×10^{11}	0.1	4.47×10^{12}	-	-	10	10
4	scAAV9-CSA	1.49×10^{12}	0.1	1.49×10^{13}	5	5	10	10

- = Not applicable; vg = vector genomes.

Draft Report

Testing Facility Study No. 32025165

Sponsor Reference No. 32025165

A 13 Week IT Toxicity Study in Rats with 4-Week and 13-Week Biodistribution

GLP

SPONSOR:
University of Massachusetts
55 North Lake Avenue
Worcester, MA 01605-2324
United States

TESTING FACILITY:
Charles River Laboratories
54943 North Main Street
Mattawan, MI 49071
United States

TABLE OF CONTENTS

LIST OF FIGURES 4
LIST OF TABLES 5
LIST OF APPENDICES 6
QUALITY ASSURANCE STATEMENT 7
COMPLIANCE STATEMENT AND REPORT APPROVAL 8
1. RESPONSIBLE PERSONNEL 9
2. SUMMARY 10
3. INTRODUCTION 12
4. MATERIALS AND METHODS 12
4.1. Test Materials 12
4.1.1. Test Material and Vehicle Characterization 12
4.1.2. Test Material Identification 13
4.1.3. Vehicle Control Identification 13
4.2. Reserve Samples 13
4.3. Test Material Inventory and Disposition 13
4.4. Dose Formulation and Analysis 13
4.4.1. Preparation of Formulations 13
4.4.2. Sample Collection and Analysis 14
4.5. Test System 14
4.5.1. Receipt 14
4.5.2. Justification for Test System and Number of Animals 14
4.5.3. Animal Identification 14
4.5.4. Environmental Acclimation 14
4.5.5. Selection, Assignment, and Disposition of Animals 14
4.5.6. Husbandry 15
4.6. Experimental Design 16
4.6.1. Administration/Application of Test Materials 16
4.6.2. Preoperative Procedures 16
4.6.3. Procedure-Related Medications 16
4.6.4. Surgical Procedure 16
4.6.5. Postoperative Procedures 17
4.6.6. Justification of Route and Dose Levels 17
4.7. In-life Procedures, Observations, and Measurements 17
4.7.1. Neurobehavioral Evaluation 18
4.8. Laboratory Evaluations 19
4.8.1. Clinical Pathology 19
4.8.2. Bone Marrow Smear Evaluation 20
4.8.3. Biodistribution and Anti-drug Antibody Evaluation 20

4.9. Terminal Procedures 22
4.9.1. Unscheduled Deaths....22
4.9.2. Scheduled Euthanasia 22
4.9.3. Necropsy 22
4.9.4. Organ Weights 22
4.9.5. Tissue Collection and Preservation....23
4.9.6. Biodistribution Frozen Tissue Collection 24
4.9.7. Biodistribution Frozen Tissue Sample Analysis....25
4.9.8. Spleen Sample Collection for Isolation of PBMC for ELISpot Assays 25
4.9.9. Histology....25
4.9.10. Microscopic Evaluations....25
5. STATISTICS 25
5.1. Constructed Variables....26
5.2. Descriptive Statistical Analyses....26
6. COMPUTERIZED SYSTEMS AND ELECTRONIC DATA ARCHIVE LOCATIONS....26
7. RETENTION AND DISPOSITION OF RECORDS, SAMPLES, AND SPECIMENS 27
8. RESULTS 27
8.1. Surgical Procedures 27
8.2. Mortality 27
8.3. Clinical Observations....27
8.4. Veterinary Observations 27
8.5. Body Weights and Body Weight Gains....27
8.6. Ophthalmology Examinations 27
8.7. Neurobehavioral Observations....28
8.8. Hematology....28
8.9. Coagulation....28
8.10. Clinical Chemistry 28
8.11. Biodistribution Evaluation....29
8.12. PBMC Analysis 29
8.13. Macroscopic Pathology....29
8.13.1. Terminal Euthanasia Animals (Day 29 (±1) and Day 91 (±2)) 29
8.14. Organ Weights 29
8.14.1. Terminal Euthanasia Animals (Day 29 (±1) and Day 91 (±2)) 29
8.15. Microscopic Evaluations....29
8.15.1. Terminal Euthanasia Animals (Day 29 (±1))....29
8.15.2. Terminal Euthanasia Animals (Day 91 (±2))....30
9. CONCLUSION....32
10. REFERENCES 33

LIST OF FIGURES

Figure 1 Summary of Group Mean Body Weights 34

LIST OF TABLES

Table 1 Summary of Clinical Observations 43
Table 2 Summary of Body Weights 52
Table 3 Summary of Body Weight Gains 56
Table 4 Summary of Neurobehavioral Evaluation: Activity 60
Table 5 Summary of Neurobehavioral Evaluation: Autonomic 72
Table 6 Summary of Neurobehavioral Evaluation: Excitability 84
Table 7 Summary of Neurobehavioral Evaluation: Neuromuscular 96
Table 8 Summary of Neurobehavioral Evaluation: Physiological 108
Table 9 Summary of Neurobehavioral Evaluation: Sensorimotor 114

LIST OF APPENDICES

Appendix 1 Deviations, Amended Protocol, and Protocol 120
Appendix 2 Test and Control Material Characterization 193
Appendix 3 Individual Mortality....210
Appendix 4 Individual Clinical Observations....218
Appendix 5 Individual Animal Exam Observation and Treatment Report....256
Appendix 6 Individual Body Weights....258
Appendix 7 Individual Body Weight Gains....274
Appendix 8 Ophthalmology Report....290
Appendix 9 Individual Neurobehavioral Evaluation: Activity....308
Appendix 10 Individual Neurobehavioral Evaluation: Autonomic....324
Appendix 11 Individual Neurobehavioral Evaluation: Excitability....356
Appendix 12 Individual Neurobehavioral Evaluation: Neuromuscular....372
Appendix 13 Individual Neurobehavioral Evaluation: Physiological....420
Appendix 14 Individual Neurobehavioral Evaluation: Sensorimotor....436
Appendix 15 Clinical Pathology Report....452
Appendix 16 Biodistribution Report....590
Appendix 17 PBMC Analysis Report....591
Appendix 18 Pathology Report....592

COMPLIANCE STATEMENT AND REPORT APPROVAL

The study was conducted in accordance with the U.S. Department of Health and Human Services, Food and Drug Administration, United States Code of Federal Regulations, Title 21, Part 58: Good Laboratory Practice (GLP) for Nonclinical Laboratory Studies and as accepted by Regulatory Authorities throughout the European Union (OECD Principles of Good Laboratory Practice), Japan (MHLW), and other countries that are signatories to the OECD Mutual Acceptance of Data Agreement.

SEND datasets were not subject to QA Audit nor used as the basis for Study Director interpretation of the study results.

Exceptions from the above regulations are listed below.

- The Biodistribution Analysis Report and PBMC Analysis Report are not yet available for inclusion in this report.
- The Pathology Contributing Scientist Report included in this report has not yet been signed.
- The Sponsor has not yet provided documentation of the stability for the batch (lot) of test article or vehicle control used on study.
- The characterization of the test article and vehicle control was performed according to established SOPs, controls, and approved test methodologies to ensure integrity and validity of the results generated; these analyses were not conducted in compliance with the GLP regulations.
- The biodistribution assays and the peripheral blood mononuclear cell (PBMC) analysis were not performed within the scope of regulations governing the conduct of nonclinical laboratory studies and were not intended to comply with such regulations. This did not impact as the study as these methodologies used were well characterized and were conducted and analyzed using sound scientific principles.
- Dose formulation and anti-drug antibody (ADA) analyses were not conducted; samples were collected for possible future analysis. If additional data are obtained from the analysis of these samples, the Study Director will be notified and will assess whether there is an impact to the study, after consultation with the Sponsor. If the Study Director determines there is impact to the study, these data will be included in an Amended Final Report. If the Study Director determines there is no impact, this decision and data will be documented and archived with the study records.

This study was conducted in accordance with the procedures described herein. All deviations authorized/acknowledged by the Study Director are documented in the Study Records.

There were no deviations from the above regulations that affected the overall integrity of the study or the interpretation of the study results and conclusions.

All electronic signatures appear at the end of the document upon finalization.

1. RESPONSIBLE PERSONNEL

Role/Phase	Quality Assurance Unit	Name	Contact Information
Study Director	Charles River	Barak Gunter, PhD	Address as cited for Testing Facility
Testing Facility Management	Charles River	Scott Fountain, PhD	Address as cited for Testing Facility
**Individual Scientists (IS) at Testing Facility**			
Clinical Pathology	Charles River	Danielle Gordon, DVM, MS, DACVP	Address as cited for Testing Facility
Pathology	Charles River	Jeremiah Lyons, MVB, PhD, DACVP	Address as cited for Testing Facility
**Principal Investigator (PI)**			
Biodistribution Analysis	Not Applicable	Miguel Sena-Esteves, PhD	Address as cited for the Sponsor

2. SUMMARY

The objective of this study was to determine the potential toxicity and biodistribution of the test article, scAAV9-CSA, for the treatment of Cockayne Syndrome when given as an intrathecal (IT) injection to rats once on Day 1, and to evaluate the potential reversibility of any findings following a 28-day and 90-day recovery period. The study design was as follows:

Text Table 1
Experimental Design

Group No.	Test Article	Dose Level (vg)	Dose Volume (mL)	Dose Concentration (vg/mL)	Main Study			
					Necropsy Day 29 (±1)		Necropsy Day 91 (±2)	
					No. of Males	No. of Females	No. of Males	No. of Females
1	Vehicle Control	0	0.1	0	5	5	5	5
2	scAAV9-CSA	1.49 $x10^{11}$	0.1	1.49 $x10^{12}$	-	-	10	10
3	scAAV9-CSA	4.47 $x10^{11}$	0.1	4.47 $x10^{12}$	-	-	10	10
4	scAAV9-CSA	1.49 $x10^{12}$	0.1	1.49 $x10^{13}$	5	5	10	10

- = Not applicable; vg = vector genomes.

The following parameters and endpoints were evaluated in this study: mortality, clinical signs, body weights, body weight gains, ophthalmology examinations, neurobehavioral evaluations, clinical pathology parameters (hematology, coagulation, and clinical chemistry), biodistribution evaluation, PBMC analysis, organ weights, and macroscopic and microscopic examinations.

Overall, intrathecal administration of scAAV9-CSA to male and female rats on Day 1 at, 1.49 $x10^{11}$, 4.47 $x10^{11}$, and 1.49 $x10^{12}$ vg was well tolerated.

Administration was not associated with any mortality nor were there any behavioral effects.

There were no scAAV9-CSA-related alterations in clinical pathology parameters at any dose level at any time point.

There were no scAAV9-CSA-related macroscopic findings nor changes in organ weights.

scAAV9-CSA-related microscopic findings at Day 29 (±1) occurred in the lumbar dorsal root ganglia, sciatic nerve, and spinal cord at 1.49 x 10^{12} vg. scAAV9-CSA-related microscopic findings were solely those associated with AAV vector administration (Bennet et al., 2023; Bolon et al., 2024).

The lumbar dorsal root ganglia findings were characterized by minimal neuronal degeneration/necrosis and mononuclear infiltration as well as minimal to mild nerve fiber degeneration.

The sciatic nerve and dorsal roots of the lumbar spinal cord in scAAV9-CSA-treated animals also contained minimal to mild nerve fiber degeneration consistent with the lumbar dorsal root ganglia findings. One sciatic nerve also contained minimal mononuclear infiltration. Minimal nerve fiber degeneration of the dorsal funiculus of the lumbar spinal cord was present in two animals.

scAAV9-CSA-related microscopic findings at Day 91 (±2) were present in the dorsal root ganglia (lumbar, thoracic, and cervical), sciatic nerve, and spinal cord. All microscopic findings were consistent with expected findings associated with the use of AAV vectors (Bennet et al., 2023; Bolon et al., 2024).

Lumbar dorsal root ganglia findings considered related to the administration of scAAV9-CSA included minimal neuronal degeneration/necrosis and minimal to mild glial cell aggregates at $\geq 4.47 \times 10^{11}$ vg. Minimal to moderate nerve fiber degeneration and minimal to mild mononuclear cell infiltration was present at increased incidence and severity in males at $\geq 4.47 \times 10^{11}$ and females at $\geq 1.49 \times 10^{11}$ vg. Neuronal degeneration/necrosis was not identified in animals administered the 1.49×10^{11} dose.

Thoracic dorsal root ganglia findings considered related to the administration of scAAV9-CSA included minimal to mild nerve fiber degeneration at 1.49×10^{12} vg in males and females and in one male at 1.49×10^{11} vg. Cervical dorsal root ganglia had one 4.47×10^{11} vg female with minimal nerve fiber degeneration considered related to scAAV9-CSA administration.

Sciatic nerve findings considered related to the administration of scAAV9-CSA included minimal nerve fiber degeneration at $\geq 1.49 \times 10^{11}$ vg in males and females. Spinal cord findings considered related to the administration of scAAV9-CSA were present mostly in the lumbar and thoracic segments in the dorsal funiculus and dorsal nerve roots. These findings included minimal to mild nerve fiber degeneration at $\geq 4.47 \times 10^{11}$ vg males and $\geq 1.49 \times 10^{11}$ vg females.

Neuronal and nerve fiber degeneration is a common class effect when using AAV vectors and at low severity its functional relevance is determined on an individual case basis (Bolon et al., 2024).

Biodistribution and PBMC analyses will be included at a later date.

Based on the totality of the observations and evaluations, the no-observed-adverse-effect level (NOAEL) of scAAV9-CSA is considered 1.49×10^{11} vg, the lowest dose administered.

3. INTRODUCTION

The objective of this study was to determine the potential toxicity and biodistribution of scAAV9-CSA for the treatment of Cockayne Syndrome when given as an IT injection to rats once, and to evaluate the potential reversibility of any findings following a 28-day and 90-day recovery period.

The design of this study was based on the following guidelines.

- FDA Guidance for Industry. Preclinical Assessment of Investigational Cellular and Gene Therapy Products.
- FDA Guidance for Industry. Human Gene Therapy for Rare Diseases.
- FDA Draft Guidance for Industry. Human Gene Therapy for Neurodegenerative Diseases.
- Final Rules of the Animal Welfare Act regulations (Code of Federal Regulations [CFR], Title 9).
- *Public Health Service Policy on Humane Care and Use of Laboratory Animals* (Office of Laboratory Animal Welfare, Current edition).
- *Guide for the Care and Use of Laboratory Animals* (National Research Council, Current edition).

The deviations, last protocol amendment, and protocol are presented in Appendix 1.

Study Initiation Date:	11 Jul 2025
Animal Arrival:	11 Jul 2025
Initiation of Dosing:	18 Jul 2025
Completion of In-life:	31 Oct 2025
Experimental Starting Date:	11 Jul 2025
Experimental Completion Date:	TBD

4. MATERIALS AND METHODS

4.1. Test Materials

4.1.1. Test Material and Vehicle Characterization

The Sponsor has provided the Testing Facility documentation of the identity, strength, purity, and composition for the vehicle control or test article, but has not yet provided documentation of the stability. Certificates of Analysis were provided to the Testing Facility and are presented in Appendix 2.

The Sponsor has appropriate documentation on file concerning the method of synthesis, fabrication, or derivation of the test article and vehicle control, and this information is available to the appropriate regulatory agencies should it be requested.

4.1.2. Test Material Identification

Text Table 2
Test Article Identification

	Test Article
**Identification:**	scAAV9-CSA
**Alternate Identification:**	Final DP; scAAV9-CB-ERCC8
**Batch/Lot No.:**	PDTP-000102
**Expiration/Retest Date:**	Not available
**Concentration:**	1.49E+13 vg/mL
**Storage Conditions:**	Frozen at -60°C to -90°C and protected from light

4.1.3. Vehicle Control Identification

Text Table 3
Vehicle Control Identification

	Vehicle Control
**Identification:**	Vehicle Control
**Alternate Identification:**	TMN200P
**Batch/Lot No.:**	TMNP0325-B
**Expiration/Retest Date:**	Not available
**Storage Conditions:**	Frozen at -60°C to -90°C and protected from light

4.2. Reserve Samples

For the batch (lot) of test article and vehicle control, a reserve sample was collected and maintained under the appropriate storage conditions by the Testing Facility.

4.3. Test Material Inventory and Disposition

The test materials were received by the Testing Facility for distribution as needed. Records of the receipt, distribution, storage, and disposition of test materials (including empty containers of Sponsor-provided materials) were maintained.

4.4. Dose Formulation and Analysis

4.4.1. Preparation of Formulations

Dose formulations were divided into aliquots where required to allow to be dispensed on each dosing occasion.

Text Table 4
Preparation Details

Component	Preparation	Frequency	Storage Conditions
Dose Formulations/Test Material	Diluted in the vehicle	Once	Refrigerated (2 to 8°C)

Any partial vials of stock test article were stored refrigerated (2°C to 8°C) following thawing and preparation. Any residual dose formulations from each dosing occasion were stored refrigerated (2°C to 8°C) until shipped to the Sponsor on cold packs with a temperature monitor.

Dosing formulations were prepared at appropriate concentrations to meet dose level requirements.

4.4.2. Sample Collection and Analysis

Dose formulation samples were collected for analysis as indicated in the following table.

Text Table 5
Dose Formulation Sample Collection Schedule

Sample Type	Groups	Stratum	No. of Samples Collected per Concentration	Sample Volume (mL)	Intervals
Concentration[a,b]	2 and 3	Middle	2	0.1	Day 1

[a] The samples were stored refrigerated at 2°C to 8°C pending shipment.
[b] Appropriate samples were collected using a positive displacement pipette and placed into sterile polypropylene containers.

All samples were shipped (on refrigerated packs with a temperature monitor) to the UMass Chan Medical School, Worchester, MA for possible future analysis.

4.5. Test System

4.5.1. Receipt

Rats were received from Charles River Laboratories, Raleigh, NC. The animals were 8 to 10 weeks old and weighed between 183 and 418 g at the initiation of dosing.

4.5.2. Justification for Test System and Number of Animals

At this time, studies in laboratory animals provide the best available basis for extrapolation to humans and are required to support regulatory submissions. Acceptable models that do not use live animals currently do not exist.

The rat was chosen as the animal model for this study as it is an accepted rodent species for nonclinical toxicity testing by regulatory agencies.

The total number of animals used in this study was considered the minimum required to properly characterize the effects of the test article. This study was designed such that it did not require an unnecessary number of animals to accomplish its objectives. Details on the group distribution, number of test groups, and number of animals per group are included in Section 4.6.

4.5.3. Animal Identification

Method:	Each animal was implanted with a microchip bearing a unique identification number. The animal cage was identified by the study number, animal number, group number, and sex.

4.5.4. Environmental Acclimation

During the 7- to 20-day acclimation period, all animals were observed daily for any clinical signs of disease, and all animals were given a detailed clinical examination the day prior to dose administration.

4.5.5. Selection, Assignment, and Disposition of Animals

Animals were randomly assigned to groups upon receipt.

The disposition of all animals was documented in the study records.

4.5.6. Husbandry

4.5.6.1. Housing

Housing:	Socially housed.<br>Housing set-up was as specified in the USDA Animal Welfare Act (9 CFR, Parts 1, 2, and 3) and as described in the *Guide for the Care and Use of Laboratory Animals* (NRC, current edition).
Caging:	Solid bottom cages with nonaromatic bedding.

4.5.6.2. Animal Enrichment

Supplemental Enrichment:	Animals were provided enrichment items as per site standard procedures.

4.5.6.3. Environmental Conditions

Temperature:	67°F to 75°F (19°C to 24°C)
Humidity:	40% to 72%

The actual temperature and humidity were occasionally outside the target range defined in the protocol; the variability was within acceptable allowance.

Light Cycle:	Target 12 hours light and 12 hours dark.

4.5.6.4. Food

Diet:	The basal diet was block Lab Diet® Certified Rodent Diet #5CR4.
Frequency:	Ad libitum.
Analysis:	Results of analysis for nutritional components and environmental contaminants are on file at the Testing Facility.<br>There are no known contaminants in the food that would interfere with this study.

4.5.6.5. Water

Type:	Tap water, treated and suitable for consumption.
Frequency:	Ad libitum via an automatic watering system (for exceptions, see Appendix 1).
Analysis:	It is considered that there are no known contaminants in the water that would interfere with the outcome of the study.

4.5.6.6. Veterinary Care

Veterinary care was available throughout the course of the study, and animals were examined by the veterinary staff as warranted by clinical signs or other changes. All veterinary examinations and recommended therapeutic treatments were documented in the study records and reviewed by

the Study Director. The medical treatments and observations recorded after initiation of dosing are presented in Appendix 5.

4.6. Experimental Design

Text Table 6
Experimental Design

Group No.	Test Article	Dose Level (vg)	Dose Volume (mL)	Dose Conc. (vg/mL)	Main Study Animal Nos.			
					Necropsy Day 29 (±1)		Necropsy Day 91 (±2)	
					Males	Females	Males	Females
1	Vehicle Control	0	0.1	0	1001 to 1005	1501 to 1505	1006 to 1010	1506 to 1510
2	scAAV9-CSA	1.49×10^{11}	0.1	1.49×10^{12}	-	-	2001 to 2010	2501 to 2510
3	scAAV9-CSA	4.47×10^{11}	0.1	4.47×10^{12}	-	-	3001 to 3010	3501 to 3510
4	scAAV9-CSA	1.49×10^{12}	0.1	1.49×10^{13}	4001 to 4005	4501 to 4505	4006 to 4015	4506 to 4515

- = Not applicable, Conc. = Concentration; vg = Vector genomes.

4.6.1. Administration/Application of Test Materials

Route of Administration/ Application:	Intrathecal Injection
Treatment Frequency/Duration:	Once on Day 1
Details:	Post-acquisition, test article was maintained on wet ice. Prior to dosing the vehicle control or test article was acclimated to ambient temperature.<br>An equipment list containing information regarding the dosing apparatus was maintained in the study records (see Appendix 1).

4.6.2. Preoperative Procedures

Preoperative procedures were performed in accordance with Testing Facility SOP. Animals were not fasted overnight prior to surgery.

4.6.3. Procedure-Related Medications

Procedure-related medications were administered according to Testing Facility SOP and as needed at the discretion of the surgeon and/or veterinarian; the administration of these medications is maintained in the study file.

4.6.4. Surgical Procedure

A 27-gauge custom LASI needle and an appropriately sized gas tight syringe was used to administer the vehicle control and test article (see Appendix 1). Prior to dosing, the dosing system was primed with vehicle control or test article, as applicable. After the system was primed, the appropriate amount of vehicle or test article was drawn up. The system was not re-primed between animals. A new needle was used for each group, and the priming steps were repeated when a new needle was used. The injection site location was documented in the study data. The duration of dose was 60 seconds ±5 seconds.

4.6.5. Postoperative Procedures

Postoperative procedures were performed in accordance with Testing Facility SOP.

4.6.6. Justification of Route and Dose Levels

Intrathecal injection is one of the intended routes of administration of potential routes of human exposure to this test article.

The dose levels were selected based on information provided by the Sponsor, references, and in an attempt to produce graded responses to the test article. The high-dose level should have produced some toxic effects, but not excessive lethality that would have prevented meaningful evaluation. The mid-dose level was expected to produce minimal to moderate toxic effects. The low-dose level should have produced no observable indications of toxicity.

4.7. In-life Procedures, Observations, and Measurements

Text Table 7
General In-life Assessments – Main Study Animals

Parameter	Population(s)	Frequency (minimum required)	Comments
Mortality/Cageside Observations	All Main Study Animals	Twice daily (morning and afternoon) beginning upon arrival through termination/release (see Appendix 1). [a,b]	Animals were observed within their cage unless necessary for identification or confirmation of possible findings.<br><br>Animals were observed for morbidity, mortality, injury, and availability of food and water. Any animals in poor health were identified for further monitoring and possible euthanasia.
Post Dose Observations	All Main Study Animals	Approximately 4 and 24 hours postdose.[c]	Animals were observed within their cage unless necessary for identification or confirmation of possible findings.
Detailed Clinical Observations	All Main Study Animals	At receipt on Day -1, and once weekly throughout the study.[c]	Animals were removed from the cage.<br><br>The examinations performed at receipt are not reported but are maintained in the study file.
Individual Body Weights	All Main Study Animals	At receipt on Day -1, and once weekly during the study.	The body weights recorded at receipt are not reported but are maintained in the study file.
Food Consumption	All Main Study Animals	Twice daily.[d]	Qualitative assessment was conducted as a part of the twice daily cageside observations.
Ophthalmology	All Main Study Animals	Pretreatment (all animals including source) and all animals designated for the Day 29 necropsy were examined prior to necropsy (see Appendix 1).	The ophthalmoscopic examinations were conducted by a staff ophthalmologist via indirect ophthalmoscopy.
Neurobehavioral Evaluation	All Main Study Animals	Day -1 and prior to each necropsy. Animals designated for the Day 91 (±2) necropsy were also evaluated approximately 4 weeks after dosing.	See details in section below.

[a] Included alternate animals until released from study.

[b] Except on days of receipt and necropsy where frequency was once daily.
[c] For observations that could not be attributed to an individual animal due to social housing (e.g., watery feces), the observation was noted for each animal in the socialized group.
[d] For observations of reduced appetite that could not be attributed to an individual animal due to social housing, the observations were noted for each animal in the socialized group.

4.7.1. Neurobehavioral Evaluation

Neurobehavioral evaluations were conducted on all animals without knowledge on the part of the testers of the treatment groups (blinded evaluations). Personal protective equipment was not changed between groups during dose administration when animals were dosed in random order due to blinding. Each animal was observed for 2 minutes in an opaque open-field observation box. Parameters were evaluated per Testing Facility SOPs and study specific procedures. The following evaluations were conducted:

Parameter Observed	Type of Data	Rating Scale or Unit of Measurement
Posture/Body carriage	Categorical	-2 to 0
Convulsions	Categorical	0 to 4
Stereotypy	Categorical	0 to 4
Tremor	Categorical	0 to 3
Palpebral closure/Ptosis	Categorical	0 to 2
Ease of removal	Categorical	-1 to 2
Handling reactivity	Categorical	-1 to 2
Rearing	Continuous	Counts
Arousal/Alertness	Categorical	-2 to 2
Gait/Mobility	Categorical	0 to 3
Vocalizations	Categorical	0 to 1
Respiration	Categorical	-1 to 2
Defecation	Categorical	-1 to 1
Appearance	Categorical	0 to 2
Lacrimation	Categorical	0 to 2
Salivation	Categorical	0 to 2
Exophthalmus	Categorical	0 to 1
Erected fur	Categorical	0 to 1
Touch response/Tactile reflex	Categorical	-1 to 1
Startle response	Categorical	-1 to 1
Thermal response	Continuous	Time in seconds
Pupil response	Categorical	0 to 2
Body temperature	Continuous	℃
Body tone	Categorical	-1 to 1
Air righting reflex	Categorical	0 to 2
Grip strength (forelimb and hindlimb)	Continuous	kg
Hindlimb splay	Continuous	Distance in centimeters

4.8. Laboratory Evaluations

4.8.1. Clinical Pathology

4.8.1.1. Sample Collection

Samples were collected according to Text Table 8.

Text Table 8
Clinical Pathology Sample Collection

Group No.	Time Point(s)	Hematology	Coagulation	Clinical Chemistry
All animals	Prior to the Day 29 (±1) and Day 91 (±2) necropsy	X	X	X
**Method/Comments:**		Cardiac puncture after carbon dioxide inhalation	Cardiac puncture after carbon dioxide inhalation	Cardiac puncture after carbon dioxide inhalation
**Target Volume (mL):**		0.25	1.2	1
**Anticoagulant:**		K_2EDTA	Sodium Citrate	None
**Special Requirements:**		-	-	-
**Processing:**		None	Plasma	Serum

X = Sample was collected; - = Not applicable.

4.8.1.2. Hematology

Blood samples were analyzed for the parameters specified in Text Table 9.

Text Table 9
Hematology Parameters

Red blood cell count<br>Hemoglobin concentration<br>Hematocrit<br>Mean corpuscular volume<br>Red blood cell distribution width<br>Mean corpuscular hemoglobin concentration<br>Mean corpuscular hemoglobin<br>Reticulocyte count (absolute and percent)<br>Platelet count	White blood cell count<br>Neutrophil count (absolute and percent)<br>Lymphocyte count (absolute and percent)<br>Monocyte count (absolute and percent)<br>Eosinophil count (absolute and percent)<br>Basophil count (absolute and percent)<br>Other cells (as appropriate)<br>Blood smear (preserved and stained)

4.8.1.3. Coagulation

Blood samples were processed for plasma, and plasma was analyzed for the parameters listed in Text Table 10.

Text Table 10
Coagulation Parameters

Prothrombin time<br>Activated partial thromboplastin time	Fibrinogen<br>Sample quality

4.8.1.4. Clinical Chemistry

Blood samples were processed for serum, and the serum was analyzed for the parameters specified in Text Table 11.

Text Table 11
Clinical Chemistry Parameters

Alkaline phosphatase<br>Total bilirubin<br>Aspartate aminotransferase<br>Alanine aminotransferase<br>Gamma glutamyl transferase<br>Sorbitol dehydrogenase<br>Urea nitrogen<br>Creatinine<br>Total protein	Albumin<br>Globulin (calculated)<br>Albumin/globulin ratio (calculated)<br>Glucose<br>Total cholesterol<br>Triglycerides<br>Electrolytes (sodium, potassium, chloride)<br>Calcium<br>Phosphorus<br>Sample quality

4.8.2. Bone Marrow Smear Evaluation

Bone marrow smears were collected and prepared as described in the Tissue Collection and Preservation table (Section 4.9.5.). Bone marrow smears were not evaluated.

4.8.3. Biodistribution and Anti-drug Antibody Evaluation

4.8.3.1. Biodistribution Sample Collection

Samples were collected according to Text Table 12.

Text Table 12
Biodistribution Sample Collection Schedule

Group No.	Target Postdose Collection Intervals – Day 29 (±1) and 91 (±2)
	Terminal Necropsy
All	X

X = Sample was collected.

4.8.3.2. Biodistribution Sample Processing

Collection Method: Cardiac puncture after carbon dioxide inhalation

Target Volume: 1 mL

Anticoagulant: K_2EDTA

Fasting: The animals were not fasted before blood collection.

Whole Blood Storage: Wet ice (see Appendix 1).

Processing: Whole Blood

The samples were divided into 2 approximately equal aliquots and flash frozen on dry ice within 4 hours of collection.

Additional Container Label Requirements: Study number, animal number, matrix of sample, interval and timepoint, analysis type, and aliquot number.

Final Storage Temperature: Frozen (-60°C to -90°C)

4.8.3.3. Biodistribution Sample Analysis

Analysis Performed By:	The samples were shipped on dry ice with a temperature monitor to UMass Chan Medical School, Worchester, MA, and analyzed for AAV9-scAAV9-CSA. All analytical work was conducted by a Sponsor-contracted laboratory, UMass Chan Medical School, using an analytical method developed and qualified by that laboratory.

4.8.3.4. ADA Sample Collection

Samples were collected according to Text Table 13.

Text Table 13
TK Sample Collection Schedule

Group No.	Target Postdose Collection Intervals – Day 29 (±1) and 91 (±2)
	Terminal Necropsy
All	X

X = Sample was collected.

4.8.3.5. ADA Sample Processing

Collection Method:	Cardiac puncture after carbon dioxide inhalation
Target Volume:	1 mL
Anticoagulant:	None, Serum Gel Separator
Fasting:	The animals were not fasted before blood collection.
Whole Blood Storage:	Ambient until centrifuged (see Appendix 1).
Processing:	Serum<br>The final processed samples were divided into 2 approximately equal aliquots.
Additional Container Label Requirements:	Study number, animal number, matrix of sample, interval and timepoint, and analysis type, and aliquot number.
Final Storage Temperature:	Frozen (-60°C to -90°C)

4.8.3.6. ADA Sample Analysis

Analysis Performed By:	The samples were shipped on dry ice with a temperature monitor to UMass Chan Medical School, Worchester, MA, and saved for possible future analysis.

4.9. Terminal Procedures

Terminal procedures are summarized in Text Table 14.

Text Table 14
Terminal Procedures

Main Study Animals

Group No.	Scheduled Euthanasia Day	Necropsy Procedures				Histology Processing	Microscopic Evaluation
		Necropsy	Tissue Collection		Organ Weights		
			Fixed	Frozen			
All	Day 29 (±1) and 91 (±2)	X	Full List[a]	Full List[a]	Full List[a]	Full List[a]	Full List[a]

Terminal Procedure Tables Footnotes:
X = Procedure conducted.
'Histology Processing'= embedded in paraffin, sectioned, mounted on glass slides, and stained with hematoxylin and eosin.
[a] Protocol-designated tissues were collected, weighed, processed, and evaluated as applicable to each procedure.

4.9.1. Unscheduled Deaths

No animals died during the course of the study.

4.9.2. Scheduled Euthanasia

Main study surviving until scheduled euthanasia were weighed and euthanized by carbon dioxide inhalation followed by perfusion.

4.9.3. Necropsy

Main study animals were subjected to a complete necropsy examination, which included evaluation of the carcass and musculoskeletal system; all external surfaces and orifices; cranial cavity and external surfaces of the brain; and thoracic, abdominal, and pelvic cavities with their associated organs and tissues.

Necropsy procedures were performed by qualified personnel with appropriate training and experience in animal anatomy and gross pathology. A veterinary pathologist, or other suitably qualified person, was available.

Following euthanasia, animals were perfused with chilled 0.9% sterile saline (for at least 5 minutes or until fluid runs clear).

4.9.4. Organ Weights

The organs identified in Text Table 15 were recorded for all animals at the scheduled necropsy, and appropriate organ weight ratios were calculated (relative to body and brain weights). Paired organs were weighed together. Organ weights were collected per Testing Facility SOP.

Text Table 15
Organs Weighed at Necropsy

Brain	Kidney
Epididymis	Liver
Gland, adrenal	Ovary
Gland, pituitary	Spleen
Gland, prostate	Testis
Gland, thyroid/parathyroid	Thymus
Heart	Uterus/Cervix

4.9.5. Tissue Collection and Preservation

Representative samples of the tissues identified in Text Table 16 were collected from all animals and preserved in 10% neutral buffered formalin (NBF).

Text Table 16
Tissue Collection and Preservation

Artery, aorta	Large intestine, rectum
Body cavity, nasal	Larynx
Bone marrow smear[a]	Liver
Bone marrow, sternum	Lung
Bone, femur	Lymph node(s) draining administration site(s): Iliac draining lymph node
Bone, sternum	Lymph node, mandibular
Brain	Lymph node, mesenteric
Dorsal root ganglia, cervical	Macroscopic abnormalities[c]
Dorsal root ganglia, thoracic	Muscle, skeletal
Dorsal root ganglia, lumbar	Nerve, optic[b]
Epididymis[b]	Nerve, sciatic
Esophagus	Nerve, tibial
Eye[b]	Ovary
Gland, adrenal	Oviduct
Gland, clitoral	Pancreas
Gland, Harderian	Skin
Gland, lacrimal	Small intestine, duodenum
Gland, mammary	Small intestine, ileum
Gland, parathyroid	Small intestine, jejunum
Gland, pituitary	Spinal cord, cervical
Gland, preputial	Spinal cord, lumbar
Gland, prostate	Spinal cord, thoracic
Gland, salivary, mandibular	Spleen
Gland, salivary, parotid	Stomach
Gland, salivary, sublingual	Testis[b]
Gland, seminal vesicle	Thymus
Gland, thyroid	Tongue
Gland, Zymbal's	Trachea
Gut-associated lymphoid tissue	Ureter
Heart	Urinary bladder
Joint, femorotibial	Uterus/Cervix
Kidney	Vagina
Large intestine, cecum	
Large intestine, colon	

[a] Bone marrow smears were allowed to air dry and were not fixed in formalin.
[b] Eyes and optic nerves were preserved in Davidson's fixative. Testes and epididymides were preserved in modified Davidson's fixative.
[c] Macroscopic abnormalities in the tissues listed were sampled, processed, and evaluated histologically.

4.9.5.1. Brain Tissue Collection

The entire brain was sliced into 3 mm coronal slabs using a brain matrix. Slabs were arranged in sequential order (rostral to caudal) and photographed (caudal surface) (see Appendix 1).

Slabs were designated for collection and processing as follows:

Each brain slab was bisected into left and right hemispheres along the sagittal midline. The left hemislabs were wrapped in parafilm, then wrapped in aluminum foil, then frozen on dry ice. The slabs were stored in pre-cooled 5 mL tubes and stored frozen (-60°C to -90°C) until shipment to the Sponsor.

For fixed brain samples, the designated right hemislabs were submitted rostral side down in standard cassettes to maintain orientation and preserved in 10% NBF.

4.9.6. Biodistribution Frozen Tissue Collection

Care was given to ensure that cross contamination between tissues did not occur. Gloves and disposable collection tools were changed between collection and dissection of each tissue for qPCR biodistribution analysis. The cutting board and non-disposable instruments were wiped down with a 10% bleach solution, rinsed with water, followed by a wipe down of 70% or greater ethanol between each of the specified organs; sterile trays were used when necessary.

Intervals and Number of Animals:	All survivors at necropsy (Day 29).
Tissues Collected:	See Text Table 17.
Sample Collection:	A portion (1 to 2 mm) of each required tissue was collected (2 to 3 samples each), without sacrificing what was needed for histopathology. Brain samples were collected as described in Section 4.9.5.<br><br>All samples (except brain slabs) were placed into pre-labeled polypropylene vials, tightly capped, and frozen in liquid nitrogen. Care was taken to minimize the time between sample collection and freezing. Samples (including brain slabs) were stored on dry ice until final storage (see Appendix 1).
Storage Conditions:	Frozen (-60° to -90°C)
Label Requirements:	Study number, animal number, group number, nominal study day, analysis type (e.g., Biodistribution), tissue type (e.g., ORGANS), and when possible, the date of collection.

Text Table 17
Biodistribution Tissue Collection

Brain<br>Dorsal root ganglia, cervical[a]<br>Dorsal root ganglia, thoracic[a]<br>Dorsal root ganglia, lumbar[a]<br>Gland, adrenal<br>Gland, mammary<br>Heart<br>Kidney<br>Liver	Lung<br>Ovary<br>Pancreas<br>Spinal cord, cervical<br>Spinal cord, lumbar<br>Spinal cord, thoracic<br>Spleen<br>Testis (Left)

[a] One pair per level was collected.

4.9.7. Biodistribution Frozen Tissue Sample Analysis

Analysis Performed By:	The samples were shipped on dry ice with a temperature monitor to UMass Chan Medical School, Worchester, MA, and analyzed for AAV9-scAAV9-CSA. All analytical work was conducted by a Sponsor-contracted laboratory, UMass Chan Medical School, using an analytical method developed and qualified by that laboratory.

4.9.8. Spleen Sample Collection for Isolation of PBMC for ELISpot Assays

One third of the spleen was collected for PBMC sample analysis from the animals designated for necropsy on the last 2 days of the study (30 Oct 2025 and 31 Oct 2025). The spleen samples were placed into a tube with media provided by the Sponsor and stored on wet ice. The remaining spleen was used for histopathology and biodistribution analysis.

The samples collected on 30 Oct 2025 were shipped on ice packs to Paul J. DiMare Center, Worcester, MA for analysis. The samples collected on 31 Oct 2025 were shipped on ice packs to Rita Batista, Holden, MA.

The samples were used to isolate PBMCs to conduct ELISpot assays to evaluate adaptive immunological responses to AAV9 capsid and human CSA protein. All analytical work was conducted by the Sponsor.

4.9.9. Histology

Histology was conducted per Testing Facility SOPs.

4.9.10. Microscopic Evaluations

Protocol designated tissues were evaluated histopathologically by light microscopy by a board-certified veterinary pathologist

5. STATISTICS

All results presented in the tables of the report are calculated using non-rounded values as per the raw data rounding procedure and may not be exactly reproduced from the individual data presented.

Means and standard deviations were calculated for body weights, body weight gains, neurobehavioral evaluations, hematology, coagulation, and clinical chemistry parameters, and organ weights (absolute weights and relative to body and brain weights.

5.1. Constructed Variables

Body Weight Gains	Calculated between each scheduled interval.
Organ Weight Relative to Body Weight	Calculated against the terminal body weight for scheduled intervals.
Organ Weight Relative to Brain Weight	Calculated against the brain weight for scheduled intervals.

5.2. Descriptive Statistical Analyses

Means, standard deviations (or % coefficient of variation or standard error, when deemed appropriate), ratio, percentages, numbers, and/or incidences were reported as appropriate by dataset.

6. COMPUTERIZED SYSTEMS AND ELECTRONIC DATA ARCHIVE LOCATIONS

Critical computerized systems used in the study and their respective electronic data archival locations are listed below or presented in the appropriate phase report. All computerized systems used in the conduct of this study have been validated; when a particular system has not satisfied all requirements, appropriate administrative and procedural controls were implemented to assure the quality and integrity of data.

Text Table 18
Critical Computerized Systems

System Name	System Description	Archive Location
Deviation Information Library	Provides a repository of deviations that allows for documenting, approving, and archiving all to be done electronically	Charles River Massachusetts Datacenter
DocuSign™ eSignature	Collection of 21 CFR Part 11 compliant signature	Charles River Massachusetts Datacenter
eInfotree™	Excel Module for data collection with 21 CFR Part 11 compliance requirements, security, audit trail, and electronic signatures	Charles River Massachusetts Datacenter<br>Testing Facility
ExyLIMS	A comprehensive laboratory information management system used to manage data, including but not limited to instrumentation, test materials, standards, and samples	Testing Facility
Logbook™	Electronic notebook and data collection system for veterinary communications, observations, and treatments	Testing Facility
M-Files®	Document management, publishing, and collection of 21 CFR Part 11 compliant signatures	Charles River Massachusetts Datacenter
Provantis®	Toxicology, clinical pathology, pathology, test material receipt, and formulation data collection, reporting, and statistics system	Charles River Massachusetts Datacenter
SAS®	Statistical analysis	Charles River Massachusetts Datacenter

System Name	System Description	Archive Location
Siemens Monitoring System	Environmental monitoring, alarming, and reporting applications	Testing Facility

7. RETENTION AND DISPOSITION OF RECORDS, SAMPLES, AND SPECIMENS

All study-specific physical raw data, documentation, and retained samples and specimens from this study are archived at the Testing Facility by no later than the date of Final Report issue.

Electronic data and documentation generated by the Testing Facility are archived. Data generated by critical computerized systems were archived as listed in the critical computerized systems list. All electronic data archived in a cloud environment are under control of the Charles River Laboratories Archivist.

All study-specific items will be retained for at least 1 year after issue of the Draft Report.

Archival location from phases is detailed in the applicable phase reports.

8. RESULTS

8.1. Surgical Procedures

Overall, intrathecal administration of scAAV9-CSA in rats on Days 1 was well tolerated.

8.2. Mortality

(Appendix 3)

All animals survived to the scheduled necropsy.

8.3. Clinical Observations

(Table 1 and Appendix 4)

There were no scAAV9-CSA -related clinical observations evident throughout the study.

8.4. Veterinary Observations

(Appendix 5)

Over the course of the study, veterinary treatments were administered and are included in Appendix 5.

8.5. Body Weights and Body Weight Gains

(Body weight - Figure 1, Table 2, and Appendix 6)

(Body weight gains - Table 3 and Appendix 7)

The administration of the scAAV9-CSA did not affect body weight values.

8.6. Ophthalmology Examinations

(Appendix 8)

There were no scAAV9-CSA-related effects on ophthalmology examinations.

8.7. Neurobehavioral Observations

(Tables 4 through 9 and Appendices 9 through 14)

There were no scAAV9-CSA-related effects on any of the neurobehavioral parameters assessed.

8.8. Hematology

(Appendix 15)

There were no definitive scAAV9-CSA-related alterations in hematology parameters at any dose level at any time point.

On Day 29 ± 1, there were three males that had sporadic changes, including moderately higher neutrophils (Animal No. 4004), mildly higher lymphocytes (Animal No. 4005), or mildly higher eosinophils (Animal No. 4003). Given the sporadic nature of these changes, they were considered to be unlikely scAAV9-CSA related.

All other fluctuations among individual and mean hematology values were sporadic, consistent with biologic and procedure-related variation, and/or negligible in magnitude. Therefore, these fluctuations were considered to be unrelated to scAAV9-CSA administration.

8.9. Coagulation

(Appendix 15)

There were no definitive scAAV9-CSA-related alterations in coagulation parameters at any dose level at any time point.

On Day 29 ± 1, both sexes at 1.49×10^{12} vg had minimally prolonged activated partial thromboplastin time (APTT; up to 1.13x). Given the minimal magnitude of change and general overlap with control group values, alterations in APTT were considered to be of uncertain relationship to scAAV9-CSA administration.

All other fluctuations among individual and mean coagulation values were sporadic, consistent with biologic and procedure-related variation, and/or negligible in magnitude. Therefore, these fluctuations were considered to be unrelated to scAAV9-CSA administration.

8.10. Clinical Chemistry

(Appendix 15)

There were no definitive scAAV9-CSA-related alterations in clinical chemistry parameters at any dose level at any time point.

On Day 29 ±1, one male (Animal No. 4003), had mildly to moderately higher aspartate aminotransferase (AST), alanine aminotransferase (ALT), and sorbitol dehydrogenase (SDH) activities. On Day 91 ±2, in males at 1.49×10^{12} vg and females at $\geq 1.49 \times 10^{11}$ vg, there were 1 to 3 animals per group that had minimal to mild increases in AST, ALT, and SDH activities. In Animal No. 4009, these changes may have correlated with microscopic findings in the liver (Pathology Report). Overall, the alterations in AST, ALT, and SDH were of uncertain relationship to scAAV9-CSA administration given the sporadic nature of the change and due to the presence of a similar increase in one control group male on Day 91 ±2 (Animal No. 1010).

On Day 91 ±2, in males at ≥ 4.47 x 10^{11} vg and female at 1.49 x 10^{12} vg, there were minimal increases in phosphorus concentration. In absence of a comparable change on Day 29 ±1, these changes were of uncertain relationship to scAAV9-CSA administration.

All other fluctuations among individual and mean clinical chemistry values were sporadic, consistent with biologic and procedure-related variation, and/or negligible in magnitude. Therefore, these fluctuations were considered to be unrelated to scAAV9-CSA administration.

8.11. Biodistribution Evaluation

(Appendix 16)

To be included at a later date.

8.12. PBMC Analysis

(Appendix 17)

To be included at a later date.

8.13. Macroscopic Pathology

(Appendix 18)

8.13.1. Terminal Euthanasia Animals (Day 29 (±1) and Day 91 (±2))

There were no scAAV9-CSA-related macroscopic findings.

8.14. Organ Weights

(Appendix 18)

8.14.1. Terminal Euthanasia Animals (Day 29 (±1) and Day 91 (±2))

There were no scAAV9-CSA-related organ weight changes.

All differences in organ weight parameters were attributed to normal biologic variation. These differences had no patterns, trends, or correlating data to suggest these differences were scAAV9-CSA related.

8.15. Microscopic Evaluations

(Appendix 18)

8.15.1. Terminal Euthanasia Animals (Day 29 (±1))

scAAV9-CSA-related microscopic findings at 1.49 x10^{12} vg occurred in the lumbar dorsal root ganglia, sciatic nerve, and spinal cord, and are summarized in Text Table 19.

scAAV9-CSA-related microscopic findings were solely those associated with AAV vector administration (Bennet et al., 2023; Bolon et al., 2024). The lumbar dorsal root ganglia findings were characterized by minimal neuronal degeneration/necrosis and mononuclear infiltration as well as minimal to mild nerve fiber degeneration. The neuronal degeneration/necrosis was identified by eosinophilic cytoplasm often surrounded by mononuclear infiltrates. The nerve fiber degeneration involved axonal swelling, myelin vacuolation, and digestion chambers.

The sciatic nerve and dorsal roots of the lumbar spinal cord in scAAV9-CSA-treated animals also contained minimal to mild nerve fiber degeneration consistent with the lumbar dorsal root ganglia findings. One sciatic nerve also contained minimal mononuclear infiltration. Minimal nerve fiber degeneration of the dorsal funiculus of the lumbar spinal cord was present in two animals.

Text Table 19
Summary of scAAV9-CSA-related Microscopic Findings – Scheduled/Terminal Euthanasia (Day 29 (±1))

	**Males**		**Females**	
**Group**	**1**	**4**	**1**	**4**
**Dose (vg)**	**0**	**1.49×10^{12}**	**0**	**1.49×10^{12}**
**No. Animals Examined**	**5**	**5**	**5**	**5**
**Ganglion, dorsal root, lumbar (No. Examined)**	5	5	5	5
Infiltration, mononuclear cell	(1)	(2)	(1)	(5)
Minimal	1	1	1	4
Mild	0	1	0	1
Degeneration; nerve fiber	(0)	(2)	(0)	(2)
Minimal	0	1	0	0
Mild	0	1	0	2
Degeneration/necrosis; neuronal	(0)	(1)	(0)	(2)
Minimal	0	1	0	2
**Nerve, sciatic (No. Examined)**	5	5	5	5
Degeneration; nerve fiber	(0)	(2)	(0)	(4)
Minimal	0	2	0	3
Mild	0	0	0	1
Infiltration, mononuclear cell	(0)	(0)	(0)	(1)
Minimal	0	0	0	1
**Spinal cord (No. Examined)**	5	5	5	5
Degeneration; nerve fiber	(0)	(2)	(0)	(3)
Minimal	0	1	0	3
Mild	0	1	0	0

All other microscopic observations in scAAV9-CSA-treated animals were considered incidental and not test article related as these are known background observations for rats, were of low incidence and/or severity, they lacked a dose response relationship, or they were not similar amongst the sexes.

8.15.2. Terminal Euthanasia Animals (Day 91 (±2))

Notable microscopic findings considered related to the administration of scAAV9-CSA at the Day 91 (±2) timepoint were present in the dorsal root ganglia (lumbar, thoracic, and cervical), sciatic nerve, and spinal cord. All microscopic findings were consistent with expected findings associated with the use of AAV vectors (Bennet et al., 2023; Bolon et al., 2024).

The lumbar dorsal root ganglia findings at $\geq 4.47 \times 10^{11}$ vg considered related to the administration of scAAV9-CSA included minimal neuronal degeneration/necrosis and minimal to mild glial cell aggregates. The neuronal degeneration/necrosis was identified by eosinophilic cytoplasm surrounded by mononuclear infiltrates. The glial cell aggregates are typical of a reparative process and indicate previous neuronal injury. Minimal to moderate nerve fiber degeneration and minimal to mild mononuclear infiltration were present at increased incidence and severity in $\geq 4.47 \times 10^{11}$ vg males and in $\geq 1.49 \times 10^{11}$ vg females. The nerve fiber

degeneration involved axonal swelling, myelin vacuolation, and occasional digestion chambers. The finding of nerve fiber degeneration had a similar appearance at all the other sites (thoracic and cervical dorsal root ganglia, sciatic nerve, and spinal cord (see Text Table 20). The location of the nerve fiber degeneration was consistent with that expected with dorsal root ganglion injury using AAV vectors.

Text Table 20
Summary of scAAV9-CSA-related Microscopic Findings – Scheduled/Terminal Euthanasia (Day 91 (±2))

	**Males**				**Females**			
**Group**	**1**	**2**	**3**	**4**	**1**	**2**	**3**	**4**
**Dose (vg)**	**0**	**1.49 x 10^{11}**	**4.47 x 10^{11}**	**1.49 x 10^{12}**	**0**	**1.49 x 10^{11}**	**4.47 x 10^{11}**	**1.49 x 10^{12}**
**No. Animals Examined**	**5**	**10**	**10**	**10**	**5**	**10**	**10**	**10**
**Ganglion, dorsal root, lumbar (No. Examined)**	5	10	10	10	5	10	10	10
Infiltration, mononuclear cell	(2)	(2)	(6)	(7)	(0)	(5)	(4)	(8)
Minimal	2	2	5	5	0	5	3	5
Mild	0	0	1	2	0	0	1	3
Degeneration; nerve fiber	(1)	(1)	(6)	(10)	(0)	(3)	(4)	(10)
Minimal	1	1	5	5	0	3	2	6
Mild	0	0	1	4	0	0	2	2
Moderate	0	0	0	1	0	0	0	2
Degeneration/necrosis; neuronal	(0)	(0)	(1)	(2)	(0)	(0)	(1)	(2)
Minimal	0	0	1	2	0	0	1	2
Aggregate; glial cell	(0)	(0)	(3)	(8)	(0)	(0)	(2)	(8)
Minimal	0	0	3	7	0	0	1	6
Mild	0	0	0	1	0	0	1	2
**Ganglion, dorsal root, cervical (No. Examined)**	5	10	10	10	5	10	10	10
Infiltration, mononuclear cell	(0)	(0)	(0)	(1)	(0)	(0)	(1)	(0)
Minimal	0	0	0	1	0	0	1	0
Degeneration; nerve fiber	(0)	(0)	(0)	(0)	(0)	(0)	(1)	(0)
Minimal	0	0	0	0	0	0	1	0
**Ganglion, dorsal root, thoracic (No. Examined)**	5	10	10	10	5	10	10	10
Degeneration; nerve fiber	(0)	(1)	(0)	(3)	(0)	(0)	(0)	(1)
Minimal	0	1	0	3	0	0	0	0
Mild	0	0	0	0	0	0	0	1
**Nerve, sciatic (No. Examined)**	5	10	10	10	5	10	10	10
Degeneration; nerve fiber	(0)	(4)	(5)	(8)	(0)	(2)	(5)	(9)
Minimal	0	4	5	8	0	2	5	9
**Spinal cord (No. Examined)**	5	10	10	10	5	10	10	10
Degeneration; nerve fiber	(1)	(2)	(3)	(7)	(0)	(2)	(6)	(9)
Minimal	0	2	3	5	0	2	4	2
Mild	1	0	0	2	0	0	2	7

In one 1.49 x 10^{12} vg male (Animal No. 4009), there was an unusual finding in the liver with hepatocellular vacuolation and hepatocytes containing eosinophilic globules. As this occurred in just one animal, it is not clearly associated with scAAV9-CSA administration and therefore was considered a background change unrelated to treatment.

9. CONCLUSION

Overall, intrathecal administration of scAAV9-CSA to male and female rats on Day 1 at, 1.49 $x10^{11}$, 4.47 $x10^{11}$, and 1.49 $x10^{12}$ vg was well tolerated.

Administration was not associated with any mortality nor were there any behavioral effects.

There were no scAAV9-CSA-related alterations in clinical pathology parameters at any dose level at any time point.

There were no scAAV9-CSA-related macroscopic findings nor changes in organ weights.

scAAV9-CSA-related microscopic findings at Day 29 (±1) occurred in the lumbar dorsal root ganglia, sciatic nerve, and spinal cord at 1.49 x 10^{12} vg. scAAV9-CSA-related microscopic findings were solely those associated with AAV vector administration (Bennet et al., 2023; Bolon et al., 2024).

The lumbar dorsal root ganglia findings were characterized by minimal neuronal degeneration/necrosis and mononuclear infiltration as well as minimal to mild nerve fiber degeneration.

The sciatic nerve and dorsal roots of the lumbar spinal cord in scAAV9-CSA-treated animals also contained minimal to mild nerve fiber degeneration consistent with the lumbar dorsal root ganglia findings. One sciatic nerve also contained minimal mononuclear infiltration. Minimal nerve fiber degeneration of the dorsal funiculus of the lumbar spinal cord was present in two animals.

scAAV9-CSA-related microscopic findings at Day 91 (±2) were present in the dorsal root ganglia (lumbar, thoracic, and cervical), sciatic nerve, and spinal cord. All microscopic findings were consistent with expected findings associated with the use of AAV vectors (Bennet et al., 2023; Bolon et al., 2024).

Lumbar dorsal root ganglia findings considered related to the administration of scAAV9-CSA included minimal neuronal degeneration/necrosis and minimal to mild glial cell aggregates at ≥ 4.47 x 10^{11} vg. Minimal to moderate nerve fiber degeneration and minimal to mild mononuclear cell infiltration was present at increased incidence and severity in males at ≥ 4.47 x 10^{11} and females at ≥ 1.49 x 10^{11} vg. Neuronal degeneration/necrosis was not identified in animals administered the 1.49 x 10^{11} dose.

Thoracic dorsal root ganglia findings considered related to the administration of scAAV9-CSA included minimal to mild nerve fiber degeneration at 1.49 x 10^{12} vg in males and females and in one male at 1.49 x 10^{11} vg. Cervical dorsal root ganglia had one 4.47 x 10^{11} vg female with minimal nerve fiber degeneration considered related to scAAV9-CSA administration.

Sciatic nerve findings considered related to the administration of scAAV9-CSA included minimal nerve fiber degeneration at ≥ 1.49 x 10^{11} vg in males and females. Spinal cord findings considered related to the administration of scAAV9-CSA were present mostly in the lumbar and thoracic segments in the dorsal funiculus and dorsal nerve roots. These findings included minimal to mild nerve fiber degeneration at ≥ 4.47 x 10^{11} vg males and ≥ 1.49 x 10^{11} vg females.

Neuronal and nerve fiber degeneration is a common class effect when using AAV vectors and at low severity its functional relevance is determined on an individual case basis (Bolon et al., 2024).

Biodistribution and PBMC analyses will be included at a later date.

Based on the totality of the observations and evaluations, the NOAEL of scAAV9-CSA is considered 1.49 x 10^{11} vg, the lowest dose administered.

10. REFERENCES

Bennet BM et al. Scientific and Regulatory Policy Committee Points to Consider: Sampling, Processing, Evaluation, Interpretation, and Reporting of Test Article-Related Ganglion Pathology for Nonclinical Toxicity Studies. Toxicologic Pathology 2023, Vol. 51(4) 176-204.

Bolon B et al. Neuropathological Findings in Nonclinical Species Following Administration of Adeno-Associated Virus (AAV)-Based Gene Therapy Vectors. Toxicologic Pathology 2024, Vol. 52(8) 498-505.

National Research Council. *Guide for the Care and Use of Laboratory Animals*. 8th ed. Washington, DC: National Academies Press. Current edition.

Office of Laboratory Animal Welfare. *Public Health Services Policy on Humane Care and Use of Laboratory Animals*. Bethesda, MD: National Institutes of Health. Current edition.

Figure 1

Summary of Group Mean Body Weights

32025165

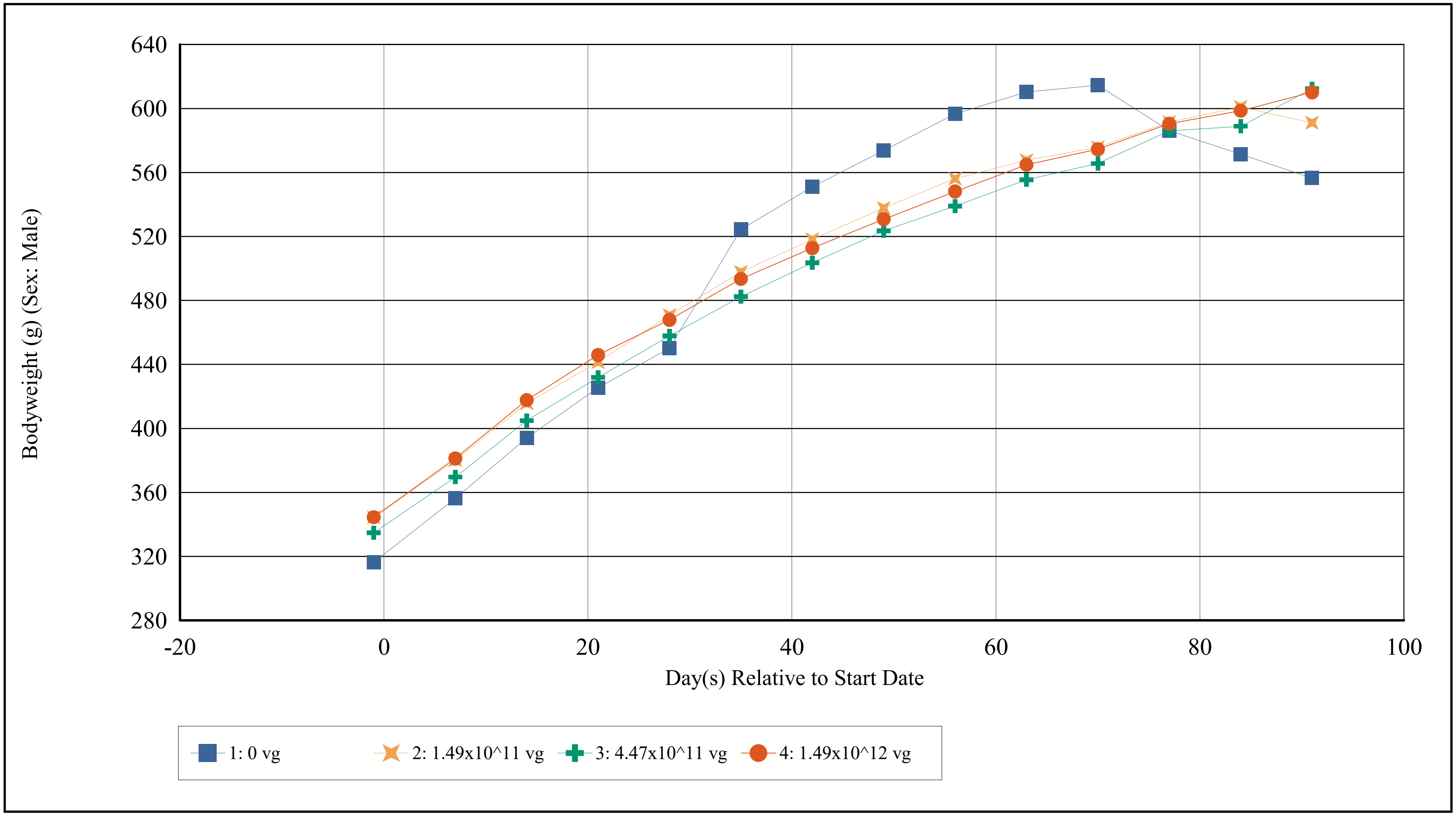

Day 91 represents data collected on Day 90 and 91.

Figure 1

Summary of Group Mean Body Weights

32025165

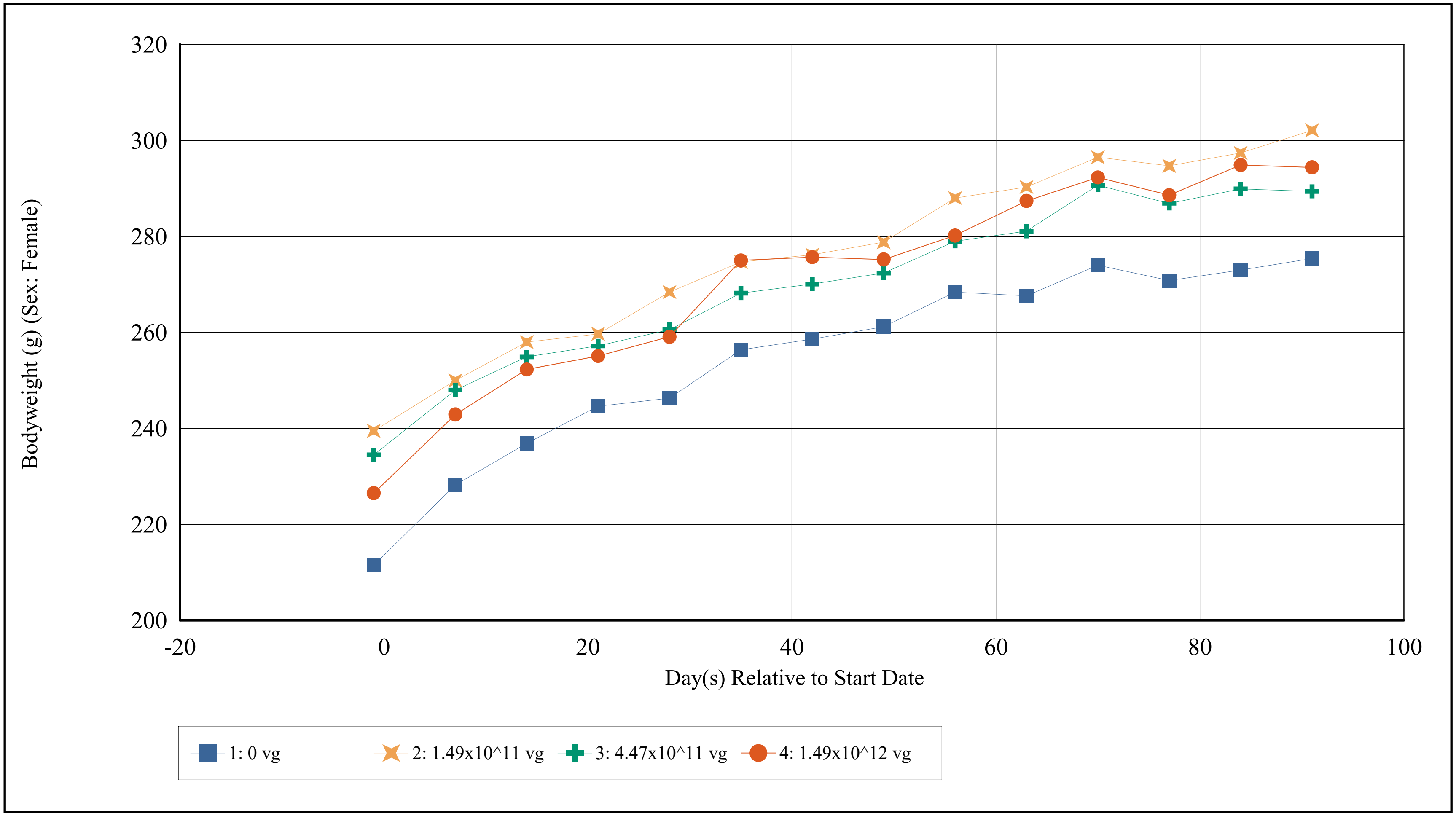

Day 91 represents data collected on Day 90 and 91.

DATA EXPLANATION PAGE

All Day(s) referenced throughout the outputs generated are based on Day 1 (day of dose administration).

Note: All abbreviations listed on these pages may not be applicable to this report.

Abbreviation	Description
% Diff	% Difference from Group 1
tCtrl	Times control (fold change)
1F, 2F, 3F, 4F	Group 1 Female, Group 2 Female, Group 3 Female, Group 4 Female
1M, 2M, 3M, 4M	Group 1 Male, Group 2 Male, Group 3 Male, Group 4 Male
N	Number of values included in analysis
M, F	Male, Female
TK	Toxicokinetic
<, >	Out of range
--	Not scheduled to be performed/dead
- or .	Not applicable

Abbreviation	Description	Abbreviation	Description
E	Exclude	Temp	Temperature
Int.	Interval	TERR	Technical error
NA	Not applicable	UPTD	Unable to perform due to technical difficulty
NC	Not calculable	UTD	Unable to determine
No.	Animal number	Wt	Weight
NSCH	Not scheduled to be performed		

TIMESLOTS

4H – 4 hours postdose
24H – 24 hours postdose

CAGESIDE AND CLINICAL OBSERVATIONS

Only animals with findings are presented in this table and certain days may not be represented if all animals had no abnormalities detected on that day.

Abbreviation	Description	Abbreviation	Description
-	Severity not recorded	N	Severity not applicable
!	Comment present	SIRT	Signs of ill response to treatment
X	Present	PT	Permanent
Unsc/Unsch	Unscheduled	NAD	No abnormalities detected

BODY WEIGHTS

Abbreviation	Description	Abbreviation	Description
AVS	Suspected aberrant value	TERR	Technical Error
E	Excluded	UPTD	Unable to perform due to technical difficulty
OA	Omitted Activity	X	Excluded from mean

SURVIVAL/MORTALITY

Abbreviation	Description	Abbreviation	Description
ACCD	Accidental death	PostRx	Observation postdosing
AD	Accidental death	PreRx	Observation predosing
AM SIRT	Mortality/moribundity check in the morning	REC	Recovery euthanasia
CSO	Cage side observation	REL	Released
DE	Detailed examination	TE	Terminal euthanasia
FD	Found dead	TERM	Terminal euthanasia
INTM	Interim euthanasia	UE	Unscheduled euthanasia
N+ve	Number positive	UNSC	Unscheduled euthanasia
PM SIRT	Mortality/moribundity check in the afternoon		

NEUROBEHAVIORAL ASSESSMENTS

Abbreviation	Description	Abbreviation	Description
°C	degree Celsius	N+ve	Number of animals with observation
Grp	Grip	RC	Result comment
Ha	Handling	refl	Reflex
HC	Home Cage	resp	Response
OF	Open Field	sec	seconds
Mn	Mean		

ACTIVITY OBSERVATIONS

Arousal/Alertness - The level of unprovoked activity/alertness in the open field.

-2	Very low (stuporous, comatose, little to no movement)
-1	Low (sluggish, some exploratory movement)
0	Normal (alert with exploratory movement)
1	High (tense, some bouts of startle, darting, or freezing without stimuli)
2	Very high (excited, multiple bouts of startle, running, darting, freezing, or body movements without stimuli)

Posture/Body Carriage - The position of body parts relative to each other. Certain postures indicate abnormal physiological states. This evaluation is performed prior to removing the animal from the home cage.

-2	Prostrate (limbs may be in the air) or flattened to cage floor (limbs may be spread out)
-1	Hunched or low carriage
0	Normal; asleep, sitting or standing normally

Stereotypy - Any behavior that is excessive or repetitive or exceeds that observed in normal animals. Examples of stereotypy include, but are not limited to, tight circling, repetitive grooming of an extended duration, repetitive sniffing and/or licking in one area, head weaving back and forth, and self-mutilation. Stereotypic behaviors are an indicator of abnormal central nervous system function. For scores other than 0, the specific behavior observed was recorded as a comment.

0	Not present; normal
1	Continuous licking, biting/gnawing of the wall or floor, continuous or excessive grooming
2	Repetitive mouth movements, and/or head weaving/bobbing
3	Circling, retropulsion, forepaw treading
4	Self-injurious behavior; licking, biting, or gnawing of the body

Rearing - A rear is defined as the simultaneous raising of both forefeet off the surface of the open-field test box. A rear may be a brief, low raising of both forelimbs. A rear in which the animal supports itself on the side of the box with 1 or both forelimbs is also counted. Rearing is a normal posture in rodents and is a sign of both intact motor functioning (the ability to support weight on the haunches) and normal arousal.

The number of times an animal rears onto its hindlimbs with both forelimbs raised off the surface of the open field box is recorded over the 2-minute observation period.

Appearance - An evaluation of the general appearance of the animal assessing cleanliness and grooming.

0	Clean and groomed
1	Slightly unkempt (slightly discolored / crusty fur)
2	Extremely unkempt (markedly discolored, matted, and/or crusty fur)

EXCITABILITY

Vocalization - Vocalizations usually occur in response to negative stimuli. The presence of multiple spontaneous vocalizations (not in reaction to being handled; also includes vocalizations in the open field) suggests an abnormal internal state of the animal.

0	Spontaneous vocalizations occur once or not at all
1	Spontaneous vocalizations occur more than once

Startle Response - An assessment of the reaction of the animal to an auditory stimulus of a loud, quick click. The clicker is positioned approximately 5 cm above the back of the animal and a click is sounded suddenly.

-1	No reaction
0	Normal reaction (some evidence that the noise was heard, e.g., normal freezing, flinching, or jumping behaviors)
1	Exaggerated reaction (e.g., excessive jumping, attacking, or vocalization)

Ease of Removal - An evaluation of the reaction of the animal to generalized stimuli. This assessment occurs while the animal is being removed from the home cage.

-1	Very easy (animal is sluggish or limp)
0	Easy (sits quietly, allows observer to pick it up with minimal resistance)
1	Moderate (e. g. rearing, following observer's hand, vocalization)
2	Difficult (e. g. running around cage, is hard to grab, vocalization, tail and throat rattles, attacking observer's hand)

Handling Reactivity - An evaluation of the reaction of the animal to generalized stimuli. This assessment occurs while the animal is held and examined upon removal from the home cage.

-1	Very low (animal is totally limp or otherwise unresponsive)
0	Low (minimal resistance, is easy to handle)
1	Moderate (slight resistance)
2	High (e.g., freezing, tense or rigid in hand, squirming, twisting, or attempting to bite)

Convulsions - Involuntary clonic movements characterized by alternating contraction and relaxation of musculature; or involuntary tonic movements characterized by continuous muscular contractions. Clonic movements may occur with or without tonic movements. For scores other than 0, the specific behavior observed was recorded as a comment.

0	None
1	Clonic movement of limbs, ears, head, or skin, repetitive movements of mouth and jaw; contraction of extensors such that limbs are rigid, extended
2	Clonic tremors or clonus of the limbs or whole body; opisthotonus (head and body rigidly arched backwards) or emprosthotonus (head and body rigidly extended forward)
3	Severe clonus of the limbs or whole body; myoclonic jerks; explosive jumps into the air with all feet leaving surface
4	Clonic convulsions affecting the whole body; clonic-tonic convulsions, possibly resulting in dyspnea, or death

AUTONOMIC OBSERVATIONS

Exophthalmus - An abnormal protrusion of the eyeball.

0	Not present
1	Present

Lacrimation - The excessive secretion of tears and a sign of abnormal autonomic functioning.

0	None (no excess lacrimation)
1	Slight (excess moisture at the margin of the eyelid)
2	Severe (dampness extends beyond the margin of the eyelid)

Erected Fur - Characterized by hair that is standing approximately vertical to the surface of the body of the animal. It is produced by the contractions of the small muscles around the hair follicles and is a sign of heightened arousal or abnormal autonomic functioning. During the evaluation, erected fur (piloerection) is distinguished from unkemptness by the inability of the technician to smooth the hair by stroking it from a rostral to caudal direction.

0	Not present
1	Present (coat does not lie down after stroking)

Pupil Response - The normal pupil constricts to a bright light stimulus; failure to constrict indicates abnormal autonomic or central nervous system functioning. The animal's eyes are covered for a minimum of 5 seconds, then a beam from a penlight is brought in from the side of the head to near the surface of the eye and the effect on pupil size is noted.

0	Normal constriction of pupils
1	Partial, slow, or lack of constriction of pupils
2	Pupils are fully constricted prior to performing the test (i.e., "pinpoint pupils")

Salivation - The excessive production of saliva to the extent that it is visible around the mouth of the animal. Excessive salivation is a sign of abnormal autonomic functioning.

0	None (no excess salivation)
1	Slight (margin of mouth wet)
2	Severe (wet zone extends beyond the margin of the mouth)

Defecation - The appearance of the feces is noted.

-1	Absent
0	Normal
1	Abnormal

Palpebral Closure/Ptosis - The partial or complete closure of the eyelids. It is a sign of an abnormal physiological state or, when the animal is not compromised, a sign of normal restfulness or sleep. If palpebral closure is seen as representing an abnormal physiologic state it is also called ptosis. During the evaluation, palpebral closure is evaluated both in the home cage, prior to handling, where its presence could be either normal or abnormal, and during handling, where, due to the aroused state of the animal, it is considered a reliable sign of abnormality.

0	Eyelids wide open
1	Eyelids drooping approximately half-way
2	Eyelids completely shut

NEUROMUSCULAR OBSERVATIONS

Gait/Mobility – Gait is the manner in which the animal walks; it is an indication of the integrity of central nervous system motor function. Mobility is the ability to locomote, despite gait abnormalities. The animal is evaluated during the 2-minute open-field observation period.

0	Normal gait/mobility
1	Slightly impaired (any or all of the following can be evident): mild ataxia, rocks or lurches during ambulation; hunched or crouched body position; walks on tiptoe
2	Moderately impaired (any or all of the following can be evident): marked ataxia; feet point outwards from the body; hind limbs show exaggerated or overcompensated movements, drag, or are splayed
3	Severely impaired (any or all of the following can be evident): Forelimbs drag or are unable to support weight; body drags or is flattened against surface

Grip Strength – Measured using a digital strain gauge with metal grasping bars/grid. An evaluation of the strength of the motor reflex elicited when an object is pulled against the plantar surfaces of the paws. Three measurements of both forelimb and hindlimb grip strengths are performed and averaged to produce a mean grip strength value for each animal.

Hindlimb Splay – An evaluation of muscle tonus. The bottom of the outside toe of each hind foot is dotted with ink and the animal is dropped approximately 32 cm. The distance between the impressions made by the inked digits is measured. Two measurements of hind foot splay are obtained and averaged to produce the hind foot splay value for the animal.

Air Righting Reflex - The reflexive ability of an animal to turn mid-air from a supine position to one allowing it to land upright. The animal is dropped from a height of approximately 30 cm. If the animal is paralyzed or severely impaired, this test should not be conducted, and the animal is given a score of “2”.

0	Normal (animal lands on four feet)
1	Abnormal
2	Animal severely impaired (not tested)

Tremor - Uncontrollable shaking of all or part of the body.

0	None
1	Slight (barely perceptible)
2	Moderate (apparent, but still able to perform other activities)
3	Severe (impairs general activity)

Body Tone - The animal is grasped with one hand while the other hand gently palpates the lower thorax and pelvic area to evaluate body tone. Body tone can also be evaluated by raising the animal off the surface in a vertical position such that the observer can evaluate the presence/strength of the extensor thrust reflex via digital palpation by gently pressing into the middle of the plantar surface (i.e., footpads) of each hindlimb. Either method may be used.

-1	Decreased (flaccidity)
0	Normal
1	Increased (resistance to palpation; rigidity)

SENSORIMOTOR OBSERVATIONS

Touch Response/Tactile Reflex - An assessment of the reaction of the animal to a tactile stimulus of a blunt object gently touching the rump.

-1	No reaction
0	Normal reaction (e. g. turns towards or away from stimulus)
1	Exaggerated reaction (freezes or flinches, jumps, bites, or attacks)

Thermal Response - A measurement of the latency of the animal to respond to a nociceptive heat stimulus. The animal is placed on a hot plate apparatus set to a temperature of 52°C (±1°C). The time (in seconds) between placement of the animal on the heated surface and the occurrence of a responses/escape behavior is recorded: licking of the fore or hind paws, an increase in ambulatory activity, rapidly repeated elevations of either hind paw, and/or jumping; clinging to the top of the enclosure. The animal is quickly removed from the hot plate when a response is emitted, or the maximum allowable duration has been reached (i.e., 60 seconds)

PHYSIOLOGICAL OBSERVATIONS

Respiration - The quality of the respiration of the animal is assessed as an indicator of autonomic involvement and of the general physiological state of the animal.

-1	Slowed
0	Normal
1	Rapid
2	Abnormal

Body Temperature

The body temperature of the animal was recorded in degrees Celsius (°C) using a rectal thermometer.

Table 1

Summary of Clinical Observations

32025165

Observation Type: Toxicology Observations<br>From Day -1 (Start Date) to 91 (Start Date)	Male<br>0 vg<br>Group 1	 1.49x10^11 vg<br>Group 2	 4.47x10^11 vg<br>Group 3	 1.49x10^12 vg<br>Group 4	Female<br>0 vg<br>Group 1	 1.49x10^11 vg<br>Group 2	 4.47x10^11 vg<br>Group 3	 1.49x10^12 vg<br>Group 4
**Normal**								
Number of Animals Affected	10	10	10	14	10	10	9	15
Number of Times Recorded	66	112	92	109	85	86	89	114
% of Affected Animals	100	100	100	93	100	100	90	100
First to Last seen	-1 - 84	-1 - 91	-1 - 91	-1 - 91	-1 - 91	-1 - 91	-1 - 91	-1 - 91
**Vocalization, Increased**								
Number of Animals Affected	0	0	1	2	0	1	0	0
Number of Times Recorded	0	0	5	6	0	3	0	0
% of Affected Animals	0	0	10	13	0	10	0	0
First to Last seen	-	-	63 - 91	63 - 91	-	49 - 63	-	-
**Dehydrated Suspected, Severity Not Recorded**								
Number of Animals Affected	2	0	0	0	0	0	0	0
Number of Times Recorded	4	0	0	0	0	0	0	0
% of Affected Animals	20	0	0	0	0	0	0	0
First to Last seen	77 - 84	-	-	-	-	-	-	-
**Fur, Staining, Dorsal Cervical, Brown**								
Number of Animals Affected	0	0	0	2	0	0	0	0
Number of Times Recorded	0	0	0	6	0	0	0	0
% of Affected Animals	0	0	0	13	0	0	0	0
First to Last seen	-	-	-	28 - 42	-	-	-	-
**Fur, Staining, Eyelid, Right, Brown**								
Number of Animals Affected	0	0	1	0	0	0	0	0
Number of Times Recorded	0	0	3	0	0	0	0	0
% of Affected Animals	0	0	10	0	0	0	0	0
First to Last seen	-	-	28 - 42	-	-	-	-	-
**Fur, Thin Cover, Abdominal**								
Number of Animals Affected	0	0	0	1	0	0	0	0

Table 1

Summary of Clinical Observations

32025165

Observation Type: Toxicology Observations	Male				Female			
From Day -1 (Start Date) to 91 (Start Date)	0 vg Group 1	1.49x10^11 vg Group 2	4.47x10^11 vg Group 3	1.49x10^12 vg Group 4	0 vg Group 1	1.49x10^11 vg Group 2	4.47x10^11 vg Group 3	1.49x10^12 vg Group 4
**Fur, Thin Cover, Abdominal (Continued...)**								
Number of Times Recorded	0	0	0	5	0	0	0	0
% of Affected Animals	0	0	0	7	0	0	0	0
First to Last seen	-	-	-	63 - 91	-	-	-	-
**Fur, Thin Cover, Cranium**								
Number of Animals Affected	0	0	0	0	0	0	1	0
Number of Times Recorded	0	0	0	0	0	0	12	0
% of Affected Animals	0	0	0	0	0	0	10	0
First to Last seen	-	-	-	-	-	-	14 - 91	-
**Fur, Thin Cover, Dorsal Cervical**								
Number of Animals Affected	0	0	0	0	0	1	0	1
Number of Times Recorded	0	0	0	0	0	10	0	6
% of Affected Animals	0	0	0	0	0	10	0	7
First to Last seen	-	-	-	-	-	28 - 91	-	56 - 91
**Fur, Thin Cover, Face**								
Number of Animals Affected	0	3	2	3	0	1	1	4
Number of Times Recorded	0	15	11	19	0	12	1	20
% of Affected Animals	0	30	20	20	0	10	10	27
First to Last seen	-	35 - 91	35 - 91	14 - 91	-	14 - 91	49 - 49	21 - 91
**Fur, Thin Cover, Forelimb, Left**								
Number of Animals Affected	0	0	0	0	0	0	1	0
Number of Times Recorded	0	0	0	0	0	0	1	0
% of Affected Animals	0	0	0	0	0	0	10	0
First to Last seen	-	-	-	-	-	-	91 - 91	-
**Fur, Thin Cover, Forelimb, Right**								
Number of Animals Affected	1	0	0	0	0	0	1	0
Number of Times Recorded	6	0	0	0	0	0	1	0

Table 1

Summary of Clinical Observations

32025165

Observation Type: Toxicology Observations	Male				Female			
From Day -1 (Start Date) to 91 (Start Date)	0 vg Group 1	1.49x10^11 vg Group 2	4.47x10^11 vg Group 3	1.49x10^12 vg Group 4	0 vg Group 1	1.49x10^11 vg Group 2	4.47x10^11 vg Group 3	1.49x10^12 vg Group 4
**Fur, Thin Cover, Forelimb, Right (Continued...)**								
% of Affected Animals	10	0	0	0	0	0	10	0
First to Last seen	56 - 90	-	-	-	-	-	91 - 91	-
**Fur, Thin Cover, Generalized**								
Number of Animals Affected	0	0	0	0	0	0	1	0
Number of Times Recorded	0	0	0	0	0	0	8	0
% of Affected Animals	0	0	0	0	0	0	10	0
First to Last seen	-	-	-	-	-	-	42 - 91	-
**Fur, Thin Cover, Inguinal, Left**								
Number of Animals Affected	0	0	0	0	0	0	1	0
Number of Times Recorded	0	0	0	0	0	0	11	0
% of Affected Animals	0	0	0	0	0	0	10	0
First to Last seen	-	-	-	-	-	-	21 - 91	-
**Fur, Thin Cover, Lumbar**								
Number of Animals Affected	0	0	0	0	0	0	1	1
Number of Times Recorded	0	0	0	0	0	0	1	1
% of Affected Animals	0	0	0	0	0	0	10	7
First to Last seen	-	-	-	-	-	-	91 - 91	91 - 91
**Skin, Discolored, Tail, Red**								
Number of Animals Affected	0	0	0	0	0	1	0	0
Number of Times Recorded	0	0	0	0	0	1	0	0
% of Affected Animals	0	0	0	0	0	10	0	0
First to Last seen	-	-	-	-	-	14 - 14	-	-
**Skin, Scab, Abdominal**								
Number of Animals Affected	0	0	0	1	0	0	0	0
Number of Times Recorded	0	0	0	2	0	0	0	0
% of Affected Animals	0	0	0	7	0	0	0	0

Table 1

Summary of Clinical Observations

32025165

Observation Type: Toxicology Observations<br>From Day -1 (Start Date) to 91 (Start Date)	Male<br>0 vg<br>Group 1	 1.49x10^11 vg<br>Group 2	 4.47x10^11 vg<br>Group 3	 1.49x10^12 vg<br>Group 4	Female<br>0 vg<br>Group 1	 1.49x10^11 vg<br>Group 2	 4.47x10^11 vg<br>Group 3	 1.49x10^12 vg<br>Group 4
**Skin, Scab, Abdominal (Continued...)**								
First to Last seen	-	-	-	63 - 70	-	-	-	-
**Skin, Scab, Cranium**								
Number of Animals Affected	0	0	0	0	0	0	1	0
Number of Times Recorded	0	0	0	0	0	0	4	0
% of Affected Animals	0	0	0	0	0	0	10	0
First to Last seen	-	-	-	-	-	-	28 - 49	-
**Skin, Scab, Dorsal Cervical**								
Number of Animals Affected	1	0	0	0	0	0	0	0
Number of Times Recorded	2	0	0	0	0	0	0	0
% of Affected Animals	10	0	0	0	0	0	0	0
First to Last seen	35 - 42	-	-	-	-	-	-	-
**Skin, Scab, Dorsal Thoracic**								
Number of Animals Affected	0	0	0	0	0	1	0	0
Number of Times Recorded	0	0	0	0	0	1	0	0
% of Affected Animals	0	0	0	0	0	10	0	0
First to Last seen	-	-	-	-	-	42 - 42	-	-
**Skin, Scab, Face**								
Number of Animals Affected	1	2	1	2	0	1	0	2
Number of Times Recorded	1	21	10	10	0	7	0	9
% of Affected Animals	10	20	10	13	0	10	0	13
First to Last seen	28 - 28	14 - 91	28 - 91	35 - 70	-	14 - 56	-	28 - 56
**Skin, Scab, Mouth**								
Number of Animals Affected	0	0	0	0	1	0	0	1
Number of Times Recorded	0	0	0	0	1	0	0	1
% of Affected Animals	0	0	0	0	10	0	0	7
First to Last seen	-	-	-	-	42 - 42	-	-	28 - 28

Table 1

Summary of Clinical Observations

32025165

Observation Type: Toxicology Observations	Male				Female			
From Day -1 (Start Date) to 91 (Start Date)	0 vg Group 1	1.49x10^11 vg Group 2	4.47x10^11 vg Group 3	1.49x10^12 vg Group 4	0 vg Group 1	1.49x10^11 vg Group 2	4.47x10^11 vg Group 3	1.49x10^12 vg Group 4
**Thin**								
Number of Animals Affected	1	0	0	0	0	0	0	0
Number of Times Recorded	1	0	0	0	0	0	0	0
% of Affected Animals	10	0	0	0	0	0	0	0
First to Last seen	84 - 84	-	-	-	-	-	-	-
**Hypersensitive, Moderate**								
Number of Animals Affected	0	0	0	2	0	0	0	0
Number of Times Recorded	0	0	0	13	0	0	0	0
% of Affected Animals	0	0	0	13	0	0	0	0
First to Last seen	-	-	-	35 - 91	-	-	-	-
**Hypersensitive, Slight**								
Number of Animals Affected	0	1	6	2	0	0	0	2
Number of Times Recorded	0	4	33	6	0	0	0	11
% of Affected Animals	0	10	60	13	0	0	0	13
First to Last seen	-	56 - 77	28 - 91	-1 - 63	-	-	-	28 - 91
**Cold to Touch, Foot, Left**								
Number of Animals Affected	0	0	0	0	0	0	0	1
Number of Times Recorded	0	0	0	0	0	0	0	5
% of Affected Animals	0	0	0	0	0	0	0	7
First to Last seen	-	-	-	-	-	-	-	14 - 42
**Cold to Touch, Foot, Right**								
Number of Animals Affected	0	0	0	0	0	0	0	1
Number of Times Recorded	0	0	0	0	0	0	0	5
% of Affected Animals	0	0	0	0	0	0	0	7
First to Last seen	-	-	-	-	-	-	-	14 - 42
**Cold to Touch, Hindpaw, Left**								
Number of Animals Affected	0	0	0	0	0	0	0	1

Table 1

Summary of Clinical Observations

32025165

Observation Type: Toxicology Observations<br>From Day -1 (Start Date) to 91 (Start Date)	Male<br>0 vg<br>Group 1	Male<br>1.49x10^11 vg<br>Group 2	Male<br>4.47x10^11 vg<br>Group 3	Male<br>1.49x10^12 vg<br>Group 4	Female<br>0 vg<br>Group 1	Female<br>1.49x10^11 vg<br>Group 2	Female<br>4.47x10^11 vg<br>Group 3	Female<br>1.49x10^12 vg<br>Group 4
**Cold to Touch, Hindpaw, Left (Continued...)**								
Number of Times Recorded	0	0	0	0	0	0	0	5
% of Affected Animals	0	0	0	0	0	0	0	7
First to Last seen	-	-	-	-	-	-	-	14 - 42
**Cold to Touch, Hindpaw, Right**								
Number of Animals Affected	0	0	0	0	0	0	0	1
Number of Times Recorded	0	0	0	0	0	0	0	5
% of Affected Animals	0	0	0	0	0	0	0	7
First to Last seen	-	-	-	-	-	-	-	14 - 42
**Cold to Touch, Tail**								
Number of Animals Affected	0	0	0	0	0	0	0	1
Number of Times Recorded	0	0	0	0	0	0	0	2
% of Affected Animals	0	0	0	0	0	0	0	7
First to Last seen	-	-	-	-	-	-	-	14 - 21
**Aggression**								
Number of Animals Affected	0	0	0	1	0	0	0	0
Number of Times Recorded	0	0	0	2	0	0	0	0
% of Affected Animals	0	0	0	7	0	0	0	0
First to Last seen	-	-	-	70 - 77	-	-	-	-
**Hyperreactive, Slight**								
Number of Animals Affected	0	0	0	0	0	0	1	0
Number of Times Recorded	0	0	0	0	0	0	2	0
% of Affected Animals	0	0	0	0	0	0	10	0
First to Last seen	-	-	-	-	-	-	-1 - 7	-
**Fur, Discolored, Dorsal Cervical, Brown**								
Number of Animals Affected	4	0	3	2	1	4	3	2
Number of Times Recorded	19	0	6	7	5	28	29	19

Table 1

Summary of Clinical Observations

32025165

Observation Type: Toxicology Observations<br>From Day -1 (Start Date) to 91 (Start Date)	Male 0 vg Group 1	Male 1.49x10^11 vg Group 2	Male 4.47x10^11 vg Group 3	Male 1.49x10^12 vg Group 4	Female 0 vg Group 1	Female 1.49x10^11 vg Group 2	Female 4.47x10^11 vg Group 3	Female 1.49x10^12 vg Group 4
**Fur, Discolored, Dorsal Cervical, Brown (Continued...)**								
% of Affected Animals	40	0	30	13	10	40	30	13
First to Last seen	28 - 91	-	70 - 84	70 - 91	35 - 63	28 - 91	28 - 91	14 - 91
**Fur, Discolored, Dorsal Thoracic, Red**								
Number of Animals Affected	0	0	1	0	0	0	0	0
Number of Times Recorded	0	0	4	0	0	0	0	0
% of Affected Animals	0	0	10	0	0	0	0	0
First to Last seen	-	-	70 - 90	-	-	-	-	-
**Fur, Discolored, Eyelid, Right, Red**								
Number of Animals Affected	0	0	0	0	1	0	0	0
Number of Times Recorded	0	0	0	0	2	0	0	0
% of Affected Animals	0	0	0	0	10	0	0	0
First to Last seen	-	-	-	-	49 - 56	-	-	-
**Fur, Discolored, Face, Brown**								
Number of Animals Affected	0	0	0	1	1	2	1	1
Number of Times Recorded	0	0	0	4	2	12	5	1
% of Affected Animals	0	0	0	7	10	20	10	7
First to Last seen	-	-	-	70 - 90	28 - 35	28 - 91	56 - 84	28 - 28
**Fur, Discolored, Periorbital, Left, Red**								
Number of Animals Affected	1	0	0	0	0	0	0	0
Number of Times Recorded	7	0	0	0	0	0	0	0
% of Affected Animals	10	0	0	0	0	0	0	0
First to Last seen	49 - 91	-	-	-	-	-	-	-
**Fur, Discolored, Periorbital, Right, Red**								
Number of Animals Affected	1	0	0	0	0	0	0	0
Number of Times Recorded	7	0	0	0	0	0	0	0
% of Affected Animals	10	0	0	0	0	0	0	0

Table 1

Summary of Clinical Observations

32025165

Observation Type: Toxicology Observations	Male				Female			
From Day -1 (Start Date) to 91 (Start Date)	0 vg Group 1	1.49x10^11 vg Group 2	4.47x10^11 vg Group 3	1.49x10^12 vg Group 4	0 vg Group 1	1.49x10^11 vg Group 2	4.47x10^11 vg Group 3	1.49x10^12 vg Group 4
**Fur, Discolored, Periorbital, Right, Red (Continued...)**								
First to Last seen	49 - 91	-	-	-	-	-	-	-

Table 1

Summary of Clinical Observations

32025165

Observation Type: SIRT/Cage Side Observations	Male				Female			
From Day -1 (Start Date) to 91 (Start Date)	0 vg Group 1	1.49x10^11 vg Group 2	4.47x10^11 vg Group 3	1.49x10^12 vg Group 4	0 vg Group 1	1.49x10^11 vg Group 2	4.47x10^11 vg Group 3	1.49x10^12 vg Group 4
**Normal**								
Number of Animals Affected	10	10	10	15	10	10	10	15
Number of Times Recorded	20	20	20	30	20	22	22	30
% of Affected Animals	100	100	100	100	100	100	100	100
First to Last seen	1 - 2	1 - 2	1 - 2	1 - 2	1 - 2	1 - 41	1 - 41	1 - 2

Table 2

Summary of Body Weights

32025165

Bodyweight (g)

Sex: Male		Day(s) Relative to Start Date						
		-1	7	14	21	28	35	42
0 vg	Mean	316.3	356.3	394.0	425.4	450.1	524.4	551.0
	SD	57.4	58.2	59.9	61.1	68.0	68.6	71.1
	N	10	10	10	10	10	5	5
Group 1		-	-	-	-	-	-	-
1.49x10^11 vg	Mean	344.4	380.2	415.7	441.5	470.8	497.8	518.2
	SD	56.1	59.7	66.9	68.9	71.5	76.3	81.9
	N	10	10	10	10	10	10	10
Group 2	%Diff	8.9	6.7	5.5	3.8	4.6	-5.1	-6.0
4.47x10^11 vg	Mean	334.8	369.6	404.9	431.9	457.9	482.4	503.5
	SD	42.1	43.8	46.0	47.1	46.8	49.8	52.4
	N	10	10	10	10	10	10	10
Group 3	%Diff	5.8	3.7	2.8	1.5	1.7	-8.0	-8.6
1.49x10^12 vg	Mean	344.5	381.2	417.7	445.9	467.9	493.5	512.7
	SD	31.5	33.6	38.2	42.3	48.8	56.3	60.4
	N	15	15	15	15	15	10	10
Group 4	%Diff	8.9	7.0	6.0	4.8	3.9	-5.9	-7.0

Day 91 represents data collected on Day 90 and 91.

Table 2

Summary of Body Weights

32025165

Bodyweight (g)

Sex: Male		Day(s) Relative to Start Date						
		49	56	63	70	77	84	91
0 vg	Mean	573.8	596.6	610.4	614.6	586.2	571.4	556.5
	SD	68.3	72.7	73.1	64.1	59.5	76.4	76.5
	N	5	5	5	5	5	5	4
Group 1		-	-	-	-	-	-	-
1.49x10^11	Mean	537.8	556.1	567.7	575.8	591.5	601.0	591.1
vg	SD	84.3	89.0	88.4	89.2	96.0	99.2	92.8
	N	10	10	10	10	10	10	9
Group 2	%Diff	-6.3	-6.8	-7.0	-6.3	0.9	5.2	6.2
4.47x10^11	Mean	523.5	539.0	555.4	565.6	586.0	588.9	612.4
vg	SD	53.0	52.5	55.8	56.4	64.9	64.0	70.1
	N	10	10	10	10	10	10	8
Group 3	%Diff	-8.8	-9.7	-9.0	-8.0	0.0	3.1	10.0
1.49x10^12	Mean	530.8	548.1	564.8	574.5	590.5	598.6	610.1
vg	SD	64.2	68.2	71.2	72.5	77.3	77.7	85.9
	N	10	10	10	10	10	10	8
Group 4	%Diff	-7.5	-8.1	-7.5	-6.5	0.7	4.8	9.6

Day 91 represents data collected on Day 90 and 91.

Table 2

Summary of Body Weights

32025165

Bodyweight (g)

Sex: Female		Day(s) Relative to Start Date						
		-1	7	14	21	28	35	42
0 vg	Mean	211.5	228.2	236.9	244.6	246.3	256.4	258.6
	SD	13.7	17.8	24.0	18.5	18.8	19.0	22.0
	N	10	10	10	10	10	5	5
Group 1		-	-	-	-	-	-	-
1.49x10^11 vg	Mean	239.5	250.0	258.0	259.7	268.4	274.7	276.2
	SD	18.9	20.5	22.3	25.5	27.9	26.5	27.9
	N	10	10	10	10	10	10	10
Group 2	%Diff	13.2	9.6	8.9	6.2	9.0	7.1	6.8
4.47x10^11 vg	Mean	234.5	248.0	254.9	257.2	260.6	268.2	270.1
	SD	21.2	23.8	22.5	25.0	23.8	24.5	21.4
	N	10	10	10	10	10	10	10
Group 3	%Diff	10.9	8.7	7.6	5.2	5.8	4.6	4.4
1.49x10^12 vg	Mean	226.5	242.9	252.3	255.1	259.1	275.0	275.7
	SD	21.4	20.6	19.3	18.5	16.6	18.6	20.3
	N	15	15	15	15	15	10	10
Group 4	%Diff	7.1	6.4	6.5	4.3	5.2	7.3	6.6

Day 91 represents data collected on Day 90 and 91.

Table 2

Summary of Body Weights

32025165

Bodyweight (g)

Sex: Female		Day(s) Relative to Start Date						
		49	56	63	70	77	84	91
0 vg	Mean	261.2	268.4	267.6	274.0	270.8	273.0	275.4
	SD	18.5	19.1	20.7	20.9	22.7	20.9	22.8
	N	5	5	5	5	5	5	5
Group 1		-	-	-	-	-	-	-
1.49x10^11	Mean	278.8	288.0	290.3	296.5	294.7	297.4	302.1
vg	SD	30.2	28.7	29.9	29.2	28.2	26.8	32.7
	N	10	10	10	10	10	10	10
Group 2	%Diff	6.7	7.3	8.5	8.2	8.8	8.9	9.7
4.47x10^11	Mean	272.4	279.0	281.1	290.7	286.9	289.9	289.4
vg	SD	19.8	25.4	24.1	26.1	32.1	30.7	31.4
	N	10	10	10	10	10	10	10
Group 3	%Diff	4.3	3.9	5.0	6.1	5.9	6.2	5.1
1.49x10^12	Mean	275.2	280.2	287.4	292.3	288.6	294.9	294.4
vg	SD	21.2	23.3	19.1	19.9	19.4	22.5	19.9
	N	10	10	10	10	10	10	10
Group 4	%Diff	5.4	4.4	7.4	6.7	6.6	8.0	6.9

Day 91 represents data collected on Day 90 and 91.

Table 3

Summary of Body Weight Gains (g)

32025165
Bodyweight Gain (Interval)

Sex: Male		Day(s) Relative to Start Date						
		-1 → 7	7 → 14	14 → 21	21 → 28	28 → 35	35 → 42	42 → 49
0 vg	Mean	40.0	37.7	31.4	24.7	28.0	26.6	22.8
	SD	11.7	7.7	11.8	11.4	6.4	4.0	5.8
	N	10	10	10	10	5	5	5
Group 1		-	-	-	-	-	-	-
1.49x10^11	Mean	35.8	35.5	25.8	29.3	27.0	20.4	19.6
vg	SD	7.0	11.8	10.2	5.0	6.1	8.4	4.2
	N	10	10	10	10	10	10	10
Group 2		-	-	-	-	-	-	-
4.47x10^11	Mean	34.8	35.3	27.0	26.0	24.5	21.1	20.0
vg	SD	8.9	9.2	4.9	6.7	7.6	4.8	5.3
	N	10	10	10	10	10	10	10
Group 3		-	-	-	-	-	-	-
1.49x10^12	Mean	36.7	36.5	28.1	22.0	20.8	19.2	18.1
vg	SD	9.3	8.1	7.7	9.1	9.1	6.1	8.4
	N	15	15	15	15	10	10	10
Group 4		-	-	-	-	-	-	-

Day 91 represents data collected on Day 90 and 91.

Table 3

Summary of Body Weight Gains (g)

32025165

Bodyweight Gain (Interval)

Sex: Male		Day(s) Relative to Start Date					
		49 → 56	56 → 63	63 → 70	70 → 77	77 → 84	84 → 91
0 vg	Mean	22.8	13.8	4.2	-28.4	-14.8	4.0
	SD	5.2	3.8	15.3	56.9	34.5	7.8
	N	5	5	5	5	5	4
Group 1		-	-	-	-	-	-
1.49x10^11 vg	Mean	18.3	11.6	8.1	15.7	9.5	5.4
	SD	6.6	10.3	10.8	8.9	6.9	10.6
	N	10	10	10	10	10	9
Group 2		-	-	-	-	-	-
4.47x10^11 vg	Mean	15.5	16.4	10.2	20.4	2.9	13.4
	SD	5.4	7.3	7.9	11.3	8.5	7.4
	N	10	10	10	10	10	8
Group 3		-	-	-	-	-	-
1.49x10^12 vg	Mean	17.3	16.7	9.7	16.0	8.1	14.6
	SD	6.8	6.8	8.5	10.6	13.5	7.6
	N	10	10	10	10	10	8
Group 4		-	-	-	-	-	-

Day 91 represents data collected on Day 90 and 91.

Table 3

Summary of Body Weight Gains (g)

32025165

Bodyweight Gain (Interval)

Sex: Female		Day(s) Relative to Start Date						
		-1 → 7	7 → 14	14 → 21	21 → 28	28 → 35	35 → 42	42 → 49
0 vg	Mean	16.7	8.7	7.7	1.7	9.8	2.2	2.6
	SD	9.3	8.3	11.7	10.0	3.3	4.3	4.8
	N	10	10	10	10	5	5	5
Group 1		-	-	-	-	-	-	-
1.49x10^11 vg	Mean	10.5	8.0	1.7	8.7	6.3	1.5	2.6
	SD	6.8	6.4	5.8	8.0	7.0	4.5	6.2
	N	10	10	10	10	10	10	10
Group 2		-	-	-	-	-	-	-
4.47x10^11 vg	Mean	13.5	6.9	2.3	3.4	7.6	1.9	2.3
	SD	6.5	3.6	5.3	6.9	7.9	9.2	6.4
	N	10	10	10	10	10	10	10
Group 3		-	-	-	-	-	-	-
1.49x10^12 vg	Mean	16.3	9.4	2.8	4.0	11.2	0.7	-0.5
	SD	7.4	6.1	3.8	6.8	4.1	5.0	3.6
	N	15	15	15	15	10	10	10
Group 4		-	-	-	-	-	-	-

Day 91 represents data collected on Day 90 and 91.

Table 3

Summary of Body Weight Gains (g)

32025165

Bodyweight Gain (Interval)

Sex: Female		Day(s) Relative to Start Date					
		49 → 56	56 → 63	63 → 70	70 → 77	77 → 84	84 → 91
0 vg	Mean	7.2	-0.8	6.4	-3.2	2.2	2.4
	SD	7.4	6.1	4.6	3.7	3.0	5.0
	N	5	5	5	5	5	5
Group 1		-	-	-	-	-	-
1.49x10^11 vg	Mean	9.2	2.3	6.2	-1.8	2.7	4.7
	SD	7.0	8.5	3.9	5.1	6.4	9.1
	N	10	10	10	10	10	10
Group 2		-	-	-	-	-	-
4.47x10^11 vg	Mean	6.6	2.1	9.6	-3.8	3.0	-0.5
	SD	7.7	4.7	4.5	7.6	5.8	8.2
	N	10	10	10	10	10	10
Group 3		-	-	-	-	-	-
1.49x10^12 vg	Mean	5.0	7.2	4.9	-3.7	6.3	-0.5
	SD	10.5	7.5	3.0	4.6	6.7	4.8
	N	10	10	10	10	10	10
Group 4		-	-	-	-	-	-

Day 91 represents data collected on Day 90 and 91.

Table 4

Summary of Neurobehavioral Evaluation: Activity

32025165
Day: -1 Relative to Start Date

Sex: Male		0 vg<br>Group 1	1.49x10^11 vg<br>Group 2	4.47x10^11 vg<br>Group 3	1.49x10^12 vg<br>Group 4
Arousal/Alertness	N	10	10	10	15
-2 Very low	N+ve	0	0	0	0
-1 Low	N+ve	0	0	0	0
0 Normal	N+ve	10	10	10	15
1 High	N+ve	0	0	0	0
2 Very High	N+ve	0	0	0	0
Posture/Body Carriage	N	10	10	10	15
-2 Prostrate or flattened	N+ve	0	0	0	0
-1 Hunched or lying low	N+ve	0	0	0	0
0 Normal (asleep/sit/stand)	N+ve	10	10	10	15
Stereotypy (Home Cage)	N	10	10	10	15
0 Not present; Normal	N+ve	10	10	10	15
1 Licking, biting, grooming	N+ve	0	0	0	0
2 Mouth movements/weaving	N+ve	0	0	0	0
3 Circling, retropulsion	N+ve	0	0	0	0
4 Self-mutilation	N+ve	0	0	0	0

Day 27 represents data collected from Day 27 to 32.

Table 4

Summary of Neurobehavioral Evaluation: Activity

32025165
Day: -1 Relative to Start Date

Sex: Male		0 vg<br><br>Group 1	1.49x10^11 vg<br><br>Group 2	4.47x10^11 vg<br><br>Group 3	1.49x10^12 vg<br><br>Group 4
Stereotypy (Open Field)	N	10	10	10	15
0 Not present; Normal	N+ve	10	10	10	15
1 Licking, biting, grooming	N+ve	0	0	0	0
2 Mouth movements/weaving	N+ve	0	0	0	0
3 Circling, retropulsion	N+ve	0	0	0	0
4 Self-mutilation	N+ve	0	0	0	0
Rearing	Mean<br>SD<br>N<br>%Diff	6.3<br>4.3<br>10<br>-	6.8<br>2.3<br>10<br>7.9	4.8<br>2.4<br>10<br>-23.8	7.3<br>3.0<br>15<br>15.3
Appearance	N	10	10	10	15
0 Clean and groomed	N+ve	10	10	10	15
1 Slightly unkempt	N+ve	0	0	0	0
2 Extremely unkempt	N+ve	0	0	0	0

Day 27 represents data collected from Day 27 to 32.

Table 4

Summary of Neurobehavioral Evaluation: Activity

32025165
Day: -1 Relative to Start Date

Sex: Female		0 vg<br>Group 1	1.49x10^11 vg<br>Group 2	4.47x10^11 vg<br>Group 3	1.49x10^12 vg<br>Group 4
Arousal/Alertness	N	10	10	10	15
-2 Very low	N+ve	0	0	0	0
-1 Low	N+ve	0	0	0	0
0 Normal	N+ve	10	10	10	15
1 High	N+ve	0	0	0	0
2 Very High	N+ve	0	0	0	0
Posture/Body Carriage	N	10	10	10	15
-2 Prostrate or flattened	N+ve	0	0	0	0
-1 Hunched or lying low	N+ve	0	0	0	0
0 Normal (asleep/sit/stand)	N+ve	10	10	10	15
Stereotypy (Home Cage)	N	10	10	10	15
0 Not present; Normal	N+ve	10	10	10	15
1 Licking, biting, grooming	N+ve	0	0	0	0
2 Mouth movements/weaving	N+ve	0	0	0	0
3 Circling, retropulsion	N+ve	0	0	0	0
4 Self-mutilation	N+ve	0	0	0	0

Day 27 represents data collected from Day 27 to 32.

Table 4

Summary of Neurobehavioral Evaluation: Activity

32025165
Day: -1 Relative to Start Date

Sex: Female		0 vg<br><br>Group 1	1.49x10^11 vg<br><br>Group 2	4.47x10^11 vg<br><br>Group 3	1.49x10^12 vg<br><br>Group 4
Stereotypy (Open Field)	N	10	10	10	15
0 Not present; Normal	N+ve	10	10	10	15
1 Licking, biting, grooming	N+ve	0	0	0	0
2 Mouth movements/weaving	N+ve	0	0	0	0
3 Circling, retropulsion	N+ve	0	0	0	0
4 Self-mutilation	N+ve	0	0	0	0
Rearing	Mean	11.9	8.2	9.4	10.7
	SD	3.2	1.8	2.9	3.2
	N	10	10	10	15
	%Diff	-	-31.1	-21.0	-9.8
Appearance	N	10	10	10	15
0 Clean and groomed	N+ve	10	10	10	15
1 Slightly unkempt	N+ve	0	0	0	0
2 Extremely unkempt	N+ve	0	0	0	0

Day 27 represents data collected from Day 27 to 32.

Table 4

Summary of Neurobehavioral Evaluation: Activity

32025165
Day: 27 Relative to Start Date

Sex: Male		0 vg<br>Group 1	1.49x10^11 vg<br>Group 2	4.47x10^11 vg<br>Group 3	1.49x10^12 vg<br>Group 4
Arousal/Alertness	N	10	10	10	15
-2 Very low	N+ve	0	0	0	0
-1 Low	N+ve	0	2	5	4
0 Normal	N+ve	10	8	5	11
1 High	N+ve	0	0	0	0
2 Very High	N+ve	0	0	0	0
Posture/Body Carriage	N	10	10	10	15
-2 Prostrate or flattened	N+ve	0	0	0	0
-1 Hunched or lying low	N+ve	0	0	0	0
0 Normal (asleep/sit/stand)	N+ve	10	10	10	15
Stereotypy (Home Cage)	N	10	10	10	15
0 Not present; Normal	N+ve	10	10	10	15
1 Licking, biting, grooming	N+ve	0	0	0	0
2 Mouth movements/weaving	N+ve	0	0	0	0
3 Circling, retropulsion	N+ve	0	0	0	0
4 Self-mutilation	N+ve	0	0	0	0

Day 27 represents data collected from Day 27 to 32.

Table 4

Summary of Neurobehavioral Evaluation: Activity

32025165
Day: 27 Relative to Start Date

Sex: Male		0 vg<br>Group 1	1.49x10^11 vg<br>Group 2	4.47x10^11 vg<br>Group 3	1.49x10^12 vg<br>Group 4
Stereotypy (Open Field)	N	10	10	10	15
0 Not present; Normal	N+ve	10	10	10	15
1 Licking, biting, grooming	N+ve	0	0	0	0
2 Mouth movements/weaving	N+ve	0	0	0	0
3 Circling, retropulsion	N+ve	0	0	0	0
4 Self-mutilation	N+ve	0	0	0	0
Rearing	Mean	5.5	7.6	4.3	5.6
	SD	4.7	4.8	4.4	4.7
	N	10	10	10	15
	%Diff	-	38.2	-21.8	1.8
Appearance	N	10	10	10	15
0 Clean and groomed	N+ve	10	10	10	15
1 Slightly unkempt	N+ve	0	0	0	0
2 Extremely unkempt	N+ve	0	0	0	0

Day 27 represents data collected from Day 27 to 32.

Table 4

Summary of Neurobehavioral Evaluation: Activity

32025165
Day: 27 Relative to Start Date

Sex: Female		0 vg<br>Group 1	1.49x10^11 vg<br>Group 2	4.47x10^11 vg<br>Group 3	1.49x10^12 vg<br>Group 4
Arousal/Alertness	N	10	10	10	15
-2 Very low	N+ve	0	0	0	0
-1 Low	N+ve	0	1	1	1
0 Normal	N+ve	10	9	9	14
1 High	N+ve	0	0	0	0
2 Very High	N+ve	0	0	0	0
Posture/Body Carriage	N	10	10	10	15
-2 Prostrate or flattened	N+ve	0	0	0	0
-1 Hunched or lying low	N+ve	0	0	1	0
0 Normal (asleep/sit/stand)	N+ve	10	10	9	15
Stereotypy (Home Cage)	N	10	10	10	15
0 Not present; Normal	N+ve	10	10	10	15
1 Licking, biting, grooming	N+ve	0	0	0	0
2 Mouth movements/weaving	N+ve	0	0	0	0
3 Circling, retropulsion	N+ve	0	0	0	0
4 Self-mutilation	N+ve	0	0	0	0

Day 27 represents data collected from Day 27 to 32.

Table 4

Summary of Neurobehavioral Evaluation: Activity

32025165
Day: 27 Relative to Start Date

Sex: Female		0 vg<br>Group 1	1.49x10^11 vg<br>Group 2	4.47x10^11 vg<br>Group 3	1.49x10^12 vg<br>Group 4
Stereotypy (Open Field)	N	10	10	10	15
0 Not present; Normal	N+ve	10	10	10	15
1 Licking, biting, grooming	N+ve	0	0	0	0
2 Mouth movements/weaving	N+ve	0	0	0	0
3 Circling, retropulsion	N+ve	0	0	0	0
4 Self-mutilation	N+ve	0	0	0	0
Rearing	Mean	9.7	8.8	7.2	8.7
	SD	5.3	4.4	6.1	4.4
	N	10	10	10	15
	%Diff	-	-9.3	-25.8	-10.7
Appearance	N	10	10	10	15
0 Clean and groomed	N+ve	10	10	10	15
1 Slightly unkempt	N+ve	0	0	0	0
2 Extremely unkempt	N+ve	0	0	0	0

Day 27 represents data collected from Day 27 to 32.

Table 4

Summary of Neurobehavioral Evaluation: Activity

32025165
Day: 88 Relative to Start Date

Sex: Male		0 vg<br>Group 1	1.49x10^11 vg<br>Group 2	4.47x10^11 vg<br>Group 3	1.49x10^12 vg<br>Group 4
Arousal/Alertness	N	5	10	10	10
-2 Very low	N+ve	0	0	0	0
-1 Low	N+ve	1	2	6	2
0 Normal	N+ve	4	8	4	7
1 High	N+ve	0	0	0	1
2 Very High	N+ve	0	0	0	0
Posture/Body Carriage	N	5	10	10	10
-2 Prostrate or flattened	N+ve	0	0	0	0
-1 Hunched or lying low	N+ve	0	0	0	0
0 Normal (asleep/sit/stand)	N+ve	5	10	10	10
Stereotypy (Home Cage)	N	5	10	10	10
0 Not present; Normal	N+ve	5	10	10	10
1 Licking, biting, grooming	N+ve	0	0	0	0
2 Mouth movements/weaving	N+ve	0	0	0	0
3 Circling, retropulsion	N+ve	0	0	0	0
4 Self-mutilation	N+ve	0	0	0	0

Day 27 represents data collected from Day 27 to 32.

Table 4

Summary of Neurobehavioral Evaluation: Activity

32025165
Day: 88 Relative to Start Date

Sex: Male		0 vg<br><br>Group 1	1.49x10^11 vg<br><br>Group 2	4.47x10^11 vg<br><br>Group 3	1.49x10^12 vg<br><br>Group 4
Stereotypy (Open Field)	N	5	10	10	10
0 Not present; Normal	N+ve	5	10	10	10
1 Licking, biting, grooming	N+ve	0	0	0	0
2 Mouth movements/weaving	N+ve	0	0	0	0
3 Circling, retropulsion	N+ve	0	0	0	0
4 Self-mutilation	N+ve	0	0	0	0
Rearing	Mean<br>SD<br>N<br>%Diff	6.0<br>4.2<br>5<br>-	6.7<br>4.1<br>10<br>11.7	3.2<br>2.5<br>10<br>-46.7	6.9<br>4.2<br>10<br>15.0
Appearance	N	5	10	10	10
0 Clean and groomed	N+ve	4	10	10	10
1 Slightly unkempt	N+ve	1	0	0	0
2 Extremely unkempt	N+ve	0	0	0	0

Day 27 represents data collected from Day 27 to 32.

Table 4

Summary of Neurobehavioral Evaluation: Activity

32025165
Day: 89 Relative to Start Date

Sex: Female		0 vg<br><br>Group 1	1.49x10^11 vg<br><br>Group 2	4.47x10^11 vg<br><br>Group 3	1.49x10^12 vg<br><br>Group 4
Arousal/Alertness	N	5	10	10	10
-2 Very low	N+ve	0	0	0	0
-1 Low	N+ve	0	0	1	0
0 Normal	N+ve	5	10	9	10
1 High	N+ve	0	0	0	0
2 Very High	N+ve	0	0	0	0
Posture/Body Carriage	N	5	10	10	10
-2 Prostrate or flattened	N+ve	0	0	0	0
-1 Hunched or lying low	N+ve	0	0	0	0
0 Normal (asleep/sit/stand)	N+ve	5	10	10	10
Stereotypy (Home Cage)	N	5	10	10	10
0 Not present; Normal	N+ve	5	10	10	10
1 Licking, biting, grooming	N+ve	0	0	0	0
2 Mouth movements/weaving	N+ve	0	0	0	0
3 Circling, retropulsion	N+ve	0	0	0	0
4 Self-mutilation	N+ve	0	0	0	0

Day 27 represents data collected from Day 27 to 32.

Table 4

Summary of Neurobehavioral Evaluation: Activity

32025165
Day: 89 Relative to Start Date

Sex: Female		0 vg<br>Group 1	1.49x10^11 vg<br>Group 2	4.47x10^11 vg<br>Group 3	1.49x10^12 vg<br>Group 4
Stereotypy (Open Field)	N	5	10	10	10
0 Not present; Normal	N+ve	5	10	10	10
1 Licking, biting, grooming	N+ve	0	0	0	0
2 Mouth movements/weaving	N+ve	0	0	0	0
3 Circling, retropulsion	N+ve	0	0	0	0
4 Self-mutilation	N+ve	0	0	0	0
Rearing	Mean	9.4	9.8	7.5	9.9
	SD	1.7	2.8	4.9	2.8
	N	5	10	10	10
	%Diff	-	4.3	-20.2	5.3
Appearance	N	5	10	10	10
0 Clean and groomed	N+ve	5	10	10	10
1 Slightly unkempt	N+ve	0	0	0	0
2 Extremely unkempt	N+ve	0	0	0	0

Day 27 represents data collected from Day 27 to 32.

Table 5

Summary of Neurobehavioral Evaluation: Autonomic

32025165
Day: -1 Relative to Start Date

Sex: Male		0 vg<br><br>Group 1	1.49x10^11 vg<br><br>Group 2	4.47x10^11 vg<br><br>Group 3	1.49x10^12 vg<br><br>Group 4
Exophthalmus	N	10	10	10	15
0 Not present	N+ve	10	10	10	15
1 Present	N+ve	0	0	0	0
Lacrimation	N	10	10	10	15
0 None	N+ve	10	10	10	15
1 Slight	N+ve	0	0	0	0
2 Severe	N+ve	0	0	0	0
Erected Fur	N	10	10	10	15
0 Not present	N+ve	10	9	9	15
1 Present	N+ve	0	1	1	0
Pupil Response	N	10	10	10	15
0 Normal constriction	N+ve	10	10	10	15
1 Partial/no constriction	N+ve	0	0	0	0
2 Pre-existing constriction	N+ve	0	0	0	0
Salivation	N	10	10	10	15
0 None	N+ve	10	10	10	15
1 Slight	N+ve	0	0	0	0
2 Severe	N+ve	0	0	0	0

Day 27 represents data collected from Day 27 to 32.

Table 5

Summary of Neurobehavioral Evaluation: Autonomic

32025165
Day: -1 Relative to Start Date

Sex: Male		0 vg<br>Group 1	1.49x10^11 vg<br>Group 2	4.47x10^11 vg<br>Group 3	1.49x10^12 vg<br>Group 4
Defecation	N	10	10	10	15
-1 Absent	N+ve	7	6	6	9
0 Normal	N+ve	3	4	4	6
1 Abnormal	N+ve	0	0	0	0
Palpebral Closure/Ptosis (HC)	N	10	10	10	15
0 Eyelids wide open	N+ve	10	10	10	15
1 Eyelids droop halfway	N+ve	0	0	0	0
2 Eyelids completely shut	N+ve	0	0	0	0
Palpebral Closure/Ptosis (OF)	N	10	10	10	15
0 Eyelids wide open	N+ve	10	10	10	15
1 Eyelids droop halfway	N+ve	0	0	0	0
2 Eyelids completely shut	N+ve	0	0	0	0

Day 27 represents data collected from Day 27 to 32.

Table 5

Summary of Neurobehavioral Evaluation: Autonomic

32025165
Day: -1 Relative to Start Date

Sex: Female		0 vg<br><br>Group 1	1.49x10^11 vg<br><br>Group 2	4.47x10^11 vg<br><br>Group 3	1.49x10^12 vg<br><br>Group 4
Exophthalmus	N	10	10	10	15
0 Not present	N+ve	10	10	10	15
1 Present	N+ve	0	0	0	0
Lacrimation	N	10	10	10	15
0 None	N+ve	10	10	10	15
1 Slight	N+ve	0	0	0	0
2 Severe	N+ve	0	0	0	0
Erected Fur	N	10	10	10	15
0 Not present	N+ve	10	10	10	15
1 Present	N+ve	0	0	0	0
Pupil Response	N	10	10	10	15
0 Normal constriction	N+ve	10	10	10	15
1 Partial/no constriction	N+ve	0	0	0	0
2 Pre-existing constriction	N+ve	0	0	0	0
Salivation	N	10	10	10	15
0 None	N+ve	10	10	10	15
1 Slight	N+ve	0	0	0	0
2 Severe	N+ve	0	0	0	0

Day 27 represents data collected from Day 27 to 32.

Table 5

Summary of Neurobehavioral Evaluation: Autonomic

32025165
Day: -1 Relative to Start Date

Sex: Female		0 vg<br><br>Group 1	1.49x10^11 vg<br><br>Group 2	4.47x10^11 vg<br><br>Group 3	1.49x10^12 vg<br><br>Group 4
Defecation	N	10	10	10	15
-1 Absent	N+ve	10	9	10	15
0 Normal	N+ve	0	1	0	0
1 Abnormal	N+ve	0	0	0	0
Palpebral Closure/Ptosis (HC)	N	10	10	10	15
0 Eyelids wide open	N+ve	10	10	10	15
1 Eyelids droop halfway	N+ve	0	0	0	0
2 Eyelids completely shut	N+ve	0	0	0	0
Palpebral Closure/Ptosis (OF)	N	10	10	10	15
0 Eyelids wide open	N+ve	10	10	10	15
1 Eyelids droop halfway	N+ve	0	0	0	0
2 Eyelids completely shut	N+ve	0	0	0	0

Day 27 represents data collected from Day 27 to 32.

Table 5

Summary of Neurobehavioral Evaluation: Autonomic

32025165
Day: 27 Relative to Start Date

Sex: Male		0 vg<br><br>Group 1	1.49x10^11 vg<br><br>Group 2	4.47x10^11 vg<br><br>Group 3	1.49x10^12 vg<br><br>Group 4
Exophthalmus	N	10	10	10	15
0 Not present	N+ve	10	10	10	15
1 Present	N+ve	0	0	0	0
Lacrimation	N	10	10	10	15
0 None	N+ve	10	10	10	15
1 Slight	N+ve	0	0	0	0
2 Severe	N+ve	0	0	0	0
Erected Fur	N	10	10	10	15
0 Not present	N+ve	10	10	10	15
1 Present	N+ve	0	0	0	0
Pupil Response	N	10	10	10	15
0 Normal constriction	N+ve	10	10	10	14
1 Partial/no constriction	N+ve	0	0	0	1
2 Pre-existing constriction	N+ve	0	0	0	0
Salivation	N	10	10	10	15
0 None	N+ve	10	10	10	15
1 Slight	N+ve	0	0	0	0
2 Severe	N+ve	0	0	0	0

Day 27 represents data collected from Day 27 to 32.

Table 5

Summary of Neurobehavioral Evaluation: Autonomic

32025165
Day: 27 Relative to Start Date

Sex: Male		0 vg<br>Group 1	1.49x10^11 vg<br>Group 2	4.47x10^11 vg<br>Group 3	1.49x10^12 vg<br>Group 4
Defecation	N	10	10	10	15
-1 Absent	N+ve	7	7	6	11
0 Normal	N+ve	3	3	4	4
1 Abnormal	N+ve	0	0	0	0
Palpebral Closure/Ptosis (HC)	N	10	10	10	15
0 Eyelids wide open	N+ve	10	10	10	14
1 Eyelids droop halfway	N+ve	0	0	0	1
2 Eyelids completely shut	N+ve	0	0	0	0
Palpebral Closure/Ptosis (OF)	N	10	10	10	15
0 Eyelids wide open	N+ve	10	10	10	15
1 Eyelids droop halfway	N+ve	0	0	0	0
2 Eyelids completely shut	N+ve	0	0	0	0

Day 27 represents data collected from Day 27 to 32.

Table 5

Summary of Neurobehavioral Evaluation: Autonomic

32025165
Day: 27 Relative to Start Date

Sex: Female		0 vg<br>Group 1	1.49x10^11 vg<br>Group 2	4.47x10^11 vg<br>Group 3	1.49x10^12 vg<br>Group 4
Exophthalmus	N	10	10	10	15
0 Not present	N+ve	10	10	10	15
1 Present	N+ve	0	0	0	0
Lacrimation	N	10	10	10	15
0 None	N+ve	10	10	10	15
1 Slight	N+ve	0	0	0	0
2 Severe	N+ve	0	0	0	0
Erected Fur	N	10	10	10	15
0 Not present	N+ve	10	10	10	15
1 Present	N+ve	0	0	0	0
Pupil Response	N	10	10	10	15
0 Normal constriction	N+ve	9	10	10	14
1 Partial/no constriction	N+ve	1	0	0	0
2 Pre-existing constriction	N+ve	0	0	0	1
Salivation	N	10	10	10	15
0 None	N+ve	10	10	10	15
1 Slight	N+ve	0	0	0	0
2 Severe	N+ve	0	0	0	0

Day 27 represents data collected from Day 27 to 32.

Table 5

Summary of Neurobehavioral Evaluation: Autonomic

32025165
Day: 27 Relative to Start Date

Sex: Female		0 vg<br><br>Group 1	1.49x10^11 vg<br><br>Group 2	4.47x10^11 vg<br><br>Group 3	1.49x10^12 vg<br><br>Group 4
Defecation	N	10	10	10	15
-1 Absent	N+ve	8	5	4	11
0 Normal	N+ve	2	5	6	4
1 Abnormal	N+ve	0	0	0	0
Palpebral Closure/Ptosis (HC)	N	10	10	10	15
0 Eyelids wide open	N+ve	10	10	10	15
1 Eyelids droop halfway	N+ve	0	0	0	0
2 Eyelids completely shut	N+ve	0	0	0	0
Palpebral Closure/Ptosis (OF)	N	10	10	10	15
0 Eyelids wide open	N+ve	10	10	10	15
1 Eyelids droop halfway	N+ve	0	0	0	0
2 Eyelids completely shut	N+ve	0	0	0	0

Day 27 represents data collected from Day 27 to 32.

Table 5

Summary of Neurobehavioral Evaluation: Autonomic

32025165
Day: 88 Relative to Start Date

Sex: Male		0 vg<br>Group 1	1.49x10^11 vg<br>Group 2	4.47x10^11 vg<br>Group 3	1.49x10^12 vg<br>Group 4
Exophthalmus	N	5	10	10	10
0 Not present	N+ve	5	10	10	10
1 Present	N+ve	0	0	0	0
Lacrimation	N	5	10	10	10
0 None	N+ve	5	10	10	10
1 Slight	N+ve	0	0	0	0
2 Severe	N+ve	0	0	0	0
Erected Fur	N	5	10	10	10
0 Not present	N+ve	5	10	10	10
1 Present	N+ve	0	0	0	0
Pupil Response	N	5	10	10	10
0 Normal constriction	N+ve	5	10	10	10
1 Partial/no constriction	N+ve	0	0	0	0
2 Pre-existing constriction	N+ve	0	0	0	0
Salivation	N	5	10	10	10
0 None	N+ve	5	10	10	10
1 Slight	N+ve	0	0	0	0
2 Severe	N+ve	0	0	0	0

Day 27 represents data collected from Day 27 to 32.

Table 5

Summary of Neurobehavioral Evaluation: Autonomic

32025165
Day: 88 Relative to Start Date

Sex: Male		0 vg<br>Group 1	1.49x10^11 vg<br>Group 2	4.47x10^11 vg<br>Group 3	1.49x10^12 vg<br>Group 4
Defecation	N	5	10	10	10
-1 Absent	N+ve	4	7	6	7
0 Normal	N+ve	1	3	4	3
1 Abnormal	N+ve	0	0	0	0
Palpebral Closure/Ptosis (HC)	N	5	10	10	10
0 Eyelids wide open	N+ve	5	10	10	10
1 Eyelids droop halfway	N+ve	0	0	0	0
2 Eyelids completely shut	N+ve	0	0	0	0
Palpebral Closure/Ptosis (OF)	N	5	10	10	10
0 Eyelids wide open	N+ve	5	10	10	10
1 Eyelids droop halfway	N+ve	0	0	0	0
2 Eyelids completely shut	N+ve	0	0	0	0

Day 27 represents data collected from Day 27 to 32.

Table 5

Summary of Neurobehavioral Evaluation: Autonomic

32025165
Day: 89 Relative to Start Date

Sex: Female		0 vg<br>Group 1	1.49x10^11 vg<br>Group 2	4.47x10^11 vg<br>Group 3	1.49x10^12 vg<br>Group 4
Exophthalmus	N	5	10	10	10
0 Not present	N+ve	5	10	10	10
1 Present	N+ve	0	0	0	0
Lacrimation	N	5	10	10	10
0 None	N+ve	5	10	10	10
1 Slight	N+ve	0	0	0	0
2 Severe	N+ve	0	0	0	0
Erected Fur	N	5	10	10	10
0 Not present	N+ve	5	10	10	10
1 Present	N+ve	0	0	0	0
Pupil Response	N	5	10	10	10
0 Normal constriction	N+ve	5	10	10	10
1 Partial/no constriction	N+ve	0	0	0	0
2 Pre-existing constriction	N+ve	0	0	0	0
Salivation	N	5	10	10	10
0 None	N+ve	5	10	10	10
1 Slight	N+ve	0	0	0	0
2 Severe	N+ve	0	0	0	0

Day 27 represents data collected from Day 27 to 32.

Table 5

Summary of Neurobehavioral Evaluation: Autonomic

32025165
Day: 89 Relative to Start Date

Sex: Female		0 vg<br><br>Group 1	1.49x10^11 vg<br><br>Group 2	4.47x10^11 vg<br><br>Group 3	1.49x10^12 vg<br><br>Group 4
Defecation	N	5	10	10	10
-1 Absent	N+ve	5	9	10	10
0 Normal	N+ve	0	1	0	0
1 Abnormal	N+ve	0	0	0	0
Palpebral Closure/Ptosis (HC)	N	5	10	10	10
0 Eyelids wide open	N+ve	5	10	10	10
1 Eyelids droop halfway	N+ve	0	0	0	0
2 Eyelids completely shut	N+ve	0	0	0	0
Palpebral Closure/Ptosis (OF)	N	5	10	10	10
0 Eyelids wide open	N+ve	5	10	10	10
1 Eyelids droop halfway	N+ve	0	0	0	0
2 Eyelids completely shut	N+ve	0	0	0	0

Day 27 represents data collected from Day 27 to 32.

Table 6

Summary of Neurobehavioral Evaluation: Excitability

32025165
Day: -1 Relative to Start Date

Sex: Male		0 vg<br><br>Group 1	1.49x10^11 vg<br><br>Group 2	4.47x10^11 vg<br><br>Group 3	1.49x10^12 vg<br><br>Group 4
Vocalizations	N	10	10	10	15
0 Once/not at all	N+ve	10	10	10	15
1 More than once	N+ve	0	0	0	0
Startle Response	N	10	10	10	15
-1 No reaction	N+ve	0	0	0	0
0 Normal reaction	N+ve	10	10	10	15
1 Exaggerated Reaction	N+ve	0	0	0	0
Ease of Removal	N	10	10	10	15
-1 Very easy	N+ve	0	0	0	0
0 Easy	N+ve	10	9	9	14
1 Moderate	N+ve	0	1	1	1
2 Difficult	N+ve	0	0	0	0
Handling Reactivity	N	10	10	10	15
-1 Very low	N+ve	0	0	0	0
0 Low	N+ve	10	10	9	14
1 Moderate	N+ve	0	0	1	1
2 High	N+ve	0	0	0	0

Day 27 represents data collected from Day 27 to 32.

Table 6

Summary of Neurobehavioral Evaluation: Excitability

32025165
Day: -1 Relative to Start Date

Sex: Male		0 vg<br><br>Group 1	1.49x10^11 vg<br><br>Group 2	4.47x10^11 vg<br><br>Group 3	1.49x10^12 vg<br><br>Group 4
Convulsions (Home Cage)	N	10	10	10	15
0 None	N+ve	10	10	10	15
1 Clonic movement	N+ve	0	0	0	0
2 Clonic tremors	N+ve	0	0	0	0
3 Severe clonus	N+ve	0	0	0	0
4 Whole body convulsions	N+ve	0	0	0	0

Day 27 represents data collected from Day 27 to 32.

Table 6

Summary of Neurobehavioral Evaluation: Excitability

32025165
Day: -1 Relative to Start Date

Sex: Female		0 vg<br>Group 1	1.49x10^11 vg<br>Group 2	4.47x10^11 vg<br>Group 3	1.49x10^12 vg<br>Group 4
Vocalizations	N	10	10	10	15
0 Once/not at all	N+ve	10	10	10	15
1 More than once	N+ve	0	0	0	0
Startle Response	N	10	10	10	15
-1 No reaction	N+ve	0	0	0	0
0 Normal reaction	N+ve	10	10	10	15
1 Exaggerated Reaction	N+ve	0	0	0	0
Ease of Removal	N	10	10	10	15
-1 Very easy	N+ve	0	0	0	0
0 Easy	N+ve	10	10	8	15
1 Moderate	N+ve	0	0	2	0
2 Difficult	N+ve	0	0	0	0
Handling Reactivity	N	10	10	10	15
-1 Very low	N+ve	0	0	0	0
0 Low	N+ve	10	10	9	15
1 Moderate	N+ve	0	0	1	0
2 High	N+ve	0	0	0	0

Day 27 represents data collected from Day 27 to 32.

Table 6

Summary of Neurobehavioral Evaluation: Excitability

32025165
Day: -1 Relative to Start Date

Sex: Female		0 vg<br><br>Group 1	1.49x10^11 vg<br><br>Group 2	4.47x10^11 vg<br><br>Group 3	1.49x10^12 vg<br><br>Group 4
Convulsions (Home Cage)	N	10	10	10	15
0 None	N+ve	10	10	10	15
1 Clonic movement	N+ve	0	0	0	0
2 Clonic tremors	N+ve	0	0	0	0
3 Severe clonus	N+ve	0	0	0	0
4 Whole body convulsions	N+ve	0	0	0	0

Day 27 represents data collected from Day 27 to 32.

Table 6

Summary of Neurobehavioral Evaluation: Excitability

32025165
Day: 27 Relative to Start Date

Sex: Male		0 vg<br><br>Group 1	1.49x10^11 vg<br><br>Group 2	4.47x10^11 vg<br><br>Group 3	1.49x10^12 vg<br><br>Group 4
Vocalizations	N	10	10	10	15
0 Once/not at all	N+ve	10	10	10	15
1 More than once	N+ve	0	0	0	0
Startle Response	N	10	10	10	15
-1 No reaction	N+ve	0	0	0	1
0 Normal reaction	N+ve	10	10	10	14
1 Exaggerated Reaction	N+ve	0	0	0	0
Ease of Removal	N	10	10	10	15
-1 Very easy	N+ve	0	0	0	0
0 Easy	N+ve	10	10	10	15
1 Moderate	N+ve	0	0	0	0
2 Difficult	N+ve	0	0	0	0
Handling Reactivity	N	10	10	10	15
-1 Very low	N+ve	0	0	0	0
0 Low	N+ve	10	10	10	14
1 Moderate	N+ve	0	0	0	1
2 High	N+ve	0	0	0	0

Day 27 represents data collected from Day 27 to 32.

Table 6

Summary of Neurobehavioral Evaluation: Excitability

32025165
Day: 27 Relative to Start Date

Sex: Male		0 vg<br>Group 1	1.49x10^11 vg<br>Group 2	4.47x10^11 vg<br>Group 3	1.49x10^12 vg<br>Group 4
Convulsions (Home Cage)	N	10	10	10	15
0 None	N+ve	10	10	10	15
1 Clonic movement	N+ve	0	0	0	0
2 Clonic tremors	N+ve	0	0	0	0
3 Severe clonus	N+ve	0	0	0	0
4 Whole body convulsions	N+ve	0	0	0	0

Day 27 represents data collected from Day 27 to 32.

Table 6

Summary of Neurobehavioral Evaluation: Excitability

32025165
Day: 27 Relative to Start Date

Sex: Female		0 vg<br><br>Group 1	1.49x10^11 vg<br><br>Group 2	4.47x10^11 vg<br><br>Group 3	1.49x10^12 vg<br><br>Group 4
Vocalizations	N	10	10	10	15
0 Once/not at all	N+ve	10	10	10	15
1 More than once	N+ve	0	0	0	0
Startle Response	N	10	10	10	15
-1 No reaction	N+ve	0	0	0	0
0 Normal reaction	N+ve	10	10	10	15
1 Exaggerated Reaction	N+ve	0	0	0	0
Ease of Removal	N	10	10	10	15
-1 Very easy	N+ve	0	0	0	0
0 Easy	N+ve	10	10	10	15
1 Moderate	N+ve	0	0	0	0
2 Difficult	N+ve	0	0	0	0
Handling Reactivity	N	10	10	10	15
-1 Very low	N+ve	0	0	0	0
0 Low	N+ve	10	10	10	15
1 Moderate	N+ve	0	0	0	0
2 High	N+ve	0	0	0	0

Day 27 represents data collected from Day 27 to 32.

Table 6

Summary of Neurobehavioral Evaluation: Excitability

32025165
Day: 27 Relative to Start Date

Sex: Female		0 vg<br><br>Group 1	1.49x10^11 vg<br><br>Group 2	4.47x10^11 vg<br><br>Group 3	1.49x10^12 vg<br><br>Group 4
Convulsions (Home Cage)	N	10	10	10	15
0 None	N+ve	10	10	10	15
1 Clonic movement	N+ve	0	0	0	0
2 Clonic tremors	N+ve	0	0	0	0
3 Severe clonus	N+ve	0	0	0	0
4 Whole body convulsions	N+ve	0	0	0	0

Day 27 represents data collected from Day 27 to 32.

Table 6

Summary of Neurobehavioral Evaluation: Excitability

32025165
Day: 88 Relative to Start Date

Sex: Male		0 vg<br>Group 1	1.49x10^11 vg<br>Group 2	4.47x10^11 vg<br>Group 3	1.49x10^12 vg<br>Group 4
Vocalizations	N	5	10	10	10
0 Once/not at all	N+ve	5	10	10	10
1 More than once	N+ve	0	0	0	0
Startle Response	N	5	10	10	10
-1 No reaction	N+ve	0	0	0	0
0 Normal reaction	N+ve	5	10	10	9
1 Exaggerated Reaction	N+ve	0	0	0	1
Ease of Removal	N	5	10	10	10
-1 Very easy	N+ve	0	0	1	0
0 Easy	N+ve	5	10	9	9
1 Moderate	N+ve	0	0	0	1
2 Difficult	N+ve	0	0	0	0
Handling Reactivity	N	5	10	10	10
-1 Very low	N+ve	0	0	1	0
0 Low	N+ve	5	10	9	9
1 Moderate	N+ve	0	0	0	1
2 High	N+ve	0	0	0	0

Day 27 represents data collected from Day 27 to 32.

Table 6

Summary of Neurobehavioral Evaluation: Excitability

32025165
Day: 88 Relative to Start Date

Sex: Male		0 vg<br><br>Group 1	1.49x10^11 vg<br><br>Group 2	4.47x10^11 vg<br><br>Group 3	1.49x10^12 vg<br><br>Group 4
Convulsions (Home Cage)	N	5	10	10	10
0 None	N+ve	5	10	10	10
1 Clonic movement	N+ve	0	0	0	0
2 Clonic tremors	N+ve	0	0	0	0
3 Severe clonus	N+ve	0	0	0	0
4 Whole body convulsions	N+ve	0	0	0	0

Day 27 represents data collected from Day 27 to 32.

Table 6

Summary of Neurobehavioral Evaluation: Excitability

32025165
Day: 89 Relative to Start Date

Sex: Female		0 vg<br>Group 1	1.49x10^11 vg<br>Group 2	4.47x10^11 vg<br>Group 3	1.49x10^12 vg<br>Group 4
Vocalizations	N	5	10	10	10
0 Once/not at all	N+ve	5	10	10	10
1 More than once	N+ve	0	0	0	0
Startle Response	N	5	10	10	10
-1 No reaction	N+ve	0	1	0	0
0 Normal reaction	N+ve	5	9	10	10
1 Exaggerated Reaction	N+ve	0	0	0	0
Ease of Removal	N	5	10	10	10
-1 Very easy	N+ve	0	0	0	0
0 Easy	N+ve	5	10	10	10
1 Moderate	N+ve	0	0	0	0
2 Difficult	N+ve	0	0	0	0
Handling Reactivity	N	5	10	10	10
-1 Very low	N+ve	0	0	0	0
0 Low	N+ve	5	9	10	9
1 Moderate	N+ve	0	1	0	1
2 High	N+ve	0	0	0	0

Day 27 represents data collected from Day 27 to 32.

Table 6

Summary of Neurobehavioral Evaluation: Excitability

32025165
Day: 89 Relative to Start Date

Sex: Female		0 vg<br>Group 1	1.49x10^11 vg<br>Group 2	4.47x10^11 vg<br>Group 3	1.49x10^12 vg<br>Group 4
Convulsions (Home Cage)	N	5	10	10	10
0 None	N+ve	5	10	10	10
1 Clonic movement	N+ve	0	0	0	0
2 Clonic tremors	N+ve	0	0	0	0
3 Severe clonus	N+ve	0	0	0	0
4 Whole body convulsions	N+ve	0	0	0	0

Day 27 represents data collected from Day 27 to 32.

Table 7

Summary of Neurobehavioral Evaluation: Neuromuscular

32025165
Day: -1 Relative to Start Date

Sex: Male		0 vg<br>Group 1	1.49x10^11 vg<br>Group 2	4.47x10^11 vg<br>Group 3	1.49x10^12 vg<br>Group 4
Gait/Mobility	N	10	10	10	15
0 Normal	N+ve	10	10	10	15
1 Slightly impaired	N+ve	0	0	0	0
2 Moderately impaired	N+ve	0	0	0	0
3 Severely impaired	N+ve	0	0	0	0
Forelimb Grip Strength Mean (Kg)	Mean	0.5150	0.3200	0.4490	0.3356
	SD	0.1320	0.1626	0.2177	0.1618
	N	10	10	10	15
	%Diff	-	-37.8641	-12.8155	-34.8436
Hindlimb Grip Strength Mean (Kg)	Mean	0.3390	0.2523	0.3180	0.2791
	SD	0.1281	0.1605	0.1626	0.1074
	N	10	10	10	15
	%Diff	-	-25.5654	-6.1947	-17.6663
Air Righting Reflex	N	10	10	10	15
0 Normal	N+ve	10	10	10	15
1 Abnormal	N+ve	0	0	0	0
2 Severely impaired	N+ve	0	0	0	0
Tremor (Home Cage)	N	10	10	10	15
0 None	N+ve	10	10	10	15
1 Slight	N+ve	0	0	0	0

Day 27 represents data collected from Day 27 to 32.

Table 7

Summary of Neurobehavioral Evaluation: Neuromuscular

32025165
Day: -1 Relative to Start Date

Sex: Male		0 vg<br>Group 1	1.49x10^11 vg<br>Group 2	4.47x10^11 vg<br>Group 3	1.49x10^12 vg<br>Group 4
2 Moderate	N+ve	0	0	0	0
3 Severe	N+ve	0	0	0	0
Tremor (Open Field)	N	10	10	10	15
0 None	N+ve	10	10	10	15
1 Slight	N+ve	0	0	0	0
2 Moderate	N+ve	0	0	0	0
3 Severe	N+ve	0	0	0	0
Body Tone	N	10	10	10	15
-1 Decreased	N+ve	0	0	0	0
0 Normal	N+ve	10	10	10	15
1 Increased	N+ve	0	0	0	0
Hindlimb Splay Mean (cm)	Mean	13.57	11.96	11.68	11.30
	SD	2.93	3.65	3.20	1.64
	N	10	9	10	15
	%Diff	-	-11.90	-13.96	-16.73

Day 27 represents data collected from Day 27 to 32.

Table 7

Summary of Neurobehavioral Evaluation: Neuromuscular

32025165
Day: -1 Relative to Start Date

Sex: Female		0 vg<br>Group 1	1.49x10^11 vg<br>Group 2	4.47x10^11 vg<br>Group 3	1.49x10^12 vg<br>Group 4
Gait/Mobility	N	10	10	10	15
0 Normal	N+ve	10	10	10	15
1 Slightly impaired	N+ve	0	0	0	0
2 Moderately impaired	N+ve	0	0	0	0
3 Severely impaired	N+ve	0	0	0	0
Forelimb Grip Strength Mean (Kg)	Mean	0.4893	0.5540	0.5553	0.5229
	SD	0.1238	0.0896	0.1882	0.1477
	N	10	10	10	15
	%Diff	-	13.2153	13.4877	6.8574
Hindlimb Grip Strength Mean (Kg)	Mean	0.2693	0.1977	0.3357	0.2667
	SD	0.1175	0.0590	0.1962	0.1149
	N	10	10	10	15
	%Diff	-	-26.6089	24.6287	-0.9901
Air Righting Reflex	N	10	10	10	15
0 Normal	N+ve	10	10	10	15
1 Abnormal	N+ve	0	0	0	0
2 Severely impaired	N+ve	0	0	0	0
Tremor (Home Cage)	N	10	10	10	15
0 None	N+ve	10	10	10	15
1 Slight	N+ve	0	0	0	0

Day 27 represents data collected from Day 27 to 32.

Table 7

Summary of Neurobehavioral Evaluation: Neuromuscular

32025165
Day: -1 Relative to Start Date

Sex: Female		0 vg<br><br>Group 1	1.49x10^11 vg<br><br>Group 2	4.47x10^11 vg<br><br>Group 3	1.49x10^12 vg<br><br>Group 4
2 Moderate	N+ve	0	0	0	0
3 Severe	N+ve	0	0	0	0
Tremor (Open Field)	N	10	10	10	15
0 None	N+ve	10	10	10	15
1 Slight	N+ve	0	0	0	0
2 Moderate	N+ve	0	0	0	0
3 Severe	N+ve	0	0	0	0
Body Tone	N	10	10	10	15
-1 Decreased	N+ve	0	0	0	0
0 Normal	N+ve	9	10	10	15
1 Increased	N+ve	1	0	0	0
Hindlimb Splay Mean (cm)	Mean	10.30	10.11	9.37	10.11
	SD	3.04	1.92	1.93	2.34
	N	10	10	10	15
	%Diff	-	-1.89	-9.03	-1.84

Day 27 represents data collected from Day 27 to 32.

Table 7

Summary of Neurobehavioral Evaluation: Neuromuscular

32025165
Day: 27 Relative to Start Date

Sex: Male		0 vg<br><br>Group 1	1.49x10^11 vg<br><br>Group 2	4.47x10^11 vg<br><br>Group 3	1.49x10^12 vg<br><br>Group 4
Gait/Mobility	N	10	10	10	15
0 Normal	N+ve	10	10	10	15
1 Slightly impaired	N+ve	0	0	0	0
2 Moderately impaired	N+ve	0	0	0	0
3 Severely impaired	N+ve	0	0	0	0
Forelimb Grip Strength Mean (Kg)	Mean<br>SD<br>N<br>%Diff	0.4563<br>0.1423<br>10<br>-	0.4493<br>0.2263<br>10<br>-1.5340	0.4717<br>0.2203<br>10<br>3.3601	0.4393<br>0.1869<br>15<br>-3.7253
Hindlimb Grip Strength Mean (Kg)	Mean<br>SD<br>N<br>%Diff	0.2583<br>0.1259<br>10<br>-	0.1813<br>0.0490<br>10<br>-29.8065	0.2037<br>0.0493<br>10<br>-21.1613	0.2020<br>0.1333<br>15<br>-21.8065
Air Righting Reflex	N	10	10	10	15
0 Normal	N+ve	10	10	10	15
1 Abnormal	N+ve	0	0	0	0
2 Severely impaired	N+ve	0	0	0	0
Tremor (Home Cage)	N	10	10	10	15
0 None	N+ve	10	10	10	15
1 Slight	N+ve	0	0	0	0

Day 27 represents data collected from Day 27 to 32.

Table 7

Summary of Neurobehavioral Evaluation: Neuromuscular

32025165
Day: 27 Relative to Start Date

Sex: Male		0 vg<br>Group 1	1.49x10^11 vg<br>Group 2	4.47x10^11 vg<br>Group 3	1.49x10^12 vg<br>Group 4
2 Moderate	N+ve	0	0	0	0
3 Severe	N+ve	0	0	0	0
Tremor (Open Field)	N	10	10	10	15
0 None	N+ve	9	10	10	15
1 Slight	N+ve	0	0	0	0
2 Moderate	N+ve	1	0	0	0
3 Severe	N+ve	0	0	0	0
Body Tone	N	10	10	10	15
-1 Decreased	N+ve	0	0	0	1
0 Normal	N+ve	10	9	9	14
1 Increased	N+ve	0	1	1	0
Hindlimb Splay Mean (cm)	Mean	14.49	12.18	11.57	11.97
	SD	2.76	2.54	2.15	2.61
	N	10	10	10	15
	%Diff	-	-15.91	-20.16	-17.39

Day 27 represents data collected from Day 27 to 32.

Table 7

Summary of Neurobehavioral Evaluation: Neuromuscular

32025165
Day: 27 Relative to Start Date

Sex: Female		0 vg<br><br>Group 1	1.49x10^11 vg<br><br>Group 2	4.47x10^11 vg<br><br>Group 3	1.49x10^12 vg<br><br>Group 4
Gait/Mobility	N	10	10	10	15
0 Normal	N+ve	10	10	10	15
1 Slightly impaired	N+ve	0	0	0	0
2 Moderately impaired	N+ve	0	0	0	0
3 Severely impaired	N+ve	0	0	0	0
Forelimb Grip Strength Mean (Kg)	Mean<br>SD<br>N<br>%Diff	0.4813<br>0.1237<br>10<br>-	0.5530<br>0.1576<br>10<br>14.8892	0.4867<br>0.1591<br>10<br>1.1080	0.5353<br>0.2190<br>15<br>11.2188
Hindlimb Grip Strength Mean (Kg)	Mean<br>SD<br>N<br>%Diff	0.3757<br>0.1252<br>10<br>-	0.2833<br>0.1374<br>10<br>-24.5785	0.3703<br>0.1401<br>10<br>-1.4197	0.2716<br>0.1295<br>15<br>-27.7137
Air Righting Reflex	N	10	10	10	15
0 Normal	N+ve	10	10	10	15
1 Abnormal	N+ve	0	0	0	0
2 Severely impaired	N+ve	0	0	0	0
Tremor (Home Cage)	N	10	10	10	15
0 None	N+ve	10	10	10	15
1 Slight	N+ve	0	0	0	0

Day 27 represents data collected from Day 27 to 32.

Table 7

Summary of Neurobehavioral Evaluation: Neuromuscular

32025165
Day: 27 Relative to Start Date

Sex: Female		0 vg<br><br>Group 1	1.49x10^11 vg<br><br>Group 2	4.47x10^11 vg<br><br>Group 3	1.49x10^12 vg<br><br>Group 4
2 Moderate	N+ve	0	0	0	0
3 Severe	N+ve	0	0	0	0
Tremor (Open Field)	N	10	10	10	15
0 None	N+ve	10	10	10	15
1 Slight	N+ve	0	0	0	0
2 Moderate	N+ve	0	0	0	0
3 Severe	N+ve	0	0	0	0
Body Tone	N	10	10	10	15
-1 Decreased	N+ve	0	0	0	0
0 Normal	N+ve	10	10	10	15
1 Increased	N+ve	0	0	0	0
Hindlimb Splay Mean (cm)	Mean	10.30	10.34	10.28	10.07
	SD	1.30	2.08	1.66	2.44
	N	10	10	10	15
	%Diff	-	0.34	-0.19	-2.27

Day 27 represents data collected from Day 27 to 32.

Table 7

Summary of Neurobehavioral Evaluation: Neuromuscular

32025165
Day: 88 Relative to Start Date

Sex: Male		0 vg<br>Group 1	1.49x10^11 vg<br>Group 2	4.47x10^11 vg<br>Group 3	1.49x10^12 vg<br>Group 4
Gait/Mobility	N	5	10	10	10
0 Normal	N+ve	5	10	10	10
1 Slightly impaired	N+ve	0	0	0	0
2 Moderately impaired	N+ve	0	0	0	0
3 Severely impaired	N+ve	0	0	0	0
Forelimb Grip Strength Mean (Kg)	Mean	0.7673	0.6977	0.5603	0.5963
	SD	0.2089	0.0853	0.2110	0.2265
	N	5	10	10	10
	%Diff	-	-9.0791	-26.9765	-22.2850
Hindlimb Grip Strength Mean (Kg)	Mean	0.2820	0.2483	0.3543	0.3357
	SD	0.1063	0.0377	0.1019	0.1184
	N	5	10	10	10
	%Diff	-	-11.9385	25.6501	19.0307
Air Righting Reflex	N	5	10	10	10
0 Normal	N+ve	5	10	10	10
1 Abnormal	N+ve	0	0	0	0
2 Severely impaired	N+ve	0	0	0	0
Tremor (Home Cage)	N	5	10	10	10
0 None	N+ve	5	10	10	10
1 Slight	N+ve	0	0	0	0

Day 27 represents data collected from Day 27 to 32.

Table 7

Summary of Neurobehavioral Evaluation: Neuromuscular

32025165
Day: 88 Relative to Start Date

Sex: Male		0 vg<br><br>Group 1	1.49x10^11 vg<br><br>Group 2	4.47x10^11 vg<br><br>Group 3	1.49x10^12 vg<br><br>Group 4
2 Moderate	N+ve	0	0	0	0
3 Severe	N+ve	0	0	0	0
Tremor (Open Field)	N	5	10	10	10
0 None	N+ve	5	10	10	10
1 Slight	N+ve	0	0	0	0
2 Moderate	N+ve	0	0	0	0
3 Severe	N+ve	0	0	0	0
Body Tone	N	5	10	10	10
-1 Decreased	N+ve	0	0	0	1
0 Normal	N+ve	5	10	10	9
1 Increased	N+ve	0	0	0	0
Hindlimb Splay Mean (cm)	Mean<br>SD<br>N<br>%Diff	14.48<br>1.51<br>5<br>-	13.02<br>1.55<br>10<br>-10.12	14.07<br>1.86<br>10<br>-2.87	15.29<br>2.12<br>10<br>5.59

Day 27 represents data collected from Day 27 to 32.

Table 7

Summary of Neurobehavioral Evaluation: Neuromuscular

32025165
Day: 89 Relative to Start Date

Sex: Female		0 vg<br>Group 1	1.49x10^11 vg<br>Group 2	4.47x10^11 vg<br>Group 3	1.49x10^12 vg<br>Group 4
Gait/Mobility	N	5	10	10	10
0 Normal	N+ve	5	10	10	10
1 Slightly impaired	N+ve	0	0	0	0
2 Moderately impaired	N+ve	0	0	0	0
3 Severely impaired	N+ve	0	0	0	0
Forelimb Grip Strength Mean (Kg)	Mean<br>SD<br>N<br>%Diff	0.2873<br>0.0483<br>5<br>-	0.3350<br>0.1173<br>10<br>16.5893	0.4013<br>0.1808<br>10<br>39.6752	0.3810<br>0.2310<br>10<br>32.5986
Hindlimb Grip Strength Mean (Kg)	Mean<br>SD<br>N<br>%Diff	0.3580<br>0.1337<br>5<br>-	0.2723<br>0.1066<br>10<br>-23.9292	0.2055<br>0.1002<br>10<br>-42.5978	0.2217<br>0.1004<br>10<br>-38.0819
Air Righting Reflex	N	5	10	10	10
0 Normal	N+ve	5	10	10	10
1 Abnormal	N+ve	0	0	0	0
2 Severely impaired	N+ve	0	0	0	0
Tremor (Home Cage)	N	5	10	10	10
0 None	N+ve	5	10	10	10
1 Slight	N+ve	0	0	0	0

Day 27 represents data collected from Day 27 to 32.

Table 7

Summary of Neurobehavioral Evaluation: Neuromuscular

32025165
Day: 89 Relative to Start Date

Sex: Female		0 vg<br>Group 1	1.49x10^11 vg<br>Group 2	4.47x10^11 vg<br>Group 3	1.49x10^12 vg<br>Group 4
2 Moderate	N+ve	0	0	0	0
3 Severe	N+ve	0	0	0	0
Tremor (Open Field)	N	5	10	10	10
0 None	N+ve	5	10	10	10
1 Slight	N+ve	0	0	0	0
2 Moderate	N+ve	0	0	0	0
3 Severe	N+ve	0	0	0	0
Body Tone	N	5	10	10	10
-1 Decreased	N+ve	0	0	0	0
0 Normal	N+ve	5	10	10	10
1 Increased	N+ve	0	0	0	0
Hindlimb Splay Mean (cm)	Mean	11.55	10.96	10.30	9.60
	SD	0.88	2.05	1.59	2.87
	N	5	10	10	10
	%Diff	-	-5.15	-10.87	-16.88

Day 27 represents data collected from Day 27 to 32.

Table 8

Summary of Neurobehavioral Evaluation: Physiological

32025165
Day: -1 Relative to Start Date

Sex: Male		0 vg<br><br>Group 1	1.49x10^11 vg<br><br>Group 2	4.47x10^11 vg<br><br>Group 3	1.49x10^12 vg<br><br>Group 4
Respiration	N	10	10	10	15
-1 Slowed	N+ve	0	0	0	0
0 Normal	N+ve	10	10	10	15
1 Rapid	N+ve	0	0	0	0
2 Abnormal	N+ve	0	0	0	0
Body Temperature (oC)	Mean	36.51	36.83	36.65	35.91
	SD	0.35	0.90	0.57	1.22
	N	10	10	10	15
	%Diff	-	0.88	0.38	-1.63

Day 27 represents data collected from Day 27 to 32.

Table 8

Summary of Neurobehavioral Evaluation: Physiological

32025165
Day: -1 Relative to Start Date

Sex: Female		0 vg<br>Group 1	1.49x10^11 vg<br>Group 2	4.47x10^11 vg<br>Group 3	1.49x10^12 vg<br>Group 4
Respiration	N	10	10	10	15
-1 Slowed	N+ve	0	0	0	0
0 Normal	N+ve	10	10	10	15
1 Rapid	N+ve	0	0	0	0
2 Abnormal	N+ve	0	0	0	0
Body Temperature (oC)	Mean	37.03	37.47	36.88	35.81
	SD	0.89	0.83	0.82	2.11
	N	10	10	10	15
	%Diff	-	1.19	-0.41	-3.30

Day 27 represents data collected from Day 27 to 32.

Table 8

Summary of Neurobehavioral Evaluation: Physiological

32025165
Day: 27 Relative to Start Date

Sex: Male		0 vg<br>Group 1	1.49x10^11 vg<br>Group 2	4.47x10^11 vg<br>Group 3	1.49x10^12 vg<br>Group 4
Respiration	N	10	10	10	15
-1 Slowed	N+ve	0	0	0	0
0 Normal	N+ve	10	10	10	15
1 Rapid	N+ve	0	0	0	0
2 Abnormal	N+ve	0	0	0	0
Body Temperature (oC)	Mean	36.45	37.10	36.67	36.31
	SD	0.80	0.63	0.41	0.96
	N	10	10	10	15
	%Diff	-	1.78	0.60	-0.37

Day 27 represents data collected from Day 27 to 32.

Table 8

Summary of Neurobehavioral Evaluation: Physiological

32025165
Day: 27 Relative to Start Date

Sex: Female		0 vg<br>Group 1	1.49x10^11 vg<br>Group 2	4.47x10^11 vg<br>Group 3	1.49x10^12 vg<br>Group 4
Respiration	N	10	10	10	15
-1 Slowed	N+ve	0	0	0	0
0 Normal	N+ve	10	9	10	15
1 Rapid	N+ve	0	1	0	0
2 Abnormal	N+ve	0	0	0	0
Body Temperature (oC)	Mean	37.54	37.31	37.54	37.00
	SD	0.99	0.54	0.44	1.32
	N	10	10	10	15
	%Diff	-	-0.61	0.00	-1.44

Day 27 represents data collected from Day 27 to 32.

Table 8

Summary of Neurobehavioral Evaluation: Physiological

32025165
Day: 88 Relative to Start Date

Sex: Male		0 vg<br><br>Group 1	1.49x10^11 vg<br><br>Group 2	4.47x10^11 vg<br><br>Group 3	1.49x10^12 vg<br><br>Group 4
Respiration	N	5	10	10	10
-1 Slowed	N+ve	0	0	0	0
0 Normal	N+ve	5	10	10	10
1 Rapid	N+ve	0	0	0	0
2 Abnormal	N+ve	0	0	0	0
Body Temperature (oC)	Mean	37.12	36.63	36.25	36.51
	SD	0.38	0.24	0.25	0.75
	N	5	10	10	10
	%Diff	-	-1.32	-2.34	-1.64

Day 27 represents data collected from Day 27 to 32.

Table 8

Summary of Neurobehavioral Evaluation: Physiological

32025165
Day: 89 Relative to Start Date

Sex: Female		0 vg<br><br>Group 1	1.49x10^11 vg<br><br>Group 2	4.47x10^11 vg<br><br>Group 3	1.49x10^12 vg<br><br>Group 4
Respiration	N	5	10	10	10
-1 Slowed	N+ve	0	0	0	0
0 Normal	N+ve	5	10	10	10
1 Rapid	N+ve	0	0	0	0
2 Abnormal	N+ve	0	0	0	0
Body Temperature (oC)	Mean	37.56	37.71	37.55	37.99
	SD	0.46	0.58	0.54	0.67
	N	5	10	10	10
	%Diff	-	0.40	-0.03	1.14

Day 27 represents data collected from Day 27 to 32.

Table 9

Summary of Neurobehavioral Evaluation: Sensorimotor

32025165
Day: -1 Relative to Start Date

Sex: Male		0 vg<br>Group 1	1.49x10^11 vg<br>Group 2	4.47x10^11 vg<br>Group 3	1.49x10^12 vg<br>Group 4
Touch Resp/Tactile Reflex	N	10	10	10	15
-1 No reaction	N+ve	0	0	0	0
0 Normal reaction	N+ve	10	10	10	15
1 Exaggerated reaction	N+ve	0	0	0	0
Thermal Response (sec)	Mean	11.962	9.163	7.762	10.645
	SD	4.368	4.109	2.409	5.343
	N	10	10	10	15
	%Diff	-	-23.399	-35.111	-11.013

Day 27 represents data collected from Day 27 to 32.

Table 9

Summary of Neurobehavioral Evaluation: Sensorimotor

32025165
Day: -1 Relative to Start Date

Sex: Female		0 vg<br><br>Group 1	1.49x10^11 vg<br><br>Group 2	4.47x10^11 vg<br><br>Group 3	1.49x10^12 vg<br><br>Group 4
Touch Resp/Tactile Reflex	N	10	10	10	15
-1 No reaction	N+ve	0	0	0	0
0 Normal reaction	N+ve	10	10	10	15
1 Exaggerated reaction	N+ve	0	0	0	0
Thermal Response (sec)	Mean	9.596	6.676	6.374	6.363
	SD	7.235	2.825	1.416	1.421
	N	10	10	10	15
	%Diff	-	-30.429	-33.576	-33.688

Day 27 represents data collected from Day 27 to 32.

Table 9

Summary of Neurobehavioral Evaluation: Sensorimotor

32025165
Day: 27 Relative to Start Date

Sex: Male		0 vg<br><br>Group 1	1.49x10^11 vg<br><br>Group 2	4.47x10^11 vg<br><br>Group 3	1.49x10^12 vg<br><br>Group 4
Touch Resp/Tactile Reflex	N	10	10	10	15
-1 No reaction	N+ve	0	0	1	3
0 Normal reaction	N+ve	10	10	9	12
1 Exaggerated reaction	N+ve	0	0	0	0
Thermal Response (sec)	Mean	8.362	7.505	8.308	9.124
	SD	2.385	2.006	3.174	3.051
	N	10	10	10	15
	%Diff	-	-10.249	-0.646	9.113

Day 27 represents data collected from Day 27 to 32.

Table 9

Summary of Neurobehavioral Evaluation: Sensorimotor

32025165
Day: 27 Relative to Start Date

Sex: Female		0 vg<br><br>Group 1	1.49x10^11 vg<br><br>Group 2	4.47x10^11 vg<br><br>Group 3	1.49x10^12 vg<br><br>Group 4
Touch Resp/Tactile Reflex	N	10	10	10	15
-1 No reaction	N+ve	0	0	0	0
0 Normal reaction	N+ve	10	10	10	15
1 Exaggerated reaction	N+ve	0	0	0	0
Thermal Response (sec)	Mean	9.968	10.360	9.226	8.291
	SD	7.046	4.693	3.668	3.657
	N	10	10	10	15
	%Diff	-	3.933	-7.444	-16.827

Day 27 represents data collected from Day 27 to 32.

Table 9

Summary of Neurobehavioral Evaluation: Sensorimotor

32025165
Day: 88 Relative to Start Date

Sex: Male		0 vg<br><br>Group 1	1.49x10^11 vg<br><br>Group 2	4.47x10^11 vg<br><br>Group 3	1.49x10^12 vg<br><br>Group 4
Touch Resp/Tactile Reflex	N	5	10	10	10
-1 No reaction	N+ve	0	0	0	0
0 Normal reaction	N+ve	5	10	10	9
1 Exaggerated reaction	N+ve	0	0	0	1
Thermal Response (sec)	Mean	6.346	8.458	8.596	7.935
	SD	1.782	2.540	2.995	4.053
	N	5	10	10	10
	%Diff	-	33.281	35.455	25.039

Day 27 represents data collected from Day 27 to 32.

Table 9

Summary of Neurobehavioral Evaluation: Sensorimotor

32025165
Day: 89 Relative to Start Date

Sex: Female		0 vg<br><br>Group 1	1.49x10^11 vg<br><br>Group 2	4.47x10^11 vg<br><br>Group 3	1.49x10^12 vg<br><br>Group 4
Touch Resp/Tactile Reflex	N	5	10	10	10
-1 No reaction	N+ve	0	1	0	0
0 Normal reaction	N+ve	5	9	10	10
1 Exaggerated reaction	N+ve	0	0	0	0
Thermal Response (sec)	Mean	8.322	8.427	9.220	11.557
	SD	3.214	3.676	3.138	7.190
	N	5	10	10	10
	%Diff	-	1.262	10.791	38.873

Day 27 represents data collected from Day 27 to 32.

Appendix 1

DEVIATIONS

All deviations that occurred during the study have been authorized/acknowledged by the Study Director, assessed for impact, and documented in the study records. All protocol deviations that could have impacted the quality or integrity of the study are listed below.

None of the deviations were considered to have impacted the overall integrity of the study or the interpretation of the study results and conclusions.

Deviations

**Formulations and Dosing**		
**Protocol Section No.**	**Deviation**	**Impact**
9.1	On Day 1, the syringe type used for dose administration was not documented.	This was a minor documentation error that did not affect dosing nor study data interpretation.
**Husbandry**		
**Protocol Section No.**	**Deviation**	**Impact**
8.5	On Day -2, two males at 4.47 $x10^{11}$ vg (Animal Nos. 3003 and 3004) did not have access to water.	Animal welfare was maintained with no impact to the study data interpretation.
8.5	On Day 41, several animals did not have access to water.	Animal welfare was maintained with no impact to the study data interpretation.
**In-life Observations, Measurements, and Evaluations**		
**Protocol Section No.**	**Deviation**	**Impact**
11	On 1 occasion, an afternoon mortality/cageside observation was not conducted.	Due to the following day morning mortality check being normal, there was no impact on study data interpretation.
11	On Day -1, hindlimb splay data were not recorded for 1 male at 1.49 $x10^{11}$ vg (Animal No. 2002).	There were a significant number of animals remaining in this group such that this error did not affect study data interpretation.
11	Animals designated for the Day 91 necropsy did not have ophthalmic exams performed prior to the Day 29 necropsy.	There were a significant number of animals remaining these groups such that this error did not affect study data interpretation.
**Laboratory Evaluations**		
**Protocol Section No.**	**Deviation**	**Impact**
13.2	On Day 90, documentation was recorded to indicate blood samples were placed on wet ice; however, the sample type placed on wet ice was not recorded. It cannot be determined whether the biodistribution samples were placed on wet ice or the ADA samples were stored ambient following collection.	There were a sufficient number of samples from the other animals in this group and as such, this error did not impact the ability to interpret the data.

Appendix 1

Postmortem and Pathology		
**Protocol Section No.**	**Deviation**	**Impact**
14.6.1	At necropsy, a photograph of the brain was not taken for 1 male at 1.49 $x10^{12}$ vg (Animal No. 4003).	There were a significant number of animals such that this error did not affect study data interpretation. Moreover, the histopathology analysis was not affected.
14.7	At necropsy, there was no documentation that the frozen samples for 1 male at 1.49 $x10^{11}$ vg (Animal No. 2010) were placed into pre-labeled polypropylene vials, tightly capped, and frozen on liquid nitrogen or that care was taken to minimize the time between sample collection and freezing.	Subsequent study data were available to demonstrate that the study objectives were satisfactorily achieved as a result of performance of this function. Therefore, this minor documentation error did not negatively impact the Study Director's ability to interpret study data.<br>Data was reviewed appropriately.
15.2	At the time of pathology assessment, the mammary gland for 1 male at 0 vg (Animal No. 1009) and one female at 4.47 $x10^{11}$ vg (Animal No. 3502), and the pancreas for 1 female at 1.49 $x10^{12}$ vg (Animal No. 4509) were not microscopically examined.	This had no impact on the pathologist or SD interpretation of the results as there was a sufficient n in each of these groups.

Appendix 1

Protocol Amendment No. 04

Testing Facility Study No. 32025165

Sponsor Reference No. 32025165

A 13 Week IT Toxicity Study in Rats with 4-Week and 13-Week Biodistribution

GLP

SPONSOR:
University of Massachusetts
55 N Lake Avenue
Worcester, MA 01605-2324
United States

TESTING FACILITY:
Charles River Laboratories
54943 North Main Street
Mattawan, MI 49071
United States

DRAFT, NOT FOR SUBMISSION

Appendix 1

SUMMARY OF CHANGES AND JUSTIFICATIONS

Protocol Effective Date: 11 Jul 2025

Note: When applicable additions are indicated in **bold underlined** text and deletions indicated in ~~**bold strikethrough**~~ text in the affected sections of the document.

Item or Section(s)	Justification
**Amendment 1**	**Effective Date of Change: 08 Aug 2025**
Title Page	Corrected spelling error.
4. Responsible Personnel	Dose formulation analysis removed, anatomic pathologist and bioanalytical scientist details included.
12. Clinical Pathology<br>12.5. Urinalysis Evaluation	Urinalysis removed, hematology volume reduced per Sysmex requirements. Blood collection priority included.
13. ADA and Biodistribution EVALUATION<br>13.2. Biodistribution Sample Processing<br>13.4 Biodistribution Sample Analysis<br>13.5 ADA Sample Collection<br>13.6. ADA Sample Processing<br>13.7. ADA Sample Shipment<br>13.8. ADA Sample Analysis	Updated storage conditions for Biodistribution samples. ISR will not occur.<br>ADA sample collection included and ADA samples will be saved for possible future analysis.
14. TERMINAL PROCEDURES	Window required for necropsy day.
14.2 Unscheduled Euthanasia	Updated sample volumes.
14.6.1. Brain Tissue Collection Details	Processing details updated.
14.7. Biodistribution Frozen Tissue Collection	Sample collection details clarified.
15.2. Microscopic Evaluation	PAI will not be utilized as a pathology test site.
18. REGULATORY COMPLIANCE	Additional exceptions included and included language regarding samples for possible future analysis.
ATTACHMENT A	Multiple clarifications for tissue collection and processing.
ATTACHMENT B	Shipment details provided for samples.
**Amendment 2**	**Effective Date of Change: 03 Sep 2025**
4. RESPONSIBLE PERSONNEL	Updated to include Anatomical Pathologist contact information.
ATTACHMENT A	Updated table to include corresponding footnotes.
**Amendment 3**	**Effective Date of Change: 22 Oct 2025**
4. RESPONSIBLE PERSONNEL	Removed Ophthalmologist.

Appendix 1

5.2. Test Material Identification	Updated concentration.
9. EXPERIMENTAL DESIGN	Updated dose level and concentration.
13. ADA AND BIODISTRIBUTION EVALUATION	Window added based on terminal procedures and necropsy interval schedule
13.3. Biodistribution Sample Shipment<br>13.4. Biodistribution Sample Analysis	Updated to indicate the samples will be shipped to the Sponsor and all analytical work will be done by them.
13.7. ADA Sample Shipment	Updated to indicate samples will be shipped to the Sponsor.
14.7.1 Biodistribution Frozen Tissue Analysis<br>Attachment B	Samples will be analyzed by the sponsor. Shipment details included. Clarification of the conditions for shipment.
24.2. Justification of Route and Dose Levels	Updated wording.
ATTACHMENT B	Updated shipping contact and conditions for shipment.
**Amendment 4**	**Effective Date of Change: 29 Oct 2025**
1. Objectives	Corrected spelling error.
14.8. Spleen Sample Collection for PBMC Analysis	Additional samples will be collected from the spleen of a cohort of animals for use in PBMC analysis by the sponsor.

Appendix 1

TABLE OF CONTENTS

SUMMARY OF CHANGES AND JUSTIFICATIONS 2
1. OBJECTIVE(S) 6
2. PROPOSED STUDY SCHEDULE 6
3. SPONSOR 6
4. RESPONSIBLE PERSONNEL 7
5. TEST AND CONTROL MATERIALS 8
6. DOSE FORMULATION AND ANALYSIS 9
7. TEST SYSTEM 10
8. HUSBANDRY 12
9. EXPERIMENTAL DESIGN 13
10. SURGICAL ANIMAL PREPARATION 14
11. IN-LIFE PROCEDURES, OBSERVATIONS, AND MEASUREMENTS 15
12. CLINICAL PATHOLOGY 17
13. ADA AND BIODISTRIBUTION EVALUATION 19
14. TERMINAL PROCEDURES 21
15. HISTOLOGY AND MICROSCOPIC EVALUATION 24
16. STATISTICAL ANALYSIS 26
17. COMPUTERIZED SYSTEMS 26
18. REGULATORY COMPLIANCE 27
19. QUALITY ASSURANCE 28
20. AMENDMENTS AND DEVIATIONS 28
21. RETENTION AND DISPOSITION OF RECORDS, SAMPLES, AND SPECIMENS 28
22. STUDY CLASSIFICATION 29
23. REPORTING 29
24. JUSTIFICATIONS AND GUIDELINES 30
25. ANIMAL WELFARE 31
26. REFERENCES 31
ATTACHMENT A 32
ATTACHMENT B 34

Appendix 1

SPONSOR APPROVAL 35
TESTING FACILITY APPROVAL...36

DRAFT, NOT FOR SUBMISSION

Appendix 1

1. OBJECTIVE(S)

The objective of this study is to determine the potential toxicity and biodistribution of scAAV9-CSA for the treatment of Cockyayne Syndrome, when given as an intrathecal injection to rats once, and to evaluate the potential reversibility of any findings following a 28-day and 90-day recovery period.

2. PROPOSED STUDY SCHEDULE

Proposed study dates are listed below. Actual dates will be included in the Final Report.

Experimental Starting Date:	11 Jul 2025<br>(First date of study-specific data collection)
Experimental Completion Date:	To be included in the Final Report<br>(Last date on which data are collected)
Initiation of Dosing:	18 Jul 2025
Completion of In-life:	29 Oct 2025<br>(Last date of euthanasia)
Interim Report	9 weeks<br>(Following last day of necropsy or last sample analyzed)
Draft Report:	9 weeks<br>(Following last day of necropsy or last sample analyzed)
Final Report:	21 Aug 2026<br>(Expected date of Study Director signature of report)
Final SEND Dataset Package Delivery:	Based on regulatory submission date

3. SPONSOR

Role	Name/Contact Information
Sponsor Monitor	Heather Gray-Edwards DVM, PhD<br>Tel: (508) 856-5737<br>E-mail: Heather.GrayEdwards@umassmed.edu<br>Address as cited for Sponsor
Sponsor Representative	Miguel Sena-Esteves, PhD<br>Tel: (508) 856-4412<br>E-mail: Miguel.Esteves@umassmed.edu<br>Address as cited for Sponsor

DRAFT, NOT FOR SUBMISSION

Appendix 1

4. RESPONSIBLE PERSONNEL

Role/Phase	Quality Assurance Unit	Name/Contact Information
Study Director	Charles River	Barak W. Gunter, PhD<br>Tel: 269-668-3336 ext. 1099<br>E-mail: barak.gunter@crl.com<br>Address as cited for Testing Facility
Testing Facility Management	Charles River	Scott T. Wilson, BS, LAT<br>Tel: 269-668-3336 ext. 1610<br>E-mail: scott.wilson@crl.com<br>Address as cited for Testing Facility
Testing Facility QAU	Charles River	Tel: 269-668-3336<br>E-mail: MWN-QAMgmt@charlesriverlabs.com<br>Address as cited for Testing Facility
SEND	Not Applicable	SEND Data Management<br>E-mail: CRL-SEND-ES@crl.com
**Individual Scientist(s) (IS)**		
Anatomic Pathologist	Charles River	Jeremiah Lyons, MVB, PhD, DACVP<br>Tel: 269-668-3336 ext. 1379<br>E-mail: jerry.lyons@crl.com<br>Address as cited for Testing Facility
Clinical Pathologist	Charles River	Danielle Gordon, DVM, MS, DACVP<br>Tel: 269-668-3336, ext. 1986<br>E-mail: danielle.gordon@crl.com<br>Address as cited for Testing Facility
**Principal Investigator(s) (PI)**		
Biodistribution Analysis[a]	Not Applicable	Miguel Sena-Esteves, PhD<br>Tel: (508) 856-4412<br>E-mail: Miguel.Esteves@umassmed.edu<br>Address as cited for Sponsor

[a] Sponsor-designated Test Site.

Each IS and PI is required to report all deviations or other circumstances that could affect the quality or integrity of the study to the Study Director in a timely manner for authorization/acknowledgement. Each IS and PI will provide a report addressing their assigned phase of the study, which will be included as an appendix to the Final Report.

The IS Phase Report will include the following:

- A listing of critical computerized systems used in the conduct and/or interpretation of the assigned study phase.

The PI Phase Report will include the following:

- A Statement of Compliance (if the applicable phase is GLP)

- A QA Statement (for Sponsor designated PI or for Testing Facility designated PI if audited by a QAU other than that of the Testing Facility and if the applicable phase is GLP)
- The archive site for all records, samples, specimens and reports generated from the phase or segment (alternatively, details regarding the retention of the materials may be provided to the Study Director for inclusion in the Final Report), if applicable.
- A listing of critical computerized systems used in the conduct and/or interpretation of the assigned study phase.

5. TEST AND CONTROL MATERIALS

5.1. Test and Control Material Characterization

The Sponsor will provide to the Testing Facility documentation of the identity, strength, purity, composition, and stability for the test and control article(s). A Certificate of Analysis or equivalent documentation will be provided (for active test material ingredient and preformulated material) for inclusion in the Final Report.

Documentation of identity, strength, purity, composition, and stability will be provided for control material.

The Sponsor has appropriate documentation on file concerning the method of synthesis, fabrication or derivation of the test and control article(s), and this information is available to the appropriate regulatory agencies should it be requested.

5.2. Test Material Identification

Test Material Identification

	**Test Material**
**Identification:**	scAAV9-CSA
**Alternate Identification:**	N/A
**Batch/Lot No.:**	PDTP-000102
**Expiration/Retest Date:**	N/A
**Physical Description:**	Clear, colorless liquid, no visible particulates
**Purity:**	-
**Correction Factor:**	-
**Concentration:**	1.49E+13 vg/mL
**Density:**	Supplied to the Testing Facility<br>N/A
**Storage Conditions:**	Frozen (-60 to -90°C)
**Test Material Contact:**	Heather Gray-Edwards DVM, PhD<br>Tel: (508) 856-5737<br>E-mail: Heather.GrayEdwards@umassmed.edu

- = Not applicable.

Appendix 1

5.3. Vehicle Control Identification

Vehicle Control Identification

	Vehicle Control
**Identification:**	Vehicle Control
**Alternate Identification:**	VVC-321
**Lot #**	TMNP0325-B
**Retest Date:**	-
**Storage Conditions:**	Frozen (-60 to -90°C)

- = Not applicable.

5.4. Reserve Samples

For each batch (lot) of test and vehicle control article(s), a reserve sample will be collected and maintained under the appropriate storage conditions by the Testing Facility.

5.5. Test and Vehicle Control Material Inventory and Disposition

Records of the receipt, distribution, storage, and disposition of test article(s) (including empty containers of Sponsor-provided materials) will be maintained. All unused Sponsor supplied bulk test article(s), with the exception of reserve samples, will be returned to the Sponsor following issuance of the Draft Report unless otherwise requested (documentation will be retained in the study record). An earlier shipment of these materials may also be requested and authorized by the Study Director and Sponsor. See shipment of samples and study documentation (Attachment B) for shipping details.

The Sponsor will be contacted for proper disposition of materials (retain/ship/discard) after completion of the in-life phase of the study and following confirmation that these materials are not assigned to other studies.

5.6. Safety

A Safety Data Sheet (SDS), or equivalent documentation, will be provided by the Sponsor (if available). It is the responsibility of the Sponsor to notify the Testing Facility of any special handling requirements of the test article(s). Otherwise, routine safety precautions will be followed. Appropriate gloves, safety glasses and arm covers will be worn by individuals working with neat test article(s) or formulations.

6. DOSE FORMULATION AND ANALYSIS

6.1. Preparation of Formulations

Dose formulations will be divided into aliquots where required to allow to be dispensed on each dosing occasion.

Appendix 1

Preparation Details

Component	Preparation	Frequency	Storage Conditions
Dose Formulations/Test Material	Diluted in the vehicle	Once	Refrigerated (2 to 8°C)

Test article mixed with vehicle to achieve desired concentrations.

Any partial vials of stock will be stored Refrigerated (2 to 8°C) following thawing and preparation. Any residual dose formulations from each dosing occasion will be stored Refrigerated (2 to 8°C) until shipped unless otherwise requested by the Study Director. Any unused, unopened vials will remain Frozen (-60 to -90°C).

6.2. Sample Collection and Analysis

Dose formulation samples (2, 0.1 ml samples) from each Group 2 and Group 3 preparation will be collected and stored Refrigerated (2 to 8°C) for possible future analysis. Additional samples may be collected and analyzed at the discretion of the Study Director.

7. TEST SYSTEM

Species/Species Family:	Rat
Strain/Breed:	Sprague Dawley®
Substrain:	Crl:CD(SD)
Animal Condition:	Naïve
Animal Supplier:	Charles River Laboratories
Animal Supplier Location:	Raleigh North Carolina, United States
Number Ordered:	98
Number of Males:	45 on study (4 alternates)
Number of Females:	45 on study (4 alternates)
Target Age at Initiation of Dosing:	8 to 10 weeks
Target Weight at the Initiation of Dosing:	120 to 370 g for males, 110 to 310 g for females
Age at Arrival:	Ordered to be 7 weeks of age at arrival

DRAFT, NOT FOR SUBMISSION

Appendix 1

Weight at Arrival	Males will be ordered to weigh 130 to 210 g and females will be ordered to weigh 100 to 170 g at the time of surgery, as measured within 4 days of the procedure. The actual range may vary but will be documented in the data.

The actual age and weight of animals received will be listed in the Final Report.

7.1. Animal Identification

Method:	Each animal will be assigned an animal number to be used in Provantis™ and will be implanted with a microchip bearing a unique identification number. The individual animal number, implant number, and the Testing Facility study number will comprise a unique identification for each animal. The animal cage will be identified by the study number, animal number, group number, and sex.

7.2. Environmental Acclimation

Acclimation Duration:	At least 1 week
Details:	During this acclimation period, all animals will be observed daily for any clinical signs of disease, and all animals will be given a detailed clinical examination within 3 days of the first dose administration.

7.3. Selection, Assignment, Replacement and Disposition of Animals

Assignment and Randomization:	Animals will be randomly assigned to groups upon receipt. Males and females will be randomized separately.
	All animals with any evidence of disease or physical abnormalities will not be selected for study. Animals considered suitable for study will be randomly selected and divided into groups as described above.
Replacement:	Before the initiation of dosing, any assigned animals considered unsuitable for use in the study will be replaced by alternate animals maintained under the same environmental conditions.
	After initiation of dosing, study animals may be replaced during the replacement period with alternate animals in the event of accidental injury, non-test article-related health issues, or similar circumstances. Any data generated will not be included in the report unless deemed appropriate by the Study Director.
	Alternate animals may be used as replacements per Testing Facility SOP.
Disposition:	Extra animals obtained for this study, but not placed on study, will be either transferred to a Testing Facility stock or training colony.

DRAFT, NOT FOR SUBMISSION

The disposition of all animals will be documented in the study records.

8. HUSBANDRY

8.1. Housing

Housing:	Socially housed, when possible (animals may be housed 2 to 3 per cage during acclimation and in-life depending on study design).<br>Animals will be separated during designated procedures/activities or will be separated as required for monitoring and/or health purposes, as deemed appropriate by Study Director and/or Clinical Veterinarian.<br>Housing set-up is as specified in the USDA Animal Welfare Act (9 CFR, Parts 1, 2 and 3) and as described in the Guide for the Care and Use of Laboratory Animals (NRC, current edition). Animals will be separated during designated procedures/activities or will be separated as required for monitoring and/or health purposes, as deemed appropriate by Study Director and/or Clinical Veterinarian. In addition, animals may be individually housed due to incompatible behavior with a cage mate(s).
Caging:	Solid bottom cages with nonaromatic bedding. The bedding will be from an approved supplier.

8.2. Animal Enrichment

Supplemental Enrichment:	Animal enrichment will be provided as per site standard procedures.

8.3. Environmental Conditions

Temperature and Humidity:	Temperature and humidity will be maintained according to Testing Facility SOP.
Lighting:	Fluorescent or equivalent lighting (e.g., LED) will be provided via an automatic timer for approximately 12 hours per day. On occasion, the light/dark cycle may be interrupted intermittently due to study-related activities.

8.4. Food

Diet:	The basal diet will be block Lab Diet® Certified Rodent Diet #5CR4, PMI Nutrition International, Inc.
Frequency:	Ad libitum, except during designated procedures

Analysis: Results of analysis for nutritional components and environmental contaminants are on file at the Testing Facility.

There are no known contaminants in the food that would interfere with this study.

8.5. Water

Type: Tap water, treated and suitable for consumption.

Frequency: Ad libitum via an automatic watering system (except during designated procedures). Water bottles can be provided, if required.

Analysis: Periodic analysis of the water is performed per Testing Facility SOP and results of these analyses are on file at the Testing Facility. It is considered that there are no known contaminants in the water that would interfere with the outcome of the study.

8.6. Veterinary Care

Veterinary care will be available throughout the course of the study and animals will be examined by the veterinary staff as warranted by clinical signs or other changes. In the event that animals show signs of illness or distress, the responsible veterinarian may make initial recommendations about treatment of the animal(s) and/or alteration of study procedures, which must be approved by the Study Director. If the condition of the animal(s) warrants therapeutic intervention or alterations in study procedures, the Sponsor will be contacted, when possible, to discuss appropriate action(s). If the condition of the animal(s) is such that emergency measures must be taken, the Study Director and/or attending veterinarian will attempt to consult with the Sponsor prior to responding to the medical crisis, but the Study Director and/or veterinarian has authority to act immediately at his/her discretion to alleviate suffering. The Sponsor will be fully informed of any such events.

9. EXPERIMENTAL DESIGN

Experimental Design

Group No.	Test Material	Dose Level (vg)	Dose Volume (mL)	Dose Concentration (vg/mL)	Main Study		Necropsy Day 29 (+/-1) M/F	
					No. of Males	No. of Females		Necropsy Day 91 (+/-2) M/F
1	Vehicle Control	0	0.1	0	10	10	5/5	5/5
2	scAAV9-CSA	1.49×10^{11}	0.1	1.49×10^{12}	10	10	-	10/10
3	scAAV9-CSA	4.47×10^{11}	0.1	4.47×10^{12}	10	10	-	10/10

Appendix 1

Group No.	Test Material	Dose Level (vg)	Dose Volume (mL)	Dose Concentration (vg/mL)	Main Study		Necropsy Day 29 (+/-1) M/F	
					No. of Males	No. of Females		Necropsy Day 91 (+/-2) M/F
4	scAAV9-CSA	1.49×10^{12}	0.1	1.49×10^{13}	15	15	5/5	10/10

9.1. Administration/Application of Test and Vehicle Control Material(s)

Route of Administration: Intrathecal Injection

Treatment Frequency/Duration: Once on Day 1

Details: Post-acquisition, test article will be maintained on wet ice. Prior to dosing the vehicle or test article will acclimate to ambient. An equipment list containing information regarding the dosing apparatus will be maintained in the study records.

10. SURGICAL ANIMAL PREPARATION

10.1. Pre-operative Procedures

Details: Anesthesia will be induced and maintained as indicated in Testing Facility SOP.

10.2. Surgical Procedure

The procedures outlined below are intended to serve as guidance. Alterations in the surgical procedures may occur as necessary and will be communicated to the Study Director. These alterations will be documented in the study data and will not be considered a protocol deviation.

A 27-gauge custom LASI needle (or equivalent) and an appropriately sized gas tight Hamilton syringe will be used to administer test article(s). Prior to dosing, the dosing system will be primed with control article or test article, as applicable. After the system is primed, the appropriate amount of test material will be drawn up. The system will not be re-primed between animals. A new needle will be used for each group or as needed. The priming steps will be repeated if a new needle is used. If a flush is needed for dosing, IT control article will be used. This will be documented in the study data. The injection site location will be documented in the study data. The duration of dose is 60 seconds +/- 5 seconds.

10.3. Postoperative Procedures

Details: Postoperative procedures will be conducted in accordance with Testing Facility SOP.

Appendix 1

11. IN-LIFE PROCEDURES, OBSERVATIONS, AND MEASUREMENTS

General In-life Assessments – Main Study Animals

Parameter	Population(s)	Target Frequency (minimum required)	Comments
Mortality/Cageside Observations	All surviving animals	At least twice daily (morning and afternoon) beginning upon arrival through termination/release.[a,b]	Animals will be observed within their cage unless necessary for identification or confirmation of possible findings.<br><br>Animals will be observed for morbidity, mortality, injury, and availability of food and water. Any animals in poor health will be identified for further monitoring and possible euthanasia.
Post Dose Observations	All Main Study animals	Approximately 4 and 24 hours post dose.[c]	Animals will be observed within their cage unless necessary for identification or confirmation of possible findings.
Detailed Clinical Observations	All Main Study animals	Once weekly; from at least Week -1 and throughout the study.[c]	Animals are removed from the cage.<br><br>Animals will be observed within their cage unless necessary for identification or confirmation of possible findings.<br><br>Pretest interval will be conducted on all animals received/transferred.
Individual Body Weights	All Main Study animals	Within 3 days of arrival, and Day -1, and once weekly during the study.	Not collected from animals found dead.
Food Consumption	All Main Study animals	Once weekly[d]	Qualitative assessment will be conducted as a part of the twice daily cageside observations. Any findings of inappetence will be recorded as an unscheduled clinical observation.
Ophthalmic Examinations	All Main Study animals	Pretest (all animals including source) and all survivors examined again prior to terminal and recovery necropsy.	See details in section below.

Appendix 1

Parameter	Population(s)	Target Frequency (minimum required)	Comments
Neurobehavioral Evaluations	All Main Study animals	Day -1, and prior to terminal necropsy. Recovery animals will also be evaluated approximately 4 weeks after the end of the Test Material administration. The same animals designated for behavioral testing will be tested at all times.	See details in section below.

[a] To include alternate animals until released from study.
[b] Except on days of receipt and necropsy where frequency will be at least once daily.
[c] For observations that cannot be attributed to an individual animal due to social housing (e.g., watery feces), the observation will be noted to each animal in the socialized group.
[d] For observations of reduced appetite that cannot be attributed to an individual animal due to social housing, the observations will be noted for each animal in the socialized group.

11.1. Ophthalmic Exams

Details: The ophthalmoscopic examinations will be conducted by a staff ophthalmologist via indirect ophthalmoscopy. Digital images for illustrative purposes may be taken. These images will not be used for data generation or interpretation and will not be archived or included in the Final Report.

Slit-lamp exams may be conducted at the discretion of the ophthalmologist.

11.1. Neurobehavioral Evaluations

Neurobehavioral evaluations will be conducted on all animals without knowledge on the part of the testers of the treatment groups (blinded evaluations). PPE need not be changed between groups during dose administration when animals are dosed in random order due to blinding. Each animal will be observed for 2 minutes in an opaque open-field observation box. Parameters will be evaluated per Testing Facility SOPs and study specific procedures. The following evaluations will be conducted:

Parameter Observed	Type of Data	Rating Scale or Unit of Measurement
Posture/Body carriage	Categorical	-2 to 0
Convulsions	Categorical	0 to 4
Stereotypy	Categorical	0 to 4
Tremor	Categorical	0 to 3
Palpebral closure/Ptosis	Categorical	0 to 2
Ease of removal	Categorical	-1 to 2
Handling reactivity	Categorical	-1 to 2
Rearing	Continuous	Counts

Appendix 1

Parameter Observed	Type of Data	Rating Scale or Unit of Measurement
Arousal/Alertness	Categorical	-2 to 2
Gait/Mobility	Categorical	0 to 3
Vocalizations	Categorical	0 to 1
Respiration	Categorical	-1 to 2
Defecation	Categorical	-1 to 1
Appearance	Categorical	0 to 2
Lacrimation	Categorical	0 to 2
Salivation	Categorical	0 to 2
Exophthalmus	Categorical	0 to 1
Erected fur	Categorical	0 to 1
Touch response/Tactile reflex	Categorical	-1 to 1
Startle response	Categorical	-1 to 1
Thermal response	Continuous	Time in seconds
Pupil response	Categorical	0 to 2
Body temperature	Continuous	℃
Body tone	Categorical	-1 to 1
Air righting reflex	Categorical	0 to 2
Grip strength (forelimb and hindlimb)	Continuous	kg

12. CLINICAL PATHOLOGY

12.1. Sample Collection

Clinical Pathology Sample Collection

Group Nos.	Time Point(s)	Recollection of Sample Acceptable[a]	Hematology	Coagulation	Clinical Chemistry
All animals	Prior to necropsy on days 29 (+/-1) and 91 (+/-2)	X	X	X	X
**Unscheduled Euthanasia (when possible)**			See Section 14.2 of this protocol.		
**Fasting:**			Animals will not be fasted.		
**Method/Comments:**			Cardiac puncture after carbon dioxide inhalation		
**Target Volume[a]:**			0.25 mL	1.2 mL	1.0 mL
**Anticoagulant:**			K_2EDTA	Sodium Citrate	Serum Gel Separator
**Special Requirements:**			-	-	-
**Processing:**			None	Plasma	Serum

X = Sample to be collected; - = Not applicable; hr = Hour; pre = Predose; post= Postdose

[a] Additional samples may be obtained as per procedure if permissible sampling Frequency and volume are not exceeded.

[b] Add footnote.

Blood Sample Special Terminal – Cardiac puncture after carbon dioxide inhalation.
Instructions:

Special Requirements: Due to the sample volume size and the number of tests indicated, it is likely that not all tests will be completed for all animals. The study data and Final Report will indicate tests not conducted as insufficient sample where appropriate. Blood collection priority is as follows: HCC>Biodistribution> ADA.

The following clinical pathology tests will be conducted on available samples, as survival allows, and the data will be interpreted by a Clinical Pathologist.

12.2. Hematology Evaluation

Hematology Parameters[a]

Red blood cell count<br>Hemoglobin concentration<br>Hematocrit<br>Mean corpuscular volume<br>Red blood cell distribution width<br>Mean corpuscular hemoglobin concentration<br>Mean corpuscular hemoglobin<br>Reticulocyte count (absolute and percent)<br>Platelet count	White blood cell count<br>Neutrophil count (absolute and percent)<br>Lymphocyte count (absolute and percent)<br>Monocyte count (absolute and percent)<br>Eosinophil count (absolute and percent)<br>Basophil count (absolute and percent)<br>Other cells (as appropriate)<br>Blood smear (preserve and stain[b])

[a] Analysis performed using Sysmex XN-1000V method.

[b] A blood smear will be prepared from each hematology sample. Blood smear review may be performed on select animals per Testing Facility SOP.

12.3. Coagulation Evaluation

Coagulation Parameters

Prothrombin time<br>Activated partial thromboplastin time	Fibrinogen<br>Sample quality

12.4. Clinical Chemistry Evaluation

Clinical Chemistry Parameters

Alkaline phosphatase<br>Total bilirubin[a]<br>Aspartate aminotransferase<br>Alanine aminotransferase<br>Gamma glutamyl transferase<br>Sorbitol dehydrogenase<br>Urea nitrogen<br>Creatinine<br>Total protein	Albumin<br>Globulin (calculated)<br>Albumin/globulin ratio (calculated)<br>Glucose<br>Total cholesterol<br>Triglycerides<br>Electrolytes (sodium, potassium, chloride)<br>Calcium<br>Phosphorus<br>Sample quality

[a] When total bilirubin is >0.5 mg/dL, direct bilirubin will also be measured, and indirect bilirubin will be calculated.

13. ADA AND BIODISTRIBUTION EVALUATION

13.1. Biodistribution Sample Collection

Biodistribution Sample Collection

Group Nos.	Target Postdose Collection Intervals – Days 29 and 91 (+/-2)
	Terminal necropsy
All	X

X = Sample to be collected; - = Not applicable; min = Minute; hr/hrs = Hour/hours.

13.2. Biodistribution Sample Processing

Collection Method:	Cardiac puncture after carbon dioxide inhalation
Target Volume:	1 mL
Anticoagulant:	K_2EDTA
Fasting:	The animals will not be fasted before blood collection unless concurrent with fasting for other procedures.
Whole Blood Storage:	Wet ice or ice block
Processing:	Whole blood will be split into two approximately equal aliquots, flash frozen on dry ice and shipped to the Sponsor for biodistribution analysis (rt-qPCR and qPCR analysis).
Additional Container Label Requirements:	Study number, animal number, matrix of sample, interval and timepoint, analysis type, and aliquot number (if applicable).
Final Storage Temperature:	Frozen (-60 to -90°C)<br>Processed samples will be placed on dry ice or in Frozen (-60 to -90°C) storage within 4 hours of collection.

13.3. Biodistribution Sample Shipment

Bioanalytical Sample Shipping Contact:	All samples to be analyzed will be shipped to the Sponsor, see Attachment B for shipping details.

13.4. Biodistribution Sample Analysis

Analysis Performed By:	All analytical work will be conducted by the Sponsor.
Test Site Regulatory Requirements:	The work performed in conjunction with this study will not be conducted in compliance with GLPs. A Final Report will be prepared and submitted

Appendix 1

to Testing Facility for inclusion as an appendix in the main study Final Report.

13.5. ADA Sample Collection

ADA Sample Collection

Group Nos.	Target Postdose Collection Intervals – Days 29 and 91
	Terminal necropsy
All	X

X = Sample to be collected; - = Not applicable; min = Minute; hr/hrs = Hour/hours

13.6. ADA Sample Processing

Collection Method:	Cardiac puncture after carbon dioxide inhalation
Target Volume:	1 mL
Anticoagulant:	None, Serum Gel Separator
Fasting:	The animals will not be fasted before blood collection unless concurrent with fasting for other procedures.
Whole Blood Storage:	Ambient until centrifuged.
Processing:	Samples will be processed serum, split into two approximately equal aliquots and frozen. Both aliquots will be shipped to the Sponsor for analysis (one for Vector ADA and the other for Transgene ADA).
Additional Container Label Requirements:	Study number, animal number, matrix of sample, interval and timepoint, analysis type, and aliquot number (if applicable).
Final Storage Temperature:	Frozen (-60 to -90°C)

13.7. ADA Sample Shipment

Bioanalytical Sample Shipping Contact:	All samples to be analyzed will be shipped to the Sponsor, see Attachment B for shipping details.

13.8. ADA Sample Analysis

Analysis Performed By:	The samples will be saved for possible future analysis.

DRAFT, NOT FOR SUBMISSION

Appendix 1

14. TERMINAL PROCEDURES

Terminal procedures are summarized in the following tables:

Unscheduled Deaths

Unscheduled Euthanasia	Necropsy Procedures				Histology Processing	Microscopic Evaluation
	Necropsy	Organ Weights	Tissue Collection			
			Fixed	Frozen		
Found Dead or Perfusion not possible Refrigerated	X	-	Full List[a]	-	Full List[a]	Full List[a]
Perfusion possible Non-Refrigerated	X	Full List[a]	Full List[a]	Full List[a]	Full List[a]	Full List[a]

Main Study Animals

Group No.	Scheduled Euthanasia Day	Necropsy Procedures				Histology Processing	Microscopic Evaluation
		Necropsy	Organ Weights	Tissue Collection			
				Fixed	Frozen		
All animals	29 (+/-1) and 91(+/-2)	X	Full List[a]	Full List[a]	Full List[a]	Full List[a]	Full List[a]

X = Procedure to be conducted; - = Not applicable.
'Histology Processing' = embedded in paraffin, sectioned, mounted on glass slides, and stained with hematoxylin and eosin.
a See Tissue Weighing, Collection, Processing and Evaluation table, (Attachment A) for list of tissues applicable to each procedure.

14.1. Method of Euthanasia

Euthanasia will be by carbon dioxide inhalation followed by a Testing Facility SOP approved method to ensure death, e.g., exsanguination or perfusion.

14.2. Unscheduled Euthanasia

Animals will be subject to Testing Facility SOP criteria and procedures for early euthanasia or when found dead. If possible, the samples below will be collected from animals euthanized following veterinary consultation. A veterinary consultation is not required if the samples are collected following anesthesia or euthanasia. Blood collection methods utilized for animals euthanized early may include suitable methods other than those presented in the respective blood collection section(s) of this Protocol.

Sample Type	Groups	Target Volume	Anticoagulant
Hematology	All	0.25 mL	K_2EDTA
Coagulation		1.2 mL	Sodium Citrate
Clinical Chemistry		1.0 mL	Serum Gel Separator
Biodistribution	All	1.0 mL	K_2EDTA
ADA	All	1.0 mL	Serum Gel Separator

Necropsy examinations will be performed 7 days a week. Animals that are found dead or euthanized early after regular working hours will be refrigerated and necropsies performed at the start of the next day.

14.3. Scheduled Euthanasia

Main Study animals surviving until scheduled euthanasia will be euthanized by the methods described above.

14.4. Necropsy

Animals as detailed in the Terminal Procedures table will be subjected to a complete necropsy examination, which will include evaluation of the carcass and musculoskeletal system; all external surfaces and orifices; cranial cavity and external surfaces of the brain; and thoracic, abdominal, and pelvic cavities with their associated organs and tissues. Veterinary pathologist or other suitably qualified personnel will be available for consultation during scheduled necropsy.

All animals will be examined carefully for external abnormalities including palpable masses.

Following euthanasia, animals will be perfused with chilled 0.9% sterile saline (for at least 5 minutes or until fluid runs clear). Effort will be made, if possible, to perfuse unscheduled euthanasia animals during standard business hours. Animals found dead or euthanized outside of standard business hours will not be perfused.

Images may be generated for illustration of or consultation on gross observations. These images will not be used for data generation or interpretation and will not be archived or included in the Final Report.

14.5. Organ Weights

Body weight and the organ weights identified in the Tissue Weighing, Collection, Processing and Evaluation table (Attachment A) will be recorded for all animals at the scheduled necropsies, and appropriate organ weight ratios will be calculated (relative to body and brain weights). Paired organs will be weighed together. Organ weights will be collected per Testing Facility SOP.

14.6. Tissue Collection and Preservation

Representative samples of tissues will be collected and preserved in 10% neutral buffered formalin, as detailed in the Terminal Procedures table and Tissue Weighing, Collection, Processing, and Evaluation table (Attachment A). Additional tissue samples may be collected to elucidate abnormal findings.

For tissues that are too small to accommodate all requested samples, sample collection priority will be as follows (samples not collected due to limited size will be noted in the study record, but not considered a protocol deviation): Histology>qPCR.

Appendix 1

14.6.1. Brain Tissue Collection Details

The entire brain will be sliced into 3 mm coronal slabs using a brain matrix. Slabs will be arranged in sequential order (rostral to caudal) and photographed (caudal surface). If additional sampling from any slab is required, images will be taken both before and after any frozen samples are collected. For unscheduled euthanasia animals that do not require frozen sample collection at necropsy, brains may be fixed whole, then slabbed and sections designated for histology processing at the time of fixed tissue trimming and trimmed following the standard CRL histology blocking scheme.

Slabs will be designated for collection and processing as follows:

Each brain slab will be bisected into left and right hemispheres along the sagittal midline. The left hemislabs will be wrapped in parafilm, then wrapped in aluminum foil, then frozen on dry ice. Then the slabs will be stored in pre- cooled 5 ml tubes and stored frozen (-60 to -90°C) until shipment to the sponsor.

The right hemislabs will be preserved in 10% NBF for histopathology

For fixed brain samples, the designated slab hemislab will be submitted rostral side down in a standard cassettes, to maintain orientation (labeled with the slab number, left/right). Select fixed hemi-slabs will be processed for microscopic evaluation following the standard CRL blocking scheme.

14.7. Biodistribution Frozen Tissue Collection

Care will be given to ensure that cross contamination between tissues does not occur. Gloves and disposable collection tools will be changed between collection and dissection of each tissue for qPCR biodistribution analysis. The cutting board and non-disposable instruments will be wiped down with a 10% bleach solution, rinsed with water, followed by a wipe down of 70% or greater ethanol between each of the specified organs; sterile trays may be used. Samples for qPCR will not be collected for animals found dead or euthanized in extremis where perfusion is not possible.

Intervals and Number of Animals:	All survivors at necropsy (Day 29) and necropsy (Day 91) and any animal euthanized with perfusion possible.
Tissues to Collect:	See Attachment A for list of tissues applicable to each procedure.
Sample Collection:	A portion of each required tissue, without sacrificing what is needed for histopathology, will be collected for analysis as follows:<br>Individual sample size: [2-3 1-2 mm pieces]. Individual sample weight will not be recorded.<br>1 DRG pair per level.

DRAFT, NOT FOR SUBMISSION

	All samples will be placed into pre-labeled polypropylene vials, tightly capped, and frozen in liquid nitrogen. Care will be taken to minimize the time between sample collection and freezing. Samples may be held on dry ice until final storage.
Storage Conditions:	Frozen (-60° to -90°C)
Label Requirements:	Testing Facility study number, Sponsor study number (if applicable), animal number, group number, nominal study day, nominal time point (if applicable), analysis type (e.g., Biodistribution), tissue type (e.g., ORGANS), and if possible, the date of collection.
Sample Shipment:	Samples will be shipped to the Test Site, see Attachment B for shipping details.

14.7.1. Biodistribution Frozen Tissue Sample Analysis

Analysis Performed By:	Samples will be analyzed for test article concentration. All analytical work will be conducted by the Sponsor-
Test Site Regulatory Requirements:	The work performed in conjunction with this study will not be conducted in compliance with GLPs. A Final Report will be prepared and submitted to Testing Facility for inclusion as an appendix in the main study Final Report.

14.8. Spleen Sample Collection for isolation of PBMC for ELISpot assays

From animals (15) scheduled for necropsy on the final two days of necropsy for the study, collect a target of 1/3 of the spleen for PBMC sample analysis in sponsor provided tubes and media (approximately 10mL/tube). The remaining spleen will be used for histopathology and biodistribution analysis.

Ship samples that are collected from the first necropsy, on ice packs to the following address:

Meghan Blackwood/Keeler Lab
Paul J. DiMare Center
N8-2003
366 Plantation Street
Worcester MA 01605

Appendix 1

Ship samples collected on the second necropsy, on ice packs, to the following address:
Rita Batista
32 Shady Ln
Holden, MA 01520

14.8.1. Spleen Sample Collection for isolation of PBMC for ELISpot assays

**Analysis Performed By:**	**Samples will used to isolate PBMC to conduct ELISpot assays to evaluate adaptive immunological responses to AAV9 capsid and human CSA protein. All analytical work will be conducted by the Sponsor.**
**Test Site Regulatory Requirements:**	**The work performed in conjunction with this study will not be conducted in compliance with GLPs. A Final Report will be prepared and submitted to Testing Facility for inclusion as an appendix in the main study Final Report.**

15. HISTOLOGY AND MICROSCOPIC EVALUATION

15.1. Histology

Histology will be conducted per Testing Facility SOPs.

15.2. Microscopic Evaluation

Tissues as detailed in the Tissue Weighing, Collection, Processing and Evaluation table (Attachment A) will be evaluated histopathologically by light microscopy) by a board-certified veterinary as identified in the Responsible Personnel section of this Protocol.

Special stains may be used at the discretion of the pathologist to further characterize lesions and changes identified during routine evaluation of individual animals. Any special stains will be documented in the individual animal data. Any additional stains or evaluations, if deemed necessary by the pathologist, may be added by Protocol Amendment following discussion with the Study Director and in consultation with the Sponsor. Efforts will be made to evaluate all Protocol-required tissues microscopically; however, it is not always feasible for every Protocol-required tissue to be present on every slide. Protocol required tissues that are not examined will be documented in the histopathology data and the impact of these missing tissues on the study will be documented in the Pathology Report.

Images may be generated for illustration of or consultation on histological observations. These images will not be used for data generation or interpretation and will not be archived or included in the Final Report.

Photomicrographs of representative microscopic lesions will be taken, archived in the study data, and all or representative images will be included in the Final Report.

In the unlikely event that a Testing Facility pathologist cannot be scheduled for the pathology evaluation due to logistics/timing, the pathology evaluation may be outsourced (by protocol amendment) to an external ACVP-certified veterinary pathologist.

16. STATISTICAL ANALYSIS

Statistical analysis will not be performed; however, descriptive statistics will be calculated. For continuous endpoints, descriptive statistics will consist of means, standard deviations, and group size for each treatment group and time period. For categorical endpoints, descriptive statistics will consist either of medians or incident counts for each treatment group and time period.

Type of Analysis: Descriptive Statistics

Endpoints:

- Body Weight and Body Weight Change
- Food Consumption
- Neurobehavioral Evaluations
- Hematology
- Coagulation
- Clinical Chemistry
- Organ Weights
- Absolute Weights
- Relative to Body and Brain Weights

16.1. Statistical Comparisons

Control Group	Comparison Group(s)
1	2, 3, 4

17. COMPUTERIZED SYSTEMS

The following critical computerized systems may be used in the study. The actual critical computerized systems used will be specified in the Final Report. The implemented paper and electronic systems are fit for the integrity of the study data.

Critical Computerized Systems

System Name	Description
eInfotree	Excel Module for data collection with 21 CFR Part 11 compliance requirements, security, audit trail, and electronic signatures.
ExyLIMS	A comprehensive laboratory information management system used to manage data, including but not limited to instrumentation, test article(s), standards, and samples.

System Name	Description
In-house reporting software Nevis 2012 (using SAS)	In-life; clinical pathology; postmortem
Logbook	Electronic notebook and data collection system for veterinary communications, observations, and treatments.
M-Files®	Reporting and collection of 21 CFR Part 11 compliant signature
Provantis®	In-life; clinical pathology; postmortem; test article(s)receipt, accountability, and/or formulation activities
SAS®	An integrated system of software products that enables a user to perform data entry, retrieval, data management, reporting, graphics, statistical analysis, and applications development.
Siemens Monitoring System	Collects and report temperature and humidity data and controls some HVAC systems.

Data for parameters not required by Protocol, which are automatically generated by analytical devices used will be retained on file but not reported. Statistical analysis results that are generated by the program but are not required by Protocol and/or are not scientifically relevant will be retained on file but will not be included in the tabulations.

18. REGULATORY COMPLIANCE

The study will be conducted in accordance with the U.S. Department of Health and Human Services, Food and Drug Administration, United States Code of Federal Regulations, Title 21, Part 58: Good Laboratory Practice for Nonclinical Laboratory Studies and as accepted by Regulatory Authorities throughout the European Union (OECD Principles of Good Laboratory Practice), Japan (MHLW), and other countries that are signatories to the OECD Mutual Acceptance of Data Agreement.

Exceptions to GLPs include the following study elements:

- Characterization of the test and control materials will be/were performed by the Sponsor or Sponsor subcontractor according to established SOPs, controls, and approved test methodologies to ensure integrity and validity of the results generated; these analyses will not be/were not conducted in compliance with the GLP regulations.
- The Biodistribution assays will not be performed within the scope of regulations governing the conduct of nonclinical laboratory studies and is not intended to comply with such regulations.
- Dose formulation and ADA analysis will not be conducted, samples will be held for possible future analysis. If additional data are obtained from analyses of these samples, the Study Director will be notified and will assess whether there is an impact to the study, after consultation with the Sponsor. If the Study Director determines there is impact to the study, these data will be included in an Amended Final Report. If the Study Director determines there is no impact, this decision and data will be documented and archived with the study records.

DRAFT, NOT FOR SUBMISSION

19. QUALITY ASSURANCE

19.1. Testing Facility

The Testing Facility Quality Assurance Unit (QAU) will monitor the study to assure the facilities, equipment, personnel, methods, practices, records, and controls are in conformance with Good Laboratory Practice regulations. The QAU will review the Protocol, conduct inspections at intervals adequate to assure the integrity of the study, and audit the Final Report to assure that it accurately describes the methods and standard operating procedures and that the reported results accurately reflect the raw data of the study.

20. AMENDMENTS AND DEVIATIONS

Changes to the approved Protocol shall be made in the form of an amendment, which will be signed and dated by the Study Director. Every reasonable effort will be made to discuss any necessary Protocol changes in advance with the Sponsor. The Study Director will notify the Sponsor of deviations that may result in a significant impact on the study as soon as possible.

21. RETENTION AND DISPOSITION OF RECORDS, SAMPLES, AND SPECIMENS

All study-specific raw data, electronic data, documentation, Protocol, retained samples and specimens, and Interim (if applicable) and Final Reports will be archived by no later than the date of Final Report issue. All materials generated by Charles River Laboratories from this study will be transferred to a Charles River Laboratories archive. At least 1 year after issue of the Draft Report, the Sponsor will be contacted.

Samples for clinical pathology evaluations are discarded per Testing Facility SOP unless otherwise indicated in the table below.

Disposition of residual/retained analytical samples will be as described in the table below.

Disposition of Residual/Retained Samples

Sample Type	Disposition	Schedule
Dose Formulation Preparation Sample	Ship to Sponsor	The disposition of the samples will occur as per Protocol if definitive results are within acceptance criteria and have been provided to the Study Director. If results are out of specification, the Study Director will need to provide approval for disposition.
Residual Dosing Formulation	Ship to Sponsor	Per disposition indicated in the Preparation of Formulations section.
ADA/Biodistribution	Ship to Sponsor	Samples will be maintained for a minimum of 6 months following issuance of the Draft Report or at an alternate time point prior to finalization as requested by the Study Director.

Appendix 1

Sample Type	Disposition	Schedule
Pathology slides	Archive	Return to Testing Facility after Issuance of the Draft Report

Records to be maintained will include, but will not be limited to, documentation and data for the following:

- Deviations, Protocol amendments, and Protocol
- Study schedule
- Study-related correspondence
- Test system receipt, health, and husbandry
- Test and control material receipt, identification, preparation, and analysis
- In-life measurements and observations
- Clinical pathology sample collection and evaluation
- Bioanalytical sample collection and evaluation
- Gross and microscopic observations and related data
- Organ weight measurements
- Statistical analysis results

22. STUDY CLASSIFICATION

Submission Study Category:	Toxicology
Submission Study Type:	Single Dose Toxcitiy
Study Design:	Parallel
Primary Treatment CAS Registry Number:	Not Applicable
Primary Treatment Unique Ingredient ID:	Not Applicable
Pharmacologic Class:	Biologic – AAV vector
Administration Dose Form:	Solution

23. REPORTING

An audited Interim Draft report containing the results through the Day 29 necropsy timepoint will be prepared and issued to the Sponsor for regulatory filing.

The Sponsor will receive an electronic version of the Draft and Final Reports, provided in Adobe Acrobat PDF format (hyperlinked and searchable at final). The PDF document will be created from native electronic files to the extent possible, including text and tables generated by the Testing Facility. Report components not available in native electronic files and/or original signature pages will be scanned and converted to PDF image files for incorporation.

Appendix 1

Reports should be finalized within 6 months of issue of the Draft Report. If the Sponsor has not provided comments to the report within 6 months of draft issue, the report will be finalized by the Testing Facility unless other arrangements are made by the Sponsor.

23.1. SEND Datasets

SEND datasets will be generated and provided outside the context of the GLP Report. These datasets will not be subject to QA Audit nor will they be used as the basis for the Study Director interpretation of the study results. SEND datasets will be provided for the Report based on regulatory submission date. The Sponsor is expected to provide submission dates.

When work in support of this study is conducted at a Test Site (i.e., Bioanalysis, TK modeling, etc.), an electronic version of all data should be provided to Testing Facility.

24. JUSTIFICATIONS AND GUIDELINES

24.1. Justification of Test System and Number of Animals

At this time, studies in laboratory animals provide the best available basis for extrapolation to humans and are required to support regulatory submissions. Acceptable models that do not use live animals currently do not exist.

The rat was chosen as the animal model for this study as it is an accepted rodent species for nonclinical toxicity testing by regulatory agencies.

The total number of animals to be used in this study is considered to be the minimum required to properly characterize the effects of the test article(s). This study has been designed such that it does not require an unnecessary number of animals to accomplish its objectives. Details on the group distribution, number of test groups, and number of animals per group are included in the Experimental Design section.

24.2. Justification of Route and Dose Levels

Intrathecal injection is one of the intended routes of administration of potential routes of human exposure to this test article(s).

The dose levels were selected based on information provided by the Sponsor references, and in an attempt to produce graded responses to the test article item substance(s). The high-dose level should produce some toxic effects, but not excessive lethality that would prevent meaningful evaluation. The mid-dose level is expected to produce minimal to moderate toxic effects. Intermediate dose levels are expected to produce minimal to moderate toxic effects. The low-dose level should produce no observable indications of toxicity.

24.3. Guidelines for Study Design

The design of this study was based on the study objective(s) and the overall product development strategy for the test article(s).

- FDA Guidance for Industry. Preclinical Assessment of Investigational Cellular and Gene Therapy Products.
- FDA Guidance for Industry. Human Gene Therapy for Rare Diseases.
- FDA Draft Guidance for Industry. Human Gene Therapy for Neurodegenerative Diseases.

25. ANIMAL WELFARE

This study will comply with all applicable sections of the Final Rules of the Animal Welfare Act regulations (Code of Federal Regulations, Title 9), the *Public Health Service Policy on Humane Care and Use of Laboratory Animals* from the Office of Laboratory Animal Welfare (OLAW, current edition), and the *Guide for the Care and Use of Laboratory Animals* (NRC, current edition). The Protocol and any amendments or procedures involving the care or use of animals in this study will be reviewed and approved by the Testing Facility Institutional Animal Care and Use Committee before the initiation of such procedures.

If an animal is determined to be in overt pain/distress or appears moribund and is beyond the point where recovery appears reasonable, the animal will be euthanized for humane reasons in accordance with the *American Veterinary Medical Association (AVMA) Guidelines on Euthanasia* (AVMA, current edition) and with the procedures outlined in the Protocol.

By approving this Protocol, the Sponsor affirms that there are no acceptable non-animal alternatives for this study, that this study is required by a relevant government regulatory agency(ies) and that it does not unnecessarily duplicate any previous experiments.

25.1. Institutional Animal Care and Use Committee Approval

The Protocol and any amendment(s) or procedures involving the care and use of animals in this study will be reviewed and approved by Charles River Laboratories, Mattawan Institutional Animal Care and Use Committee (IACUC) before conduct. During the study, the care and use of animals will be conducted with guidance from the guidelines of the USA National Research Council.

26. REFERENCES

American Veterinary Medical Association. *AVMA Guidelines on Euthanasia*. Current edition.

National Research Council. *Guide for the Care and Use of Laboratory Animals*. Current edition.

Office of Laboratory Animal Welfare. *Public Health Services Policy on Humane Care and Use of Laboratory Animals*. Bethesda, MD: National Institutes of Health. Current edition.

Appendix 1

ATTACHMENT A

Tissue Weighing, Collection, Processing and Evaluation Table

Organ	Organ Weight	Fixed Tissue Collection	Histology Processing	Microscopic Evaluation	Biodistribution Collection and Analysis
Animal ID	-	X	-	-	-
Artery, aorta	-	X	-	-	-
Body cavity, nasal	-	X	-	-	-
Bone marrow smear	-	X[a]	-	-	-
Bone marrow, sternum	-	X	-	-	-
Bone, femur	-	X (1)	-	-	-
Bone, sternum	-	X	-	-	-
Brain	X	X	X	X	X
Dorsal Root Ganglia, cervical	-	X(2)	X(2)	X(2)	X
Dorsal Root Ganglia, thoracic	-	X(2)	X(2)	X(2)	X
Dorsal Root Ganglia, lumbar	-	X(2)	X(2)	X(2)	X
Epididymis	X (2)	X (2)[b]	-	-	-
Esophagus	-	X	-	-	-
Eye	-	X (2)[b]	-	-	-
Gland, adrenal	X (2)	X (1)	X (1)	X (1)	X (1)
Gland, clitoral	-	X (2)	-	-	-
Gland, Harderian	-	X (2)	-	-	-
Gland, lacrimal	-	X (2)	-	-	-
Gland, mammary	-	X	X	X	X
Gland, parathyroid	-	X (2)	-	-	-
Gland, pituitary	X	X	-	-	-
Gland, preputial	-	X (2)	-	-	-
Gland, prostate	X	X	-	-	-
Gland, salivary, parotid	-	X (2)	-	-	-
Gland, salivary, sublingual	-	X (2)	-	-	-
Gland, salivary, mandibular	-	X (2)	-	-	-
Gland, seminal vesicle	-	X (2)	-	-	-
Gland, thyroid	-	X (2)	-	-	-
Gland, thyroid/parathyroid	X (2)	X (2)	-	-	-
Gland, Zymbal's	-	X (2)	-	-	-
Gut-associated lymphoid tissue	-	X	-	-	-
Heart	X	X	X	X	X
Joint, femorotibial	-	X (1)	-	-	-
Kidney	X (2)	X (2)	X (2)	X (2)	X (1)
Large intestine, cecum	-	X	-	-	-
Large intestine, colon	-	X	X	X	-

DRAFT, NOT FOR SUBMISSION

Appendix 1

Organ	Organ Weight	Fixed Tissue Collection	Histology Processing	Microscopic Evaluation	Biodistribution Collection and Analysis
Large intestine, rectum	-	X	-	-	-
Larynx	-	X	-	-	-
Liver	X	X	X	X	X
Lung	-	X	X	X	X
Lymph node(s) draining administration site(s): Iliac draining lymph node	-	X (1)	X (1)	X (1)	-
Lymph node, mandibular	-	X (2)	-	-	-
Lymph node, mesenteric	-	X	-	-	-
Muscle, skeletal	-	X (2)	-	-	-
Nerve, optic	-	X (2)[b]	-	-	-
Nerve, sciatic	-	X (2)	X (1)	X (1)	-
Nerve, tibial	-	X (2)	-	-	-
Ovary	X (2)	X (1)	X (1)	X (1)	X (1)
Oviduct	-	X (2)	-	-	-
Pancreas	-	X	X	X	X
Skin	-	X	-	-	-
Small intestine, duodenum	-	X	-	-	-
Small intestine, ileum	-	X	-	-	-
Small intestine, jejunum	-	X	-	-	-
Spinal cord, cervical	-	X	X	X	X
Spinal cord, thoracic	-	X	X	X	X
Spinal cord, lumbar	-	X	X	X	X
Spleen	X	X	X	X	X
Stomach	-	X	-	-	-
Testis	X (2)	X (Right)	X (Right)	X (Right)	X (1) (Left)
Thymus	X	X	-	-	-
Tongue	-	X	-	-	-
Trachea	-	X	-	-	-
Ureter	-	X (2)	-	-	-
Urinary bladder	-	X	-	-	-
Uterus/Cervix	X	X	-	-	-
Vagina	-	X	-	-	-
Macroscopic abnormalities[c]	-	X	X	X	X

X = Procedure to be conducted. - = Not applicable. (1) = one side. (2) = both sides.

[a] Bone marrow smears will be collected from the femur at scheduled and unscheduled necropsies (for possible examination). Smears will not be collected from animals that are found dead or from animals that were euthanized moribund and then stored in the refrigerator prior to necropsy. Bone marrow smears will be allowed to air dry and are not fixed in formalin.

[b] Eyes and optic nerves will be preserved in Davidson’s fixative. Testes and epididymides will be preserved in modified Davidson’s fixative.

[c] Macroscopic abnormalities in the tissues listed, and in any other tissues, will be sampled, processed and evaluated histologically.

Appendix 1

ATTACHMENT B

Samples will be shipped on Monday through Wednesday for next day delivery for domestic shipments and expedited delivery for international shipments using the Conditions for Shipment(s) listed below.

Shipment of Samples and Study Records

Sample Matrix	Sample Type	Aliquot of Sample	Proposed Shipment Timeline	Conditions for Shipment	Recipient/Address
Vehicle/Test article	Dose Formulations Residual Dosing Formulation	All	After collection	cold packs wet ice or ice blocks on dry ice Temperature monitor to be included in shipment.	Miguel Sena Esteves UMass Chan Medical School Dept. Genetic and Cellular Medicine DiMare Center, N8-1001 368 Plantation Street Worchester, MA 01605 Tel: 508-856-4412 E-mail: Miguel.esteves@umassmed.edu
Serum/Whole Bood	ADA/Biodistribution	All	After final necropsy collection	ice blocks on dry ice Temperature monitor to be included in shipment	Miguel Sena Esteves UMass Chan Medical School Dept. Genetic and Cellular Medicine DiMare Center, N8-1001 368 Plantation Street Worchester, MA 01605 Tel: 508-856-4412 E-mail: Miguel.esteves@umassmed.edu
Tissue	Biodistribution	All	After the final necropsy collection	ice blocks on dry ice Temperature monitor to be included in shipment	Miguel Sena EstevesUMass Chan Medical School Dept. Genetic and Cellular MedicineDiMare Center, N8-1001 368 Plantation Street Worchester, MA 01605 Tel: 508-856-4412 E-mail: Miguel.esteves@umassmed.edu

Appendix 1

SPONSOR APPROVAL

The Protocol Amendment was approved by the Sponsor by e-mail on the date designated below. The correspondence giving approval will be archived, as appropriate with other Sponsor communications.

28 Oct 2025

Date of Sponsor Approval

DRAFT, NOT FOR SUBMISSION

Appendix 1

TESTING FACILITY APPROVAL

All electronic signatures appear at the end of the document upon finalization.

Appendix 1

SIGNATURE(S) FOR DOCUMENT: 32025165 - Protocol Amendment 4

**Study Director Approval:**	I approve this document.	
Name:	**Gunter, Barak**	
		29-Oct-2025 13:01:46 (UTC+00:00)
Electronically Signed in M-Files		Timestamp

Appendix 1

Final Protocol

Testing Facility Study No. 32025165

Sponsor Reference No. 32025165

A 13 Week IT Toxicity Study in Rats with 4-Week and 13-Week Biodistribution

GLP

SPONSOR:
University of Massachusetts
55 N Lake Avenue
Worcester, MA 01605-2324
United States

TESTING FACILITY:
Charles River Laboratories
54943 North Main Street
Mattawan, MI 49071
United States

DRAFT, NOT FOR SUBMISSION

Appendix 1

TABLE OF CONTENTS

1. OBJECTIVE(S) 4
2. PROPOSED STUDY SCHEDULE 4
3. SPONSOR 4
4. RESPONSIBLE PERSONNEL 5
5. TEST AND CONTROL MATERIALS 6
6. DOSE FORMULATION AND ANALYSIS 7
7. TEST SYSTEM 8
8. HUSBANDRY 9
9. EXPERIMENTAL DESIGN 11
10. SURGICAL ANIMAL PREPARATION 12
11. IN-LIFE PROCEDURES, OBSERVATIONS, AND MEASUREMENTS 12
12. CLINICAL PATHOLOGY 15
13. BIODISTRIBUTION EVALUATION 17
14. TERMINAL PROCEDURES 18
15. HISTOLOGY AND MICROSCOPIC EVALUATION 22
16. STATISTICAL ANALYSIS 23
17. COMPUTERIZED SYSTEMS 23
18. REGULATORY COMPLIANCE 24
19. QUALITY ASSURANCE 24
20. AMENDMENTS AND DEVIATIONS 24
21. RETENTION AND DISPOSITION OF RECORDS, SAMPLES, AND SPECIMENS 25
22. STUDY CLASSIFICATION 26
23. REPORTING 26
24. JUSTIFICATIONS AND GUIDELINES 27
25. ANIMAL WELFARE 28
26. REFERENCES 28

ATTACHMENT A 29
ATTACHMENT B 31
SPONSOR APPROVAL 32

Appendix 1

TESTING FACILITY APPROVAL..33

DRAFT, NOT FOR SUBMISSION

Appendix 1

1. OBJECTIVE(S)

The objective of this study is to determine the potential toxicity and biodistribution of scAAV9-CSA for the treatment of Cockyane Syndrome, when given as an intrathecal injection to rats once, and to evaluate the potential reversibility of any findings following a 28-day and 90-day recovery period.

2. PROPOSED STUDY SCHEDULE

Proposed study dates are listed below. Actual dates will be included in the Final Report.

Experimental Starting Date:	11 Jul 2025<br>(First date of study-specific data collection)
Experimental Completion Date:	To be included in the Final Report<br>(Last date on which data are collected)
Initiation of Dosing:	18 Jul 2025
Completion of In-life:	29 Oct 2025<br>(Last date of euthanasia)
Interim Report	9 weeks<br>(Following last day of necropsy or last sample analyzed)
Draft Report:	9 weeks<br>(Following last day of necropsy or last sample analyzed)
Final Report:	21 Aug 2026<br>(Expected date of Study Director signature of report)
Final SEND Dataset Package Delivery:	Based on regulatory submission date

3. SPONSOR

Role	Name/Contact Information
Sponsor Monitor	Heather Gray-Edwards DVM, PhD<br>Tel: (508) 856-5737<br>E-mail: Heather.GrayEdwards@umassmed.edu<br>Address as cited for Sponsor
Sponsor Representative	Miguel Sena-Esteves, PhD<br>Tel: (508) 856-4412<br>E-mail: Miguel.Esteves@umassmed.edu<br>Address as cited for Sponsor

Appendix 1

4. RESPONSIBLE PERSONNEL

Role/Phase	Quality Assurance Unit	Name/Contact Information
Study Director	Charles River	Barak W. Gunter, PhD<br>Tel: 269-668-3336 ext. 1099<br>E-mail: barak.gunter@crl.com<br>Address as cited for Testing Facility
Testing Facility Management	Charles River	Scott T. Wilson, BS, LAT<br>Tel: 269-668-3336 ext. 1610<br>E-mail: scott.wilson@crl.com<br>Address as cited for Testing Facility
Testing Facility QAU	Charles River	Tel: 269-668-3336<br>E-mail: MWN-QAMgmt@charlesriverlabs.com<br>Address as cited for Testing Facility
SEND	Not Applicable	SEND Data Management<br>E-mail: CRL-SEND-ES@crl.com
**Individual Scientist(s) (IS)**		
Dose Formulation Analysis	Charles River	Will be included via Amendment
Ophthalmology	Charles River	Will be included via Amendment
Anatomic Pathologist	Charles River	Will be included via Amendment
Clinical Pathologist	Charles River	Will be included via Amendment
**Principal Investigator(s) (PI)**		
Biodistribution Analysis[a]	Will be included via Amendment	Miguel Sena-Esteves, PhD<br>Tel: (508) 856-4412<br>E-mail: Miguel.Esteves@umassmed.edu<br>Address as cited for Sponsor

[a] Sponsor-designated Test Site.

Each IS and PI is required to report all deviations or other circumstances that could affect the quality or integrity of the study to the Study Director in a timely manner for authorization/acknowledgement. Each IS and PI will provide a report addressing their assigned phase of the study, which will be included as an appendix to the Final Report.

The IS Phase Report will include the following:

- A listing of critical computerized systems used in the conduct and/or interpretation of the assigned study phase.

The PI Phase Report will include the following:

- A Statement of Compliance (if the applicable phase is GLP)
- A QA Statement (for Sponsor designated PI or for Testing Facility designated PI if audited by a QAU other than that of the Testing Facility and if the applicable phase is GLP)
- The archive site for all records, samples, specimens and reports generated from the phase or segment (alternatively, details regarding the retention of the materials may be provided to the Study Director for inclusion in the Final Report), if applicable.

DRAFT, NOT FOR SUBMISSION

Appendix 1

- A listing of critical computerized systems used in the conduct and/or interpretation of the assigned study phase.

5. TEST AND CONTROL MATERIALS

5.1. Test and Control Material Characterization

The Sponsor will provide to the Testing Facility documentation of the identity, strength, purity, composition, and stability for the test and control article(s). A Certificate of Analysis or equivalent documentation will be provided (for active test material ingredient and preformulated material) for inclusion in the Final Report.

Documentation of identity, strength, purity, composition, and stability will be provided for control material.

The Sponsor has appropriate documentation on file concerning the method of synthesis, fabrication or derivation of the test and control article(s), and this information is available to the appropriate regulatory agencies should it be requested.

5.2. Test Material Identification

Test Material Identification

	**Test Material**
**Identification:**	scAAV9-CSA
**Alternate Identification:**	N/A
**Batch/Lot No.:**	PDTP-000102
**Expiration/Retest Date:**	N/A
**Physical Description:**	Clear, colorless liquid, no visible particulates
**Purity:**	-
**Correction Factor:**	-
**Concentration:**	2.16E+13 vg/mL
**Density:**	Supplied to the Testing Facility<br>N/A
**Storage Conditions:**	Frozen (-60 to -90°C)
**Test Material Contact:**	Heather Gray-Edwards DVM, PhD<br>Tel: (508) 856-5737<br>E-mail: Heather.GrayEdwards@umassmed.edu

- = Not applicable.

5.3. Vehicle Control Identification

Vehicle Control Identification

	**Vehicle Control**
**Identification:**	Vehicle Control
**Alternate Identification:**	VVC-321
**Lot #**	TMNP0325-B
**Retest Date:**	-

Storage Conditions:	Frozen (-60 to -90°C)

- = Not applicable.

5.4. Reserve Samples

For each batch (lot) of test and vehicle control article(s), a reserve sample will be collected and maintained under the appropriate storage conditions by the Testing Facility.

5.5. Test and Vehicle Control Material Inventory and Disposition

Records of the receipt, distribution, storage, and disposition of test article(s) (including empty containers of Sponsor-provided materials) will be maintained. All unused Sponsor supplied bulk test article(s), with the exception of reserve samples, will be returned to the Sponsor following issuance of the Draft Report unless otherwise requested (documentation will be retained in the study record). An earlier shipment of these materials may also be requested and authorized by the Study Director and Sponsor. See shipment of samples and study documentation (Attachment B) for shipping details.

The Sponsor will be contacted for proper disposition of materials (retain/ship/discard) after completion of the in-life phase of the study and following confirmation that these materials are not assigned to other studies.

5.6. Safety

A Safety Data Sheet (SDS), or equivalent documentation, will be provided by the Sponsor (if available). It is the responsibility of the Sponsor to notify the Testing Facility of any special handling requirements of the test article(s). Otherwise, routine safety precautions will be followed. Appropriate gloves, safety glasses and arm covers will be worn by individuals working with neat test article(s) or formulations.

6. DOSE FORMULATION AND ANALYSIS

6.1. Preparation of Formulations

Dose formulations will be divided into aliquots where required to allow to be dispensed on each dosing occasion.

Preparation Details

Component	Preparation	Frequency	Storage Conditions
Dose Formulations/Test Material	Diluted in the vehicle	Once	Refrigerated (2 to 8°C)

Test article mixed with vehicle to achieve desired concentrations.

Any partial vials of stock will be stored Refrigerated (2 to 8°C) following thawing and preparation. Any residual dose formulations from each dosing occasion will be stored

DRAFT, NOT FOR SUBMISSION

Appendix 1

Refrigerated (2 to 8°C) until shipped unless otherwise requested by the Study Director. Any unused, unopened vials will remain Frozen (-60 to -90°C).

6.2. Sample Collection and Analysis

Dose formulation samples (2, 0.1 ml samples) from each Group 2 and Group 3 preparation will be collected and stored Refrigerated (2 to 8°C) for possible future analysis. Additional samples may be collected and analyzed at the discretion of the Study Director.

7. TEST SYSTEM

Species/Species Family:	Rat
Strain/Breed:	Sprague Dawley®
Substrain:	Crl:CD(SD)
Animal Condition:	Naïve
Animal Supplier:	Charles River Laboratories
Animal Supplier Location:	Raleigh North Carolina, United States
Number Ordered:	98
Number of Males:	45 on study (4 alternates)
Number of Females:	45 on study (4 alternates)
Target Age at Initiation of Dosing:	8 to 10 weeks
Target Weight at the Initiation of Dosing:	120 to 370 g for males, 110 to 310 g for females
Age at Arrival:	Ordered to be 7 weeks of age at arrival
Weight at Arrival	Males will be ordered to weigh 130 to 210 g and females will be ordered to weigh 100 to 170 g at the time of surgery, as measured within 4 days of the procedure. The actual range may vary but will be documented in the data.

The actual age and weight of animals received will be listed in the Final Report.

7.1. Animal Identification

Method:	Each animal will be assigned an animal number to be used in Provantis™ and will be implanted with a microchip bearing a unique identification number. The individual animal number, implant number, and the Testing

	Facility study number will comprise a unique identification for each animal. The animal cage will be identified by the study number, animal number, group number, and sex.

7.2. Environmental Acclimation

Acclimation Duration:	At least 1 week
Details:	During this acclimation period, all animals will be observed daily for any clinical signs of disease, and all animals will be given a detailed clinical examination within 3 days of the first dose administration.

7.3. Selection, Assignment, Replacement and Disposition of Animals

Assignment and Randomization:	Animals will be randomly assigned to groups upon receipt. Males and females will be randomized separately.
	All animals with any evidence of disease or physical abnormalities will not be selected for study. Animals considered suitable for study will be randomly selected and divided into groups as described above.
Replacement:	Before the initiation of dosing, any assigned animals considered unsuitable for use in the study will be replaced by alternate animals maintained under the same environmental conditions.
	After initiation of dosing, study animals may be replaced during the replacement period with alternate animals in the event of accidental injury, non-test article-related health issues, or similar circumstances. Any data generated will not be included in the report unless deemed appropriate by the Study Director.
	Alternate animals may be used as replacements per Testing Facility SOP.
Disposition:	Extra animals obtained for this study, but not placed on study, will be either transferred to a Testing Facility stock or training colony.
	The disposition of all animals will be documented in the study records.

8. HUSBANDRY

8.1. Housing

Housing:	Socially housed, when possible (animals may be housed 2 to 3 per cage during acclimation and in-life depending on study design).
	Animals will be separated during designated procedures/activities or will be separated as required for monitoring and/or health purposes, as deemed appropriate by Study Director and/or Clinical Veterinarian.

	Housing set-up is as specified in the USDA Animal Welfare Act (9 CFR, Parts 1, 2 and 3) and as described in the Guide for the Care and Use of Laboratory Animals (NRC, current edition). Animals will be separated during designated procedures/activities or will be separated as required for monitoring and/or health purposes, as deemed appropriate by Study Director and/or Clinical Veterinarian. In addition, animals may be individually housed due to incompatible behavior with a cage mate(s).
Caging:	Solid bottom cages with nonaromatic bedding. The bedding will be from an approved supplier.

8.2. Animal Enrichment

Supplemental Enrichment:	Animal enrichment will be provided as per site standard procedures.

8.3. Environmental Conditions

Temperature and Humidity:	Temperature and humidity will be maintained according to Testing Facility SOP.
Lighting:	Fluorescent or equivalent lighting (e.g., LED) will be provided via an automatic timer for approximately 12 hours per day. On occasion, the light/dark cycle may be interrupted intermittently due to study-related activities.

8.4. Food

Diet:	The basal diet will be block Lab Diet® Certified Rodent Diet #5CR4, PMI Nutrition International, Inc.
Frequency:	Ad libitum, except during designated procedures
Analysis:	Results of analysis for nutritional components and environmental contaminants are on file at the Testing Facility.
	There are no known contaminants in the food that would interfere with this study.

8.5. Water

Type:	Tap water, treated and suitable for consumption.
Frequency:	Ad libitum via an automatic watering system (except during designated procedures). Water bottles can be provided, if required.
Analysis:	Periodic analysis of the water is performed per Testing Facility SOP and results of these analyses are on file at the Testing Facility. It is considered

that there are no known contaminants in the water that would interfere with the outcome of the study.

8.6. Veterinary Care

Veterinary care will be available throughout the course of the study and animals will be examined by the veterinary staff as warranted by clinical signs or other changes. In the event that animals show signs of illness or distress, the responsible veterinarian may make initial recommendations about treatment of the animal(s) and/or alteration of study procedures, which must be approved by the Study Director. If the condition of the animal(s) warrants therapeutic intervention or alterations in study procedures, the Sponsor will be contacted, when possible, to discuss appropriate action(s). If the condition of the animal(s) is such that emergency measures must be taken, the Study Director and/or attending veterinarian will attempt to consult with the Sponsor prior to responding to the medical crisis, but the Study Director and/or veterinarian has authority to act immediately at his/her discretion to alleviate suffering. The Sponsor will be fully informed of any such events.

9. EXPERIMENTAL DESIGN

Experimental Design

Group No.	Test Material	Dose Level (vg)	Dose Volume (mL)	Dose Concentration (vg/mL)	Main Study		Necropsy Day 29 (+/-1) M/F	
					No. of Males	No. of Females		Necropsy Day 91 (+/-2) M/F
1	Vehicle Control	0	0.1	0	10	10	5/5	5/5
2	scAAV9 -CSA	2.16×10^{11}	0.1	2.16×10^{12}	10	10	-	10/10
3	scAAV9 -CSA	6.55×10^{11}	0.1	6.55×10^{12}	10	10	-	10/10
4	scAAV9 -CSA	2.16×10^{12}	0.1	2.16×10^{13}	15	15	5/5	10/10

9.1. Administration/Application of Test and Vehicle Control Material(s)

Route of Administration:	Intrathecal Injection
Treatment Frequency/Duration:	Once on Day 1
Details:	Post-acquisition, test article will be maintained on wet ice. Prior to dosing the vehicle or test article will acclimate to ambient. An equipment list containing information regarding the dosing apparatus will be maintained in the study records.

Appendix 1

10. SURGICAL ANIMAL PREPARATION

10.1. Pre-operative Procedures

Details: Anesthesia will be induced and maintained as indicated in Testing Facility SOP.

10.2. Surgical Procedure

The procedures outlined below are intended to serve as guidance. Alterations in the surgical procedures may occur as necessary and will be communicated to the Study Director. These alterations will be documented in the study data and will not be considered a protocol deviation.

A 27-gauge custom LASI needle (or equivalent) and an appropriately sized gas tight Hamilton syringe will be used to administer test article(s). Prior to dosing, the dosing system will be primed with control article or test article, as applicable. After the system is primed, the appropriate amount of test material will be drawn up. The system will not be re-primed between animals. A new needle will be used for each group or as needed. The priming steps will be repeated if a new needle is used. If a flush is needed for dosing, IT control article will be used. This will be documented in the study data. The injection site location will be documented in the study data. The duration of dose is 60 seconds +/- 5 seconds.

10.3. Postoperative Procedures

Details: Postoperative procedures will be conducted in accordance with Testing Facility SOP.

11. IN-LIFE PROCEDURES, OBSERVATIONS, AND MEASUREMENTS

General In-life Assessments – Main Study Animals

Parameter	Population(s)	Target Frequency (minimum required)	Comments
Mortality/Cageside Observations	All surviving animals	At least twice daily (morning and afternoon) beginning upon arrival through termination/release.[a,b]	Animals will be observed within their cage unless necessary for identification or confirmation of possible findings.<br><br>Animals will be observed for morbidity, mortality, injury, and availability of food and water. Any animals in poor health will be identified for further monitoring and possible euthanasia.
Post Dose Observations	All Main Study animals	Approximately 4 and 24 hours post dose.[c]	Animals will be observed within their cage unless necessary for identification or confirmation of possible findings.

DRAFT, NOT FOR SUBMISSION

Appendix 1

Parameter	Population(s)	Target Frequency (minimum required)	Comments
Detailed Clinical Observations	All Main Study animals	Once weekly; from at least Week -1 and throughout the study.[c]	Animals are removed from the cage.<br><br>Animals will be observed within their cage unless necessary for identification or confirmation of possible findings.<br><br>Pretest interval will be conducted on all animals received/transferred.
Individual Body Weights	All Main Study animals	Within 3 days of arrival, and Day -1, and once weekly during the study.	Not collected from animals found dead.
Food Consumption	All Main Study animals	Once weekly[d]	Qualitative assessment will be conducted as a part of the twice daily cageside observations. Any findings of inappetence will be recorded as an unscheduled clinical observation.
Ophthalmic Examinations	All Main Study animals	Pretest (all animals including source) and all survivors examined again prior to terminal and recovery necropsy.	See details in section below.
Neurobehavioral Evaluations	All Main Study animals	Day -1, and prior to terminal necropsy. Recovery animals will also be evaluated approximately 4 weeks after the end of the Test Material administration. The same animals designated for behavioral testing will be tested at all times.	See details in section below.

[a] To include alternate animals until released from study.
[b] Except on days of receipt and necropsy where frequency will be at least once daily.
[c] For observations that cannot be attributed to an individual animal due to social housing (e.g., watery feces), the observation will be noted to each animal in the socialized group.
[d] For observations of reduced appetite that cannot be attributed to an individual animal due to social housing, the observations will be noted for each animal in the socialized group.

11.1. Ophthalmic Exams

Details: The ophthalmoscopic examinations will be conducted by a staff ophthalmologist via indirect ophthalmoscopy. Digital images for illustrative purposes may be taken. These images will not be used for data generation or interpretation and will not be archived or included in the Final Report.

Slit-lamp exams may be conducted at the discretion of the ophthalmologist.

11.1. Neurobehavioral Evaluations

Neurobehavioral evaluations will be conducted on all animals without knowledge on the part of the testers of the treatment groups (blinded evaluations). PPE need not be changed between groups during dose administration when animals are dosed in random order due to blinding. Each animal will be observed for 2 minutes in an opaque open-field observation box. Parameters will be evaluated per Testing Facility SOPs and study specific procedures. The following evaluations will be conducted:

Parameter Observed	Type of Data	Rating Scale or Unit of Measurement
Posture/Body carriage	Categorical	-2 to 0
Convulsions	Categorical	0 to 4
Stereotypy	Categorical	0 to 4
Tremor	Categorical	0 to 3
Palpebral closure/Ptosis	Categorical	0 to 2
Ease of removal	Categorical	-1 to 2
Handling reactivity	Categorical	-1 to 2
Rearing	Continuous	Counts
Arousal/Alertness	Categorical	-2 to 2
Gait/Mobility	Categorical	0 to 3
Vocalizations	Categorical	0 to 1
Respiration	Categorical	-1 to 2
Defecation	Categorical	-1 to 1
Appearance	Categorical	0 to 2
Lacrimation	Categorical	0 to 2
Salivation	Categorical	0 to 2
Exophthalmus	Categorical	0 to 1
Erected fur	Categorical	0 to 1
Touch response/Tactile reflex	Categorical	-1 to 1
Startle response	Categorical	-1 to 1
Thermal response	Continuous	Time in seconds
Pupil response	Categorical	0 to 2
Body temperature	Continuous	℃
Body tone	Categorical	-1 to 1
Air righting reflex	Categorical	0 to 2
Grip strength (forelimb and hindlimb)	Continuous	kg

Appendix 1

12. CLINICAL PATHOLOGY

12.1. Sample Collection

Clinical Pathology Sample Collection

Group Nos.	Time Point(s)	Recollection of Sample Acceptable[a]	Hematology	Coagulation	Clinical Chemistry	Urinalysis
All animals	Prior to necropsy on days 29 (+/-1) and 91 (+/-2)	X	X	X	X	X
**Unscheduled Euthanasia (when possible)**			See Section 14.2 of this protocol.			
**Fasting:**			Animals will not be fasted.			
**Method/Comments:**			Cardiac puncture after carbon dioxide inhalation			Urine will be collected from the animals for a period of at least 12 hours.
**Target Volume[a]:**			1 mL	1.2 mL	1.0 mL	Maximum obtainable
**Anticoagulant:**			K_2EDTA	Sodium Citrate	Serum Gel Separator	-
**Special Requirements:**			-	-	-	X
**Processing:**			None	Plasma	Serum	-

X = Sample to be collected; - = Not applicable; hr = Hour; pre = Predose; post= Postdose

[a] Additional samples may be obtained as per procedure if permissible sampling Frequency and volume are not exceeded.

[b] Add footnote.

Blood Sample Special Instructions: Terminal – Cardiac puncture after carbon dioxide inhalation.

Urine Sample Collection: Urine will be collected on wet ice and will be centrifuged at 4°C and the supernatant retained for analysis. The supernatant will be placed into 2 freezer vials of 250 µL each and stored at -60°C to -90°C.

Urine Sample Collection: Urine will be collected from the animals for a period of at least 12 hours.

Special Requirements: Due to the sample volume size and the number of tests indicated, it is likely that not all tests will be completed for all animals. The study data and Final Report will indicate tests not conducted as insufficient sample where appropriate.

Appendix 1

The following clinical pathology tests will be conducted on available samples, as survival allows, and the data will be interpreted by a Clinical Pathologist.

12.2. Hematology Evaluation

Hematology Parameters[a]

Red blood cell count<br>Hemoglobin concentration<br>Hematocrit<br>Mean corpuscular volume<br>Red blood cell distribution width<br>Mean corpuscular hemoglobin concentration<br>Mean corpuscular hemoglobin<br>Reticulocyte count (absolute and percent)<br>Platelet count	White blood cell count<br>Neutrophil count (absolute and percent)<br>Lymphocyte count (absolute and percent)<br>Monocyte count (absolute and percent)<br>Eosinophil count (absolute and percent)<br>Basophil count (absolute and percent)<br>Other cells (as appropriate)<br>Blood smear (preserve and stain[b])

[a] Analysis performed using Sysmex XN-1000V method.

[b] A blood smear will be prepared from each hematology sample. Blood smear review may be performed on select animals per Testing Facility SOP.

12.3. Coagulation Evaluation

Coagulation Parameters

Prothrombin time<br>Activated partial thromboplastin time	Fibrinogen<br>Sample quality

12.4. Clinical Chemistry Evaluation

Clinical Chemistry Parameters

Alkaline phosphatase<br>Total bilirubin[a]<br>Aspartate aminotransferase<br>Alanine aminotransferase<br>Gamma glutamyl transferase<br>Sorbitol dehydrogenase<br>Urea nitrogen<br>Creatinine<br>Total protein	Albumin<br>Globulin (calculated)<br>Albumin/globulin ratio (calculated)<br>Glucose<br>Total cholesterol<br>Triglycerides<br>Electrolytes (sodium, potassium, chloride)<br>Calcium<br>Phosphorus<br>Sample quality

[a] When total bilirubin is >0.5 mg/dL, direct bilirubin will also be measured, and indirect bilirubin will be calculated.

DRAFT, NOT FOR SUBMISSION

Appendix 1

12.5. Urinalysis Evaluation

Urinalysis Parameters

Volume<br>Color<br>Appearance<br>Specific gravity<br>pH<br>Protein	Glucose<br>Bilirubin<br>Ketones<br>Blood<br>Urobilinogen<br>Leukocyte esterase<br>Nitrites

13. BIODISTRIBUTION EVALUATION

13.1. Biodistribution Sample Collection

Biodistribution Sample Collection

Group Nos.	Target Postdose Collection Intervals – Days 29 and 91
	**Terminal necropsy**
All	X

X = Sample to be collected; - = Not applicable; min = Minute; hr/hrs = Hour/hours.

13.2. Biodistribution Sample Processing

Collection Method:	Cardiac puncture after carbon dioxide inhalation
Target Volume:	1 mL
Anticoagulant:	K_2EDTA
Fasting:	The animals will not be fasted before blood collection unless concurrent with fasting for other procedures.
Whole Blood Storage:	Wet ice or ice block
Processing:	Samples will be processed, split into two aliquots, snap frozen, and shipped to the Sponsor for biodistribution analysis (rt-qPCR and qPCR analysis).
Additional Container Label Requirements:	Study number, animal number, matrix of sample, interval and timepoint, analysis type, and aliquot number (if applicable).
Final Storage Temperature:	Frozen (-60 to -90°C)<br>Processed samples will be placed on dry ice or in Frozen (-60 to -90°C) storage 4 hours of collection.

13.3. Biodistribution Sample Shipment

Bioanalytical Sample Shipping Contact:	All samples to be analyzed will be shipped to the designated Test Site, see Attachment B for shipping details.

13.4. Biodistribution Sample Analysis

Analysis Performed By:	The samples will be analyzed for AAV9- scAAV9-CSA All analytical work will be conducted by a Sponsor-contracted laboratory, using a Sponsor-supplied analytical method an analytical method developed and qualified by that laboratory.
Incurred Sample Reanalysis:	Incurred sample reanalysis (ISR) will be conducted for this study in accordance with Test Site SOP's.
Test Site Regulatory Requirements:	The work performed in conjunction with this study will not be conducted in compliance with GLPs. A Final Report will be prepared and submitted to Testing Facility for inclusion as an appendix in the main study Final Report.

14. TERMINAL PROCEDURES

Terminal procedures are summarized in the following tables:

Unscheduled Deaths

Unscheduled Euthanasia	Necropsy Procedures				Histology Processing	Microscopic Evaluation
	Necropsy	Organ Weights	Tissue Collection			
			Fixed	Frozen		
Found Dead or Perfusion not possible Refrigerated	X	-	Full List[a]	-	Full List[a]	Full List[a]
Perfusion possible Non-Refrigerated	X	Full List[a]	Full List[a]	Full List[a]	Full List[a]	Full List[a]

Main Study Animals

Group No.	Scheduled Euthanasia Day	Necropsy Procedures				Histology Processing	Microscopic Evaluation
		Necropsy	Organ Weights	Tissue Collection			
				Fixed	Frozen		
All animals	29 **(+/-1)** and 91**(+/-2)**	X	Full List[a]	Full List[a]	Full List[a]	Full List[a]	Full List[a]

Appendix 1

Group No.	Scheduled Euthanasia Day	Necropsy Procedures				Histology Processing	Microscopic Evaluation
		Necropsy	Organ Weights	Tissue Collection			
				Fixed	Frozen		

X = Procedure to be conducted; - = Not applicable.
'Histology Processing' = embedded in paraffin, sectioned, mounted on glass slides, and stained with hematoxylin and eosin.
a See Tissue Weighing, Collection, Processing and Evaluation table, (Attachment A) for list of tissues applicable to each procedure.

14.1. Method of Euthanasia

Euthanasia will be by carbon dioxide inhalation followed by a Testing Facility SOP approved method to ensure death, e.g., exsanguination or perfusion.

14.2. Unscheduled Euthanasia

Animals will be subject to Testing Facility SOP criteria and procedures for early euthanasia or when found dead. If possible, the samples below will be collected from animals euthanized following veterinary consultation. A veterinary consultation is not required if the samples are collected following anesthesia or euthanasia. Blood collection methods utilized for animals euthanized early may include suitable methods other than those presented in the respective blood collection section(s) of this Protocol.

Sample Type	Groups	Target Volume	Anticoagulant
Hematology	All	1 mL	K_2EDTA
Coagulation		1.2 mL	Sodium Citrate
Clinical Chemistry		1.0 mL	Serum Gel Separator
Biodistribution	All	1.0 mL	K_2EDTA

Necropsy examinations will be performed 7 days a week. Animals that are found dead or euthanized early after regular working hours will be refrigerated and necropsies performed at the start of the next day.

14.3. Scheduled Euthanasia

Main Study animals surviving until scheduled euthanasia will be euthanized by the methods described above.

14.4. Necropsy

Animals as detailed in the Terminal Procedures table will be subjected to a complete necropsy examination, which will include evaluation of the carcass and musculoskeletal system; all external surfaces and orifices; cranial cavity and external surfaces of the brain; and thoracic, abdominal, and pelvic cavities with their associated organs and tissues. Veterinary pathologist or other suitably qualified personnel will be available for consultation during scheduled necropsy.

DRAFT, NOT FOR SUBMISSION

All animals will be examined carefully for external abnormalities including palpable masses.

Following euthanasia, animals will be perfused with chilled 0.9% sterile saline (for at least 5 minutes or until fluid runs clear). Effort will be made, if possible, to perfuse unscheduled euthanasia animals during standard business hours. Animals found dead or euthanized outside of standard business hours will not be perfused.

Images may be generated for illustration of or consultation on gross observations. These images will not be used for data generation or interpretation and will not be archived or included in the Final Report.

14.5. Organ Weights

Body weight and the organ weights identified in the Tissue Weighing, Collection, Processing and Evaluation table (Attachment A) will be recorded for all animals at the scheduled necropsies, and appropriate organ weight ratios will be calculated (relative to body and brain weights). Paired organs will be weighed together. Organ weights will be collected per Testing Facility SOP.

14.6. Tissue Collection and Preservation

Representative samples of tissues will be collected and preserved in 10% neutral buffered formalin, as detailed in the Terminal Procedures table and Tissue Weighing, Collection, Processing, and Evaluation table (Attachment A). Additional tissue samples may be collected to elucidate abnormal findings.

For tissues that are too small to accommodate all requested samples, sample collection priority will be as follows (samples not collected due to limited size will be noted in the study record, but not considered a protocol deviation):

Histology>qPCR

14.6.1. Brain Tissue Collection Details

The entire brain will be sliced into 3 mm coronal slabs using a brain matrix. Slabs will be arranged in sequential order (rostral to caudal) and photographed (caudal surface). If additional sampling from any slab is required, images will be taken both before and after any frozen samples are collected. For unscheduled euthanasia animals that do not require frozen sample collection at necropsy, brains may be fixed whole, then slabbed and sections designated for histology processing at the time of fixed tissue trimming and trimmed following the standard CRL histology blocking scheme.

Slabs will be designated for collection and processing as follows:

Each brain slab will be bisected into left and right hemispheres along the sagittal midline. The left hemislabs will be frozen on dry ice, and wrapped in aluminum foil. Then the slabs will be stored in pre- cooled 5 ml tubes and stored frozen (-60 to -90°C) until shipment to the sponsor.

Appendix 1

The right hemislabs will be preserved in 10% NBF for histopathology

For fixed brain samples, the designated slab hemislab will be submitted rostral side down in a standard cassettes, to maintain orientation (labeled with the slab number, left/right). Select fixed hemi-slabs will be processed for microscopic evaluation following the standard CRL blocking scheme.

14.7. Biodistribution Frozen Tissue Collection

Care will be given to ensure that cross contamination between tissues does not occur. Gloves and disposable collection tools will be changed between collection and dissection of each tissue for qPCR biodistribution analysis. The cutting board and non-disposable instruments will be wiped down with a 10% bleach solution, rinsed with water, followed by a wipe down of 70% or greater ethanol between each of the specified organs; sterile trays may be used. Samples for qPCR will not be collected for animals found dead or euthanized in extremis where perfusion is not possible.

Intervals and Number of Animals:	All survivors at necropsy (Day 29) and necropsy (Day 91) and any animal euthanized with perfusion possible.
Tissues to Collect:	See Attachment A for list of tissues applicable to each procedure.
Sample Collection:	A portion of each required tissue, without sacrificing what is needed for histopathology, will be collected for analysis as follows:<br>Individual sample size: [2-3 1-2 mm pieces]. Individual sample weight will not be recorded.<br>1 DRG per level.<br>All samples will be placed into pre-labeled polypropylene vials, tightly capped, and frozen in liquid nitrogen. Care will be taken to minimize the time between sample collection and freezing. Samples may be held on dry ice until final storage.
Storage Conditions:	Frozen (-60° to -90°C)
Label Requirements:	Testing Facility study number, Sponsor study number (if applicable), animal number, group number, nominal study day, nominal time point (if applicable), analysis type (e.g., Biodistribution), tissue type (e.g., ORGANS), and if possible, the date of collection.
Sample Shipment:	Samples will be shipped to the Test Site, see Attachment B for shipping details.

DRAFT, NOT FOR SUBMISSION

14.7.1. Biodistribution Frozen Tissue Sample Analysis

Analysis Performed By:	Samples will be analyzed for test article concentration. All analytical work will be conducted by a Sponsor-contracted laboratory to be added by amendment.
Test Site Regulatory Requirements:	The work performed in conjunction with this study will not be conducted in compliance with GLPs. A Final Report will be prepared and submitted to Testing Facility for inclusion as an appendix in the main study Final Report.

15. HISTOLOGY AND MICROSCOPIC EVALUATION

15.1. Histology

Histology will be conducted per Testing Facility SOPs.

15.2. Microscopic Evaluation

Tissues as detailed in the Tissue Weighing, Collection, Processing and Evaluation table (Attachment A) will be evaluated histopathologically by light microscopy) by a board-certified veterinary as identified in the Responsible Personnel section of this Protocol.

Special stains may be used at the discretion of the pathologist to further characterize lesions and changes identified during routine evaluation of individual animals. Any special stains will be documented in the individual animal data. Any additional stains or evaluations, if deemed necessary by the pathologist, may be added by Protocol Amendment following discussion with the Study Director and in consultation with the Sponsor. Efforts will be made to evaluate all Protocol-required tissues microscopically; however, it is not always feasible for every Protocol-required tissue to be present on every slide. Protocol required tissues that are not examined will be documented in the histopathology data and the impact of these missing tissues on the study will be documented in the Pathology Report.

Images may be generated for illustration of or consultation on histological observations. These images will not be used for data generation or interpretation and will not be archived or included in the Final Report.

Photomicrographs of representative microscopic lesions will be taken, archived in the study data, and all or representative images will be included in the Final Report.

In the unlikely event that a Testing Facility pathologist cannot be scheduled for the pathology evaluation due to logistics/timing, the pathology evaluation may be outsourced (by protocol amendment) to an external ACVP-certified veterinary pathologist.

The appropriate Charles River Laboratories Pathology Associates Test Site will be contacted for the slide shipping address.

Appendix 1

16. STATISTICAL ANALYSIS

Statistical analysis will not be performed; however, descriptive statistics will be calculated. For continuous endpoints, descriptive statistics will consist of means, standard deviations, and group size for each treatment group and time period. For categorical endpoints, descriptive statistics will consist either of medians or incident counts for each treatment group and time period.

Type of Analysis: Descriptive Statistics

Endpoints:

- Body Weight and Body Weight Change
- Food Consumption
- Neurobehavioral Evaluations
- Hematology
- Coagulation
- Clinical Chemistry
- Organ Weights
- Absolute Weights
- Relative to Body and Brain Weights

16.1. Statistical Comparisons

Control Group	Comparison Group(s)
1	2, 3, 4

17. COMPUTERIZED SYSTEMS

The following critical computerized systems may be used in the study. The actual critical computerized systems used will be specified in the Final Report. The implemented paper and electronic systems are fit for the integrity of the study data.

Critical Computerized Systems

System Name	Description
eInfotree	Excel Module for data collection with 21 CFR Part 11 compliance requirements, security, audit trail, and electronic signatures.
ExyLIMS	A comprehensive laboratory information management system used to manage data, including but not limited to instrumentation, test article(s), standards, and samples.
In-house reporting software Nevis 2012 (using SAS)	In-life; clinical pathology; postmortem
Logbook	Electronic notebook and data collection system for veterinary communications, observations, and treatments.
M-Files®	Reporting and collection of 21 CFR Part 11 compliant signature
Provantis®	In-life; clinical pathology; postmortem; test article(s)receipt, accountability, and/or formulation activities

DRAFT, NOT FOR SUBMISSION

Appendix 1

System Name	Description
SAS®	An integrated system of software products that enables a user to perform data entry, retrieval, data management, reporting, graphics, statistical analysis, and applications development.
Siemens Monitoring System	Collects and report temperature and humidity data and controls some HVAC systems.

Data for parameters not required by Protocol, which are automatically generated by analytical devices used will be retained on file but not reported. Statistical analysis results that are generated by the program but are not required by Protocol and/or are not scientifically relevant will be retained on file but will not be included in the tabulations.

18. REGULATORY COMPLIANCE

The study will be conducted in accordance with the U.S. Department of Health and Human Services, Food and Drug Administration, United States Code of Federal Regulations, Title 21, Part 58: Good Laboratory Practice for Nonclinical Laboratory Studies and as accepted by Regulatory Authorities throughout the European Union (OECD Principles of Good Laboratory Practice), Japan (MHLW), and other countries that are signatories to the OECD Mutual Acceptance of Data Agreement.

Exceptions to GLPs include the following study elements:

- Characterization of the test and control materials will be/were performed by the Sponsor or Sponsor subcontractor according to established SOPs, controls, and approved test methodologies to ensure integrity and validity of the results generated; these analyses will not be/were not conducted in compliance with the GLP regulations.
- The Biodistribution assays will not be performed within the scope of regulations governing the conduct of nonclinical laboratory studies and is not intended to comply with such regulations.

19. QUALITY ASSURANCE

19.1. Testing Facility

The Testing Facility Quality Assurance Unit (QAU) will monitor the study to assure the facilities, equipment, personnel, methods, practices, records, and controls are in conformance with Good Laboratory Practice regulations. The QAU will review the Protocol, conduct inspections at intervals adequate to assure the integrity of the study, and audit the Final Report to assure that it accurately describes the methods and standard operating procedures and that the reported results accurately reflect the raw data of the study.

20. AMENDMENTS AND DEVIATIONS

Changes to the approved Protocol shall be made in the form of an amendment, which will be signed and dated by the Study Director. Every reasonable effort will be made to discuss any

necessary Protocol changes in advance with the Sponsor. The Study Director will notify the Sponsor of deviations that may result in a significant impact on the study as soon as possible.

21. RETENTION AND DISPOSITION OF RECORDS, SAMPLES, AND SPECIMENS

All study-specific raw data, electronic data, documentation, Protocol, retained samples and specimens, and Interim (if applicable) and Final Reports will be archived by no later than the date of Final Report issue. All materials generated by Charles River Laboratories from this study will be transferred to a Charles River Laboratories archive. At least 1 year after issue of the Draft Report, the Sponsor will be contacted.

Samples for clinical pathology evaluations are discarded per Testing Facility SOP unless otherwise indicated in the table below.

Disposition of residual/retained analytical samples will be as described in the table below.

Disposition of Residual/Retained Samples

Sample Type	Disposition	Schedule
Dose Formulation Preparation Sample	Ship to Sponsor	The disposition of the samples will occur as per Protocol if definitive results are within acceptance criteria and have been provided to the Study Director. If results are out of specification, the Study Director will need to provide approval for disposition.
Residual Dosing Formulation	Ship to Sponsor	Per disposition indicated in the Preparation of Formulations section.
ADA/Biodistribution	Ship to Sponsor	Samples will be maintained for a minimum of 6 months following issuance of the Draft Report or at an alternate time point prior to finalization as requested by the Study Director.
Pathology slides	Archive	Return to Testing Facility after Issuance of the Draft Report

Records to be maintained will include, but will not be limited to, documentation and data for the following:

- Deviations, Protocol amendments, and Protocol
- Study schedule
- Study-related correspondence
- Test system receipt, health, and husbandry
- Test and control material receipt, identification, preparation, and analysis
- In-life measurements and observations
- Clinical pathology sample collection and evaluation
- Bioanalytical sample collection and evaluation
- Gross and microscopic observations and related data
- Organ weight measurements
- Statistical analysis results

22. STUDY CLASSIFICATION

Submission Study Category:	Toxicology
Submission Study Type:	Single Dose Toxcitiy
Study Design:	Parallel
Primary Treatment CAS Registry Number:	Not Applicable
Primary Treatment Unique Ingredient ID:	Not Applicable
Pharmacologic Class:	Biologic – AAV vector
Administration Dose Form:	Solution

23. REPORTING

An audited Interim Draft report containing the results through the Day 29 necropsy timepoint will be prepared and issued to the Sponsor for regulatory filing.

The Sponsor will receive an electronic version of the Draft and Final Reports, provided in Adobe Acrobat PDF format (hyperlinked and searchable at final). The PDF document will be created from native electronic files to the extent possible, including text and tables generated by the Testing Facility. Report components not available in native electronic files and/or original signature pages will be scanned and converted to PDF image files for incorporation.

Reports should be finalized within 6 months of issue of the Draft Report. If the Sponsor has not provided comments to the report within 6 months of draft issue, the report will be finalized by the Testing Facility unless other arrangements are made by the Sponsor.

Appendix 1

23.1. SEND Datasets

SEND datasets will be generated and provided outside the context of the GLP Report. These datasets will not be subject to QA Audit nor will they be used as the basis for the Study Director interpretation of the study results. SEND datasets will be provided for the Report based on regulatory submission date. The Sponsor is expected to provide submission dates.

When work in support of this study is conducted at a Test Site (i.e., Bioanalysis, TK modeling, etc.), an electronic version of all data should be provided to Testing Facility.

24. JUSTIFICATIONS AND GUIDELINES

24.1. Justification of Test System and Number of Animals

At this time, studies in laboratory animals provide the best available basis for extrapolation to humans and are required to support regulatory submissions. Acceptable models that do not use live animals currently do not exist.

The rat was chosen as the animal model for this study as it is an accepted rodent species for nonclinical toxicity testing by regulatory agencies.

The total number of animals to be used in this study is considered to be the minimum required to properly characterize the effects of the test article(s). This study has been designed such that it does not require an unnecessary number of animals to accomplish its objectives. Details on the group distribution, number of test groups, and number of animals per group are included in the Experimental Design section.

24.2. Justification of Route and Dose Levels

Intrathecal injection is one of the intended routes of administration of potential routes of human exposure to this test article(s).

The dose levels were selected based on information provided by the Sponsor provided details, references, and in an attempt to produce graded responses to the test article item substance(s). The high-dose level should produce some toxic effects, but not excessive lethality that would prevent meaningful evaluation. The mid-dose level is expected to produce minimal to moderate toxic effects. Intermediate dose levels are expected to produce minimal to moderate toxic effects. The low-dose level should produce no observable indications of toxicity.

24.3. Guidelines for Study Design

The design of this study was based on the study objective(s) and the overall product development strategy for the test article(s).

- FDA Guidance for Industry. Preclinical Assessment of Investigational Cellular and Gene Therapy Products.

- FDA Guidance for Industry. Human Gene Therapy for Rare Diseases.
- FDA Draft Guidance for Industry. Human Gene Therapy for Neurodegenerative Diseases.

25. ANIMAL WELFARE

This study will comply with all applicable sections of the Final Rules of the Animal Welfare Act regulations (Code of Federal Regulations, Title 9), the *Public Health Service Policy on Humane Care and Use of Laboratory Animals* from the Office of Laboratory Animal Welfare (OLAW, current edition), and the *Guide for the Care and Use of Laboratory Animals* (NRC, current edition). The Protocol and any amendments or procedures involving the care or use of animals in this study will be reviewed and approved by the Testing Facility Institutional Animal Care and Use Committee before the initiation of such procedures.

If an animal is determined to be in overt pain/distress or appears moribund and is beyond the point where recovery appears reasonable, the animal will be euthanized for humane reasons in accordance with the *American Veterinary Medical Association (AVMA) Guidelines on Euthanasia* (AVMA, current edition) and with the procedures outlined in the Protocol.

By approving this Protocol, the Sponsor affirms that there are no acceptable non-animal alternatives for this study, that this study is required by a relevant government regulatory agency(ies) and that it does not unnecessarily duplicate any previous experiments.

25.1. Institutional Animal Care and Use Committee Approval

The Protocol and any amendment(s) or procedures involving the care and use of animals in this study will be reviewed and approved by Charles River Laboratories, Mattawan Institutional Animal Care and Use Committee (IACUC) before conduct. During the study, the care and use of animals will be conducted with guidance from the guidelines of the USA National Research Council.

26. REFERENCES

American Veterinary Medical Association. *AVMA Guidelines on Euthanasia*. Current edition.

National Research Council. *Guide for the Care and Use of Laboratory Animals*. Current edition.

Office of Laboratory Animal Welfare. *Public Health Services Policy on Humane Care and Use of Laboratory Animals*. Bethesda, MD: National Institutes of Health. Current edition.

Appendix 1

ATTACHMENT A

Tissue Weighing, Collection, Processing and Evaluation Table

Organ	Organ Weight	Fixed Tissue Collection	Histology Processing	Microscopic Evaluation	Biodistribution Collection and Analysis
Animal ID	-	X	-	-	-
Artery, aorta	-	X	-	-	-
Body cavity, nasal	-	X	-	-	-
Bone marrow smear	-	X[a]	-	-	-
Bone marrow, sternum	-	X	-	-	-
Bone, femur	-	X (1)	-	-	-
Bone, sternum	-	X	-	-	-
Brain	X	X	X	X	X
Dorsal Root Ganglia, cervical	-	X(2)	X(2)	X(2)	X
Dorsal Root Ganglia, thoracic	-	X(2)	X(2)	X(2)	X
Dorsal Root Ganglia, lumbar	-	X(2)	X(2)	X(2)	X
Epididymis	X (2)	X (2)[b]	-	-	-
Esophagus	-	X	-	-	-
Eye	-	X (2)[b]	-	-	-
Gland, adrenal	X (2)	X (1)	X (1)	X (1)	X (1)
Gland, clitoral	-	X (2)	-	-	-
Gland, Harderian	-	X (2)	-	-	-
Gland, lacrimal	-	X (2)	-	-	-
Gland, mammary	-	X	-	-	-
Gland, parathyroid	-	X (2)	-	-	-
Gland, pituitary	X	X	-	-	-
Gland, preputial	-	X (2)	-	-	-
Gland, prostate	X	X	-	-	-
Gland, salivary, parotid	-	X (2)	-	-	-
Gland, salivary, sublingual	-	X (2)	-	-	-
Gland, salivary, mandibular	-	X (2)	-	-	-
Gland, seminal vesicle	-	X (2)	-	-	-
Gland, thyroid	-	X (2)	-	-	-
Gland, thyroid/parathyroid	X (2)	X (2)	-	-	-
Gland, Zymbal's	-	X (2)	-	-	-
Gut-associated lymphoid tissue	-	X	-	-	-
Heart	X	X	X	X	X
Joint, femorotibial	-	X (1)	-	-	-
Kidney	X (2)	X (2)	X (2)	X (2)	X (1)
Large intestine, cecum	-	X	-	-	-
Large intestine, colon	-	X	X	X	X

DRAFT, NOT FOR SUBMISSION

Appendix 1

Organ	Organ Weight	Fixed Tissue Collection	Histology Processing	Microscopic Evaluation	Biodistribution Collection and Analysis
Large intestine, rectum	-	X	-	-	-
Larynx	-	X	-	-	-
Liver	X	X	X	X	X
Lung	-	X	X	X	X
Lymph node(s) draining administration site(s): Iliac draining lymph node	-	X (1)	X (1)	X (1)	X (1)
Lymph node, mandibular	-	X (2)	-	-	-
Lymph node, mesenteric	-	X	-	-	-
Muscle, skeletal	-	X (2)	-	-	-
Nerve, optic	-	X (2)[b]	-	-	-
Nerve, sciatic	-	X (2)	X (1)	X (1)	X (1)
Nerve, tibial	-	X (2)	-	-	-
Ovary	X (2)	X (1)	X (1)	X (1)	X (1)
Oviduct	-	X (2)	-	-	-
Pancreas	-	X	-	-	-
Skin	-	X	-	-	-
Small intestine, duodenum	-	X	-	-	-
Small intestine, ileum	-	X	-	-	-
Small intestine, jejunum	-	X	-	-	-
Spinal cord, cervical	-	X	X	X	X
Spinal cord, thoracic	-	X	X	X	X
Spinal cord, lumbar	-	X	X	X	X
Spinal cord, intrathecal injection site	-	X	X	X	X
Spleen	X	X	X	X	X
Stomach	-	X	X	X	X
Testis	X (2)	X (Right)	X (Right)	X (Right)	X (1) (Left)
Thymus	X	X	-	-	-
Tongue	-	X	-	-	-
Trachea	-	X	-	-	-
Ureter	-	X (2)	-	-	-
Urinary bladder	-	X	-	-	-
Uterus/Cervix	X	X	-	-	-
Vagina	-	X	-	-	-
Macroscopic abnormalities[e]	-	X	X	X	-

Appendix 1

ATTACHMENT B

Samples will be shipped on Monday through Wednesday for next day delivery for domestic shipments and expedited delivery for international shipments using the Conditions for Shipment(s) listed below.

Shipment of Samples and Study Records

Sample Matrix	Sample Type	Aliquot of Sample	Proposed Shipment Timeline	Conditions for Shipment	Recipient/Address
Vehicle/Test article	Dose Formulations	All	After collection	cold packs	To be added by amendment
Plasma	Biodistribution	All	After Day 29 and collection And after final necropsy collection	on dry ice	To be added by amendment
Not applicable	Pathology slides	Not applicable	After all slides are processed and ready for shipment	Ambient	To be added by amendment
Not applicable	Return of Pathology slides	Not applicable	Ship after Issuance of the Draft Report	Ambient	Charles River Laboratories<br>ATTN: Path Services, Room LS-30<br>54943 North Main Street<br>Mattawan, MI 49071<br>Tel: 269-668-3336 ext. 1643<br>E-mail:<br>MWN-HistologySupport@crl.com

Appendix 1

SPONSOR APPROVAL

The Protocol was approved by the Sponsor by e-mail on the date designated below. The correspondence giving approval will be archived, as appropriate with other Sponsor communications.

11 Jul 2025

Date of Sponsor Approval

Appendix 1

TESTING FACILITY APPROVAL

All electronic signatures appear at the end of the document upon finalization.

DRAFT, NOT FOR SUBMISSION

Appendix 1

SIGNATURE(S) FOR DOCUMENT: 32025165 - Protocol

**Management Approval-GLP:**	I approve the Study Director identified in this document and management's responsibility to the study as defined by the relevant GLP.
Name:	**Wilson, Scott**
	11-Jul-2025 15:48:33 (UTC+00:00)
Electronically Signed in	Timestamp

**Study Director Approval:**	I approve this document.
Name:	**Gunter, Barak**
	11-Jul-2025 16:19:10 (UTC+00:00)
Electronically Signed in	Timestamp

Appendix 2

Cover Page for R&D CoA – scAAV9-CB-ERCC8, Lot: PDTP-000102 RDCOA-000136

Document Details	
Document Name	R&D CoA – scAAV9-CB-ERCC8, Lot: PDTP-000102
Document Number	RDCOA-000136
Version Status	Effective
Effective Date Time	11 Jul 2025 12:52:27 (EDT)
Document Version	1

Effective

Document Reviewers			
**First Name**	**Last Name**	**Email**	**Date/Time Reviewed**
Cara	Allen	cara.allen@andelynbiosciences.com	11 Jul 2025 12:13:41 (EDT)

Document Approvers			
**First Name**	**Last Name**	**Email**	**Date/Time Approved**

Appendix 2

Marissa	Evans	marissa.evans@andelynbiosciences.com	11 Jul 2025 12:50:49 (EDT)

Effective

Appendix 2

Andelyn BIOSCIENCES | Advancing Discoveries. Manufacturing Cures.

R&D CERTIFICATE OF ANALYSIS			Page 1 of 3
**R&D COA ID #:**	**RDCOA-000136**	**Version:**	**1**
**Product Name:**	scAAV9-CB-ERCC8		
**Product Description:**	The biological product scAAV9-CB-ERCC8 is a self-complimentary (sc) non-replicating recombinant adeno-associated virus 9 (AAV9) for non-clinical research use.		
**Lot Number:**	PDTP-000102		
**Concentration:**	2.16E+13vg/mL		
**Date of Manufacture:**	30JUN25 (Vialing Date)		
**Expiration Date:**	Pending Stability Testing		
**Storage Conditions:**	≤ -60 °C		
**Yield (for Research Use):**	4 vials x 1.5 mL, 1 vial x 0.6 mL, 1 vial x 2.0 mL		

Release Testing

Test Description	Testing Facility & Method ID	Acceptance Criteria	Result
**Genomic Titer** – Droplet Digital Polymerase Chain Reaction (ddPCR) Assay. When performed using the AAV2 ITR (DDPPS002) Target, the result is equal to the Vector Genome titer.	Andelyn ADMD-000008, Version 2	Report Result	2.16E+13 vg/mL
**Genomic Titer** – Droplet Digital Polymerase Chain Reaction (ddPCR) Assay. When performed using the ERCC8 (DDPPS015) Target, the result is equal to the Vector Genome titer.	Andelyn SPECMD-000072, Version 1	Report Result	1.49E+13 vg/mL
**Total Protein Quantification** - Micro-BCA quantification of total protein.	Andelyn ADMD-000004, Version 1	Report Result	231 µg/mL
**Purity** - Semi-quantitative determination of purty by SDS-PAGE. Impurities are quantified by comparison of band intensities to standards of known quantity (1%, 2% and 3% of total protein).	Andelyn, ADMD-000009, Version 1	Report Result	No impurity bands detected<br>Total Purity: 100%
**Endotoxin** – Kinetic Chromogenic LAL Assay.	Andelyn ADMD-000003, Version 2	Report Result	0.178 EU/mL
**Bioburden** – Membrane Filtration with modifications for quantitative analysis.	Andelyn SPECMD-000026-A, Version 3	Report Result	No Growth<br><br>TAMC: < 1 CFU/0.240 mL<br>TYMC: < 1 CFU/0.240 mL
**Mycoplasma (USP)** – PCR for the detection of Mycoplasma in cell culture	Andelyn SPECMD-000030-A, Version 3	Report Result	Negative for Mycoplasma DNA

Effective

Confidential and Proprietary
THE USER OF THIS DOCUMENT IS RESPONSIBLE FOR ENSURING IT IS THE CURRENT VERSION

Appendix 2

Andelyn BIOSCIENCES | Advancing Discoveries. Manufacturing Cures.

R&D CERTIFICATE OF ANALYSIS			Page 2 of 3
**R&D COA ID #:**	**RDCOA-000136**	**Version:**	**1**
**Product Name:**	scAAV9-CB-ERCC8		
**Product Description:**	The biological product scAAV9-CB-ERCC8 is a self-complimentary (sc) non-replicating recombinant adeno-associated virus 9 (AAV9) for non-clinical research use.		
**Lot Number:**	PDTP-000102		
**Concentration:**	2.16E+13vg/mL		
**Date of Manufacture:**	30JUN25 (Vialing Date)		
**Expiration Date:**	Pending Stability Testing		
**Storage Conditions:**	≤ -60 °C		
**Yield (for Research Use):**	4 vials x 1.5 mL, 1 vial x 0.6 mL, 1 vial x 2.0 mL		

Release Testing

Test Description	Testing Facility & Method ID	Acceptance Criteria	Result
**Identity (DNA)** – Vector DNA Identity by Next Generation Sequencing	Andelyn SPECMD-000045-A, Version 1	Report Result	TBD
**Identity (Protein)** – Vector Protein Identity by Western Blot analysis using antibodies specific to AAV VP1, VP2, and VP3.	Andelyn SPECMD-000057-A, Version 1	Report Result	Positive for AAV Capsid Protein
**Color & Achromicity** – observation and visual inspection for color and Achromicity.	Andelyn SPECMD-000037-A, Version 2	Report Result	TBD
**Visible Particulates** – observation and visual inspection for presence of visible particulate	Andelyn SPECMD-000060-A, Version 1	Report Result	TBD
**Osmolality** – Freezing Point Depression	Andelyn SPECMD-000010-A, Version 4	Report Result	415.3 mOsm/kg
**pH** – pH assay utilizing a micro volume pH probe	Andelyn ADMD-000025, Version 1	Report Result	pH = 7.98
**Replication Competent AAV** - Detection and quantification of replication competent AAV by rcAAV amplification in HEK293 cells. Presence of rcAAV is determined after serial passage by qPCR using AAV REP2 Target. Sensitivity of the assay is determined by simultaneous testing of wild-type AAV2	Andelyn SPECMD-000032-A, Version 2	Report Result	TBD
**Full: Empty Ratio** – Analytical Ultra Centrifugation to determine the ratio of full and empty viral particles	Andelyn SPECMD-000078-A, Version 1	Report Result	TBD

Effective

Confidential and Proprietary
THE USER OF THIS DOCUMENT IS RESPONSIBLE FOR ENSURING IT IS THE CURRENT VERSION

Appendix 2

Andelyn BIOSCIENCES | Advancing Discoveries. Manufacturing Cures.

R&D CERTIFICATE OF ANALYSIS			Page 3 of 3
**R&D COA ID #:**	**RDCOA-000136**	**Version:**	**1**
**Product Name:**	scAAV9-CB-ERCC8		
**Product Description:**	The biological product scAAV9-CB-ERCC8 is a self-complimentary (sc) non-replicating recombinant adeno-associated virus 9 (AAV9) for non-clinical research use.		
**Lot Number:**	PDTP-000102		
**Concentration:**	2.16E+13vg/mL		
**Date of Manufacture:**	30JUN25 (Vialing Date)		
**Expiration Date:**	Pending Stability Testing		
**Storage Conditions:**	≤ -60 °C		
**Yield (for Research Use):**	4 vials x 1.5 mL, 1 vial x 0.6 mL, 1 vial x 2.0 mL		

Release Testing			
**Test Description**	**Testing Facility & Method ID**	**Acceptance Criteria**	**Result**
**Residual FectoVIR**	To be performed and reported by sponsor.		
**Residual Affinity Ligand**	Andelyn SPECMD-000069-A	Report Result	TBD
**Residual Poloxamer Concentration**	Element TBD	Report Result	TBD

Effective

Confidential and Proprietary
THE USER OF THIS DOCUMENT IS RESPONSIBLE FOR ENSURING IT IS THE CURRENT VERSION

Appendix 2

Andelyn BIOSCIENCES | Advancing Discoveries. Manufacturing Cures.

R&D CERTIFICATE OF ANALYSIS		Page 1 of 1
TITLE:	R&D COA ID:	RDCOA-000136
R&D Certificate of Analysis for Product scAAV9-CB-ERCC8, PDTP-000102	Version:	1

DOCUMENT HISTORY		
Summary of Changes from Prior Versions		
DOCUMENT VERSION	DESCRIPTION OF CHANGES	JUSTIFICATION
1	N/A	New Document

Effective

Appendix 2

Cover Page for R&D CoA – TMN200P, TMNP0325-B RDCOA-000127

Document Details	
Document Name	R&D CoA – TMN200P, TMNP0325-B
Document Number	RDCOA-000127
Version Status	Effective
Effective Date Time	11 Jun 2025 16:37:38 (EDT)
Document Version	1

Effective

Document Reviewers

First Name	Last Name	Email	Date/Time Reviewed
Cara	Allen	cara.allen@andelynbiosciences.com	09 Jun 2025 14:48:24 (EDT)

Document Approvers

First Name	Last Name	Email	Date/Time Approved

Appendix 2

Marissa	Evans	marissa.evans@andelynbiosciences.com	11 Jun 2025 16:35:01 (EDT)

Effective

Appendix 2

Andelyn BIOSCIENCES | Advancing Discoveries. Manufacturing Cures.

R&D CERTIFICATE OF ANALYSIS			Page 1 of 1
**R&D COA ID #:**	**RDCOA-000127**	**Version:**	**1**
**Product Name:**	TMN200P		
**Product Description:**	Formulation Buffer [20 mM Tris (pH 8.0), 1 mM $MgCl_2$ and 200 mM NaCl containing 0.006% (w/v) Poloxamer 188] which will serve as a diluent for client Toxicology studies.		
**Lot Number:**	TMNP0325-B		
**Concentration:**	N/A		
**Date of Manufacture:**	19MAR25		
**Expiration Date:**	Pending Stability Testing		
**Storage Conditions:**	≤ -60 °C		
**Yield (for Research Use):**	51 Vials at 10.0 mL		

Release Testing			
**Test Description**	**Testing Facility & Method ID**	**Acceptance Criteria**	**Result**
**Bioburden**- Membrane Filtration with modifications for quantitative analysis.	Andelyn SPECMD-000026-A, Version 2	Report Result	No Growth Observed<br><br>Vial # 8<br>TAMC: < 1 CFU/4.9 mL<br>TYMC: < 1 CFU/4.9 mL<br><br>Vial # 22<br>TAMC: < 1 CFU/4.9 mL<br>TYMC: < 1 CFU/4.9 mL<br><br>Vial # 37<br>TAMC: < 1 CFU/5.0 mL<br>TYMC: < 1 CFU/5.0 mL<br><br>Vial # 57<br>TAMC: < 1 CFU/4.9 mL<br>TYMC: < 1 CFU/4.9 mL
**Endotoxin** – Kinetic Chromogenic (USP/FDA I/E Assay).	Andelyn ADMD-000003, Version 2	≤ 0.2 EU/mL	<0.050 EU/mL
**Osmolality** – Freezing Point Depression.	Andelyn SPECMD-000010-A, Version 3[1]	382-423 mOsm/kg	413.3 mOsm/kg
**pH** – pH assay utilizing a micro volume pH probe.	Andelyn ADMD-000025, Version 1	pH = 8.0 ± 0.5	pH = 7.87
**Appearance** – Visual observation of Color.	Andelyn SPECMD-000037-A, Version 2	Colorless to Faint White Solution	Meets Specification<br><br>Colorless

[1] SPECMD-000010-A is GxP version of ADMD-000026; no Quality Assurance review required.

Effective

Confidential and Proprietary
THE USER OF THIS DOCUMENT IS RESPONSIBLE FOR ENSURING IT IS THE CURRENT VERSION

Appendix 2

Andelyn BIOSCIENCES

R&D CERTIFICATE OF ANALYSIS		Page 1 of 1
TITLE:	R&D COA ID:	RDCOA-000127
R&D Certificate of Analysis for TMN200P, TMNP0325-B	Version:	1

DOCUMENT HISTORY		
Summary of Changes from Prior Versions		
DOCUMENT VERSION	DESCRIPTION OF CHANGES	JUSTIFICATION
1	None	New Document

Effective

DATA EXPLANATION PAGE

All Day(s) referenced throughout the outputs generated are based on Day 1 (day of dose administration).

Note: All abbreviations listed on these pages may not be applicable to this report.

Abbreviation	Description
% Diff	% Difference from Group 1
tCtrl	Times control (fold change)
1F, 2F, 3F, 4F	Group 1 Female, Group 2 Female, Group 3 Female, Group 4 Female
1M, 2M, 3M, 4M	Group 1 Male, Group 2 Male, Group 3 Male, Group 4 Male
N	Number of values included in analysis
M, F	Male, Female
TK	Toxicokinetic
<, >	Out of range
--	Not scheduled to be performed/dead
- or .	Not applicable

Abbreviation	Description	Abbreviation	Description
E	Exclude	Temp	Temperature
Int.	Interval	TERR	Technical error
NA	Not applicable	UPTD	Unable to perform due to technical difficulty
NC	Not calculable	UTD	Unable to determine
No.	Animal number	Wt	Weight
NSCH	Not scheduled to be performed		

TIMESLOTS

4H – 4 hours postdose
24H – 24 hours postdose

CAGESIDE AND CLINICAL OBSERVATIONS

Only animals with findings are presented in this table and certain days may not be represented if all animals had no abnormalities detected on that day.

Abbreviation	Description	Abbreviation	Description
-	Severity not recorded	N	Severity not applicable
!	Comment present	SIRT	Signs of ill response to treatment
X	Present	PT	Permanent
Unsc/Unsch	Unscheduled	NAD	No abnormalities detected

BODY WEIGHTS

Abbreviation	Description	Abbreviation	Description
AVS	Suspected aberrant value	TERR	Technical Error
E	Excluded	UPTD	Unable to perform due to technical difficulty
OA	Omitted Activity	X	Excluded from mean

SURVIVAL/MORTALITY

Abbreviation	Description	Abbreviation	Description
ACCD	Accidental death	PostRx	Observation postdosing
AD	Accidental death	PreRx	Observation predosing
AM SIRT	Mortality/moribundity check in the morning	REC	Recovery euthanasia
CSO	Cage side observation	REL	Released
DE	Detailed examination	TE	Terminal euthanasia
FD	Found dead	TERM	Terminal euthanasia
INTM	Interim euthanasia	UE	Unscheduled euthanasia
N+ve	Number positive	UNSC	Unscheduled euthanasia
PM SIRT	Mortality/moribundity check in the afternoon		

NEUROBEHAVIORAL ASSESSMENTS

Abbreviation	Description	Abbreviation	Description
°C	degree Celsius	N+ve	Number of animals with observation
Grp	Grip	RC	Result comment
Ha	Handling	refl	Reflex
HC	Home Cage	resp	Response
OF	Open Field	sec	seconds
Mn	Mean		

ACTIVITY OBSERVATIONS

Arousal/Alertness - The level of unprovoked activity/alertness in the open field.

-2	Very low (stuporous, comatose, little to no movement)
-1	Low (sluggish, some exploratory movement)
0	Normal (alert with exploratory movement)
1	High (tense, some bouts of startle, darting, or freezing without stimuli)
2	Very high (excited, multiple bouts of startle, running, darting, freezing, or body movements without stimuli)

Posture/Body Carriage - The position of body parts relative to each other. Certain postures indicate abnormal physiological states. This evaluation is performed prior to removing the animal from the home cage.

-2	Prostrate (limbs may be in the air) or flattened to cage floor (limbs may be spread out)
-1	Hunched or low carriage
0	Normal; asleep, sitting or standing normally

Stereotypy - Any behavior that is excessive or repetitive or exceeds that observed in normal animals. Examples of stereotypy include, but are not limited to, tight circling, repetitive grooming of an extended duration, repetitive sniffing and/or licking in one area, head weaving back and forth, and self-mutilation. Stereotypic behaviors are an indicator of abnormal central nervous system function. For scores other than 0, the specific behavior observed was recorded as a comment.

0	Not present; normal
1	Continuous licking, biting/gnawing of the wall or floor, continuous or excessive grooming
2	Repetitive mouth movements, and/or head weaving/bobbing
3	Circling, retropulsion, forepaw treading
4	Self-injurious behavior; licking, biting, or gnawing of the body

Rearing - A rear is defined as the simultaneous raising of both forefeet off the surface of the open-field test box. A rear may be a brief, low raising of both forelimbs. A rear in which the animal supports itself on the side of the box with 1 or both forelimbs is also counted. Rearing is a normal posture in rodents and is a sign of both intact motor functioning (the ability to support weight on the haunches) and normal arousal.

The number of times an animal rears onto its hindlimbs with both forelimbs raised off the surface of the open field box is recorded over the 2-minute observation period.

Appearance - An evaluation of the general appearance of the animal assessing cleanliness and grooming.

0	Clean and groomed
1	Slightly unkempt (slightly discolored / crusty fur)
2	Extremely unkempt (markedly discolored, matted, and/or crusty fur)

EXCITABILITY

Vocalization - Vocalizations usually occur in response to negative stimuli. The presence of multiple spontaneous vocalizations (not in reaction to being handled; also includes vocalizations in the open field) suggests an abnormal internal state of the animal.

0	Spontaneous vocalizations occur once or not at all
1	Spontaneous vocalizations occur more than once

Startle Response - An assessment of the reaction of the animal to an auditory stimulus of a loud, quick click. The clicker is positioned approximately 5 cm above the back of the animal and a click is sounded suddenly.

-1	No reaction
0	Normal reaction (some evidence that the noise was heard, e.g., normal freezing, flinching, or jumping behaviors)
1	Exaggerated reaction (e.g., excessive jumping, attacking, or vocalization)

Ease of Removal - An evaluation of the reaction of the animal to generalized stimuli. This assessment occurs while the animal is being removed from the home cage.

-1	Very easy (animal is sluggish or limp)
0	Easy (sits quietly, allows observer to pick it up with minimal resistance)
1	Moderate (e. g. rearing, following observer's hand, vocalization)
2	Difficult (e. g. running around cage, is hard to grab, vocalization, tail and throat rattles, attacking observer's hand)

Handling Reactivity - An evaluation of the reaction of the animal to generalized stimuli. This assessment occurs while the animal is held and examined upon removal from the home cage.

-1	Very low (animal is totally limp or otherwise unresponsive)
0	Low (minimal resistance, is easy to handle)
1	Moderate (slight resistance)
2	High (e.g., freezing, tense or rigid in hand, squirming, twisting, or attempting to bite)

Convulsions - Involuntary clonic movements characterized by alternating contraction and relaxation of musculature; or involuntary tonic movements characterized by continuous muscular contractions. Clonic movements may occur with or without tonic movements. For scores other than 0, the specific behavior observed was recorded as a comment.

0	None
1	Clonic movement of limbs, ears, head, or skin, repetitive movements of mouth and jaw; contraction of extensors such that limbs are rigid, extended
2	Clonic tremors or clonus of the limbs or whole body; opisthotonus (head and body rigidly arched backwards) or emprosthotonus (head and body rigidly extended forward)
3	Severe clonus of the limbs or whole body; myoclonic jerks; explosive jumps into the air with all feet leaving surface
4	Clonic convulsions affecting the whole body; clonic-tonic convulsions, possibly resulting in dyspnea, or death

AUTONOMIC OBSERVATIONS

Exophthalmus - An abnormal protrusion of the eyeball.

0	Not present
1	Present

Lacrimation - The excessive secretion of tears and a sign of abnormal autonomic functioning.

0	None (no excess lacrimation)
1	Slight (excess moisture at the margin of the eyelid)
2	Severe (dampness extends beyond the margin of the eyelid)

Erected Fur - Characterized by hair that is standing approximately vertical to the surface of the body of the animal. It is produced by the contractions of the small muscles around the hair follicles and is a sign of heightened arousal or abnormal autonomic functioning. During the evaluation, erected fur (piloerection) is distinguished from unkemptness by the inability of the technician to smooth the hair by stroking it from a rostral to caudal direction.

0	Not present
1	Present (coat does not lie down after stroking)

Pupil Response - The normal pupil constricts to a bright light stimulus; failure to constrict indicates abnormal autonomic or central nervous system functioning. The animal's eyes are covered for a minimum of 5 seconds, then a beam from a penlight is brought in from the side of the head to near the surface of the eye and the effect on pupil size is noted.

0	Normal constriction of pupils
1	Partial, slow, or lack of constriction of pupils
2	Pupils are fully constricted prior to performing the test (i.e., "pinpoint pupils")

Salivation - The excessive production of saliva to the extent that it is visible around the mouth of the animal. Excessive salivation is a sign of abnormal autonomic functioning.

0	None (no excess salivation)
1	Slight (margin of mouth wet)
2	Severe (wet zone extends beyond the margin of the mouth)

Defecation - The appearance of the feces is noted.

-1	Absent
0	Normal
1	Abnormal

Palpebral Closure/Ptosis - The partial or complete closure of the eyelids. It is a sign of an abnormal physiological state or, when the animal is not compromised, a sign of normal restfulness or sleep. If palpebral closure is seen as representing an abnormal physiologic state it is also called ptosis. During the evaluation, palpebral closure is evaluated both in the home cage, prior to handling, where its presence could be either normal or abnormal, and during handling, where, due to the aroused state of the animal, it is considered a reliable sign of abnormality.

0	Eyelids wide open
1	Eyelids drooping approximately half-way
2	Eyelids completely shut

NEUROMUSCULAR OBSERVATIONS

Gait/Mobility – Gait is the manner in which the animal walks; it is an indication of the integrity of central nervous system motor function. Mobility is the ability to locomote, despite gait abnormalities. The animal is evaluated during the 2-minute open-field observation period.

0	Normal gait/mobility
1	Slightly impaired (any or all of the following can be evident): mild ataxia, rocks or lurches during ambulation; hunched or crouched body position; walks on tiptoe
2	Moderately impaired (any or all of the following can be evident): marked ataxia; feet point outwards from the body; hind limbs show exaggerated or overcompensated movements, drag, or are splayed
3	Severely impaired (any or all of the following can be evident): Forelimbs drag or are unable to support weight; body drags or is flattened against surface

Grip Strength – Measured using a digital strain gauge with metal grasping bars/grid. An evaluation of the strength of the motor reflex elicited when an object is pulled against the plantar surfaces of the paws. Three measurements of both forelimb and hindlimb grip strengths are performed and averaged to produce a mean grip strength value for each animal.

Hindlimb Splay – An evaluation of muscle tonus. The bottom of the outside toe of each hind foot is dotted with ink and the animal is dropped approximately 32 cm. The distance between the impressions made by the inked digits is measured. Two measurements of hind foot splay are obtained and averaged to produce the hind foot splay value for the animal.

Air Righting Reflex - The reflexive ability of an animal to turn mid-air from a supine position to one allowing it to land upright. The animal is dropped from a height of approximately 30 cm. If the animal is paralyzed or severely impaired, this test should not be conducted, and the animal is given a score of “2”.

0	Normal (animal lands on four feet)
1	Abnormal
2	Animal severely impaired (not tested)

Tremor - Uncontrollable shaking of all or part of the body.

0	None
1	Slight (barely perceptible)
2	Moderate (apparent, but still able to perform other activities)
3	Severe (impairs general activity)

Body Tone - The animal is grasped with one hand while the other hand gently palpates the lower thorax and pelvic area to evaluate body tone. Body tone can also be evaluated by raising the animal off the surface in a vertical position such that the observer can evaluate the presence/strength of the extensor thrust reflex via digital palpation by gently pressing into the middle of the plantar surface (i.e., footpads) of each hindlimb. Either method may be used.

-1	Decreased (flaccidity)
0	Normal
1	Increased (resistance to palpation; rigidity)

SENSORIMOTOR OBSERVATIONS

Touch Response/Tactile Reflex - An assessment of the reaction of the animal to a tactile stimulus of a blunt object gently touching the rump.

-1	No reaction
0	Normal reaction (e. g. turns towards or away from stimulus)
1	Exaggerated reaction (freezes or flinches, jumps, bites, or attacks)

Thermal Response - A measurement of the latency of the animal to respond to a nociceptive heat stimulus. The animal is placed on a hot plate apparatus set to a temperature of 52°C (±1°C). The time (in seconds) between placement of the animal on the heated surface and the occurrence of a responses/escape behavior is recorded: licking of the fore or hind paws, an increase in ambulatory activity, rapidly repeated elevations of either hind paw, and/or jumping; clinging to the top of the enclosure. The animal is quickly removed from the hot plate when a response is emitted, or the maximum allowable duration has been reached (i.e., 60 seconds)

PHYSIOLOGICAL OBSERVATIONS

Respiration - The quality of the respiration of the animal is assessed as an indicator of autonomic involvement and of the general physiological state of the animal.

-1	Slowed
0	Normal
1	Rapid
2	Abnormal

Body Temperature

The body temperature of the animal was recorded in degrees Celsius (°C) using a rectal thermometer.

Appendix 3

Individual Mortality

32025165

Sex: Male Day(s): - Relative to Start Date

0 vg<br>Group 1	Day of Death	Removal Date	Path Removal Reason
1001	28	14-Aug-2025	TERM
1002	28	14-Aug-2025	TERM
1003	28	14-Aug-2025	TERM
1004	28	14-Aug-2025	TERM
1005	28	14-Aug-2025	TERM
1006	89	21-Oct-2025	TERM
1007	90	22-Oct-2025	TERM
1008	91	23-Oct-2025	TERM
1009	91	23-Oct-2025	TERM
1010	92	24-Oct-2025	TERM

TE/TERM = Terminal Euthanasia FD = Found Dead UE/UNSC = Unscheduled Euthanasia
IE/INTM = Interim Euthanasia REC = Recovery Euthanasia ETEA = Early Terminal Euthanasia

Appendix 3

Individual Mortality

32025165

Sex: Male Day(s): - Relative to Start Date

1.49x10^11 vg<br><br>Group 2	Day of Death	Removal Date	Path Removal Reason
2001	89	21-Oct-2025	TERM
2002	90	22-Oct-2025	TERM
2003	90	22-Oct-2025	TERM
2004	90	22-Oct-2025	TERM
2005	91	23-Oct-2025	TERM
2006	91	23-Oct-2025	TERM
2007	91	23-Oct-2025	TERM
2008	91	23-Oct-2025	TERM
2009	92	24-Oct-2025	TERM
2010	92	24-Oct-2025	TERM

TE/TERM = Terminal Euthanasia FD = Found Dead UE/UNSC = Unscheduled Euthanasia
IE/INTM = Interim Euthanasia REC = Recovery Euthanasia ETEA = Early Terminal Euthanasia

Appendix 3

Individual Mortality

32025165

Sex: Male Day(s): - Relative to Start Date

4.47x10^11 vg Group 3	Day of Death	Removal Date	Path Removal Reason
3001	89	21-Oct-2025	TERM
3002	89	21-Oct-2025	TERM
3003	90	22-Oct-2025	TERM
3004	90	22-Oct-2025	TERM
3005	90	22-Oct-2025	TERM
3006	91	23-Oct-2025	TERM
3007	91	23-Oct-2025	TERM
3008	91	23-Oct-2025	TERM
3009	92	24-Oct-2025	TERM
3010	92	24-Oct-2025	TERM

TE/TERM = Terminal Euthanasia FD = Found Dead UE/UNSC = Unscheduled Euthanasia
IE/INTM = Interim Euthanasia REC = Recovery Euthanasia ETEA = Early Terminal Euthanasia

Appendix 3

Individual Mortality

32025165

Sex: Male Day(s): - Relative to Start Date

1.49x10^12 vg<br><br>Group 4	Day of Death	Removal Date	Path Removal Reason
4001	28	14-Aug-2025	TERM
4002	28	14-Aug-2025	TERM
4003	28	14-Aug-2025	TERM
4004	28	14-Aug-2025	TERM
4005	28	14-Aug-2025	TERM
4006	89	21-Oct-2025	TERM
4007	89	21-Oct-2025	TERM
4008	90	22-Oct-2025	TERM
4009	90	22-Oct-2025	TERM
4010	91	23-Oct-2025	TERM
4011	91	23-Oct-2025	TERM
4012	91	23-Oct-2025	TERM
4013	92	24-Oct-2025	TERM
4014	92	24-Oct-2025	TERM
4015	92	24-Oct-2025	TERM

TE/TERM = Terminal Euthanasia FD = Found Dead UE/UNSC = Unscheduled Euthanasia
IE/INTM = Interim Euthanasia REC = Recovery Euthanasia ETEA = Early Terminal Euthanasia

Appendix 3

Individual Mortality

32025165

Sex: Female Day(s): - Relative to Start Date

0 vg<br>Group 1	Day of Death	Removal Date	Path Removal Reason
1501	29	15-Aug-2025	TERM
1502	29	15-Aug-2025	TERM
1503	29	15-Aug-2025	TERM
1504	29	15-Aug-2025	TERM
1505	29	15-Aug-2025	TERM
1506	91	29-Oct-2025	TERM
1507	91	29-Oct-2025	TERM
1508	91	29-Oct-2025	TERM
1509	92	30-Oct-2025	TERM
1510	93	31-Oct-2025	TERM

TE/TERM = Terminal Euthanasia FD = Found Dead UE/UNSC = Unscheduled Euthanasia
IE/INTM = Interim Euthanasia REC = Recovery Euthanasia ETEA = Early Terminal Euthanasia

Appendix 3

Individual Mortality

32025165

Sex: Female Day(s): - Relative to Start Date

1.49x10^11 vg<br>Group 2	Day of Death	Removal Date	Path Removal Reason
2501	91	29-Oct-2025	TERM
2502	91	29-Oct-2025	TERM
2503	91	29-Oct-2025	TERM
2504	91	29-Oct-2025	TERM
2505	91	29-Oct-2025	TERM
2506	91	29-Oct-2025	TERM
2507	92	30-Oct-2025	TERM
2508	92	30-Oct-2025	TERM
2509	92	30-Oct-2025	TERM
2510	93	31-Oct-2025	TERM

TE/TERM = Terminal Euthanasia FD = Found Dead UE/UNSC = Unscheduled Euthanasia
IE/INTM = Interim Euthanasia REC = Recovery Euthanasia ETEA = Early Terminal Euthanasia

Appendix 3

Individual Mortality

32025165

Sex: Female Day(s): - Relative to Start Date

4.47x10^11 vg<br><br>Group 3	Day of Death	Removal Date	Path Removal Reason
3501	91	29-Oct-2025	TERM
3502	91	29-Oct-2025	TERM
3503	91	29-Oct-2025	TERM
3504	91	29-Oct-2025	TERM
3505	91	29-Oct-2025	TERM
3506	91	29-Oct-2025	TERM
3507	92	30-Oct-2025	TERM
3508	92	30-Oct-2025	TERM
3509	92	30-Oct-2025	TERM
3510	93	31-Oct-2025	TERM

TE/TERM = Terminal Euthanasia FD = Found Dead UE/UNSC = Unscheduled Euthanasia
IE/INTM = Interim Euthanasia REC = Recovery Euthanasia ETEA = Early Terminal Euthanasia

Appendix 3

Individual Mortality

32025165

Sex: Female Day(s): - Relative to Start Date

1.49x10^12 vg<br><br>Group 4	Day of Death	Removal Date	Path Removal Reason
4501	29	15-Aug-2025	TERM
4502	29	15-Aug-2025	TERM
4503	29	15-Aug-2025	TERM
4504	29	15-Aug-2025	TERM
4505	29	15-Aug-2025	TERM
4506	91	29-Oct-2025	TERM
4507	91	29-Oct-2025	TERM
4508	91	29-Oct-2025	TERM
4509	91	29-Oct-2025	TERM
4510	91	29-Oct-2025	TERM
4511	92	30-Oct-2025	TERM
4512	92	30-Oct-2025	TERM
4513	92	30-Oct-2025	TERM
4514	93	31-Oct-2025	TERM
4515	93	31-Oct-2025	TERM

TE/TERM = Terminal Euthanasia FD = Found Dead UE/UNSC = Unscheduled Euthanasia
IE/INTM = Interim Euthanasia REC = Recovery Euthanasia ETEA = Early Terminal Euthanasia

Appendix 4

Individual Clinical Observations

32025165

0 vg<br>Group 1<br>Sex: Male	Observation Type: Toxicology Observations	Day(s) Relative to Start Date						
		-1	7	14	21	28	35	42
1001	Normal	X	X	X	X	X	.	.
1002	Normal	X	X	X	X	X	.	.
1003	Normal	X	X	X	X	X	.	.
1004	Normal	X	X	X	X	X	.	.
1005	Normal	X	X	X	X	X	.	.
1006	Normal	X	X	X	X	X	X	X
1007	Normal	X	X	X	X	X	X	X
	Fur, Thin Cover, Forelimb, Right	.	.	.	.	.	.	.
	Fur, Discolored, Dorsal Cervical, Brown	.	.	.	.	.	.	.
1008	Normal	X	X	X	X	.	.	.
	Skin, Scab, Dorsal Cervical	.	.	.	.	.	X	X
	Fur, Discolored, Dorsal Cervical, Brown	.	.	.	.	X	X	X
1009	Normal	X	X	X	X	X	X	X
	Dehydrated Suspected, Severity Not Recorded	.	.	.	.	.	.	.
	Thin	.	.	.	.	.	.	.
	Fur, Discolored, Dorsal Cervical, Brown	.	.	.	.	.	.	.
1010	Normal	X	X	X	X	.	X	X
	Dehydrated Suspected, Severity Not Recorded	.	.	.	.	.	.	.
	Skin, Scab, Face	.	.	.	.	X	.	.
	Fur, Discolored, Dorsal Cervical, Brown	.	.	.	.	X	.	.
	Fur, Discolored, Periorbital, Left, Red	.	.	.	.	.	.	.
	Fur, Discolored, Periorbital, Right, Red	.	.	.	.	.	.	.

X=Present

Appendix 4

Individual Clinical Observations

32025165

0 vg<br>Group 1<br>Sex: Male	Observation Type: Toxicology Observations	Day(s) Relative to Start Date						
		49	56	63	70	77	84	90
1001	Normal	.	.	.	.	.	.	.
1002	Normal	.	.	.	.	.	.	.
1003	Normal	.	.	.	.	.	.	.
1004	Normal	.	.	.	.	.	.	.
1005	Normal	.	.	.	.	.	.	.
1006	Normal	X	X	X	X	X	X	.
1007	Normal	X	.	.	.	.	.	.
	Fur, Thin Cover, Forelimb, Right	.	X	X	X	X	X	X
	Fur, Discolored, Dorsal Cervical, Brown	.	.	.	X	X	X	X
1008	Normal	.	.	.	.	.	.	.
	Skin, Scab, Dorsal Cervical	.	.	.	.	.	.	.
	Fur, Discolored, Dorsal Cervical, Brown	X	X	X	X	X	X	.
1009	Normal	X	X	X	.	.	.	.
	Dehydrated Suspected, Severity Not Recorded	.	.	.	.	X	X	.
	Thin	.	.	.	.	.	X	.
	Fur, Discolored, Dorsal Cervical, Brown	.	.	.	X	X	X	.
1010	Normal	.	.	.	.	.	.	.
	Dehydrated Suspected, Severity Not Recorded	.	.	.	.	X	X	.
	Skin, Scab, Face	.	.	.	.	.	.	.
	Fur, Discolored, Dorsal Cervical, Brown	.	.	.	.	.	.	.
	Fur, Discolored, Periorbital, Left, Red	X	X	X	X	X	X	.
	Fur, Discolored, Periorbital, Right, Red	X	X	X	X	X	X	.

X=Present

Appendix 4

Individual Clinical Observations

32025165

0 vg<br>Group 1<br>Sex: Male	Observation Type: Toxicology Observations	Day(s) Relative to Start Date						
		91						
1001	Normal	.						
1002	Normal	.						
1003	Normal	.						
1004	Normal	.						
1005	Normal	.						
1006	Normal	.						
1007	Normal	.						
	Fur, Thin Cover, Forelimb, Right	.						
	Fur, Discolored, Dorsal Cervical, Brown	.						
1008	Normal	.						
	Skin, Scab, Dorsal Cervical	.						
	Fur, Discolored, Dorsal Cervical, Brown	X						
1009	Normal	.						
	Dehydrated Suspected, Severity Not Recorded	.						
	Thin	.						
	Fur, Discolored, Dorsal Cervical, Brown	X						
1010	Normal	.						
	Dehydrated Suspected, Severity Not Recorded	.						
	Skin, Scab, Face	.						
	Fur, Discolored, Dorsal Cervical, Brown	.						
	Fur, Discolored, Periorbital, Left, Red	X						
	Fur, Discolored, Periorbital, Right, Red	X						

X=Present

Appendix 4

Individual Clinical Observations

32025165

1.49x10^11 vg Group 2 Sex: Male	Observation Type: Toxicology Observations	Day(s) Relative to Start Date						
		-1	7	14	21	28	35	42
2001	Normal	X	X	X	X	X	X	X
2002	Normal	X	X	X	X	X	.	.
	Fur, Thin Cover, Face	.	.	.	.	.	X	X
2003	Normal	X	X	X	X	X	X	X
2004	Normal	X	X	X	X	X	X	X
2005	Normal	X	X	X	X	X	X	X
	Hypersensitive, Slight	.	.	.	.	.	.	.
2006	Normal	X	X	X	X	X	.	.
	Fur, Thin Cover, Face	.	.	.	.	.	.	.
	Skin, Scab, Face	.	.	.	.	.	X	X
2007	Normal	X	X	X	X	X	X	X
2008	Normal	X	X	X	X	X	X	X
2009	Normal	X	X	.	.	.	.	.
	Fur, Thin Cover, Face	.	.	.	.	.	X	X
	Skin, Scab, Face	.	.	X	X	X	X	X
2010	Normal	X	X	X	X	X	X	X

X=Present

Appendix 4

Individual Clinical Observations

32025165

1.49x10^11 vg Group 2 Sex: Male	Observation Type: Toxicology Observations	Day(s) Relative to Start Date						
		49	56	63	70	77	84	90
2001	Normal	X	X	X	X	X	X	.
2002	Normal	X	X	X	X	X	X	X
	Fur, Thin Cover, Face	.	.	.	.	.	.	.
2003	Normal	X	X	X	X	X	X	X
2004	Normal	X	X	X	X	X	X	X
2005	Normal	X	.	.	.	.	X	.
	Hypersensitive, Slight	.	X	X	X	X	.	.
2006	Normal	.	.	.	.	.	.	.
	Fur, Thin Cover, Face	.	.	.	X	X	X	.
	Skin, Scab, Face	X	X	X	X	X	X	.
2007	Normal	X	X	X	X	X	X	.
2008	Normal	X	X	X	X	X	X	.
2009	Normal	.	.	.	.	.	.	.
	Fur, Thin Cover, Face	X	X	X	X	X	X	.
	Skin, Scab, Face	X	X	X	X	X	X	.
2010	Normal	X	X	X	X	X	X	.

X=Present

Appendix 4

Individual Clinical Observations

32025165

1.49x10^11 vg<br>Group 2<br>Sex: Male	Observation Type: Toxicology Observations	Day(s) Relative to Start Date						
		91						
2001	Normal	.						
2002	Normal	.						
	Fur, Thin Cover, Face	.						
2003	Normal	.						
2004	Normal	.						
2005	Normal	X						
	Hypersensitive, Slight	.						
2006	Normal	.						
	Fur, Thin Cover, Face	X						
	Skin, Scab, Face	X						
2007	Normal	X						
2008	Normal	X						
2009	Normal	.						
	Fur, Thin Cover, Face	X						
	Skin, Scab, Face	X						
2010	Normal	X						

X=Present

Appendix 4

Individual Clinical Observations

32025165

4.47x10^11 vg Group 3 Sex: Male	Observation Type: Toxicology Observations	Day(s) Relative to Start Date						
		-1	7	14	21	28	35	42
3001	Normal	X	X	X	X	X	X	X
3002	Normal	X	X	X	X	X	X	X
	Fur, Discolored, Dorsal Cervical, Brown	.	.	.	.	.	.	.
3003	Normal	X	X	X	X	X	X	X
	Fur, Thin Cover, Face	.	.	.	.	.	.	.
3004	Normal	X	X	X	X	.	.	.
	Fur, Staining, Eyelid, Right, Brown	.	.	.	.	X	X	X
	Hypersensitive, Slight	.	.	.	.	.	.	.
	Fur, Discolored, Dorsal Cervical, Brown	.	.	.	.	.	.	.
3005	Normal	X	X	X	X	X	X	X
	Hypersensitive, Slight	.	.	.	.	.	.	.
	Fur, Discolored, Dorsal Thoracic, Red	.	.	.	.	.	.	.
3006	Normal	X	X	X	X	.	.	.
	Fur, Thin Cover, Face	.	.	.	.	.	X	X
	Skin, Scab, Face	.	.	.	.	X	X	X
	Hypersensitive, Slight	.	.	.	.	.	.	.
3007	Normal	X	X	X	X	X	X	X
	Hypersensitive, Slight	.	.	.	.	.	.	.
	Fur, Discolored, Dorsal Cervical, Brown	.	.	.	.	.	.	.
3008	Normal	X	X	X	X	X	X	X
	Hypersensitive, Slight	.	.	.	.	.	.	.
3009	Normal	X	X	X	X	X	X	X
3010	Normal	X	X	X	X	.	.	.
	Vocalization, Increased	.	.	.	.	.	.	.
	Hypersensitive, Slight	.	.	.	.	X	X	X

X=Present

Appendix 4

Individual Clinical Observations

32025165

4.47x10^11 vg Group 3 Sex: Male	Observation Type: Toxicology Observations	Day(s) Relative to Start Date						
		49	56	63	70	77	84	90
3001	Normal	X	X	X	X	X	X	.
3002	Normal	X	X	X	.	.	.	.
	Fur, Discolored, Dorsal Cervical, Brown	.	.	.	X	X	X	.
3003	Normal	X	X	X	.	.	X	X
	Fur, Thin Cover, Face	.	.	.	X	X	.	.
3004	Normal	X	.	.	.	.	.	.
	Fur, Staining, Eyelid, Right, Brown	.	.	.	.	.	.	.
	Hypersensitive, Slight	.	X	X	X	X	X	X
	Fur, Discolored, Dorsal Cervical, Brown	.	.	.	X	X	.	.
3005	Normal	X	X	.	.	.	.	.
	Hypersensitive, Slight	.	.	X	X	X	X	X
	Fur, Discolored, Dorsal Thoracic, Red	.	.	.	X	X	X	X
3006	Normal	.	.	.	.	.	.	.
	Fur, Thin Cover, Face	X	X	X	X	X	X	.
	Skin, Scab, Face	X	X	X	X	X	X	.
	Hypersensitive, Slight	.	.	X	X	X	X	.
3007	Normal	X	X	.	.	.	.	.
	Hypersensitive, Slight	.	.	X	X	X	X	.
	Fur, Discolored, Dorsal Cervical, Brown	.	.	.	X	.	.	.
3008	Normal	X	X	X	X	X	.	.
	Hypersensitive, Slight	.	.	.	.	.	X	.
3009	Normal	X	X	X	X	X	X	.
3010	Normal	.	.	.	.	.	.	.
	Vocalization, Increased	.	.	X	X	X	X	.
	Hypersensitive, Slight	X	X	X	X	X	X	.

X=Present

Appendix 4

Individual Clinical Observations

32025165

4.47x10^11 vg Group 3 Sex: Male	Observation Type: Toxicology Observations	Day(s) Relative to Start Date						
		91						
3001	Normal	.						
3002	Normal	.						
	Fur, Discolored, Dorsal Cervical, Brown	.						
3003	Normal	.						
	Fur, Thin Cover, Face	.						
3004	Normal	.						
	Fur, Staining, Eyelid, Right, Brown	.						
	Hypersensitive, Slight	.						
	Fur, Discolored, Dorsal Cervical, Brown	.						
3005	Normal	.						
	Hypersensitive, Slight	.						
	Fur, Discolored, Dorsal Thoracic, Red	.						
3006	Normal	.						
	Fur, Thin Cover, Face	X						
	Skin, Scab, Face	X						
	Hypersensitive, Slight	X						
3007	Normal	.						
	Hypersensitive, Slight	X						
	Fur, Discolored, Dorsal Cervical, Brown	.						
3008	Normal	.						
	Hypersensitive, Slight	X						
3009	Normal	X						
3010	Normal	.						
	Vocalization, Increased	X						
	Hypersensitive, Slight	X						

X=Present

Appendix 4

Individual Clinical Observations

32025165

1.49x10^12 vg Group 4 Sex: Male	Observation Type: Toxicology Observations	Day(s) Relative to Start Date						
		-1	7	14	21	28	35	42
4001	Normal	X	X	X	X	X	.	.
4002	Normal	X	X	X	X	X	.	.
4003	Normal	X	X	X	X	X	.	.
4004	Normal	X	X	X	X	X	.	.
4005	Normal	X	X	X	X	X	.	.
4006	Normal	X	X	X	X	.	.	.
	Vocalization, Increased	.	.	.	.	.	.	.
	Fur, Staining, Dorsal Cervical, Brown	.	.	.	.	X	X	X
	Fur, Discolored, Dorsal Cervical, Brown	.	.	.	.	.	.	.
4007	Normal	X	X	X	X	X	X	X
4008	Normal	X	X	X	X	X	X	X
	Aggression	.	.	.	.	.	.	.
4009	Normal	X	X	X	X	X	X	X
	Fur, Discolored, Face, Brown	.	.	.	.	.	.	.
4010	Normal	X	X	X	X	X	X	X
	Vocalization, Increased	.	.	.	.	.	.	.
	Hypersensitive, Moderate	.	.	.	.	.	.	.
	Hypersensitive, Slight	.	.	.	.	.	.	.
4011	Normal	X	X	X	X	.	.	.
	Fur, Thin Cover, Face	.	.	.	.	X	X	X
	Skin, Scab, Face	.	.	.	.	.	X	X
4012	Fur, Thin Cover, Face	.	.	X	X	X	.	.
	Hypersensitive, Moderate	.	.	.	.	.	X	X
	Hypersensitive, Slight	X	X	X	X	X	.	.
4013	Normal	X	X	X	X	X	.	.

X=Present

Appendix 4

Individual Clinical Observations

32025165

1.49x10^12 vg Group 4 Sex: Male	Observation Type: Toxicology Observations	Day(s) Relative to Start Date						
		-1	7	14	21	28	35	42
4013	Fur, Thin Cover, Abdominal	.	.	.	.	.	.	.
	Fur, Thin Cover, Face	.	.	.	.	.	X	X
	Skin, Scab, Abdominal	.	.	.	.	.	.	.
	Skin, Scab, Face	.	.	.	.	.	X	X
4014	Normal	X	X	X	X	.	.	.
	Fur, Staining, Dorsal Cervical, Brown	.	.	.	.	X	X	X
	Fur, Discolored, Dorsal Cervical, Brown	.	.	.	.	.	.	.
4015	Normal	X	X	X	X	X	X	X

X=Present

Appendix 4

Individual Clinical Observations

32025165

1.49x10^12 vg Group 4 Sex: Male	Observation Type: Toxicology Observations	Day(s) Relative to Start Date						
		49	56	63	70	77	84	90
4001	Normal	.	.	.	.	.	.	.
4002	Normal	.	.	.	.	.	.	.
4003	Normal	.	.	.	.	.	.	.
4004	Normal	.	.	.	.	.	.	.
4005	Normal	.	.	.	.	.	.	.
4006	Normal	X	X	X	.	.	.	.
	Vocalization, Increased	.	.	.	.	.	X	.
	Fur, Staining, Dorsal Cervical, Brown	.	.	.	.	.	.	.
	Fur, Discolored, Dorsal Cervical, Brown	.	.	.	X	X	X	.
4007	Normal	X	X	X	X	X	X	.
4008	Normal	X	X	X	.	.	X	X
	Aggression	.	.	.	X	X	.	.
4009	Normal	X	X	X	.	.	.	.
	Fur, Discolored, Face, Brown	.	.	.	X	X	X	X
4010	Normal	X	X	.	.	.	.	.
	Vocalization, Increased	.	.	X	X	X	X	.
	Hypersensitive, Moderate	.	.	.	X	X	X	.
	Hypersensitive, Slight	.	.	X	.	.	.	.
4011	Normal	.	.	.	.	X	X	.
	Fur, Thin Cover, Face	X	X	X	X	.	.	.
	Skin, Scab, Face	X	X	X	X	.	.	.
4012	Fur, Thin Cover, Face	.	.	.	.	.	.	.
	Hypersensitive, Moderate	X	X	X	X	X	X	.
	Hypersensitive, Slight	.	.	.	.	.	.	.
4013	Normal	.	.	.	.	.	.	.

X=Present

Appendix 4

Individual Clinical Observations

32025165

1.49x10^12 vg Group 4 Sex: Male	Observation Type: Toxicology Observations	Day(s) Relative to Start Date						
		49	56	63	70	77	84	90
4013	Fur, Thin Cover, Abdominal	.	.	X	X	X	X	.
	Fur, Thin Cover, Face	X	X	X	X	X	X	.
	Skin, Scab, Abdominal	.	.	X	X	.	.	.
	Skin, Scab, Face	X	X	.	.	.	.	.
4014	Normal	X	X	X	.	.	.	.
	Fur, Staining, Dorsal Cervical, Brown	.	.	.	.	.	.	.
	Fur, Discolored, Dorsal Cervical, Brown	.	.	.	X	X	X	.
4015	Normal	X	X	X	X	X	X	.

X=Present

Appendix 4

Individual Clinical Observations

32025165

1.49x10^12 vg Group 4 Sex: Male	Observation Type: Toxicology Observations	Day(s) Relative to Start Date						
		91						
4001	Normal	.						
4002	Normal	.						
4003	Normal	.						
4004	Normal	.						
4005	Normal	.						
4006	Normal	.						
	Vocalization, Increased	.						
	Fur, Staining, Dorsal Cervical, Brown	.						
	Fur, Discolored, Dorsal Cervical, Brown	.						
4007	Normal	.						
4008	Normal	.						
	Aggression	.						
4009	Normal	.						
	Fur, Discolored, Face, Brown	.						
4010	Normal	.						
	Vocalization, Increased	X						
	Hypersensitive, Moderate	X						
	Hypersensitive, Slight	.						
4011	Normal	X						
	Fur, Thin Cover, Face	.						
	Skin, Scab, Face	.						
4012	Fur, Thin Cover, Face	.						
	Hypersensitive, Moderate	X						
	Hypersensitive, Slight	.						
4013	Normal	.						

X=Present

Appendix 4

Individual Clinical Observations

32025165

1.49x10^12 vg<br>Group 4<br>Sex: Male	Observation Type: Toxicology Observations	Day(s) Relative to Start Date						
		91						
4013	Fur, Thin Cover, Abdominal	X						
	Fur, Thin Cover, Face	X						
	Skin, Scab, Abdominal	.						
	Skin, Scab, Face	.						
4014	Normal	.						
	Fur, Staining, Dorsal Cervical, Brown	.						
	Fur, Discolored, Dorsal Cervical, Brown	X						
4015	Normal	X						

X=Present

Appendix 4

Individual Clinical Observations

32025165

0 vg<br>Group 1<br>Sex: Female	Observation Type: Toxicology Observations	Day(s) Relative to Start Date						
		-1	7	14	21	28	35	42
1501	Normal	X	X	X	X	X	.	.
1502	Normal	X	X	X	X	X	.	.
1503	Normal	X	X	X	X	X	.	.
1504	Normal	X	X	X	X	X	.	.
1505	Normal	X	X	X	X	X	.	.
1506	Normal	X	X	X	X	.	.	X
	Fur, Discolored, Face, Brown	.	.	.	.	X	X	.
1507	Normal	X	X	X	X	X	X	X
1508	Normal	X	X	X	X	X	.	.
	Fur, Discolored, Dorsal Cervical, Brown	.	.	.	.	.	X	X
1509	Normal	X	X	X	X	X	X	X
1510	Normal	X	X	X	X	X	X	.
	Skin, Scab, Mouth	.	.	.	.	.	.	X
	Fur, Discolored, Eyelid, Right, Red	.	.	.	.	.	.	.

X=Present

Appendix 4

Individual Clinical Observations

32025165

0 vg<br>Group 1<br>Sex: Female	Observation Type: Toxicology Observations	Day(s) Relative to Start Date						
		49	56	63	70	77	84	90
1501	Normal	.	.	.	.	.	.	.
1502	Normal	.	.	.	.	.	.	.
1503	Normal	.	.	.	.	.	.	.
1504	Normal	.	.	.	.	.	.	.
1505	Normal	.	.	.	.	.	.	.
1506	Normal	X	X	X	X	X	X	.
	Fur, Discolored, Face, Brown	.	.	.	.	.	.	.
1507	Normal	X	X	X	X	X	X	.
1508	Normal	.	.	.	X	X	X	.
	Fur, Discolored, Dorsal Cervical, Brown	X	X	X	.	.	.	.
1509	Normal	X	X	X	X	X	X	.
1510	Normal	.	.	X	X	X	X	.
	Skin, Scab, Mouth	.	.	.	.	.	.	.
	Fur, Discolored, Eyelid, Right, Red	X	X	.	.	.	.	.

X=Present

Appendix 4

Individual Clinical Observations

32025165

0 vg<br>Group 1<br>Sex: Female	Observation Type: Toxicology Observations	Day(s) Relative to Start Date						
		91						
1501	Normal	.						
1502	Normal	.						
1503	Normal	.						
1504	Normal	.						
1505	Normal	.						
1506	Normal	X						
	Fur, Discolored, Face, Brown	.						
1507	Normal	X						
1508	Normal	X						
	Fur, Discolored, Dorsal Cervical, Brown	.						
1509	Normal	X						
1510	Normal	X						
	Skin, Scab, Mouth	.						
	Fur, Discolored, Eyelid, Right, Red	.						

X=Present

Appendix 4

Individual Clinical Observations

32025165

1.49x10^11 vg Group 2 Sex: Female	Observation Type: Toxicology Observations	Day(s) Relative to Start Date						
		-1	7	14	21	28	35	42
2501	Normal	X	X	X	X	X	.	.
	Vocalization, Increased	.	.	.	.	.	.	.
	Fur, Discolored, Dorsal Cervical, Brown	.	.	.	.	.	X	X
2502	Normal	X	X	X	X	X	X	X
2503	Normal	X	X	X	X	X	X	.
	Skin, Scab, Dorsal Thoracic	.	.	.	.	.	.	X
	Fur, Discolored, Face, Brown	.	.	.	.	.	.	.
2504	Normal	X	X	.	.	.	.	.
	Fur, Thin Cover, Dorsal Cervical	.	.	.	.	X	X	X
	Fur, Thin Cover, Face	.	.	X	X	X	X	X
	Skin, Scab, Face	.	.	X	X	X	X	X
2505	Normal	X	X	X	X	.	.	.
	Fur, Discolored, Face, Brown	.	.	.	.	X	X	X
2506	Normal	X	X	.	X	.	.	.
	Skin, Discolored, Tail, Red	.	.	X	.	.	.	.
	Fur, Discolored, Dorsal Cervical, Brown	.	.	.	.	X	X	X
2507	Normal	X	X	X	X	X	X	X
	Fur, Discolored, Dorsal Cervical, Brown	.	.	.	.	.	.	.
2508	Normal	X	X	X	X	X	X	.
	Fur, Discolored, Dorsal Cervical, Brown	.	.	.	.	.	.	X
2509	Normal	X	X	X	X	X	X	X
2510	Normal	X	X	X	X	X	X	X

X=Present

Appendix 4

Individual Clinical Observations

32025165

1.49x10^11 vg Group 2 Sex: Female	Observation Type: Toxicology Observations	Day(s) Relative to Start Date						
		49	56	63	70	77	84	90
2501	Normal	.	.	.	.	.	.	.
	Vocalization, Increased	X	X	X	.	.	.	.
	Fur, Discolored, Dorsal Cervical, Brown	X	X	X	X	X	X	.
2502	Normal	X	X	X	X	X	X	.
2503	Normal	X	X	.	.	X	X	.
	Skin, Scab, Dorsal Thoracic	.	.	.	.	.	.	.
	Fur, Discolored, Face, Brown	.	.	X	X	.	.	.
2504	Normal	.	.	.	.	.	.	.
	Fur, Thin Cover, Dorsal Cervical	X	X	X	X	X	X	.
	Fur, Thin Cover, Face	X	X	X	X	X	X	.
	Skin, Scab, Face	X	X	.	.	.	.	.
2505	Normal	.	.	.	.	.	.	.
	Fur, Discolored, Face, Brown	X	X	X	X	X	X	.
2506	Normal	.	.	.	.	.	.	.
	Skin, Discolored, Tail, Red	.	.	.	.	.	.	.
	Fur, Discolored, Dorsal Cervical, Brown	X	X	X	X	X	X	.
2507	Normal	X	X	X	X	X	X	.
	Fur, Discolored, Dorsal Cervical, Brown	.	.	.	.	.	.	.
2508	Normal	.	.	.	.	.	.	.
	Fur, Discolored, Dorsal Cervical, Brown	X	X	X	X	X	X	.
2509	Normal	X	X	X	X	X	X	.
2510	Normal	X	X	X	X	X	X	.

X=Present

Appendix 4

Individual Clinical Observations

32025165

1.49x10^11 vg Group 2 Sex: Female	Observation Type: Toxicology Observations	Day(s) Relative to Start Date						
		91						
2501	Normal	.						
	Vocalization, Increased	.						
	Fur, Discolored, Dorsal Cervical, Brown	X						
2502	Normal	X						
2503	Normal	X						
	Skin, Scab, Dorsal Thoracic	.						
	Fur, Discolored, Face, Brown	.						
2504	Normal	.						
	Fur, Thin Cover, Dorsal Cervical	X						
	Fur, Thin Cover, Face	X						
	Skin, Scab, Face	.						
2505	Normal	.						
	Fur, Discolored, Face, Brown	X						
2506	Normal	.						
	Skin, Discolored, Tail, Red	.						
	Fur, Discolored, Dorsal Cervical, Brown	X						
2507	Normal	.						
	Fur, Discolored, Dorsal Cervical, Brown	X						
2508	Normal	.						
	Fur, Discolored, Dorsal Cervical, Brown	X						
2509	Normal	X						
2510	Normal	X						

X=Present

Appendix 4

Individual Clinical Observations

32025165

4.47x10^11 vg Group 3 Sex: Female	Observation Type: Toxicology Observations	Day(s) Relative to Start Date						
		-1	7	14	21	28	35	42
3501	Normal	X	X	X	X	X	X	X
	Fur, Thin Cover, Lumbar	.	.	.	.	.	.	.
3502	Normal	X	X	X	X	X	X	X
3503	Normal	X	X	X	X	X	X	X
3504	Normal	X	X	X	X	X	X	X
	Fur, Thin Cover, Face	.	.	.	.	.	.	.
	Fur, Discolored, Face, Brown	.	.	.	.	.	.	.
3505	Normal	X	X	X	X	X	.	.
	Fur, Discolored, Dorsal Cervical, Brown	.	.	.	.	.	X	X
3506	Normal	X	X	X	X	X	X	X
3507	Normal	X	X	X	X	.	.	.
	Fur, Discolored, Dorsal Cervical, Brown	.	.	.	.	X	X	X
3508	Normal	X	X	X	X	.	.	.
	Fur, Discolored, Dorsal Cervical, Brown	.	.	.	.	X	X	X
3509	Fur, Thin Cover, Cranium	.	.	X	X	X	X	X
	Fur, Thin Cover, Generalized	.	.	.	.	.	.	X
	Fur, Thin Cover, Inguinal, Left	.	.	.	X	X	X	X
	Skin, Scab, Cranium	.	.	.	.	X	X	X
	Hyperreactive, Slight	X	X	.	.	.	.	.
3510	Normal	X	X	X	X	X	X	X
	Fur, Thin Cover, Forelimb, Left	.	.	.	.	.	.	.
	Fur, Thin Cover, Forelimb, Right	.	.	.	.	.	.	.

X=Present

Appendix 4

Individual Clinical Observations

32025165

4.47x10^11 vg Group 3 Sex: Female	Observation Type: Toxicology Observations	Day(s) Relative to Start Date						
		49	56	63	70	77	84	90
3501	Normal	X	X	X	X	X	X	.
	Fur, Thin Cover, Lumbar	.	.	.	.	.	.	.
3502	Normal	X	X	X	X	X	X	.
3503	Normal	X	X	X	X	X	X	.
3504	Normal	.	.	.	.	.	.	.
	Fur, Thin Cover, Face	X	.	.	.	.	.	.
	Fur, Discolored, Face, Brown	.	X	X	X	X	X	.
3505	Normal	.	.	.	.	.	.	.
	Fur, Discolored, Dorsal Cervical, Brown	X	X	X	X	X	X	.
3506	Normal	X	X	X	X	X	X	.
3507	Normal	.	.	.	.	.	.	.
	Fur, Discolored, Dorsal Cervical, Brown	X	X	X	X	X	X	.
3508	Normal	.	.	.	.	.	.	.
	Fur, Discolored, Dorsal Cervical, Brown	X	X	X	X	X	X	.
3509	Fur, Thin Cover, Cranium	X	X	X	X	X	X	.
	Fur, Thin Cover, Generalized	X	X	X	X	X	X	.
	Fur, Thin Cover, Inguinal, Left	X	X	X	X	X	X	.
	Skin, Scab, Cranium	X	.	.	.	.	.	.
	Hyperreactive, Slight	.	.	.	.	.	.	.
3510	Normal	X	X	X	X	X	X	.
	Fur, Thin Cover, Forelimb, Left	.	.	.	.	.	.	.
	Fur, Thin Cover, Forelimb, Right	.	.	.	.	.	.	.

X=Present

Appendix 4

Individual Clinical Observations

32025165

4.47x10^11 vg Group 3 Sex: Female	Observation Type: Toxicology Observations	Day(s) Relative to Start Date						
		91						
3501	Normal	.						
	Fur, Thin Cover, Lumbar	X						
3502	Normal	X						
3503	Normal	X						
3504	Normal	X						
	Fur, Thin Cover, Face	.						
	Fur, Discolored, Face, Brown	.						
3505	Normal	.						
	Fur, Discolored, Dorsal Cervical, Brown	X						
3506	Normal	X						
3507	Normal	.						
	Fur, Discolored, Dorsal Cervical, Brown	X						
3508	Normal	.						
	Fur, Discolored, Dorsal Cervical, Brown	X						
3509	Fur, Thin Cover, Cranium	X						
	Fur, Thin Cover, Generalized	X						
	Fur, Thin Cover, Inguinal, Left	X						
	Skin, Scab, Cranium	.						
	Hyperreactive, Slight	.						
3510	Normal	.						
	Fur, Thin Cover, Forelimb, Left	X						
	Fur, Thin Cover, Forelimb, Right	X						

X=Present

Appendix 4

Individual Clinical Observations

32025165

1.49x10^12 vg Group 4 Sex: Female	Observation Type: Toxicology Observations	Day(s) Relative to Start Date						
		-1	7	14	21	28	35	42
4501	Normal	X	X	X	X	X	.	.
4502	Normal	X	X	X	X	X	.	.
4503	Normal	X	X	X	X	X	.	.
4504	Normal	X	X	X	X	X	.	.
4505	Normal	X	X	X	X	X	.	.
4506	Normal	X	X	X	X	.	.	.
	Fur, Thin Cover, Face	.	.	.	.	.	.	.
	Fur, Thin Cover, Lumbar	.	.	.	.	.	.	.
	Skin, Scab, Face	.	.	.	.	X	X	X
4507	Normal	X	X	.	.	.	.	.
	Cold to Touch, Foot, Left	.	.	X	X	X	X	X
	Cold to Touch, Foot, Right	.	.	X	X	X	X	X
	Cold to Touch, Hindpaw, Left	.	.	X	X	X	X	X
	Cold to Touch, Hindpaw, Right	.	.	X	X	X	X	X
	Fur, Discolored, Face, Brown	.	.	.	.	X	.	.
4508	Normal	X	X	X	X	X	X	X
4509	Normal	X	X	X	X	X	X	X
4510	Normal	X	X	X	X	X	X	X
	Fur, Discolored, Dorsal Cervical, Brown	.	.	.	.	.	.	.
4511	Normal	X	X	X	X	X	X	X
	Fur, Thin Cover, Face	.	.	.	.	.	.	.
4512	Normal	X	X	.	.	X	X	X
	Cold to Touch, Tail	.	.	X	X	.	.	.
4513	Normal	X	X	.	.	.	.	.
	Fur, Thin Cover, Dorsal Cervical	.	.	.	.	.	.	.

X=Present

Appendix 4

Individual Clinical Observations

32025165

1.49x10^12 vg Group 4 Sex: Female	Observation Type: Toxicology Observations	Day(s) Relative to Start Date						
		-1	7	14	21	28	35	42
4513	Fur, Discolored, Dorsal Cervical, Brown	.	.	X	X	X	X	X
4514	Normal	X	X	X	X	.	X	X
	Fur, Thin Cover, Face	.	.	.	.	.	.	.
	Skin, Scab, Mouth	.	.	.	.	X	.	.
	Hypersensitive, Slight	.	.	.	.	.	.	.
4515	Normal	X	X	X	.	.	.	.
	Fur, Thin Cover, Face	.	.	.	X	X	X	X
	Skin, Scab, Face	.	.	.	.	X	X	X
	Hypersensitive, Slight	.	.	.	.	X	X	X

X=Present

Appendix 4

Individual Clinical Observations

32025165

1.49x10^12 vg Group 4 Sex: Female	Observation Type: Toxicology Observations	Day(s) Relative to Start Date						
		49	56	63	70	77	84	90
4501	Normal	.	.	.	.	.	.	.
4502	Normal	.	.	.	.	.	.	.
4503	Normal	.	.	.	.	.	.	.
4504	Normal	.	.	.	.	.	.	.
4505	Normal	.	.	.	.	.	.	.
4506	Normal	.	.	.	X	X	X	.
	Fur, Thin Cover, Face	X	X	X	.	.	.	.
	Fur, Thin Cover, Lumbar	.	.	.	.	.	.	.
	Skin, Scab, Face	X	X	.	.	.	.	.
4507	Normal	X	X	X	X	X	X	.
	Cold to Touch, Foot, Left	.	.	.	.	.	.	.
	Cold to Touch, Foot, Right	.	.	.	.	.	.	.
	Cold to Touch, Hindpaw, Left	.	.	.	.	.	.	.
	Cold to Touch, Hindpaw, Right	.	.	.	.	.	.	.
	Fur, Discolored, Face, Brown	.	.	.	.	.	.	.
4508	Normal	X	X	X	X	X	X	.
4509	Normal	X	X	X	X	X	X	.
4510	Normal	.	.	.	.	.	.	.
	Fur, Discolored, Dorsal Cervical, Brown	X	X	X	X	X	X	.
4511	Normal	X	X	.	.	.	.	.
	Fur, Thin Cover, Face	.	.	X	X	X	X	.
4512	Normal	X	X	X	X	X	X	.
	Cold to Touch, Tail	.	.	.	.	.	.	.
4513	Normal	.	.	.	.	.	.	.
	Fur, Thin Cover, Dorsal Cervical	.	X	X	X	X	X	.

X=Present

Appendix 4

Individual Clinical Observations

32025165

1.49x10^12 vg Group 4 Sex: Female	Observation Type: Toxicology Observations	Day(s) Relative to Start Date						
		49	56	63	70	77	84	90
4513	Fur, Discolored, Dorsal Cervical, Brown	X	X	X	X	X	X	.
4514	Normal	X	X	X	X	X	X	.
	Fur, Thin Cover, Face	.	.	.	.	.	.	.
	Skin, Scab, Mouth	.	.	.	.	.	.	.
	Hypersensitive, Slight	.	.	.	.	.	.	.
4515	Normal	.	.	.	.	.	.	.
	Fur, Thin Cover, Face	X	X	X	X	X	X	.
	Skin, Scab, Face	X	.	.	.	.	.	.
	Hypersensitive, Slight	X	X	X	X	X	X	.

X=Present

Appendix 4

Individual Clinical Observations

32025165

1.49x10^12 vg Group 4 Sex: Female	Observation Type: Toxicology Observations	Day(s) Relative to Start Date						
		91						
4501	Normal	.						
4502	Normal	.						
4503	Normal	.						
4504	Normal	.						
4505	Normal	.						
4506	Normal	.						
	Fur, Thin Cover, Face	.						
	Fur, Thin Cover, Lumbar	X						
	Skin, Scab, Face	.						
4507	Normal	X						
	Cold to Touch, Foot, Left	.						
	Cold to Touch, Foot, Right	.						
	Cold to Touch, Hindpaw, Left	.						
	Cold to Touch, Hindpaw, Right	.						
	Fur, Discolored, Face, Brown	.						
4508	Normal	X						
4509	Normal	X						
4510	Normal	.						
	Fur, Discolored, Dorsal Cervical, Brown	X						
4511	Normal	.						
	Fur, Thin Cover, Face	X						
4512	Normal	X						
	Cold to Touch, Tail	.						
4513	Normal	.						
	Fur, Thin Cover, Dorsal Cervical	X						

X=Present

Appendix 4

Individual Clinical Observations

32025165

1.49x10^12 vg Group 4 Sex: Female	Observation Type: Toxicology Observations	Day(s) Relative to Start Date						
		91						
4513	Fur, Discolored, Dorsal Cervical, Brown	X						
4514	Normal	.						
	Fur, Thin Cover, Face	X						
	Skin, Scab, Mouth	.						
	Hypersensitive, Slight	X						
4515	Normal	.						
	Fur, Thin Cover, Face	X						
	Skin, Scab, Face	.						
	Hypersensitive, Slight	X						

X=Present

Appendix 4

Individual Clinical Observations

32025165

0 vg<br>Group 1<br>Sex: Male	Observation Type: SIRT/Cage Side Observations	Day(s) Relative to Start Date						
		1<br>4H	2<br>24H	41				
1001	Normal	X	X	.				
1002	Normal	X	X	.				
1003	Normal	X	X	.				
1004	Normal	X	X	.				
1005	Normal	X	X	.				
1006	Normal	X	X	.				
1007	Normal	X	X	.				
1008	Normal	X	X	.				
1009	Normal	X	X	.				
1010	Normal	X	X	.				

X=Present

Appendix 4

Individual Clinical Observations

32025165

1.49x10^11 vg Group 2 Sex: Male	Observation Type: SIRT/Cage Side Observations	Day(s) Relative to Start Date						
		1 4H	2 24H	41				
2001	Normal	X	X	.				
2002	Normal	X	X	.				
2003	Normal	X	X	.				
2004	Normal	X	X	.				
2005	Normal	X	X	.				
2006	Normal	X	X	.				
2007	Normal	X	X	.				
2008	Normal	X	X	.				
2009	Normal	X	X	.				
2010	Normal	X	X	.				

X=Present

Appendix 4

Individual Clinical Observations

32025165

4.47x10^11 vg Group 3 Sex: Male	Observation Type: SIRT/Cage Side Observations	Day(s) Relative to Start Date						
		1 4H	2 24H	41				
3001	Normal	X	X	.				
3002	Normal	X	X	.				
3003	Normal	X	X	.				
3004	Normal	X	X	.				
3005	Normal	X	X	.				
3006	Normal	X	X	.				
3007	Normal	X	X	.				
3008	Normal	X	X	.				
3009	Normal	X	X	.				
3010	Normal	X	X	.				

X=Present

Appendix 4

Individual Clinical Observations

32025165

1.49x10^12 vg Group 4 Sex: Male	Observation Type: SIRT/Cage Side Observations	Day(s) Relative to Start Date						
		1 4H	2 24H	41				
4001	Normal	X	X	.				
4002	Normal	X	X	.				
4003	Normal	X	X	.				
4004	Normal	X	X	.				
4005	Normal	X	X	.				
4006	Normal	X	X	.				
4007	Normal	X	X	.				
4008	Normal	X	X	.				
4009	Normal	X	X	.				
4010	Normal	X	X	.				
4011	Normal	X	X	.				
4012	Normal	X	X	.				
4013	Normal	X	X	.				
4014	Normal	X	X	.				
4015	Normal	X	X	.				

X=Present

Appendix 4

Individual Clinical Observations

32025165

0 vg<br>Group 1<br>Sex: Female	Observation Type: SIRT/Cage Side Observations	Day(s) Relative to Start Date						
		1<br>4H	2<br>24H	41				
1501	Normal	X	X	.				
1502	Normal	X	X	.				
1503	Normal	X	X	.				
1504	Normal	X	X	.				
1505	Normal	X	X	.				
1506	Normal	X	X	.				
1507	Normal	X	X	.				
1508	Normal	X	X	.				
1509	Normal	X	X	.				
1510	Normal	X	X	.				

X=Present

Appendix 4

Individual Clinical Observations

32025165

1.49x10^11 vg<br>Group 2<br>Sex: Female	Observation Type: SIRT/Cage Side Observations	Day(s) Relative to Start Date						
		1<br>4H	2<br>24H	41				
2501	Normal	X	X	.				
2502	Normal	X	X	.				
2503	Normal	X	X	.				
2504	Normal	X	X	.				
2505	Normal	X	X	.				
2506	Normal	X	X	.				
2507	Normal	X	X	X				
2508	Normal	X	X	X				
2509	Normal	X	X	.				
2510	Normal	X	X	.				

X=Present

Appendix 4

Individual Clinical Observations

32025165

4.47x10^11 vg<br>Group 3<br>Sex: Female	Observation Type: SIRT/Cage Side Observations	Day(s) Relative to Start Date						
		1<br>4H	2<br>24H	41				
3501	Normal	X	X	.				
3502	Normal	X	X	.				
3503	Normal	X	X	.				
3504	Normal	X	X	.				
3505	Normal	X	X	.				
3506	Normal	X	X	.				
3507	Normal	X	X	.				
3508	Normal	X	X	.				
3509	Normal	X	X	X				
3510	Normal	X	X	X				

X=Present

Appendix 4

Individual Clinical Observations

32025165

1.49x10^12 vg Group 4 Sex: Female	Observation Type: SIRT/Cage Side Observations	Day(s) Relative to Start Date						
		1 4H	2 24H	41				
4501	Normal	X	X	.				
4502	Normal	X	X	.				
4503	Normal	X	X	.				
4504	Normal	X	X	.				
4505	Normal	X	X	.				
4506	Normal	X	X	.				
4507	Normal	X	X	.				
4508	Normal	X	X	.				
4509	Normal	X	X	.				
4510	Normal	X	X	.				
4511	Normal	X	X	.				
4512	Normal	X	X	.				
4513	Normal	X	X	.				
4514	Normal	X	X	.				
4515	Normal	X	X	.				

X=Present

Appendix 5

32025165

A 13 Week IT Toxicity Study in Rats with 4-Week and 13-Week Biodistribution

0 vg Group 1	Individual Animal Exam Observation and Treatment Report: Male			
	Study Day	Animal Number	Activity	Entry Summary
	77	1009	Assessment	Animal noted with decreased fecal output and weight loss. Sipper malfunction noted and repaired during exam. Offer DG once; no additional veterinary care required at this time.
	77	1009	Examination	Obs: Activity/Gait/Locomotion; Obs: Activity, Normal
	77	1009	Examination	Obs: Appetite; Obs: Appetite, Recently Fed
	77	1009	Examination	Obs: Attitude; Obs: Bright, Alert, Responsive
	77	1009	Examination	Obs: Gastrointestinal System; Obs: Feces, Abnormal Output; Obs: Decreased, moderate
	77	1009	Examination	Obs: General Body Condition; Obs: 3 - Optimum
	77	1009	Examination	Obs: Hydration; Obs: Hydration, Normal
	77	1009	Examination	Obs: Sipper Function Assessed; Obs: Malfunction, no water, sipper repaired during exam
	77	1009	Reassessment	NA
	77	1009	Treatment	Category: Enrichment; Treatment: DietGel; Amount: 1 unit; Route: Offer; Frequency: Once; Treatment Date: 09-OCT-25; 09-Oct-2025; 13:07; Comments: 1 unit offered to cage
	84	1009	Assessment	Animal noted with mild dehydration and weight loss. Water accessible and flow optimized. Offer FE once. No further vet care required.
	84	1009	Examination	Obs: Activity/Gait/Locomotion; Obs: Activity, Normal
	84	1009	Examination	Obs: Attitude; Obs: Bright, Alert, Responsive
	84	1009	Examination	Obs: Gastrointestinal System; Obs: Feces, Abnormal Output; Obs: Decreased, moderate
	84	1009	Examination	Obs: General Body Condition; Obs: 2.5 - Lean
	84	1009	Examination	Obs: Hydration; Obs: Dehydrated; Obs: Mild
	84	1009	Examination	Obs: Sipper Function Assessed; Obs: Water Accessible, flow optimized
	84	1009	Reassessment	NA
	84	1009	Treatment	Category: Enrichment; Treatment: Food Enrichment; Route: Offer; Frequency: Once; Treatment Date: 16-OCT-25; 16-Oct-2025; 09:59; Comments: 1 jar baby food

Appendix 5

32025165

A 13 Week IT Toxicity Study in Rats with 4-Week and 13-Week Biodistribution

	Individual Animal Exam Observation and Treatment Report: Male			
0 vg<br><br>Group 1	Study Day	Animal Number	Activity	Entry Summary
	77	1010	Assessment	Animal noted with decreased fecal output and weight loss. Sipper malfunction noted and repaired during exam. Offer DG once; no additional veterinary care required at this time.
	77	1010	Examination	Obs: Activity/Gait/Locomotion; Obs: Activity, Normal
	77	1010	Examination	Obs: Appetite; Obs: Appetite, Recently Fed
	77	1010	Examination	Obs: Attitude; Obs: Bright, Alert, Responsive
	77	1010	Examination	Obs: Gastrointestinal System; Obs: Feces, Abnormal Output; Obs: Decreased, moderate
	77	1010	Examination	Obs: General Body Condition; Obs: 3 - Optimum
	77	1010	Examination	Obs: Hydration; Obs: Hydration, Normal
	77	1010	Examination	Obs: Sipper Function Assessed; Obs: Malfunction, no water, sipper repaired during exam
	77	1010	Reassessment	NA
	77	1010	Treatment	Category: Enrichment; Treatment: DietGel; Amount: 1 unit; Route: Offer; Frequency: Once; Treatment Date: 09-OCT-25; 09-Oct-2025; 13:07; Comments: 1 unit offered to cage
	84	1010	Assessment	Animal noted with mild dehydration and weight loss. Water accessible and flow optimized. Offer FE once. No further vet care required.
	84	1010	Examination	Obs: Activity/Gait/Locomotion; Obs: Activity, Normal
	84	1010	Examination	Obs: Attitude; Obs: Bright, Alert, Responsive
	84	1010	Examination	Obs: Gastrointestinal System; Obs: Feces, Abnormal Output; Obs: Decreased, moderate
	84	1010	Examination	Obs: General Body Condition; Obs: 2.5 - Lean
	84	1010	Examination	Obs: Hydration; Obs: Dehydrated; Obs: Mild
	84	1010	Examination	Obs: Sipper Function Assessed; Obs: Water Accessible, flow optimized
	84	1010	Reassessment	NA
	84	1010	Treatment	Category: Enrichment; Treatment: Food Enrichment; Route: Offer; Frequency: Once; Treatment Date: 16-OCT-25; 16-Oct-2025; 09:59; Comments: 3 WN

Appendix 6

Individual Body Weights

32025165
Sex: Male Bodyweight (g)

0 vg	Day(s) Relative to Start Date						
Group 1	-1	7	14	21	28	35	42
1001	292	342	386	420	451	-	-
1002	239	280	321	359	372	-	-
1003	272	317	351	397	416	-	-
1004	254	304	338	380	391	-	-
1005	296	323	361	382	389	-	-
1006	392	420	447	469	508	529	560
1007	339	363	410	439	468	493	520
1008	312	343	372	380	418	444	467
1009	356	402	435	466	495	525	547
1010	411	469	519	562	593	631	661
Mean	316.3	356.3	394.0	425.4	450.1	524.4	551.0
SD	57.4	58.2	59.9	61.1	68.0	68.6	71.1
N	10	10	10	10	10	5	5

Day 91 represents data collected on Day 90 and 91.

Appendix 6

Individual Body Weights

32025165
Sex: Male Bodyweight (g)

0 vg	Day(s) Relative to Start Date						
Group 1	49	56	63	70	77	84	91
1001	-	-	-	-	-	-	-
1002	-	-	-	-	-	-	-
1003	-	-	-	-	-	-	-
1004	-	-	-	-	-	-	-
1005	-	-	-	-	-	-	-
1006	584	612	628	641	648	647	-
1007	543	564	582	590	607	620	630
1008	498	514	527	550	565	582	577
1009	562	583	591	579	494	449	449
1010	682	710	724	713	617	559	570
Mean	573.8	596.6	610.4	614.6	586.2	571.4	556.5
SD	68.3	72.7	73.1	64.1	59.5	76.4	76.5
N	5	5	5	5	5	5	4

Day 91 represents data collected on Day 90 and 91.

Appendix 6

Individual Body Weights

32025165
Sex: Male Bodyweight (g)

1.49x10^11 vg	Day(s) Relative to Start Date						
Group 2	-1	7	14	21	28	35	42
2001	375	425	482	515	548	589	622
2002	296	322	345	370	397	417	427
2003	398	430	453	464	501	525	551
2004	267	294	322	337	366	389	403
2005	311	349	389	425	450	473	497
2006	310	348	387	398	422	449	465
2007	402	438	486	516	554	584	618
2008	418	460	502	528	555	586	599
2009	284	319	341	379	405	427	443
2010	383	417	450	483	510	539	557
Mean	344.4	380.2	415.7	441.5	470.8	497.8	518.2
SD	56.1	59.7	66.9	68.9	71.5	76.3	81.9
N	10	10	10	10	10	10	10
%Diff	8.9	6.7	5.5	3.8	4.6	-5.1	-6.0

Day 91 represents data collected on Day 90 and 91.

Appendix 6

Individual Body Weights

32025165
Sex: Male Bodyweight (g)

1.49x10^11 vg	Day(s) Relative to Start Date						
Group 2	49	56	63	70	77	84	91
2001	643	669	674	699	730	739	-
2002	441	461	482	484	494	497	497
2003	566	586	593	586	589	600	580
2004	419	425	451	452	462	477	483
2005	514	527	529	552	562	578	585
2006	489	500	496	516	534	533	543
2007	645	666	693	696	718	735	741
2008	622	643	656	657	680	695	709
2009	463	481	493	500	507	506	522
2010	576	603	610	616	639	650	660
Mean	537.8	556.1	567.7	575.8	591.5	601.0	591.1
SD	84.3	89.0	88.4	89.2	96.0	99.2	92.8
N	10	10	10	10	10	10	9
%Diff	-6.3	-6.8	-7.0	-6.3	0.9	5.2	6.2

Day 91 represents data collected on Day 90 and 91.

Appendix 6

Individual Body Weights

32025165
Sex: Male Bodyweight (g)

4.47x10^11 vg	Day(s) Relative to Start Date						
Group 3	-1	7	14	21	28	35	42
3001	334	373	414	436	468	485	501
3002	268	294	329	360	396	429	454
3003	269	307	347	376	401	425	448
3004	360	405	456	491	515	551	575
3005	380	426	469	498	528	556	583
3006	396	414	438	462	488	515	540
3007	321	356	389	416	434	457	474
3008	327	354	376	394	411	426	438
3009	336	370	396	421	454	467	487
3010	357	397	435	465	484	513	535
Mean	334.8	369.6	404.9	431.9	457.9	482.4	503.5
SD	42.1	43.8	46.0	47.1	46.8	49.8	52.4
N	10	10	10	10	10	10	10
%Diff	5.8	3.7	2.8	1.5	1.7	-8.0	-8.6

Day 91 represents data collected on Day 90 and 91.

Appendix 6

Individual Body Weights

32025165
Sex: Male Bodyweight (g)

4.47x10^11 vg	Day(s) Relative to Start Date						
Group 3	49	56	63	70	77	84	91
3001	524	531	535	548	561	562	-
3002	474	499	519	534	545	535	-
3003	471	486	495	509	520	524	537
3004	603	623	646	654	677	678	691
3005	601	609	627	635	682	679	689
3006	553	568	596	594	624	621	633
3007	490	506	515	523	538	553	562
3008	453	466	486	484	500	509	512
3009	515	531	546	563	577	575	603
3010	551	571	589	612	636	653	672
Mean	523.5	539.0	555.4	565.6	586.0	588.9	612.4
SD	53.0	52.5	55.8	56.4	64.9	64.0	70.1
N	10	10	10	10	10	10	8
%Diff	-8.8	-9.7	-9.0	-8.0	0.0	3.1	10.0

Day 91 represents data collected on Day 90 and 91.

Appendix 6

Individual Body Weights

32025165
Sex: Male Bodyweight (g)

1.49x10^12 vg	Day(s) Relative to Start Date						
Group 4	-1	7	14	21	28	35	42
4001	354	401	444	479	499	-	-
4002	332	363	397	421	439	-	-
4003	298	339	367	389	400	-	-
4004	297	333	375	409	427	-	-
4005	342	405	457	494	526	-	-
4006	358	398	437	476	506	534	556
4007	340	370	407	422	448	466	484
4008	354	388	432	454	489	519	540
4009	367	400	440	473	507	523	543
4010	391	429	466	497	520	553	568
4011	410	449	486	522	546	574	602
4012	359	385	425	445	472	491	519
4013	313	348	368	400	404	407	415
4014	320	345	374	397	415	435	452
4015	333	365	391	410	420	433	448
Mean	344.5	381.2	417.7	445.9	467.9	493.5	512.7
SD	31.5	33.6	38.2	42.3	48.8	56.3	60.4
N	15	15	15	15	15	10	10
%Diff	8.9	7.0	6.0	4.8	3.9	-5.9	-7.0

Day 91 represents data collected on Day 90 and 91.

Appendix 6

Individual Body Weights

32025165
Sex: Male Bodyweight (g)

1.49x10^12 vg Group 4	Day(s) Relative to Start Date						
	49	56	63	70	77	84	91
4001	-	-	-	-	-	-	-
4002	-	-	-	-	-	-	-
4003	-	-	-	-	-	-	-
4004	-	-	-	-	-	-	-
4005	-	-	-	-	-	-	-
4006	587	604	625	649	670	672	-
4007	498	505	527	532	544	550	-
4008	562	577	596	603	614	609	616
4009	554	578	590	593	594	613	625
4010	593	623	638	635	676	704	724
4011	617	640	669	685	701	700	729
4012	546	558	569	585	606	606	620
4013	436	451	472	490	501	532	537
4014	462	480	488	497	513	521	534
4015	453	465	474	476	486	479	496
Mean	530.8	548.1	564.8	574.5	590.5	598.6	610.1
SD	64.2	68.2	71.2	72.5	77.3	77.7	85.9
N	10	10	10	10	10	10	8
%Diff	-7.5	-8.1	-7.5	-6.5	0.7	4.8	9.6

Day 91 represents data collected on Day 90 and 91.

Appendix 6

Individual Body Weights

32025165
Sex: Female Bodyweight (g)

0 vg	Day(s) Relative to Start Date						
Group 1	-1	7	14	21	28	35	42
1501	209	225	227	231	241	-	-
1502	197	221	222	228	235	-	-
1503	207	228	253	260	257	-	-
1504	225	260	278	282	279	-	-
1505	183	196	197	215	218	-	-
1506	218	236	245	244	245	255	259
1507	228	246	258	255	270	282	284
1508	209	209	209	246	225	229	224
1509	217	230	242	244	243	254	258
1510	222	231	238	241	250	262	268
Mean	211.5	228.2	236.9	244.6	246.3	256.4	258.6
SD	13.7	17.8	24.0	18.5	18.8	19.0	22.0
N	10	10	10	10	10	5	5

Day 91 represents data collected on Day 90 and 91.

Appendix 6

Individual Body Weights

32025165
Sex: Female Bodyweight (g)

0 vg	Day(s) Relative to Start Date						
Group 1	49	56	63	70	77	84	91
1501	-	-	-	-	-	-	-
1502	-	-	-	-	-	-	-
1503	-	-	-	-	-	-	-
1504	-	-	-	-	-	-	-
1505	-	-	-	-	-	-	-
1506	258	262	264	274	265	270	274
1507	283	299	295	296	297	295	305
1508	234	248	238	240	236	240	242
1509	258	261	265	276	274	274	272
1510	273	272	276	284	282	286	284
Mean	261.2	268.4	267.6	274.0	270.8	273.0	275.4
SD	18.5	19.1	20.7	20.9	22.7	20.9	22.8
N	5	5	5	5	5	5	5

Day 91 represents data collected on Day 90 and 91.

Appendix 6

Individual Body Weights

32025165
Sex: Female Bodyweight (g)

1.49x10^11 vg	Day(s) Relative to Start Date						
Group 2	-1	7	14	21	28	35	42
2501	219	234	241	237	255	257	258
2502	243	243	255	262	261	259	266
2503	252	259	274	282	294	292	297
2504	221	231	231	229	240	254	247
2505	220	231	240	241	240	256	258
2506	279	299	311	318	332	340	344
2507	245	244	258	264	270	269	276
2508	241	254	253	259	271	275	272
2509	225	242	255	251	248	259	260
2510	250	263	262	254	273	286	284
Mean	239.5	250.0	258.0	259.7	268.4	274.7	276.2
SD	18.9	20.5	22.3	25.5	27.9	26.5	27.9
N	10	10	10	10	10	10	10
%Diff	13.2	9.6	8.9	6.2	9.0	7.1	6.8

Day 91 represents data collected on Day 90 and 91.

Appendix 6

Individual Body Weights

32025165
Sex: Female Bodyweight (g)

1.49x10^11 vg Group 2	Day(s) Relative to Start Date						
	49	56	63	70	77	84	91
2501	260	279	266	279	281	289	288
2502	270	280	279	282	279	290	288
2503	305	316	309	320	321	323	319
2504	250	262	264	269	268	272	276
2505	258	256	269	277	270	274	282
2506	353	353	365	367	361	359	387
2507	271	283	283	287	293	283	289
2508	283	292	297	305	304	307	311
2509	263	266	278	279	281	278	283
2510	275	293	293	300	289	299	298
Mean	278.8	288.0	290.3	296.5	294.7	297.4	302.1
SD	30.2	28.7	29.9	29.2	28.2	26.8	32.7
N	10	10	10	10	10	10	10
%Diff	6.7	7.3	8.5	8.2	8.8	8.9	9.7

Day 91 represents data collected on Day 90 and 91.

Appendix 6

Individual Body Weights

32025165
Sex: Female Bodyweight (g)

4.47x10^11 vg	Day(s) Relative to Start Date						
Group 3	-1	7	14	21	28	35	42
3501	278	301	306	316	314	329	311
3502	233	248	255	261	257	272	274
3503	246	264	273	267	264	279	283
3504	209	227	238	241	240	249	254
3505	232	233	244	249	253	257	262
3506	223	238	245	249	252	258	263
3507	219	237	247	246	259	266	273
3508	253	264	264	272	287	275	292
3509	210	218	224	222	232	240	237
3510	242	250	253	249	248	257	252
Mean	234.5	248.0	254.9	257.2	260.6	268.2	270.1
SD	21.2	23.8	22.5	25.0	23.8	24.5	21.4
N	10	10	10	10	10	10	10
%Diff	10.9	8.7	7.6	5.2	5.8	4.6	4.4

Day 91 represents data collected on Day 90 and 91.

Appendix 6

Individual Body Weights

32025165
Sex: Female Bodyweight (g)

4.47x10^11 vg Group 3	Day(s) Relative to Start Date 49	56	63	70	77	84	91
3501	316	340	336	353	367	371	366
3502	272	279	286	296	288	286	290
3503	284	287	294	306	301	296	308
3504	255	261	259	269	267	271	279
3505	262	275	279	288	276	288	283
3506	260	265	273	281	275	282	280
3507	277	274	274	286	285	285	286
3508	289	298	296	296	297	292	292
3509	256	255	252	258	251	259	259
3510	253	256	262	274	262	269	251
Mean	272.4	279.0	281.1	290.7	286.9	289.9	289.4
SD	19.8	25.4	24.1	26.1	32.1	30.7	31.4
N	10	10	10	10	10	10	10
%Diff	4.3	3.9	5.0	6.1	5.9	6.2	5.1

Day 91 represents data collected on Day 90 and 91.

Appendix 6

Individual Body Weights

32025165
Sex: Female Bodyweight (g)

1.49x10^12 vg<br>Group 4	Day(s) Relative to Start Date						
	-1	7	14	21	28	35	42
4501	193	196	215	227	251	-	-
4502	217	242	264	266	266	-	-
4503	197	223	229	233	235	-	-
4504	202	223	231	234	242	-	-
4505	211	236	254	253	254	-	-
4506	262	268	275	279	283	290	295
4507	233	249	251	253	255	268	269
4508	227	243	252	253	256	269	270
4509	258	264	275	273	283	289	286
4510	214	226	234	238	239	245	245
4511	223	236	246	243	247	257	259
4512	252	274	283	288	291	309	313
4513	235	249	249	248	254	264	265
4514	243	261	269	276	269	285	292
4515	231	253	257	262	261	274	263
Mean	226.5	242.9	252.3	255.1	259.1	275.0	275.7
SD	21.4	20.6	19.3	18.5	16.6	18.6	20.3
N	15	15	15	15	15	10	10
%Diff	7.1	6.4	6.5	4.3	5.2	7.3	6.6

Day 91 represents data collected on Day 90 and 91.

Appendix 6

Individual Body Weights

32025165
Sex: Female Bodyweight (g)

1.49x10^12 vg Group 4	Day(s) Relative to Start Date						
	49	56	63	70	77	84	91
4501	-	-	-	-	-	-	-
4502	-	-	-	-	-	-	-
4503	-	-	-	-	-	-	-
4504	-	-	-	-	-	-	-
4505	-	-	-	-	-	-	-
4506	295	302	302	308	311	327	316
4507	261	282	280	280	276	288	285
4508	271	272	277	285	285	285	287
4509	289	303	304	309	306	318	320
4510	242	251	262	264	265	268	268
4511	256	260	268	271	264	270	274
4512	313	324	325	330	322	325	323
4513	268	275	286	289	279	277	278
4514	290	277	295	302	293	307	303
4515	267	256	275	285	285	284	290
Mean	275.2	280.2	287.4	292.3	288.6	294.9	294.4
SD	21.2	23.3	19.1	19.9	19.4	22.5	19.9
N	10	10	10	10	10	10	10
%Diff	5.4	4.4	7.4	6.7	6.6	8.0	6.9

Day 91 represents data collected on Day 90 and 91.

Appendix 7

Individual Body Weight Gains (g)

32025165
Sex: Male Bodyweight Gain (Interval)

0 vg	Day(s) Relative to Start Date						
Group 1	-1 → 7	7 → 14	14 → 21	21 → 28	28 → 35	35 → 42	42 → 49
1001	50	44	34	31	-	-	-
1002	41	41	38	13	-	-	-
1003	45	34	46	19	-	-	-
1004	50	34	42	11	-	-	-
1005	27	38	21	7	-	-	-
1006	28	27	22	39	21	31	24
1007	24	47	29	29	25	27	23
1008	31	29	8	38	26	23	31
1009	46	33	31	29	30	22	15
1010	58	50	43	31	38	30	21
Mean	40.0	37.7	31.4	24.7	28.0	26.6	22.8
SD	11.7	7.7	11.8	11.4	6.4	4.0	5.8
N	10	10	10	10	5	5	5

Appendix 7

Individual Body Weight Gains (g)

32025165
Sex: Male Bodyweight Gain (Interval)

0 vg	Day(s) Relative to Start Date					
Group 1	49 → 56	56 → 63	63 → 70	70 → 77	77 → 84	84 → 91
1001	-	-	-	-	-	-
1002	-	-	-	-	-	-
1003	-	-	-	-	-	-
1004	-	-	-	-	-	-
1005	-	-	-	-	-	-
1006	28	16	13	7	-1	-
1007	21	18	8	17	13	10
1008	16	13	23	15	17	-5
1009	21	8	-12	-85	-45	0
1010	28	14	-11	-96	-58	11
Mean	22.8	13.8	4.2	-28.4	-14.8	4.0
SD	5.2	3.8	15.3	56.9	34.5	7.8
N	5	5	5	5	5	4

Appendix 7

Individual Body Weight Gains (g)

32025165
Sex: Male Bodyweight Gain (Interval)

1.49x10^11 vg	Day(s) Relative to Start Date						
Group 2	-1 → 7	7 → 14	14 → 21	21 → 28	28 → 35	35 → 42	42 → 49
2001	50	57	33	33	41	33	21
2002	26	23	25	27	20	10	14
2003	32	23	11	37	24	26	15
2004	27	28	15	29	23	14	16
2005	38	40	36	25	23	24	17
2006	38	39	11	24	27	16	24
2007	36	48	30	38	30	34	27
2008	42	42	26	27	31	13	23
2009	35	22	38	26	22	16	20
2010	34	33	33	27	29	18	19
Mean	35.8	35.5	25.8	29.3	27.0	20.4	19.6
SD	7.0	11.8	10.2	5.0	6.1	8.4	4.2
N	10	10	10	10	10	10	10

Appendix 7

Individual Body Weight Gains (g)

32025165
Sex: Male Bodyweight Gain (Interval)

1.49x10^11 vg Group 2	Day(s) Relative to Start Date					
	49 → 56	56 → 63	63 → 70	70 → 77	77 → 84	84 → 91
2001	26	5	25	31	9	-
2002	20	21	2	10	3	0
2003	20	7	-7	3	11	-20
2004	6	26	1	10	15	6
2005	13	2	23	10	16	7
2006	11	-4	20	18	-1	10
2007	21	27	3	22	17	6
2008	21	13	1	23	15	14
2009	18	12	7	7	-1	16
2010	27	7	6	23	11	10
Mean	18.3	11.6	8.1	15.7	9.5	5.4
SD	6.6	10.3	10.8	8.9	6.9	10.6
N	10	10	10	10	10	9

Appendix 7

Individual Body Weight Gains (g)

32025165
Sex: Male Bodyweight Gain (Interval)

4.47x10^11 vg	Day(s) Relative to Start Date						
Group 3	-1 → 7	7 → 14	14 → 21	21 → 28	28 → 35	35 → 42	42 → 49
3001	39	41	22	32	17	16	23
3002	26	35	31	36	33	25	20
3003	38	40	29	25	24	23	23
3004	45	51	35	24	36	24	28
3005	46	43	29	30	28	27	18
3006	18	24	24	26	27	25	13
3007	35	33	27	18	23	17	16
3008	27	22	18	17	15	12	15
3009	34	26	25	33	13	20	28
3010	40	38	30	19	29	22	16
Mean	34.8	35.3	27.0	26.0	24.5	21.1	20.0
SD	8.9	9.2	4.9	6.7	7.6	4.8	5.3
N	10	10	10	10	10	10	10

Appendix 7

Individual Body Weight Gains (g)

32025165
Sex: Male Bodyweight Gain (Interval)

4.47x10^11 vg	Day(s) Relative to Start Date					
Group 3	49 → 56	56 → 63	63 → 70	70 → 77	77 → 84	84 → 91
3001	7	4	13	13	1	-
3002	25	20	15	11	-10	-
3003	15	9	14	11	4	13
3004	20	23	8	23	1	13
3005	8	18	8	47	-3	10
3006	15	28	-2	30	-3	12
3007	16	9	8	15	15	9
3008	13	20	-2	16	9	3
3009	16	15	17	14	-2	28
3010	20	18	23	24	17	19
Mean	15.5	16.4	10.2	20.4	2.9	13.4
SD	5.4	7.3	7.9	11.3	8.5	7.4
N	10	10	10	10	10	8

Appendix 7

Individual Body Weight Gains (g)

32025165
Sex: Male Bodyweight Gain (Interval)

1.49x10^12 vg	Day(s) Relative to Start Date						
Group 4	-1 → 7	7 → 14	14 → 21	21 → 28	28 → 35	35 → 42	42 → 49
4001	47	43	35	20	-	-	-
4002	31	34	24	18	-	-	-
4003	41	28	22	11	-	-	-
4004	36	42	34	18	-	-	-
4005	63	52	37	32	-	-	-
4006	40	39	39	30	28	22	31
4007	30	37	15	26	18	18	14
4008	34	44	22	35	30	21	22
4009	33	40	33	34	16	20	11
4010	38	37	31	23	33	15	25
4011	39	37	36	24	28	28	15
4012	26	40	20	27	19	28	27
4013	35	20	32	4	3	8	21
4014	25	29	23	18	20	17	10
4015	32	26	19	10	13	15	5
Mean	36.7	36.5	28.1	22.0	20.8	19.2	18.1
SD	9.3	8.1	7.7	9.1	9.1	6.1	8.4
N	15	15	15	15	10	10	10

Appendix 7

Individual Body Weight Gains (g)

32025165
Sex: Male Bodyweight Gain (Interval)

1.49x10^12 vg	Day(s) Relative to Start Date					
Group 4	49 → 56	56 → 63	63 → 70	70 → 77	77 → 84	84 → 91
4001	-	-	-	-	-	-
4002	-	-	-	-	-	-
4003	-	-	-	-	-	-
4004	-	-	-	-	-	-
4005	-	-	-	-	-	-
4006	17	21	24	21	2	-
4007	7	22	5	12	6	-
4008	15	19	7	11	-5	7
4009	24	12	3	1	19	12
4010	30	15	-3	41	28	20
4011	23	29	16	16	-1	29
4012	12	11	16	21	0	14
4013	15	21	18	11	31	5
4014	18	8	9	16	8	13
4015	12	9	2	10	-7	17
Mean	17.3	16.7	9.7	16.0	8.1	14.6
SD	6.8	6.8	8.5	10.6	13.5	7.6
N	10	10	10	10	10	8

Appendix 7

Individual Body Weight Gains (g)

32025165
Sex: Female Bodyweight Gain (Interval)

0 vg	Day(s) Relative to Start Date						
Group 1	-1 → 7	7 → 14	14 → 21	21 → 28	28 → 35	35 → 42	42 → 49
1501	16	2	4	10	-	-	-
1502	24	1	6	7	-	-	-
1503	21	25	7	-3	-	-	-
1504	35	18	4	-3	-	-	-
1505	13	1	18	3	-	-	-
1506	18	9	-1	1	10	4	-1
1507	18	12	-3	15	12	2	-1
1508	0	0	37	-21	4	-5	10
1509	13	12	2	-1	11	4	0
1510	9	7	3	9	12	6	5
Mean	16.7	8.7	7.7	1.7	9.8	2.2	2.6
SD	9.3	8.3	11.7	10.0	3.3	4.3	4.8
N	10	10	10	10	5	5	5

Appendix 7

Individual Body Weight Gains (g)

32025165
Sex: Female Bodyweight Gain (Interval)

0 vg	Day(s) Relative to Start Date					
Group 1	49 → 56	56 → 63	63 → 70	70 → 77	77 → 84	84 → 91
1501	-	-	-	-	-	-
1502	-	-	-	-	-	-
1503	-	-	-	-	-	-
1504	-	-	-	-	-	-
1505	-	-	-	-	-	-
1506	4	2	10	-9	5	4
1507	16	-4	1	1	-2	10
1508	14	-10	2	-4	4	2
1509	3	4	11	-2	0	-2
1510	-1	4	8	-2	4	-2
Mean	7.2	-0.8	6.4	-3.2	2.2	2.4
SD	7.4	6.1	4.6	3.7	3.0	5.0
N	5	5	5	5	5	5

Appendix 7

Individual Body Weight Gains (g)

32025165
Sex: Female Bodyweight Gain (Interval)

1.49x10^11 vg	Day(s) Relative to Start Date						
Group 2	-1 → 7	7 → 14	14 → 21	21 → 28	28 → 35	35 → 42	42 → 49
2501	15	7	-4	18	2	1	2
2502	0	12	7	-1	-2	7	4
2503	7	15	8	12	-2	5	8
2504	10	0	-2	11	14	-7	3
2505	11	9	1	-1	16	2	0
2506	20	12	7	14	8	4	9
2507	-1	14	6	6	-1	7	-5
2508	13	-1	6	12	4	-3	11
2509	17	13	-4	-3	11	1	3
2510	13	-1	-8	19	13	-2	-9
Mean	10.5	8.0	1.7	8.7	6.3	1.5	2.6
SD	6.8	6.4	5.8	8.0	7.0	4.5	6.2
N	10	10	10	10	10	10	10

Appendix 7

Individual Body Weight Gains (g)

32025165
Sex: Female Bodyweight Gain (Interval)

1.49x10^11 vg	Day(s) Relative to Start Date					
Group 2	49 → 56	56 → 63	63 → 70	70 → 77	77 → 84	84 → 91
2501	19	-13	13	2	8	-1
2502	10	-1	3	-3	11	-2
2503	11	-7	11	1	2	-4
2504	12	2	5	-1	4	4
2505	-2	13	8	-7	4	8
2506	0	12	2	-6	-2	28
2507	12	0	4	6	-10	6
2508	9	5	8	-1	3	4
2509	3	12	1	2	-3	5
2510	18	0	7	-11	10	-1
Mean	9.2	2.3	6.2	-1.8	2.7	4.7
SD	7.0	8.5	3.9	5.1	6.4	9.1
N	10	10	10	10	10	10

Appendix 7

Individual Body Weight Gains (g)

32025165
Sex: Female Bodyweight Gain (Interval)

4.47x10^11 vg	Day(s) Relative to Start Date						
Group 3	-1 → 7	7 → 14	14 → 21	21 → 28	28 → 35	35 → 42	42 → 49
3501	23	5	10	-2	15	-18	5
3502	15	7	6	-4	15	2	-2
3503	18	9	-6	-3	15	4	1
3504	18	11	3	-1	9	5	1
3505	1	11	5	4	4	5	0
3506	15	7	4	3	6	5	-3
3507	18	10	-1	13	7	7	4
3508	11	0	8	15	-12	17	-3
3509	8	6	-2	10	8	-3	19
3510	8	3	-4	-1	9	-5	1
Mean	13.5	6.9	2.3	3.4	7.6	1.9	2.3
SD	6.5	3.6	5.3	6.9	7.9	9.2	6.4
N	10	10	10	10	10	10	10

Appendix 7

Individual Body Weight Gains (g)

32025165
Sex: Female Bodyweight Gain (Interval)

4.47x10^11 vg	Day(s) Relative to Start Date					
Group 3	49 → 56	56 → 63	63 → 70	70 → 77	77 → 84	84 → 91
3501	24	-4	17	14	4	-5
3502	7	7	10	-8	-2	4
3503	3	7	12	-5	-5	12
3504	6	-2	10	-2	4	8
3505	13	4	9	-12	12	-5
3506	5	8	8	-6	7	-2
3507	-3	0	12	-1	0	1
3508	9	-2	0	1	-5	0
3509	-1	-3	6	-7	8	0
3510	3	6	12	-12	7	-18
Mean	6.6	2.1	9.6	-3.8	3.0	-0.5
SD	7.7	4.7	4.5	7.6	5.8	8.2
N	10	10	10	10	10	10

Appendix 7

Individual Body Weight Gains (g)

32025165
Sex: Female Bodyweight Gain (Interval)

1.49x10^12 vg	Day(s) Relative to Start Date						
Group 4	-1 → 7	7 → 14	14 → 21	21 → 28	28 → 35	35 → 42	42 → 49
4501	3	19	12	24	-	-	-
4502	25	22	2	0	-	-	-
4503	26	6	4	2	-	-	-
4504	21	8	3	8	-	-	-
4505	25	18	-1	1	-	-	-
4506	6	7	4	4	7	5	0
4507	16	2	2	2	13	1	-8
4508	16	9	1	3	13	1	1
4509	6	11	-2	10	6	-3	3
4510	12	8	4	1	6	0	-3
4511	13	10	-3	4	10	2	-3
4512	22	9	5	3	18	4	0
4513	14	0	-1	6	10	1	3
4514	18	8	7	-7	16	7	-2
4515	22	4	5	-1	13	-11	4
Mean	16.3	9.4	2.8	4.0	11.2	0.7	-0.5
SD	7.4	6.1	3.8	6.8	4.1	5.0	3.6
N	15	15	15	15	10	10	10

Appendix 7

Individual Body Weight Gains (g)

32025165
Sex: Female Bodyweight Gain (Interval)

1.49x10^12 vg	Day(s) Relative to Start Date					
Group 4	49 → 56	56 → 63	63 → 70	70 → 77	77 → 84	84 → 91
4501	-	-	-	-	-	-
4502	-	-	-	-	-	-
4503	-	-	-	-	-	-
4504	-	-	-	-	-	-
4505	-	-	-	-	-	-
4506	7	0	6	3	16	-11
4507	21	-2	0	-4	12	-3
4508	1	5	8	0	0	2
4509	14	1	5	-3	12	2
4510	9	11	2	1	3	0
4511	4	8	3	-7	6	4
4512	11	1	5	-8	3	-2
4513	7	11	3	-10	-2	1
4514	-13	18	7	-9	14	-4
4515	-11	19	10	0	-1	6
Mean	5.0	7.2	4.9	-3.7	6.3	-0.5
SD	10.5	7.5	3.0	4.6	6.7	4.8
N	10	10	10	10	10	10

Appendix 8

Audited Draft Report

Study Phase: Ophthalmology

Testing Facility Study No. 32025165

Sponsor Reference No. 32025165

GLP

TESTING FACILITY:
Charles River Laboratories
54943 North Main Street
Mattawan, MI 49071
United States

TABLE OF CONTENTS

LIST OF APPENDICES 3
1. SUMMARY 4
2. MATERIALS AND METHODS 4
2.1. Computerized Systems and Electronic Data Archive Locations 4
2.2. Retention and Disposition of Study Phase Materials 5

LIST OF APPENDICES

Appendix 1 Ophthalmology Examinations and Tables..7

Appendix 8

1. SUMMARY

This study was conducted for the University of Massachusetts to determine the potential toxicity and biodistribution of the test article, scAAV9-CSA, for the treatment of Cockayne Syndrome when given as an intrathecal injection to rats once on Day 1, and to evaluation the potential reversibility of any findings following a 28-day and 90-day recovery period.

2. MATERIALS AND METHODS

Animals designated for ophthalmology evaluations were assigned to the study as indicated in Text Table 1.

Text Table 1
Experimental Design

Group No.	Test Article	Dose Level (vg)	Dose Volume (mL)	Dose Concentration (vg/mL)	Necropsy Day 29 (±1)		Necropsy Day 91 (±2)	
					No. of Males	No. of Females	No. of Males	No. of Females
1	Vehicle Control	0	0.1	0	5	5	5	5
2	scAAV9-CSA	1.49×10^{11}	0.1	1.49×10^{12}	-	-	10	10
3	scAAV9-CSA	4.47×10^{11}	0.1	4.47×10^{12}	-	-	10	10
4	scAAV9-CSA	1.49×10^{12}	0.1	1.49×10^{13}	5	5	10	10

- = Not applicable.

2.1. Computerized Systems and Electronic Data Archive Locations

Critical computerized systems used in the study phase and their respective electronic data archival locations are listed below. All computerized systems used in the conduct of this study have been validated; when a particular system has not satisfied all requirements, appropriate administrative and procedural controls were implemented to assure the quality and integrity of data.

Text Table 2
Critical Computer Systems

System Name	System Description	Archive Location
DocuSign™ eSignature	Collection of 21 CFR Part 11 compliant signature	Charles River Massachusetts Datacenter
eInfotree™	Excel Module for data collection with 21 CFR Part 11 compliance requirements, security, audit trail, and electronic signatures	Charles River Massachusetts Datacenter<br>Testing Facility
M-Files®	Document management, publishing, and collection of 21 CFR Part 11 compliant signatures	Charles River Massachusetts Datacenter
Provantis®	Toxicology, clinical pathology, pathology, test material receipt, and formulation data collection, reporting, and statistics system	Charles River Massachusetts Datacenter
SAS®	Statistical analysis	Charles River Massachusetts Datacenter

DRAFT, NOT FOR SUBMISSION

2.2. Retention and Disposition of Study Phase Materials

All study phase-specific physical raw data and documentation from this study phase are archived at the Testing Facility by no later than the date of Final Report issue.

Electronic data and documentation generated by this study phase are archived. Data generated by critical computerized systems are archived as listed in the critical computerized systems list. Electronic data archived in a cloud environment are under control of the Charles River Laboratories Archivist.

Appendix 8

DATA EXPLANATION PAGE

All Day(s) referenced throughout the outputs generated are based on Day 1 (first day of dose initiation).

Note: All abbreviations listed on this page may not be applicable to this report.

Abbreviation	Description
% Diff	% Difference from Group 1
tCtrl	Times control (fold change)
1F, 2F, 3F, 4F	Group 1 Female, Group 2 Female, Group 3 Female, Group 4 Female
1M, 2M, 3M, 4M	Group 1 Male, Group 2 Male, Group 3 Male, Group 4 Male
N	Number of values included in analysis
M, F	Male, Female
TK	Toxicokinetic
<, >	Out of range
--	Not scheduled to be performed/dead
- or .	Not applicable

Abbreviation	Description	Abbreviation	Description
Aqu	Aqueous	NA	Not applicable
BM	Bruch's membrane	NFL	Nerve fiber layer
CC	Choriocapillaris	No.	Animal number
CE	Corneal esthesiometry	NSF	No significant findings
C/NJ	Cortical/Nuclear junction	OCT	Optical coherence tomography
CS	Choroidal stroma	OD, R	Right eye
cSLO	Confocal scanning laser ophthalmoscopy	ONH	Optic nerve head
E	Exclude	ONL	Outer nuclear layer
ELM	External limiting membrane	OPL	Outer plexiform layer
ERG	Electroretinography	OS, L	Left eye
GCL	Ganglion cell layer	OU	Both eyes
GFP	Green fluorescent protein	PR1/2	Photoreceptor layers
ILM	Internal limiting membrane	RNFL	Retinal nerve fiber layer
INL	Inner nuclear layer	RPE	Retinal pigment epithelium
IOP	Intraocular Pressures	Vit	Vitreous
IPL	Inner plexiform layer	WNL	Within normal limits
Myd	Mydriatic		

OPHTHALMIC EXAMINATIONS

Abbreviation	Description	Abbreviation	Description
Ant	Anterior	Op	Opacity
Cap	Capsule	Pers	Persistent
Chor	Choroid	Post	Posterior
Diff	Diffuse	Pup	Pupil
Disch	Discharge	Ret	Retina
Rej	Reject	Vasc	Vascularization
Conj	Conjunctiva	Cont	Control
Multi	Multifocal		

Appendix 8

Appendix 1

Ophthalmological Examination

Study Number:	32025165	Study Interval	Pretest

Summary of Ophthalmological Findings: (please check all that apply)

☑ Observations noted were within limits of variation commonly encountered in animals of this sex, age, and strain.

☐ The observations noted were representative of pathology that would not be expected for this group of animals considering age, sex, and strain; trends in pathology would suggest that test material-related reactions had occurred.

☐ Other Comments (if applicable): ______

☐ Photographs taken

Ophthalmologist: LNS Date: 14-JUL-2025 (dd-mmm-yyyy)

Reviewed By (Initial/Date): AKW 15Jul2025

SFRM-TOX-50-14

Appendix 8

Appendix 1

Ophthalmological Examination

Study Number:	32025165	Study Interval	Terminal

Summary of Ophthalmological Findings: (please check all that apply)

- [x] Observations noted were within limits of variation commonly encountered in animals of this sex, age, and strain.
- [ ] The observations noted were representative of pathology that would not be expected for this group of animals considering age, sex, and strain; trends in pathology would suggest that test material-related reactions had occurred.
- [ ] Other Comments (if applicable): ______
- [ ] Photographs taken

Ophthalmologist: LNS Date: 13-AUG-2025
(dd-mmm-yyyy)

Reviewed By (Initial/Date): AKW 28Aug2025

SFRM-TOX-50-14

Appendix 8

Appendix 1

Ophthalmological Examination

Study Number:	32025165	Study Interval	Recovery

Summary of Ophthalmological Findings: (please check all that apply)

☑ Observations noted were within limits of variation commonly encountered in animals of this sex, age, and strain.

☐ The observations noted were representative of pathology that would not be expected for this group of animals considering age, sex, and strain; trends in pathology would suggest that test material-related reactions had occurred.

☐ Other Comments (if applicable): ____________________

☐ Photographs taken

Ophthalmologist: LNS Date: 21-OCT-2025
(dd-mmm-yyyy)

Reviewed By (Initial/Date): Ahw 23Oct2025

SFRM-TOX-50-14

Appendix 8

Appendix 1

Ophthalmological Examination

Study Number:	32025165	Study Interval	recovery

Summary of Ophthalmological Findings: (please check all that apply)

☑ Observations noted were within limits of variation commonly encountered in animals of this sex, age, and strain.

☐ The observations noted were representative of pathology that would not be expected for this group of animals considering age, sex, and strain; trends in pathology would suggest that test material-related reactions had occurred.

☐ Other Comments (if applicable): ______

☐ Photographs taken

Ophthalmologist: LNS Date: 28-OCT-2025 (dd-mmm-yyyy)

Reviewed By (Initial/Date): TJL 31Oct2025

SFRM-TOX-50-14

DRAFT, NOT FOR SUBMISSION

Appendix 8

Appendix 1

Individual Ophthalmology Observations

32025165

0 vg<br>Group 1<br>Sex: Male	Observation Type: All Types	Day(s) Relative to Start Date					
		-17	-11	-4	27	89	90
1001	Vitreous, Pers Hyaloid Artery, Right	.	.	X	.	.	.
	Vitreous, Hemorrhage, Right Inferior, 2 Slight	.	.	.	X	.	.
1002	Normal	.	.	X	X	.	.
1003	Normal	.	.	X	.	.	.
	Vitreous, Pers Hyaloid Artery, Right	.	.	.	X	.	.
1004	Normal	.	.	X	X	.	.
1005	Normal	.	.	X	X	.	.
1006	Normal	.	X	.	.	X	.
1007	Normal	.	X	.	.	X	.
1008	Normal	.	X	.	.	X	.
1009	Normal	.	X	.	.	X	.
1010	Normal	.	X	.	.	X	.

X=Present

DRAFT, NOT FOR SUBMISSION

Appendix 8

Appendix 1

Individual Ophthalmology Observations

32025165

1.49x10^11 vg Group 2 Sex: Male	Observation Type: All Types	Day(s) Relative to Start Date						
		-17	-11	-4	27	89	90	
2001	Normal	.	X	.	.	X	.	
2002	Normal	.	X	.	.	X	.	
2003	Normal	.	X	.	.	X	.	
2004	Normal	.	X	.	.	X	.	
2005	Normal	.	X	.	.	X	.	
2006	Normal	.	X	.	.	X	.	
2007	Normal	.	X	.	.	X	.	
2008	Normal	.	X	.	.	X	.	
2009	Normal	.	X	.	.	X	.	
2010	Normal	.	X	.	.	X	.	

X=Present

Appendix 8

Appendix 1

Individual Ophthalmology Observations

32025165

4.47x10^11 vg Group 3 Sex: Male	Observation Type: All Types	Day(s) Relative to Start Date						
		-17	-11	-4	27	89	90	
3001	Normal	.	X	.	.	X	.	
3002	Normal	.	X	.	.	X	.	
3003	Normal	.	X	.	.	X	.	
3004	Normal	.	X	.	.	X	.	
3005	Normal	.	X	.	.	X	.	
3006	Normal	.	X	.	.	X	.	
3007	Normal	.	X	.	.	X	.	
3008	Normal	.	X	.	.	X	.	
3009	Normal	.	X	.	.	X	.	
3010	Normal	.	X	.	.	X	.	

X=Present

Appendix 8

Appendix 1

Individual Ophthalmology Observations

32025165

1.49x10^12 vg Group 4 Sex: Male	Observation Type: All Types	Day(s) Relative to Start Date						
		-17	-11	-4	27	89	90	
4001	Normal	.	.	X	X	.	.	
4002	Normal	.	.	X	X	.	.	
4003	Normal	.	.	X	X	.	.	
4004	Normal	.	.	X	X	.	.	
4005	Normal	.	.	X	X	.	.	
4006	Normal	.	X	.	.	X	.	
4007	Normal	.	X	.	.	X	.	
4008	Normal	.	X	.	.	X	.	
4009	Normal	.	X	.	.	X	.	
4010	Normal	.	X	.	.	X	.	
4011	Normal	.	X	.	.	X	.	
4012	Normal	.	X	.	.	X	.	
4013	Normal	.	X	.	.	X	.	
4014	Normal	.	X	.	.	X	.	
4015	Normal	.	X	.	.	X	.	

X=Present

DRAFT, NOT FOR SUBMISSION

Appendix 8

Appendix 1

Individual Ophthalmology Observations

32025165

0 vg<br>Group 1<br>Sex: Female	Observation Type: All Types	Day(s) Relative to Start Date						
		-17	-11	-4	27	89	90	
1501	Normal	.	.	X	X	.	.	
1502	Normal	.	.	X	X	.	.	
1503	Normal	.	.	X	X	.	.	
1504	Normal	.	.	X	X	.	.	
1505	Normal	.	.	X	X	.	.	
1506	Normal	X	.	.	.	.	X	
1507	Normal	X	.	.	.	.	X	
1508	Normal	X	.	.	.	.	X	
1509	Normal	X	.	.	.	.	X	
1510	Normal	X	.	.	.	.	X	

X=Present

DRAFT, NOT FOR SUBMISSION

Appendix 8

Appendix 1

Individual Ophthalmology Observations

32025165

1.49x10^11 vg Group 2 Sex: Female	Observation Type: All Types	Day(s) Relative to Start Date						
		-17	-11	-4	27	89	90	
2501	Normal	X	.	.	.	.	X	
2502	Normal	X	.	.	.	.	X	
2503	Normal	X	.	.	.	.	X	
2504	Normal	X	.	.	.	.	X	
2505	Normal	X	.	.	.	.	X	
2506	Normal	X	.	.	.	.	X	
2507	Normal	X	.	.	.	.	X	
2508	Normal	X	.	.	.	.	X	
2509	Normal	X	.	.	.	.	X	
2510	Normal	X	.	.	.	.	X	

X=Present

Appendix 8

Appendix 1

Individual Ophthalmology Observations

32025165

4.47x10^11 vg Group 3 Sex: Female	Observation Type: All Types	Day(s) Relative to Start Date						
		-17	-11	-4	27	89	90	
3501	Normal	X	.	.	.	.	X	
3502	Normal	X	.	.	.	.	X	
3503	Normal	X	.	.	.	.	X	
3504	Normal	X	.	.	.	.	X	
3505	Normal	X	.	.	.	.	X	
3506	Normal	X	.	.	.	.	X	
3507	Normal	X	.	.	.	.	X	
3508	Normal	X	.	.	.	.	X	
3509	Normal	X	.	.	.	.	X	
3510	Normal	X	.	.	.	.	X	

X=Present

DRAFT, NOT FOR SUBMISSION

Appendix 8

Appendix 1

Individual Ophthalmology Observations

32025165

1.49x10^12 vg Group 4 Sex: Female	Observation Type: All Types	Day(s) Relative to Start Date						
		-17	-11	-4	27	89	90	
4501	Normal	.	.	X	X	.	.	
4502	Normal	.	.	X	X	.	.	
4503	Normal	.	.	X	X	.	.	
4504	Normal	.	.	X	X	.	.	
4505	Normal	.	.	X	X	.	.	
4506	Normal	X	.	.	.	.	X	
4507	Normal	X	.	.	.	.	X	
4508	Normal	X	.	.	.	.	X	
4509	Normal	X	.	.	.	.	X	
4510	Normal	X	.	.	.	.	X	
4511	Normal	X	.	.	.	.	X	
4512	Normal	X	.	.	.	.	X	
4513	Normal	X	.	.	.	.	X	
4514	Normal	X	.	.	.	.	X	
4515	Normal	X	.	.	.	.	X	

X=Present

DRAFT, NOT FOR SUBMISSION

Appendix 9

Individual Neurobehavioral Evaluation: Activity

32025165
Sex: Male

0 vg		Activity					
Group 1	Day(s) Relative to Start Date	Arousal/ Alertness	Posture/ BodyCarriage	Stereotypy (HC)	Stereotypy (OF)	Rearing	Appearance
1001	-1	0	0	0	0	10	0
	27	0	0	0	0	2	0
1002	-1	0	0	0	0	12	0
	27	0	0	0	0	14	0
1003	-1	0	0	0	0	1	0
	27	0	0	0	0	3	0
1004	-1	0	0	0	0	1	0
	27	0	0	0	0	0	0
1005	-1	0	0	0	0	6	0
	27	0	0	0	0	0	0
1006	-1	0	0	0	0	9	0
	27	0	0	0	0	9	0
	88	0	0	0	0	6	0
1007	-1	0	0	0	0	4	0
	27	0	0	0	0	3	0
	88	-1	0	0	0	2	0
1008	-1	0	0	0	0	12	0
	27	0	0	0	0	9	0
	88	0	0	0	0	13	0

Day 27 represents data collected from Day 27 to 32.

Appendix 9

Individual Neurobehavioral Evaluation: Activity

32025165
Sex: Male

0 vg		Activity					
Group 1	Day(s) Relative to Start Date	Arousal/ Alertness	Posture/ BodyCarriage	Stereotypy (HC)	Stereotypy (OF)	Rearing	Appearance
1009	-1	0	0	0	0	2	0
	27	0	0	0	0	10	0
	88	0	0	0	0	4	0
1010	-1	0	0	0	0	6	0
	27	0	0	0	0	5	0
	88	0	0	0	0	5	1

Day 27 represents data collected from Day 27 to 32.

Appendix 9

Individual Neurobehavioral Evaluation: Activity

32025165
Sex: Male

1.49x10^11 vg Group 2	Day(s) Relative to Start Date	Activity					
		Arousal/ Alertness	Posture/ BodyCarriage	Stereotypy (HC)	Stereotypy (OF)	Rearing	Appearance
2001	-1	0	0	0	0	6	0
	27	0	0	0	0	5	0
	88	0	0	0	0	7	0
2002	-1	0	0	0	0	7	0
	27	-1	0	0	0	2	0
	88	0	0	0	0	1	0
2003	-1	0	0	0	0	10	0
	27	0	0	0	0	8	0
	88	0	0	0	0	8	0
2004	-1	0	0	0	0	4	0
	27	0	0	0	0	12	0
	88	0	0	0	0	13	0
2005	-1	0	0	0	0	6	0
	27	-1	0	0	0	4	0
	88	0	0	0	0	4	0
2006	-1	0	0	0	0	7	0
	27	0	0	0	0	18	0
	88	0	0	0	0	8	0
2007	-1	0	0	0	0	11	0
	27	0	0	0	0	7	0
	88	0	0	0	0	10	0

Day 27 represents data collected from Day 27 to 32.

Appendix 9

Individual Neurobehavioral Evaluation: Activity

32025165
Sex: Male

1.49x10^11 vg Group 2	Day(s) Relative to Start Date	Activity					
		Arousal/ Alertness	Posture/ BodyCarriage	Stereotypy (HC)	Stereotypy (OF)	Rearing	Appearance
2008	-1	0	0	0	0	6	0
	27	0	0	0	0	7	0
	88	-1	0	0	0	3	0
2009	-1	0	0	0	0	7	0
	27	0	0	0	0	10	0
	88	0	0	0	0	11	0
2010	-1	0	0	0	0	4	0
	27	0	0	0	0	3	0
	88	-1	0	0	0	2	0

Day 27 represents data collected from Day 27 to 32.

Appendix 9

Individual Neurobehavioral Evaluation: Activity

32025165
Sex: Male

4.47x10^11 vg Group 3	Day(s) Relative to Start Date	Activity					
		Arousal/ Alertness	Posture/ BodyCarriage	Stereotypy (HC)	Stereotypy (OF)	Rearing	Appearance
3001	-1	0	0	0	0	6	0
	27	0	0	0	0	7	0
	88	0	0	0	0	6	0
3002	-1	0	0	0	0	8	0
	27	-1	0	0	0	0	0
	88	-1	0	0	0	3	0
3003	-1	0	0	0	0	9	0
	27	0	0	0	0	8	0
	88	-1	0	0	0	0	0
3004	-1	0	0	0	0	2	0
	27	-1	0	0	0	0	0
	88	-1	0	0	0	0	0
3005	-1	0	0	0	0	5	0
	27	0	0	0	0	7	0
	88	0	0	0	0	4	0
3006	-1	0	0	0	0	6	0
	27	0	0	0	0	12	0
	88	0	0	0	0	4	0
3007	-1	0	0	0	0	2	0
	27	-1	0	0	0	0	0
	88	-1	0	0	0	1	0

Day 27 represents data collected from Day 27 to 32.

Appendix 9

Individual Neurobehavioral Evaluation: Activity

32025165
Sex: Male

4.47x10^11 vg Group 3	Day(s) Relative to Start Date	Activity					
		Arousal/ Alertness	Posture/ BodyCarriage	Stereotypy (HC)	Stereotypy (OF)	Rearing	Appearance
3008	-1	0	0	0	0	4	0
	27	-1	0	0	0	0	0
	88	-1	0	0	0	3	0
3009	-1	0	0	0	0	3	0
	27	0	0	0	0	7	0
	88	0	0	0	0	8	0
3010	-1	0	0	0	0	3	0
	27	-1	0	0	0	2	0
	88	-1	0	0	0	3	0

Day 27 represents data collected from Day 27 to 32.

Appendix 9

Individual Neurobehavioral Evaluation: Activity

32025165
Sex: Male

1.49x10^12 vg Group 4	Day(s) Relative to Start Date	Activity					
		Arousal/ Alertness	Posture/ BodyCarriage	Stereotypy (HC)	Stereotypy (OF)	Rearing	Appearance
4001	-1	0	0	0	0	7	0
	27	0	0	0	0	0	0
4002	-1	0	0	0	0	11	0
	27	0	0	0	0	4	0
4003	-1	0	0	0	0	7	0
	27	0	0	0	0	9	0
4004	-1	0	0	0	0	12	0
	27	0	0	0	0	4	0
4005	-1	0	0	0	0	9	0
	27	0	0	0	0	9	0
4006	-1	0	0	0	0	7	0
	27	0	0	0	0	4	0
	88	0	0	0	0	6	0
4007	-1	0	0	0	0	10	0
	27	0	0	0	0	10	0
	88	0	0	0	0	13	0
4008	-1	0	0	0	0	7	0
	27	-1	0	0	0	1	0
	88	0	0	0	0	6	0

Day 27 represents data collected from Day 27 to 32.

Appendix 9

Individual Neurobehavioral Evaluation: Activity

32025165
Sex: Male

1.49x10^12 vg Group 4	Day(s) Relative to Start Date	Activity					
		Arousal/ Alertness	Posture/ BodyCarriage	Stereotypy (HC)	Stereotypy (OF)	Rearing	Appearance
4009	-1	0	0	0	0	6	0
	27	0	0	0	0	5	0
	88	0	0	0	0	3	0
4010	-1	0	0	0	0	3	0
	27	0	0	0	0	13	0
	88	0	0	0	0	8	0
4011	-1	0	0	0	0	7	0
	27	-1	0	0	0	1	0
	88	0	0	0	0	3	0
4012	-1	0	0	0	0	7	0
	27	0	0	0	0	15	0
	88	1	0	0	0	15	0
4013	-1	0	0	0	0	3	0
	27	0	0	0	0	7	0
	88	0	0	0	0	8	0
4014	-1	0	0	0	0	2	0
	27	-1	0	0	0	0	0
	88	-1	0	0	0	3	0
4015	-1	0	0	0	0	11	0
	27	-1	0	0	0	2	0
	88	-1	0	0	0	4	0

Day 27 represents data collected from Day 27 to 32.

Appendix 9

Individual Neurobehavioral Evaluation: Activity

32025165
Sex: Female

0 vg		Activity					
Group 1	Day(s) Relative to Start Date	Arousal/ Alertness	Posture/ BodyCarriage	Stereotypy (HC)	Stereotypy (OF)	Rearing	Appearance
1501	-1	0	0	0	0	18	0
	27	0	0	0	0	13	0
1502	-1	0	0	0	0	15	0
	27	0	0	0	0	20	0
1503	-1	0	0	0	0	11	0
	27	0	0	0	0	10	0
1504	-1	0	0	0	0	7	0
	27	0	0	0	0	8	0
1505	-1	0	0	0	0	15	0
	27	0	0	0	0	11	0
1506	-1	0	0	0	0	12	0
	27	0	0	0	0	13	0
	89	0	0	0	0	10	0
1507	-1	0	0	0	0	10	0
	27	0	0	0	0	6	0
	89	0	0	0	0	12	0
1508	-1	0	0	0	0	9	0
	27	0	0	0	0	4	0
	89	0	0	0	0	8	0

Day 27 represents data collected from Day 27 to 32.

Appendix 9

Individual Neurobehavioral Evaluation: Activity

32025165
Sex: Female

0 vg		Activity					
Group 1	Day(s) Relative to Start Date	Arousal/ Alertness	Posture/ BodyCarriage	Stereotypy (HC)	Stereotypy (OF)	Rearing	Appearance
1509	-1	0	0	0	0	11	0
	27	0	0	0	0	11	0
	89	0	0	0	0	9	0
1510	-1	0	0	0	0	11	0
	27	0	0	0	0	1	0
	89	0	0	0	0	8	0

Day 27 represents data collected from Day 27 to 32.

Appendix 9

Individual Neurobehavioral Evaluation: Activity

32025165
Sex: Female

1.49x10^11 vg Group 2	Day(s) Relative to Start Date	Activity					
		Arousal/ Alertness	Posture/ BodyCarriage	Stereotypy (HC)	Stereotypy (OF)	Rearing	Appearance
2501	-1	0	0	0	0	9	0
	27	0	0	0	0	13	0
	89	0	0	0	0	13	0
2502	-1	0	0	0	0	8	0
	27	0	0	0	0	8	0
	89	0	0	0	0	6	0
2503	-1	0	0	0	0	9	0
	27	0	0	0	0	14	0
	89	0	0	0	0	7	0
2504	-1	0	0	0	0	8	0
	27	0	0	0	0	10	0
	89	0	0	0	0	10	0
2505	-1	0	0	0	0	7	0
	27	0	0	0	0	3	0
	89	0	0	0	0	9	0
2506	-1	0	0	0	0	9	0
	27	0	0	0	0	9	0
	89	0	0	0	0	7	0
2507	-1	0	0	0	0	4	0
	27	-1	0	0	0	0	0
	89	0	0	0	0	11	0

Day 27 represents data collected from Day 27 to 32.

Appendix 9

Individual Neurobehavioral Evaluation: Activity

32025165

Sex: Female

1.49x10^11 vg Group 2	Day(s) Relative to Start Date	Activity					
		Arousal/ Alertness	Posture/ BodyCarriage	Stereotypy (HC)	Stereotypy (OF)	Rearing	Appearance
2508	-1	0	0	0	0	9	0
	27	0	0	0	0	10	0
	89	0	0	0	0	10	0
2509	-1	0	0	0	0	8	0
	27	0	0	0	0	8	0
	89	0	0	0	0	10	0
2510	-1	0	0	0	0	11	0
	27	0	0	0	0	13	0
	89	0	0	0	0	15	0

Day 27 represents data collected from Day 27 to 32.

Appendix 9

Individual Neurobehavioral Evaluation: Activity

32025165
Sex: Female

4.47x10^11 vg<br>Group 3	Day(s) Relative to Start Date	Activity					
		Arousal/ Alertness	Posture/ BodyCarriage	Stereotypy (HC)	Stereotypy (OF)	Rearing	Appearance
3501	-1	0	0	0	0	8	0
	27	0	0	0	0	8	0
	89	0	0	0	0	12	0
3502	-1	0	0	0	0	11	0
	27	0	0	0	0	0	0
	89	0	0	0	0	6	0
3503	-1	0	0	0	0	9	0
	27	0	0	0	0	5	0
	89	0	0	0	0	5	0
3504	-1	0	0	0	0	9	0
	27	0	0	0	0	7	0
	89	0	0	0	0	12	0
3505	-1	0	0	0	0	6	0
	27	-1	0	0	0	2	0
	89	0	0	0	0	2	0
3506	-1	0	0	0	0	9	0
	27	0	0	0	0	17	0
	89	0	0	0	0	9	0
3507	-1	0	0	0	0	12	0
	27	0	0	0	0	8	0
	89	0	0	0	0	13	0

Day 27 represents data collected from Day 27 to 32.

Appendix 9

Individual Neurobehavioral Evaluation: Activity

32025165
Sex: Female

4.47x10^11 vg<br>Group 3	Day(s) Relative to Start Date	Activity					
		Arousal/ Alertness	Posture/ BodyCarriage	Stereotypy (HC)	Stereotypy (OF)	Rearing	Appearance
3508	-1	0	0	0	0	15	0
	27	0	0	0	0	18	0
	89	0	0	0	0	13	0
3509	-1	0	0	0	0	5	0
	27	0	-1	0	0	1	0
	89	0	0	0	0	2	0
3510	-1	0	0	0	0	10	0
	27	0	0	0	0	6	0
	89	-1	0	0	0	1	0

Day 27 represents data collected from Day 27 to 32.

Appendix 9

Individual Neurobehavioral Evaluation: Activity

32025165
Sex: Female

1.49x10^12 vg Group 4	Day(s) Relative to Start Date	Activity					
		Arousal/ Alertness	Posture/ BodyCarriage	Stereotypy (HC)	Stereotypy (OF)	Rearing	Appearance
4501	-1	0	0	0	0	7	0
	27	0	0	0	0	2	0
4502	-1	0	0	0	0	13	0
	27	0	0	0	0	14	0
4503	-1	0	0	0	0	10	0
	27	0	0	0	0	6	0
4504	-1	0	0	0	0	8	0
	27	0	0	0	0	10	0
4505	-1	0	0	0	0	13	0
	27	0	0	0	0	11	0
4506	-1	0	0	0	0	11	0
	27	0	0	0	0	8	0
	89	0	0	0	0	9	0
4507	-1	0	0	0	0	10	0
	27	0	0	0	0	10	0
	89	0	0	0	0	13	0
4508	-1	0	0	0	0	4	0
	27	-1	0	0	0	2	0
	89	0	0	0	0	4	0

Day 27 represents data collected from Day 27 to 32.

Appendix 9

Individual Neurobehavioral Evaluation: Activity

32025165
Sex: Female

1.49x10^12 vg Group 4	Day(s) Relative to Start Date	Activity					
		Arousal/ Alertness	Posture/ BodyCarriage	Stereotypy (HC)	Stereotypy (OF)	Rearing	Appearance
4509	-1	0	0	0	0	11	0
	27	0	0	0	0	18	0
	89	0	0	0	0	14	0
4510	-1	0	0	0	0	10	0
	27	0	0	0	0	7	0
	89	0	0	0	0	10	0
4511	-1	0	0	0	0	14	0
	27	0	0	0	0	9	0
	89	0	0	0	0	11	0
4512	-1	0	0	0	0	10	0
	27	0	0	0	0	7	0
	89	0	0	0	0	9	0
4513	-1	0	0	0	0	18	0
	27	0	0	0	0	13	0
	89	0	0	0	0	10	0
4514	-1	0	0	0	0	12	0
	27	0	0	0	0	4	0
	89	0	0	0	0	11	0
4515	-1	0	0	0	0	10	0
	27	0	0	0	0	9	0
	89	0	0	0	0	8	0

Day 27 represents data collected from Day 27 to 32.

Appendix 10

Individual Neurobehavioral Evaluation: Autonomic

32025165
Sex: Male

0 vg		Autonomic					
Group 1	Day(s) Relative to Start Date	Exophthalmus	Lacrimation	Erected Fur	Pupil Response	Salivation	Defecation
1001	-1	0	0	0	0	0	-1
	27	0	0	0	0	0	-1
1002	-1	0	0	0	0	0	-1
	27	0	0	0	0	0	-1
1003	-1	0	0	0	0	0	0
	27	0	0	0	0	0	0
1004	-1	0	0	0	0	0	-1
	27	0	0	0	0	0	-1
1005	-1	0	0	0	0	0	0
	27	0	0	0	0	0	-1
1006	-1	0	0	0	0	0	-1
	27	0	0	0	0	0	-1
	88	0	0	0	0	0	-1
1007	-1	0	0	0	0	0	-1
	27	0	0	0	0	0	0
	88	0	0	0	0	0	0
1008	-1	0	0	0	0	0	-1
	27	0	0	0	0	0	-1
	88	0	0	0	0	0	-1

Day 27 represents data collected from Day 27 to 32.

Appendix 10

Individual Neurobehavioral Evaluation: Autonomic

32025165

Sex: Male

0 vg		Autonomic	
Group 1	Day(s) Relative to Start Date	Palpebral Closure (HC)	Palpebral Closure (OF)
1001	-1	0	0
	27	0	0
1002	-1	0	0
	27	0	0
1003	-1	0	0
	27	0	0
1004	-1	0	0
	27	0	0
1005	-1	0	0
	27	0	0
1006	-1	0	0
	27	0	0
	88	0	0
1007	-1	0	0
	27	0	0
	88	0	0
1008	-1	0	0
	27	0	0
	88	0	0

Day 27 represents data collected from Day 27 to 32.

Appendix 10

Individual Neurobehavioral Evaluation: Autonomic

32025165
Sex: Male

0 vg		Autonomic					
Group 1	Day(s) Relative to Start Date	Exophthalmus	Lacrimation	Erected Fur	Pupil Response	Salivation	Defecation
1009	-1	0	0	0	0	0	-1
	27	0	0	0	0	0	-1
	88	0	0	0	0	0	-1
1010	-1	0	0	0	0	0	0
	27	0	0	0	0	0	0
	88	0	0	0	0	0	-1

Day 27 represents data collected from Day 27 to 32.

Appendix 10

Individual Neurobehavioral Evaluation: Autonomic

32025165
Sex: Male

0 vg		Autonomic	
Group 1	Day(s) Relative to Start Date	Palpebral Closure (HC)	Palpebral Closure (OF)
1009	-1	0	0
	27	0	0
	88	0	0
1010	-1	0	0
	27	0	0
	88	0	0

Day 27 represents data collected from Day 27 to 32.

Appendix 10

Individual Neurobehavioral Evaluation: Autonomic

32025165
Sex: Male

1.49x10^11 vg Group 2	Day(s) Relative to Start Date	Autonomic					
		Exophthalmus	Lacrimation	Erected Fur	Pupil Response	Salivation	Defecation
2001	-1	0	0	0	0	0	0
	27	0	0	0	0	0	0
	88	0	0	0	0	0	-1
2002	-1	0	0	0	0	0	0
	27	0	0	0	0	0	0
	88	0	0	0	0	0	0
2003	-1	0	0	0	0	0	-1
	27	0	0	0	0	0	-1
	88	0	0	0	0	0	-1
2004	-1	0	0	1	0	0	-1
	27	0	0	0	0	0	-1
	88	0	0	0	0	0	-1
2005	-1	0	0	0	0	0	0
	27	0	0	0	0	0	-1
	88	0	0	0	0	0	-1
2006	-1	0	0	0	0	0	0
	27	0	0	0	0	0	-1
	88	0	0	0	0	0	-1
2007	-1	0	0	0	0	0	-1
	27	0	0	0	0	0	-1
	88	0	0	0	0	0	-1

Day 27 represents data collected from Day 27 to 32.

Appendix 10

Individual Neurobehavioral Evaluation: Autonomic

32025165
Sex: Male

1.49x10^11 vg Group 2	Day(s) Relative to Start Date	Autonomic Palpebral Closure (HC)	Autonomic Palpebral Closure (OF)
2001	-1	0	0
	27	0	0
	88	0	0
2002	-1	0	0
	27	0	0
	88	0	0
2003	-1	0	0
	27	0	0
	88	0	0
2004	-1	0	0
	27	0	0
	88	0	0
2005	-1	0	0
	27	0	0
	88	0	0
2006	-1	0	0
	27	0	0
	88	0	0
2007	-1	0	0
	27	0	0
	88	0	0

Day 27 represents data collected from Day 27 to 32.

Appendix 10

Individual Neurobehavioral Evaluation: Autonomic

32025165
Sex: Male

1.49x10^11 vg Group 2	Day(s) Relative to Start Date	Autonomic					
		Exophthalmus	Lacrimation	Erected Fur	Pupil Response	Salivation	Defecation
2008	-1	0	0	0	0	0	-1
	27	0	0	0	0	0	0
	88	0	0	0	0	0	0
2009	-1	0	0	0	0	0	-1
	27	0	0	0	0	0	-1
	88	0	0	0	0	0	-1
2010	-1	0	0	0	0	0	-1
	27	0	0	0	0	0	-1
	88	0	0	0	0	0	0

Day 27 represents data collected from Day 27 to 32.

Appendix 10

Individual Neurobehavioral Evaluation: Autonomic

32025165

Sex: Male

1.49x10^11 vg<br>Group 2	Day(s) Relative to Start Date	Autonomic	
		Palpebral Closure (HC)	Palpebral Closure (OF)
2008	-1	0	0
	27	0	0
	88	0	0
2009	-1	0	0
	27	0	0
	88	0	0
2010	-1	0	0
	27	0	0
	88	0	0

Day 27 represents data collected from Day 27 to 32.

Appendix 10

Individual Neurobehavioral Evaluation: Autonomic

32025165
Sex: Male

4.47x10^11 vg Group 3	Day(s) Relative to Start Date	Autonomic					
		Exophthalmus	Lacrimation	Erected Fur	Pupil Response	Salivation	Defecation
3001	-1	0	0	0	0	0	-1
	27	0	0	0	0	0	-1
	88	0	0	0	0	0	-1
3002	-1	0	0	0	0	0	0
	27	0	0	0	0	0	0
	88	0	0	0	0	0	-1
3003	-1	0	0	0	0	0	-1
	27	0	0	0	0	0	-1
	88	0	0	0	0	0	0
3004	-1	0	0	0	0	0	0
	27	0	0	0	0	0	0
	88	0	0	0	0	0	0
3005	-1	0	0	0	0	0	0
	27	0	0	0	0	0	-1
	88	0	0	0	0	0	-1
3006	-1	0	0	0	0	0	-1
	27	0	0	0	0	0	-1
	88	0	0	0	0	0	-1
3007	-1	0	0	1	0	0	-1
	27	0	0	0	0	0	-1
	88	0	0	0	0	0	-1

Day 27 represents data collected from Day 27 to 32.

Appendix 10

Individual Neurobehavioral Evaluation: Autonomic

32025165
Sex: Male

4.47x10^11 vg Group 3	Day(s) Relative to Start Date	Autonomic	
		Palpebral Closure (HC)	Palpebral Closure (OF)
3001	-1	0	0
	27	0	0
	88	0	0
3002	-1	0	0
	27	0	0
	88	0	0
3003	-1	0	0
	27	0	0
	88	0	0
3004	-1	0	0
	27	0	0
	88	0	0
3005	-1	0	0
	27	0	0
	88	0	0
3006	-1	0	0
	27	0	0
	88	0	0
3007	-1	0	0
	27	0	0
	88	0	0

Day 27 represents data collected from Day 27 to 32.

Appendix 10

Individual Neurobehavioral Evaluation: Autonomic

32025165
Sex: Male

4.47x10^11 vg Group 3	Day(s) Relative to Start Date	Autonomic					
		Exophthalmus	Lacrimation	Erected Fur	Pupil Response	Salivation	Defecation
3008	-1	0	0	0	0	0	-1
	27	0	0	0	0	0	0
	88	0	0	0	0	0	0
3009	-1	0	0	0	0	0	0
	27	0	0	0	0	0	0
	88	0	0	0	0	0	0
3010	-1	0	0	0	0	0	-1
	27	0	0	0	0	0	-1
	88	0	0	0	0	0	-1

Day 27 represents data collected from Day 27 to 32.

Appendix 10

Individual Neurobehavioral Evaluation: Autonomic

32025165

Sex: Male

4.47x10^11 vg<br>Group 3	Day(s) Relative to Start Date	Autonomic	
		Palpebral Closure (HC)	Palpebral Closure (OF)
3008	-1	0	0
	27	0	0
	88	0	0
3009	-1	0	0
	27	0	0
	88	0	0
3010	-1	0	0
	27	0	0
	88	0	0

Day 27 represents data collected from Day 27 to 32.

Appendix 10

Individual Neurobehavioral Evaluation: Autonomic

32025165

Sex: Male

1.49x10^12 vg Group 4	Day(s) Relative to Start Date	Autonomic					
		Exophthalmus	Lacrimation	Erected Fur	Pupil Response	Salivation	Defecation
4001	-1	0	0	0	0	0	-1
	27	0	0	0	0	0	-1
4002	-1	0	0	0	0	0	0
	27	0	0	0	0	0	-1
4003	-1	0	0	0	0	0	-1
	27	0	0	0	0	0	-1
4004	-1	0	0	0	0	0	-1
	27	0	0	0	1 [a]	0	-1
4005	-1	0	0	0	0	0	-1
	27	0	0	0	0	0	-1
4006	-1	0	0	0	0	0	-1
	27	0	0	0	0	0	-1
	88	0	0	0	0	0	-1
4007	-1	0	0	0	0	0	-1
	27	0	0	0	0	0	-1
	88	0	0	0	0	0	-1
4008	-1	0	0	0	0	0	-1
	27	0	0	0	0	0	-1
	88	0	0	0	0	0	-1

[a] [RC:lack of constriction of pupils]
Day 27 represents data collected from Day 27 to 32.

Appendix 10

Individual Neurobehavioral Evaluation: Autonomic

32025165

Sex: Male

1.49x10^12 vg<br>Group 4	Day(s) Relative to Start Date	Autonomic	
		Palpebral Closure (HC)	Palpebral Closure (OF)
4001	-1	0	0
	27	0	0
4002	-1	0	0
	27	0	0
4003	-1	0	0
	27	0	0
4004	-1	0	0
	27	0	0
4005	-1	0	0
	27	0	0
4006	-1	0	0
	27	0	0
	88	0	0
4007	-1	0	0
	27	0	0
	88	0	0
4008	-1	0	0
	27	0	0
	88	0	0

Day 27 represents data collected from Day 27 to 32.

Appendix 10

Individual Neurobehavioral Evaluation: Autonomic

32025165

Sex: Male

1.49x10^12 vg Group 4	Day(s) Relative to Start Date	Autonomic					
		Exophthalmus	Lacrimation	Erected Fur	Pupil Response	Salivation	Defecation
4009	-1	0	0	0	0	0	-1
	27	0	0	0	0	0	-1
	88	0	0	0	0	0	-1
4010	-1	0	0	0	0	0	0
	27	0	0	0	0	0	0
	88	0	0	0	0	0	-1
4011	-1	0	0	0	0	0	0
	27	0	0	0	0	0	0
	88	0	0	0	0	0	-1
4012	-1	0	0	0	0	0	-1
	27	0	0	0	0	0	-1
	88	0	0	0	0	0	0
4013	-1	0	0	0	0	0	0
	27	0	0	0	0	0	-1
	88	0	0	0	0	0	-1
4014	-1	0	0	0	0	0	0
	27	0	0	0	0	0	0
	88	0	0	0	0	0	0
4015	-1	0	0	0	0	0	0
	27	0	0	0	0	0	0
	88	0	0	0	0	0	0

Day 27 represents data collected from Day 27 to 32.

Appendix 10

Individual Neurobehavioral Evaluation: Autonomic

32025165
Sex: Male

1.49x10^12 vg Group 4	Day(s) Relative to Start Date	Autonomic	
		Palpebral Closure (HC)	Palpebral Closure (OF)
4009	-1	0	0
	27	0	0
	88	0	0
4010	-1	0	0
	27	0	0
	88	0	0
4011	-1	0	0
	27	1	0
	88	0	0
4012	-1	0	0
	27	0	0
	88	0	0
4013	-1	0	0
	27	0	0
	88	0	0
4014	-1	0	0
	27	0	0
	88	0	0
4015	-1	0	0
	27	0	0
	88	0	0

Day 27 represents data collected from Day 27 to 32.

Appendix 10

Individual Neurobehavioral Evaluation: Autonomic

32025165
Sex: Female

0 vg		Autonomic					
Group 1	Day(s) Relative to Start Date	Exophthalmus	Lacrimation	Erected Fur	Pupil Response	Salivation	Defecation
1501	-1	0	0	0	0	0	-1
	27	0	0	0	0	0	-1
1502	-1	0	0	0	0	0	-1
	27	0	0	0	0	0	-1
1503	-1	0	0	0	0	0	-1
	27	0	0	0	1 [a]	0	-1
1504	-1	0	0	0	0	0	-1
	27	0	0	0	0	0	-1
1505	-1	0	0	0	0	0	-1
	27	0	0	0	0	0	-1
1506	-1	0	0	0	0	0	-1
	27	0	0	0	0	0	0
	89	0	0	0	0	0	-1
1507	-1	0	0	0	0	0	-1
	27	0	0	0	0	0	-1
	89	0	0	0	0	0	-1
1508	-1	0	0	0	0	0	-1
	27	0	0	0	0	0	0
	89	0	0	0	0	0	-1

[a] [RC:lack of constriction of pupils]
Day 27 represents data collected from Day 27 to 32.

Appendix 10

Individual Neurobehavioral Evaluation: Autonomic

32025165
Sex: Female

0 vg		Autonomic	
Group 1	Day(s) Relative to Start Date	Palpebral Closure (HC)	Palpebral Closure (OF)
1501	-1	0	0
	27	0	0
1502	-1	0	0
	27	0	0
1503	-1	0	0
	27	0	0
1504	-1	0	0
	27	0	0
1505	-1	0	0
	27	0	0
1506	-1	0	0
	27	0	0
	89	0	0
1507	-1	0	0
	27	0	0
	89	0	0
1508	-1	0	0
	27	0	0
	89	0	0

Day 27 represents data collected from Day 27 to 32.

Appendix 10

Individual Neurobehavioral Evaluation: Autonomic

32025165
Sex: Female

0 vg		Autonomic					
Group 1	Day(s) Relative to Start Date	Exophthalmus	Lacrimation	Erected Fur	Pupil Response	Salivation	Defecation
1509	-1	0	0	0	0	0	-1
	27	0	0	0	0	0	-1
	89	0	0	0	0	0	-1
1510	-1	0	0	0	0	0	-1
	27	0	0	0	0	0	-1
	89	0	0	0	0	0	-1

Day 27 represents data collected from Day 27 to 32.

Appendix 10

Individual Neurobehavioral Evaluation: Autonomic

32025165

Sex: Female

0 vg		Autonomic	
Group 1	Day(s) Relative to Start Date	Palpebral Closure (HC)	Palpebral Closure (OF)
1509	-1	0	0
	27	0	0
	89	0	0
1510	-1	0	0
	27	0	0
	89	0	0

Day 27 represents data collected from Day 27 to 32.

Appendix 10

Individual Neurobehavioral Evaluation: Autonomic

32025165
Sex: Female

1.49x10^11 vg Group 2	Day(s) Relative to Start Date	Autonomic					
		Exophthalmus	Lacrimation	Erected Fur	Pupil Response	Salivation	Defecation
2501	-1	0	0	0	0	0	-1
	27	0	0	0	0	0	-1
	89	0	0	0	0	0	-1
2502	-1	0	0	0	0	0	0
	27	0	0	0	0	0	0
	89	0	0	0	0	0	0
2503	-1	0	0	0	0	0	-1
	27	0	0	0	0	0	-1
	89	0	0	0	0	0	-1
2504	-1	0	0	0	0	0	-1
	27	0	0	0	0	0	0
	89	0	0	0	0	0	-1
2505	-1	0	0	0	0	0	-1
	27	0	0	0	0	0	0
	89	0	0	0	0	0	-1
2506	-1	0	0	0	0	0	-1
	27	0	0	0	0	0	-1
	89	0	0	0	0	0	-1
2507	-1	0	0	0	0	0	-1
	27	0	0	0	0	0	-1
	89	0	0	0	0	0	-1

Day 27 represents data collected from Day 27 to 32.

Appendix 10

Individual Neurobehavioral Evaluation: Autonomic

32025165

Sex: Female

1.49x10^11 vg<br>Group 2	Day(s) Relative to Start Date	Autonomic<br>Palpebral Closure (HC)	 Palpebral Closure (OF)
2501	-1	0	0
	27	0	0
	89	0	0
2502	-1	0	0
	27	0	0
	89	0	0
2503	-1	0	0
	27	0	0
	89	0	0
2504	-1	0	0
	27	0	0
	89	0	0
2505	-1	0	0
	27	0	0
	89	0	0
2506	-1	0	0
	27	0	0
	89	0	0
2507	-1	0	0
	27	0	0
	89	0	0

Day 27 represents data collected from Day 27 to 32.

Appendix 10

Individual Neurobehavioral Evaluation: Autonomic

32025165
Sex: Female

1.49x10^11 vg<br>Group 2	Day(s) Relative to Start Date	Autonomic					
		Exophthalmus	Lacrimation	Erected Fur	Pupil Response	Salivation	Defecation
2508	-1	0	0	0	0	0	-1
	27	0	0	0	0	0	0
	89	0	0	0	0	0	-1
2509	-1	0	0	0	0	0	-1
	27	0	0	0	0	0	-1
	89	0	0	0	0	0	-1
2510	-1	0	0	0	0	0	-1
	27	0	0	0	0	0	0
	89	0	0	0	0	0	-1

Day 27 represents data collected from Day 27 to 32.

Appendix 10

Individual Neurobehavioral Evaluation: Autonomic

32025165

Sex: Female

1.49x10^11 vg Group 2	Day(s) Relative to Start Date	Autonomic	
		Palpebral Closure (HC)	Palpebral Closure (OF)
2508	-1	0	0
	27	0	0
	89	0	0
2509	-1	0	0
	27	0	0
	89	0	0
2510	-1	0	0
	27	0	0
	89	0	0

Day 27 represents data collected from Day 27 to 32.

Appendix 10

Individual Neurobehavioral Evaluation: Autonomic

32025165
Sex: Female

4.47x10^11 vg Group 3	Day(s) Relative to Start Date	Autonomic					
		Exophthalmus	Lacrimation	Erected Fur	Pupil Response	Salivation	Defecation
3501	-1	0	0	0	0	0	-1
	27	0	0	0	0	0	-1
	89	0	0	0	0	0	-1
3502	-1	0	0	0	0	0	-1
	27	0	0	0	0	0	0
	89	0	0	0	0	0	-1
3503	-1	0	0	0	0	0	-1
	27	0	0	0	0	0	-1
	89	0	0	0	0	0	-1
3504	-1	0	0	0	0	0	-1
	27	0	0	0	0	0	0
	89	0	0	0	0	0	-1
3505	-1	0	0	0	0	0	-1
	27	0	0	0	0	0	-1
	89	0	0	0	0	0	-1
3506	-1	0	0	0	0	0	-1
	27	0	0	0	0	0	0
	89	0	0	0	0	0	-1
3507	-1	0	0	0	0	0	-1
	27	0	0	0	0	0	-1
	89	0	0	0	0	0	-1

Day 27 represents data collected from Day 27 to 32.

Appendix 10

Individual Neurobehavioral Evaluation: Autonomic

32025165

Sex: Female

4.47x10^11 vg<br>Group 3	Day(s) Relative to Start Date	Autonomic	
		Palpebral Closure (HC)	Palpebral Closure (OF)
3501	-1	0	0
	27	0	0
	89	0	0
3502	-1	0	0
	27	0	0
	89	0	0
3503	-1	0	0
	27	0	0
	89	0	0
3504	-1	0	0
	27	0	0
	89	0	0
3505	-1	0	0
	27	0	0
	89	0	0
3506	-1	0	0
	27	0	0
	89	0	0
3507	-1	0	0
	27	0	0
	89	0	0

Day 27 represents data collected from Day 27 to 32.

Appendix 10

Individual Neurobehavioral Evaluation: Autonomic

32025165
Sex: Female

4.47x10^11 vg		Autonomic					
Group 3	Day(s) Relative to Start Date	Exophthalmus	Lacrimation	Erected Fur	Pupil Response	Salivation	Defecation
3508	-1	0	0	0	0	0	-1
	27	0	0	0	0	0	0
	89	0	0	0	0	0	-1
3509	-1	0	0	0	0	0	-1
	27	0	0	0	0	0	0
	89	0	0	0	0	0	-1
3510	-1	0	0	0	0	0	-1
	27	0	0	0	0	0	0
	89	0	0	0	0	0	-1

Day 27 represents data collected from Day 27 to 32.

Appendix 10

Individual Neurobehavioral Evaluation: Autonomic

32025165

Sex: Female

4.47x10^11 vg<br>Group 3	Day(s) Relative to Start Date	Autonomic	
		Palpebral Closure (HC)	Palpebral Closure (OF)
3508	-1	0	0
	27	0	0
	89	0	0
3509	-1	0	0
	27	0	0
	89	0	0
3510	-1	0	0
	27	0	0
	89	0	0

Day 27 represents data collected from Day 27 to 32.

Appendix 10

Individual Neurobehavioral Evaluation: Autonomic

32025165
Sex: Female

1.49x10^12 vg Group 4	Day(s) Relative to Start Date	Autonomic					
		Exophthalmus	Lacrimation	Erected Fur	Pupil Response	Salivation	Defecation
4501	-1	0	0	0	0	0	-1
	27	0	0	0	0	0	-1
4502	-1	0	0	0	0	0	-1
	27	0	0	0	0	0	-1
4503	-1	0	0	0	0	0	-1
	27	0	0	0	2 [a]	0	-1
4504	-1	0	0	0	0	0	-1
	27	0	0	0	0	0	-1
4505	-1	0	0	0	0	0	-1
	27	0	0	0	0	0	-1
4506	-1	0	0	0	0	0	-1
	27	0	0	0	0	0	-1
	89	0	0	0	0	0	-1
4507	-1	0	0	0	0	0	-1
	27	0	0	0	0	0	0
	89	0	0	0	0	0	-1
4508	-1	0	0	0	0	0	-1
	27	0	0	0	0	0	-1
	89	0	0	0	0	0	-1

[a] [RC:Left pupil fully constricted prior to performing the test Lack of constriction of the right pupil]
Day 27 represents data collected from Day 27 to 32.

Appendix 10

Individual Neurobehavioral Evaluation: Autonomic

32025165

Sex: Female

1.49x10^12 vg<br>Group 4	Day(s) Relative to Start Date	Autonomic	
		Palpebral Closure (HC)	Palpebral Closure (OF)
4501	-1	0	0
	27	0	0
4502	-1	0	0
	27	0	0
4503	-1	0	0
	27	0	0
4504	-1	0	0
	27	0	0
4505	-1	0	0
	27	0	0
4506	-1	0	0
	27	0	0
	89	0	0
4507	-1	0	0
	27	0	0
	89	0	0
4508	-1	0	0
	27	0	0
	89	0	0

Day 27 represents data collected from Day 27 to 32.

Appendix 10

Individual Neurobehavioral Evaluation: Autonomic

32025165

Sex: Female

1.49x10^12 vg Group 4	Day(s) Relative to Start Date	Autonomic					
		Exophthalmus	Lacrimation	Erected Fur	Pupil Response	Salivation	Defecation
4509	-1	0	0	0	0	0	-1
	27	0	0	0	0	0	-1
	89	0	0	0	0	0	-1
4510	-1	0	0	0	0	0	-1
	27	0	0	0	0	0	0
	89	0	0	0	0	0	-1
4511	-1	0	0	0	0	0	-1
	27	0	0	0	0	0	0
	89	0	0	0	0	0	-1
4512	-1	0	0	0	0	0	-1
	27	0	0	0	0	0	-1
	89	0	0	0	0	0	-1
4513	-1	0	0	0	0	0	-1
	27	0	0	0	0	0	-1
	89	0	0	0	0	0	-1
4514	-1	0	0	0	0	0	-1
	27	0	0	0	0	0	0
	89	0	0	0	0	0	-1
4515	-1	0	0	0	0	0	-1
	27	0	0	0	0	0	-1
	89	0	0	0	0	0	-1

Day 27 represents data collected from Day 27 to 32.

Appendix 10

Individual Neurobehavioral Evaluation: Autonomic

32025165

Sex: Female

1.49x10^12 vg<br>Group 4	Day(s) Relative to Start Date	Autonomic	
		Palpebral Closure (HC)	Palpebral Closure (OF)
4509	-1	0	0
	27	0	0
	89	0	0
4510	-1	0	0
	27	0	0
	89	0	0
4511	-1	0	0
	27	0	0
	89	0	0
4512	-1	0	0
	27	0	0
	89	0	0
4513	-1	0	0
	27	0	0
	89	0	0
4514	-1	0	0
	27	0	0
	89	0	0
4515	-1	0	0
	27	0	0
	89	0	0

Day 27 represents data collected from Day 27 to 32.

Appendix 11

Individual Neurobehavioral Evaluation: Excitability

32025165
Sex: Male

0 vg		Excitability				
Group 1	Day(s) Relative to Start Date	Vocal-izations	Startle Response	Ease of Removal	Handling Reactivity	Convulsions (HC)
1001	-1	0	0	0	0	0
	27	0	0	0	0	0
1002	-1	0	0	0	0	0
	27	0	0	0	0	0
1003	-1	0	0	0	0	0
	27	0	0	0	0	0
1004	-1	0	0	0	0	0
	27	0	0	0	0	0
1005	-1	0	0	0	0	0
	27	0	0	0	0	0
1006	-1	0	0	0	0	0
	27	0	0	0	0	0
	88	0	0	0	0	0
1007	-1	0	0	0	0	0
	27	0	0	0	0	0
	88	0	0	0	0	0
1008	-1	0	0	0	0	0
	27	0	0	0	0	0
	88	0	0	0	0	0

Day 27 represents data collected from Day 27 to 32.

Appendix 11

Individual Neurobehavioral Evaluation: Excitability

32025165
Sex: Male

0 vg		Excitability				
Group 1	Day(s) Relative to Start Date	Vocal-izations	Startle Response	Ease of Removal	Handling Reactivity	Convulsions (HC)
1009	-1	0	0	0	0	0
	27	0	0	0	0	0
	88	0	0	0	0	0
1010	-1	0	0	0	0	0
	27	0	0	0	0	0
	88	0	0	0	0	0

Day 27 represents data collected from Day 27 to 32.

Appendix 11

Individual Neurobehavioral Evaluation: Excitability

32025165
Sex: Male

1.49x10^11 vg Group 2	Day(s) Relative to Start Date	Excitability: Vocal-izations	Excitability: Startle Response	Excitability: Ease of Removal	Excitability: Handling Reactivity	Excitability: Convulsions (HC)
2001	-1	0	0	0	0	0
	27	0	0	0	0	0
	88	0	0	0	0	0
2002	-1	0	0	0	0	0
	27	0	0	0	0	0
	88	0	0	0	0	0
2003	-1	0	0	0	0	0
	27	0	0	0	0	0
	88	0	0	0	0	0
2004	-1	0	0	1	0	0
	27	0	0	0	0	0
	88	0	0	0	0	0
2005	-1	0	0	0	0	0
	27	0	0	0	0	0
	88	0	0	0	0	0
2006	-1	0	0	0	0	0
	27	0	0	0	0	0
	88	0	0	0	0	0
2007	-1	0	0	0	0	0
	27	0	0	0	0	0
	88	0	0	0	0	0

Day 27 represents data collected from Day 27 to 32.

Appendix 11

Individual Neurobehavioral Evaluation: Excitability

32025165
Sex: Male

1.49x10^11 vg Group 2	Day(s) Relative to Start Date	Excitability				
		Vocal-izations	Startle Response	Ease of Removal	Handling Reactivity	Convulsions (HC)
2008	-1	0	0	0	0	0
	27	0	0	0	0	0
	88	0	0	0	0	0
2009	-1	0	0	0	0	0
	27	0	0	0	0	0
	88	0	0	0	0	0
2010	-1	0	0	0	0	0
	27	0	0	0	0	0
	88	0	0	0	0	0

Day 27 represents data collected from Day 27 to 32.

Appendix 11

Individual Neurobehavioral Evaluation: Excitability

32025165
Sex: Male

4.47x10^11 vg Group 3	Day(s) Relative to Start Date	Excitability				
		Vocal-izations	Startle Response	Ease of Removal	Handling Reactivity	Convulsions (HC)
3001	-1	0	0	0	0	0
	27	0	0	0	0	0
	88	0	0	0	0	0
3002	-1	0	0	0	0	0
	27	0	0	0	0	0
	88	0	0	0	0	0
3003	-1	0	0	0	0	0
	27	0	0	0	0	0
	88	0	0	0	0	0
3004	-1	0	0	0	1	0
	27	0	0	0	0	0
	88	0	0	0	0	0
3005	-1	0	0	0	0	0
	27	0	0	0	0	0
	88	0	0	0	0	0
3006	-1	0	0	0	0	0
	27	0	0	0	0	0
	88	0	0	0	0	0
3007	-1	0	0	1	0	0
	27	0	0	0	0	0
	88	0	0	0	0	0

Day 27 represents data collected from Day 27 to 32.

Appendix 11

Individual Neurobehavioral Evaluation: Excitability

32025165
Sex: Male

4.47x10^11 vg Group 3	Day(s) Relative to Start Date	Excitability				
		Vocal-izations	Startle Response	Ease of Removal	Handling Reactivity	Convulsions (HC)
3008	-1	0	0	0	0	0
	27	0	0	0	0	0
	88	0	0	0	0	0
3009	-1	0	0	0	0	0
	27	0	0	0	0	0
	88	0	0	-1	-1	0
3010	-1	0	0	0	0	0
	27	0	0	0	0	0
	88	0	0	0	0	0

Day 27 represents data collected from Day 27 to 32.

Appendix 11

Individual Neurobehavioral Evaluation: Excitability

32025165
Sex: Male

1.49x10^12 vg<br>Group 4	Day(s) Relative to Start Date	Excitability				
		Vocal-izations	Startle Response	Ease of Removal	Handling Reactivity	Convulsions (HC)
4001	-1	0	0	0	0	0
	27	0	0	0	0	0
4002	-1	0	0	0	0	0
	27	0	0	0	0	0
4003	-1	0	0	0	0	0
	27	0	0	0	0	0
4004	-1	0	0	0	0	0
	27	0	0	0	0	0
4005	-1	0	0	0	0	0
	27	0	0	0	0	0
4006	-1	0	0	0	0	0
	27	0	-1	0	0	0
	88	0	0	0	0	0
4007	-1	0	0	0	0	0
	27	0	0	0	0	0
	88	0	0	0	0	0
4008	-1	0	0	0	0	0
	27	0	0	0	0	0
	88	0	0	0	0	0

Day 27 represents data collected from Day 27 to 32.

Appendix 11

Individual Neurobehavioral Evaluation: Excitability

32025165
Sex: Male

1.49x10^12 vg Group 4	Day(s) Relative to Start Date	Excitability				
		Vocal-izations	Startle Response	Ease of Removal	Handling Reactivity	Convulsions (HC)
4009	-1	0	0	0	0	0
	27	0	0	0	0	0
	88	0	0	0	0	0
4010	-1	0	0	0	0	0
	27	0	0	0	0	0
	88	0	0	0	0	0
4011	-1	0	0	0	0	0
	27	0	0	0	0	0
	88	0	0	0	0	0
4012	-1	0	0	1	1	0
	27	0	0	0	0	0
	88	0	1	1	1	0
4013	-1	0	0	0	0	0
	27	0	0	0	1	0
	88	0	0	0	0	0
4014	-1	0	0	0	0	0
	27	0	0	0	0	0
	88	0	0	0	0	0
4015	-1	0	0	0	0	0
	27	0	0	0	0	0
	88	0	0	0	0	0

Day 27 represents data collected from Day 27 to 32.

Appendix 11

Individual Neurobehavioral Evaluation: Excitability

32025165
Sex: Female

0 vg		Excitability				
Group 1	Day(s) Relative to Start Date	Vocal-izations	Startle Response	Ease of Removal	Handling Reactivity	Convulsions (HC)
1501	-1	0	0	0	0	0
	27	0	0	0	0	0
1502	-1	0	0	0	0	0
	27	0	0	0	0	0
1503	-1	0	0	0	0	0
	27	0	0	0	0	0
1504	-1	0	0	0	0	0
	27	0	0	0	0	0
1505	-1	0	0	0	0	0
	27	0	0	0	0	0
1506	-1	0	0	0	0	0
	27	0	0	0	0	0
	89	0	0	0	0	0
1507	-1	0	0	0	0	0
	27	0	0	0	0	0
	89	0	0	0	0	0
1508	-1	0	0	0	0	0
	27	0	0	0	0	0
	89	0	0	0	0	0

Day 27 represents data collected from Day 27 to 32.

Appendix 11

Individual Neurobehavioral Evaluation: Excitability

32025165
Sex: Female

0 vg		Excitability				
Group 1	Day(s) Relative to Start Date	Vocal-izations	Startle Response	Ease of Removal	Handling Reactivity	Convulsions (HC)
1509	-1	0	0	0	0	0
	27	0	0	0	0	0
	89	0	0	0	0	0
1510	-1	0	0	0	0	0
	27	0	0	0	0	0
	89	0	0	0	0	0

Day 27 represents data collected from Day 27 to 32.

Appendix 11

Individual Neurobehavioral Evaluation: Excitability

32025165
Sex: Female

1.49x10^11 vg Group 2	Day(s) Relative to Start Date	Excitability: Vocal-izations	Excitability: Startle Response	Excitability: Ease of Removal	Excitability: Handling Reactivity	Excitability: Convulsions (HC)
2501	-1	0	0	0	0	0
	27	0	0	0	0	0
	89	0	0	0	0	0
2502	-1	0	0	0	0	0
	27	0	0	0	0	0
	89	0	0	0	1	0
2503	-1	0	0	0	0	0
	27	0	0	0	0	0
	89	0	-1	0	0	0
2504	-1	0	0	0	0	0
	27	0	0	0	0	0
	89	0	0	0	0	0
2505	-1	0	0	0	0	0
	27	0	0	0	0	0
	89	0	0	0	0	0
2506	-1	0	0	0	0	0
	27	0	0	0	0	0
	89	0	0	0	0	0
2507	-1	0	0	0	0	0
	27	0	0	0	0	0
	89	0	0	0	0	0

Day 27 represents data collected from Day 27 to 32.

Appendix 11

Individual Neurobehavioral Evaluation: Excitability

32025165
Sex: Female

1.49x10^11 vg Group 2	Day(s) Relative to Start Date	Excitability				
		Vocal-izations	Startle Response	Ease of Removal	Handling Reactivity	Convulsions (HC)
2508	-1	0	0	0	0	0
	27	0	0	0	0	0
	89	0	0	0	0	0
2509	-1	0	0	0	0	0
	27	0	0	0	0	0
	89	0	0	0	0	0
2510	-1	0	0	0	0	0
	27	0	0	0	0	0
	89	0	0	0	0	0

Day 27 represents data collected from Day 27 to 32.

Appendix 11

Individual Neurobehavioral Evaluation: Excitability

32025165
Sex: Female

4.47x10^11 vg Group 3	Day(s) Relative to Start Date	Excitability				
		Vocal-izations	Startle Response	Ease of Removal	Handling Reactivity	Convulsions (HC)
3501	-1	0	0	0	0	0
	27	0	0	0	0	0
	89	0	0	0	0	0
3502	-1	0	0	0	0	0
	27	0	0	0	0	0
	89	0	0	0	0	0
3503	-1	0	0	0	0	0
	27	0	0	0	0	0
	89	0	0	0	0	0
3504	-1	0	0	1	1	0
	27	0	0	0	0	0
	89	0	0	0	0	0
3505	-1	0	0	0	0	0
	27	0	0	0	0	0
	89	0	0	0	0	0
3506	-1	0	0	0	0	0
	27	0	0	0	0	0
	89	0	0	0	0	0
3507	-1	0	0	0	0	0
	27	0	0	0	0	0
	89	0	0	0	0	0

Day 27 represents data collected from Day 27 to 32.

Appendix 11

Individual Neurobehavioral Evaluation: Excitability

32025165
Sex: Female

4.47x10^11 vg Group 3	Day(s) Relative to Start Date	Excitability				
		Vocal-izations	Startle Response	Ease of Removal	Handling Reactivity	Convulsions (HC)
3508	-1	0	0	0	0	0
	27	0	0	0	0	0
	89	0	0	0	0	0
3509	-1	0	0	1	0	0
	27	0	0	0	0	0
	89	0	0	0	0	0
3510	-1	0	0	0	0	0
	27	0	0	0	0	0
	89	0	0	0	0	0

Day 27 represents data collected from Day 27 to 32.

Appendix 11

Individual Neurobehavioral Evaluation: Excitability

32025165
Sex: Female

1.49x10^12 vg Group 4	Day(s) Relative to Start Date	Excitability: Vocal-izations	Excitability: Startle Response	Excitability: Ease of Removal	Excitability: Handling Reactivity	Excitability: Convulsions (HC)
4501	-1	0	0	0	0	0
	27	0	0	0	0	0
4502	-1	0	0	0	0	0
	27	0	0	0	0	0
4503	-1	0	0	0	0	0
	27	0	0	0	0	0
4504	-1	0	0	0	0	0
	27	0	0	0	0	0
4505	-1	0	0	0	0	0
	27	0	0	0	0	0
4506	-1	0	0	0	0	0
	27	0	0	0	0	0
	89	0	0	0	0	0
4507	-1	0	0	0	0	0
	27	0	0	0	0	0
	89	0	0	0	0	0
4508	-1	0	0	0	0	0
	27	0	0	0	0	0
	89	0	0	0	0	0

Day 27 represents data collected from Day 27 to 32.

Appendix 11

Individual Neurobehavioral Evaluation: Excitability

32025165
Sex: Female

1.49x10^12 vg Group 4	Day(s) Relative to Start Date	Excitability				
		Vocal-izations	Startle Response	Ease of Removal	Handling Reactivity	Convulsions (HC)
4509	-1	0	0	0	0	0
	27	0	0	0	0	0
	89	0	0	0	0	0
4510	-1	0	0	0	0	0
	27	0	0	0	0	0
	89	0	0	0	1	0
4511	-1	0	0	0	0	0
	27	0	0	0	0	0
	89	0	0	0	0	0
4512	-1	0	0	0	0	0
	27	0	0	0	0	0
	89	0	0	0	0	0
4513	-1	0	0	0	0	0
	27	0	0	0	0	0
	89	0	0	0	0	0
4514	-1	0	0	0	0	0
	27	0	0	0	0	0
	89	0	0	0	0	0
4515	-1	0	0	0	0	0
	27	0	0	0	0	0
	89	0	0	0	0	0

Day 27 represents data collected from Day 27 to 32.

Appendix 12

Individual Neurobehavioral Evaluation: Neuromuscular

32025165
Sex: Male

0 vg		Neuromuscular					
Group 1	Day(s) Relative to Start Date	Gait/ Mobility	Forelimb Grp Strength kg1 (Kg)	Forelimb Grp Strength kg2 (Kg)	Forelimb Grp Strength kg3 (Kg)	Hindlimb Grp Strength kg1 (Kg)	Hindlimb Grp Strength kg2 (Kg)
1001	-1	0	0.570	0.520	0.690	0.330	0.490
	27	0	0.140	0.190	0.280	0.200	0.240
1002	-1	0	0.760	0.290	0.810	0.320	0.270
	27	0	0.610	0.760	0.540	0.200	0.420
1003	-1	0	0.460	0.540	0.760	0.310	0.330
	27	0	0.640	0.390	0.360	0.460	0.560
1004	-1	0	0.500	0.420	0.590	0.500	0.390
	27	0	0.310	0.270	0.580	0.160	0.100
1005	-1	0	0.990	0.400	0.620	0.600	0.570
	27	0	0.540	0.460	0.530	0.440	0.200
1006	-1	0	0.080	0.520	0.260	0.110	0.060
	27	0	0.620	0.610	0.690	0.200	0.340
	88	0	0.830	1.030	0.990	0.240	0.370
1007	-1	0	0.270	0.320	0.410	0.280	0.290
	27	0	0.370	0.860	0.430	0.170	0.230
	88	0	1.030	1.030	0.840	0.190	0.100
1008	-1	0	0.410	0.380	0.410	0.270	0.310
	27	0	0.480	0.350	0.300	0.240	0.150
	88	0	0.530	0.220	0.640	0.180	0.140

Day 27 represents data collected from Day 27 to 32.

Appendix 12

Individual Neurobehavioral Evaluation: Neuromuscular

32025165
Sex: Male

0 vg		Neuromuscular					
Group 1	Day(s) Relative to Start Date	Hindlimb Grp Strength kg3 (Kg)	Mn Hindlimb Grp Strength (Kg)	Mn Forelimb Grp Strength (Kg)	Air Right ing Reflex	Tremor (HC)	Tremor (OF)
1001	-1	0.310	0.377	0.593	0	0	0
	27	0.270	0.237	0.203	0	0	2
1002	-1	0.300	0.297	0.620	0	0	0
	27	0.320	0.313	0.637	0	0	0
1003	-1	0.310	0.317	0.587	0	0	0
	27	0.660	0.560	0.463	0	0	0
1004	-1	0.380	0.423	0.503	0	0	0
	27	0.500	0.253	0.387	0	0	0
1005	-1	0.380	0.517	0.670	0	0	0
	27	0.360	0.333	0.510	0	0	0
1006	-1	0.080	0.083	0.287	0	0	0
	27	0.230	0.257	0.640	0	0	0
	88	0.340	0.317	0.950	0	0	0
1007	-1	0.310	0.293	0.333	0	0	0
	27	0.140	0.180	0.553	0	0	0
	88	0.310	0.200	0.967	0	0	0
1008	-1	0.280	0.287	0.400	0	0	0
	27	0.140	0.177	0.377	0	0	0
	88	0.160	0.160	0.463	0	0	0

Day 27 represents data collected from Day 27 to 32.

Appendix 12

Individual Neurobehavioral Evaluation: Neuromuscular

32025165
Sex: Male

0 vg Group 1	Day(s) Relative to Start Date	Neuromuscular Body Tone	Hindlimb Splay Trial1 (cm)	Hindlimb Splay Trial2 (cm)	Mn Hindlimb Splay (cm)
1001	-1	0	10.5	11.7	11.1
	27	0	15.5	16.6	16.1
1002	-1	0	15.6	5.7	10.7
	27	0	17.9	16.2	17.1
1003	-1	0	13.4	12.8	13.1
	27	0	16.2	17.0	16.6
1004	-1	0	14.5	15.8	15.2
	27	0	18.4	17.7	18.1
1005	-1	0	14.8	13.5	14.2
	27	0	13.4	14.4	13.9
1006	-1	0	8.8	7.8	8.3
	27	0	13.7	10.8	12.3
	88	0	14.6	14.0	14.3
1007	-1	0	17.4	16.7	17.1
	27	0	17.5	15.6	16.6
	88	0	17.7	15.4	16.6
1008	-1	0	14.1	11.7	12.9
	27	0	10.1	11.7	10.9
	88	0	16.2	10.3	13.3

Day 27 represents data collected from Day 27 to 32.

Appendix 12

Individual Neurobehavioral Evaluation: Neuromuscular

32025165
Sex: Male

0 vg		Neuromuscular					
Group 1	Day(s) Relative to Start Date	Gait/ Mobility	Forelimb Grp Strength kg1 (Kg)	Forelimb Grp Strength kg2 (Kg)	Forelimb Grp Strength kg3 (Kg)	Hindlimb Grp Strength kg1 (Kg)	Hindlimb Grp Strength kg2 (Kg)
1009	-1	0	0.660	0.530	0.680	0.470	0.540
	27	0	0.660	0.360	0.480	0.170	0.140
	88	0	0.430	0.810	0.780	0.280	0.490
1010	-1	0	0.390	0.590	0.620	0.330	0.350
	27	0	0.360	0.240	0.280	0.130	0.150
	88	0	0.940	0.670	0.740	0.370	0.170

Day 27 represents data collected from Day 27 to 32.

Appendix 12

Individual Neurobehavioral Evaluation: Neuromuscular

32025165
Sex: Male

0 vg		Neuromuscular					
Group 1	Day(s) Relative to Start Date	Hindlimb Grp Strength kg3 (Kg)	Mn Hindlimb Grp Strength (Kg)	Mn Forelimb Grp Strength (Kg)	Air Right ing Reflex	Tremor (HC)	Tremor (OF)
1009	-1	0.540	0.517	0.623	0	0	0
	27	0.090	0.133	0.500	0	0	0
	88	0.520	0.430	0.673	0	0	0
1010	-1	0.160	0.280	0.533	0	0	0
	27	0.140	0.140	0.293	0	0	0
	88	0.370	0.303	0.783	0	0	0

Day 27 represents data collected from Day 27 to 32.

Appendix 12

Individual Neurobehavioral Evaluation: Neuromuscular

32025165
Sex: Male

0 vg Group 1	Day(s) Relative to Start Date	Neuromuscular Body Tone	Hindlimb Splay Trial1 (cm)	Hindlimb Splay Trial2 (cm)	Mn Hindlimb Splay (cm)
1009	-1	0	17.1	15.9	16.5
	27	0	16.0	10.7	13.4
	88	0	12.1	13.7	12.9
1010	-1	0	16.4	17.2	16.8
	27	0	10.4	9.9	10.2
	88	0	17.2	13.6	15.4

Day 27 represents data collected from Day 27 to 32.

Appendix 12

Individual Neurobehavioral Evaluation: Neuromuscular

32025165
Sex: Male

1.49x10^11 vg<br>Group 2	Day(s) Relative to Start Date	Neuromuscular					
		Gait/ Mobility	Forelimb Grp Strength kg1 (Kg)	Forelimb Grp Strength kg2 (Kg)	Forelimb Grp Strength kg3 (Kg)	Hindlimb Grp Strength kg1 (Kg)	Hindlimb Grp Strength kg2 (Kg)
2001	-1	0	0.280	0.260	0.220	0.140	0.170
	27	0	0.510	0.650	0.680	0.030	0.220
	88	0	0.770	0.750	0.670	0.240	0.190
2002	-1	0	0.380	0.340	0.140	0.050	0.090
	27	0	0.200	0.210	0.220	0.240	0.110
	88	0	0.780	0.710	0.440	0.220	0.250
2003	-1	0	0.350	0.440	0.540	0.310	0.320
	27	0	0.480	0.520	0.620	0.290	0.240
	88	0	0.470	0.940	0.650	0.310	0.390
2004	-1	0	0.340	0.620	0.720	0.400	0.700
	27	0	0.490	0.420	0.350	0.260	0.280
	88	0	0.680	0.710	0.710	0.240	0.170
2005	-1	0	0.180	0.240	0.120	0.160	0.170
	27	0	0.390	0.500	0.350	0.140	0.170
	88	0	0.370	0.750	0.540	0.170	0.390
2006	-1	0	0.040	0.120	0.110	0.020	0.060
	27	0	0.740	0.810	0.740	0.170	0.060
	88	0	0.620	0.820	0.840	0.140	0.410
2007	-1	0	0.200	0.440	0.510	0.310	0.370
	27	0	0.690	0.530	0.550	0.240	0.170
	88	0	0.830	0.570	0.680	0.380	0.170

Day 27 represents data collected from Day 27 to 32.

Appendix 12

Individual Neurobehavioral Evaluation: Neuromuscular

32025165
Sex: Male

1.49x10^11 vg Group 2	Day(s) Relative to Start Date	Neuromuscular					
		Hindlimb Grp Strength kg3 (Kg)	Mn Hindlimb Grp Strength (Kg)	Mn Forelimb Grp Strength (Kg)	Air Right ing Reflex	Tremor (HC)	Tremor (OF)
2001	-1	0.120	0.143	0.253	0	0	0
	27	0.150	0.133	0.613	0	0	0
	88	0.360	0.263	0.730	0	0	0
2002	-1	0.070	0.070	0.287	0	0	0
	27	0.210	0.187	0.210	0	0	0
	88	0.290	0.253	0.643	0	0	0
2003	-1	0.330	0.320	0.443	0	0	0
	27	0.210	0.247	0.540	0	0	0
	88	0.170	0.290	0.687	0	0	0
2004	-1	0.450	0.517	0.560	0	0	0
	27	0.250	0.263	0.420	0	0	0
	88	0.180	0.197	0.700	0	0	0
2005	-1	0.190	0.173	0.180	0	0	0
	27	0.150	0.153	0.413	0	0	0
	88	0.170	0.243	0.553	0	0	0
2006	-1	0.030	0.037	0.090	0	0	0
	27	0.350	0.193	0.763	0	0	0
	88	0.270	0.273	0.760	0	0	0
2007	-1	0.390	0.357	0.383	0	0	0
	27	0.240	0.217	0.590	0	0	0
	88	0.310	0.287	0.693	0	0	0

Day 27 represents data collected from Day 27 to 32.

Appendix 12

Individual Neurobehavioral Evaluation: Neuromuscular

32025165
Sex: Male

1.49x10^11 vg Group 2	Day(s) Relative to Start Date	Neuromuscular Body Tone	Neuromuscular Hindlimb Splay Trial1 (cm)	Neuromuscular Hindlimb Splay Trial2 (cm)	Neuromuscular Mn Hindlimb Splay (cm)
2001	-1	0	12.3	9.3	10.8
	27	0	13.1	12.8	13.0
	88	0	15.2	13.0	14.1
2002	-1	0	-	-	-
	27	0	8.3	9.0	8.7
	88	0	13.9	13.8	13.9
2003	-1	0	18.2	13.4	15.8
	27	0	16.8	14.6	15.7
	88	0	16.4	11.4	13.9
2004	-1	0	15.2	10.5	12.9
	27	0	12.7	11.8	12.3
	88	0	13.8	9.4	11.6
2005	-1	0	13.7	11.0	12.4
	27	0	12.7	9.4	11.1
	88	0	13.5	10.7	12.1
2006	-1	0	5.4	6.2	5.8
	27	0	14.5	13.5	14.0
	88	0	10.8	10.6	10.7
2007	-1	0	16.0	12.1	14.1
	27	0	13.5	12.7	13.1
	88	0	16.6	12.2	14.4

Day 27 represents data collected from Day 27 to 32.

Appendix 12

Individual Neurobehavioral Evaluation: Neuromuscular

32025165
Sex: Male

1.49x10^11 vg Group 2	Day(s) Relative to Start Date	Neuromuscular					
		Gait/ Mobility	Forelimb Grp Strength kg1 (Kg)	Forelimb Grp Strength kg2 (Kg)	Forelimb Grp Strength kg3 (Kg)	Hindlimb Grp Strength kg1 (Kg)	Hindlimb Grp Strength kg2 (Kg)
2008	-1	0	0.200	0.200	0.130	0.270	0.030
	27	0	0.670	0.620	0.720	0.120	0.080
	88	0	0.770	0.650	0.650	0.160	0.220
2009	-1	0	0.450	0.580	0.660	0.440	0.410
	27	0	0.110	0.150	0.280	0.160	0.200
	88	0	0.650	0.630	0.640	0.300	0.240
2010	-1	0	0.230	0.320	0.240	0.240	0.240
	27	0	0.090	0.100	0.090	0.120	0.180
	88	0	0.950	0.860	0.830	0.180	0.220

Day 27 represents data collected from Day 27 to 32.

Appendix 12

Individual Neurobehavioral Evaluation: Neuromuscular

32025165
Sex: Male

1.49x10^11 vg Group 2	Day(s) Relative to Start Date	Neuromuscular					
		Hindlimb Grp Strength kg3 (Kg)	Mn Hindlimb Grp Strength (Kg)	Mn Forelimb Grp Strength (Kg)	Air Right ing Reflex	Tremor (HC)	Tremor (OF)
2008	-1	0.220	0.173	0.177	0	0	0
	27	0.160	0.120	0.670	0	0	0
	88	0.210	0.197	0.690	0	0	0
2009	-1	0.520	0.457	0.563	0	0	0
	27	0.130	0.163	0.180	0	0	0
	88	0.300	0.280	0.640	0	0	0
2010	-1	0.350	0.277	0.263	0	0	0
	27	0.110	0.137	0.093	0	0	0
	88	0.200	0.200	0.880	0	0	0

Day 27 represents data collected from Day 27 to 32.

Appendix 12

Individual Neurobehavioral Evaluation: Neuromuscular

32025165
Sex: Male

1.49x10^11 vg Group 2	Day(s) Relative to Start Date	Neuromuscular			
		Body Tone	Hindlimb Splay Trial1 (cm)	Hindlimb Splay Trial2 (cm)	Mn Hindlimb Splay (cm)
2008	-1	0	17.6	17.6	17.6
	27	0	14.4	16.3	15.4
	88	0	15.1	11.5	13.3
2009	-1	0	10.8	7.5	9.2
	27	1	10.1	10.3	10.2
	88	0	10.7	11.3	11.0
2010	-1	0	12.1	6.3	9.2
	27	0	8.1	9.0	8.6
	88	0	16.9	13.5	15.2

Day 27 represents data collected from Day 27 to 32.

Appendix 12

Individual Neurobehavioral Evaluation: Neuromuscular

32025165
Sex: Male

4.47x10^11 vg Group 3	Day(s) Relative to Start Date	Neuromuscular					
		Gait/ Mobility	Forelimb Grp Strength kg1 (Kg)	Forelimb Grp Strength kg2 (Kg)	Forelimb Grp Strength kg3 (Kg)	Hindlimb Grp Strength kg1 (Kg)	Hindlimb Grp Strength kg2 (Kg)
3001	-1	0	0.230	0.310	0.520	0.180	0.160
	27	0	0.500	0.470	0.590	0.060	0.350
	88	0	0.640	0.760	0.820	0.230	0.290
3002	-1	0	0.380	0.260	0.390	0.510	0.220
	27	0	0.220	0.180	0.090	0.260	0.200
	88	0	0.670	0.750	0.600	0.250	0.240
3003	-1	0	0.480	0.520	0.510	0.330	0.410
	27	0	0.510	0.270	0.480	0.160	0.090
	88	0	0.250	0.280	0.190	0.210	0.300
3004	-1	0	0.070	0.060	0.050	0.030	0.060
	27	0	0.220	0.060	0.340	0.120	0.120
	88	0	0.380	0.490	0.380	0.220	0.220
3005	-1	0	0.670	0.700	0.740	0.190	0.300
	27	0	0.800	0.640	0.620	0.260	0.310
	88	0	0.630	0.970	0.600	0.410	0.500
3006	-1	0	0.260	0.140	0.210	0.160	0.100
	27	0	0.790	0.660	0.700	0.150	0.280
	88	0	1.170	0.710	0.940	0.410	0.730
3007	-1	0	0.270	0.550	0.340	0.630	0.670
	27	0	0.810	0.780	0.550	0.200	0.180
	88	0	0.280	0.500	0.330	0.550	0.090

Day 27 represents data collected from Day 27 to 32.

Appendix 12

Individual Neurobehavioral Evaluation: Neuromuscular

32025165
Sex: Male

4.47x10^11 vg Group 3	Day(s) Relative to Start Date	Neuromuscular					
		Hindlimb Grp Strength kg3 (Kg)	Mn Hindlimb Grp Strength (Kg)	Mn Forelimb Grp Strength (Kg)	Air Right ing Reflex	Tremor (HC)	Tremor (OF)
3001	-1	0.590	0.310	0.353	0	0	0
	27	0.190	0.200	0.520	0	0	0
	88	0.300	0.273	0.740	0	0	0
3002	-1	0.440	0.390	0.343	0	0	0
	27	0.070	0.177	0.163	0	0	0
	88	0.190	0.227	0.673	0	0	0
3003	-1	0.480	0.407	0.503	0	0	0
	27	0.160	0.137	0.420	0	0	0
	88	0.480	0.330	0.240	0	0	0
3004	-1	0.030	0.040	0.060	0	0	0
	27	0.150	0.130	0.207	0	0	0
	88	0.150	0.197	0.417	0	0	0
3005	-1	0.190	0.227	0.703	0	0	0
	27	0.280	0.283	0.687	0	0	0
	88	0.340	0.417	0.733	0	0	0
3006	-1	0.070	0.110	0.203	0	0	0
	27	0.210	0.213	0.717	0	0	0
	88	0.460	0.533	0.940	0	0	0
3007	-1	0.460	0.587	0.387	0	0	0
	27	0.300	0.227	0.713	0	0	0
	88	0.440	0.360	0.370	0	0	0

Day 27 represents data collected from Day 27 to 32.

Appendix 12

Individual Neurobehavioral Evaluation: Neuromuscular

32025165
Sex: Male

4.47x10^11 vg Group 3	Day(s) Relative to Start Date	Neuromuscular Body Tone	Hindlimb Splay Trial1 (cm)	Hindlimb Splay Trial2 (cm)	Mn Hindlimb Splay (cm)
3001	-1	0	9.3	8.4	8.9
	27	0	11.5	8.9	10.2
	88	0	11.9	9.6	10.8
3002	-1	0	13.7	12.4	13.1
	27	1	12.2	11.9	12.1
	88	0	12.2	12.5	12.4
3003	-1	0	15.6	13.5	14.6
	27	0	13.9	10.1	12.0
	88	0	16.9	14.2	15.6
3004	-1	0	5.1	5.6	5.4
	27	0	7.7	8.4	8.1
	88	0	16.8	14.2	15.5
3005	-1	0	11.3	9.0	10.2
	27	0	15.0	9.4	12.2
	88	0	13.5	14.2	13.9
3006	-1	0	11.6	10.8	11.2
	27	0	13.2	13.1	13.2
	88	0	15.0	15.7	15.4
3007	-1	0	13.7	11.7	12.7
	27	0	13.2	11.3	12.3
	88	0	14.3	14.5	14.4

Day 27 represents data collected from Day 27 to 32.

Appendix 12

Individual Neurobehavioral Evaluation: Neuromuscular

32025165
Sex: Male

4.47x10^11 vg Group 3	Day(s) Relative to Start Date	Neuromuscular					
		Gait/ Mobility	Forelimb Grp Strength kg1 (Kg)	Forelimb Grp Strength kg2 (Kg)	Forelimb Grp Strength kg3 (Kg)	Hindlimb Grp Strength kg1 (Kg)	Hindlimb Grp Strength kg2 (Kg)
3008	-1	0	0.650	0.730	0.470	0.140	0.110
	27	0	0.100	0.270	0.160	0.240	0.270
	88	0	0.570	0.350	0.340	0.520	0.400
3009	-1	0	0.910	0.640	0.270	0.590	0.180
	27	0	0.560	0.560	0.510	0.130	0.200
	88	0	0.470	0.710	0.350	0.690	0.240
3010	-1	0	0.560	0.880	0.700	0.510	0.150
	27	0	0.400	0.540	0.770	0.170	0.330
	88	0	0.730	0.500	0.450	0.660	0.400

Day 27 represents data collected from Day 27 to 32.

Appendix 12

Individual Neurobehavioral Evaluation: Neuromuscular

32025165
Sex: Male

4.47x10^11 vg Group 3	Day(s) Relative to Start Date	Neuromuscular Hindlimb Grp Strength kg3 (Kg)	Mn Hindlimb Grp Strength (Kg)	Mn Forelimb Grp Strength (Kg)	Air Right ing Reflex	Tremor (HC)	Tremor (OF)
3008	-1	0.630	0.293	0.617	0	0	0
	27	0.130	0.213	0.177	0	0	0
	88	0.190	0.370	0.420	0	0	0
3009	-1	0.280	0.350	0.607	0	0	0
	27	0.240	0.190	0.543	0	0	0
	88	0.270	0.400	0.510	0	0	0
3010	-1	0.740	0.467	0.713	0	0	0
	27	0.300	0.267	0.570	0	0	0
	88	0.250	0.437	0.560	0	0	0

Day 27 represents data collected from Day 27 to 32.

Appendix 12

Individual Neurobehavioral Evaluation: Neuromuscular

32025165
Sex: Male

4.47x10^11 vg<br>Group 3	Day(s) Relative to Start Date	Neuromuscular			
		Body Tone	Hindlimb Splay Trial1 (cm)	Hindlimb Splay Trial2 (cm)	Mn Hindlimb Splay (cm)
3008	-1	0	10.0	10.9	10.5
	27	0	7.1	9.0	8.1
	88	0	14.0	9.2	11.6
3009	-1	0	15.0	12.8	13.9
	27	0	15.0	11.4	13.2
	88	0	18.2	13.2	15.7
3010	-1	0	17.4	15.7	16.6
	27	0	14.9	14.1	14.5
	88	0	18.7	12.5	15.6

Day 27 represents data collected from Day 27 to 32.

Appendix 12

Individual Neurobehavioral Evaluation: Neuromuscular

32025165
Sex: Male

1.49x10^12 vg Group 4	Day(s) Relative to Start Date	Neuromuscular					
		Gait/ Mobility	Forelimb Grp Strength kg1 (Kg)	Forelimb Grp Strength kg2 (Kg)	Forelimb Grp Strength kg3 (Kg)	Hindlimb Grp Strength kg1 (Kg)	Hindlimb Grp Strength kg2 (Kg)
4001	-1	0	0.400	0.350	0.230	0.240	0.410
	27	0	0.690	0.770	0.620	0.260	0.290
4002	-1	0	0.260	0.220	0.170	0.500	0.360
	27	0	0.370	0.520	0.430	0.360	0.140
4003	-1	0	0.290	0.260	0.120	0.390	0.390
	27	0	0.500	0.610	0.680	0.220	0.680
4004	-1	0	0.220	0.300	0.180	0.130	0.210
	27	0	0.400	0.490	0.530	0.060	0.260
4005	-1	0	0.330	0.220	0.210	0.350	0.310
	27	0	0.460	0.580	0.500	0.740	0.520
4006	-1	0	0.190	0.310	0.300	0.220	0.310
	27	0	0.230	0.460	0.340	0.090	0.090
	88	0	1.160	1.010	1.170	0.460	0.800
4007	-1	0	0.340	0.380	0.370	0.090	0.100
	27	0	0.590	0.370	0.160	0.100	0.130
	88	0	0.600	0.300	0.300	0.140	0.280
4008	-1	0	0.270	0.270	0.180	0.140	0.130
	27	0	0.540	0.640	0.660	0.160	0.460
	88	0	1.060	0.300	0.360	0.280	0.210

Day 27 represents data collected from Day 27 to 32.

Appendix 12

Individual Neurobehavioral Evaluation: Neuromuscular

32025165
Sex: Male

1.49x10^12 vg<br>Group 4	Day(s) Relative to Start Date	Neuromuscular					
		Hindlimb Grp Strength kg3 (Kg)	Mn Hindlimb Grp Strength (Kg)	Mn Forelimb Grp Strength (Kg)	Air Right ing Reflex	Tremor (HC)	Tremor (OF)
4001	-1	0.300	0.317	0.327	0	0	0
	27	0.020	0.190	0.693	0	0	0
4002	-1	0.370	0.410	0.217	0	0	0
	27	0.200	0.233	0.440	0	0	0
4003	-1	0.520	0.433	0.223	0	0	0
	27	0.260	0.387	0.597	0	0	0
4004	-1	0.380	0.240	0.233	0	0	0
	27	0.340	0.220	0.473	0	0	0
4005	-1	0.250	0.303	0.253	0	0	0
	27	0.440	0.567	0.513	0	0	0
4006	-1	0.420	0.317	0.267	0	0	0
	27	0.070	0.083	0.343	0	0	0
	88	0.420	0.560	1.113	0	0	0
4007	-1	0.150	0.113	0.363	0	0	0
	27	0.090	0.107	0.373	0	0	0
	88	0.310	0.243	0.400	0	0	0
4008	-1	0.080	0.117	0.240	0	0	0
	27	0.080	0.233	0.613	0	0	0
	88	0.340	0.277	0.573	0	0	0

Day 27 represents data collected from Day 27 to 32.

Appendix 12

Individual Neurobehavioral Evaluation: Neuromuscular

32025165
Sex: Male

1.49x10^12 vg Group 4	Day(s) Relative to Start Date	Neuromuscular Body Tone	Hindlimb Splay Trial1 (cm)	Hindlimb Splay Trial2 (cm)	Mn Hindlimb Splay (cm)
4001	-1	0	13.2	12.9	13.1
	27	0	16.4	14.4	15.4
4002	-1	0	15.7	9.9	12.8
	27	0	16.4	14.4	15.4
4003	-1	0	13.2	10.4	11.8
	27	0	11.3	14.3	12.8
4004	-1	0	9.2	6.5	7.9
	27	0	9.5	8.6	9.1
4005	-1	0	12.5	11.8	12.2
	27	0	16.4	15.4	15.9
4006	-1	0	11.7	13.3	12.5
	27	-1	9.7	10.0	9.9
	88	-1	20.0	18.2	19.1
4007	-1	0	9.8	7.3	8.6
	27	0	12.6	11.9	12.3
	88	0	17.0	10.0	13.5
4008	-1	0	10.5	11.0	10.8
	27	0	15.5	14.0	14.8
	88	0	12.7	16.3	14.5

Day 27 represents data collected from Day 27 to 32.

Appendix 12

Individual Neurobehavioral Evaluation: Neuromuscular

32025165
Sex: Male

1.49x10^12 vg Group 4	Day(s) Relative to Start Date	Neuromuscular					
		Gait/ Mobility	Forelimb Grp Strength kg1 (Kg)	Forelimb Grp Strength kg2 (Kg)	Forelimb Grp Strength kg3 (Kg)	Hindlimb Grp Strength kg1 (Kg)	Hindlimb Grp Strength kg2 (Kg)
4009	-1	0	0.210	0.300	0.250	0.300	0.380
	27	0	0.080	0.130	0.080	0.050	0.150
	88	0	0.580	0.640	0.830	0.230	0.140
4010	-1	0	0.220	0.200	0.360	0.360	0.240
	27	0	0.770	0.620	0.700	0.270	0.250
	88	0	0.270	0.370	0.440	0.260	0.230
4011	-1	0	0.060	0.160	0.220	0.120	0.170
	27	0	0.220	0.220	0.270	0.130	0.240
	88	0	0.630	0.540	0.430	0.340	0.400
4012	-1	0	0.430	0.820	0.270	0.480	0.410
	27	0	0.800	0.430	0.600	0.130	0.110
	88	0	0.910	0.580	0.560	0.100	0.630
4013	-1	0	0.690	0.520	0.900	0.210	0.480
	27	0	0.100	0.070	0.270	0.040	0.110
	88	0	0.800	0.520	0.820	0.190	0.540
4014	-1	0	0.630	0.730	0.550	0.140	0.560
	27	0	0.470	0.100	0.460	0.180	0.060
	88	0	0.240	0.380	0.400	0.210	0.260
4015	-1	0	0.500	0.160	0.550	0.170	0.150
	27	0	0.510	0.530	0.200	0.220	0.150
	88	0	0.360	0.380	0.950	0.410	0.770

Day 27 represents data collected from Day 27 to 32.

Appendix 12

Individual Neurobehavioral Evaluation: Neuromuscular

32025165
Sex: Male

1.49x10^12 vg<br>Group 4	Day(s) Relative to Start Date	Neuromuscular					
		Hindlimb Grp Strength kg3 (Kg)	Mn Hindlimb Grp Strength (Kg)	Mn Forelimb Grp Strength (Kg)	Air Right ing Reflex	Tremor (HC)	Tremor (OF)
4009	-1	0.300	0.327	0.253	0	0	0
	27	0.050	0.083	0.097	0	0	0
	88	0.240	0.203	0.683	0	0	0
4010	-1	0.190	0.263	0.260	0	0	0
	27	0.340	0.287	0.697	0	0	0
	88	0.110	0.200	0.360	0	0	0
4011	-1	0.100	0.130	0.147	0	0	0
	27	0.060	0.143	0.237	0	0	0
	88	0.430	0.390	0.533	0	0	0
4012	-1	0.330	0.407	0.507	0	0	0
	27	0.090	0.110	0.610	0	0	0
	88	0.110	0.280	0.683	0	0	0
4013	-1	0.300	0.330	0.703	0	0	0
	27	0.070	0.073	0.147	0	0	0
	88	0.440	0.390	0.713	0	0	0
4014	-1	0.280	0.327	0.637	0	0	0
	27	0.130	0.123	0.343	0	0	0
	88	0.550	0.340	0.340	0	0	0
4015	-1	0.140	0.153	0.403	0	0	0
	27	0.200	0.190	0.413	0	0	0
	88	0.240	0.473	0.563	0	0	0

Day 27 represents data collected from Day 27 to 32.

Appendix 12

Individual Neurobehavioral Evaluation: Neuromuscular

32025165
Sex: Male

1.49x10^12 vg Group 4	Day(s) Relative to Start Date	Neuromuscular Body Tone	Hindlimb Splay Trial1 (cm)	Hindlimb Splay Trial2 (cm)	Mn Hindlimb Splay (cm)
4009	-1	0	15.0	11.4	13.2
	27	0	8.1	7.5	7.8
	88	0	16.1	13.3	14.7
4010	-1	0	11.4	7.9	9.7
	27	0	9.7	10.5	10.1
	88	0	18.1	11.7	14.9
4011	-1	0	8.8	11.6	10.2
	27	0	12.2	11.4	11.8
	88	0	15.8	18.1	17.0
4012	-1	0	12.9	9.4	11.2
	27	0	12.3	14.6	13.5
	88	0	15.0	12.2	13.6
4013	-1	0	12.6	9.6	11.1
	27	0	9.8	9.4	9.6
	88	0	14.1	11.8	13.0
4014	-1	0	11.0	13.0	12.0
	27	0	9.6	9.3	9.5
	88	0	18.8	18.0	18.4
4015	-1	0	14.1	11.4	12.8
	27	0	12.3	11.5	11.9
	88	0	17.5	11.1	14.3

Day 27 represents data collected from Day 27 to 32.

Appendix 12

Individual Neurobehavioral Evaluation: Neuromuscular

32025165

Sex: Female

0 vg		Neuromuscular					
Group 1	Day(s) Relative to Start Date	Gait/ Mobility	Forelimb Grp Strength kg1 (Kg)	Forelimb Grp Strength kg2 (Kg)	Forelimb Grp Strength kg3 (Kg)	Hindlimb Grp Strength kg1 (Kg)	Hindlimb Grp Strength kg2 (Kg)
1501	-1	0	0.700	0.520	0.370	0.220	0.360
	27	0	0.560	0.540	0.620	0.200	0.400
1502	-1	0	0.370	0.890	0.950	0.200	0.120
	27	0	0.670	0.380	0.180	0.660	0.440
1503	-1	0	0.350	0.550	0.610	0.400	0.310
	27	0	0.450	0.310	0.390	0.620	0.400
1504	-1	0	0.830	0.230	0.350	0.380	0.470
	27	0	0.240	0.400	0.320	0.360	0.040
1505	-1	0	0.160	0.390	0.310	0.170	0.190
	27	0	0.420	0.400	0.330	0.360	0.420
1506	-1	0	0.370	0.220	0.570	0.140	0.150
	27	0	0.540	0.490	0.760	0.300	0.270
	89	0	0.680	0.190	0.130	0.640	0.300
1507	-1	0	0.480	0.430	0.440	0.040	0.130
	27	0	0.700	0.660	0.550	0.400	0.610
	89	0	0.180	0.170	0.350	0.490	0.380
1508	-1	0	0.600	0.510	0.400	0.290	0.280
	27	0	0.890	0.620	0.490	0.370	0.380
	89	0	0.280	0.290	0.380	0.120	0.120

Day 27 represents data collected from Day 27 to 32.

Appendix 12

Individual Neurobehavioral Evaluation: Neuromuscular

32025165
Sex: Female

0 vg		Neuromuscular					
Group 1	Day(s) Relative to Start Date	Hindlimb Grp Strength kg3 (Kg)	Mn Hindlimb Grp Strength (Kg)	Mn Forelimb Grp Strength (Kg)	Air Right ing Reflex	Tremor (HC)	Tremor (OF)
1501	-1	0.360	0.313	0.530	0	0	0
	27	0.460	0.353	0.573	0	0	0
1502	-1	0.210	0.177	0.737	0	0	0
	27	0.400	0.500	0.410	0	0	0
1503	-1	0.650	0.453	0.503	0	0	0
	27	0.800	0.607	0.383	0	0	0
1504	-1	0.260	0.370	0.470	0	0	0
	27	0.260	0.220	0.320	0	0	0
1505	-1	0.250	0.203	0.287	0	0	0
	27	0.280	0.353	0.383	0	0	0
1506	-1	0.140	0.143	0.387	0	0	0
	27	0.260	0.277	0.597	0	0	0
	89	0.300	0.413	0.333	0	0	0
1507	-1	0.190	0.120	0.450	0	0	0
	27	0.430	0.480	0.637	0	0	0
	89	0.460	0.443	0.233	0	0	0
1508	-1	0.360	0.310	0.503	0	0	0
	27	0.170	0.307	0.667	0	0	0
	89	0.150	0.130	0.317	0	0	0

Day 27 represents data collected from Day 27 to 32.

Appendix 12

Individual Neurobehavioral Evaluation: Neuromuscular

32025165
Sex: Female

0 vg		Neuromuscular			
Group 1	Day(s) Relative to Start Date	Body Tone	Hindlimb Splay Trial1 (cm)	Hindlimb Splay Trial2 (cm)	Mn Hindlimb Splay (cm)
1501	-1	0	2.7	6.1	4.4
	27	0	12.5	11.2	11.9
1502	-1	0	10.0	6.6	8.3
	27	0	10.0	7.6	8.8
1503	-1	0	9.5	10.3	9.9
	27	0	11.6	10.8	11.2
1504	-1	0	14.7	15.0	14.9
	27	0	6.0	14.8	10.4
1505	-1	1	14.3	13.0	13.7
	27	0	10.3	10.8	10.6
1506	-1	0	10.1	11.0	10.6
	27	0	11.8	10.9	11.4
	89	0	13.5	9.2	11.4
1507	-1	0	12.2	9.6	10.9
	27	0	11.2	8.1	9.7
	89	0	11.5	10.5	11.0
1508	-1	0	12.3	12.6	12.5
	27	0	12.1	8.6	10.4
	89	0	10.7	10.5	10.6

Day 27 represents data collected from Day 27 to 32.

Appendix 12

Individual Neurobehavioral Evaluation: Neuromuscular

32025165
Sex: Female

0 vg		Neuromuscular					
Group 1	Day(s) Relative to Start Date	Gait/ Mobility	Forelimb Grp Strength kg1 (Kg)	Forelimb Grp Strength kg2 (Kg)	Forelimb Grp Strength kg3 (Kg)	Hindlimb Grp Strength kg1 (Kg)	Hindlimb Grp Strength kg2 (Kg)
1509	-1	0	0.750	0.490	0.600	0.230	0.510
	27	0	0.270	0.500	0.510	0.200	0.210
	89	0	0.120	0.240	0.350	0.380	0.350
1510	-1	0	0.280	0.360	0.600	0.140	0.260
	27	0	0.420	0.510	0.320	0.390	0.590
	89	0	0.400	0.240	0.310	0.310	0.350

Day 27 represents data collected from Day 27 to 32.

Appendix 12

Individual Neurobehavioral Evaluation: Neuromuscular

32025165
Sex: Female

0 vg		Neuromuscular					
Group 1	Day(s) Relative to Start Date	Hindlimb Grp Strength kg3 (Kg)	Mn Hindlimb Grp Strength (Kg)	Mn Forelimb Grp Strength (Kg)	Air Right ing Reflex	Tremor (HC)	Tremor (OF)
1509	-1	0.490	0.410	0.613	0	0	0
	27	0.300	0.237	0.427	0	0	0
	89	0.320	0.350	0.237	0	0	0
1510	-1	0.180	0.193	0.413	0	0	0
	27	0.290	0.423	0.417	0	0	0
	89	0.700	0.453	0.317	0	0	0

Day 27 represents data collected from Day 27 to 32.

Appendix 12

Individual Neurobehavioral Evaluation: Neuromuscular

32025165
Sex: Female

0 vg		Neuromuscular			
Group 1	Day(s) Relative to Start Date	Body Tone	Hindlimb Splay Trial1 (cm)	Hindlimb Splay Trial2 (cm)	Mn Hindlimb Splay (cm)
1509	-1	0	8.5	6.8	7.7
	27	0	6.1	9.1	7.6
	89	0	13.5	10.4	12.0
1510	-1	0	10.2	10.5	10.4
	27	0	10.8	11.7	11.3
	89	0	12.7	13.0	12.9

Day 27 represents data collected from Day 27 to 32.

Appendix 12

Individual Neurobehavioral Evaluation: Neuromuscular

32025165
Sex: Female

1.49x10^11 vg Group 2	Day(s) Relative to Start Date	Neuromuscular					
		Gait/ Mobility	Forelimb Grp Strength kg1 (Kg)	Forelimb Grp Strength kg2 (Kg)	Forelimb Grp Strength kg3 (Kg)	Hindlimb Grp Strength kg1 (Kg)	Hindlimb Grp Strength kg2 (Kg)
2501	-1	0	0.400	0.440	0.460	0.080	0.380
	27	0	0.670	0.310	0.640	0.120	0.270
	89	0	0.360	0.280	0.280	0.230	0.220
2502	-1	0	0.580	0.400	0.590	0.140	0.310
	27	0	0.240	0.890	0.460	0.050	0.150
	89	0	0.550	0.320	0.140	0.080	0.180
2503	-1	0	0.580	0.510	0.530	0.190	0.140
	27	0	0.880	1.020	0.510	0.670	0.220
	89	0	0.500	0.340	0.220	0.230	0.260
2504	-1	0	0.580	0.420	0.420	0.070	0.250
	27	0	0.780	0.520	0.430	0.130	0.180
	89	0	0.410	0.830	0.330	0.290	0.180
2505	-1	0	0.460	0.480	0.660	0.140	0.040
	27	0	0.700	0.640	0.490	0.310	0.400
	89	0	0.600	0.260	0.480	0.140	0.180
2506	-1	0	0.750	0.570	0.730	0.140	0.140
	27	0	1.030	0.680	0.640	0.160	0.100
	89	0	0.350	0.200	0.550	0.630	0.450
2507	-1	0	0.600	0.640	0.650	0.160	0.350
	27	0	0.620	0.520	0.330	0.380	0.610
	89	0	0.150	0.210	0.150	0.290	0.440

Day 27 represents data collected from Day 27 to 32.

Appendix 12

Individual Neurobehavioral Evaluation: Neuromuscular

32025165
Sex: Female

1.49x10^11 vg<br>Group 2	Day(s) Relative to Start Date	Neuromuscular					
		Hindlimb Grp Strength kg3 (Kg)	Mn Hindlimb Grp Strength (Kg)	Mn Forelimb Grp Strength (Kg)	Air Right ing Reflex	Tremor (HC)	Tremor (OF)
2501	-1	0.210	0.223	0.433	0	0	0
	27	0.140	0.177	0.540	0	0	0
	89	0.340	0.263	0.307	0	0	0
2502	-1	0.430	0.293	0.523	0	0	0
	27	0.110	0.103	0.530	0	0	0
	89	0.230	0.163	0.337	0	0	0
2503	-1	0.190	0.173	0.540	0	0	0
	27	0.550	0.480	0.803	0	0	0
	89	0.200	0.230	0.353	0	0	0
2504	-1	0.150	0.157	0.473	0	0	0
	27	0.260	0.190	0.577	0	0	0
	89	0.130	0.200	0.523	0	0	0
2505	-1	0.220	0.133	0.533	0	0	0
	27	0.290	0.333	0.610	0	0	0
	89	0.140	0.153	0.447	0	0	0
2506	-1	0.300	0.193	0.683	0	0	0
	27	0.070	0.110	0.783	0	0	0
	89	0.350	0.477	0.367	0	0	0
2507	-1	0.110	0.207	0.630	0	0	0
	27	0.300	0.430	0.490	0	0	0
	89	0.480	0.403	0.170	0	0	0

Day 27 represents data collected from Day 27 to 32.

Appendix 12

Individual Neurobehavioral Evaluation: Neuromuscular

32025165
Sex: Female

1.49x10^11 vg Group 2	Day(s) Relative to Start Date	Neuromuscular Body Tone	Hindlimb Splay Trial1 (cm)	Hindlimb Splay Trial2 (cm)	Mn Hindlimb Splay (cm)
2501	-1	0	12.8	9.2	11.0
	27	0	11.3	9.7	10.5
	89	0	12.0	11.0	11.5
2502	-1	0	13.4	13.5	13.5
	27	0	10.0	8.2	9.1
	89	0	13.4	9.5	11.5
2503	-1	0	8.4	14.4	11.4
	27	0	15.1	13.4	14.3
	89	0	12.7	10.4	11.6
2504	-1	0	9.6	8.8	9.2
	27	0	11.1	8.6	9.9
	89	0	13.8	6.2	10.0
2505	-1	0	6.9	8.8	7.9
	27	0	9.9	11.3	10.6
	89	0	10.3	6.5	8.4
2506	-1	0	12.0	13.1	12.6
	27	0	12.8	11.4	12.1
	89	0	15.4	13.2	14.3
2507	-1	0	11.1	7.6	9.4
	27	0	10.0	9.7	9.9
	89	0	13.2	13.3	13.3

Day 27 represents data collected from Day 27 to 32.

Appendix 12

Individual Neurobehavioral Evaluation: Neuromuscular

32025165
Sex: Female

1.49x10^11 vg Group 2	Day(s) Relative to Start Date	Neuromuscular					
		Gait/ Mobility	Forelimb Grp Strength kg1 (Kg)	Forelimb Grp Strength kg2 (Kg)	Forelimb Grp Strength kg3 (Kg)	Hindlimb Grp Strength kg1 (Kg)	Hindlimb Grp Strength kg2 (Kg)
2508	-1	0	0.420	0.560	0.420	0.170	0.160
	27	0	0.400	0.410	0.350	0.330	0.390
	89	0	0.210	0.150	0.150	0.430	0.450
2509	-1	0	0.370	0.780	0.930	0.140	0.340
	27	0	0.520	0.440	0.610	0.310	0.500
	89	0	0.480	0.450	0.380	0.250	0.240
2510	-1	0	0.400	0.600	0.690	0.120	0.100
	27	0	0.280	0.240	0.340	0.450	0.350
	89	0	0.150	0.220	0.350	0.320	0.230

Day 27 represents data collected from Day 27 to 32.

Appendix 12

Individual Neurobehavioral Evaluation: Neuromuscular

32025165
Sex: Female

1.49x10^11 vg Group 2	Day(s) Relative to Start Date	Neuromuscular					
		Hindlimb Grp Strength kg3 (Kg)	Mn Hindlimb Grp Strength (Kg)	Mn Forelimb Grp Strength (Kg)	Air Right ing Reflex	Tremor (HC)	Tremor (OF)
2508	-1	0.210	0.180	0.467	0	0	0
	27	0.070	0.263	0.387	0	0	0
	89	0.200	0.360	0.170	0	0	0
2509	-1	0.400	0.293	0.693	0	0	0
	27	0.500	0.437	0.523	0	0	0
	89	0.210	0.233	0.437	0	0	0
2510	-1	0.150	0.123	0.563	0	0	0
	27	0.130	0.310	0.287	0	0	0
	89	0.170	0.240	0.240	0	0	0

Day 27 represents data collected from Day 27 to 32.

Appendix 12

Individual Neurobehavioral Evaluation: Neuromuscular

32025165
Sex: Female

1.49x10^11 vg Group 2	Day(s) Relative to Start Date	Neuromuscular			
		Body Tone	Hindlimb Splay Trial1 (cm)	Hindlimb Splay Trial2 (cm)	Mn Hindlimb Splay (cm)
2508	-1	0	8.0	7.4	7.7
	27	0	11.0	8.7	9.9
	89	0	9.7	6.5	8.1
2509	-1	0	11.1	7.5	9.3
	27	0	12.8	9.4	11.1
	89	0	14.3	9.7	12.0
2510	-1	0	11.1	7.4	9.3
	27	0	6.7	5.6	6.2
	89	0	9.7	8.3	9.0

Day 27 represents data collected from Day 27 to 32.

Appendix 12

Individual Neurobehavioral Evaluation: Neuromuscular

32025165
Sex: Female

4.47x10^11 vg Group 3	Day(s) Relative to Start Date	Neuromuscular Gait/ Mobility	Forelimb Grp Strength kg1 (Kg)	Forelimb Grp Strength kg2 (Kg)	Forelimb Grp Strength kg3 (Kg)	Hindlimb Grp Strength kg1 (Kg)	Hindlimb Grp Strength kg2 (Kg)
3501	-1	0	0.760	0.660	0.520	0.090	0.080
	27	0	0.720	0.660	0.710	0.590	0.540
	89	0	0.860	0.650	0.910	0.160	0.150
3502	-1	0	0.180	0.770	0.670	0.340	0.650
	27	0	0.790	0.900	0.450	0.440	0.450
	89	0	0.450	0.190	0.240	0.270	0.380
3503	-1	0	0.610	0.450	0.510	0.070	0.150
	27	0	0.410	0.300	0.270	0.370	0.400
	89	0	0.630	0.470	0.380	0.080	0.070
3504	-1	0	0.680	0.860	0.610	0.810	0.410
	27	0	0.350	0.510	0.250	0.180	0.130
	89	0	0.130	0.550	0.330	0.120	0.270
3505	-1	0	0.280	0.560	0.340	0.350	0.380
	27	0	0.240	0.600	0.420	0.390	0.310
	89	0	0.580	0.450	0.500	0.130	0.110
3506	-1	0	0.390	0.280	0.310	0.170	0.120
	27	0	0.360	0.740	0.650	0.280	0.370
	89	0	0.610	0.230	0.140	0.240	0.160
3507	-1	0	0.300	0.400	0.580	0.490	0.510
	27	0	0.770	0.900	0.320	0.230	0.250
	89	0	0.390	0.300	0.550	0.420	0.490

Day 27 represents data collected from Day 27 to 32.

Appendix 12

Individual Neurobehavioral Evaluation: Neuromuscular

32025165
Sex: Female

4.47x10^11 vg<br>Group 3	Day(s) Relative to Start Date	Neuromuscular					
		Hindlimb Grp Strength kg3 (Kg)	Mn Hindlimb Grp Strength (Kg)	Mn Forelimb Grp Strength (Kg)	Air Right ing Reflex	Tremor (HC)	Tremor (OF)
3501	-1	0.120	0.097	0.647	0	0	0
	27	0.530	0.553	0.697	0	0	0
	89	0.200	0.170	0.807	0	0	0
3502	-1	0.510	0.500	0.540	0	0	0
	27	0.470	0.453	0.713	0	0	0
	89	0.270	0.307	0.293	0	0	0
3503	-1	0.140	0.120	0.523	0	0	0
	27	0.450	0.407	0.327	0	0	0
	89	0.150	0.100	0.493	0	0	0
3504	-1	0.450	0.557	0.717	0	0	0
	27	0.260	0.190	0.370	0	0	0
	89	0.190	0.193	0.337	0	0	0
3505	-1	0.480	0.403	0.393	0	0	0
	27	0.240	0.313	0.420	0	0	0
	89	0.085	0.108	0.510	0	0	0
3506	-1	0.180	0.157	0.327	0	0	0
	27	0.480	0.377	0.583	0	0	0
	89	0.200	0.200	0.327	0	0	0
3507	-1	0.330	0.443	0.427	0	0	0
	27	0.360	0.280	0.663	0	0	0
	89	0.300	0.403	0.413	0	0	0

Day 27 represents data collected from Day 27 to 32.

Appendix 12

Individual Neurobehavioral Evaluation: Neuromuscular

32025165

Sex: Female

4.47x10^11 vg Group 3	Day(s) Relative to Start Date	Neuromuscular Body Tone	Hindlimb Splay Trial1 (cm)	Hindlimb Splay Trial2 (cm)	Mn Hindlimb Splay (cm)
3501	-1	0	9.5	11.9	10.7
	27	0	12.1	12.5	12.3
	89	0	11.7	12.8	12.3
3502	-1	0	8.9	7.6	8.3
	27	0	11.8	10.9	11.4
	89	0	13.3	8.5	10.9
3503	-1	0	11.0	9.3	10.2
	27	0	14.1	9.3	11.7
	89	0	13.0	10.4	11.7
3504	-1	0	3.5	7.1	5.3
	27	0	8.1	7.5	7.8
	89	0	9.1	8.7	8.9
3505	-1	0	11.6	12.3	12.0
	27	0	12.7	10.0	11.4
	89	0	7.8	9.5	8.7
3506	-1	0	8.1	7.6	7.9
	27	0	12.6	6.6	9.6
	89	0	11.0	14.7	12.9
3507	-1	0	9.3	8.2	8.8
	27	0	11.1	11.0	11.1
	89	0	11.0	8.5	9.8

Day 27 represents data collected from Day 27 to 32.

Appendix 12

Individual Neurobehavioral Evaluation: Neuromuscular

32025165
Sex: Female

4.47x10^11 vg Group 3	Day(s) Relative to Start Date	Neuromuscular					
		Gait/ Mobility	Forelimb Grp Strength kg1 (Kg)	Forelimb Grp Strength kg2 (Kg)	Forelimb Grp Strength kg3 (Kg)	Hindlimb Grp Strength kg1 (Kg)	Hindlimb Grp Strength kg2 (Kg)
3508	-1	0	0.900	0.960	1.080	0.620	0.530
	27	0	0.360	0.370	0.270	0.310	0.220
	89	0	0.340	0.560	0.190	0.120	0.130
3509	-1	0	0.370	0.520	0.690	0.350	0.390
	27	0	0.280	0.280	0.490	0.180	0.140
	89	0	0.470	0.450	0.160	0.220	0.550
3510	-1	0	0.460	0.270	0.690	0.170	0.050
	27	0	0.500	0.400	0.330	0.600	0.680
	89	0	0.070	0.130	0.130	0.200	0.150

Day 27 represents data collected from Day 27 to 32.

Appendix 12

Individual Neurobehavioral Evaluation: Neuromuscular

32025165

Sex: Female

4.47x10^11 vg Group 3	Day(s) Relative to Start Date	Neuromuscular					
		Hindlimb Grp Strength kg3 (Kg)	Mn Hindlimb Grp Strength (Kg)	Mn Forelimb Grp Strength (Kg)	Air Right ing Reflex	Tremor (HC)	Tremor (OF)
3508	-1	0.640	0.597	0.980	0	0	0
	27	0.510	0.347	0.333	0	0	0
	89	0.080	0.110	0.363	0	0	0
3509	-1	0.370	0.370	0.527	0	0	0
	27	0.220	0.180	0.350	0	0	0
	89	0.120	0.297	0.360	0	0	0
3510	-1	0.120	0.113	0.473	0	0	0
	27	0.530	0.603	0.410	0	0	0
	89	0.150	0.167	0.110	0	0	0

Day 27 represents data collected from Day 27 to 32.

Appendix 12

Individual Neurobehavioral Evaluation: Neuromuscular

32025165

Sex: Female

4.47x10^11 vg<br>Group 3	Day(s) Relative to Start Date	Neuromuscular Body Tone	Hindlimb Splay Trial1 (cm)	Hindlimb Splay Trial2 (cm)	Mn Hindlimb Splay (cm)
3508	-1	0	10.8	11.7	11.3
	27	0	8.2	7.1	7.7
	89	0	9.7	7.6	8.7
3509	-1	0	9.0	10.2	9.6
	27	0	11.8	10.2	11.0
	89	0	9.3	11.7	10.5
3510	-1	0	11.0	8.8	9.9
	27	0	9.1	8.9	9.0
	89	0	9.3	8.3	8.8

Day 27 represents data collected from Day 27 to 32.

Appendix 12

Individual Neurobehavioral Evaluation: Neuromuscular

32025165
Sex: Female

1.49x10^12 vg Group 4	Day(s) Relative to Start Date	Neuromuscular Gait/ Mobility	Forelimb Grp Strength kg1 (Kg)	Forelimb Grp Strength kg2 (Kg)	Forelimb Grp Strength kg3 (Kg)	Hindlimb Grp Strength kg1 (Kg)	Hindlimb Grp Strength kg2 (Kg)
4501	-1	0	0.750	0.360	0.860	0.140	0.260
	27	0	0.370	0.310	0.240	0.140	0.220
4502	-1	0	0.550	0.590	0.370	0.190	0.310
	27	0	0.810	0.870	0.480	0.600	0.280
4503	-1	0	0.670	1.070	0.430	0.210	0.120
	27	0	0.910	0.560	0.380	0.520	0.300
4504	-1	0	0.700	0.280	0.490	0.130	0.250
	27	0	0.400	0.240	0.240	0.040	0.120
4505	-1	0	0.550	0.510	0.500	0.140	0.220
	27	0	0.410	0.270	0.410	0.300	0.240
4506	-1	0	0.330	0.330	0.470	0.330	0.260
	27	0	0.640	0.680	0.670	0.160	0.100
	89	0	0.180	0.340	0.320	0.130	0.220
4507	-1	0	0.350	0.500	0.250	0.190	0.300
	27	0	0.670	0.430	0.350	0.100	0.300
	89	0	0.330	0.410	0.330	0.080	0.170
4508	-1	0	0.980	0.330	0.920	0.480	0.250
	27	0	0.940	0.880	0.800	0.400	0.250
	89	0	0.910	0.610	0.950	0.390	0.290

Day 27 represents data collected from Day 27 to 32.

Appendix 12

Individual Neurobehavioral Evaluation: Neuromuscular

32025165
Sex: Female

1.49x10^12 vg<br>Group 4	Day(s) Relative to Start Date	Neuromuscular<br>Hindlimb Grp Strength kg3 (Kg)	Mn Hindlimb Grp Strength (Kg)	Mn Forelimb Grp Strength (Kg)	Air Right ing Reflex	Tremor (HC)	Tremor (OF)
4501	-1	0.260	0.220	0.657	0	0	0
	27	0.200	0.187	0.307	0	0	0
4502	-1	0.310	0.270	0.503	0	0	0
	27	0.460	0.447	0.720	0	0	0
4503	-1	0.240	0.190	0.723	0	0	0
	27	0.420	0.413	0.617	0	0	0
4504	-1	0.130	0.170	0.490	0	0	0
	27	0.300	0.153	0.293	0	0	0
4505	-1	0.310	0.223	0.520	0	0	0
	27	0.180	0.240	0.363	0	0	0
4506	-1	0.330	0.307	0.377	0	0	0
	27	0.120	0.127	0.663	0	0	0
	89	0.120	0.157	0.280	0	0	0
4507	-1	0.290	0.260	0.367	0	0	0
	27	0.270	0.223	0.483	0	0	0
	89	0.180	0.143	0.357	0	0	0
4508	-1	0.420	0.383	0.743	0	0	0
	27	0.440	0.363	0.873	0	0	0
	89	0.350	0.343	0.823	0	0	0

Day 27 represents data collected from Day 27 to 32.

Appendix 12

Individual Neurobehavioral Evaluation: Neuromuscular

32025165
Sex: Female

1.49x10^12 vg Group 4	Day(s) Relative to Start Date	Neuromuscular: Body Tone	Neuromuscular: Hindlimb Splay Trial1 (cm)	Neuromuscular: Hindlimb Splay Trial2 (cm)	Neuromuscular: Mn Hindlimb Splay (cm)
4501	-1	0	13.1	12.6	12.9
	27	0	12.4	10.7	11.6
4502	-1	0	10.8	10.2	10.5
	27	0	12.1	13.4	12.8
4503	-1	0	11.9	12.0	12.0
	27	0	11.6	10.9	11.3
4504	-1	0	10.7	10.6	10.7
	27	0	9.6	8.8	9.2
4505	-1	0	12.9	9.6	11.3
	27	0	13.8	11.8	12.8
4506	-1	0	6.7	4.9	5.8
	27	0	7.5	7.8	7.7
	89	0	8.2	7.6	7.9
4507	-1	0	9.4	6.5	8.0
	27	0	4.9	4.9	4.9
	89	0	5.9	7.0	6.5
4508	-1	0	12.7	8.1	10.4
	27	0	12.6	12.3	12.5
	89	0	8.2	14.2	11.2

Day 27 represents data collected from Day 27 to 32.

Appendix 12

Individual Neurobehavioral Evaluation: Neuromuscular

32025165
Sex: Female

1.49x10^12 vg Group 4	Day(s) Relative to Start Date	Neuromuscular					
		Gait/ Mobility	Forelimb Grp Strength kg1 (Kg)	Forelimb Grp Strength kg2 (Kg)	Forelimb Grp Strength kg3 (Kg)	Hindlimb Grp Strength kg1 (Kg)	Hindlimb Grp Strength kg2 (Kg)
4509	-1	0	0.470	0.610	0.460	0.040	0.130
	27	0	0.270	0.570	0.540	0.190	0.350
	89	0	0.190	0.380	0.280	0.160	0.220
4510	-1	0	0.840	0.750	0.440	0.450	0.530
	27	0	0.540	0.340	0.140	0.310	0.240
	89	0	0.140	0.110	0.330	0.130	0.020
4511	-1	0	0.490	0.730	0.420	0.480	0.410
	27	0	0.550	0.430	0.780	0.090	0.130
	89	0	0.280	0.150	0.090	0.180	0.160
4512	-1	0	0.550	0.770	0.610	0.190	0.290
	27	0	0.620	0.920	0.500	0.330	0.300
	89	0	0.700	0.120	0.100	0.350	0.400
4513	-1	0	0.330	0.180	0.120	0.450	0.370
	27	0	0.420	0.380	0.160	0.590	0.470
	89	0	0.160	0.120	0.160	0.380	0.280
4514	-1	0	0.540	0.520	0.230	0.290	0.290
	27	0	0.200	0.300	0.510	0.220	0.290
	89	0	0.940	0.460	0.280	0.350	0.230
4515	-1	0	0.350	0.700	0.280	0.090	0.070
	27	0	0.900	1.010	1.050	0.140	0.220
	89	0	0.600	0.690	0.770	0.180	0.290

Day 27 represents data collected from Day 27 to 32.

Appendix 12

Individual Neurobehavioral Evaluation: Neuromuscular

32025165
Sex: Female

1.49x10^12 vg<br>Group 4	Day(s) Relative to Start Date	Neuromuscular<br>Hindlimb Grp Strength kg3 (Kg)	Mn Hindlimb Grp Strength (Kg)	Mn Forelimb Grp Strength (Kg)	Air Right ing Reflex	Tremor (HC)	Tremor (OF)
4509	-1	0.090	0.087	0.513	0	0	0
	27	0.130	0.223	0.460	0	0	0
	89	0.130	0.170	0.283	0	0	0
4510	-1	0.480	0.487	0.677	0	0	0
	27	0.310	0.287	0.340	0	0	0
	89	0.030	0.060	0.193	0	0	0
4511	-1	0.340	0.410	0.547	0	0	0
	27	0.090	0.103	0.587	0	0	0
	89	0.150	0.163	0.173	0	0	0
4512	-1	0.220	0.233	0.643	0	0	0
	27	0.470	0.367	0.680	0	0	0
	89	0.350	0.367	0.307	0	0	0
4513	-1	0.420	0.413	0.210	0	0	0
	27	0.580	0.547	0.320	0	0	0
	89	0.240	0.300	0.147	0	0	0
4514	-1	0.140	0.240	0.430	0	0	0
	27	0.200	0.237	0.337	0	0	0
	89	0.320	0.300	0.560	0	0	0
4515	-1	0.160	0.107	0.443	0	0	0
	27	0.110	0.157	0.987	0	0	0
	89	0.170	0.213	0.687	0	0	0

Day 27 represents data collected from Day 27 to 32.

Appendix 12

Individual Neurobehavioral Evaluation: Neuromuscular

32025165

Sex: Female

1.49x10^12 vg Group 4	Day(s) Relative to Start Date	Neuromuscular Body Tone	Hindlimb Splay Trial1 (cm)	Hindlimb Splay Trial2 (cm)	Mn Hindlimb Splay (cm)
4509	-1	0	12.8	13.9	13.4
	27	0	10.4	11.6	11.0
	89	0	15.5	15.4	15.5
4510	-1	0	7.2	6.8	7.0
	27	0	6.2	8.5	7.4
	89	0	6.0	8.0	7.0
4511	-1	0	9.7	9.7	9.7
	27	0	10.1	6.2	8.2
	89	0	7.3	5.0	6.2
4512	-1	0	11.6	13.5	12.6
	27	0	12.6	12.5	12.6
	89	0	12.7	11.0	11.9
4513	-1	0	6.1	6.8	6.5
	27	0	8.6	9.0	8.8
	89	0	10.5	8.7	9.6
4514	-1	0	10.0	10.2	10.1
	27	0	7.2	9.8	8.5
	89	0	11.7	8.4	10.1
4515	-1	0	10.2	12.1	11.2
	27	0	12.0	12.2	12.1
	89	0	10.7	10.0	10.4

Day 27 represents data collected from Day 27 to 32.

Appendix 13

Individual Neurobehavioral Evaluation: Physiological

32025165
Sex: Male

0 vg		Physiological	
Group 1	Day(s) Relative to Start Date	Respiration	Body Temperature (oC)
1001	-1	0	36.3
	27	0	35.7
1002	-1	0	36.5
	27	0	35.2
1003	-1	0	36.3
	27	0	35.8
1004	-1	0	36.7
	27	0	37.3
1005	-1	0	36.0
	27	0	35.7
1006	-1	0	36.6
	27	0	36.6
	88	0	37.6
1007	-1	0	37.2
	27	0	36.8
	88	0	36.8
1008	-1	0	36.4
	27	0	37.6
	88	0	37.4

Day 27 represents data collected from Day 27 to 32.

Appendix 13

Individual Neurobehavioral Evaluation: Physiological

32025165
Sex: Male

0 vg		Physiological	
Group 1	Day(s) Relative to Start Date	Respiration	Body Temperature (oC)
1009	-1	0	36.9
	27	0	36.8
	88	0	36.7
1010	-1	0	36.2
	27	0	37.0
	88	0	37.1

Day 27 represents data collected from Day 27 to 32.

Appendix 13

Individual Neurobehavioral Evaluation: Physiological

32025165
Sex: Male

1.49x10^11 vg Group 2	Day(s) Relative to Start Date	Physiological Respiration	Physiological Body Temperature (oC)
2001	-1	0	36.9
	27	0	36.3
	88	0	36.7
2002	-1	0	34.9
	27	0	36.3
	88	0	36.5
2003	-1	0	36.7
	27	0	37.5
	88	0	36.9
2004	-1	0	37.0
	27	0	36.6
	88	0	36.3
2005	-1	0	37.1
	27	0	37.5
	88	0	36.6
2006	-1	0	36.6
	27	0	37.0
	88	0	36.9
2007	-1	0	37.6
	27	0	37.9
	88	0	37.0

Day 27 represents data collected from Day 27 to 32.

Appendix 13

Individual Neurobehavioral Evaluation: Physiological

32025165

Sex: Male

1.49x10^11 vg<br>Group 2	Day(s) Relative to Start Date	Physiological	
		Respiration	Body Temperature (oC)
2008	-1	0	38.2
	27	0	38.0
	88	0	36.4
2009	-1	0	37.3
	27	0	37.3
	88	0	36.5
2010	-1	0	36.0
	27	0	36.6
	88	0	36.5

Day 27 represents data collected from Day 27 to 32.

Appendix 13

Individual Neurobehavioral Evaluation: Physiological

32025165
Sex: Male

4.47x10^11 vg<br>Group 3	Day(s) Relative to Start Date	Physiological	
		Respiration	Body Temperature (oC)
3001	-1	0	37.0
	27	0	36.2
	88	0	36.5
3002	-1	0	37.3
	27	0	36.4
	88	0	36.7
3003	-1	0	37.6
	27	0	36.9
	88	0	36.2
3004	-1	0	36.2
	27	0	36.0
	88	0	36.5
3005	-1	0	36.0
	27	0	36.6
	88	0	36.2
3006	-1	0	36.0
	27	0	36.5
	88	0	36.0
3007	-1	0	36.8
	27	0	37.3
	88	0	36.0

Day 27 represents data collected from Day 27 to 32.

Appendix 13

Individual Neurobehavioral Evaluation: Physiological

32025165
Sex: Male

4.47x10^11 vg<br>Group 3	Day(s) Relative to Start Date	Physiological	
		Respiration	Body Temperature (oC)
3008	-1	0	36.4
	27	0	36.8
	88	0	36.1
3009	-1	0	37.0
	27	0	37.1
	88	0	36.3
3010	-1	0	36.2
	27	0	36.9
	88	0	36.0

Day 27 represents data collected from Day 27 to 32.

Appendix 13

Individual Neurobehavioral Evaluation: Physiological

32025165

Sex: Male

1.49x10^12 vg Group 4	Day(s) Relative to Start Date	Physiological	
		Respiration	Body Temperature (oC)
4001	-1	0	35.0
	27	0	35.1
4002	-1	0	35.2
	27	0	34.9
4003	-1	0	34.2
	27	0	34.5
4004	-1	0	34.0
	27	0	36.2
4005	-1	0	34.0
	27	0	35.6
4006	-1	0	36.5
	27	0	36.5
	88	0	36.5
4007	-1	0	35.5
	27	0	37.1
	88	0	36.4
4008	-1	0	37.6
	27	0	36.5
	88	0	36.6

Day 27 represents data collected from Day 27 to 32.

Appendix 13

Individual Neurobehavioral Evaluation: Physiological

32025165
Sex: Male

1.49x10^12 vg<br>Group 4	Day(s) Relative to Start Date	Physiological	
		Respiration	Body Temperature (oC)
4009	-1	0	36.4
	27	0	36.7
	88	0	36.0
4010	-1	0	36.0
	27	0	37.5
	88	0	36.0
4011	-1	0	36.9
	27	0	36.3
	88	0	35.7
4012	-1	0	36.6
	27	0	37.8
	88	0	38.4
4013	-1	0	36.4
	27	0	36.1
	88	0	36.6
4014	-1	0	36.6
	27	0	36.6
	88	0	36.1
4015	-1	0	37.8
	27	0	37.3
	88	0	36.8

Day 27 represents data collected from Day 27 to 32.

Appendix 13

Individual Neurobehavioral Evaluation: Physiological

32025165

Sex: Female

0 vg		Physiological	
Group 1	Day(s) Relative to Start Date	Respiration	Body Temperature (oC)
1501	-1	0	37.6
	27	0	38.9
1502	-1	0	36.8
	27	0	37.6
1503	-1	0	38.2
	27	0	39.0
1504	-1	0	35.9
	27	0	36.7
1505	-1	0	35.5
	27	0	38.3
1506	-1	0	37.4
	27	0	38.0
	89	0	37.5
1507	-1	0	38.1
	27	0	37.2
	89	0	38.2
1508	-1	0	36.4
	27	0	36.5
	89	0	37.8

Day 27 represents data collected from Day 27 to 32.

Appendix 13

Individual Neurobehavioral Evaluation: Physiological

32025165
Sex: Female

0 vg		Physiological	
Group 1	Day(s) Relative to Start Date	Respiration	Body Temperature (oC)
1509	-1	0	37.2
	27	0	37.0
	89	0	37.3
1510	-1	0	37.2
	27	0	36.2
	89	0	37.0

Day 27 represents data collected from Day 27 to 32.

Appendix 13

Individual Neurobehavioral Evaluation: Physiological

32025165
Sex: Female

1.49x10^11 vg Group 2	Day(s) Relative to Start Date	Physiological	
		Respiration	Body Temperature (oC)
2501	-1	0	36.7
	27	0	37.0
	89	0	38.0
2502	-1	0	36.5
	27	0	37.4
	89	0	37.4
2503	-1	0	37.9
	27	0	37.0
	89	0	37.8
2504	-1	0	38.8
	27	0	37.9
	89	0	38.8
2505	-1	0	38.3
	27	0	37.9
	89	0	38.2
2506	-1	0	37.8
	27	0	36.3
	89	0	37.4
2507	-1	0	37.6
	27	0	37.0
	89	0	36.7

Day 27 represents data collected from Day 27 to 32.

Appendix 13

Individual Neurobehavioral Evaluation: Physiological

32025165
Sex: Female

1.49x10^11 vg<br>Group 2	Day(s) Relative to Start Date	Physiological	
		Respiration	Body Temperature (oC)
2508	-1	0	37.6
	27	0	37.9
	89	0	37.8
2509	-1	0	36.1
	27	0	37.0
	89	0	37.8
2510	-1	0	37.4
	27	1	37.7
	89	0	37.2

Day 27 represents data collected from Day 27 to 32.

Appendix 13

Individual Neurobehavioral Evaluation: Physiological

32025165
Sex: Female

4.47x10^11 vg<br><br>Group 3	Day(s) Relative to Start Date	Physiological	
		Respiration	Body Temperature (oC)
3501	-1	0	38.2
	27	0	37.1
	89	0	38.2
3502	-1	0	36.2
	27	0	38.2
	89	0	37.6
3503	-1	0	38.4
	27	0	37.1
	89	0	37.9
3504	-1	0	36.5
	27	0	37.2
	89	0	37.1
3505	-1	0	36.0
	27	0	37.3
	89	0	37.2
3506	-1	0	37.2
	27	0	38.1
	89	0	37.5
3507	-1	0	36.7
	27	0	37.5
	89	0	37.7

Day 27 represents data collected from Day 27 to 32.

Appendix 13

Individual Neurobehavioral Evaluation: Physiological

32025165
Sex: Female

4.47x10^11 vg Group 3	Day(s) Relative to Start Date	Physiological	
		Respiration	Body Temperature (oC)
3508	-1	0	36.6
	27	0	37.9
	89	0	38.5
3509	-1	0	36.2
	27	0	37.1
	89	0	37.0
3510	-1	0	36.8
	27	0	37.9
	89	0	36.8

Day 27 represents data collected from Day 27 to 32.

Appendix 13

Individual Neurobehavioral Evaluation: Physiological

32025165
Sex: Female

1.49x10^12 vg Group 4	Day(s) Relative to Start Date	Physiological Respiration	Physiological Body Temperature (oC)
4501	-1	0	33.0
	27	0	34.8
4502	-1	0	35.1
	27	0	37.6
4503	-1	0	31.0
	27	0	38.8
4504	-1	0	33.7
	27	0	35.2
4505	-1	0	34.3
	27	0	36.8
4506	-1	0	36.0
	27	0	36.4
	89	0	37.2
4507	-1	0	38.3
	27	0	35.5
	89	0	38.3
4508	-1	0	37.1
	27	0	36.8
	89	0	37.8

Day 27 represents data collected from Day 27 to 32.

Appendix 13

Individual Neurobehavioral Evaluation: Physiological

32025165
Sex: Female

1.49x10^12 vg<br>Group 4	Day(s) Relative to Start Date	Physiological	
		Respiration	Body Temperature (oC)
4509	-1	0	37.1
	27	0	38.2
	89	0	38.7
4510	-1	0	36.0
	27	0	38.3
	89	0	38.5
4511	-1	0	35.2
	27	0	37.0
	89	0	38.2
4512	-1	0	37.4
	27	0	36.9
	89	0	37.3
4513	-1	0	36.9
	27	0	37.3
	89	0	36.8
4514	-1	0	37.3
	27	0	36.0
	89	0	38.6
4515	-1	0	38.7
	27	0	39.4
	89	0	38.5

Day 27 represents data collected from Day 27 to 32.

Appendix 14

Individual Neurobehavioral Evaluation: Sensorimotor

32025165
Sex: Male

0 vg<br>Group 1	Day(s) Relative to Start Date	Sensorimotor<br>Touch Resp/ Tactile Refl	Sensorimotor<br>Thermal Response (sec)
1001	-1	0	14.08
	27	0	5.66
1002	-1	0	11.75
	27	0	5.50
1003	-1	0	7.69
	27	0	7.75
1004	-1	0	9.40
	27	0	5.00
1005	-1	0	21.65
	27	0	11.68
1006	-1	0	9.53
	27	0	10.06
	88	0	7.44
1007	-1	0	16.47
	27	0	9.78
	88	0	4.84
1008	-1	0	7.65
	27	0	11.13
	88	0	4.13

Day 27 represents data collected from Day 27 to 32.

Appendix 14

Individual Neurobehavioral Evaluation: Sensorimotor

32025165
Sex: Male

0 vg Group 1	Day(s) Relative to Start Date	Sensorimotor Touch Resp/ Tactile Refl	Sensorimotor Thermal Response (sec)
1009	-1	0	10.19
	27	0	8.09
	88	0	8.32
1010	-1	0	11.21
	27	0	8.97
	88	0	7.00

Day 27 represents data collected from Day 27 to 32.

Appendix 14

Individual Neurobehavioral Evaluation: Sensorimotor

32025165
Sex: Male

1.49x10^11 vg<br>Group 2	Day(s) Relative to Start Date	Sensorimotor<br>Touch Resp/ Tactile Refl	Sensorimotor<br>Thermal Response (sec)
2001	-1	0	16.13
	27	0	10.69
	88	0	12.77
2002	-1	0	5.83
	27	0	7.38
	88	0	6.58
2003	-1	0	8.31
	27	0	4.25
	88	0	7.53
2004	-1	0	5.95
	27	0	8.90
	88	0	9.45
2005	-1	0	15.66
	27	0	7.19
	88	0	6.40
2006	-1	0	5.75
	27	0	9.51
	88	0	10.72
2007	-1	0	5.59
	27	0	6.06
	88	0	4.84

Day 27 represents data collected from Day 27 to 32.

Appendix 14

Individual Neurobehavioral Evaluation: Sensorimotor

32025165
Sex: Male

1.49x10^11 vg<br>Group 2	Day(s) Relative to Start Date	Sensorimotor<br>Touch Resp/ Tactile Refl	Sensorimotor<br>Thermal Response (sec)
2008	-1	0	10.77
	27	0	6.94
	88	0	6.02
2009	-1	0	11.25
	27	0	8.88
	88	0	9.90
2010	-1	0	6.39
	27	0	5.25
	88	0	10.37

Day 27 represents data collected from Day 27 to 32.

Appendix 14

Individual Neurobehavioral Evaluation: Sensorimotor

32025165
Sex: Male

4.47x10^11 vg<br>Group 3	Day(s) Relative to Start Date	Sensorimotor<br>Touch Resp/ Tactile Refl	Sensorimotor<br>Thermal Response (sec)
3001	-1	0	5.95
	27	0	5.57
	88	0	5.01
3002	-1	0	9.16
	27	0	13.78
	88	0	9.90
3003	-1	0	9.46
	27	0	11.75
	88	0	11.32
3004	-1	0	8.57
	27	0	6.07
	88	0	7.45
3005	-1	0	7.48
	27	0	5.78
	88	0	12.86
3006	-1	0	9.90
	27	0	11.41
	88	0	10.40
3007	-1	0	2.25
	27	0	7.15
	88	0	5.52

Day 27 represents data collected from Day 27 to 32.

Appendix 14

Individual Neurobehavioral Evaluation: Sensorimotor

32025165
Sex: Male

4.47x10^11 vg<br><br>Group 3	Day(s) Relative to Start Date	Sensorimotor<br>Touch Resp/ Tactile Refl	Sensorimotor<br>Thermal Response (sec)
3008	-1	0	10.28
	27	-1	9.81
	88	0	10.45
3009	-1	0	6.38
	27	0	4.30
	88	0	3.83
3010	-1	0	8.19
	27	0	7.46
	88	0	9.22

Day 27 represents data collected from Day 27 to 32.

Appendix 14

Individual Neurobehavioral Evaluation: Sensorimotor

32025165
Sex: Male

1.49x10^12 vg<br>Group 4	Day(s) Relative to Start Date	Sensorimotor	
		Touch Resp/ Tactile Refl	Thermal Response (sec)
4001	-1	0	19.21
	27	0	6.41
4002	-1	0	13.72
	27	0	8.71
4003	-1	0	23.46
	27	0	4.62
4004	-1	0	8.57
	27	0	8.16
4005	-1	0	11.89
	27	0	4.00
4006	-1	0	6.44
	27	-1	13.19
	88	0	5.90
4007	-1	0	8.31
	27	0	12.31
	88	0	5.53
4008	-1	0	10.21
	27	0	9.53
	88	0	12.08

Day 27 represents data collected from Day 27 to 32.

DRAFT, NOT FOR SUBMISSION

Appendix 14

Individual Neurobehavioral Evaluation: Sensorimotor

32025165
Sex: Male

1.49x10^12 vg Group 4	Day(s) Relative to Start Date	Sensorimotor Touch Resp/ Tactile Refl	Sensorimotor Thermal Response (sec)
4009	-1	0	6.84
	27	0	7.12
	88	0	12.39
4010	-1	0	11.63
	27	0	11.09
	88	0	5.45
4011	-1	0	9.03
	27	-1	10.03
	88	0	2.68
4012	-1	0	6.20
	27	0	9.59
	88	1	4.45
4013	-1	0	14.66
	27	-1	8.69
	88	0	7.20
4014	-1	0	4.78
	27	0	8.10
	88	0	15.30
4015	-1	0	4.72
	27	0	15.31
	88	0	8.37

Day 27 represents data collected from Day 27 to 32.

Appendix 14

Individual Neurobehavioral Evaluation: Sensorimotor

32025165
Sex: Female

0 vg		Sensorimotor	
Group 1	Day(s) Relative to Start Date	Touch Resp/ Tactile Refl	Thermal Response (sec)
1501	-1	0	10.91
	27	0	2.60
1502	-1	0	28.85
	27	0	4.50
1503	-1	0	10.76
	27	0	4.82
1504	-1	0	6.82
	27	0	8.25
1505	-1	0	10.81
	27	0	4.18
1506	-1	0	6.01
	27	0	19.78
	89	0	6.14
1507	-1	0	5.62
	27	0	4.06
	89	0	5.49
1508	-1	0	4.21
	27	0	15.71
	89	0	6.84

Day 27 represents data collected from Day 27 to 32.

Appendix 14

Individual Neurobehavioral Evaluation: Sensorimotor

32025165

Sex: Female

0 vg		Sensorimotor	
Group 1	Day(s) Relative to Start Date	Touch Resp/ Tactile Refl	Thermal Response (sec)
1509	-1	0	7.46
	27	0	20.13
	89	0	13.19
1510	-1	0	4.51
	27	0	15.65
	89	0	9.95

Day 27 represents data collected from Day 27 to 32.

Appendix 14

Individual Neurobehavioral Evaluation: Sensorimotor

32025165

Sex: Female

1.49x10^11 vg<br>Group 2	Day(s) Relative to Start Date	Sensorimotor<br>Touch Resp/ Tactile Refl	Sensorimotor<br>Thermal Response (sec)
2501	-1	0	7.76
	27	0	6.09
	89	0	5.12
2502	-1	0	14.20
	27	0	13.78
	89	0	17.13
2503	-1	0	5.34
	27	0	4.15
	89	-1	7.19
2504	-1	0	4.82
	27	0	13.23
	89	0	4.75
2505	-1	0	6.09
	27	0	7.90
	89	0	7.53
2506	-1	0	6.63
	27	0	11.81
	89	0	10.65
2507	-1	0	6.71
	27	0	19.40
	89	0	5.82

Day 27 represents data collected from Day 27 to 32.

Appendix 14

Individual Neurobehavioral Evaluation: Sensorimotor

32025165

Sex: Female

1.49x10^11 vg Group 2	Day(s) Relative to Start Date	Sensorimotor Touch Resp/ Tactile Refl	Sensorimotor Thermal Response (sec)
2508	-1	0	5.15
	27	0	10.18
	89	0	6.52
2509	-1	0	5.62
	27	0	5.00
	89	0	9.97
2510	-1	0	4.44
	27	0	12.06
	89	0	9.59

Day 27 represents data collected from Day 27 to 32.

Appendix 14

Individual Neurobehavioral Evaluation: Sensorimotor

32025165
Sex: Female

4.47x10^11 vg<br>Group 3	Day(s) Relative to Start Date	Sensorimotor	
		Touch Resp/ Tactile Refl	Thermal Response (sec)
3501	-1	0	5.32
	27	0	4.78
	89	0	5.95
3502	-1	0	5.50
	27	0	9.50
	89	0	5.33
3503	-1	0	6.20
	27	0	6.63
	89	0	9.39
3504	-1	0	4.78
	27	0	10.15
	89	0	5.08
3505	-1	0	8.25
	27	0	4.78
	89	0	12.58
3506	-1	0	6.13
	27	0	11.04
	89	0	13.98
3507	-1	0	7.96
	27	0	7.94
	89	0	9.46

Day 27 represents data collected from Day 27 to 32.

Appendix 14

Individual Neurobehavioral Evaluation: Sensorimotor

32025165

Sex: Female

4.47x10^11 vg<br>Group 3	Day(s) Relative to Start Date	Sensorimotor	
		Touch Resp/ Tactile Refl	Thermal Response (sec)
3508	-1	0	8.50
	27	0	17.43
	89	0	11.70
3509	-1	0	6.45
	27	0	10.45
	89	0	11.01
3510	-1	0	4.65
	27	0	9.56
	89	0	7.72

Day 27 represents data collected from Day 27 to 32.

Appendix 14

Individual Neurobehavioral Evaluation: Sensorimotor

32025165

Sex: Female

1.49x10^12 vg Group 4	Day(s) Relative to Start Date	Sensorimotor Touch Resp/ Tactile Refl	Sensorimotor Thermal Response (sec)
4501	-1	0	4.13
	27	0	8.06
4502	-1	0	6.89
	27	0	5.65
4503	-1	0	8.00
	27	0	4.15
4504	-1	0	8.39
	27	0	10.28
4505	-1	0	5.72
	27	0	8.97
4506	-1	0	6.71
	27	0	6.53
	89	0	9.52
4507	-1	0	3.77
	27	0	8.69
	89	0	27.38
4508	-1	0	7.52
	27	0	5.28
	89	0	4.75

Day 27 represents data collected from Day 27 to 32.

Appendix 14

Individual Neurobehavioral Evaluation: Sensorimotor

32025165

Sex: Female

1.49x10^12 vg Group 4	Day(s) Relative to Start Date	Sensorimotor Touch Resp/ Tactile Refl	Sensorimotor Thermal Response (sec)
4509	-1	0	6.09
	27	0	7.53
	89	0	18.14
4510	-1	0	7.65
	27	0	9.10
	89	0	16.70
4511	-1	0	6.40
	27	0	3.76
	89	0	6.37
4512	-1	0	6.19
	27	0	17.48
	89	0	10.40
4513	-1	0	5.77
	27	0	7.37
	89	0	5.33
4514	-1	0	7.75
	27	0	14.44
	89	0	6.39
4515	-1	0	4.47
	27	0	7.07
	89	0	10.59

Day 27 represents data collected from Day 27 to 32.

Appendix 15

Audited Draft Report

Study Phase: Clinical Pathology

Testing Facility Study No. 32025165

Sponsor Reference No. 32025165

GLP

TESTING FACILITY:
Charles River Laboratories
54943 North Main Street
Mattawan, MI 49071
United States

Appendix 15

TABLE OF CONTENTS

LIST OF TABLES 3
LIST OF APPENDICES 4
REPORT APPROVAL 5
1. RESPONSIBLE PERSONNEL 6
2. SUMMARY 7
3. MATERIALS AND METHODS 7
3.1. Group Assignments 7
3.2. Computerized Systems and Electronic Data Archive Locations 8
4. RETENTION AND DISPOSITION OF STUDY PHASE MATERIAL 8
5. RESULTS AND DISCUSSION 9
5.1. Hematology 9
5.2. Coagulation 9
5.3. Clinical Chemistry 9

Appendix 15

LIST OF TABLES

Table 1 Summary of Hematology Values 16
Table 2 Summary of Coagulation Values..38
Table 3 Summary of Clinical Chemistry Values..42

LIST OF APPENDICES

Appendix 1 Individual Hematology Values....67
Appendix 2 Individual Coagulation Values....99
Appendix 3 Individual Clinical Chemistry Values....107

REPORT APPROVAL

All electronic signatures appear at the end of the document upon finalization.

Appendix 15

1. RESPONSIBLE PERSONNEL

Individual Scientist, Clinical Pathologist	Danielle Gordon, DVM, MS, DACVP<br>Charles River Laboratories
Testing Facility Management	Scott Fountain, PhD<br>Charles River Laboratories

DRAFT, NOT FOR SUBMISSION

2. SUMMARY

This study was conducted for University of Massachusetts to determine the potential toxicity and biodistribution of the test article, scAAV9-CSA, for the treatment of Cockayne Syndrome, when given as a cerebrospinal (intrathecal [IT]) injection to rats once on Day 1, and to evaluate the potential reversibility of any findings following a 28-day and 90-day recovery period.

Ninety Sprague Dawley rats (5/sex/Groups 1 and 4 [Main Study] and 5 [Group 1] or 10 [Groups 2, 3, and 4]/sex/Group [Recovery Study]) were administered the vehicle control or scAAV9-CSA (test item) at 1.49 x 10^{11}, 4.47 x 10^{11}, or 1.49 x 10^{12} vg via intrathecal injection once on Day 1. Hematology, coagulation, and clinical chemistry parameters were evaluated prior to necropsy on Day 29 ± 1 (5/sex/Groups 1 and 4) or following a 90-day recovery period on Day 91 ± 2 (5 or 10/sex/group). 20 animals (5 males and 5 females in Groups 1 and 4) were euthanized on Day 29 ± 1 and remaining animals were terminated on Day 91 ± 2 following at 90-day recovery period.

There were no scAAV9-CSA-related alterations in clinical pathology parameters at any dose level at any time point.

3. MATERIALS AND METHODS

3.1. Group Assignments

Animals were assigned to the study as indicated in Text Table 1. Experimental procedures applicable to clinical pathology are summarized in Text Table 2.

Text Table 1
Group Assignments

Group No.	Dose Level (vg)	No. of Animals			
		Necropsy Day 29 (±1)		Necropsy Day 91 (±2)	
		Male	Female	Male	Female
1	0	5	5	5	5
2	1.49 x 10^{11}	-	-	10	10
3	4.47 x 10^{11}	-	-	10	10
4	1.49 x 10^{12}	5	5	10	10

- = Not applicable; vg = Vector genome.

Text Table 2
Clinical Pathology Evaluation

Group Nos.	Time Point	Hematology	Coagulation	Clinical Chemistry
All Animals	Prior to necropsy on Days 29 (±1) and 91 (±2)	X	X	X

X = Sample collected.

3.2. Computerized Systems and Electronic Data Archive Locations

Critical computerized systems used in the study phase and their respective electronic data archival locations are listed below. All computerized systems used in the conduct of this study have been validated; when a particular system has not satisfied all requirements, appropriate administrative and procedural controls were implemented to assure the quality and integrity of data.

Text Table 3
Critical Computerized Systems

System Name	System Description	Archive Location
AU5800™	Chemistry analyzer	Testing Facility
DocuSign™ eSignature	Collection of 21 CFR Part 11 compliant signature	Charles River Massachusetts Datacenter
eInfotree™	Excel Module for data collection with 21 CFR Part 11 compliance requirements, security, audit trail, and electronic signatures	Charles River Massachusetts Datacenter Testing Facility
ExyLIMS	A comprehensive laboratory information management system used to manage data, including but not limited to instrumentation, test materials, standards, and samples	Testing Facility
M-Files®	Document management, publishing, and collection of 21 CFR Part 11 compliant signatures	Charles River Massachusetts Datacenter
Provantis®	Toxicology, clinical pathology, pathology, test material receipt, and formulation data collection, reporting, and statistics system	Charles River Massachusetts Datacenter
Siemens Monitoring System	Environmental monitoring, alarming, and reporting applications	Testing Facility
Stago®	Coagulation analyzer	Testing Facility
XN®	Hematology analyzer	Testing Facility

4. RETENTION AND DISPOSITION OF STUDY PHASE MATERIAL

All study phase-specific physical raw data, documentation, and retained samples from this study phase are archived at the Testing Facility no later than the date of Final Report issue.

Electronic data and documentation generated by this study phase are archived. Data generated by critical computerized systems are archived as listed in the critical computerized systems list. Electronic data archived in a cloud environment are under control of the Charles River Laboratories Archivist.

Text Table 4
Sample Disposition

Clinical Pathology	Discarded

5. RESULTS AND DISCUSSION

For the purpose of this report, treated animals' values were compared to control animals' values. Fold change (x) in clinical pathology parameters was determined by comparing the scAAV9-CSA group mean to the respective control group mean unless otherwise noted.

5.1. Hematology

(Table 1 and Appendix 1)

There were no definitive scAAV9-CSA-related alterations in hematology parameters at any dose level at any time point.

On Day 29 ± 1, there were three males that had sporadic changes, including moderately higher neutrophils (Animal No. 4004), mildly higher lymphocytes (Animal No. 4005), or mildly higher eosinophils (Animal No. 4003). Given the sporadic nature of these changes, they were considered to be unlikely scAAV9-CSA related.

All other fluctuations among individual and mean hematology values were sporadic, consistent with biologic and procedure-related variation, and/or negligible in magnitude. Therefore, these fluctuations were considered to be unrelated to scAAV9-CSA administration.

5.2. Coagulation

(Table 2 and Appendix 2)

There were no definitive scAAV9-CSA-related alterations in coagulation parameters at any dose level at any time point.

On Day 29 ± 1, both sexes at 1.49×10^{12} vg had minimally prolonged activated partial thromboplastin time (APTT; up to 1.13x). Given the minimal magnitude of change and general overlap with control group values, alterations in APTT were considered to be of uncertain relationship to scAAV9-CSA administration.

All other fluctuations among individual and mean coagulation values were sporadic, consistent with biologic and procedure-related variation, and/or negligible in magnitude. Therefore, these fluctuations were considered to be unrelated to scAAV9-CSA administration.

5.3. Clinical Chemistry

(Table 3 and Appendix 3)

There were no definitive scAAV9-CSA-related alterations in clinical chemistry parameters at any dose level at any time point.

On Day 29 ± 1, one male (Animal No. 4003), had mildly to moderately higher aspartate aminotransferase (AST), alanine aminotransferase (ALT), and sorbitol dehydrogenase (SDH) activities. On Day 91 ± 2, in males at 1.49×10^{12} vg and females at $\geq 1.49 \times 10^{11}$ vg, there were 1-3 animals per group that had minimal to mild increases in AST, ALT, and SDH activities. In Animal No. 4009, these changes may have correlated with microscopic findings in the liver (Pathology Report). Overall, the alterations in AST, ALT, and SDH were of uncertain

Appendix 15

relationship to scAAV9-CSA administration given the sporadic nature of the change and due to the presence of a similar increase in one control group male on Day 91 ± 2 (Animal No. 1010).

On Day 91 ± 2, in males at ≥ 4.47 x 10^{11} vg and female at 1.49 x 10^{12} vg, there were minimal increases in phosphorus concentration. In absence of a comparable change on Day 29 ± 1, these changes were of uncertain relationship to scAAV9-CSA administration.

All other fluctuations among individual and mean clinical chemistry values were sporadic, consistent with biologic and procedure-related variation, and/or negligible in magnitude. Therefore, these fluctuations were considered to be unrelated to scAAV9-CSA administration.

DRAFT, NOT FOR SUBMISSION

Appendix 15

DATA EXPLANATION PAGE

Note: All abbreviations listed on these pages may not be applicable to this report.

Abbreviation	Description
% Diff	% Difference from Group 1
tCtrl	Times control (fold change)
1F, 2F, 3F, 4F	Group 1 Female, Group 2 Female, Group 3 Female, Group 4 Female
1M, 2M, 3M, 4M	Group 1 Male, Group 2 Male, Group 3 Male, Group 4 Male
N	Number of values included in analysis
M, F	Male, Female
TK	Toxicokinetic
<, >	Out of range
--	Not scheduled to be performed/dead
- or .	Not applicable
mlmig	mL/min/g

TIMESLOTS

Abbreviation	Description
ETRM	Early Termination
H	Hours postdose
M	Minutes postdose
P	Postdose
Pre	Predose
Pre1	Pretest 1
Pre2	Pretest 2
Unsc	Unscheduled bleed
Vet	Veterinary Aid

CLINICAL PATHOLOGY

Abbreviation	Description	Abbreviation	Description
ADQ	Adequate	NT	Not Taken
BDSM	Blood Smear Created	NVO	No Value Obtained
CLOT	Sample Clotted	OA	Omitted Activity
DEC	Decreased	OD	Optical Density
INC	Increased	QNS	Quantity Not Sufficient
LLOQ	Less than Lower Limit of Quantitation	RC	Result Comment
MDIFF	Manual Differential	SNR	Sample Not Received
NAF	No Abnormal Findings	TNR	Test Not Reported
NC	Not Calculable	UTD	Unable to Determine
NCD	No Clot Detected	UTDH	Unable to Determine due to Hemolysis
NHT	Non Hemolyzed Trace	UTDM	Unable to Determine due to no Microscopic Confirmation
NRR	No Reportable Result	UTDR	Unable to Determine due to Non-Reproducibility
NSR	No Sample Received	COMM	Comment

HEMATOLOGY

Abbreviation	Description	Abbreviation	Description
WBC	White Blood Cell Count	HYPO	Hypochromasia
NEUT	Neutrophils	IMM NEUT MORPH	Immature Neutrophils Morphology
LYMPH	Lymphocytes	KERA	Keratocyte
MONO	Monocytes	KURL	Kurloff Cell

Appendix 15

Abbreviation	Description	Abbreviation	Description
EOS	Eosinophils	LGPLT	Large Platelets
BASO	Basophils	LRE%	Low Absorption Retic Count Percent
LUC	Large Unstained Cells	LYMVAC	Vacuolated Lymphocytes
RBC	Red Blood Cell Count	MACRO	Macrocytes
HGB	Hemoglobin	mBNeut	Neutrophils Band, Manual Count
HCT	Hematocrit	MCVG	MCV Gated
MCV	Mean Corpuscular Volume	MCVR	MCV Reticulocytes
MCH	Mean Corpuscular Hemoglobin	MEGA	Megakaryocytes
MCHC	Mean Corpuscular Hemoglobin Concentration	MICRO	Microcytes
RDW	Red Blood Cell Distribution Width	mINeut	Neutrophils Immature, Manual Count
PLT	Platelet Count	mNeut	Neutrophils, Manual Count
RETIC	Reticulocytes	MPC	Mean Platelet Component
ACAN	Acanthocytes	mPEST	mPlatelet Estimate
AGGL	Agglutination	MPM	Mean Platelet Dry Mass
ANISO	Anisocytosis	MPV	Mean Platelet Volume
BASTP	Basophilic Stippling	MRT%	Med Absorption Retic Count Percent
CH	Corpuscular Hemoglobin Content	mWBC	White Blood Cell Count, Measured
CHCM	Corpuscular Hemoglobin Concentration Mean	mWEST	mWhite Blood Cell Estimate
CHDW	Corpuscular Hemoglobin Concentration Distribution Width	NEUT BAND MORPH	Neutrophils Band Form Morphology
cHGB	Cellular Hemoglobin	NRBL	Nucleated Red Blood Cells/100 Leukocytes
CHR	Reticulocyte Corpuscular Hemoglobin Content	UNSPCECE	Other Cell Type Percent – White Blood Differential
CODO	Codocytes	UNSPCE	Other Cell Type – White Blood Differential
CYTO BASO NEUT	Cytoplasmic Basophilia Neutrophil	OVAL	Ovalocytes
DACR	Dacryocytes	PAP BODIES	Pappenheimer Bodies
DEG CELL	Degenerated Cells	PCDW	Platelet Component Distribution Width
ECCE	Eccentrocytes	PCT	Platelet Hematocrit
ECHINO	Echinocytes	PDW	Platelet Distribution Width
GCHCM	Corpuscular Hemoglobin Concentration Mean Gated	PLASMOD	Plasmodium
HDW	Hemoglobin Concentration Distribution Width	PLT MORPH	Platelet Morphology
HEINZ	Heinz Bodies	POIK	Poikilocytosis
HJB	Howell Jolly Bodies	POLY	Polychromasia
HRT%	High Absorption Retic Count Percent	RBCL	Red Blood Cell Clumping
RBC MORPH	Red Blood Cell Morphology	SCHZ	Schistocytes
RBCNUCLE	Nucleated Red Blood Cells/100 Leukocytes	SMUDGE	Smudge Cells
RCHCM	Reticulocyte Corpuscular Hemoglobin Concentration Mean	SPHR	Spherocytes
RDWG	Red Blood Cell Distribution Width Gated (Wistar Rat)*	STOM	Stomatocytes
REACTIVE LYMPH	Reactive Lymphocytes	VAC NEUT	Vacuolated Neutrophils
ROUL	Rouleaux Formation	WBC MORPH	White Blood Cell Morphology
RTCRBC	Red Blood Cell Count Reticulocyte	WBCP	White Blood Cell Count Perox
TOXG	Toxic Granulation	VAC LYM	Vacuolated Lymphocytes

Appendix 15

MANUAL EVALUATION – BLOOD CELL MORPHOLOGY

Platelets:

Species	DEC	ADQ	INC
Dog	0 - 7	6 - 40	≥ 41
Monkey	0 - 13	11 - 40	≥ 41
Rat	0 - 33	26 - 100	≥ 100
Mouse	0 - 33	26 - 120	≥ 121
Rabbit	0 - 7	6 - 67	≥ 68
Pig	0 - 13	11 - 53	≥ 54
Guinea Pig	0 - 7	6 - 40	≥ 41
Cat	0 - 7	6 - 40	≥ 41
Cattle	0 - 7	6 - 53	≥ 54
Sheep	0 - 15	14 - 53	≥ 54

Platelet Morphology:

Observation	1+ (Minimal)	2+ (Mild)	3+ (Moderate)	4+ (Marked)
Platelet Clumps/slide	1-7 small clumps	8-15 small clumps or 1-3 large clumps	>15 small clumps or >3 large clumps	>10 large clumps
Large Platelets	1%-5%	6%-10%	11%-30%	>30%

WBC Morphology:

Observation	1+ (Minimal)	2+ (Mild)	3+ (Moderate)	4+ (Marked)
Immature neutrophils	1%-5%	6%-10%	11%- 30%	>30%
Immature unclassified cells	1%-5%	6% -10%	11%- 30%	>30%
Cytoplasmic vacuolation of lymphocytes	1%-5%	6%-10%	11%- 30%	>30%
Other	1%-5%	6% -10%	11%- 30%	>30%
Neutrophils band form	1%-5%	6% -10%	11% - 30%	>30%
Cytoplasmic vacuolation of neutrophils	1%-5%	6% -10%	11% - 30%	>30%
Döhle Bodies	1%-5%	6% -10%	11% - 30%	>30%
Cytoplasmic basophilia	1%-5%	6% -10%	11% - 30%	>30%

RBC Morphology:

Observation	1+ (Minimal)	2+ (Mild)	3+ (Moderate)	4+ (Marked)
Hypochromasia	3% - 5%	6% - 15%	16% - 30%	>30%
Spherocytes	1% - 10%	11% - 20%	21% - 50%	>50%
Keratocytes	1% - 5%	6% - 10%	11% - 20%	>20%
Heinz Bodies	1-3 cells	4-8 cells	9-20 cells	>20 cells
RBC Agglutination	2-4 clumps	5-8 clumps	9-12 clumps	>12 clumps
Eccentrocytes	1% - 5%	6% - 10%	11% - 20%	>20%
Other	1% - 5%	6% - 10%	11% - 20%	>20%
Anisocytosis	3% - 5%	6% - 15%	16% - 30%	>30%
Microcytes	3% - 5%	6% - 15%	16% - 30%	>30%
Macrocytes	3% - 5%	6% - 15%	16% - 30%	>30%
Polychromasia	3% - 5%	6% - 10%	11% - 20%	>20%
Poikilocytosis	1% - 5%	6% - 10%	11% - 20%	>20%

DRAFT, NOT FOR SUBMISSION

Appendix 15

Observation	1+ (Minimal)	2+ (Mild)	3+ (Moderate)	4+ (Marked)
Echinocytes	3% - 5%	6% - 15%	16% - 30%	>30%
Acanthocytes	1% - 3%	4% - 8 %	9% - 20%	>20%
Schistocytes	1% - 5%	6% - 10%	11% - 20%	>20%
Ovalocytes	1% - 5%	6% - 10%	11% - 20%	>20%
Dacrocytes	1% - 5%	6% - 10%	11% - 20%	>20%
Codocytes	1% - 5%	6% - 10%	11% - 20%	>20%
Stomatocytes	1% - 5%	6% - 10%	11% - 20%	>20%
Howell-Jolly Bodies	1-3 cells	4-8 cells	9-20 cells	>20 cells
Basophilic Stippling	1-3 cells	4-8 cells	9-20 cells	>20 cells
Rouleaux	2-4 stacks	5-8 stacks	9-12 stacks	>12 stacks
RBC Clumping	2-4 clumps	5-8 clumps	9-12 clumps	>12 clumps
Plasmodium Species	Present in at least 3 fields	Present in at least 4-6 fields	Present in at least 7-9 fields	Present in >10 fields

COAGULATION

Abbreviation	Description	Abbreviation	Description
PT	Prothrombin Time	SAMQ	Sample Quality Coagulation
APTT	Activated Partial Thromboplastin Time	N	Normal
FIB	Fibrinogen	H	Hemolyzed
AAPTT	Activated Partial Thromboplastin Time (Alternate)	+	Slight or Mild (pale/light red)
AT3	Antithrombin III	++	Moderate (red)
DDIM	D-Dimer	+++	Severe or Marked (dark red)
TT	Thrombin Time	I	Icterus
		L	Lipemia

CLINICAL CHEMISTRY

Abbreviation	Description	Abbreviation	Description
AST	Aspartate Aminotransferase	CRP	C-Reactive Protein
ALT	Alanine Aminotransferase	CSFGLU	CSF Glucose
ALP	Alkaline Phosphatase	CSFPRO	CSF Protein
GGT	Gamma Glutamyl Transferase	DBIL	Direct Bilirubin
TBIL	Total Bilirubin	FE	Iron
UREAN	Urea Nitrogen	FERR	Ferritin
CREAT	Creatinine	FPHGB	Plasma Free Hemoglobin
GLUC	Glucose	FRUC	Fructosamine
CHOL	Cholesterol	GLDH	Glutamate Dehydrogenase
TRIG	Triglycerides	GLYH	Glycosylated Hemoglobin
TPROT	Total Protein	HBGDL	Total Hemoglobin g/dL (HbA1c)
ALB	Albumin	HDL	High-density lipoprotein
GLOB	Globulin	IBIL	Indirect Bilirubin
A/G	Albumin/Globulin Ratio	IGA	Immunoglobulin A
CA	Calcium	IGG	Immunoglobulin G
PHOS	Phosphorus	IGM	Immunoglobulin M
NA	Sodium	LACT	Lactate
K	Potassium	LDH	Lactate Dehydrogenase
CL	Chloride	LDL	Low-density lipoprotein
A1C%	Glycosylated Hemoglobin Percent	LIPASE	Lipase
ALD2	Aldosterone (pg/mL, 2 decimal)	MG	Magnesium
AMM	Ammonia	NEFA	Non-Esterified Fatty Acids
AMYL	Amylase	OSMLTY	Osmolality
ANGAP	Anion Gap	PLIP	Phospholipids

Appendix 15

Abbreviation	Description	Abbreviation	Description
APOA	Apolipoprotein A-1	SDH	Sorbitol Dehydrogenase
APOB	Apolipoprotein B	TIBC	Total Iron Binding Capacity
BILEAC	Bile Acids	TRSMG	Transferrin (mg/dL)
CC3	Complement C3 (mg/dL)	U/C	Urea Nitrogen/Creatinine
CK	Creatine Kinase	UIBC	Unsaturated Iron Binding Capacity
CKMB	CK MB Isoenzyme	URIAC	Uric Acid
CO2	Bicarbonate	HEMO	Hemolytic

Hemolysis Indice:

Abbreviation	Description
N	Normal
H	Hemolyzed
+	Slight or Mild (pale/light red)
++	Moderate (red)
+++	Severe or Marked (dark red)

Lipemia Indice:

Abbreviation	Description
N	Normal
L	Lipemic
+	Slight or Mild (cloudy)
++	Moderate (turbid)
+++	Severe or Marked (opaque)

Icterus Indice:

Abbreviation	Description
N	Normal
I	Icterus
+	Slight or Mild (dark yellow)
++	Moderate (very dark yellow)
+++	Severe or Marked (dark yellow-green)

DRAFT, NOT FOR SUBMISSION

Appendix 15
Table 1

Summary of Hematology Values

32025165

Sex: Male<br>Day(s) Relative to Start Date			0 vg<br>Group 1	1.49x10^11 vg<br>Group 2	4.47x10^11 vg<br>Group 3	1.49x10^12 vg<br>Group 4
WBC Reporting (10^3/uL)	28	Mean	11.330	-	-	15.236
		SD	3.373	-	-	3.640
		N	5	-	-	5
		tCtrl	-	-	-	1.34
	91	Mean	11.166	12.905	12.760	12.168
		SD	6.349	3.168	3.299	2.866
		N	5	10	10	10
		tCtrl	-	1.16	1.14	1.09
NEUT Percent (%)	28	Mean	14.56	-	-	15.62
		SD	5.55	-	-	5.49
		N	5	-	-	5
		tCtrl	-	-	-	1.07
	91	Mean	16.70	9.52	10.62	9.98
		SD	5.49	3.52	2.85	2.76
		N	5	10	10	10
		tCtrl	-	0.57	0.64	0.60

Day 91 represents all day 91 (±2) collection days

DRAFT, NOT FOR SUBMISSION

Appendix 15
Table 1

Summary of Hematology Values

32025165

Sex: Male	Day(s) Relative to Start Date		0 vg<br>Group 1	1.49x10^11 vg<br>Group 2	4.47x10^11 vg<br>Group 3	1.49x10^12 vg<br>Group 4
LYMPH Percent (%)	28	Mean	81.76	-	-	79.28
		SD	5.51	-	-	6.68
		N	5	-	-	5
		tCtrl	-	-	-	0.97
	91	Mean	77.14	85.08	85.05	84.49
		SD	4.35	4.60	4.05	4.17
		N	5	10	10	10
		tCtrl	-	1.10	1.10	1.10
MONO Percent (%)	28	Mean	2.58	-	-	3.56
		SD	0.45	-	-	0.95
		N	5	-	-	5
		tCtrl	-	-	-	1.38
	91	Mean	4.58	4.19	3.35	4.31
		SD	2.63	1.55	1.44	1.61
		N	5	10	10	10
		tCtrl	-	0.91	0.73	0.94

Day 91 represents all day 91 (±2) collection days

DRAFT, NOT FOR SUBMISSION

Appendix 15
Table 1

Summary of Hematology Values

32025165

Sex: Male<br>Day(s) Relative to Start Date			0 vg<br>Group 1	1.49x10^11 vg<br>Group 2	4.47x10^11 vg<br>Group 3	1.49x10^12 vg<br>Group 4
EOS Percent (%)	28	Mean	0.68	-	-	1.06
		SD	0.20	-	-	0.88
		N	5	-	-	5
		tCtrl	-	-	-	1.56
	91	Mean	1.12	0.83	0.66	0.79
		SD	0.88	0.24	0.35	0.48
		N	5	10	10	10
		tCtrl	-	0.74	0.59	0.71
BASO Percent (%)	28	Mean	0.42	-	-	0.48
		SD	0.11	-	-	0.08
		N	5	-	-	5
		tCtrl	-	-	-	1.14
	91	Mean	0.46	0.38	0.32	0.43
		SD	0.50	0.11	0.06	0.17
		N	5	10	10	10
		tCtrl	-	0.83	0.70	0.93

Day 91 represents all day 91 (±2) collection days

DRAFT, NOT FOR SUBMISSION

Appendix 15
Table 1

Summary of Hematology Values

32025165

Sex: Male		Day(s) Relative to Start Date	0 vg Group 1	1.49x10^11 vg Group 2	4.47x10^11 vg Group 3	1.49x10^12 vg Group 4
NEUT (10^3/uL)	28	Mean	1.606	-	-	2.376
		SD	0.677	-	-	1.127
		N	5	-	-	5
		tCtrl	-	-	-	1.48
	91	Mean	1.832	1.170	1.308	1.223
		SD	1.363	0.362	0.352	0.420
		N	5	10	10	10
		tCtrl	-	0.64	0.71	0.67
LYMPH (10^3/uL)	28	Mean	9.310	-	-	12.088
		SD	3.114	-	-	3.202
		N	5	-	-	5
		tCtrl	-	-	-	1.30
	91	Mean	8.540	11.068	10.917	10.279
		SD	4.775	3.113	3.054	2.543
		N	5	10	10	10
		tCtrl	-	1.30	1.28	1.20

Day 91 represents all day 91 (±2) collection days

DRAFT, NOT FOR SUBMISSION

Appendix 15

Table 1

Summary of Hematology Values

32025165

Sex: Male<br>Day(s) Relative to Start Date			0 vg<br>Group 1	1.49x10^11 vg<br>Group 2	4.47x10^11 vg<br>Group 3	1.49x10^12 vg<br>Group 4
MONO (10^3/uL)	28	Mean	0.288	-	-	0.548
		SD	0.083	-	-	0.227
		N	5	-	-	5
		tCtrl	-	-	-	1.90
	91	Mean	0.618	0.515	0.411	0.519
		SD	0.581	0.172	0.148	0.198
		N	5	10	10	10
		tCtrl	-	0.83	0.67	0.84
EOS (10^3/uL)	28	Mean	0.074	-	-	0.148
		SD	0.030	-	-	0.109
		N	5	-	-	5
		tCtrl	-	-	-	2.00
	91	Mean	0.122	0.100	0.084	0.094
		SD	0.078	0.022	0.044	0.057
		N	5	10	10	10
		tCtrl	-	0.82	0.69	0.77

Day 91 represents all day 91 (±2) collection days

DRAFT, NOT FOR SUBMISSION

Appendix 15

Table 1

Summary of Hematology Values

32025165

Sex: Male		Day(s) Relative to Start Date	0 vg<br>Group 1	1.49x10^11 vg<br>Group 2	4.47x10^11 vg<br>Group 3	1.49x10^12 vg<br>Group 4
BASO (10^3/uL)	28	Mean	0.052	-	-	0.076
		SD	0.025	-	-	0.018
		N	5	-	-	5
		tCtrl	-	-	-	1.46
	91	Mean	0.054	0.052	0.040	0.053
		SD	0.043	0.021	0.012	0.021
		N	5	10	10	10
		tCtrl	-	0.96	0.74	0.98
RBC (10^6/uL)	28	Mean	8.574	-	-	8.692
		SD	0.509	-	-	0.564
		N	5	-	-	5
		tCtrl	-	-	-	1.01
	91	Mean	9.444	9.274	9.146	8.957
		SD	0.589	0.548	0.501	0.560
		N	5	10	10	10
		tCtrl	-	0.98	0.97	0.95

Day 91 represents all day 91 (±2) collection days

DRAFT, NOT FOR SUBMISSION

Appendix 15
Table 1

Summary of Hematology Values

32025165

Sex: Male		Day(s) Relative to Start Date	0 vg<br>Group 1	1.49x10^11 vg<br>Group 2	4.47x10^11 vg<br>Group 3	1.49x10^12 vg<br>Group 4
HGB (g/dL)	28	Mean	15.86	-	-	16.40
		SD	0.99	-	-	0.75
		N	5	-	-	5
		tCtrl	-	-	-	1.03
	91	Mean	16.24	16.12	15.90	15.24
		SD	1.17	1.04	0.90	0.88
		N	5	10	10	10
		tCtrl	-	0.99	0.98	0.94
HCT (%)	28	Mean	51.62	-	-	52.58
		SD	2.92	-	-	2.16
		N	5	-	-	5
		tCtrl	-	-	-	1.02
	91	Mean	50.56	50.99	50.05	47.83
		SD	3.18	3.55	2.02	3.07
		N	5	10	10	10
		tCtrl	-	1.01	0.99	0.95

Day 91 represents all day 91 (±2) collection days

DRAFT, NOT FOR SUBMISSION

Appendix 15
Table 1

Summary of Hematology Values

32025165

Sex: Male<br>Day(s) Relative to Start Date			0 vg<br>Group 1	1.49x10^11 vg<br>Group 2	4.47x10^11 vg<br>Group 3	1.49x10^12 vg<br>Group 4
MCV (fL)	28	Mean	60.22	-	-	60.58
		SD	2.02	-	-	1.56
		N	5	-	-	5
		tCtrl	-	-	-	1.01
	91	Mean	53.54	54.99	54.78	53.44
		SD	1.58	2.47	1.30	1.99
		N	5	10	10	10
		tCtrl	-	1.03	1.02	1.00
MCH (pg)	28	Mean	18.52	-	-	18.92
		SD	0.73	-	-	0.43
		N	5	-	-	5
		tCtrl	-	-	-	1.02
	91	Mean	17.20	17.39	17.39	17.02
		SD	0.60	0.65	0.57	0.59
		N	5	10	10	10
		tCtrl	-	1.01	1.01	0.99

Day 91 represents all day 91 (±2) collection days

DRAFT, NOT FOR SUBMISSION

Appendix 15

Table 1

Summary of Hematology Values

32025165

Sex: Male		Day(s) Relative to Start Date	0 vg Group 1	1.49x10^11 vg Group 2	4.47x10^11 vg Group 3	1.49x10^12 vg Group 4
MCHC (g/dL)	28	Mean	30.70	-	-	31.20
		SD	0.35	-	-	0.23
		N	5	-	-	5
		tCtrl	-	-	-	1.02
	91	Mean	32.12	31.62	31.75	31.86
		SD	1.26	0.52	0.76	0.44
		N	5	10	10	10
		tCtrl	-	0.98	0.99	0.99
RDW (%)	28	Mean	14.24	-	-	13.88
		SD	1.51	-	-	1.43
		N	5	-	-	5
		tCtrl	-	-	-	0.97
	91	Mean	17.44	17.07	16.78	17.09
		SD	1.03	0.51	0.82	0.75
		N	5	10	10	10
		tCtrl	-	0.98	0.96	0.98

Day 91 represents all day 91 (±2) collection days

DRAFT, NOT FOR SUBMISSION

Appendix 15
Table 1

Summary of Hematology Values

32025165

Sex: Male		Day(s) Relative to Start Date	0 vg<br>Group 1	1.49x10^11 vg<br>Group 2	4.47x10^11 vg<br>Group 3	1.49x10^12 vg<br>Group 4
PLT (10^3/uL)	28	Mean	1048.2	-	-	971.6
		SD	81.5	-	-	200.0
		N	5	-	-	5
		tCtrl	-	-	-	0.93
	91	Mean	965.2	1090.1	1199.8	1177.1
		SD	207.1	161.0	138.3	256.0
		N	5	10	10	10
		tCtrl	-	1.13	1.24	1.22
RETIC (10^9/L)	28	Mean	299.16	-	-	293.44
		SD	37.74	-	-	46.93
		N	5	-	-	5
		tCtrl	-	-	-	0.98
	91	Mean	234.66	247.38	234.70	254.60
		SD	34.81	45.88	26.40	33.43
		N	5	10	10	10
		tCtrl	-	1.05	1.00	1.08

Day 91 represents all day 91 (±2) collection days

DRAFT, NOT FOR SUBMISSION

Appendix 15

Table 1

Summary of Hematology Values

32025165

Sex: Male<br>Day(s) Relative to Start Date			0 vg<br>Group 1	1.49x10^11 vg<br>Group 2	4.47x10^11 vg<br>Group 3	1.49x10^12 vg<br>Group 4
RETIC Percent (%)	28	Mean	3.50	-	-	3.38
		SD	0.45	-	-	0.44
		N	5	-	-	5
		tCtrl	-	-	-	0.97
	91	Mean	2.48	2.68	2.58	2.86
		SD	0.41	0.56	0.29	0.39
		N	5	10	10	10
		tCtrl	-	1.08	1.04	1.15

Day 91 represents all day 91 (±2) collection days

DRAFT, NOT FOR SUBMISSION

Appendix 15

Table 1

Summary of Hematology Values

32025165

Sex: Female	Day(s) Relative to Start Date		0 vg Group 1	1.49x10^11 vg Group 2	4.47x10^11 vg Group 3	1.49x10^12 vg Group 4
WBC Reporting (10^3/uL)	29	Mean	8.188	-	-	8.124
		SD	3.113	-	-	4.811
		N	5	-	-	5
		tCtrl	-	-	-	0.99
	91	Mean	7.573	8.534	9.753	9.649
		SD	3.193	1.474	3.866	2.071
		N	4	8	10	10
		tCtrl	-	1.13	1.29	1.27
NEUT Percent (%)	29	Mean	12.60	-	-	13.76
		SD	3.95	-	-	6.89
		N	5	-	-	5
		tCtrl	-	-	-	1.09
	91	Mean	10.55	11.16	9.12	8.38
		SD	4.13	3.10	2.91	2.32
		N	4	8	10	10
		tCtrl	-	1.06	0.86	0.79

Day 91 represents all day 91 (±2) collection days

DRAFT, NOT FOR SUBMISSION

Appendix 15

Table 1

Summary of Hematology Values

32025165

Sex: Female	Day(s) Relative to Start Date		0 vg Group 1	1.49x10^11 vg Group 2	4.47x10^11 vg Group 3	1.49x10^12 vg Group 4
LYMPH Percent (%)	29	Mean	82.98	-	-	81.68
		SD	3.78	-	-	6.21
		N	5	-	-	5
		tCtrl	-	-	-	0.98
	91	Mean	84.10	84.29	86.41	86.16
		SD	4.88	4.15	3.63	3.60
		N	4	8	10	10
		tCtrl	-	1.00	1.03	1.02
MONO Percent (%)	29	Mean	2.94	-	-	3.12
		SD	0.78	-	-	1.00
		N	5	-	-	5
		tCtrl	-	-	-	1.06
	91	Mean	3.18	3.60	3.37	4.35
		SD	0.70	1.27	1.47	1.22
		N	4	8	10	10
		tCtrl	-	1.13	1.06	1.37

Day 91 represents all day 91 (±2) collection days

DRAFT, NOT FOR SUBMISSION

Appendix 15

Table 1

Summary of Hematology Values

32025165

Sex: Female		Day(s) Relative to Start Date	0 vg Group 1	1.49x10^11 vg Group 2	4.47x10^11 vg Group 3	1.49x10^12 vg Group 4
EOS Percent (%)	29	Mean	0.86	-	-	1.00
		SD	0.38	-	-	0.29
		N	5	-	-	5
		tCtrl	-	-	-	1.16
	91	Mean	1.73	0.65	0.71	0.73
		SD	2.05	0.33	0.23	0.25
		N	4	8	10	10
		tCtrl	-	0.38	0.41	0.42
BASO Percent (%)	29	Mean	0.62	-	-	0.44
		SD	0.30	-	-	0.34
		N	5	-	-	5
		tCtrl	-	-	-	0.71
	91	Mean	0.45	0.30	0.39	0.38
		SD	0.21	0.16	0.18	0.12
		N	4	8	10	10
		tCtrl	-	0.67	0.87	0.84

Day 91 represents all day 91 (±2) collection days

Appendix 15
Table 1

Summary of Hematology Values

32025165

Sex: Female<br>Day(s) Relative to Start Date			0 vg<br>Group 1	1.49x10^11 vg<br>Group 2	4.47x10^11 vg<br>Group 3	1.49x10^12 vg<br>Group 4
NEUT (10^3/uL)	29	Mean	0.974	-	-	0.866
		SD	0.243	-	-	0.344
		N	5	-	-	5
		tCtrl	-	-	-	0.89
	91	Mean	0.743	0.968	0.866	0.777
		SD	0.264	0.372	0.425	0.161
		N	4	8	10	10
		tCtrl	-	1.30	1.17	1.05
LYMPH (10^3/uL)	29	Mean	6.866	-	-	6.852
		SD	2.899	-	-	4.269
		N	5	-	-	5
		tCtrl	-	-	-	1.00
	91	Mean	6.458	7.178	8.484	8.356
		SD	3.062	1.180	3.558	2.009
		N	4	8	10	10
		tCtrl	-	1.11	1.31	1.29

Day 91 represents all day 91 (±2) collection days

DRAFT, NOT FOR SUBMISSION

Appendix 15

Table 1

Summary of Hematology Values

32025165

Sex: Female	Day(s) Relative to Start Date		0 vg Group 1	1.49x10^11 vg Group 2	4.47x10^11 vg Group 3	1.49x10^12 vg Group 4
MONO (10^3/uL)	29	Mean	0.234	-	-	0.270
		SD	0.079	-	-	0.195
		N	5	-	-	5
		tCtrl	-	-	-	1.15
	91	Mean	0.238	0.304	0.302	0.409
		SD	0.111	0.116	0.113	0.109
		N	4	8	10	10
		tCtrl	-	1.28	1.27	1.72
EOS (10^3/uL)	29	Mean	0.068	-	-	0.090
		SD	0.029	-	-	0.061
		N	5	-	-	5
		tCtrl	-	-	-	1.32
	91	Mean	0.103	0.058	0.067	0.071
		SD	0.088	0.029	0.023	0.025
		N	4	8	10	10
		tCtrl	-	0.56	0.65	0.69

Day 91 represents all day 91 (±2) collection days

DRAFT, NOT FOR SUBMISSION

Appendix 15
Table 1

Summary of Hematology Values

32025165

Sex: Female Day(s) Relative to Start Date			0 vg Group 1	1.49x10^11 vg Group 2	4.47x10^11 vg Group 3	1.49x10^12 vg Group 4
BASO (10^3/uL)	29	Mean	0.046	-	-	0.046
		SD	0.015	-	-	0.039
		N	5	-	-	5
		tCtrl	-	-	-	1.00
	91	Mean	0.033	0.028	0.034	0.036
		SD	0.013	0.015	0.013	0.012
		N	4	8	10	10
		tCtrl	-	0.85	1.05	1.11
RBC (10^6/uL)	29	Mean	8.322	-	-	8.082
		SD	0.199	-	-	0.258
		N	5	-	-	5
		tCtrl	-	-	-	0.97
	91	Mean	8.228	8.109	8.311	8.269
		SD	0.406	0.221	0.240	0.292
		N	4	8	10	10
		tCtrl	-	0.99	1.01	1.01

Day 91 represents all day 91 (±2) collection days

Appendix 15

Table 1

Summary of Hematology Values

32025165

Sex: Female<br>Day(s) Relative to Start Date			0 vg<br>Group 1	1.49x10^11 vg<br>Group 2	4.47x10^11 vg<br>Group 3	1.49x10^12 vg<br>Group 4
HGB (g/dL)	29	Mean	15.34	-	-	15.16
		SD	0.37	-	-	0.61
		N	5	-	-	5
		tCtrl	-	-	-	0.99
	91	Mean	15.35	14.95	15.39	15.33
		SD	0.72	0.58	0.52	0.43
		N	4	8	10	10
		tCtrl	-	0.97	1.00	1.00
HCT (%)	29	Mean	47.94	-	-	46.96
		SD	1.20	-	-	1.46
		N	5	-	-	5
		tCtrl	-	-	-	0.98
	91	Mean	47.70	46.68	48.19	47.68
		SD	2.54	2.00	1.44	1.27
		N	4	8	10	10
		tCtrl	-	0.98	1.01	1.00

Day 91 represents all day 91 (±2) collection days

DRAFT, NOT FOR SUBMISSION

Appendix 15
Table 1

Summary of Hematology Values

32025165

Sex: Female		Day(s) Relative to Start Date	0 vg Group 1	1.49x10^11 vg Group 2	4.47x10^11 vg Group 3	1.49x10^12 vg Group 4
MCV (fL)	29	Mean	57.62	-	-	58.12
		SD	1.19	-	-	0.78
		N	5	-	-	5
		tCtrl	-	-	-	1.01
	91	Mean	57.98	57.55	58.00	57.70
		SD	0.76	1.21	1.20	1.75
		N	4	8	10	10
		tCtrl	-	0.99	1.00	1.00
MCH (pg)	29	Mean	18.44	-	-	18.76
		SD	0.32	-	-	0.29
		N	5	-	-	5
		tCtrl	-	-	-	1.02
	91	Mean	18.65	18.44	18.52	18.55
		SD	0.33	0.47	0.39	0.53
		N	4	8	10	10
		tCtrl	-	0.99	0.99	0.99

Day 91 represents all day 91 (±2) collection days

DRAFT, NOT FOR SUBMISSION

Appendix 15
Table 1

Summary of Hematology Values

32025165

Sex: Female		Day(s) Relative to Start Date	0 vg<br>Group 1	1.49x10^11 vg<br>Group 2	4.47x10^11 vg<br>Group 3	1.49x10^12 vg<br>Group 4
MCHC (g/dL)	29	Mean	32.02	-	-	32.28
		SD	0.13	-	-	0.38
		N	5	-	-	5
		tCtrl	-	-	-	1.01
	91	Mean	32.18	32.04	31.95	32.14
		SD	0.36	0.57	0.36	0.34
		N	4	8	10	10
		tCtrl	-	1.00	0.99	1.00
RDW (%)	29	Mean	12.72	-	-	11.78
		SD	0.61	-	-	0.44
		N	5	-	-	5
		tCtrl	-	-	-	0.93
	91	Mean	13.20	13.28	13.56	13.32
		SD	0.91	0.51	0.68	0.70
		N	4	8	10	10
		tCtrl	-	1.01	1.03	1.01

Day 91 represents all day 91 (±2) collection days

DRAFT, NOT FOR SUBMISSION

Appendix 15

Table 1

Summary of Hematology Values

32025165

Sex: Female<br>Day(s) Relative to Start Date			0 vg<br>Group 1	1.49x10^11 vg<br>Group 2	4.47x10^11 vg<br>Group 3	1.49x10^12 vg<br>Group 4
PLT (10^3/uL)	29	Mean	1136.6	-	-	1109.8
		SD	103.7	-	-	97.2
		N	5	-	-	5
		tCtrl	-	-	-	0.98
	91	Mean	944.0	1054.9	989.7	1012.6
		SD	55.3	113.5	66.6	135.0
		N	4	8	10	10
		tCtrl	-	1.12	1.05	1.07
RETIC (10^9/L)	29	Mean	235.98	-	-	227.26
		SD	42.15	-	-	68.08
		N	5	-	-	5
		tCtrl	-	-	-	0.96
	91	Mean	209.93	187.51	191.32	197.71
		SD	38.96	16.57	32.26	25.64
		N	4	8	10	10
		tCtrl	-	0.89	0.91	0.94

Day 91 represents all day 91 (±2) collection days

DRAFT, NOT FOR SUBMISSION

Appendix 15

Table 1

Summary of Hematology Values

32025165

Sex: Female<br>Day(s) Relative to Start Date			0 vg<br>Group 1	1.49x10^11 vg<br>Group 2	4.47x10^11 vg<br>Group 3	1.49x10^12 vg<br>Group 4
RETIC Percent (%)	29	Mean	2.84	-	-	2.80
		SD	0.48	-	-	0.82
		N	5	-	-	5
		tCtrl	-	-	-	0.99
	91	Mean	2.55	2.30	2.31	2.40
		SD	0.39	0.24	0.42	0.37
		N	4	8	10	10
		tCtrl	-	0.90	0.91	0.94

Day 91 represents all day 91 (±2) collection days

DRAFT, NOT FOR SUBMISSION

Appendix 15

Table 2

Summary of Coagulation Values

32025165

Sex: Male<br>Day(s) Relative to Start Date			0 vg<br>Group 1	1.49x10^11 vg<br>Group 2	4.47x10^11 vg<br>Group 3	1.49x10^12 vg<br>Group 4
PT (sec)	28	Mean	16.15	-	-	16.18
		SD	0.69	-	-	0.49
		N	4	-	-	4
		tCtrl	-	-	-	1.00
	91	Mean	15.75	16.28	16.12	16.80
		SD	0.48	0.52	0.67	0.85
		N	4	10	10	10
		tCtrl	-	1.03	1.02	1.07
APTT (sec)	28	Mean	11.48	-	-	12.98
		SD	1.33	-	-	1.70
		N	4	-	-	4
		tCtrl	-	-	-	1.13
	91	Mean	15.18	13.82	12.64	13.07
		SD	1.57	1.35	1.89	1.60
		N	4	10	10	10
		tCtrl	-	0.91	0.83	0.86

Day 91 represents all day 91 (±2) collection days

DRAFT, NOT FOR SUBMISSION

Appendix 15

Table 2

Summary of Coagulation Values

32025165

Sex: Male<br>Day(s) Relative to Start Date			0 vg<br>Group 1	1.49x10^11 vg<br>Group 2	4.47x10^11 vg<br>Group 3	1.49x10^12 vg<br>Group 4
FIB (mg/dL)	28	Mean	336.3	-	-	351.8
		SD	28.5	-	-	28.1
		N	4	-	-	4
		tCtrl	-	-	-	1.05
	91	Mean	362.8	338.7	333.2	348.3
		SD	35.2	17.7	17.4	40.2
		N	4	10	10	10
		tCtrl	-	0.93	0.92	0.96

Day 91 represents all day 91 (±2) collection days

DRAFT, NOT FOR SUBMISSION

Appendix 15
Table 2

Summary of Coagulation Values

32025165

Sex: Female<br>Day(s) Relative to Start Date			0 vg<br>Group 1	1.49x10^11 vg<br>Group 2	4.47x10^11 vg<br>Group 3	1.49x10^12 vg<br>Group 4
PT (sec)	29	Mean	16.98	-	-	16.60
		SD	0.89	-	-	0.80
		N	5	-	-	5
		tCtrl	-	-	-	0.98
	91	Mean	17.00	16.90	16.23	16.13
		SD	0.30	0.89	0.59	0.70
		N	3	7	10	10
		tCtrl	-	0.99	0.95	0.95
APTT (sec)	29	Mean	13.00	-	-	14.36
		SD	3.01	-	-	0.95
		N	5	-	-	5
		tCtrl	-	-	-	1.10
	91	Mean	14.60	13.17	12.82	13.22
		SD	1.48	2.11	2.11	2.24
		N	3	7	10	10
		tCtrl	-	0.90	0.88	0.91

Day 91 represents all day 91 (±2) collection days

DRAFT, NOT FOR SUBMISSION

Appendix 15

Table 2

Summary of Coagulation Values

32025165

Sex: Female		Day(s) Relative to Start Date	0 vg<br>Group 1	1.49x10^11 vg<br>Group 2	4.47x10^11 vg<br>Group 3	1.49x10^12 vg<br>Group 4
FIB (mg/dL)	29	Mean	245.6	-	-	246.8
		SD	21.3	-	-	18.5
		N	5	-	-	5
		tCtrl	-	-	-	1.00
	91	Mean	217.3	249.3	225.5	208.1
		SD	10.1	29.5	25.2	23.1
		N	3	7	10	10
		tCtrl	-	1.15	1.04	0.96

Day 91 represents all day 91 (±2) collection days

DRAFT, NOT FOR SUBMISSION

Appendix 15

Table 3

Summary of Clinical Chemistry Values

32025165

Sex: Male<br>Day(s) Relative to Start Date			0 vg<br>Group 1	1.49x10^11 vg<br>Group 2	4.47x10^11 vg<br>Group 3	1.49x10^12 vg<br>Group 4
AST (U/L)	28	Mean	109.8	-	-	143.2
		SD	15.3	-	-	96.6
		N	5	-	-	5
		tCtrl	-	-	-	1.30
	91	Mean	136.4	88.0	87.8	106.7
		SD	137.5	23.3	34.8	54.7
		N	5	10	10	10
		tCtrl	-	0.65	0.64	0.78
ALT (U/L)	28	Mean	67.0	-	-	83.6
		SD	13.8	-	-	50.0
		N	5	-	-	5
		tCtrl	-	-	-	1.25
	91	Mean	79.4	59.9	55.5	56.0
		SD	63.7	21.9	22.2	30.0
		N	5	10	10	10
		tCtrl	-	0.75	0.70	0.71

Day 91 represents all day 91 (±2) collection days

DRAFT, NOT FOR SUBMISSION

Appendix 15
Table 3

Summary of Clinical Chemistry Values

32025165

Sex: Male<br>Day(s) Relative to Start Date			0 vg<br>Group 1	1.49x10^11 vg<br>Group 2	4.47x10^11 vg<br>Group 3	1.49x10^12 vg<br>Group 4
ALP (U/L)	28	Mean	277.8	-	-	224.8
		SD	56.3	-	-	40.3
		N	5	-	-	5
		tCtrl	-	-	-	0.81
	91	Mean	128.8	150.6	137.2	136.1
		SD	32.0	42.5	29.7	29.3
		N	5	10	10	10
		tCtrl	-	1.17	1.07	1.06
GGT (U/L)	28	Mean	1.0	-	-	1.0
		SD	0.0	-	-	0.0
		N	5	-	-	5
		tCtrl	-	-	-	1.00
	91	Mean	1.0	1.0	1.0	1.0
		SD	0.0	0.0	0.0	0.0
		N	5	10	10	10
		tCtrl	-	1.00	1.00	1.00

Day 91 represents all day 91 (±2) collection days

DRAFT, NOT FOR SUBMISSION

Appendix 15
Table 3

Summary of Clinical Chemistry Values

32025165

Sex: Male			0 vg	1.49x10^11 vg	4.47x10^11 vg	1.49x10^12 vg
Day(s) Relative to Start Date			Group 1	Group 2	Group 3	Group 4
TBIL (mg/dL)	28	Mean	0.134	-	-	0.136
		SD	0.011	-	-	0.015
		N	5	-	-	5
		tCtrl	-	-	-	1.01
	91	Mean	0.156	0.147	0.141	0.145
		SD	0.036	0.018	0.019	0.038
		N	5	10	10	10
		tCtrl	-	0.94	0.90	0.93
UREAN (mg/dL)	28	Mean	12.4	-	-	12.0
		SD	1.9	-	-	1.6
		N	5	-	-	5
		tCtrl	-	-	-	0.97
	91	Mean	14.4	12.5	13.4	12.2
		SD	5.0	1.1	1.1	1.6
		N	5	10	10	10
		tCtrl	-	0.87	0.93	0.85

Day 91 represents all day 91 (±2) collection days

DRAFT, NOT FOR SUBMISSION

Appendix 15
Table 3

Summary of Clinical Chemistry Values

32025165

Sex: Male			0 vg	1.49x10^11 vg	4.47x10^11 vg	1.49x10^12 vg
Day(s) Relative to Start Date			Group 1	Group 2	Group 3	Group 4
CREAT (mg/dL)	28	Mean	0.38	-	-	0.38
		SD	0.04	-	-	0.04
		N	5	-	-	5
		tCtrl	-	-	-	1.00
	91	Mean	0.42	0.39	0.38	0.34
		SD	0.11	0.09	0.08	0.05
		N	5	10	10	10
		tCtrl	-	0.93	0.90	0.81
GLUC (mg/dL)	28	Mean	377.6	-	-	445.4
		SD	121.5	-	-	102.7
		N	5	-	-	5
		tCtrl	-	-	-	1.18
	91	Mean	271.4	317.4	257.4	267.7
		SD	106.4	81.9	74.4	114.7
		N	5	10	10	10
		tCtrl	-	1.17	0.95	0.99

Day 91 represents all day 91 (±2) collection days

DRAFT, NOT FOR SUBMISSION

Appendix 15
Table 3

Summary of Clinical Chemistry Values

32025165

Sex: Male		Day(s) Relative to Start Date	0 vg Group 1	1.49x10^11 vg Group 2	4.47x10^11 vg Group 3	1.49x10^12 vg Group 4
CHOL (mg/dL)	28	Mean	99.6	-	-	95.8
		SD	13.3	-	-	16.8
		N	5	-	-	5
		tCtrl	-	-	-	0.96
	91	Mean	84.8	72.0	64.9	65.2
		SD	20.5	14.7	7.7	8.9
		N	5	10	10	10
		tCtrl	-	0.85	0.77	0.77
TRIG (mg/dL)	28	Mean	162.0	-	-	229.0
		SD	48.9	-	-	72.4
		N	5	-	-	5
		tCtrl	-	-	-	1.41
	91	Mean	206.4	229.8	223.3	167.1
		SD	128.3	56.7	85.3	86.4
		N	5	10	10	10
		tCtrl	-	1.11	1.08	0.81

Day 91 represents all day 91 (±2) collection days

DRAFT, NOT FOR SUBMISSION

Appendix 15
Table 3

Summary of Clinical Chemistry Values

32025165

Sex: Male / Day(s) Relative to Start Date			0 vg Group 1	1.49x10^11 vg Group 2	4.47x10^11 vg Group 3	1.49x10^12 vg Group 4
TPROT (g/dL)	28	Mean	6.98	-	-	7.10
		SD	0.48	-	-	0.19
		N	5	-	-	5
		tCtrl	-	-	-	1.02
	91	Mean	7.32	7.34	7.11	7.02
		SD	0.36	0.39	0.26	0.39
		N	5	10	10	10
		tCtrl	-	1.00	0.97	0.96
ALB (g/dL)	28	Mean	3.72	-	-	3.72
		SD	0.13	-	-	0.08
		N	5	-	-	5
		tCtrl	-	-	-	1.00
	91	Mean	3.62	3.66	3.62	3.45
		SD	0.19	0.23	0.18	0.20
		N	5	10	10	10
		tCtrl	-	1.01	1.00	0.95

Day 91 represents all day 91 (±2) collection days

DRAFT, NOT FOR SUBMISSION

Appendix 15

Table 3

Summary of Clinical Chemistry Values

32025165

Sex: Male		Day(s) Relative to Start Date	0 vg Group 1	1.49x10^11 vg Group 2	4.47x10^11 vg Group 3	1.49x10^12 vg Group 4
GLOB (g/dL)	28	Mean	3.26	-	-	3.38
		SD	0.36	-	-	0.13
		N	5	-	-	5
		tCtrl	-	-	-	1.04
	91	Mean	3.70	3.68	3.49	3.57
		SD	0.19	0.23	0.13	0.33
		N	5	10	10	10
		tCtrl	-	0.99	0.94	0.96
A/G (ratio)	28	Mean	1.16	-	-	1.12
		SD	0.05	-	-	0.04
		N	5	-	-	5
		tCtrl	-	-	-	0.97
	91	Mean	0.96	1.01	1.04	0.98
		SD	0.05	0.09	0.07	0.09
		N	5	10	10	10
		tCtrl	-	1.05	1.08	1.02

Day 91 represents all day 91 (±2) collection days

DRAFT, NOT FOR SUBMISSION

Appendix 15
Table 3

Summary of Clinical Chemistry Values

32025165

Sex: Male			0 vg	1.49x10^11 vg	4.47x10^11 vg	1.49x10^12 vg
Day(s) Relative to Start Date			Group 1	Group 2	Group 3	Group 4
CA (mg/dL)	28	Mean	13.26	-	-	14.12
		SD	0.50	-	-	0.73
		N	5	-	-	5
		tCtrl	-	-	-	1.06
	91	Mean	12.12	12.72	12.33	12.46
		SD	1.00	0.68	0.59	0.73
		N	5	10	10	10
		tCtrl	-	1.05	1.02	1.03
PHOS (mg/dL)	28	Mean	11.54	-	-	11.54
		SD	0.90	-	-	0.71
		N	5	-	-	5
		tCtrl	-	-	-	1.00
	91	Mean	7.88	9.29	10.25	10.09
		SD	1.29	0.75	1.22	0.95
		N	5	10	10	10
		tCtrl	-	1.18	1.30	1.28

Day 91 represents all day 91 (±2) collection days

DRAFT, NOT FOR SUBMISSION

Appendix 15
Table 3

Summary of Clinical Chemistry Values

32025165

Sex: Male		Day(s) Relative to Start Date	0 vg Group 1	1.49x10^11 vg Group 2	4.47x10^11 vg Group 3	1.49x10^12 vg Group 4
NA (mEq/L)	28	Mean	147.2	-	-	146.0
		SD	4.1	-	-	1.9
		N	5	-	-	5
		tCtrl	-	-	-	0.99
	91	Mean	144.2	143.8	144.4	144.9
		SD	2.2	2.1	2.3	1.5
		N	5	10	10	10
		tCtrl	-	1.00	1.00	1.00
K (mEq/L)	28	Mean	7.80	-	-	7.60
		SD	0.82	-	-	0.33
		N	5	-	-	5
		tCtrl	-	-	-	0.97
	91	Mean	6.94	7.65	8.18	7.15
		SD	1.04	1.09	1.05	1.37
		N	5	10	10	10
		tCtrl	-	1.10	1.18	1.03

Day 91 represents all day 91 (±2) collection days

DRAFT, NOT FOR SUBMISSION

Appendix 15

Table 3

Summary of Clinical Chemistry Values

32025165

Sex: Male		Day(s) Relative to Start Date	0 vg<br>Group 1	1.49x10^11 vg<br>Group 2	4.47x10^11 vg<br>Group 3	1.49x10^12 vg<br>Group 4
CL (mEq/L)	28	Mean	100.2	-	-	98.8
		SD	2.8	-	-	2.2
		N	5	-	-	5
		tCtrl	-	-	-	0.99
	91	Mean	98.8	99.1	99.5	99.4
		SD	3.3	1.1	2.5	1.5
		N	5	10	10	10
		tCtrl	-	1.00	1.01	1.01
SDH Alternate (U/L)	28	Mean	35.96	-	-	40.70
		SD	24.31	-	-	25.71
		N	5	-	-	5
		tCtrl	-	-	-	1.13
	91	Mean	38.16	20.88	16.77	23.63
		SD	57.93	20.31	12.72	33.15
		N	5	10	10	10
		tCtrl	-	0.55	0.44	0.62

Day 91 represents all day 91 (±2) collection days

DRAFT, NOT FOR SUBMISSION

Appendix 15
Table 3

Summary of Clinical Chemistry Values

32025165

Sex: Female			0 vg	1.49x10^11 vg	4.47x10^11 vg	1.49x10^12 vg
Day(s) Relative to Start Date			Group 1	Group 2	Group 3	Group 4
AST (U/L)	29	Mean	105.4	-	-	95.0
		SD	24.8	-	-	18.0
		N	5	-	-	5
		tCtrl	-	-	-	0.90
	91	Mean	126.4	155.7	147.7	116.9
		SD	40.7	86.9	67.6	47.8
		N	5	10	10	10
		tCtrl	-	1.23	1.17	0.92
ALT (U/L)	29	Mean	58.0	-	-	52.6
		SD	15.2	-	-	9.4
		N	5	-	-	5
		tCtrl	-	-	-	0.91
	91	Mean	56.6	80.0	69.5	64.5
		SD	13.6	26.6	29.0	25.0
		N	5	10	10	10
		tCtrl	-	1.41	1.23	1.14

Day 91 represents all day 91 (±2) collection days

DRAFT, NOT FOR SUBMISSION

Appendix 15
Table 3

Summary of Clinical Chemistry Values

32025165

Sex: Female			0 vg	1.49x10^11 vg	4.47x10^11 vg	1.49x10^12 vg
Day(s) Relative to Start Date			Group 1	Group 2	Group 3	Group 4
ALP (U/L)	29	Mean	165.8	-	-	151.6
		SD	53.0	-	-	23.8
		N	5	-	-	5
		tCtrl	-	-	-	0.91
	91	Mean	79.2	112.8	93.8	76.5
		SD	11.9	44.4	30.0	28.5
		N	5	10	10	10
		tCtrl	-	1.42	1.18	0.97
GGT (U/L)	29	Mean	1.0	-	-	1.0
		SD	0.0	-	-	0.0
		N	5	-	-	5
		tCtrl	-	-	-	1.00
	91	Mean	1.0	1.0	1.0	1.0
		SD	0.0	0.0	0.0	0.0
		N	5	10	10	10
		tCtrl	-	1.00	1.00	1.00

Day 91 represents all day 91 (±2) collection days

DRAFT, NOT FOR SUBMISSION

Appendix 15

Table 3

Summary of Clinical Chemistry Values

32025165

Sex: Female<br>Day(s) Relative to Start Date			0 vg<br>Group 1	1.49x10^11 vg<br>Group 2	4.47x10^11 vg<br>Group 3	1.49x10^12 vg<br>Group 4
TBIL (mg/dL)	29	Mean	0.132	-	-	0.126
		SD	0.013	-	-	0.013
		N	5	-	-	5
		tCtrl	-	-	-	0.95
	91	Mean	0.156	0.146	0.144	0.149
		SD	0.009	0.022	0.018	0.030
		N	5	10	10	10
		tCtrl	-	0.94	0.92	0.96
UREAN (mg/dL)	29	Mean	13.8	-	-	12.8
		SD	1.3	-	-	1.1
		N	5	-	-	5
		tCtrl	-	-	-	0.93
	91	Mean	14.4	14.7	12.6	12.5
		SD	1.1	3.1	2.1	2.0
		N	5	10	10	10
		tCtrl	-	1.02	0.88	0.87

Day 91 represents all day 91 (±2) collection days

DRAFT, NOT FOR SUBMISSION

Appendix 15

Table 3

Summary of Clinical Chemistry Values

32025165

Sex: Female<br>Day(s) Relative to Start Date			0 vg<br>Group 1	1.49x10^11 vg<br>Group 2	4.47x10^11 vg<br>Group 3	1.49x10^12 vg<br>Group 4
CREAT (mg/dL)	29	Mean	0.38	-	-	0.32
		SD	0.04	-	-	0.04
		N	5	-	-	5
		tCtrl	-	-	-	0.84
	91	Mean	0.38	0.38	0.41	0.38
		SD	0.04	0.04	0.09	0.06
		N	5	10	10	10
		tCtrl	-	1.00	1.08	1.00
GLUC (mg/dL)	29	Mean	277.4	-	-	303.2
		SD	30.5	-	-	74.4
		N	5	-	-	5
		tCtrl	-	-	-	1.09
	91	Mean	231.8	277.1	270.1	276.4
		SD	54.1	49.1	75.9	64.9
		N	5	10	10	10
		tCtrl	-	1.20	1.17	1.19

Day 91 represents all day 91 (±2) collection days

DRAFT, NOT FOR SUBMISSION

Appendix 15
Table 3

Summary of Clinical Chemistry Values

32025165

Sex: Female		Day(s) Relative to Start Date	0 vg Group 1	1.49x10^11 vg Group 2	4.47x10^11 vg Group 3	1.49x10^12 vg Group 4
CHOL (mg/dL)	29	Mean	78.8	-	-	95.2
		SD	21.2	-	-	14.0
		N	5	-	-	5
		tCtrl	-	-	-	1.21
	91	Mean	66.6	68.0	73.1	76.2
		SD	13.0	16.0	17.1	9.7
		N	5	10	10	10
		tCtrl	-	1.02	1.10	1.14
TRIG (mg/dL)	29	Mean	81.2	-	-	107.0
		SD	26.2	-	-	59.4
		N	5	-	-	5
		tCtrl	-	-	-	1.32
	91	Mean	136.2	160.3	145.8	165.3
		SD	59.9	66.0	78.3	70.7
		N	5	10	10	10
		tCtrl	-	1.18	1.07	1.21

Day 91 represents all day 91 (±2) collection days

DRAFT, NOT FOR SUBMISSION

Appendix 15

Table 3

Summary of Clinical Chemistry Values

32025165

Sex: Female<br>Day(s) Relative to Start Date			0 vg<br>Group 1	1.49x10^11 vg<br>Group 2	4.47x10^11 vg<br>Group 3	1.49x10^12 vg<br>Group 4
TPROT (g/dL)	29	Mean	7.74	-	-	7.60
		SD	0.30	-	-	0.42
		N	5	-	-	5
		tCtrl	-	-	-	0.98
	91	Mean	7.84	8.05	8.11	8.15
		SD	0.50	0.40	0.65	0.36
		N	5	10	10	10
		tCtrl	-	1.03	1.03	1.04
ALB (g/dL)	29	Mean	4.32	-	-	4.14
		SD	0.16	-	-	0.30
		N	5	-	-	5
		tCtrl	-	-	-	0.96
	91	Mean	4.58	4.57	4.47	4.55
		SD	0.22	0.23	0.32	0.17
		N	5	10	10	10
		tCtrl	-	1.00	0.98	0.99

Day 91 represents all day 91 (±2) collection days

DRAFT, NOT FOR SUBMISSION

Appendix 15
Table 3

Summary of Clinical Chemistry Values

32025165

Sex: Female		Day(s) Relative to Start Date	0 vg Group 1	1.49x10^11 vg Group 2	4.47x10^11 vg Group 3	1.49x10^12 vg Group 4
GLOB (g/dL)	29	Mean	3.42	-	-	3.46
		SD	0.19	-	-	0.21
		N	5	-	-	5
		tCtrl	-	-	-	1.01
	91	Mean	3.26	3.48	3.64	3.60
		SD	0.37	0.23	0.42	0.26
		N	5	10	10	10
		tCtrl	-	1.07	1.12	1.10
A/G (ratio)	29	Mean	1.28	-	-	1.20
		SD	0.08	-	-	0.12
		N	5	-	-	5
		tCtrl	-	-	-	0.94
	91	Mean	1.42	1.33	1.25	1.26
		SD	0.16	0.09	0.14	0.07
		N	5	10	10	10
		tCtrl	-	0.94	0.88	0.89

Day 91 represents all day 91 (±2) collection days

DRAFT, NOT FOR SUBMISSION

Appendix 15
Table 3

Summary of Clinical Chemistry Values

32025165

Sex: Female	Day(s) Relative to Start Date		0 vg Group 1	1.49x10^11 vg Group 2	4.47x10^11 vg Group 3	1.49x10^12 vg Group 4
CA (mg/dL)	29	Mean	12.96	-	-	13.38
		SD	0.44	-	-	0.44
		N	5	-	-	5
		tCtrl	-	-	-	1.03
	91	Mean	12.62	12.95	13.40	13.51
		SD	0.93	0.30	0.76	0.55
		N	5	10	10	10
		tCtrl	-	1.03	1.06	1.07
PHOS (mg/dL)	29	Mean	10.58	-	-	11.04
		SD	1.59	-	-	1.64
		N	5	-	-	5
		tCtrl	-	-	-	1.04
	91	Mean	7.54	7.33	8.55	9.25
		SD	0.81	1.13	1.03	1.06
		N	5	10	10	10
		tCtrl	-	0.97	1.13	1.23

Day 91 represents all day 91 (±2) collection days

DRAFT, NOT FOR SUBMISSION

Appendix 15
Table 3

Summary of Clinical Chemistry Values

32025165

Sex: Female		Day(s) Relative to Start Date	0 vg Group 1	1.49x10^11 vg Group 2	4.47x10^11 vg Group 3	1.49x10^12 vg Group 4
NA (mEq/L)	29	Mean	146.8	-	-	145.2
		SD	2.4	-	-	1.3
		N	5	-	-	5
		tCtrl	-	-	-	0.99
	91	Mean	145.2	143.4	144.5	143.7
		SD	0.8	1.1	1.4	2.5
		N	5	10	10	10
		tCtrl	-	0.99	1.00	0.99
K (mEq/L)	29	Mean	7.72	-	-	7.56
		SD	1.01	-	-	1.44
		N	5	-	-	5
		tCtrl	-	-	-	0.98
	91	Mean	7.80	7.79	7.64	7.69
		SD	0.36	0.92	0.64	0.87
		N	5	10	10	10
		tCtrl	-	1.00	0.98	0.99

Day 91 represents all day 91 (±2) collection days

DRAFT, NOT FOR SUBMISSION

Appendix 15
Table 3

Summary of Clinical Chemistry Values

32025165

Sex: Female			0 vg	1.49x10^11 vg	4.47x10^11 vg	1.49x10^12 vg
Day(s) Relative to Start Date			Group 1	Group 2	Group 3	Group 4
CL (mEq/L)	29	Mean	102.0	-	-	100.8
		SD	1.4	-	-	1.1
		N	5	-	-	5
		tCtrl	-	-	-	0.99
	91	Mean	101.8	101.2	100.0	99.6
		SD	1.8	1.8	1.6	1.9
		N	5	10	10	10
		tCtrl	-	0.99	0.98	0.98
SDH Alternate (U/L)	29	Mean	14.68	-	-	13.86
		SD	5.62	-	-	7.08
		N	5	-	-	5
		tCtrl	-	-	-	0.94
	91	Mean	23.04	34.63	32.92	28.53
		SD	18.90	24.71	21.53	24.00
		N	5	10	10	10
		tCtrl	-	1.50	1.43	1.24

Day 91 represents all day 91 (±2) collection days

DRAFT, NOT FOR SUBMISSION

Appendix 15

DATA EXPLANATION PAGE

Note: All abbreviations listed on these pages may not be applicable to this report.

Abbreviation	Description
% Diff	% Difference from Group 1
tCtrl	Times control (fold change)
1F, 2F, 3F, 4F	Group 1 Female, Group 2 Female, Group 3 Female, Group 4 Female
1M, 2M, 3M, 4M	Group 1 Male, Group 2 Male, Group 3 Male, Group 4 Male
N	Number of values included in analysis
M, F	Male, Female
TK	Toxicokinetic
<, >	Out of range
--	Not scheduled to be performed/dead
- or .	Not applicable
mlmig	mL/min/g

TIMESLOTS

Abbreviation	Description
ETRM	Early Termination
H	Hours postdose
M	Minutes postdose
P	Postdose
Pre	Predose
Pre1	Pretest 1
Pre2	Pretest 2
Unsc	Unscheduled bleed
Vet	Veterinary Aid

CLINICAL PATHOLOGY

Abbreviation	Description	Abbreviation	Description
ADQ	Adequate	NT	Not Taken
BDSM	Blood Smear Created	NVO	No Value Obtained
CLOT	Sample Clotted	OA	Omitted Activity
DEC	Decreased	OD	Optical Density
INC	Increased	QNS	Quantity Not Sufficient
LLOQ	Less than Lower Limit of Quantitation	RC	Result Comment
MDIFF	Manual Differential	SNR	Sample Not Received
NAF	No Abnormal Findings	TNR	Test Not Reported
NC	Not Calculable	UTD	Unable to Determine
NCD	No Clot Detected	UTDH	Unable to Determine due to Hemolysis
NHT	Non Hemolyzed Trace	UTDM	Unable to Determine due to no Microscopic Confirmation
NRR	No Reportable Result	UTDR	Unable to Determine due to Non-Reproducibility
NSR	No Sample Received	COMM	Comment

HEMATOLOGY

Abbreviation	Description	Abbreviation	Description
WBC	White Blood Cell Count	HYPO	Hypochromasia
NEUT	Neutrophils	IMM NEUT MORPH	Immature Neutrophils Morphology
LYMPH	Lymphocytes	KERA	Keratocyte
MONO	Monocytes	KURL	Kurloff Cell

Appendix 15

Abbreviation	Description	Abbreviation	Description
EOS	Eosinophils	LGPLT	Large Platelets
BASO	Basophils	LRE%	Low Absorption Retic Count Percent
LUC	Large Unstained Cells	LYMVAC	Vacuolated Lymphocytes
RBC	Red Blood Cell Count	MACRO	Macrocytes
HGB	Hemoglobin	mBNeut	Neutrophils Band, Manual Count
HCT	Hematocrit	MCVG	MCV Gated
MCV	Mean Corpuscular Volume	MCVR	MCV Reticulocytes
MCH	Mean Corpuscular Hemoglobin	MEGA	Megakaryocytes
MCHC	Mean Corpuscular Hemoglobin Concentration	MICRO	Microcytes
RDW	Red Blood Cell Distribution Width	mINeut	Neutrophils Immature, Manual Count
PLT	Platelet Count	mNeut	Neutrophils, Manual Count
RETIC	Reticulocytes	MPC	Mean Platelet Component
ACAN	Acanthocytes	mPEST	mPlatelet Estimate
AGGL	Agglutination	MPM	Mean Platelet Dry Mass
ANISO	Anisocytosis	MPV	Mean Platelet Volume
BASTP	Basophilic Stippling	MRT%	Med Absorption Retic Count Percent
CH	Corpuscular Hemoglobin Content	mWBC	White Blood Cell Count, Measured
CHCM	Corpuscular Hemoglobin Concentration Mean	mWEST	mWhite Blood Cell Estimate
CHDW	Corpuscular Hemoglobin Concentration Distribution Width	NEUT BAND MORPH	Neutrophils Band Form Morphology
cHGB	Cellular Hemoglobin	NRBL	Nucleated Red Blood Cells/100 Leukocytes
CHR	Reticulocyte Corpuscular Hemoglobin Content	UNSPCECE	Other Cell Type Percent – White Blood Differential
CODO	Codocytes	UNSPCE	Other Cell Type – White Blood Differential
CYTO BASO NEUT	Cytoplasmic Basophilia Neutrophil	OVAL	Ovalocytes
DACR	Dacryocytes	PAP BODIES	Pappenheimer Bodies
DEG CELL	Degenerated Cells	PCDW	Platelet Component Distribution Width
ECCE	Eccentrocytes	PCT	Platelet Hematocrit
ECHINO	Echinocytes	PDW	Platelet Distribution Width
GCHCM	Corpuscular Hemoglobin Concentration Mean Gated	PLASMOD	Plasmodium
HDW	Hemoglobin Concentration Distribution Width	PLT MORPH	Platelet Morphology
HEINZ	Heinz Bodies	POIK	Poikilocytosis
HJB	Howell Jolly Bodies	POLY	Polychromasia
HRT%	High Absorption Retic Count Percent	RBCL	Red Blood Cell Clumping
RBC MORPH	Red Blood Cell Morphology	SCHZ	Schistocytes
RBCNUCLE	Nucleated Red Blood Cells/100 Leukocytes	SMUDGE	Smudge Cells
RCHCM	Reticulocyte Corpuscular Hemoglobin Concentration Mean	SPHR	Spherocytes
RDWG	Red Blood Cell Distribution Width Gated (Wistar Rat)*	STOM	Stomatocytes
REACTIVE LYMPH	Reactive Lymphocytes	VAC NEUT	Vacuolated Neutrophils
ROUL	Rouleaux Formation	WBC MORPH	White Blood Cell Morphology
RTCRBC	Red Blood Cell Count Reticulocyte	WBCP	White Blood Cell Count Perox
TOXG	Toxic Granulation	VAC LYM	Vacuolated Lymphocytes

Appendix 15

MANUAL EVALUATION – BLOOD CELL MORPHOLOGY

Platelets:

Species	DEC	ADQ	INC
Dog	0 - 7	6 - 40	≥ 41
Monkey	0 - 13	11 - 40	≥ 41
Rat	0 - 33	26 - 100	≥ 100
Mouse	0 - 33	26 - 120	≥ 121
Rabbit	0 - 7	6 - 67	≥ 68
Pig	0 - 13	11 - 53	≥ 54
Guinea Pig	0 - 7	6 - 40	≥ 41
Cat	0 - 7	6 - 40	≥ 41
Cattle	0 - 7	6 - 53	≥ 54
Sheep	0 - 15	14 - 53	≥ 54

Platelet Morphology:

Observation	1+ (Minimal)	2+ (Mild)	3+ (Moderate)	4+ (Marked)
Platelet Clumps/slide	1-7 small clumps	8-15 small clumps or 1-3 large clumps	>15 small clumps or >3 large clumps	>10 large clumps
Large Platelets	1%-5%	6%-10%	11%-30%	>30%

WBC Morphology:

Observation	1+ (Minimal)	2+ (Mild)	3+ (Moderate)	4+ (Marked)
Immature neutrophils	1%-5%	6%-10%	11%- 30%	>30%
Immature unclassified cells	1%-5%	6% -10%	11%- 30%	>30%
Cytoplasmic vacuolation of lymphocytes	1%-5%	6%-10%	11%- 30%	>30%
Other	1%-5%	6% -10%	11%- 30%	>30%
Neutrophils band form	1%-5%	6% -10%	11% - 30%	>30%
Cytoplasmic vacuolation of neutrophils	1%-5%	6% -10%	11% - 30%	>30%
Döhle Bodies	1%-5%	6% -10%	11% - 30%	>30%
Cytoplasmic basophilia	1%-5%	6% -10%	11% - 30%	>30%

RBC Morphology:

Observation	1+ (Minimal)	2+ (Mild)	3+ (Moderate)	4+ (Marked)
Hypochromasia	3% - 5%	6% - 15%	16% - 30%	>30%
Spherocytes	1% - 10%	11% - 20%	21% - 50%	>50%
Keratocytes	1% - 5%	6% - 10%	11% - 20%	>20%
Heinz Bodies	1-3 cells	4-8 cells	9-20 cells	>20 cells
RBC Agglutination	2-4 clumps	5-8 clumps	9-12 clumps	>12 clumps
Eccentrocytes	1% - 5%	6% - 10%	11% - 20%	>20%
Other	1% - 5%	6% - 10%	11% - 20%	>20%
Anisocytosis	3% - 5%	6% - 15%	16% - 30%	>30%
Microcytes	3% - 5%	6% - 15%	16% - 30%	>30%
Macrocytes	3% - 5%	6% - 15%	16% - 30%	>30%
Polychromasia	3% - 5%	6% - 10%	11% - 20%	>20%
Poikilocytosis	1% - 5%	6% - 10%	11% - 20%	>20%

Appendix 15

Observation	1+ (Minimal)	2+ (Mild)	3+ (Moderate)	4+ (Marked)
Echinocytes	3% - 5%	6% - 15%	16% - 30%	>30%
Acanthocytes	1% - 3%	4% - 8 %	9% - 20%	>20%
Schistocytes	1% - 5%	6% - 10%	11% - 20%	>20%
Ovalocytes	1% - 5%	6% - 10%	11% - 20%	>20%
Dacrocytes	1% - 5%	6% - 10%	11% - 20%	>20%
Codocytes	1% - 5%	6% - 10%	11% - 20%	>20%
Stomatocytes	1% - 5%	6% - 10%	11% - 20%	>20%
Howell-Jolly Bodies	1-3 cells	4-8 cells	9-20 cells	>20 cells
Basophilic Stippling	1-3 cells	4-8 cells	9-20 cells	>20 cells
Rouleaux	2-4 stacks	5-8 stacks	9-12 stacks	>12 stacks
RBC Clumping	2-4 clumps	5-8 clumps	9-12 clumps	>12 clumps
Plasmodium Species	Present in at least 3 fields	Present in at least 4-6 fields	Present in at least 7-9 fields	Present in >10 fields

COAGULATION

Abbreviation	Description	Abbreviation	Description
PT	Prothrombin Time	SAMQ	Sample Quality Coagulation
APTT	Activated Partial Thromboplastin Time	N	Normal
FIB	Fibrinogen	H	Hemolyzed
AAPTT	Activated Partial Thromboplastin Time (Alternate)	+	Slight or Mild (pale/light red)
AT3	Antithrombin III	++	Moderate (red)
DDIM	D-Dimer	+++	Severe or Marked (dark red)
TT	Thrombin Time	I	Icterus
		L	Lipemia

CLINICAL CHEMISTRY

Abbreviation	Description	Abbreviation	Description
AST	Aspartate Aminotransferase	CRP	C-Reactive Protein
ALT	Alanine Aminotransferase	CSFGLU	CSF Glucose
ALP	Alkaline Phosphatase	CSFPRO	CSF Protein
GGT	Gamma Glutamyl Transferase	DBIL	Direct Bilirubin
TBIL	Total Bilirubin	FE	Iron
UREAN	Urea Nitrogen	FERR	Ferritin
CREAT	Creatinine	FPHGB	Plasma Free Hemoglobin
GLUC	Glucose	FRUC	Fructosamine
CHOL	Cholesterol	GLDH	Glutamate Dehydrogenase
TRIG	Triglycerides	GLYH	Glycosylated Hemoglobin
TPROT	Total Protein	HBGDL	Total Hemoglobin g/dL (HbA1c)
ALB	Albumin	HDL	High-density lipoprotein
GLOB	Globulin	IBIL	Indirect Bilirubin
A/G	Albumin/Globulin Ratio	IGA	Immunoglobulin A
CA	Calcium	IGG	Immunoglobulin G
PHOS	Phosphorus	IGM	Immunoglobulin M
NA	Sodium	LACT	Lactate
K	Potassium	LDH	Lactate Dehydrogenase
CL	Chloride	LDL	Low-density lipoprotein
A1C%	Glycosylated Hemoglobin Percent	LIPASE	Lipase
ALD2	Aldosterone (pg/mL, 2 decimal)	MG	Magnesium
AMM	Ammonia	NEFA	Non-Esterified Fatty Acids
AMYL	Amylase	OSMLTY	Osmolality
ANGAP	Anion Gap	PLIP	Phospholipids

Appendix 15

Abbreviation	Description	Abbreviation	Description
APOA	Apolipoprotein A-1	SDH	Sorbitol Dehydrogenase
APOB	Apolipoprotein B	TIBC	Total Iron Binding Capacity
BILEAC	Bile Acids	TRSMG	Transferrin (mg/dL)
CC3	Complement C3 (mg/dL)	U/C	Urea Nitrogen/Creatinine
CK	Creatine Kinase	UIBC	Unsaturated Iron Binding Capacity
CKMB	CK MB Isoenzyme	URIAC	Uric Acid
CO2	Bicarbonate	HEMO	Hemolytic

Hemolysis Indice:

Abbreviation	Description
N	Normal
H	Hemolyzed
+	Slight or Mild (pale/light red)
++	Moderate (red)
+++	Severe or Marked (dark red)

Lipemia Indice:

Abbreviation	Description
N	Normal
L	Lipemic
+	Slight or Mild (cloudy)
++	Moderate (turbid)
+++	Severe or Marked (opaque)

Icterus Indice:

Abbreviation	Description
N	Normal
I	Icterus
+	Slight or Mild (dark yellow)
++	Moderate (very dark yellow)
+++	Severe or Marked (dark yellow-green)

Appendix 15

Appendix 1

Individual Hematology Values

32025165

Sex: Male

0 vg			Reporting Hematology				
Group 1	Day(s) Relative to Start Date	WBC Reporting (10^3/uL)	NEUT (%)	LYMPH (%)	MONO (%)	EOS (%)	BASO (%)
1001	28	15.36	8.2	88.3	2.2	0.7	0.6
1002	28	11.28	23.5	73.0	2.6	0.5	0.4
1003	28	11.52	13.3	82.0	3.3	1.0	0.4
1004	28	6.04	13.6	82.8	2.6	0.7	0.3
1005	28	12.45	14.2	82.7	2.2	0.5	0.4
1006	89	9.67	16.9	79.4	2.0	1.2	0.5
1007	90	2.47	19.5	78.1	2.0	0.4	0.0
1008	91	16.82	24.2	69.5	5.4	0.7	0.2
1009	91	17.97	10.6	80.2	8.2	0.7	0.3
1010	92	8.90	12.3	78.5	5.3	2.6	1.3

DRAFT, NOT FOR SUBMISSION

Appendix 15

Appendix 1

Individual Hematology Values

32025165

Sex: Male

0 vg Group 1	Day(s) Relative to Start Date	Reporting Hematology NEUT (10^3/uL)	LYMPH (10^3/uL)	MONO (10^3/uL)	EOS (10^3/uL)	BASO (10^3/uL)	RBC (10^6/uL)
1001	28	1.27	13.56	0.34	0.10	0.09	8.31
1002	28	2.64	8.24	0.29	0.06	0.05	8.69
1003	28	1.53	9.45	0.38	0.11	0.05	9.01
1004	28	0.82	5.00	0.16	0.04	0.02	7.83
1005	28	1.77	10.30	0.27	0.06	0.05	9.03
1006	89	1.63	7.68	0.19	0.12	0.05	9.24
1007	90	0.48	1.93	0.05	0.01	0.00	8.83
1008	91	4.07	11.69	0.90	0.12	0.04	9.88
1009	91	1.89	14.41	1.48	0.13	0.06	10.23
1010	92	1.09	6.99	0.47	0.23	0.12	9.04

DRAFT, NOT FOR SUBMISSION

Appendix 15

Appendix 1

Individual Hematology Values

32025165

Sex: Male

0 vg		Reporting Hematology					
Group 1	Day(s) Relative to Start Date	HGB (g/dL)	HCT (%)	MCV (fL)	MCH (pg)	MCHC (g/dL)	RDW (%)
1001	28	15.6	50.1	60.3	18.8	31.1	13.6
1002	28	15.1	50.0	57.5	17.4	30.2	16.2
1003	28	17.3	55.9	62.0	19.2	30.9	14.1
1004	28	14.9	48.8	62.3	19.0	30.5	12.2
1005	28	16.4	53.3	59.0	18.2	30.8	15.1
1006	89	16.4	52.0	56.3	17.7	31.5	16.9
1007	90	15.6	46.3	52.4	17.7	33.7	16.4
1008	91	16.7	52.4	53.0	16.9	31.9	17.2
1009	91	17.8	53.9	52.7	17.4	33.0	17.6
1010	92	14.7	48.2	53.3	16.3	30.5	19.1

DRAFT, NOT FOR SUBMISSION

Appendix 15

Appendix 1

Individual Hematology Values

32025165

Sex: Male

0 vg		Reporting Hematology					
Group 1	Day(s) Relative to Start Date	PLT (10^3/uL)	RETIC (10^9/L)	RETIC (%)	RBC MORPH	PLATELET CLUMPS	WBC MORPH
1001	28	984	325.8	3.9	-	-	-
1002	28	1093	338.9	3.9	-	-	-
1003	28	1171	312.6	3.5	-	-	-
1004	28	998	263.9	3.4	-	-	-
1005	28	995	254.6	2.8	-	-	-
1006	89	991	224.5	2.4	-	-	-
1007	90	1051	212.8	2.4	-	-	-
1008	91	1095	266.8	2.7	-	-	-
1009	91	1087	194.4	1.9	-	-	-
1010	92	602	274.8	3.0	-	-	-

DRAFT, NOT FOR SUBMISSION

Appendix 15

Appendix 1

Individual Hematology Values

32025165

Sex: Male

1.49x10^11 vg<br>Group 2	Day(s) Relative to Start Date	WBC Reporting (10^3/uL)	Reporting Hematology NEUT (%)	LYMPH (%)	MONO (%)	EOS (%)	BASO (%)
2001	89	14.04	9.9	86.4	2.3	1.1	0.3
2002	90	12.84	5.3	90.5	3.0	0.9	0.3
2003	90	16.08	6.0	89.9	3.0	0.7	0.4
2004	90	16.21	6.4	90.2	2.4	0.6	0.4
2005	91	6.30	11.4	82.2	4.9	1.3	0.2
2006	91	11.69	12.9	81.9	4.2	0.7	0.3
2007	91	12.86	7.6	84.4	6.5	0.9	0.6
2008	91	16.91	7.9	87.4	3.8	0.5	0.4
2009	92	11.25	16.3	76.7	5.6	0.9	0.5
2010	92	10.87	11.5	81.2	6.2	0.7	0.4

DRAFT, NOT FOR SUBMISSION

Appendix 15

Appendix 1

Individual Hematology Values

32025165

Sex: Male

1.49x10^11 vg Group 2	Day(s) Relative to Start Date	Reporting Hematology NEUT (10^3/uL)	LYMPH (10^3/uL)	MONO (10^3/uL)	EOS (10^3/uL)	BASO (10^3/uL)	RBC (10^6/uL)
2001	89	1.40	12.13	0.32	0.15	0.04	8.40
2002	90	0.68	11.62	0.39	0.11	0.04	9.23
2003	90	0.96	14.46	0.48	0.11	0.07	9.01
2004	90	1.03	14.62	0.39	0.10	0.07	10.01
2005	91	0.72	5.18	0.31	0.08	0.01	8.59
2006	91	1.51	9.57	0.49	0.08	0.04	9.46
2007	91	0.98	10.86	0.83	0.11	0.08	9.58
2008	91	1.34	14.78	0.64	0.08	0.07	9.12
2009	92	1.83	8.63	0.63	0.10	0.06	10.11
2010	92	1.25	8.83	0.67	0.08	0.04	9.23

DRAFT, NOT FOR SUBMISSION

Appendix 15

Appendix 1

Individual Hematology Values

32025165

Sex: Male

1.49x10^11 vg<br>Group 2	Day(s) Relative to Start Date	Reporting Hematology<br>HGB (g/dL)	HCT (%)	MCV (fL)	MCH (pg)	MCHC (g/dL)	RDW (%)
2001	89	14.4	46.6	55.5	17.1	30.9	16.4
2002	90	16.5	52.1	56.4	17.9	31.7	16.5
2003	90	16.6	52.1	57.8	18.4	31.9	16.7
2004	90	16.6	52.3	52.2	16.6	31.7	17.7
2005	91	15.3	48.1	56.0	17.8	31.8	16.6
2006	91	15.7	48.9	51.7	16.6	32.1	17.5
2007	91	16.4	50.6	52.8	17.1	32.4	17.2
2008	91	15.2	48.0	52.6	16.7	31.7	17.0
2009	92	18.2	59.2	58.6	18.0	30.7	17.3
2010	92	16.3	52.0	56.3	17.7	31.3	17.8

DRAFT, NOT FOR SUBMISSION

Appendix 15

Appendix 1

Individual Hematology Values

32025165

Sex: Male

1.49x10^11 vg Group 2	Day(s) Relative to Start Date	Reporting Hematology PLT (10^3/uL)	RETIC (10^9/L)	RETIC (%)	RBC MORPH	PLATELET CLUMPS	WBC MORPH
2001	89	1119	331.8	4.0	-	-	-
2002	90	1098	267.7	2.9	-	-	-
2003	90	789	177.5	2.0	-	-	-
2004	90	994	245.2	2.5	-	-	-
2005	91	966	196.7	2.3	-	-	-
2006	91	1066	202.4	2.1	-	-	-
2007	91	1080	271.1	2.8	-	-	-
2008	91	1185	246.2	2.7	-	-	-
2009	92	1396	284.1	2.8	-	-	-
2010	92	1208	251.1	2.7	-	-	-

DRAFT, NOT FOR SUBMISSION

Appendix 15

Appendix 1

Individual Hematology Values

32025165

Sex: Male

4.47x10^11 vg Group 3	Day(s) Relative to Start Date	WBC Reporting (10^3/uL)	Reporting Hematology NEUT (%)	LYMPH (%)	MONO (%)	EOS (%)	BASO (%)
3001	89	12.44	9.2	89.0	1.3	0.2	0.3
3002	89	11.33	9.2	87.6	2.6	0.3	0.3
3003	90	12.20	11.8	84.3	2.5	1.1	0.3
3004	90	13.39	8.4	86.6	3.9	0.8	0.3
3005	90	11.17	8.9	87.4	2.1	1.3	0.3
3006	91	17.47	12.8	82.8	3.2	0.8	0.4
3007	91	18.39	6.9	89.2	3.2	0.5	0.2
3008	91	14.13	9.3	86.4	3.6	0.4	0.3
3009	92	7.76	16.2	76.4	6.4	0.6	0.4
3010	92	9.32	13.5	80.8	4.7	0.6	0.4

DRAFT, NOT FOR SUBMISSION

Appendix 15

Appendix 1

Individual Hematology Values

32025165

Sex: Male

4.47x10^11 vg Group 3	Day(s) Relative to Start Date	Reporting Hematology NEUT (10^3/uL)	LYMPH (10^3/uL)	MONO (10^3/uL)	EOS (10^3/uL)	BASO (10^3/uL)	RBC (10^6/uL)
3001	89	1.14	11.07	0.16	0.03	0.04	8.45
3002	89	1.05	9.92	0.30	0.03	0.03	9.39
3003	90	1.44	10.29	0.30	0.13	0.04	9.82
3004	90	1.13	11.59	0.52	0.11	0.04	8.60
3005	90	1.00	9.76	0.24	0.14	0.03	9.39
3006	91	2.24	14.46	0.56	0.14	0.07	9.04
3007	91	1.27	16.41	0.58	0.09	0.04	9.91
3008	91	1.31	12.21	0.51	0.06	0.04	9.27
3009	92	1.25	5.93	0.50	0.05	0.03	8.65
3010	92	1.25	7.53	0.44	0.06	0.04	8.94

Appendix 15

Appendix 1

Individual Hematology Values

32025165

Sex: Male

4.47x10^11 vg Group 3	Day(s) Relative to Start Date	Reporting Hematology					
		HGB (g/dL)	HCT (%)	MCV (fL)	MCH (pg)	MCHC (g/dL)	RDW (%)
3001	89	14.2	46.8	55.4	16.8	30.3	15.5
3002	89	15.8	51.0	54.3	16.8	31.0	16.8
3003	90	16.7	52.7	53.7	17.0	31.7	16.7
3004	90	15.7	48.9	56.9	18.3	32.1	16.7
3005	90	16.0	49.7	52.9	17.0	32.2	17.1
3006	91	16.6	50.9	56.3	18.4	32.6	16.1
3007	91	17.3	53.4	53.9	17.5	32.4	17.2
3008	91	16.3	50.1	54.0	17.6	32.5	16.2
3009	92	15.0	48.4	56.0	17.3	31.0	16.9
3010	92	15.4	48.6	54.4	17.2	31.7	18.6

DRAFT, NOT FOR SUBMISSION

Appendix 15

Appendix 1

Individual Hematology Values

32025165

Sex: Male

4.47x10^11 vg Group 3	Day(s) Relative to Start Date	Reporting Hematology PLT (10^3/uL)	RETIC (10^9/L)	RETIC (%)	RBC MORPH	PLATELET CLUMPS	WBC MORPH
3001	89	1154	200.3	2.4	-	-	-
3002	89	1108	233.8	2.5	-	-	-
3003	90	1322	237.6	2.4	-	-	-
3004	90	1249	228.8	2.7	-	-	-
3005	90	1334	195.3	2.1	-	-	-
3006	91	1105	254.9	2.8	-	-	-
3007	91	1336	245.8	2.5	-	-	-
3008	91	930	229.0	2.5	-	-	-
3009	92	1120	232.7	2.7	-	-	-
3010	92	1340	288.8	3.2	-	-	-

DRAFT, NOT FOR SUBMISSION

Appendix 15

Appendix 1

Individual Hematology Values

32025165

Sex: Male

1.49x10^12 vg Group 4	Day(s) Relative to Start Date	WBC Reporting (10^3/uL)	Reporting Hematology NEUT (%)	LYMPH (%)	MONO (%)	EOS (%)	BASO (%)
4001	28	13.36	14.7	80.1	3.8	0.8	0.6
4002	28	11.38	12.9	83.0	2.8	0.9	0.4
4003	28	13.19	19.2	73.9	3.8	2.6	0.5
4004	28	18.84	22.7	71.5	4.9	0.4	0.5
4005	28	19.41	8.6	87.9	2.5	0.6	0.4
4006	89	18.26	9.7	86.6	3.0	0.3	0.4
4007	89	7.08	7.1	88.8	2.8	0.6	0.7
4008	90	12.08	14.2	81.0	3.6	0.8	0.4
4009	90	10.30	9.9	85.0	4.3	0.4	0.4
4010	91	12.91	9.3	83.7	5.5	0.9	0.6
4011	91	11.42	14.2	75.3	8.3	1.8	0.4
4012	91	10.58	7.7	87.8	4.1	0.3	0.1
4013	92	11.98	12.4	81.8	4.4	1.1	0.3
4014	92	13.62	8.7	87.2	3.2	0.5	0.4
4015	92	13.45	6.6	87.7	3.9	1.2	0.6

DRAFT, NOT FOR SUBMISSION

Appendix 15

Appendix 1

Individual Hematology Values

32025165

Sex: Male

1.49x10^12 vg Group 4	Day(s) Relative to Start Date	Reporting Hematology					
		NEUT (10^3/uL)	LYMPH (10^3/uL)	MONO (10^3/uL)	EOS (10^3/uL)	BASO (10^3/uL)	RBC (10^6/uL)
4001	28	1.96	10.70	0.51	0.11	0.08	8.68
4002	28	1.46	9.45	0.32	0.10	0.05	7.86
4003	28	2.53	9.75	0.50	0.34	0.07	9.10
4004	28	4.26	13.48	0.93	0.07	0.10	9.31
4005	28	1.67	17.06	0.48	0.12	0.08	8.51
4006	89	1.78	15.81	0.54	0.06	0.07	9.41
4007	89	0.50	6.29	0.20	0.04	0.05	9.06
4008	90	1.70	9.79	0.44	0.10	0.05	9.01
4009	90	1.03	8.75	0.44	0.04	0.04	8.93
4010	91	1.21	10.80	0.71	0.11	0.08	8.75
4011	91	1.62	8.60	0.95	0.20	0.05	8.72
4012	91	0.82	9.29	0.43	0.03	0.01	8.38
4013	92	1.48	9.80	0.53	0.13	0.04	8.12
4014	92	1.19	11.87	0.43	0.07	0.06	9.02
4015	92	0.90	11.79	0.52	0.16	0.08	10.17

Appendix 15

Appendix 1

Individual Hematology Values

32025165

Sex: Male

1.49x10^12 vg Group 4	Day(s) Relative to Start Date	Reporting Hematology HGB (g/dL)	HCT (%)	MCV (fL)	MCH (pg)	MCHC (g/dL)	RDW (%)
4001	28	16.2	52.0	59.9	18.7	31.2	13.0
4002	28	15.3	49.4	62.8	19.5	31.0	12.4
4003	28	17.1	54.1	59.5	18.8	31.6	13.4
4004	28	17.1	55.0	59.1	18.4	31.1	16.0
4005	28	16.3	52.4	61.6	19.2	31.1	14.6
4006	89	16.0	50.3	53.5	17.0	31.8	17.9
4007	89	15.1	48.5	53.5	16.7	31.1	16.2
4008	90	15.3	48.2	53.5	17.0	31.7	17.3
4009	90	14.5	45.6	51.1	16.2	31.8	17.9
4010	91	14.4	45.0	51.4	16.5	32.0	17.4
4011	91	15.3	48.2	55.3	17.5	31.7	16.2
4012	91	14.1	42.8	51.1	16.8	32.9	15.9
4013	92	14.9	46.5	57.3	18.3	32.0	17.2
4014	92	15.7	49.4	54.8	17.4	31.8	17.0
4015	92	17.1	53.8	52.9	16.8	31.8	17.9

Appendix 15

Appendix 1

Individual Hematology Values

32025165

Sex: Male

1.49x10^12 vg<br>Group 4	Day(s) Relative to Start Date	Reporting Hematology<br>PLT (10^3/uL)	RETIC (10^9/L)	RETIC (%)	RBC MORPH	PLATELET CLUMPS	WBC MORPH
4001	28	972	266.5	3.1	-	-	-
4002	28	794	254.7	3.2	-	-	-
4003	28	757	262.1	2.9	-	-	-
4004	28	1114	363.1	3.9	-	-	-
4005	28	1221	320.8	3.8	-	-	-
4006	89	1170	287.9	3.1	-	-	-
4007	89	978	215.6	2.4	-	-	-
4008	90	1239	260.4	2.9	-	-	-
4009	90	1431	279.5	3.1	-	-	-
4010	91	1121	231.9	2.7	-	-	-
4011	91	1479	312.2	3.6	-	-	-
4012	91	809	201.1	2.4	-	-	-
4013	92	806	254.2	3.1	-	-	-
4014	92	1512	257.1	2.9	-	-	-
4015	92	1226	246.1	2.4	-	-	-

DRAFT, NOT FOR SUBMISSION

Appendix 15

Appendix 1

Individual Hematology Values

32025165

Sex: Female

0 vg		Reporting Hematology					
Group 1	Day(s) Relative to Start Date	WBC Reporting (10^3/uL)	NEUT (%)	LYMPH (%)	MONO (%)	EOS (%)	BASO (%)
1501	29	5.91	19.3	77.0	2.4	0.3	1.0
1502	29	7.71	10.0	83.8	4.2	1.2	0.8
1503	29	8.22	12.5	83.1	2.9	0.9	0.6
1504	29	5.70	11.8	83.5	3.0	1.2	0.5
1505	29	13.40	9.4	87.5	2.2	0.7	0.2
1506	91	CLOT	CLOT	CLOT	CLOT	CLOT	CLOT
1507	91	4.75	10.2	80.6	4.0	4.8	0.4
1508	91	6.79	16.2	79.4	3.1	0.6	0.7
1509	92	6.59	9.5	86.9	2.3	0.8	0.5
1510	93	12.16	6.3	89.5	3.3	0.7	0.2

DRAFT, NOT FOR SUBMISSION

Appendix 15

Appendix 1

Individual Hematology Values

32025165

Sex: Female

0 vg		Reporting Hematology					
Group 1	Day(s) Relative to Start Date	NEUT (10^3/uL)	LYMPH (10^3/uL)	MONO (10^3/uL)	EOS (10^3/uL)	BASO (10^3/uL)	RBC (10^6/uL)
1501	29	1.14	4.55	0.14	0.02	0.06	8.36
1502	29	0.78	6.46	0.32	0.09	0.06	8.35
1503	29	1.03	6.83	0.24	0.07	0.05	8.05
1504	29	0.67	4.76	0.17	0.07	0.03	8.25
1505	29	1.25	11.73	0.30	0.09	0.03	8.60
1506	91	CLOT	CLOT	CLOT	CLOT	CLOT	CLOT
1507	91	0.48	3.83	0.19	0.23	0.02	8.13
1508	91	1.10	5.39	0.21	0.04	0.05	8.68
1509	92	0.63	5.73	0.15	0.05	0.03	7.72
1510	93	0.76	10.88	0.40	0.09	0.03	8.38

DRAFT, NOT FOR SUBMISSION

Appendix 15

Appendix 1

Individual Hematology Values

32025165

Sex: Female

0 vg		Reporting Hematology					
Group 1	Day(s) Relative to Start Date	HGB (g/dL)	HCT (%)	MCV (fL)	MCH (pg)	MCHC (g/dL)	RDW (%)
1501	29	15.1	46.9	56.1	18.1	32.2	13.2
1502	29	15.2	47.7	57.1	18.2	31.9	12.5
1503	29	15.2	47.7	59.3	18.9	31.9	12.4
1504	29	15.2	47.4	57.5	18.4	32.1	12.0
1505	29	16.0	50.0	58.1	18.6	32.0	13.5
1506	91	CLOT	CLOT	CLOT	CLOT	CLOT	CLOT
1507	91	14.8	46.3	56.9	18.2	32.0	11.9
1508	91	16.2	50.4	58.1	18.7	32.1	14.0
1509	92	14.7	44.9	58.2	19.0	32.7	13.3
1510	93	15.7	49.2	58.7	18.7	31.9	13.6

DRAFT, NOT FOR SUBMISSION

Appendix 15

Appendix 1

Individual Hematology Values

32025165

Sex: Female

0 vg		Reporting Hematology					
Group 1	Day(s) Relative to Start Date	PLT (10^3/uL)	RETIC (10^9/L)	RETIC (%)	RBC MORPH	PLATELET CLUMPS	WBC MORPH
1501	29	1259	281.7	3.4	-	-	-
1502	29	1121	264.7	3.2	-	-	-
1503	29	1022	186.8	2.3	-	-	-
1504	29	1055	196.4	2.4	-	-	-
1505	29	1226	250.3	2.9	-	-	-
1506	91	CLOT	CLOT	CLOT	-	-	-
1507	91	1003	178.9	2.2	-	-	-
1508	91	879	266.5	3.1	-	-	-
1509	92	920	191.5	2.5	-	-	-
1510	93	974	202.8	2.4	-	-	-

Appendix 15

Appendix 1

Individual Hematology Values

32025165

Sex: Female

1.49x10^11 vg<br>Group 2	Day(s) Relative to Start Date	WBC Reporting (10^3/uL)	Reporting Hematology<br>NEUT (%)	LYMPH (%)	MONO (%)	EOS (%)	BASO (%)
2501	91	9.18	9.7	86.8	2.6	0.5	0.4
2502	91	10.34	14.1	79.6	5.3	0.8	0.2
2503	91	9.11	10.5	85.9	3.0	0.4	0.2
2504	91	9.35	17.0	77.9	3.7	1.0	0.4
2505	91	9.24	9.7	86.6	2.4	0.9	0.4
2506	91	6.37	8.0	89.0	3.0	0.0	0.0
2507	92	8.46	8.2	87.5	3.0	0.8	0.5
2508	92	CLOT	CLOT	CLOT	CLOT	CLOT	CLOT
2509	92	CLOT	CLOT	CLOT	CLOT	CLOT	CLOT
2510	93	6.22	12.1	81.0	5.8	0.8	0.3

DRAFT, NOT FOR SUBMISSION

Appendix 15

Appendix 1

Individual Hematology Values

32025165

Sex: Female

1.49x10^11 vg<br>Group 2	Day(s) Relative to Start Date	Reporting Hematology<br>NEUT (10^3/uL)	LYMPH (10^3/uL)	MONO (10^3/uL)	EOS (10^3/uL)	BASO (10^3/uL)	RBC (10^6/uL)
2501	91	0.88	7.97	0.24	0.05	0.04	7.99
2502	91	1.46	8.23	0.55	0.08	0.02	7.81
2503	91	0.95	7.83	0.27	0.04	0.02	8.31
2504	91	1.59	7.28	0.35	0.09	0.04	8.37
2505	91	0.90	8.00	0.22	0.08	0.04	8.25
2506	91	0.51	5.67	0.19	0.00	0.00	7.79
2507	92	0.70	7.40	0.25	0.07	0.04	8.17
2508	92	CLOT	CLOT	CLOT	CLOT	CLOT	CLOT
2509	92	CLOT	CLOT	CLOT	CLOT	CLOT	CLOT
2510	93	0.75	5.04	0.36	0.05	0.02	8.18

DRAFT, NOT FOR SUBMISSION

Appendix 15

Appendix 1

Individual Hematology Values

32025165

Sex: Female

1.49x10^11 vg Group 2	Day(s) Relative to Start Date	Reporting Hematology HGB (g/dL)	HCT (%)	MCV (fL)	MCH (pg)	MCHC (g/dL)	RDW (%)
2501	91	14.3	45.2	56.6	17.9	31.6	14.0
2502	91	14.1	44.1	56.5	18.1	32.0	13.2
2503	91	14.9	47.1	56.7	17.9	31.6	12.6
2504	91	15.8	49.4	59.0	18.9	32.0	13.5
2505	91	15.6	48.6	58.9	18.9	32.1	13.6
2506	91	14.8	44.4	57.0	19.0	33.3	13.6
2507	92	15.2	48.3	59.1	18.6	31.5	13.2
2508	92	CLOT	CLOT	CLOT	CLOT	CLOT	CLOT
2509	92	CLOT	CLOT	CLOT	CLOT	CLOT	CLOT
2510	93	14.9	46.3	56.6	18.2	32.2	12.5

DRAFT, NOT FOR SUBMISSION

Appendix 15

Appendix 1

Individual Hematology Values

32025165

Sex: Female

1.49x10^11 vg Group 2	Day(s) Relative to Start Date	Reporting Hematology PLT (10^3/uL)	RETIC (10^9/L)	RETIC (%)	RBC MORPH	PLATELET CLUMPS	WBC MORPH
2501	91	1104	167.0	2.1	-	-	-
2502	91	1001	201.5	2.6	-	-	-
2503	91	1076	186.1	2.2	-	-	-
2504	91	1041	161.5	1.9	-	-	-
2505	91	1068	199.7	2.4	-	-	-
2506	91	815	204.9	2.6	NAF	4+	NAF
2507	92	1193	180.6	2.2	-	-	-
2508	92	CLOT	CLOT	CLOT	-	-	-
2509	92	CLOT	CLOT	CLOT	-	-	-
2510	93	1141	198.8	2.4	-	-	-

DRAFT, NOT FOR SUBMISSION

Appendix 15

Appendix 1

Individual Hematology Values

32025165

Sex: Female

4.47x10^11 vg<br>Group 3	Day(s) Relative to Start Date	WBC Reporting (10^3/uL)	Reporting Hematology<br>NEUT (%)	LYMPH (%)	MONO (%)	EOS (%)	BASO (%)
3501	91	7.25	11.9	83.3	3.7	0.8	0.3
3502	91	10.99	7.8	88.4	2.9	0.5	0.4
3503	91	14.54	5.1	92.0	2.1	0.5	0.3
3504	91	13.34	6.5	90.1	2.4	0.7	0.3
3505	91	7.24	9.3	87.4	2.6	0.4	0.3
3506	91	15.48	9.6	87.7	1.8	0.6	0.3
3507	92	5.89	10.3	85.6	3.1	0.8	0.2
3508	92	4.83	9.0	85.7	3.7	0.8	0.8
3509	92	11.38	15.1	79.1	4.6	0.8	0.4
3510	93	6.59	6.6	84.8	6.8	1.2	0.6

DRAFT, NOT FOR SUBMISSION

Appendix 15

Appendix 1

Individual Hematology Values

32025165

Sex: Female

4.47x10^11 vg<br>Group 3	Day(s) Relative to Start Date	Reporting Hematology<br>NEUT (10^3/uL)	LYMPH (10^3/uL)	MONO (10^3/uL)	EOS (10^3/uL)	BASO (10^3/uL)	RBC (10^6/uL)
3501	91	0.86	6.04	0.27	0.06	0.02	8.16
3502	91	0.85	9.72	0.32	0.06	0.04	8.47
3503	91	0.73	13.38	0.31	0.07	0.05	8.64
3504	91	0.87	12.02	0.32	0.09	0.04	8.48
3505	91	0.67	6.33	0.19	0.03	0.02	8.39
3506	91	1.48	13.58	0.28	0.10	0.04	8.31
3507	92	0.61	5.04	0.18	0.05	0.01	8.02
3508	92	0.43	4.14	0.18	0.04	0.04	7.87
3509	92	1.73	9.00	0.52	0.09	0.04	8.52
3510	93	0.43	5.59	0.45	0.08	0.04	8.25

DRAFT, NOT FOR SUBMISSION

Appendix 15

Appendix 1

Individual Hematology Values

32025165

Sex: Female

4.47x10^11 vg Group 3	Day(s) Relative to Start Date	Reporting Hematology HGB (g/dL)	HCT (%)	MCV (fL)	MCH (pg)	MCHC (g/dL)	RDW (%)
3501	91	14.8	46.2	56.6	18.1	32.0	14.0
3502	91	16.1	49.6	58.6	19.0	32.5	13.2
3503	91	15.6	48.2	55.8	18.1	32.4	14.1
3504	91	15.7	49.6	58.5	18.5	31.7	13.3
3505	91	15.4	48.6	57.9	18.4	31.7	14.3
3506	91	15.4	47.7	57.4	18.5	32.3	13.9
3507	92	14.6	46.4	57.9	18.2	31.5	13.6
3508	92	14.7	46.4	59.0	18.7	31.7	13.2
3509	92	15.7	49.7	58.3	18.4	31.6	14.0
3510	93	15.9	49.5	60.0	19.3	32.1	12.0

DRAFT, NOT FOR SUBMISSION

Appendix 15

Appendix 1

Individual Hematology Values

32025165

Sex: Female

4.47x10^11 vg Group 3	Day(s) Relative to Start Date	Reporting Hematology PLT (10^3/uL)	RETIC (10^9/L)	RETIC (%)	RBC MORPH	PLATELET CLUMPS	WBC MORPH
3501	91	937	175.4	2.2	-	-	-
3502	91	1024	194.0	2.3	-	-	-
3503	91	971	176.3	2.0	-	-	-
3504	91	1011	178.9	2.1	-	-	-
3505	91	953	232.4	2.8	-	-	-
3506	91	990	208.6	2.5	-	-	-
3507	92	1041	243.0	3.0	-	-	-
3508	92	858	177.1	2.3	-	-	-
3509	92	1106	129.5	1.5	-	-	-
3510	93	1006	198.0	2.4	-	-	-

Appendix 15

Appendix 1

Individual Hematology Values

32025165

Sex: Female

1.49x10^12 vg Group 4	Day(s) Relative to Start Date	WBC Reporting (10^3/uL)	NEUT (%)	LYMPH (%)	MONO (%)	EOS (%)	BASO (%)
		Reporting Hematology					
4501	29	1.60	24.4	72.5	2.5	0.6	0.0
4502	29	9.55	11.6	81.9	4.5	1.3	0.7
4503	29	4.76	16.3	80.0	2.7	0.8	0.2
4504	29	11.70	6.7	89.3	2.1	1.1	0.8
4505	29	13.01	9.8	84.7	3.8	1.2	0.5
4506	91	6.33	9.2	86.4	3.5	0.6	0.3
4507	91	12.13	6.5	89.1	3.5	0.7	0.2
4508	91	11.13	7.7	85.6	5.2	1.1	0.4
4509	91	9.24	6.4	88.7	4.0	0.4	0.5
4510	91	11.05	7.6	87.0	4.3	0.6	0.5
4511	92	10.12	6.8	88.6	3.4	0.7	0.5
4512	92	9.76	10.1	84.1	4.6	0.8	0.4
4513	92	11.93	5.7	91.0	2.6	0.4	0.3
4514	93	6.52	10.9	81.9	5.8	0.9	0.5
4515	93	8.28	12.9	79.2	6.6	1.1	0.2

DRAFT, NOT FOR SUBMISSION

Appendix 15

Appendix 1

Individual Hematology Values

32025165

Sex: Female

1.49x10^12 vg<br>Group 4	Day(s) Relative to Start Date	Reporting Hematology<br>NEUT (10^3/uL)	LYMPH (10^3/uL)	MONO (10^3/uL)	EOS (10^3/uL)	BASO (10^3/uL)	RBC (10^6/uL)
4501	29	0.39	1.16	0.04	0.01	0.00	7.79
4502	29	1.11	7.82	0.43	0.12	0.07	7.88
4503	29	0.77	3.81	0.13	0.04	0.01	8.11
4504	29	0.78	10.45	0.25	0.13	0.09	8.44
4505	29	1.28	11.02	0.50	0.15	0.06	8.19
4506	91	0.58	5.47	0.22	0.04	0.02	7.87
4507	91	0.78	10.81	0.42	0.09	0.03	8.13
4508	91	0.86	9.53	0.58	0.12	0.04	8.50
4509	91	0.58	8.20	0.37	0.04	0.05	8.29
4510	91	0.85	9.61	0.47	0.07	0.05	8.59
4511	92	0.69	8.97	0.34	0.07	0.05	8.44
4512	92	0.98	8.21	0.45	0.08	0.04	8.10
4513	92	0.68	10.86	0.31	0.05	0.03	8.64
4514	93	0.71	5.34	0.38	0.06	0.03	8.34
4515	93	1.06	6.56	0.55	0.09	0.02	7.79

DRAFT, NOT FOR SUBMISSION

Appendix 15

Appendix 1

Individual Hematology Values

32025165

Sex: Female

1.49x10^12 vg<br>Group 4	Day(s) Relative to Start Date	Reporting Hematology HGB (g/dL)	HCT (%)	MCV (fL)	MCH (pg)	MCHC (g/dL)	RDW (%)
4501	29	14.5	45.7	58.7	18.6	31.7	12.0
4502	29	14.7	45.8	58.1	18.7	32.1	11.1
4503	29	15.5	47.6	58.7	19.1	32.6	11.6
4504	29	16.0	49.2	58.3	19.0	32.5	12.2
4505	29	15.1	46.5	56.8	18.4	32.5	12.0
4506	91	14.8	46.1	58.6	18.8	32.1	14.7
4507	91	15.3	47.9	58.9	18.8	31.9	12.3
4508	91	15.2	47.0	55.3	17.9	32.3	13.5
4509	91	15.2	46.5	56.1	18.3	32.7	13.1
4510	91	16.1	49.4	57.5	18.7	32.6	13.3
4511	92	15.0	47.4	56.2	17.8	31.6	13.4
4512	92	14.9	46.3	57.2	18.4	32.2	13.1
4513	92	15.7	49.2	56.9	18.2	31.9	14.0
4514	93	15.9	49.4	59.2	19.1	32.2	13.4
4515	93	15.2	47.6	61.1	19.5	31.9	12.4

DRAFT, NOT FOR SUBMISSION

Appendix 15

Appendix 1

Individual Hematology Values

32025165

Sex: Female

1.49x10^12 vg Group 4	Day(s) Relative to Start Date	Reporting Hematology PLT (10^3/uL)	RETIC (10^9/L)	RETIC (%)	RBC MORPH	PLATELET CLUMPS	WBC MORPH
4501	29	1071	278.9	3.6	-	-	-
4502	29	1147	134.7	1.7	-	-	-
4503	29	959	220.6	2.7	-	-	-
4504	29	1202	306.4	3.6	-	-	-
4505	29	1170	195.7	2.4	-	-	-
4506	91	982	242.4	3.1	-	-	-
4507	91	835	155.3	1.9	-	-	-
4508	91	810	164.1	1.9	-	-	-
4509	91	1001	186.5	2.3	-	-	-
4510	91	1073	213.0	2.5	-	-	-
4511	92	1237	199.2	2.4	-	-	-
4512	92	1156	188.7	2.3	-	-	-
4513	92	936	200.4	2.3	-	-	-
4514	93	987	210.2	2.5	-	-	-
4515	93	1109	217.3	2.8	-	-	-

DRAFT, NOT FOR SUBMISSION

Appendix 15

Appendix 2

Individual Coagulation Values

32025165

Sex: Male

0 vg		Reporting Coagulation			
Group 1	Day(s) Relative to Start Date	PT (sec)	APTT (sec)	FIB (mg/dL)	SAMQ Coagulation
1001	28	17.1	11.2	323	N
1002	28	16.2	10.9	320	N
1003	28	15.6	10.4	379	H+
1004	28	CLOT	CLOT	CLOT	CLOT
1005	28	15.7	13.4	323	N
1006	89	15.5	16.0	344	N
1007	90	16.1	14.9	332	N
1008	91	15.2	13.1	363	N
1009	91	16.2	16.7	412	H+
1010	92	CLOT	CLOT	CLOT	CLOT

DRAFT, NOT FOR SUBMISSION

Appendix 15

Appendix 2

Individual Coagulation Values

32025165

Sex: Male

1.49x10^11 vg Group 2	Day(s) Relative to Start Date	Reporting Coagulation PT (sec)	APTT (sec)	FIB (mg/dL)	SAMQ Coagulation
2001	89	15.7	12.1	368	N
2002	90	16.6	13.7	364	N
2003	90	16.5	12.3	334	N
2004	90	17.5	14.1	333	N
2005	91	15.8	15.8	339	N
2006	91	16.0	13.0	318	N
2007	91	16.2	14.3	311	N
2008	91	16.1	14.6	342	N
2009	92	16.4	12.5	345	N
2010	92	16.0	15.8	333	N

DRAFT, NOT FOR SUBMISSION

Appendix 15

Appendix 2

Individual Coagulation Values

32025165

Sex: Male

4.47x10^11 vg Group 3	Day(s) Relative to Start Date	Reporting Coagulation PT (sec)	APTT (sec)	FIB (mg/dL)	SAMQ Coagulation
3001	89	14.8	9.5	308	N
3002	89	16.1	11.6	345	N
3003	90	16.3	11.3	353	N
3004	90	16.1	15.0	350	N
3005	90	17.0	12.1	352	N
3006	91	16.1	14.5	326	N
3007	91	17.0	13.7	314	N
3008	91	16.1	14.3	313	N
3009	92	15.3	10.5	342	N
3010	92	16.4	13.9	329	N

DRAFT, NOT FOR SUBMISSION

Appendix 15

Appendix 2

Individual Coagulation Values

32025165

Sex: Male

1.49x10^12 vg Group 4	Day(s) Relative to Start Date	Reporting Coagulation PT (sec)	APTT (sec)	FIB (mg/dL)	SAMQ Coagulation
4001	28	16.1	14.8	330	N
4002	28	16.2	11.9	330	H+
4003	28	CLOT	CLOT	CLOT	CLOT
4004	28	15.6	14.0	389	N
4005	28	16.8	11.2	358	N
4006	89	15.5	14.1	408	N
4007	89	15.8	10.9	334	N
4008	90	17.1	13.9	350	N
4009	90	16.9	10.2	426	N
4010	91	17.0	13.3	317	N
4011	91	17.7	13.4	325	N
4012	91	16.3	11.7	369	N
4013	92	18.0	13.6	316	N
4014	92	16.1	14.6	320	N
4015	92	17.6	15.0	318	N

DRAFT, NOT FOR SUBMISSION

Appendix 15

Appendix 2

Individual Coagulation Values

32025165

Sex: Female

0 vg		Reporting Coagulation			
Group 1	Day(s) Relative to Start Date	PT (sec)	APTT (sec)	FIB (mg/dL)	SAMQ Coagulation
1501	29	16.6	16.3	236	N
1502	29	17.7	13.7	228	H+
1503	29	17.9	12.2	272	N
1504	29	15.7	8.3	265	N
1505	29	17.0	14.5	227	N
1506	91	CLOT	CLOT	CLOT	CLOT
1507	91	CLOT	CLOT	CLOT	CLOT
1508	91	17.0	12.9	211	N
1509	92	17.3	15.6	212	N
1510	93	16.7	15.3	229	N

DRAFT, NOT FOR SUBMISSION

Appendix 15

Appendix 2

Individual Coagulation Values

32025165

Sex: Female

1.49x10^11 vg Group 2	Day(s) Relative to Start Date	Reporting Coagulation PT (sec)	APTT (sec)	FIB (mg/dL)	SAMQ Coagulation
2501	91	15.9	9.7	251	N
2502	91	17.9	14.2	309	N
2503	91	18.1	14.6	235	N
2504	91	16.8	10.7	240	N
2505	91	15.8	14.4	256	N
2506	91	CLOT	CLOT	CLOT	CLOT
2507	92	16.7	13.5	214	N
2508	92	CLOT	CLOT	CLOT	CLOT
2509	92	CLOT	CLOT	CLOT	CLOT
2510	93	17.1	15.1	240	N

Appendix 15

Appendix 2

Individual Coagulation Values

32025165

Sex: Female

4.47x10^11 vg Group 3	Day(s) Relative to Start Date	Reporting Coagulation			
		PT (sec)	APTT (sec)	FIB (mg/dL)	SAMQ Coagulation
3501	91	16.0	13.6	260	N
3502	91	16.8	12.8	221	N
3503	91	16.3	12.0	241	N
3504	91	16.3	11.8	234	N
3505	91	17.0	14.8	244	N
3506	91	14.9	9.3	189	N
3507	92	16.7	15.5	195	N
3508	92	16.2	13.1	208	N
3509	92	15.9	10.0	256	N
3510	93	16.2	15.3	207	N

DRAFT, NOT FOR SUBMISSION

Appendix 15

Appendix 2

Individual Coagulation Values

32025165

Sex: Female

1.49x10^12 vg<br>Group 4	Day(s) Relative to Start Date	Reporting Coagulation PT (sec)	APTT (sec)	FIB (mg/dL)	SAMQ Coagulation
4501	29	17.1	13.3	237	N
4502	29	15.5	14.5	236	N
4503	29	16.0	13.5	236	H+
4504	29	17.1	15.0	246	N
4505	29	17.3	15.5	279	N
4506	91	16.3	13.1	215	N
4507	91	16.8	13.3	227	N
4508	91	15.2	9.1	173	N
4509	91	16.0	11.9	244	N
4510	91	15.3	13.9	204	N
4511	92	15.5	11.9	183	N
4512	92	16.1	11.8	210	N
4513	92	17.4	14.3	180	N
4514	93	16.0	16.6	225	N
4515	93	16.7	16.3	220	N

DRAFT, NOT FOR SUBMISSION

Appendix 15

Appendix 3

Individual Clinical Chemistry Values

32025165

Sex: Male

0 vg		Reporting Biochemistry					
Group 1	Day(s) Relative to Start Date	AST (U/L)	ALT (U/L)	ALP (U/L)	GGT (U/L)	TBIL (mg/dL)	UREAN (mg/dL)
1001	28	97	68	205	1 [a]	0.14	13
1002	28	121	55	335	1 [a]	0.12	10
1003	28	131	80	334	1 [a]	0.15	13
1004	28	101	51	266	1 [a]	0.13	15
1005	28	99	81	249	1 [a]	0.13	11
1006	89	80	54	115	1 [a]	0.14	11
1007	90	64	42	90	1 [a]	0.12	11
1008	91	74	52	121	1 [a]	0.13	11
1009	91	82	56	175	1 [a]	0.20	22
1010	92	382	193	143	1 [a]	0.19	17

[a] [RC:Assigned value below the reportable range]

DRAFT, NOT FOR SUBMISSION

Appendix 15

Appendix 3

Individual Clinical Chemistry Values

32025165

Sex: Male

0 vg		Reporting Biochemistry					
Group 1	Day(s) Relative to Start Date	CREAT (mg/dL)	GLUC (mg/dL)	CHOL (mg/dL)	TRIG (mg/dL)	TPROT (g/dL)	ALB (g/dL)
1001	28	0.4	475	91	214	7.4	3.8
1002	28	0.4	478	82	111	6.7	3.7
1003	28	0.4	427	116	206	7.6	3.9
1004	28	0.3	197	106	165	6.6	3.6
1005	28	0.4	311	103	114	6.6	3.6
1006	89	0.4	301	81	157	7.4	3.6
1007	90	0.3	171	61	385	6.9	3.4
1008	91	0.4	441	73	290	7.7	3.9
1009	91	0.4	240	95	131	7.0	3.5
1010	92	0.6	204	114	69	7.6	3.7

DRAFT, NOT FOR SUBMISSION

Appendix 15

Appendix 3

Individual Clinical Chemistry Values

32025165

Sex: Male

0 vg		Reporting Biochemistry					
Group 1	Day(s) Relative to Start Date	GLOB (g/dL)	A/G (ratio)	CA (mg/dL)	PHOS (mg/dL)	NA (mEq/L)	K (mEq/L)
1001	28	3.6	1.1	13.8	11.6	144	7.8
1002	28	3.0	1.2	13.3	10.0	147	7.0
1003	28	3.7	1.1	13.7	12.3	144	9.1
1004	28	3.0	1.2	12.8	11.9	154	7.2
1005	28	3.0	1.2	12.7	11.9	147	7.9
1006	89	3.8	0.9	11.7	7.3	145	6.5
1007	90	3.5	1.0	11.7	9.7	141	7.4
1008	91	3.8	1.0	13.9	8.7	144	8.5
1009	91	3.5	1.0	11.6	6.5	144	5.8
1010	92	3.9	0.9	11.7	7.2	147	6.5

DRAFT, NOT FOR SUBMISSION

Appendix 15

Appendix 3

Individual Clinical Chemistry Values

32025165

Sex: Male

0 vg		Reporting Biochemistry				
Group 1	Day(s) Relative to Start Date	CL (mEq/L)	HEMO INDICE	ICTERUS INDICE	LIPEMIA INDICE	SDH Alternate (U/L)
1001	28	98	+	N	N	35.6
1002	28	101	N	N	N	18.9
1003	28	97	+	N	N	76.0
1004	28	104	N	N	N	14.3
1005	28	101	N	N	N	35.0
1006	89	104	N	N	N	10.0
1007	90	99	+	N	N	9.1
1008	91	99	+	N	N	18.0
1009	91	95	++	N	N	12.1
1010	92	97	+	N	N	141.6

DRAFT, NOT FOR SUBMISSION

Appendix 15

Appendix 3

Individual Clinical Chemistry Values

32025165

Sex: Male

1.49x10^11 vg Group 2	Day(s) Relative to Start Date	Reporting Biochemistry AST (U/L)	ALT (U/L)	ALP (U/L)	GGT (U/L)	TBIL (mg/dL)	UREAN (mg/dL)
2001	89	128	56	117	1 [a]	0.16	14
2002	90	62	40	108	1 [a]	0.16	13
2003	90	116	113	88	1 [a]	0.11	12
2004	90	69	56	127	1 [a]	0.14	13
2005	91	55	40	228	1 [a]	0.14	11
2006	91	90	59	192	1 [a]	0.15	12
2007	91	82	40	177	1 [a]	0.17	14
2008	91	87	54	146	1 [a]	0.15	11
2009	92	106	71	152	1 [a]	0.13	13
2010	92	85	70	171	1 [a]	0.16	12

[a] [RC:Assigned value below the reportable range]

Appendix 15

Appendix 3

Individual Clinical Chemistry Values

32025165

Sex: Male

1.49x10^11 vg<br>Group 2	Day(s) Relative to Start Date	Reporting Biochemistry CREAT (mg/dL)	GLUC (mg/dL)	CHOL (mg/dL)	TRIG (mg/dL)	TPROT (g/dL)	ALB (g/dL)
2001	89	0.3	398	86	199	7.8	3.9
2002	90	0.5	412	95	334	8.2	4.2
2003	90	0.5	370	59	146	7.0	3.4
2004	90	0.4	314	58	248	7.2	3.7
2005	91	0.4	185	78	248	7.0	3.6
2006	91	0.3	255	51	269	7.0	3.6
2007	91	0.4	315	61	216	7.2	3.6
2008	91	0.3	279	88	254	7.3	3.5
2009	92	0.5	421	72	146	7.5	3.5
2010	92	0.3	225	72	238	7.2	3.6

DRAFT, NOT FOR SUBMISSION

Appendix 15

Appendix 3

Individual Clinical Chemistry Values

32025165

Sex: Male

1.49x10^11 vg<br>Group 2	Day(s) Relative to Start Date	Reporting Biochemistry GLOB (g/dL)	A/G (ratio)	CA (mg/dL)	PHOS (mg/dL)	NA (mEq/L)	K (mEq/L)
2001	89	3.9	1.0	13.1	9.0	144	8.2
2002	90	4.0	1.1	14.0	10.7	142	8.8
2003	90	3.6	0.9	12.7	9.5	142	9.2
2004	90	3.5	1.1	12.4	9.7	143	7.8
2005	91	3.4	1.1	12.2	9.3	147	6.4
2006	91	3.4	1.1	11.9	8.5	142	7.8
2007	91	3.6	1.0	13.1	8.9	142	8.1
2008	91	3.8	0.9	12.7	9.8	146	6.2
2009	92	4.0	0.9	13.3	9.5	143	8.0
2010	92	3.6	1.0	11.8	8.0	147	6.0

DRAFT, NOT FOR SUBMISSION

Appendix 15

Appendix 3

Individual Clinical Chemistry Values

32025165

Sex: Male

1.49x10^11 vg Group 2	Day(s) Relative to Start Date	Reporting Biochemistry CL (mEq/L)	HEMO INDICE	ICTERUS INDICE	LIPEMIA INDICE	SDH Alternate (U/L)
2001	89	99	N	N	N	29.8
2002	90	98	N	N	N	14.5
2003	90	101	N	N	N	75.4
2004	90	99	+	N	N	14.7
2005	91	99	N	N	N	4.3
2006	91	98	++	N	N	13.1
2007	91	99	+	N	N	8.6
2008	91	98	N	N	N	19.1
2009	92	99	N	N	N	12.1
2010	92	101	+	N	N	17.2

DRAFT, NOT FOR SUBMISSION

Appendix 15

Appendix 3

Individual Clinical Chemistry Values

32025165

Sex: Male

4.47x10^11 vg Group 3	Day(s) Relative to Start Date	Reporting Biochemistry AST (U/L)	ALT (U/L)	ALP (U/L)	GGT (U/L)	TBIL (mg/dL)	UREAN (mg/dL)
3001	89	98	40	145	1 [a]	0.13	13
3002	89	103	44	117	1 [a]	0.13	13
3003	90	68	55	132	1 [a]	0.13	15
3004	90	62	41	88	1 [a]	0.14	12
3005	90	55	40	140	1 [a]	0.15	12
3006	91	63	46	140	1 [a]	0.19	13
3007	91	90	62	115	1 [a]	0.15	14
3008	91	105	66	167	1 [a]	0.13	13
3009	92	171	113	131	1 [a]	0.13	15
3010	92	63	48	197	1 [a]	0.13	14

[a] [RC:Assigned value below the reportable range]

DRAFT, NOT FOR SUBMISSION

Appendix 15

Appendix 3

Individual Clinical Chemistry Values

32025165

Sex: Male

4.47x10^11 vg Group 3	Day(s) Relative to Start Date	Reporting Biochemistry CREAT (mg/dL)	GLUC (mg/dL)	CHOL (mg/dL)	TRIG (mg/dL)	TPROT (g/dL)	ALB (g/dL)
3001	89	0.3	175	57	218	6.7	3.4
3002	89	0.3	199	62	205	6.9	3.5
3003	90	0.5	323	64	146	7.4	3.8
3004	90	0.4	300	74	224	7.4	3.8
3005	90	0.4	298	67	318	7.5	3.9
3006	91	0.3	182	63	92	7.0	3.4
3007	91	0.4	326	60	139	7.2	3.6
3008	91	0.3	165	60	376	7.0	3.5
3009	92	0.5	237	60	248	6.9	3.6
3010	92	0.4	369	82	267	7.1	3.7

DRAFT, NOT FOR SUBMISSION

Appendix 15

Appendix 3

Individual Clinical Chemistry Values

32025165

Sex: Male

4.47x10^11 vg<br>Group 3	Day(s) Relative to Start Date	Reporting Biochemistry					
		GLOB (g/dL)	A/G (ratio)	CA (mg/dL)	PHOS (mg/dL)	NA (mEq/L)	K (mEq/L)
3001	89	3.3	1.0	11.6	9.8	148	7.1
3002	89	3.4	1.0	12.0	9.0	145	7.7
3003	90	3.6	1.1	12.8	10.2	142	10.2
3004	90	3.6	1.1	12.8	10.6	142	8.8
3005	90	3.6	1.1	12.6	11.0	143	8.9
3006	91	3.6	0.9	11.9	12.2	141	9.3
3007	91	3.6	1.0	12.9	11.8	145	7.2
3008	91	3.5	1.0	11.9	10.4	146	7.3
3009	92	3.3	1.1	11.6	8.5	146	7.7
3010	92	3.4	1.1	13.2	9.0	146	7.6

DRAFT, NOT FOR SUBMISSION

Appendix 15
Appendix 3

Individual Clinical Chemistry Values

32025165
Sex: Male

4.47x10^11 vg Group 3	Day(s) Relative to Start Date	Reporting Biochemistry CL (mEq/L)	HEMO INDICE	ICTERUS INDICE	LIPEMIA INDICE	SDH Alternate (U/L)
3001	89	102	N	N	N	6.8
3002	89	104	N	N	N	7.4
3003	90	98	N	N	N	12.8
3004	90	97	N	N	N	12.7
3005	90	96	+	N	N	34.0
3006	91	98	+	N	N	8.3
3007	91	99	+	N	N	15.3
3008	91	99	N	N	N	14.7
3009	92	100	+	N	N	45.4
3010	92	102	N	N	N	10.3

DRAFT, NOT FOR SUBMISSION

Appendix 15

Appendix 3

Individual Clinical Chemistry Values

32025165

Sex: Male

1.49x10^12 vg<br>Group 4	Day(s) Relative to Start Date	Reporting Biochemistry AST (U/L)	ALT (U/L)	ALP (U/L)	GGT (U/L)	TBIL (mg/dL)	UREAN (mg/dL)
4001	28	110	55	265	1 [a]	0.15	12
4002	28	118	79	174	1 [a]	0.14	14
4003	28	314	170	261	1 [a]	0.12	13
4004	28	95	69	230	1 [a]	0.15	10
4005	28	79	45	194	1 [a]	0.12	11
4006	89	100	56	129	1 [a]	0.13	13
4007	89	114	47	116	1 [a]	0.12	13
4008	90	51	35	92	1 [a]	0.13	13
4009	90	236	139	171	1 [a]	0.13	13
4010	91	74	53	124	1 [a]	0.15	13
4011	91	101	48	150	1 [a]	0.12	15
4012	91	141	44	123	1 [a]	0.13	10
4013	92	129	56	169	1 [a]	0.25	10
4014	92	62	39	109	1 [a]	0.15	11
4015	92	59	43	178	1 [a]	0.14	11

[a] [RC:Assigned value below the reportable range]

Appendix 15

Appendix 3

Individual Clinical Chemistry Values

32025165

Sex: Male

1.49x10^12 vg Group 4	Day(s) Relative to Start Date	Reporting Biochemistry CREAT (mg/dL)	GLUC (mg/dL)	CHOL (mg/dL)	TRIG (mg/dL)	TPROT (g/dL)	ALB (g/dL)
4001	28	0.4	388	95	193	7.3	3.8
4002	28	0.4	355	90	354	7.1	3.8
4003	28	0.4	451	91	218	6.8	3.6
4004	28	0.3	415	79	169	7.1	3.7
4005	28	0.4	618	124	211	7.2	3.7
4006	89	0.3	189	72	260	7.3	3.6
4007	89	0.3	268	75	146	7.2	3.7
4008	90	0.3	278	57	159	6.9	3.6
4009	90	0.3	184	51	31	7.6	3.3
4010	91	0.4	327	72	279	7.1	3.5
4011	91	0.4	483	62	265	7.0	3.4
4012	91	0.3	131	78	211	6.8	3.2
4013	92	0.3	136	59	53	6.1	3.1
4014	92	0.4	275	67	125	7.0	3.5
4015	92	0.4	406	59	142	7.2	3.6

DRAFT, NOT FOR SUBMISSION

Appendix 15

Appendix 3

Individual Clinical Chemistry Values

32025165

Sex: Male

1.49x10^12 vg Group 4	Day(s) Relative to Start Date	Reporting Biochemistry GLOB (g/dL)	A/G (ratio)	CA (mg/dL)	PHOS (mg/dL)	NA (mEq/L)	K (mEq/L)
4001	28	3.5	1.1	13.6	11.4	144	7.6
4002	28	3.3	1.2	14.3	12.0	149	7.3
4003	28	3.2	1.1	14.2	10.8	146	7.3
4004	28	3.4	1.1	13.3	11.0	146	7.7
4005	28	3.5	1.1	15.2	12.5	145	8.1
4006	89	3.7	1.0	12.6	10.3	145	7.5
4007	89	3.5	1.1	12.3	10.5	144	7.7
4008	90	3.3	1.1	12.4	10.7	144	9.0
4009	90	4.3	0.8	12.7	11.3	145	7.9
4010	91	3.6	1.0	12.5	10.6	143	6.5
4011	91	3.6	0.9	13.5	8.8	147	4.6
4012	91	3.6	0.9	11.3	8.5	148	5.1
4013	92	3.0	1.0	11.4	9.6	145	7.4
4014	92	3.5	1.0	12.4	11.1	144	8.2
4015	92	3.6	1.0	13.5	9.5	144	7.6

DRAFT, NOT FOR SUBMISSION

Appendix 15

Appendix 3

Individual Clinical Chemistry Values

32025165

Sex: Male

1.49x10^12 vg Group 4	Day(s) Relative to Start Date	Reporting Biochemistry CL (mEq/L)	HEMO INDICE	ICTERUS INDICE	LIPEMIA INDICE	SDH Alternate (U/L)
4001	28	98	N	N	N	19.1
4002	28	102	+	N	N	48.8
4003	28	100	N	N	N	79.8
4004	28	97	+	N	N	39.2
4005	28	97	N	N	N	16.6
4006	89	100	N	N	N	19.1
4007	89	102	N	N	N	12.1
4008	90	99	N	N	N	11.6
4009	90	98	N	N	N	113.6
4010	91	99	N	N	N	13.2
4011	91	99	N	N	N	39.5
4012	91	98	N	N	N	6.1
4013	92	102	N	N	N	6.8
4014	92	99	+	N	N	8.5
4015	92	98	N	N	N	5.8

DRAFT, NOT FOR SUBMISSION

Appendix 15

Appendix 3

Individual Clinical Chemistry Values

32025165

Sex: Female

0 vg		Reporting Biochemistry					
Group 1	Day(s) Relative to Start Date	AST (U/L)	ALT (U/L)	ALP (U/L)	GGT (U/L)	TBIL (mg/dL)	UREAN (mg/dL)
1501	29	64	33	106	1 [a]	0.13	16
1502	29	120	55	179	1 [a]	0.12	13
1503	29	128	70	245	1 [a]	0.15	13
1504	29	110	69	131	1 [a]	0.12	14
1505	29	105	63	168	1 [a]	0.14	13
1506	91	186	73	82	1 [a]	0.16	14
1507	91	89	49	97	1 [a]	0.16	16
1508	91	141	40	64	1 [a]	0.16	13
1509	92	88	53	77	1 [a]	0.16	14
1510	93	128	68	76	1 [a]	0.14	15

[a] [RC:Assigned value below the reportable range]

DRAFT, NOT FOR SUBMISSION

Appendix 15

Appendix 3

Individual Clinical Chemistry Values

32025165

Sex: Female

0 vg Group 1	Day(s) Relative to Start Date	Reporting Biochemistry CREAT (mg/dL)	GLUC (mg/dL)	CHOL (mg/dL)	TRIG (mg/dL)	TPROT (g/dL)	ALB (g/dL)
1501	29	0.4	253	109	52	8.1	4.6
1502	29	0.4	313	77	77	7.7	4.2
1503	29	0.4	262	55	92	7.9	4.3
1504	29	0.4	251	89	120	7.7	4.3
1505	29	0.3	308	64	65	7.3	4.2
1506	91	0.4	314	67	187	8.1	4.7
1507	91	0.4	222	89	193	8.3	4.9
1508	91	0.3	163	60	45	7.0	4.4
1509	92	0.4	222	57	122	7.9	4.4
1510	93	0.4	238	60	134	7.9	4.5

DRAFT, NOT FOR SUBMISSION

Appendix 15

Appendix 3

Individual Clinical Chemistry Values

32025165

Sex: Female

0 vg		Reporting Biochemistry					
Group 1	Day(s) Relative to Start Date	GLOB (g/dL)	A/G (ratio)	CA (mg/dL)	PHOS (mg/dL)	NA (mEq/L)	K (mEq/L)
1501	29	3.5	1.3	12.5	9.5	144	8.0
1502	29	3.5	1.2	13.1	11.4	145	9.0
1503	29	3.6	1.2	12.6	8.4	147	6.6
1504	29	3.4	1.3	13.0	11.3	150	6.8
1505	29	3.1	1.4	13.6	12.3	148	8.2
1506	91	3.4	1.4	14.0	8.3	145	7.9
1507	91	3.4	1.4	13.0	7.9	145	7.9
1508	91	2.6	1.7	11.7	6.4	144	8.3
1509	92	3.5	1.3	11.9	7.0	146	7.4
1510	93	3.4	1.3	12.5	8.1	146	7.5

DRAFT, NOT FOR SUBMISSION

Appendix 15

Appendix 3

Individual Clinical Chemistry Values

32025165

Sex: Female

0 vg		Reporting Biochemistry				
Group 1	Day(s) Relative to Start Date	CL (mEq/L)	HEMO INDICE	ICTERUS INDICE	LIPEMIA INDICE	SDH Alternate (U/L)
1501	29	103	N	N	N	5.3
1502	29	101	N	N	N	20.0
1503	29	104	N	N	N	15.5
1504	29	101	N	N	N	17.7
1505	29	101	N	N	N	14.9
1506	91	99	+	N	N	55.5
1507	91	102	+	N	N	12.1
1508	91	104	+	N	N	8.0
1509	92	102	+	N	N	17.7
1510	93	102	N	N	N	21.9

DRAFT, NOT FOR SUBMISSION

Appendix 15

Appendix 3

Individual Clinical Chemistry Values

32025165

Sex: Female

1.49x10^11 vg Group 2	Day(s) Relative to Start Date	Reporting Biochemistry AST (U/L)	ALT (U/L)	ALP (U/L)	GGT (U/L)	TBIL (mg/dL)	UREAN (mg/dL)
2501	91	179	83	90	1 [a]	0.15	22
2502	91	345	140	93	1 [a]	0.17	12
2503	91	116	67	101	1 [a]	0.14	14
2504	91	75	59	93	1 [a]	0.11	12
2505	91	118	78	153	1 [a]	0.13	13
2506	91	264	93	83	1 [a]	0.13	15
2507	92	93	63	80	1 [a]	0.18	15
2508	92	77	42	65	1 [a]	0.13	12
2509	92	143	80	187	1 [a]	0.16	17
2510	93	147	95	183	1 [a]	0.16	15

[a] [RC:Assigned value below the reportable range]

DRAFT, NOT FOR SUBMISSION

Appendix 15

Appendix 3

Individual Clinical Chemistry Values

32025165

Sex: Female

1.49x10^11 vg Group 2	Day(s) Relative to Start Date	Reporting Biochemistry CREAT (mg/dL)	GLUC (mg/dL)	CHOL (mg/dL)	TRIG (mg/dL)	TPROT (g/dL)	ALB (g/dL)
2501	91	0.3	244	85	107	7.9	4.7
2502	91	0.4	291	72	125	7.9	4.5
2503	91	0.4	258	61	98	7.4	4.3
2504	91	0.3	315	74	232	7.8	4.4
2505	91	0.4	239	60	130	8.1	4.7
2506	91	0.4	221	93	151	8.7	5.0
2507	92	0.4	208	81	142	8.3	4.6
2508	92	0.4	329	54	96	8.6	4.8
2509	92	0.4	321	60	247	7.7	4.3
2510	93	0.4	345	40	275	8.1	4.4

Appendix 15

Appendix 3

Individual Clinical Chemistry Values

32025165

Sex: Female

1.49x10^11 vg Group 2	Day(s) Relative to Start Date	Reporting Biochemistry GLOB (g/dL)	A/G (ratio)	CA (mg/dL)	PHOS (mg/dL)	NA (mEq/L)	K (mEq/L)
2501	91	3.2	1.5	12.5	9.4	145	7.8
2502	91	3.4	1.3	12.9	7.1	144	7.4
2503	91	3.1	1.4	12.8	8.4	144	7.9
2504	91	3.4	1.3	13.3	7.0	144	8.3
2505	91	3.4	1.4	12.9	7.5	142	8.0
2506	91	3.7	1.4	13.3	5.4	144	6.5
2507	92	3.7	1.2	12.5	6.4	142	7.8
2508	92	3.8	1.3	13.2	6.5	144	7.8
2509	92	3.4	1.3	13.2	8.0	142	9.8
2510	93	3.7	1.2	12.9	7.6	143	6.6

DRAFT, NOT FOR SUBMISSION

Appendix 15

Appendix 3

Individual Clinical Chemistry Values

32025165

Sex: Female

1.49x10^11 vg Group 2	Day(s) Relative to Start Date	Reporting Biochemistry				SDH Alternate (U/L)
		CL (mEq/L)	HEMO INDICE	ICTERUS INDICE	LIPEMIA INDICE	
2501	91	103	+	N	N	65.2
2502	91	103	N	N	N	84.6
2503	91	103	N	N	N	21.2
2504	91	101	+	N	N	13.0
2505	91	101	N	N	N	17.4
2506	91	97	N	N	N	52.7
2507	92	101	+	N	N	16.9
2508	92	102	N	N	N	18.8
2509	92	101	++	N	N	19.7
2510	93	100	+	N	N	36.8

DRAFT, NOT FOR SUBMISSION

Appendix 15

Appendix 3

Individual Clinical Chemistry Values

32025165

Sex: Female

4.47x10^11 vg Group 3	Day(s) Relative to Start Date	Reporting Biochemistry AST (U/L)	ALT (U/L)	ALP (U/L)	GGT (U/L)	TBIL (mg/dL)	UREAN (mg/dL)
3501	91	267	86	95	1 [a]	0.14	16
3502	91	56	43	92	1 [a]	0.13	16
3503	91	135	72	104	1 [a]	0.14	13
3504	91	117	59	71	1 [a]	0.16	11
3505	91	123	38	121	1 [a]	0.14	11
3506	91	134	62	102	1 [a]	0.11	10
3507	92	155	75	71	1 [a]	0.14	11
3508	92	92	46	62	1 [a]	0.15	14
3509	92	263	139	158	1 [a]	0.18	12
3510	93	135	75	62	1 [a]	0.15	12

[a] [RC:Assigned value below the reportable range]

DRAFT, NOT FOR SUBMISSION

Appendix 15

Appendix 3

Individual Clinical Chemistry Values

32025165

Sex: Female

4.47x10^11 vg<br>Group 3	Day(s) Relative to Start Date	Reporting Biochemistry					
		CREAT (mg/dL)	GLUC (mg/dL)	CHOL (mg/dL)	TRIG (mg/dL)	TPROT (g/dL)	ALB (g/dL)
3501	91	0.4	286	113	306	8.9	4.7
3502	91	0.4	274	62	137	7.8	4.4
3503	91	0.4	260	63	247	8.0	4.6
3504	91	0.4	293	64	97	7.7	4.6
3505	91	0.4	300	59	87	7.5	4.2
3506	91	0.3	400	73	105	7.5	4.2
3507	92	0.6	352	75	195	9.3	4.9
3508	92	0.4	158	69	126	8.8	4.9
3509	92	0.5	209	92	82	7.7	4.0
3510	93	0.3	169	61	76	7.9	4.2

DRAFT, NOT FOR SUBMISSION

Appendix 15

Appendix 3

Individual Clinical Chemistry Values

32025165

Sex: Female

4.47x10^11 vg Group 3	Day(s) Relative to Start Date	Reporting Biochemistry GLOB (g/dL)	A/G (ratio)	CA (mg/dL)	PHOS (mg/dL)	NA (mEq/L)	K (mEq/L)
3501	91	4.2	1.1	14.6	8.3	144	8.3
3502	91	3.4	1.3	13.2	7.5	144	8.0
3503	91	3.4	1.4	13.1	7.4	143	7.9
3504	91	3.1	1.5	13.7	9.2	146	6.5
3505	91	3.3	1.3	13.4	8.9	145	7.8
3506	91	3.3	1.3	13.1	9.3	143	8.2
3507	92	4.4	1.1	14.8	9.3	144	7.4
3508	92	3.9	1.3	12.6	6.7	143	8.3
3509	92	3.7	1.1	12.6	9.9	147	6.8
3510	93	3.7	1.1	12.9	9.0	146	7.2

DRAFT, NOT FOR SUBMISSION

Appendix 15

Appendix 3

Individual Clinical Chemistry Values

32025165

Sex: Female

4.47x10^11 vg Group 3	Day(s) Relative to Start Date	Reporting Biochemistry CL (mEq/L)	HEMO INDICE	ICTERUS INDICE	LIPEMIA INDICE	SDH Alternate (U/L)
3501	91	98	N	N	N	47.2
3502	91	100	N	N	N	6.4
3503	91	99	N	N	N	44.6
3504	91	100	N	N	N	14.3
3505	91	101	N	N	N	28.5
3506	91	98	N	N	N	51.8
3507	92	99	+	N	N	26.2
3508	92	101	+	N	N	12.2
3509	92	101	N	N	N	75.6
3510	93	103	N	N	N	22.4

DRAFT, NOT FOR SUBMISSION

Appendix 15

Appendix 3

Individual Clinical Chemistry Values

32025165

Sex: Female

1.49x10^12 vg Group 4	Day(s) Relative to Start Date	Reporting Biochemistry AST (U/L)	ALT (U/L)	ALP (U/L)	GGT (U/L)	TBIL (mg/dL)	UREAN (mg/dL)
4501	29	121	56	180	1 [a]	0.11	14
4502	29	84	56	116	1 [a]	0.12	14
4503	29	105	63	144	1 [a]	0.14	12
4504	29	76	38	162	1 [a]	0.14	12
4505	29	89	50	156	1 [a]	0.12	12
4506	91	79	43	63	1 [a]	0.17	12
4507	91	184	116	83	1 [a]	0.16	11
4508	91	121	60	73	1 [a]	0.10	13
4509	91	113	58	66	1 [a]	0.11	13
4510	91	116	51	45	1 [a]	0.17	9
4511	92	213	98	81	1 [a]	0.19	13
4512	92	59	34	43	1 [a]	0.17	11
4513	92	93	55	107	1 [a]	0.16	16
4514	93	112	73	66	1 [a]	0.13	12
4515	93	79	57	138	1 [a]	0.13	15

[a] [RC:Assigned value below the reportable range]

Appendix 15

Appendix 3

Individual Clinical Chemistry Values

32025165

Sex: Female

1.49x10^12 vg<br>Group 4	Day(s) Relative to Start Date	Reporting Biochemistry CREAT (mg/dL)	GLUC (mg/dL)	CHOL (mg/dL)	TRIG (mg/dL)	TPROT (g/dL)	ALB (g/dL)
4501	29	0.4	426	85	51	7.3	4.0
4502	29	0.3	292	100	129	8.1	4.3
4503	29	0.3	232	112	113	8.0	4.6
4504	29	0.3	306	102	50	7.2	3.9
4505	29	0.3	260	77	192	7.4	3.9
4506	91	0.4	345	94	149	8.4	4.8
4507	91	0.4	423	71	164	7.5	4.3
4508	91	0.4	283	60	287	8.0	4.7
4509	91	0.3	276	77	69	7.7	4.4
4510	91	0.4	252	85	253	8.1	4.5
4511	92	0.4	216	76	82	8.7	4.7
4512	92	0.3	212	70	107	8.3	4.6
4513	92	0.4	275	77	145	8.5	4.7
4514	93	0.5	260	68	201	8.1	4.4
4515	93	0.3	222	84	196	8.2	4.4

DRAFT, NOT FOR SUBMISSION

Appendix 15

Appendix 3

Individual Clinical Chemistry Values

32025165

Sex: Female

1.49x10^12 vg Group 4	Day(s) Relative to Start Date	Reporting Biochemistry GLOB (g/dL)	A/G (ratio)	CA (mg/dL)	PHOS (mg/dL)	NA (mEq/L)	K (mEq/L)
4501	29	3.3	1.2	13.2	12.0	144	9.3
4502	29	3.8	1.1	13.9	11.9	144	7.6
4503	29	3.4	1.4	13.0	8.9	147	5.6
4504	29	3.3	1.2	13.8	12.7	146	8.5
4505	29	3.5	1.1	13.0	9.7	145	6.8
4506	91	3.6	1.3	14.8	11.0	142	8.9
4507	91	3.2	1.3	13.8	8.1	146	6.6
4508	91	3.3	1.4	13.6	8.8	139	8.1
4509	91	3.3	1.3	13.2	9.6	146	6.8
4510	91	3.6	1.3	13.7	9.7	143	7.8
4511	92	4.0	1.2	13.0	8.5	143	8.2
4512	92	3.7	1.2	12.8	10.7	145	7.1
4513	92	3.8	1.2	13.4	8.7	141	9.0
4514	93	3.7	1.2	13.3	9.6	147	6.8
4515	93	3.8	1.2	13.5	7.8	145	7.6

DRAFT, NOT FOR SUBMISSION

Appendix 15

Appendix 3

Individual Clinical Chemistry Values

32025165

Sex: Female

1.49x10^12 vg Group 4	Day(s) Relative to Start Date	Reporting Biochemistry CL (mEq/L)	HEMO INDICE	ICTERUS INDICE	LIPEMIA INDICE	SDH Alternate (U/L)
4501	29	100	N	N	N	25.1
4502	29	100	N	N	N	8.7
4503	29	100	N	N	N	14.0
4504	29	102	N	N	N	7.0
4505	29	102	N	N	N	14.5
4506	91	97	N	N	N	9.6
4507	91	98	N	N	N	46.7
4508	91	98	N	N	N	84.8
4509	91	100	N	N	N	15.3
4510	91	98	N	N	N	13.1
4511	92	100	+	N	N	42.5
4512	92	100	N	N	N	9.2
4513	92	100	+	N	N	26.1
4514	93	102	+	N	N	27.9
4515	93	103	N	N	N	10.1

DRAFT, NOT FOR SUBMISSION

Appendix 16

Appendix 17

Appendix 18

Audited Draft Report

Study Phase: Pathology

Testing Facility Study No. 32025165

Sponsor Reference No. 32025165

GLP

TESTING FACILITY:
Charles River Laboratories
54943 North Main Street
Mattawan, MI 49071
United States

Appendix 18

TABLE OF CONTENTS

LIST OF TABLES 3
LIST OF APPENDICES 4
REPORT APPROVAL 5
1. RESPONSIBLE PERSONNEL 6
2. SUMMARY 7
3. MATERIALS AND METHODS 8
3.1. Group Assignments 8
3.2. Computerized Systems and Electronic Data Archive Locations 8
3.3. Retention and Disposition of Study Phase Materials 9
4. RESULTS AND DISCUSSIONS 9
4.1. Mortality 9
4.2. Macroscopic Pathology 9
4.2.1. Terminal Euthanasia Animals (Day 29 (±1) and Day 91 (±2)) 9
4.3. Organ Weights 9
4.3.1. Terminal Euthanasia Animals (Day 29 (±1) and Day 91 (±2)) 9
4.4. Microscopic Evaluations 9
4.4.1. Terminal Euthanasia Animals (Day 29 (±1)) 9
4.4.2. Terminal Euthanasia Animals (Day 91 (±2)) 10
5. REFERENCES 12

Appendix 18

LIST OF TABLES

Table 1 Summary of Macroscopic Pathology: Day 29 (±1) ... 14
Table 2 Summary of Macroscopic Pathology: Day 91 (±2) ... 20
Table 3 Summary of Organ Weights: Day 29 (±1) ... 27
Table 4 Summary of Organ Weights: Day 91 (±2) ... 35
Table 5 Summary of Microscopic Pathology: Day 29 (±1) ... 43
Table 6 Summary of Microscopic Pathology: Day 91 (±2) ... 46

DRAFT, NOT FOR SUBMISSION

LIST OF APPENDICES

Appendix 1 Individual Organ Weights: Day 29 (±1) 52
Appendix 2 Individual Organ Weights: Day 91 (±2) 76
Appendix 3 Individual Macroscopic and Microscopic Pathology 124
Appendix 4 Representative Images 214

REPORT APPROVAL

All electronic signatures appear at the end of the document upon finalization.

1. RESPONSIBLE PERSONNEL

Individual Scientist, Anatomic Pathologist	Jeremiah Lyons, MVB, PhD, DACVP<br>Charles River Laboratories
Testing Facility Management	Scott Fountain, PhD<br>Charles River Laboratories

2. SUMMARY

This study was conducted for the University of Massachusetts to determine the potential toxicity and biodistribution of scAAV9-CSA for the treatment of Cockayne Syndrome, when given as an intrathecal injection to rats once, and to evaluate the potential reversibility of any findings following a 28-day and 90-day recovery period.

There were no early deaths, or scAAV9-CSA-related macroscopic or organ weight changes at Day 29 (±1) or Day 91 (±2) necropsy timepoints.

scAAV9-CSA-related microscopic findings at Day 29 (±1) occurred in the lumbar dorsal root ganglia, sciatic nerve, and spinal cord at 1.49 x 10^{12} vg. scAAV9-CSA-related microscopic findings were solely those associated with AAV vector administration (Bennet et al., 2023; Bolon et al., 2024).

The lumbar dorsal root ganglia findings were characterized by minimal neuronal degeneration/necrosis and mononuclear infiltration as well as minimal to mild nerve fiber degeneration.

The sciatic nerve and dorsal roots of the lumbar spinal cord in scAAV9-CSA-treated animals also contained minimal to mild nerve fiber degeneration consistent with the lumbar dorsal root ganglia findings. One sciatic nerve also contained minimal mononuclear infiltration. Minimal nerve fiber degeneration of the dorsal funiculus of the lumbar spinal cord was present in two animals.

scAAV9-CSA-related microscopic findings at Day 91 (±2) were present in the dorsal root ganglia (lumbar, thoracic, and cervical), sciatic nerve, and spinal cord. All microscopic findings were consistent with expected findings associated with the use of AAV vectors (Bennet et al., 2023; Bolon et al., 2024).

Lumbar dorsal root ganglia findings considered related to the administration of scAAV9-CSA included minimal neuronal degeneration/necrosis and minimal to mild glial cell aggregates at ≥ 4.47 x 10^{11} vg. Minimal to moderate nerve fiber degeneration and minimal to mild mononuclear cell infiltration was present at increased incidence and severity in males at ≥ 4.47 x 10^{11} and females at ≥ 1.49 x 10^{11} vg. Neuronal degeneration/necrosis was not identified in animals administered the 1.49 x 10^{11} dose.

Thoracic dorsal root ganglia findings considered related to the administration of scAAV9-CSA included minimal to mild nerve fiber degeneration at 1.49 x 10^{12} vg in males and females and in one male at 1.49 x 10^{11} vg. Cervical dorsal root ganglia had one 4.47 x 10^{11} vg female with minimal nerve fiber degeneration considered related to scAAV9-CSA administration.

Sciatic nerve findings considered related to the administration of scAAV9-CSA included minimal nerve fiber degeneration at ≥ 1.49 x 10^{11} vg in males and females. Spinal cord findings considered related to the administration of scAAV9-CSA were present mostly in the lumbar and thoracic segments in the dorsal funiculus and dorsal nerve roots. These findings included minimal to mild nerve fiber degeneration at ≥ 4.47 x 10^{11} vg males and ≥ 1.49 x 10^{11} vg females.

Appendix 18

Neuronal and nerve fiber degeneration is a common class effect when using AAV vectors and at low severity its functional relevance is determined on an individual case basis (Bolon et al., 2024).

3. MATERIALS AND METHODS

3.1. Group Assignments

Experimental procedures applicable to pathology investigations are summarized in Text Table 1.

Text Table 1
Experimental Design

Group No.	Test Article	Dose Level (vg)	Dose Volume (mL)	Dose Concentration (vg/mL)	Main Study			
					Necropsy Day 29 (±1)		Necropsy Day 91 (±2)	
					No. of Males	No. of Females	No. of Males	No. of Females
1	Vehicle Control	0	0.1	0	5	5	5	5
2	scAAV9-CSA	1.49×10^{11}	0.1	1.49×10^{12}	-	-	10	10
3	scAAV9-CSA	4.47×10^{11}	0.1	4.47×10^{12}	-	-	10	10
4	scAAV9-CSA	1.49×10^{12}	0.1	1.49×10^{13}	5	5	10	10

- = Not applicable.

3.2. Computerized Systems and Electronic Data Archive Locations

Critical computerized systems used in the study phase and their respective electronic data archival locations are listed below. All computerized systems used in the conduct of this study have been validated; when a particular system has not satisfied all requirements, appropriate administrative and procedural controls were implemented to assure the quality and integrity of data.

Text Table 2
Critical Computerized Systems

System Name	System Description	Archive Location
Deviation Information Library	Provides a repository of deviations that allows for documenting, approving, and archiving all to be done electronically	Charles River Massachusetts Datacenter
DocuSign™ eSignature	Collection of 21 CFR Part 11 compliant signature	Charles River Massachusetts Datacenter
eInfotree™	Excel Module for data collection with 21 CFR Part 11 compliance requirements, security, audit trail, and electronic signatures	Charles River Massachusetts Datacenter, Testing Facility
M-Files®	Document management, publishing, and collection of 21 CFR Part 11 compliant signatures	Charles River Massachusetts Datacenter
Provantis®	Toxicology, clinical pathology, pathology, test material receipt, and formulation data collection, reporting, and statistics system	Charles River Massachusetts Datacenter

3.3. Retention and Disposition of Study Phase Materials

All study phase-specific physical raw data, documentation, and retained samples and/or specimens from this study phase are archived at the Testing Facility by no later than the date of Final Report issue.

Electronic data and documentation generated by this study phase are archived. Data generated by critical computerized systems are archived as listed in the critical computerized systems list. Electronic data archived in a cloud environment are under control of the Charles River Laboratories Archivist.

4. RESULTS AND DISCUSSIONS

Photomicrographs of representative microscopic lesions were taken and are included in Appendix 4.

4.1. Mortality

There were no unscheduled deaths prior to the Day 29 (±1) or Day 91 (±2) necropsy.

4.2. Macroscopic Pathology

4.2.1. Terminal Euthanasia Animals (Day 29 (±1) and Day 91 (±2))

(Table 1, Table 2, and Appendix 3)

There were no scAAV9-CSA-related macroscopic findings.

4.3. Organ Weights

4.3.1. Terminal Euthanasia Animals (Day 29 (±1) and Day 91 (±2))

(Table 3, Table 4, Appendix 1, and Appendix 2)

There were no scAAV9-CSA-related organ weight changes.

All differences in organ weight parameters were attributed to normal biologic variation. These differences had no patterns, trends, or correlating data to suggest these differences were scAAV9-CSA related.

4.4. Microscopic Evaluations

4.4.1. Terminal Euthanasia Animals (Day 29 (±1))

(Table 5 and Appendix 3)

scAAV9-CSA-related microscopic findings at 1.49 x 10^{12} vg occurred in the lumbar dorsal root ganglia, sciatic nerve, and spinal cord, and are summarized in Text Table 3.

scAAV9-CSA-related microscopic findings were solely those associated with AAV vector administration (Bennet et al., 2023; Bolon et al., 2024). The lumbar dorsal root ganglia findings were characterized by minimal neuronal degeneration/necrosis and mononuclear infiltration as well as minimal to mild nerve fiber degeneration. The neuronal degeneration/necrosis was

identified by eosinophilic cytoplasm often surrounded by mononuclear infiltrates. The nerve fiber degeneration involved axonal swelling, myelin vacuolation, and digestion chambers.

The sciatic nerve and dorsal roots of the lumbar spinal cord in scAAV9-CSA-treated animals also contained minimal to mild nerve fiber degeneration consistent with the lumbar dorsal root ganglia findings. One sciatic nerve also contained minimal mononuclear infiltration. Minimal nerve fiber degeneration of the dorsal funiculus of the lumbar spinal cord was present in two animals.

Text Table 3
Summary of scAAV9-CSA-related Microscopic Findings – Scheduled/Terminal Euthanasia (Day 29 (±1))

	**Males**		**Females**	
**Group**	**1**	**4**	**1**	**4**
**Dose (vg)**	**0**	**1.49×10^{12}**	**0**	**1.49×10^{12}**
**No. Animals Examined**	**5**	**5**	**5**	**5**
**Ganglion, dorsal root, lumbar (No. Examined)**	5	5	5	5
Infiltration, mononuclear cell	(1)	(2)	(1)	(5)
Minimal	1	1	1	4
Mild	0	1	0	1
Degeneration; nerve fiber	(0)	(2)	(0)	(2)
Minimal	0	1	0	0
Mild	0	1	0	2
Degeneration/necrosis; neuronal	(0)	(1)	(0)	(2)
Minimal	0	1	0	2
**Nerve, sciatic (No. Examined)**	5	5	5	5
Degeneration; nerve fiber	(0)	(2)	(0)	(4)
Minimal	0	2	0	3
Mild	0	0	0	1
Infiltration, mononuclear cell	(0)	(0)	(0)	(1)
Minimal	0	0	0	1
**Spinal cord (No. Examined)**	5	5	5	5
Degeneration; nerve fiber	(0)	(2)	(0)	(3)
Minimal	0	1	0	3
Mild	0	1	0	0

All other microscopic observations in scAAV9-CSA-treated animals were considered incidental and not test article related as these are known background observations for rats, were of low incidence and/or severity, they lacked a dose response relationship, or they were not similar amongst the sexes.

4.4.2. Terminal Euthanasia Animals (Day 91 (±2))

(Table 6 and Appendix 3)

Notable microscopic findings considered related to the administration of scAAV9-CSA at the Day 91 (±2) timepoint were present in the dorsal root ganglia (lumbar, thoracic, and cervical), sciatic nerve, and spinal cord. All microscopic findings were consistent with expected findings associated with the use of AAV vectors (Bennet et al., 2023; Bolon et al., 2024).

Appendix 18

The lumbar dorsal root ganglia findings at ≥ 4.47 x 10^{11} vg considered related to the administration of scAAV9-CSA included minimal neuronal degeneration/necrosis and minimal to mild glial cell aggregates. The neuronal degeneration/necrosis was identified by eosinophilic cytoplasm surrounded by mononuclear infiltrates. The glial cell aggregates are typical of a reparative process and indicate previous neuronal injury. Minimal to moderate nerve fiber degeneration and minimal to mild mononuclear infiltration were present at increased incidence and severity in ≥ 4.47 x 10^{11} vg males and in ≥ 1.49 x 10^{11} vg females. The nerve fiber degeneration involved axonal swelling, myelin vacuolation, and occasional digestion chambers. The finding of nerve fiber degeneration had a similar appearance at all the other sites (thoracic and cervical dorsal root ganglia, sciatic nerve, and spinal cord (see Text Table 4). The location of the nerve fiber degeneration was consistent with that expected with dorsal root ganglion injury using AAV vectors.

Text Table 4
Summary of scAAV9-CSA-related Microscopic Findings – Scheduled/Terminal Euthanasia (Day 91 (±2))

	**Males**				**Females**			
**Group**	**1**	**2**	**3**	**4**	**1**	**2**	**3**	**4**
**Dose (vg)**	**0**	**1.49 x 10^{11}**	**4.47 x 10^{11}**	**1.49 x 10^{12}**	**0**	**1.49 x 10^{11}**	**4.47 x 10^{11}**	**1.49 x 10^{12}**
**No. Animals Examined**	**5**	**10**	**10**	**10**	**5**	**10**	**10**	**10**
**Ganglion, dorsal root, lumbar (No. Examined)**	5	10	10	10	5	10	10	10
Infiltration, mononuclear cell	(2)	(2)	(6)	(7)	(0)	(5)	(4)	(8)
Minimal	2	2	5	5	0	5	3	5
Mild	0	0	1	2	0	0	1	3
Degeneration; nerve fiber	(1)	(1)	(6)	(10)	(0)	(3)	(4)	(10)
Minimal	1	1	5	5	0	3	2	6
Mild	0	0	1	4	0	0	2	2
Moderate	0	0	0	1	0	0	0	2
Degeneration/necrosis; neuronal	(0)	(0)	(1)	(2)	(0)	(0)	(1)	(2)
Minimal	0	0	1	2	0	0	1	2
Aggregate; glial cell	(0)	(0)	(3)	(8)	(0)	(0)	(2)	(8)
Minimal	0	0	3	7	0	0	1	6
Mild	0	0	0	1	0	0	1	2
**Ganglion, dorsal root, cervical (No. Examined)**	5	10	10	10	5	10	10	10
Infiltration, mononuclear cell	(0)	(0)	(0)	(1)	(0)	(0)	(1)	(0)
Minimal	0	0	0	1	0	0	1	0
Degeneration; nerve fiber	(0)	(0)	(0)	(0)	(0)	(0)	(1)	(0)
Minimal	0	0	0	0	0	0	1	0
**Ganglion, dorsal root, thoracic (No. Examined)**	5	10	10	10	5	10	10	10
Degeneration; nerve fiber	(0)	(1)	(0)	(3)	(0)	(0)	(0)	(1)
Minimal	0	1	0	3	0	0	0	0
Mild	0	0	0	0	0	0	0	1

Appendix 18

	Males				Females			
**Group**	**1**	**2**	**3**	**4**	**1**	**2**	**3**	**4**
**Dose (vg)**	**0**	**1.49 x 10^{11}**	**4.47 x 10^{11}**	**1.49 x 10^{12}**	**0**	**1.49 x 10^{11}**	**4.47 x 10^{11}**	**1.49 x 10^{12}**
**No. Animals Examined**	**5**	**10**	**10**	**10**	**5**	**10**	**10**	**10**
**Nerve, sciatic (No. Examined)**	5	10	10	10	5	10	10	10
Degeneration; nerve fiber	(0)	(4)	(5)	(8)	(0)	(2)	(5)	(9)
Minimal	0	4	5	8	0	2	5	9
**Spinal cord (No. Examined)**	5	10	10	10	5	10	10	10
Degeneration; nerve fiber	(1)	(2)	(3)	(7)	(0)	(2)	(6)	(9)
Minimal	0	2	3	5	0	2	4	2
Mild	1	0	0	2	0	0	2	7

In one 1.49 x 10^{12} vg male (Animal No. 4009), there was an unusual finding in the liver with hepatocellular vacuolation and hepatocytes containing eosinophilic globules. As this occurred in just one animal, it is not clearly associated with scAAV9-CSA administration and therefore was considered a background change unrelated to treatment.

5. REFERENCES

Bennet BM et al. Scientific and Regulatory Policy Committee Points to Consider: Sampling, Processing, Evaluation, Interpretation, and Reporting of Test Article-Related Ganglion Pathology for Nonclinical Toxicity Studies. Toxicologic Pathology 2023, Vol. 51(4) 176-204.

Bolon B et al. Neuropathological Findings in Nonclinical Species Following Administration of Adeno-Associated Virus (AAV)-Based Gene Therapy Vectors. Toxicologic Pathology 2024, Vol. 52(8) 498-505.

Appendix 18

EXPLANATION PAGE

All Day(s) referenced throughout the outputs generated are based upon Day 1 (first day of dose initiation).

Note: All abbreviations listed on this page may not be applicable to this report.

Abbreviation	Description
% Diff	% Difference from Group 1
tCtrl	Times control (fold change)
1F, 2F, 3F, 4F	Group 1 Female, Group 2 Female, Group 3 Female, Group 4 Female
1M, 2M, 3M, 4M	Group 1 Male, Group 2 Male, Group 3 Male, Group 4 Male
N	Number of values included in analysis
M, F	Male, Female
TK	Toxicokinetic
--	Not scheduled to be performed/dead
- or .	Not applicable

Abbreviation	Description	Abbreviation	Description
%br	% Organ Weight/Brain Weight	%bw	% Organ Weight/Terminal Bodyweight
COME	See comment value excluded	Sup	Suppress
E	Exclude	NA	Not applicable
No.	Animal number	Wt	Weight
C	clinical observations	G	gross observations
TGL	trackable gross lesions		

Appendix 18
Table 1

Summary of Macroscopic Pathology: Terminal Day 29±1

32025165

Removal Reason(s): TERMINAL EUTHANASIA<br>Summary: Incidence	Male<br>0 vg Group 1	Male<br>1.49x10^12 vg Group 4	Female<br>0 vg Group 1	Female<br>1.49x10^12 vg Group 4
Number of Animals:	5	5	5	5
**ARTERY, AORTA**				
Submitted	5	5	5	5
No Visible Lesions	5	5	5	5
**BODY CAVITY, NASAL**				
Submitted	5	5	5	5
No Visible Lesions	5	5	5	5
**BONE MARROW, STERNUM**				
Submitted	5	5	5	5
No Visible Lesions	5	5	5	5
**BONE, FEMUR**				
Submitted	5	5	5	5
No Visible Lesions	5	5	5	5
**BONE, STERNUM**				
Submitted	5	5	5	5
No Visible Lesions	5	5	5	5
**BRAIN**				
Submitted	5	5	5	5
No Visible Lesions	5	5	5	5
**CERVIX**				
Submitted	.	.	5	5
No Visible Lesions	.	.	5	5
**EPIDIDYMIS**				
Submitted	5	5	.	.
No Visible Lesions	5	5	.	.
**ESOPHAGUS**				
Submitted	5	5	5	5
No Visible Lesions	5	5	5	5
**EYE**				
Submitted	5	5	5	5
No Visible Lesions	5	5	5	5
**GALT**				
Submitted	5	5	5	5
No Visible Lesions	5	5	5	5
**GANGLION, DORSAL ROOT, CERVICAL**				
Submitted	5	5	5	5

Appendix 18
Table 1

Summary of Macroscopic Pathology: Terminal Day 29±1

32025165

Removal Reason(s): TERMINAL EUTHANASIA<br>Summary: Incidence	Male		Female	
	0 vg Group 1	1.49x10^12 vg Group 4	0 vg Group 1	1.49x10^12 vg Group 4
Number of Animals:	5	5	5	5
**GANGLION, DORSAL ROOT, CERVICAL (Continued...)**				
No Visible Lesions	5	5	5	5
**GANGLION, DORSAL ROOT, LUMBAR**				
Submitted	5	5	5	5
No Visible Lesions	5	5	5	5
**GANGLION, DORSAL ROOT, THORACIC**				
Submitted	5	5	5	5
No Visible Lesions	5	5	5	5
**GLAND, ADRENAL**				
Submitted	5	5	5	5
No Visible Lesions	5	5	5	5
**GLAND, CLITORAL**				
Submitted	.	.	5	5
No Visible Lesions	.	.	5	5
**GLAND, HARDERIAN**				
Submitted	5	5	5	5
No Visible Lesions	5	5	5	5
**GLAND, LACRIMAL**				
Submitted	5	5	5	5
No Visible Lesions	5	5	5	5
**GLAND, MAMMARY**				
Submitted	5	5	5	5
No Visible Lesions	5	5	5	5
**GLAND, PARATHYROID**				
Submitted	5	5	5	5
No Visible Lesions	5	5	5	5
**GLAND, PITUITARY**				
Submitted	5	5	5	5
No Visible Lesions	5	5	5	5
**GLAND, PREPUTIAL**				
Submitted	5	5	.	.
No Visible Lesions	5	5	.	.
**GLAND, PROSTATE**				
Submitted	5	5	.	.
No Visible Lesions	5	5	.	.

DRAFT, NOT FOR SUBMISSION

Appendix 18
Table 1

Summary of Macroscopic Pathology: Terminal Day 29±1

32025165

Removal Reason(s): TERMINAL EUTHANASIA<br>Summary: Incidence	Male 0 vg Group 1	Male 1.49x10^12 vg Group 4	Female 0 vg Group 1	Female 1.49x10^12 vg Group 4
Number of Animals:	5	5	5	5
**GLAND, SALIVARY, MANDIBULAR**				
Submitted	5	5	5	5
No Visible Lesions	5	5	5	5
**GLAND, SALIVARY, PAROTID**				
Submitted	5	5	5	5
No Visible Lesions	5	5	5	5
**GLAND, SALIVARY, SUBLINGUAL**				
Submitted	5	5	5	5
No Visible Lesions	5	5	5	5
**GLAND, SEMINAL VESICLE**				
Submitted	5	5	.	.
No Visible Lesions	5	5	.	.
**GLAND, THYROID**				
Submitted	5	5	5	5
No Visible Lesions	5	5	5	5
**GLAND, ZYMBALS**				
Submitted	5	5	5	5
No Visible Lesions	5	5	5	5
**HEART**				
Submitted	5	5	5	5
No Visible Lesions	5	5	5	5
**JOINT, FEMOROTIBIAL**				
Submitted	5	5	5	5
No Visible Lesions	5	5	5	5
**KIDNEY**				
Submitted	5	5	5	5
No Visible Lesions	5	5	5	5
**LARGE INTESTINE, CECUM**				
Submitted	5	5	5	5
No Visible Lesions	5	5	5	5
**LARGE INTESTINE, COLON**				
Submitted	5	5	5	5
No Visible Lesions	5	5	5	5
**LARGE INTESTINE, RECTUM**				
Submitted	5	5	5	5

Appendix 18
Table 1

Summary of Macroscopic Pathology: Terminal Day 29±1

32025165

Removal Reason(s): TERMINAL EUTHANASIA<br>Summary: Incidence	Male		Female	
	0 vg Group 1	1.49x10^12 vg Group 4	0 vg Group 1	1.49x10^12 vg Group 4
Number of Animals:	5	5	5	5
**LARGE INTESTINE, RECTUM (Continued...)**				
No Visible Lesions	5	5	5	5
**LARYNX**				
Submitted	5	5	5	5
No Visible Lesions	5	5	5	5
**LIVER**				
Submitted	5	5	5	5
No Visible Lesions	5	5	5	5
**LUNG**				
Submitted	5	5	5	5
No Visible Lesions	5	5	5	5
**LYMPH NODE, ILIAC, LEFT**				
Submitted	5	5	5	5
No Visible Lesions	5	5	5	5
**LYMPH NODE, ILIAC, RIGHT**				
Submitted	5	5	5	5
No Visible Lesions	5	5	5	5
**LYMPH NODE, MANDIBULAR, LEFT**				
Submitted	5	5	5	5
No Visible Lesions	5	5	5	5
**LYMPH NODE, MANDIBULAR, RIGHT**				
Submitted	5	5	5	5
No Visible Lesions	5	5	5	5
**LYMPH NODE, MESENTERIC**				
Submitted	5	5	5	5
No Visible Lesions	5	5	5	5
**MUSCLE, SKELETAL**				
Submitted	5	5	5	5
No Visible Lesions	5	5	5	5
**NERVE, OPTIC**				
Submitted	5	5	5	5
No Visible Lesions	5	5	5	5
**NERVE, SCIATIC**				
Submitted	5	5	5	5
No Visible Lesions	5	5	5	5

DRAFT, NOT FOR SUBMISSION

Appendix 18

Table 1

Summary of Macroscopic Pathology: Terminal Day 29±1

32025165

Removal Reason(s): TERMINAL EUTHANASIA<br>Summary: Incidence	Male		Female	
	0 vg Group 1	1.49x10^12 vg Group 4	0 vg Group 1	1.49x10^12 vg Group 4
Number of Animals:	5	5	5	5
**NERVE, TIBIAL**				
Submitted	5	5	5	5
No Visible Lesions	5	5	5	5
**OVARY**				
Submitted	.	.	5	5
No Visible Lesions	.	.	5	5
**OVIDUCT**				
Submitted	.	.	5	5
No Visible Lesions	.	.	5	5
**PANCREAS**				
Submitted	5	5	5	5
No Visible Lesions	5	5	5	5
**SKIN**				
Submitted	5	5	5	5
No Visible Lesions	5	5	5	5
**SMALL INTESTINE, DUODENUM**				
Submitted	5	5	5	5
No Visible Lesions	5	5	5	5
**SMALL INTESTINE, ILEUM**				
Submitted	5	5	5	5
No Visible Lesions	5	5	5	5
**SMALL INTESTINE, JEJUNUM**				
Submitted	5	5	5	5
No Visible Lesions	5	5	5	5
**SPINAL CORD, CERVICAL**				
Submitted	5	5	5	5
No Visible Lesions	5	5	5	5
**SPINAL CORD, LUMBAR**				
Submitted	5	5	5	5
No Visible Lesions	5	5	5	5
**SPINAL CORD, THORACIC**				
Submitted	5	5	5	5
No Visible Lesions	5	5	5	5
**SPLEEN**				
Submitted	5	5	5	5

DRAFT, NOT FOR SUBMISSION

Appendix 18
Table 1

Summary of Macroscopic Pathology: Terminal Day 29±1

32025165

Removal Reason(s): TERMINAL EUTHANASIA<br>Summary: Incidence	Male		Female	
	0 vg Group 1	1.49x10^12 vg Group 4	0 vg Group 1	1.49x10^12 vg Group 4
Number of Animals:	5	5	5	5
**SPLEEN (Continued...)**				
No Visible Lesions	5	5	5	5
**STOMACH**				
Submitted	5	5	5	5
No Visible Lesions	5	5	5	5
**TESTIS**				
Submitted	5	5	.	.
No Visible Lesions	5	5	.	.
**THYMUS**				
Submitted	5	5	5	5
No Visible Lesions	5	5	5	5
**TONGUE**				
Submitted	5	5	5	5
No Visible Lesions	5	5	5	5
**TRACHEA**				
Submitted	5	5	5	5
No Visible Lesions	5	5	5	5
**URETER**				
Submitted	5	5	5	5
No Visible Lesions	5	5	5	5
**URINARY BLADDER**				
Submitted	5	5	5	5
No Visible Lesions	5	5	5	5
**UTERUS**				
Submitted	.	.	5	5
No Visible Lesions	.	.	5	5
**VAGINA**				
Submitted	.	.	5	5
No Visible Lesions	.	.	5	5

Appendix 18
Table 2

Summary of Macroscopic Pathology: Terminal Day 91±2

32025165

Removal Reason(s): TERMINAL EUTHANASIA	Male				Female			
Summary: Incidence	0 vg Group 1	1.49x10^11 vg Group 2	4.47x10^11 vg Group 3	1.49x10^12 vg Group 4	0 vg Group 1	1.49x10^11 vg Group 2	4.47x10^11 vg Group 3	1.49x10^12 vg Group 4
Number of Animals:	5	10	10	10	5	10	10	10
**ARTERY, AORTA**								
Submitted	5	10	10	10	5	10	10	10
No Visible Lesions	5	10	10	10	5	10	10	10
**BODY CAVITY, NASAL**								
Submitted	5	10	10	10	5	10	10	10
No Visible Lesions	5	10	10	10	5	10	10	10
**BONE MARROW, STERNUM**								
Submitted	5	10	10	10	5	10	10	10
No Visible Lesions	5	10	10	10	5	10	10	10
**BONE, FEMUR**								
Submitted	5	10	10	10	5	10	10	10
No Visible Lesions	5	10	10	10	5	10	10	10
**BONE, STERNUM**								
Submitted	5	10	10	10	5	10	10	10
No Visible Lesions	5	10	10	10	5	10	10	10
**BRAIN**								
Submitted	5	10	10	10	5	10	10	10
No Visible Lesions	5	10	10	10	5	10	10	10
**CERVIX**								
Submitted	.	.	.	.	5	10	10	10
No Visible Lesions	.	.	.	.	5	10	10	10
**EPIDIDYMIS**								
Submitted	5	10	10	10	.	.	.	.
No Visible Lesions	5	10	10	10	.	.	.	.
**ESOPHAGUS**								
Submitted	5	10	10	10	5	10	10	10
No Visible Lesions	5	10	10	10	5	10	10	10
**EYE**								
Submitted	5	10	10	10	5	10	10	10
No Visible Lesions	5	10	10	10	5	10	10	10
**GALT**								
Submitted	5	10	10	10	5	10	10	10
No Visible Lesions	5	10	10	10	5	10	10	10

DRAFT, NOT FOR SUBMISSION

Appendix 18
Table 2

Summary of Macroscopic Pathology: Terminal Day 91±2

32025165

Removal Reason(s): TERMINAL EUTHANASIA	Male				Female			
Summary: Incidence	0 vg Group 1	1.49x10^11 vg Group 2	4.47x10^11 vg Group 3	1.49x10^12 vg Group 4	0 vg Group 1	1.49x10^11 vg Group 2	4.47x10^11 vg Group 3	1.49x10^12 vg Group 4
Number of Animals:	5	10	10	10	5	10	10	10
**GANGLION, DORSAL ROOT, CERVICAL**								
Submitted	5	10	10	10	5	10	10	10
No Visible Lesions	5	10	10	10	5	10	10	10
**GANGLION, DORSAL ROOT, LUMBAR**								
Submitted	5	10	10	10	5	10	10	10
No Visible Lesions	5	10	10	10	5	10	10	10
**GANGLION, DORSAL ROOT, THORACIC**								
Submitted	5	10	10	10	5	10	10	10
No Visible Lesions	5	10	10	10	5	10	10	10
**GLAND, ADRENAL**								
Submitted	5	10	10	10	5	10	10	10
No Visible Lesions	5	10	10	10	5	10	10	10
**GLAND, CLITORAL**								
Submitted	.	.	.	.	5	10	10	10
No Visible Lesions	.	.	.	.	5	10	10	10
**GLAND, HARDERIAN**								
Submitted	5	10	10	10	5	10	10	10
No Visible Lesions	5	10	10	10	5	10	10	10
**GLAND, LACRIMAL**								
Submitted	5	10	10	10	5	10	10	10
No Visible Lesions	5	10	10	10	5	10	10	10
**GLAND, MAMMARY**								
Submitted	5	10	10	10	5	10	10	10
No Visible Lesions	5	10	10	10	5	10	10	10
**GLAND, PARATHYROID**								
Submitted	5	10	10	10	5	10	10	10
No Visible Lesions	5	10	10	10	5	10	10	10
**GLAND, PITUITARY**								
Submitted	5	10	10	10	5	10	10	10
No Visible Lesions	5	10	10	10	5	10	10	10

DRAFT, NOT FOR SUBMISSION

Appendix 18
Table 2

Summary of Macroscopic Pathology: Terminal Day 91±2

32025165

Removal Reason(s): TERMINAL EUTHANASIA	Male				Female			
Summary: Incidence	0 vg Group 1	1.49x10^11 vg Group 2	4.47x10^11 vg Group 3	1.49x10^12 vg Group 4	0 vg Group 1	1.49x10^11 vg Group 2	4.47x10^11 vg Group 3	1.49x10^12 vg Group 4
Number of Animals:	5	10	10	10	5	10	10	10
**GLAND, PREPUTIAL**								
Submitted	5	10	10	10	.	.	.	.
No Visible Lesions	5	10	10	10	.	.	.	.
**GLAND, PROSTATE**								
Submitted	5	10	10	10	.	.	.	.
No Visible Lesions	5	10	10	10	.	.	.	.
**GLAND, SALIVARY, MANDIBULAR**								
Submitted	5	10	10	10	5	10	10	10
No Visible Lesions	5	10	10	10	5	10	10	10
**GLAND, SALIVARY, PAROTID**								
Submitted	5	10	10	10	5	10	10	10
No Visible Lesions	5	10	10	10	5	10	10	10
**GLAND, SALIVARY, SUBLINGUAL**								
Submitted	5	10	10	10	5	10	10	10
No Visible Lesions	5	10	10	10	5	10	10	10
**GLAND, SEMINAL VESICLE**								
Submitted	5	10	10	10	.	.	.	.
No Visible Lesions	5	10	10	10	.	.	.	.
**GLAND, THYROID**								
Submitted	5	10	10	10	5	10	10	10
No Visible Lesions	5	10	10	10	5	10	10	10
**GLAND, ZYMBALS**								
Submitted	5	10	10	10	5	10	10	10
No Visible Lesions	5	10	10	10	5	10	10	10
**HEART**								
Submitted	5	10	10	10	5	10	10	10
No Visible Lesions	5	10	10	10	5	10	10	10
**JOINT, FEMOROTIBIAL**								
Submitted	5	10	10	10	5	10	10	10
No Visible Lesions	5	10	10	10	5	10	10	10
**KIDNEY**								
Submitted	5	10	10	10	5	10	10	10

DRAFT, NOT FOR SUBMISSION

Appendix 18

Table 2

Summary of Macroscopic Pathology: Terminal Day 91±2

32025165

Removal Reason(s): TERMINAL EUTHANASIA	Male				Female			
Summary: Incidence	0 vg Group 1	1.49x10^11 vg Group 2	4.47x10^11 vg Group 3	1.49x10^12 vg Group 4	0 vg Group 1	1.49x10^11 vg Group 2	4.47x10^11 vg Group 3	1.49x10^12 vg Group 4
Number of Animals:	5	10	10	10	5	10	10	10
**KIDNEY (Continued...)**								
No Visible Lesions	5	10	9	10	5	10	10	10
Cyst, clear	0	0	1	0	0	0	0	0
**LARGE INTESTINE, CECUM**								
Submitted	5	10	10	10	5	10	10	10
No Visible Lesions	5	10	10	10	5	10	10	10
**LARGE INTESTINE, COLON**								
Submitted	5	10	10	10	5	10	10	10
No Visible Lesions	5	10	10	10	5	10	10	10
**LARGE INTESTINE, RECTUM**								
Submitted	5	10	10	10	5	10	10	10
No Visible Lesions	5	10	10	10	5	10	10	10
**LARYNX**								
Submitted	5	10	10	10	5	10	10	10
No Visible Lesions	5	10	10	10	5	10	10	10
**LIVER**								
Submitted	5	10	10	10	5	10	10	10
No Visible Lesions	5	10	10	9	5	10	10	10
Nodule	0	0	0	1	0	0	0	0
**LUNG**								
Submitted	5	10	10	10	5	10	10	10
No Visible Lesions	5	10	10	10	5	10	10	10
**LYMPH NODE, ILIAC, LEFT**								
Submitted	5	10	10	10	5	10	10	10
No Visible Lesions	5	10	10	10	5	10	10	10
**LYMPH NODE, ILIAC, RIGHT**								
Submitted	5	10	10	10	5	10	10	10
No Visible Lesions	5	10	10	10	5	10	10	10
**LYMPH NODE, MANDIBULAR, LEFT**								
Submitted	5	10	10	10	5	10	10	10
No Visible Lesions	5	10	10	10	5	10	10	10
**LYMPH NODE, MANDIBULAR, RIGHT**								

DRAFT, NOT FOR SUBMISSION

Appendix 18
Table 2

Summary of Macroscopic Pathology: Terminal Day 91±2

32025165

Removal Reason(s): TERMINAL EUTHANASIA	Male				Female			
Summary: Incidence	0 vg Group 1	1.49x10^11 vg Group 2	4.47x10^11 vg Group 3	1.49x10^12 vg Group 4	0 vg Group 1	1.49x10^11 vg Group 2	4.47x10^11 vg Group 3	1.49x10^12 vg Group 4
Number of Animals:	5	10	10	10	5	10	10	10
**LYMPH NODE, MANDIBULAR, RIGHT (Continued...)**								
Submitted	5	10	10	10	5	10	10	10
No Visible Lesions	5	10	10	10	5	10	10	10
**LYMPH NODE, MESENTERIC**								
Submitted	5	10	10	10	5	10	10	10
No Visible Lesions	5	10	10	10	5	10	10	10
**MUSCLE, DIAPHRAGM**								
Submitted	0	0	0	1	.	.	.	.
Hernia	.	.	.	1	.	.	.	.
**MUSCLE, SKELETAL**								
Submitted	5	10	10	10	5	10	10	10
No Visible Lesions	5	10	10	10	5	10	10	10
**NERVE, OPTIC**								
Submitted	5	10	10	10	5	10	10	10
No Visible Lesions	5	10	10	10	5	10	10	10
**NERVE, SCIATIC**								
Submitted	5	10	10	10	5	10	10	10
No Visible Lesions	5	10	10	10	5	10	10	10
**NERVE, TIBIAL**								
Submitted	5	10	10	10	5	10	10	10
No Visible Lesions	5	10	10	10	5	10	10	10
**OVARY**								
Submitted	.	.	.	.	5	10	10	10
No Visible Lesions	.	.	.	.	4	10	10	10
Cyst, clear	.	.	.	.	1	0	0	0
**OVIDUCT**								
Submitted	.	.	.	.	5	10	10	10
No Visible Lesions	.	.	.	.	5	10	10	10
**PANCREAS**								
Submitted	5	10	10	10	5	10	10	10
No Visible Lesions	5	10	10	10	5	10	10	10

Appendix 18

Table 2

Summary of Macroscopic Pathology: Terminal Day 91±2

32025165

Removal Reason(s): TERMINAL EUTHANASIA	Male				Female			
Summary: Incidence	0 vg Group 1	1.49x10^11 vg Group 2	4.47x10^11 vg Group 3	1.49x10^12 vg Group 4	0 vg Group 1	1.49x10^11 vg Group 2	4.47x10^11 vg Group 3	1.49x10^12 vg Group 4
Number of Animals:	5	10	10	10	5	10	10	10
**SKIN**								
Submitted	5	10	10	10	5	10	10	10
No Visible Lesions	5	10	10	10	5	10	10	10
**SMALL INTESTINE, DUODENUM**								
Submitted	5	10	10	10	5	10	10	10
No Visible Lesions	5	10	10	10	5	10	10	10
**SMALL INTESTINE, ILEUM**								
Submitted	5	10	10	10	5	10	10	10
No Visible Lesions	5	10	10	10	5	10	10	10
**SMALL INTESTINE, JEJUNUM**								
Submitted	5	10	10	10	5	10	10	10
No Visible Lesions	5	10	10	10	5	10	10	10
**SPINAL CORD, CERVICAL**								
Submitted	5	10	10	10	5	10	10	10
No Visible Lesions	5	10	10	10	5	10	10	10
**SPINAL CORD, LUMBAR**								
Submitted	5	10	10	10	5	10	10	10
No Visible Lesions	5	10	10	10	5	10	10	10
**SPINAL CORD, THORACIC**								
Submitted	5	10	10	10	5	10	10	10
No Visible Lesions	5	10	10	10	5	10	10	10
**SPLEEN**								
Submitted	5	10	10	10	5	10	10	10
No Visible Lesions	5	10	10	10	5	10	10	10
**STOMACH**								
Submitted	5	10	10	10	5	10	10	10
No Visible Lesions	5	10	10	10	5	10	10	10
**TESTIS**								
Submitted	5	10	10	10	.	.	.	.
No Visible Lesions	5	10	10	10	.	.	.	.
**THYMUS**								
Submitted	5	10	10	10	5	10	10	10

DRAFT, NOT FOR SUBMISSION

Appendix 18

Table 2

Summary of Macroscopic Pathology: Terminal Day 91±2

32025165

Removal Reason(s): TERMINAL EUTHANASIA	Male				Female			
Summary: Incidence	0 vg Group 1	1.49x10^11 vg Group 2	4.47x10^11 vg Group 3	1.49x10^12 vg Group 4	0 vg Group 1	1.49x10^11 vg Group 2	4.47x10^11 vg Group 3	1.49x10^12 vg Group 4
Number of Animals:	5	10	10	10	5	10	10	10
**THYMUS (Continued...)**								
No Visible Lesions	5	10	10	10	5	10	9	10
Discoloration, dark	0	0	0	0	0	0	1	0
**TONGUE**								
Submitted	5	10	10	10	5	10	10	10
No Visible Lesions	5	10	10	10	5	10	10	10
**TRACHEA**								
Submitted	5	10	10	10	5	10	10	10
No Visible Lesions	5	10	10	10	5	10	10	10
**URETER**								
Submitted	5	10	10	10	5	10	10	10
No Visible Lesions	5	10	10	10	5	10	10	10
**URINARY BLADDER**								
Submitted	5	10	10	10	5	10	10	10
No Visible Lesions	5	10	10	10	5	10	10	10
**UTERUS**								
Submitted	.	.	.	.	5	10	10	10
No Visible Lesions	.	.	.	.	5	9	10	10
Cyst, clear	.	.	.	.	0	1	0	0
**VAGINA**								
Submitted	.	.	.	.	5	10	10	10
No Visible Lesions	.	.	.	.	5	10	10	10

DRAFT, NOT FOR SUBMISSION

Appendix 18
Table 3

Summary of Organ Weights: Terminal Day 29±1

32025165

Sex: Male<br>Day(s) Relative to Start Date		0 vg<br>Group 1	1.49x10^12 vg<br>Group 4
Terminal Body Weight (g)	Mean	389.4144	449.8924
	SD	41.2920	48.2029
	N	5	5
	%Diff	-	15.5305
Brain (g)	Mean	2.0924	2.0838
	SD	0.1224	0.0272
	N	5	5
	%Diff	-	-0.4110
Brain (%bw)	Mean	0.54193	0.46762
	SD	0.06142	0.05204
	N	5	5
	%Diff	-	-13.71254
Epididymis Weight (g)	Mean	1.1630	1.3272
	SD	0.0504	0.0893
	N	5	5
	%Diff	-	14.1187
Epididymis (%br)	Mean	55.81485	63.69321
	SD	5.13450	4.27137
	N	5	5
	%Diff	-	14.11516
Epididymis (%bw)	Mean	0.30121	0.29759
	SD	0.03337	0.03483
	N	5	5
	%Diff	-	-1.20199
Adrenal (g)	Mean	0.05560	0.05560
	SD	0.00619	0.00913
	N	5	5
	%Diff	-	0.00000
Adrenal (%br)	Mean	2.67149	2.66763
	SD	0.40213	0.43338
	N	5	5
	%Diff	-	-0.14424
Adrenal (%bw)	Mean	0.01437	0.01253
	SD	0.00187	0.00294
	N	5	5
	%Diff	-	-12.76556

DRAFT, NOT FOR SUBMISSION

Appendix 18
Table 3

Summary of Organ Weights: Terminal Day 29±1

32025165

Sex: Male<br>Day(s) Relative to Start Date		0 vg<br>Group 1	1.49x10^12 vg<br>Group 4
Pituitary (g)	Mean	0.01666	0.01490
	SD	0.00085	0.00182
	N	5	5
	%Diff	-	-10.56423
Pituitary (%br)	Mean	0.79892	0.71572
	SD	0.06786	0.09376
	N	5	5
	%Diff	-	-10.41395
Pituitary (%bw)	Mean	0.00431	0.00332
	SD	0.00048	0.00025
	N	5	5
	%Diff	-	-23.16143
Prostate (g)	Mean	0.9690	1.1186
	SD	0.0980	0.1934
	N	5	5
	%Diff	-	15.4386
Prostate (%br)	Mean	46.52783	53.71171
	SD	6.55135	9.54412
	N	5	5
	%Diff	-	15.43997
Prostate (%bw)	Mean	0.25297	0.24857
	SD	0.05219	0.03176
	N	5	5
	%Diff	-	-1.73859
Thyroid/Parathyroid (g)	Mean	0.02568	0.02836
	SD	0.00312	0.00570
	N	5	5
	%Diff	-	10.43614
Thyroid/Parathyroid (%br)	Mean	1.23489	1.35898
	SD	0.19368	0.25646
	N	5	5
	%Diff	-	10.04863
Thyroid/Parathyroid (%bw)	Mean	0.00668	0.00635
	SD	0.00128	0.00141
	N	5	5
	%Diff	-	-4.92102

DRAFT, NOT FOR SUBMISSION

Appendix 18

Table 3

Summary of Organ Weights: Terminal Day 29±1

32025165

Sex: Male<br>Day(s) Relative to Start Date		0 vg<br>Group 1	1.49x10^12 vg<br>Group 4
Heart Weight (g)	Mean	1.3980	1.5312
	SD	0.0874	0.1157
	N	5	5
	%Diff	-	9.5279
Heart (%br)	Mean	67.09843	73.48872
	SD	7.10145	5.61373
	N	5	5
	%Diff	-	9.52376
Heart (%bw)	Mean	0.36162	0.34182
	SD	0.03772	0.02366
	N	5	5
	%Diff	-	-5.47514
Kidney Weight (g)	Mean	2.9940	3.1536
	SD	0.1910	0.2320
	N	5	5
	%Diff	-	5.3307
Kidney (%br)	Mean	143.74569	151.44804
	SD	15.73667	12.65103
	N	5	5
	%Diff	-	5.35832
Kidney (%bw)	Mean	0.77387	0.70564
	SD	0.07594	0.07401
	N	5	5
	%Diff	-	-8.81711
Liver Weight (g)	Mean	15.5816	18.2034
	SD	2.1946	1.2540
	N	5	5
	%Diff	-	16.8263
Liver (%br)	Mean	750.08208	873.76838
	SD	137.82549	62.94961
	N	5	5
	%Diff	-	16.48970
Liver (%bw)	Mean	4.01308	4.06295
	SD	0.49865	0.24539
	N	5	5
	%Diff	-	1.24277

DRAFT, NOT FOR SUBMISSION

Appendix 18

Table 3

Summary of Organ Weights: Terminal Day 29±1

32025165

Sex: Male<br>Day(s) Relative to Start Date		0 vg<br>Group 1	1.49x10^12 vg<br>Group 4
Spleen (g)	Mean	0.6822	0.8386
	SD	0.1575	0.1390
	N	5	5
	%Diff	-	22.9258
Spleen (%br)	Mean	32.87556	40.25908
	SD	8.55467	6.79614
	N	5	5
	%Diff	-	22.45901
Spleen (%bw)	Mean	0.17426	0.18727
	SD	0.02817	0.03085
	N	5	5
	%Diff	-	7.46358
Testis Weight (g)	Mean	3.3160	3.7762
	SD	0.4204	0.2307
	N	5	5
	%Diff	-	13.8782
Testis (%br)	Mean	159.38813	181.32341
	SD	24.98131	12.89405
	N	5	5
	%Diff	-	13.76218
Testis (%bw)	Mean	0.85643	0.84366
	SD	0.11998	0.05889
	N	5	5
	%Diff	-	-1.49065
Thymus Weight (g)	Mean	0.5410	0.5130
	SD	0.1203	0.1217
	N	5	5
	%Diff	-	-5.1756
Thymus (%br)	Mean	26.11842	24.58616
	SD	6.87435	5.62576
	N	5	5
	%Diff	-	-5.86659
Thymus (%bw)	Mean	0.13898	0.11457
	SD	0.02805	0.02774
	N	5	5
	%Diff	-	-17.56722

DRAFT, NOT FOR SUBMISSION

Appendix 18
Table 3

Summary of Organ Weights: Terminal Day 29±1

32025165

Sex: Female<br>Day(s) Relative to Start Date		0 vg<br>Group 1	1.49x10^12 vg<br>Group 4
Terminal Body Weight (g)	Mean	236.7406	245.1306
	SD	24.2587	18.3883
	N	5	5
	%Diff	-	3.5440
Brain (g)	Mean	1.9204	2.0162
	SD	0.1337	0.0889
	N	5	5
	%Diff	-	4.9885
Brain (%bw)	Mean	0.81635	0.82460
	SD	0.08469	0.04224
	N	5	5
	%Diff	-	1.01001
Adrenal (g)	Mean	0.06380	0.06880
	SD	0.00746	0.00817
	N	5	5
	%Diff	-	7.83699
Adrenal (%br)	Mean	3.34190	3.42812
	SD	0.52911	0.52331
	N	5	5
	%Diff	-	2.58003
Adrenal (%bw)	Mean	0.02729	0.02833
	SD	0.00496	0.00504
	N	5	5
	%Diff	-	3.81953
Pituitary (g)	Mean	0.01796	0.01902
	SD	0.00295	0.00243
	N	5	5
	%Diff	-	5.90200
Pituitary (%br)	Mean	0.93898	0.94629
	SD	0.17207	0.14355
	N	5	5
	%Diff	-	0.77807
Pituitary (%bw)	Mean	0.00766	0.00784
	SD	0.00147	0.00146
	N	5	5
	%Diff	-	2.35526

DRAFT, NOT FOR SUBMISSION

Appendix 18
Table 3

Summary of Organ Weights: Terminal Day 29±1

32025165

Sex: Female<br>Day(s) Relative to Start Date		0 vg<br>Group 1	1.49x10^12 vg<br>Group 4
Thyroid/Parathyroid (g)	Mean	0.02346	0.02826
	SD	0.00205	0.00429
	N	5	5
	%Diff	-	20.46036
Thyroid/Parathyroid (%br)	Mean	1.22380	1.39863
	SD	0.09926	0.16727
	N	5	5
	%Diff	-	14.28590
Thyroid/Parathyroid (%bw)	Mean	0.00994	0.01153
	SD	0.00069	0.00149
	N	5	5
	%Diff	-	16.01569
Heart Weight (g)	Mean	0.9372	0.9660
	SD	0.1064	0.0448
	N	5	5
	%Diff	-	3.0730
Heart (%br)	Mean	48.85264	47.95759
	SD	5.32772	2.48588
	N	5	5
	%Diff	-	-1.83214
Heart (%bw)	Mean	0.39691	0.39538
	SD	0.04090	0.02776
	N	5	5
	%Diff	-	-0.38754
Kidney Weight (g)	Mean	1.8464	2.0314
	SD	0.2004	0.2414
	N	5	5
	%Diff	-	10.0195
Kidney (%br)	Mean	96.45023	100.56452
	SD	11.58718	8.76313
	N	5	5
	%Diff	-	4.26572
Kidney (%bw)	Mean	0.78146	0.82956
	SD	0.06025	0.08761
	N	5	5
	%Diff	-	6.15638

DRAFT, NOT FOR SUBMISSION

Appendix 18
Table 3

Summary of Organ Weights: Terminal Day 29±1

32025165

Sex: Female<br>Day(s) Relative to Start Date		0 vg<br>Group 1	1.49x10^12 vg<br>Group 4
Liver Weight (g)	Mean	9.0750	9.3232
	SD	1.4364	0.8311
	N	5	5
	%Diff	-	2.7350
Liver (%br)	Mean	472.81605	462.19055
	SD	65.67654	32.74388
	N	5	5
	%Diff	-	-2.24728
Liver (%bw)	Mean	3.82308	3.80831
	SD	0.28888	0.29625
	N	5	5
	%Diff	-	-0.38647
Ovary Paired (g)	Mean	0.0940	0.0880
	SD	0.0104	0.0135
	N	5	5
	%Diff	-	-6.3830
Ovary Paired (%br)	Mean	4.93758	4.38850
	SD	0.81872	0.80596
	N	5	5
	%Diff	-	-11.12051
Ovary Paired (%bw)	Mean	0.03995	0.03625
	SD	0.00530	0.00737
	N	5	5
	%Diff	-	-9.26120
Spleen (g)	Mean	0.4942	0.5630
	SD	0.0280	0.0142
	N	5	5
	%Diff	-	13.9215
Spleen (%br)	Mean	25.79504	27.94808
	SD	1.70438	0.83822
	N	5	5
	%Diff	-	8.34674
Spleen (%bw)	Mean	0.20966	0.23063
	SD	0.01288	0.01686
	N	5	5
	%Diff	-	10.00180

DRAFT, NOT FOR SUBMISSION

Appendix 18

Table 3

Summary of Organ Weights: Terminal Day 29±1

32025165

Sex: Female<br>Day(s) Relative to Start Date		0 vg<br>Group 1	1.49x10^12 vg<br>Group 4
Thymus Weight (g)	Mean	0.3908	0.4306
	SD	0.0586	0.0665
	N	5	5
	%Diff	-	10.1842
Thymus (%br)	Mean	20.46009	21.37221
	SD	3.40924	3.32286
	N	5	5
	%Diff	-	4.45803
Thymus (%bw)	Mean	0.16485	0.17567
	SD	0.01572	0.02440
	N	5	5
	%Diff	-	6.56190
Uterus/Cervix (g)	Mean	0.6982	0.7236
	SD	0.2503	0.2978
	N	5	5
	%Diff	-	3.6379
Uterus/Cervix (%br)	Mean	36.97493	36.01461
	SD	15.20040	15.01613
	N	5	5
	%Diff	-	-2.59720
Uterus/Cervix (%bw)	Mean	0.30363	0.30051
	SD	0.13603	0.13712
	N	5	5
	%Diff	-	-1.02605

DRAFT, NOT FOR SUBMISSION

Appendix 18

Table 4

Summary of Organ Weights: Terminal Day 91±2

32025165

Sex: Male<br>Day(s) Relative to Start Date		0 vg<br>Group 1	1.49x10^11 vg<br>Group 2	4.47x10^11 vg<br>Group 3	1.49x10^12 vg<br>Group 4
Terminal Body Weight (g)	Mean	563.8794	600.1980	585.7299	590.0850
	SD	82.5196	103.1047	60.7826	76.3519
	N	5	10	10	10
	%Diff	-	6.4408	3.8750	4.6474
Brain (g)	Mean	2.2612	2.2260	2.2615	2.2542
	SD	0.0807	0.1217	0.1048	0.1074
	N	5	10	10	10
	%Diff	-	-1.5567	0.0133	-0.3096
Brain (%bw)	Mean	0.40669	0.37995	0.38929	0.38588
	SD	0.05104	0.06262	0.03822	0.03395
	N	5	10	10	10
	%Diff	-	-6.57496	-4.27824	-5.11794
Epididymis Weight (g)	Mean	1.5864	1.6100	1.6162	1.6057
	SD	0.0663	0.1836	0.1635	0.2214
	N	5	10	10	10
	%Diff	-	1.4876	1.8785	1.2166
Epididymis (%br)	Mean	70.28772	72.57570	71.50849	71.09513
	SD	5.08782	9.88027	6.69272	8.18138
	N	5	10	10	10
	%Diff	-	3.25517	1.73683	1.14872
Epididymis (%bw)	Mean	0.28682	0.27445	0.27704	0.27344
	SD	0.04850	0.05274	0.02495	0.03320
	N	5	10	10	10
	%Diff	-	-4.31261	-3.40951	-4.66297
Adrenal (g)	Mean	0.06940	0.05180	0.05120	0.05490
	SD	0.01341	0.01153	0.00758	0.01301
	N	5	10	10	10
	%Diff	-	-25.36023	-26.22478	-20.89337
Adrenal (%br)	Mean	3.08168	2.31470	2.26483	2.42394
	SD	0.67470	0.42592	0.32763	0.52060
	N	5	10	10	10
	%Diff	-	-24.88848	-26.50653	-21.34355
Adrenal (%bw)	Mean	0.01268	0.00874	0.00879	0.00929
	SD	0.00383	0.00195	0.00143	0.00188
	N	5	10	10	10
	%Diff	-	-31.12662	-30.66521	-26.79111

DRAFT, NOT FOR SUBMISSION

Appendix 18
Table 4

Summary of Organ Weights: Terminal Day 91±2

32025165

Sex: Male<br>Day(s) Relative to Start Date		0 vg<br>Group 1	1.49x10^11 vg<br>Group 2	4.47x10^11 vg<br>Group 3	1.49x10^12 vg<br>Group 4
Pituitary (g)	Mean	0.01738	0.01593	0.01566	0.01613
	SD	0.00218	0.00159	0.00148	0.00150
	N	5	10	9	10
	%Diff	-	-8.34292	-9.92200	-7.19217
Pituitary (%br)	Mean	0.76711	0.71695	0.69112	0.71472
	SD	0.07197	0.07647	0.05946	0.04380
	N	5	10	9	10
	%Diff	-	-6.53924	-9.90692	-6.82941
Pituitary (%bw)	Mean	0.00310	0.00270	0.00265	0.00275
	SD	0.00023	0.00036	0.00019	0.00026
	N	5	10	9	10
	%Diff	-	-12.87697	-14.35165	-11.04792
Prostate (g)	Mean	1.5248	1.5211	1.5366	1.4274
	SD	0.3623	0.2257	0.3217	0.2613
	N	5	10	10	10
	%Diff	-	-0.2427	0.7739	-6.3877
Prostate (%br)	Mean	67.60148	68.49829	68.01718	63.04687
	SD	16.70155	10.75066	14.42217	9.48554
	N	5	10	10	10
	%Diff	-	1.32661	0.61494	-6.73743
Prostate (%bw)	Mean	0.27877	0.25701	0.26411	0.24120
	SD	0.09267	0.03795	0.05978	0.02644
	N	5	10	10	10
	%Diff	-	-7.80534	-5.25787	-13.47766
Thyroid/Parathyroid (g)	Mean	0.02872	0.02966	0.02802	0.02986
	SD	0.00487	0.00542	0.00782	0.00345
	N	5	10	10	10
	%Diff	-	3.27298	-2.43733	3.96936
Thyroid/Parathyroid (%br)	Mean	1.27154	1.33541	1.24183	1.32584
	SD	0.21951	0.25031	0.35832	0.15125
	N	5	10	10	10
	%Diff	-	5.02342	-2.33661	4.27062
Thyroid/Parathyroid (%bw)	Mean	0.00515	0.00503	0.00480	0.00512
	SD	0.00092	0.00095	0.00135	0.00079
	N	5	10	10	10
	%Diff	-	-2.34725	-6.72087	-0.50800

DRAFT, NOT FOR SUBMISSION

Appendix 18
Table 4

Summary of Organ Weights: Terminal Day 91±2

32025165

Sex: Male<br>Day(s) Relative to Start Date		0 vg<br>Group 1	1.49x10^11 vg<br>Group 2	4.47x10^11 vg<br>Group 3	1.49x10^12 vg<br>Group 4
Heart Weight (g)	Mean	1.6982	1.7842	1.7238	1.8099
	SD	0.2263	0.2913	0.1596	0.3020
	N	5	10	10	10
	%Diff	-	5.0642	1.5075	6.5776
Heart (%br)	Mean	75.24956	80.22451	76.25274	80.20235
	SD	11.03212	12.80385	6.65235	12.35084
	N	5	10	10	10
	%Diff	-	6.61127	1.33313	6.58182
Heart (%bw)	Mean	0.30460	0.29845	0.29479	0.30838
	SD	0.05080	0.02111	0.01300	0.04804
	N	5	10	10	10
	%Diff	-	-2.01729	-3.21892	1.24269
Kidney Weight (g)	Mean	3.7300	3.4210	3.6491	3.7924
	SD	0.5684	0.5817	0.3805	0.4061
	N	5	10	10	10
	%Diff	-	-8.2842	-2.1689	1.6729
Kidney (%br)	Mean	165.02527	154.22700	161.23886	168.23485
	SD	24.57872	27.70715	13.23888	16.48505
	N	5	10	10	10
	%Diff	-	-6.54340	-2.29444	1.94490
Kidney (%bw)	Mean	0.66547	0.58287	0.62464	0.64824
	SD	0.08678	0.11636	0.04586	0.07939
	N	5	10	10	10
	%Diff	-	-12.41278	-6.13622	-2.58857
Liver Weight (g)	Mean	18.6000	20.9116	19.9842	22.1079
	SD	4.1260	4.4133	3.2559	8.2191
	N	5	10	10	10
	%Diff	-	12.4280	7.4419	18.8597
Liver (%br)	Mean	824.77441	940.75873	885.13367	972.59706
	SD	192.66420	194.43763	147.18669	328.22100
	N	5	10	10	10
	%Diff	-	14.06255	7.31828	17.92280
Liver (%bw)	Mean	3.32157	3.47819	3.40044	3.73369
	SD	0.78393	0.34461	0.28468	1.28863
	N	5	10	10	10
	%Diff	-	4.71528	2.37476	12.40757

Appendix 18
Table 4

Summary of Organ Weights: Terminal Day 91±2

32025165

Sex: Male<br>Day(s) Relative to Start Date		0 vg<br>Group 1	1.49x10^11 vg<br>Group 2	4.47x10^11 vg<br>Group 3	1.49x10^12 vg<br>Group 4
Spleen (g)	Mean	0.7320	0.8063	0.7990	0.9056
	SD	0.0901	0.2055	0.1254	0.1455
	N	5	10	10	10
	%Diff	-	10.1503	9.1530	23.7158
Spleen (%br)	Mean	32.36146	36.12140	35.31364	40.06184
	SD	3.64732	8.70492	5.15866	5.23330
	N	5	10	10	10
	%Diff	-	11.61857	9.12251	23.79492
Spleen (%bw)	Mean	0.13172	0.13531	0.13779	0.15465
	SD	0.02311	0.02949	0.02515	0.02472
	N	5	10	10	10
	%Diff	-	2.72406	4.60518	17.40990
Testis Weight (g)	Mean	3.7714	3.7355	3.8981	3.6405
	SD	0.2113	0.4865	0.3200	0.4347
	N	5	10	10	10
	%Diff	-	-0.9519	3.3595	-3.4709
Testis (%br)	Mean	166.81624	168.21851	172.66245	161.18764
	SD	7.95587	23.33300	15.67563	14.23085
	N	5	10	10	10
	%Diff	-	0.84061	3.50458	-3.37413
Testis (%bw)	Mean	0.68052	0.63341	0.67004	0.62132
	SD	0.11061	0.09711	0.06819	0.07115
	N	5	10	10	10
	%Diff	-	-6.92251	-1.54078	-8.70044
Thymus Weight (g)	Mean	0.2172	0.3892	0.3800	0.3867
	SD	0.0704	0.1219	0.1320	0.1423
	N	5	10	10	10
	%Diff	-	79.1897	74.9540	78.0387
Thymus (%br)	Mean	9.64741	17.52831	16.68101	17.11034
	SD	3.27021	5.33566	5.54348	6.01049
	N	5	10	10	10
	%Diff	-	81.68939	72.90674	77.35685
Thymus (%bw)	Mean	0.03889	0.06566	0.06480	0.06509
	SD	0.01317	0.01939	0.02140	0.01986
	N	5	10	10	10
	%Diff	-	68.84918	66.61576	67.36275

Appendix 18
Table 4

Summary of Organ Weights: Terminal Day 91±2

32025165

Sex: Female<br>Day(s) Relative to Start Date		0 vg<br>Group 1	1.49x10^11 vg<br>Group 2	4.47x10^11 vg<br>Group 3	1.49x10^12 vg<br>Group 4
Terminal Body Weight (g)	Mean	270.2232	294.1848	284.3957	288.0944
	SD	24.0554	28.2415	33.6386	19.6647
	N	5	10	10	10
	%Diff	-	8.8673	5.2447	6.6135
Brain (g)	Mean	1.9744	1.9896	2.0096	1.9860
	SD	0.0380	0.1475	0.1144	0.0733
	N	5	10	10	10
	%Diff	-	0.7699	1.7828	0.5875
Brain (%bw)	Mean	0.73560	0.67887	0.71194	0.69209
	SD	0.07011	0.05079	0.05897	0.05057
	N	5	10	10	10
	%Diff	-	-7.71196	-3.21653	-5.91533
Adrenal (g)	Mean	0.05580	0.06190	0.07440	0.06980
	SD	0.00773	0.00687	0.01362	0.00800
	N	5	10	10	10
	%Diff	-	10.93190	33.33333	25.08961
Adrenal (%br)	Mean	2.82699	3.12423	3.70694	3.51910
	SD	0.39602	0.39683	0.67335	0.42903
	N	5	10	10	10
	%Diff	-	10.51439	31.12672	24.48220
Adrenal (%bw)	Mean	0.02089	0.02121	0.02638	0.02420
	SD	0.00433	0.00308	0.00522	0.00177
	N	5	10	10	10
	%Diff	-	1.49805	26.27761	15.84585
Pituitary (g)	Mean	0.01814	0.02126	0.02368	0.02352
	SD	0.00548	0.00391	0.00726	0.00376
	N	5	10	10	10
	%Diff	-	17.19956	30.54024	29.65821
Pituitary (%br)	Mean	0.91885	1.06544	1.17411	1.18410
	SD	0.27572	0.14658	0.33004	0.18296
	N	5	10	10	10
	%Diff	-	15.95308	27.78037	28.86773
Pituitary (%bw)	Mean	0.00682	0.00720	0.00825	0.00823
	SD	0.00243	0.00086	0.00195	0.00162
	N	5	10	10	10
	%Diff	-	5.60438	21.02906	20.72752

DRAFT, NOT FOR SUBMISSION

Appendix 18

Table 4

Summary of Organ Weights: Terminal Day 91±2

32025165

Sex: Female / Day(s) Relative to Start Date		0 vg Group 1	1.49x10^11 vg Group 2	4.47x10^11 vg Group 3	1.49x10^12 vg Group 4
Thyroid/Parathyroid (g)	Mean	0.02236	0.02348	0.02217	0.02274
	SD	0.00399	0.00661	0.00316	0.00538
	N	5	10	10	10
	%Diff	-	5.00894	-0.84973	1.69946
Thyroid/Parathyroid (%br)	Mean	1.13230	1.17335	1.10202	1.14856
	SD	0.19976	0.26100	0.13483	0.28994
	N	5	10	10	10
	%Diff	-	3.62511	-2.67384	1.43569
Thyroid/Parathyroid (%bw)	Mean	0.00838	0.00788	0.00781	0.00794
	SD	0.00197	0.00132	0.00089	0.00196
	N	5	10	10	10
	%Diff	-	-5.93380	-6.79350	-5.26203
Heart Weight (g)	Mean	1.0196	1.0662	1.0898	1.0320
	SD	0.0680	0.1295	0.1529	0.1034
	N	5	10	10	10
	%Diff	-	4.5704	6.8851	1.2162
Heart (%br)	Mean	51.70504	53.73666	54.09707	52.05441
	SD	4.31756	6.56080	5.32683	5.87610
	N	5	10	10	10
	%Diff	-	3.92925	4.62630	0.67569
Heart (%bw)	Mean	0.37991	0.36212	0.38313	0.35798
	SD	0.04572	0.02352	0.02682	0.02112
	N	5	10	10	10
	%Diff	-	-4.68252	0.84766	-5.77340
Kidney Weight (g)	Mean	2.0688	2.1416	2.1294	2.1626
	SD	0.1568	0.2151	0.2971	0.2308
	N	5	10	10	10
	%Diff	-	3.5189	2.9292	4.5340
Kidney (%br)	Mean	104.86099	108.00397	105.78886	108.76869
	SD	9.03630	11.58756	11.65393	9.51367
	N	5	10	10	10
	%Diff	-	2.99728	0.88485	3.72654
Kidney (%bw)	Mean	0.76679	0.72902	0.74921	0.75142
	SD	0.02902	0.05013	0.06910	0.06924
	N	5	10	10	10
	%Diff	-	-4.92554	-2.29273	-2.00458

DRAFT, NOT FOR SUBMISSION

Appendix 18
Table 4

Summary of Organ Weights: Terminal Day 91±2

32025165

Sex: Female<br>Day(s) Relative to Start Date		0 vg<br>Group 1	1.49x10^11 vg<br>Group 2	4.47x10^11 vg<br>Group 3	1.49x10^12 vg<br>Group 4
Liver Weight (g)	Mean	9.7814	10.8227	11.0738	10.6517
	SD	1.6849	1.4650	2.1847	0.9547
	N	5	10	10	10
	%Diff	-	10.6457	13.2128	8.8975
Liver (%br)	Mean	495.77864	544.44464	548.87295	536.98234
	SD	88.19115	61.39349	84.28069	52.55795
	N	5	10	10	10
	%Diff	-	9.81607	10.70928	8.31090
Liver (%bw)	Mean	3.60655	3.67349	3.87148	3.70196
	SD	0.41546	0.24553	0.36844	0.28798
	N	5	10	10	10
	%Diff	-	1.85620	7.34580	2.64556
Ovary Paired (g)	Mean	0.0824	0.0863	0.0841	0.0786
	SD	0.0115	0.0233	0.0199	0.0174
	N	5	10	10	10
	%Diff	-	4.7330	2.0631	-4.6117
Ovary Paired (%br)	Mean	4.18178	4.37509	4.19865	3.94374
	SD	0.64194	1.26147	1.02503	0.76504
	N	5	10	10	10
	%Diff	-	4.62267	0.40359	-5.69231
Ovary Paired (%bw)	Mean	0.03083	0.02962	0.02989	0.02715
	SD	0.00638	0.00849	0.00737	0.00491
	N	5	10	10	10
	%Diff	-	-3.91709	-3.05176	-11.94725
Spleen (g)	Mean	0.4836	0.5276	0.5298	0.5359
	SD	0.0659	0.1032	0.0604	0.1344
	N	5	10	10	10
	%Diff	-	9.0984	9.5533	10.8147
Spleen (%br)	Mean	24.54022	26.53119	26.38923	26.94212
	SD	3.74130	4.92886	2.94322	6.30674
	N	5	10	10	10
	%Diff	-	8.11309	7.53462	9.78762
Spleen (%bw)	Mean	0.17908	0.18041	0.18704	0.18556
	SD	0.01846	0.03860	0.01804	0.04243
	N	5	10	10	10
	%Diff	-	0.74571	4.44757	3.61809

DRAFT, NOT FOR SUBMISSION

Appendix 18

Table 4

Summary of Organ Weights: Terminal Day 91±2

32025165

Sex: Female<br>Day(s) Relative to Start Date		0 vg<br>Group 1	1.49x10^11 vg<br>Group 2	4.47x10^11 vg<br>Group 3	1.49x10^12 vg<br>Group 4
Thymus Weight (g)	Mean	0.2468	0.2988	0.2726	0.3165
	SD	0.0870	0.0480	0.0340	0.0722
	N	5	10	10	10
	%Diff	-	21.0697	10.4538	28.2415
Thymus (%br)	Mean	12.51016	15.13856	13.60802	15.96553
	SD	4.49498	3.00549	1.92782	3.67566
	N	5	10	10	10
	%Diff	-	21.01014	8.77575	27.62053
Thymus (%bw)	Mean	0.08971	0.10246	0.09678	0.11036
	SD	0.02531	0.01980	0.01503	0.02740
	N	5	10	10	10
	%Diff	-	14.20638	7.87346	23.01207
Uterus/Cervix (g)	Mean	0.8956	0.8645	0.7642	0.7731
	SD	0.2212	0.1859	0.2051	0.1693
	N	5	10	10	10
	%Diff	-	-3.4725	-14.6717	-13.6780
Uterus/Cervix (%br)	Mean	45.24799	43.59582	37.92227	38.98913
	SD	10.47432	9.84129	9.53187	8.69580
	N	5	10	10	10
	%Diff	-	-3.65138	-16.19017	-13.83236
Uterus/Cervix (%bw)	Mean	0.33383	0.29573	0.26590	0.26939
	SD	0.08709	0.06968	0.05512	0.06258
	N	5	10	10	10
	%Diff	-	-11.41368	-20.34777	-19.30498

DRAFT, NOT FOR SUBMISSION

Appendix 18
Table 5

Summary of Microscopic Pathology: Terminal Day 29±1

32025165

Removal Reason(s): TERMINAL EUTHANASIA<br>Summary: Incidence	Male		Female	
	0 vg Group 1	1.49x10^12 vg Group 4	0 vg Group 1	1.49x10^12 vg Group 4
Number of Animals:	5	5	5	5
**BRAIN**				
Examined	5	5	5	5
No Visible Lesions	5	5	5	5
**GANGLION, DORSAL ROOT, CERVICAL**				
Examined	5	5	5	5
No Visible Lesions	5	5	5	5
**GANGLION, DORSAL ROOT, LUMBAR**				
Examined	5	5	5	5
No Visible Lesions	4	3	4	0
Infiltration, mononuclear cell	1	2	1	5
.... minimal	1	1	1	4
.... mild	0	1	0	1
Degeneration; nerve fiber	0	2	0	2
.... minimal	0	1	0	0
.... mild	0	1	0	2
Degeneration/necrosis; neuronal	0	1	0	2
.... minimal	0	1	0	2
**GANGLION, DORSAL ROOT, THORACIC**				
Examined	5	5	5	5
No Visible Lesions	5	5	5	5
**GLAND, ADRENAL**				
Examined	5	5	5	5
No Visible Lesions	5	5	5	5
**GLAND, MAMMARY**				
Examined	5	5	5	5
No Visible Lesions	5	4	5	5
Infiltration, mixed cell	0	1	0	0
.... minimal	0	1	0	0
Necrosis	0	1	0	0
.... minimal	0	1	0	0
**HEART**				
Examined	5	5	5	5
No Visible Lesions	5	5	5	5

DRAFT, NOT FOR SUBMISSION

Appendix 18
Table 5

Summary of Microscopic Pathology: Terminal Day 29±1

32025165

Removal Reason(s): TERMINAL EUTHANASIA<br>Summary: Incidence	Male		Female	
	0 vg Group 1	1.49x10^12 vg Group 4	0 vg Group 1	1.49x10^12 vg Group 4
Number of Animals:	5	5	5	5
**KIDNEY**				
Examined	5	5	5	5
No Visible Lesions	4	5	4	3
Mineralization	1	0	0	1
.... mild	1	0	0	1
Necrosis	1	0	0	0
.... mild	1	0	0	0
Infiltration, mixed cell; adipose tissue	0	0	0	1
.... minimal	0	0	0	1
Dilatation; pelvis	0	0	1	1
.... minimal	0	0	1	1
**LARGE INTESTINE, COLON**				
Examined	5	5	5	5
No Visible Lesions	5	5	5	5
**LIVER**				
Examined	5	5	5	5
No Visible Lesions	5	4	5	5
Vacuolation; hepatocellular	0	1	0	0
.... minimal	0	1	0	0
**LUNG**				
Examined	5	5	5	5
No Visible Lesions	5	5	4	5
Alveolar macrophages, increased	0	0	1	0
.... minimal	0	0	1	0
**LYMPH NODE, ILIAC**				
Examined	5	5	5	5
No Visible Lesions	5	5	5	5
**NERVE, SCIATIC**				
Examined	5	5	5	5
No Visible Lesions	5	3	5	1
Degeneration; nerve fiber	0	2	0	4
.... minimal	0	2	0	3
.... mild	0	0	0	1
Infiltration, mononuclear cell	0	0	0	1

DRAFT, NOT FOR SUBMISSION

Appendix 18

Table 5

Summary of Microscopic Pathology: Terminal Day 29±1

32025165

Removal Reason(s): TERMINAL EUTHANASIA<br>Summary: Incidence	Male		Female	
	0 vg Group 1	1.49x10^12 vg Group 4	0 vg Group 1	1.49x10^12 vg Group 4
Number of Animals:	5	5	5	5
**NERVE, SCIATIC (Continued...)**				
.... minimal	0	0	0	1
**OVARY**				
Examined	.	.	5	5
No Visible Lesions	.	.	5	5
**PANCREAS**				
Examined	5	5	5	5
No Visible Lesions	5	5	5	5
**SPINAL CORD**				
Examined	5	5	5	5
No Visible Lesions	5	3	5	2
Degeneration; nerve fiber	0	2	0	3
.... minimal	0	1	0	3
.... mild	0	1	0	0
**SPLEEN**				
Examined	5	5	5	5
No Visible Lesions	5	5	5	5
**TESTIS, RIGHT**				
Examined	5	5	.	.
No Visible Lesions	5	5	.	.

DRAFT, NOT FOR SUBMISSION

Appendix 18
Table 6

Summary of Microscopic Pathology: Terminal Day 91±2

32025165

Removal Reason(s): TERMINAL EUTHANASIA	Male				Female			
Summary: Incidence	0 vg Group 1	1.49x10^11 vg Group 2	4.47x10^11 vg Group 3	1.49x10^12 vg Group 4	0 vg Group 1	1.49x10^11 vg Group 2	4.47x10^11 vg Group 3	1.49x10^12 vg Group 4
Number of Animals:	5	10	10	10	5	10	10	10
**BRAIN**								
Examined	5	10	10	10	5	10	10	10
No Visible Lesions	5	10	10	10	5	10	10	10
**GANGLION, DORSAL ROOT, CERVICAL**								
Examined	5	10	10	10	5	10	10	10
No Visible Lesions	5	10	10	9	5	10	9	10
Infiltration, mononuclear cell	0	0	0	1	0	0	1	0
.... minimal	0	0	0	1	0	0	1	0
Degeneration; nerve fiber	0	0	0	0	0	0	1	0
.... minimal	0	0	0	0	0	0	1	0
**GANGLION, DORSAL ROOT, LUMBAR**								
Examined	5	10	10	10	5	10	10	10
No Visible Lesions	2	8	3	0	5	4	5	0
Infiltration, mononuclear cell	2	2	6	7	0	5	4	8
.... minimal	2	2	5	5	0	5	3	5
.... mild	0	0	1	2	0	0	1	3
Degeneration; nerve fiber	1	1	6	10	0	3	4	10
.... minimal	1	1	5	5	0	3	2	6
.... mild	0	0	1	4	0	0	2	2
.... moderate	0	0	0	1	0	0	0	2
Degeneration/necrosis; neuronal	0	0	1	2	0	0	1	2
.... minimal	0	0	1	2	0	0	1	2
Aggregate; glial cell	0	0	3	8	0	0	2	8
.... minimal	0	0	3	7	0	0	1	6
.... mild	0	0	0	1	0	0	1	2
**GANGLION, DORSAL ROOT, THORACIC**								
Examined	5	10	10	10	5	10	10	10
No Visible Lesions	5	9	9	7	5	10	10	9
Degeneration; nerve fiber	0	1	0	3	0	0	0	1
.... minimal	0	1	0	3	0	0	0	0

Appendix 18
Table 6

Summary of Microscopic Pathology: Terminal Day 91±2

32025165

Removal Reason(s): TERMINAL EUTHANASIA	Male				Female			
Summary: Incidence	0 vg Group 1	1.49x10^11 vg Group 2	4.47x10^11 vg Group 3	1.49x10^12 vg Group 4	0 vg Group 1	1.49x10^11 vg Group 2	4.47x10^11 vg Group 3	1.49x10^12 vg Group 4
Number of Animals:	5	10	10	10	5	10	10	10
**GANGLION, DORSAL ROOT, THORACIC (Continued...)**								
.... mild	0	0	0	0	0	0	0	1
Autophagy	0	0	1	0	0	0	0	0
.... minimal	0	0	1	0	0	0	0	0
**GLAND, ADRENAL**								
Examined	5	10	10	10	5	10	10	10
No Visible Lesions	4	10	10	10	5	10	10	10
Vacuolation; cortical	1	0	0	0	0	0	0	0
.... mild	1	0	0	0	0	0	0	0
**GLAND, MAMMARY**								
Examined	4	10	10	10	5	10	9	10
No Visible Lesions	4	10	10	10	5	10	9	10
Not Examined: Not Present In Section.	1	0	0	0	0	0	1	0
**HEART**								
Examined	5	10	10	10	5	10	10	10
No Visible Lesions	5	10	10	8	5	10	10	10
Infiltration, mononuclear cell	0	0	0	2	0	0	0	0
.... minimal	0	0	0	1	0	0	0	0
.... mild	0	0	0	1	0	0	0	0
**KIDNEY**								
Examined	5	10	10	10	5	10	10	10
No Visible Lesions	5	9	9	9	5	10	10	10
Infarct	0	1	0	1	0	0	0	0
.... moderate	0	1	0	1	0	0	0	0
Cyst	0	0	1	0	0	0	0	0
.... mild	0	0	1	0	0	0	0	0
**LARGE INTESTINE, COLON**								
Examined	5	10	10	10	5	10	10	10
No Visible Lesions	5	10	10	10	5	10	10	10
**LIVER**								
Examined	5	10	10	10	5	10	10	10
No Visible Lesions	5	10	9	8	3	10	10	10

DRAFT, NOT FOR SUBMISSION

Appendix 18
Table 6

Summary of Microscopic Pathology: Terminal Day 91±2

32025165

Removal Reason(s): TERMINAL EUTHANASIA	Male				Female			
Summary: Incidence	0 vg Group 1	1.49x10^11 vg Group 2	4.47x10^11 vg Group 3	1.49x10^12 vg Group 4	0 vg Group 1	1.49x10^11 vg Group 2	4.47x10^11 vg Group 3	1.49x10^12 vg Group 4
Number of Animals:	5	10	10	10	5	10	10	10
**LIVER (Continued...)**								
Hepatodiaphragmatic nodule	0	0	0	1	0	0	0	0
.... mild	0	0	0	1	0	0	0	0
Vacuolation; hepatocellular	0	0	0	1	0	0	0	0
.... marked	0	0	0	1	0	0	0	0
Infiltration, mononuclear cell	0	0	1	1	2	0	0	0
.... minimal	0	0	1	1	2	0	0	0
Eosinophilic globules; hepatocellular	0	0	0	1	0	0	0	0
.... marked	0	0	0	1	0	0	0	0
**LUNG**								
Examined	5	10	10	10	5	10	10	10
No Visible Lesions	5	10	10	10	5	10	10	10
**LYMPH NODE, ILIAC**								
Examined	5	10	10	10	5	10	10	10
No Visible Lesions	5	10	10	10	5	10	10	10
**NERVE, SCIATIC**								
Examined	5	10	10	10	5	10	10	10
No Visible Lesions	5	6	5	2	5	8	5	1
Degeneration; nerve fiber	0	4	5	8	0	2	5	9
.... minimal	0	4	5	8	0	2	5	9
**OVARY**								
Examined	.	.	.	.	5	10	10	10
No Visible Lesions	.	.	.	.	3	6	8	7
Atrophy	.	.	.	.	1	4	2	3
.... minimal	.	.	.	.	1	2	1	2
.... mild	.	.	.	.	0	0	1	1
.... moderate	.	.	.	.	0	2	0	0
Cyst	.	.	.	.	1	0	0	0
.... mild	.	.	.	.	1	0	0	0
**PANCREAS**								
Examined	5	10	10	10	5	10	10	9
No Visible Lesions	2	9	8	7	5	10	10	9

DRAFT, NOT FOR SUBMISSION

Appendix 18
Table 6

Summary of Microscopic Pathology: Terminal Day 91±2

32025165

Removal Reason(s): TERMINAL EUTHANASIA	Male				Female			
Summary: Incidence	0 vg Group 1	1.49x10^11 vg Group 2	4.47x10^11 vg Group 3	1.49x10^12 vg Group 4	0 vg Group 1	1.49x10^11 vg Group 2	4.47x10^11 vg Group 3	1.49x10^12 vg Group 4
Number of Animals:	5	10	10	10	5	10	10	10
**PANCREAS (Continued...)**								
Not Examined: Not Present In Section.	.	.	.	.	0	0	0	1
Atrophy	2	0	2	2	0	0	0	0
.... minimal	2	0	1	2	0	0	0	0
.... mild	0	0	1	0	0	0	0	0
Fibrosis; islet of langerhans	1	1	2	2	0	0	0	0
.... minimal	1	0	1	2	0	0	0	0
.... mild	0	1	1	0	0	0	0	0
Infiltration, mononuclear cell	0	0	2	1	0	0	0	0
.... minimal	0	0	1	1	0	0	0	0
.... mild	0	0	1	0	0	0	0	0
**SPINAL CORD**								
Examined	5	10	10	10	5	10	10	10
No Visible Lesions	4	8	7	3	5	8	4	1
Degeneration; nerve fiber	1	2	3	7	0	2	6	9
.... minimal	0	2	3	5	0	2	4	2
.... mild	1	0	0	2	0	0	2	7
**SPLEEN**								
Examined	5	10	10	10	5	10	10	10
No Visible Lesions	5	10	10	10	5	10	10	10
**TESTIS, RIGHT**								
Examined	5	10	10	10	.	.	.	.
No Visible Lesions	5	9	10	10	.	.	.	.
Hypospermatogenesis	0	1	0	0	.	.	.	.
.... mild	0	1	0	0	.	.	.	.
**THYMUS**								
Examined	.	.	.	.	0	0	1	0
Hemorrhage	.	.	.	.	.	.	1	.
.... mild	.	.	.	.	.	.	1	.
**UTERUS**								
Examined	.	.	.	.	0	1	0	0
Cyst	.	.	.	.	.	1	.	.

DRAFT, NOT FOR SUBMISSION

Appendix 18
Table 6

Summary of Microscopic Pathology: Terminal Day 91±2

32025165

Removal Reason(s): TERMINAL EUTHANASIA	Male				Female			
Summary: Incidence	0 vg Group 1	1.49x10^11 vg Group 2	4.47x10^11 vg Group 3	1.49x10^12 vg Group 4	0 vg Group 1	1.49x10^11 vg Group 2	4.47x10^11 vg Group 3	1.49x10^12 vg Group 4
Number of Animals:	5	10	10	10	5	10	10	10
**UTERUS (Continued...)**								
.... mild	.	.	.	.	.	1	.	.

DRAFT, NOT FOR SUBMISSION

Appendix 18

EXPLANATION PAGE

All Day(s) referenced throughout the outputs generated are based upon Day 1 (first day of dose initiation).

Note: All abbreviations listed on this page may not be applicable to this report.

Abbreviation	Description
% Diff	% Difference from Group 1
tCtrl	Times control (fold change)
1F, 2F, 3F, 4F	Group 1 Female, Group 2 Female, Group 3 Female, Group 4 Female
1M, 2M, 3M, 4M	Group 1 Male, Group 2 Male, Group 3 Male, Group 4 Male
N	Number of values included in analysis
M, F	Male, Female
TK	Toxicokinetic
--	Not scheduled to be performed/dead
- or .	Not applicable

Abbreviation	Description	Abbreviation	Description
%br	% Organ Weight/Brain Weight	%bw	% Organ Weight/Terminal Bodyweight
COME	See comment value excluded	Sup	Suppress
E	Exclude	NA	Not applicable
No.	Animal number	Wt	Weight
C	clinical observations	G	gross observations
TGL	trackable gross lesions		

Appendix 18
Appendix 1

Individual Organ Weights: Terminal Day 29±1

32025165
Sex: Male Day(s) Relative to Start Date

0 vg	Organ Weights					
Group 1	Terminal Body Wt (g)	Brain (g)	Brain (%bw)	Epididymis (g)	Epididymis (%br)	Epididymis (%bw)
	-	-	-	-	-	-
1001	436.410	2.017	0.4622	1.208	59.8909	0.2768
1002	365.193	2.137	0.5852	1.187	55.5452	0.3250
1003	423.834	2.072	0.4889	1.172	56.5637	0.2765
1004	336.276	1.959	0.5826	1.171	59.7754	0.3482
1005	385.359	2.277	0.5909	1.077	47.2991	0.2795
Mean	389.4144	2.0924	0.54193	1.1630	55.81485	0.30121
SD	41.2920	0.1224	0.06142	0.0504	5.13450	0.03337
N	5	5	5	5	5	5

DRAFT, NOT FOR SUBMISSION

Appendix 18

Appendix 1

Individual Organ Weights: Terminal Day 29±1

32025165

Sex: Male Day(s) Relative to Start Date

0 vg	Organ Weights					
Group 1	Adrenal (g)	Adrenal (%br)	Adrenal (%bw)	Pituitary (g)	Pituitary (%br)	Pituitary (%bw)
	-	-	-	-	-	-
1001	0.0650	3.2226	0.0149	0.0164	0.8131	0.0038
1002	0.0490	2.2929	0.0134	0.0174	0.8142	0.0048
1003	0.0520	2.5097	0.0123	0.0176	0.8494	0.0042
1004	0.0580	2.9607	0.0172	0.0164	0.8372	0.0049
1005	0.0540	2.3715	0.0140	0.0155	0.6807	0.0040
Mean	0.05560	2.67149	0.01437	0.01666	0.79892	0.00431
SD	0.00619	0.40213	0.00187	0.00085	0.06786	0.00048
N	5	5	5	5	5	5

DRAFT, NOT FOR SUBMISSION

Appendix 18

Appendix 1

Individual Organ Weights: Terminal Day 29±1

32025165

Sex: Male Day(s) Relative to Start Date

0 vg	Organ Weights					
Group 1	Prostate (g)	Prostate (%br)	Prostate (%bw)	Thyroid/ Parathyroid (g)	Thyroid/ Parathyroid (%br)	Thyroid/ Parathyroid (%bw)
	-	-	-	-	-	-
1001	0.880	43.6292	0.2016	0.0249	1.2345	0.0057
1002	0.934	43.7061	0.2558	0.0287	1.3430	0.0079
1003	0.940	45.3668	0.2218	0.0265	1.2790	0.0063
1004	1.137	58.0398	0.3381	0.0276	1.4089	0.0082
1005	0.954	41.8972	0.2476	0.0207	0.9091	0.0054
Mean	0.9690	46.52783	0.25297	0.02568	1.23489	0.00668
SD	0.0980	6.55135	0.05219	0.00312	0.19368	0.00128
N	5	5	5	5	5	5

DRAFT, NOT FOR SUBMISSION

Appendix 18

Appendix 1

Individual Organ Weights: Terminal Day 29±1

32025165

Sex: Male Day(s) Relative to Start Date

0 vg	Organ Weights					
	Heart	Heart	Heart	Kidney	Kidney	Kidney
Group 1	(g)	(%br)	(%bw)	(g)	(%br)	(%bw)
	-	-	-	-	-	-
1001	1.542	76.4502	0.3533	3.255	161.3783	0.7459
1002	1.327	62.0964	0.3634	2.751	128.7319	0.7533
1003	1.347	65.0097	0.3178	3.036	146.5251	0.7163
1004	1.418	72.3839	0.4217	3.051	155.7427	0.9073
1005	1.356	59.5520	0.3519	2.877	126.3505	0.7466
Mean	1.3980	67.09843	0.36162	2.9940	143.74569	0.77387
SD	0.0874	7.10145	0.03772	0.1910	15.73667	0.07594
N	5	5	5	5	5	5

DRAFT, NOT FOR SUBMISSION

Appendix 18

Appendix 1

Individual Organ Weights: Terminal Day 29±1

32025165

Sex: Male Day(s) Relative to Start Date

0 vg	Organ Weights					
Group 1	Liver (g)	Liver (%br)	Liver (%bw)	Spleen (g)	Spleen (%br)	Spleen (%bw)
	-	-	-	-	-	-
1001	18.960	940.0099	4.3445	0.880	43.6292	0.2016
1002	13.831	647.2157	3.7873	0.636	29.7613	0.1742
1003	15.864	765.6371	3.7430	0.804	38.8031	0.1897
1004	15.827	807.9122	4.7066	0.599	30.5768	0.1781
1005	13.426	589.6355	3.4840	0.492	21.6074	0.1277
Mean	15.5816	750.08208	4.01308	0.6822	32.87556	0.17426
SD	2.1946	137.82549	0.49865	0.1575	8.55467	0.02817
N	5	5	5	5	5	5

Appendix 18

Appendix 1

Individual Organ Weights: Terminal Day 29±1

32025165

Sex: Male Day(s) Relative to Start Date

0 vg	Organ Weights					
	Testis	Testis	Testis	Thymus	Thymus	Thymus
Group 1	(g)	(%br)	(%bw)	(g)	(%br)	(%bw)
	-	-	-	-	-	-
1001	3.617	179.3257	0.8288	0.690	34.2092	0.1581
1002	3.640	170.3322	0.9967	0.396	18.5307	0.1084
1003	3.511	169.4498	0.8284	0.592	28.5714	0.1397
1004	3.168	161.7152	0.9421	0.586	29.9132	0.1743
1005	2.644	116.1177	0.6861	0.441	19.3676	0.1144
Mean	3.3160	159.38813	0.85643	0.5410	26.11842	0.13898
SD	0.4204	24.98131	0.11998	0.1203	6.87435	0.02805
N	5	5	5	5	5	5

DRAFT, NOT FOR SUBMISSION

Appendix 18

Appendix 1

Individual Organ Weights: Terminal Day 29±1

32025165

Sex: Male Day(s) Relative to Start Date

1.49x10^12 vg Group 4	Organ Weights					
	Terminal Body Wt (g)	Brain (g)	Brain (%bw)	Epididymis (g)	Epididymis (%br)	Epididymis (%bw)
	-	-	-	-	-	-
4001	490.369	2.059	0.4199	1.446	70.2283	0.2949
4002	434.867	2.129	0.4896	1.387	65.1480	0.3189
4003	395.719	2.087	0.5274	1.296	62.0987	0.3275
4004	419.072	2.074	0.4949	1.288	62.1022	0.3073
4005	509.435	2.070	0.4063	1.219	58.8889	0.2393
Mean	449.8924	2.0838	0.46762	1.3272	63.69321	0.29759
SD	48.2029	0.0272	0.05204	0.0893	4.27137	0.03483
N	5	5	5	5	5	5
%Diff	15.5305	-0.4110	-13.71254	14.1187	14.11516	-1.20199

DRAFT, NOT FOR SUBMISSION

Appendix 18

Appendix 1

Individual Organ Weights: Terminal Day 29±1

32025165

Sex: Male Day(s) Relative to Start Date

1.49x10^12 vg Group 4	Organ Weights					
	Adrenal (g)	Adrenal (%br)	Adrenal (%bw)	Pituitary (g)	Pituitary (%br)	Pituitary (%bw)
	-	-	-	-	-	-
4001	0.0470	2.2827	0.0096	0.0175	0.8499	0.0036
4002	0.0540	2.5364	0.0124	0.0135	0.6341	0.0031
4003	0.0690	3.3062	0.0174	0.0143	0.6852	0.0036
4004	0.0480	2.3144	0.0115	0.0132	0.6365	0.0031
4005	0.0600	2.8986	0.0118	0.0160	0.7729	0.0031
Mean	0.05560	2.66763	0.01253	0.01490	0.71572	0.00332
SD	0.00913	0.43338	0.00294	0.00182	0.09376	0.00025
N	5	5	5	5	5	5
%Diff	0.00000	-0.14424	-12.76556	-10.56423	-10.41395	-23.16143

DRAFT, NOT FOR SUBMISSION

Appendix 18

Appendix 1

Individual Organ Weights: Terminal Day 29±1

32025165

Sex: Male Day(s) Relative to Start Date

1.49x10^12 vg Group 4	Organ Weights					
	Prostate (g)	Prostate (%br)	Prostate (%bw)	Thyroid/ Parathyroid (g)	Thyroid/ Parathyroid (%br)	Thyroid/ Parathyroid (%bw)
	-	-	-	-	-	-
4001	1.425	69.2084	0.2906	0.0255	1.2385	0.0052
4002	1.157	54.3448	0.2661	0.0377	1.7708	0.0087
4003	0.906	43.4116	0.2290	0.0225	1.0781	0.0057
4004	1.041	50.1929	0.2484	0.0280	1.3500	0.0067
4005	1.064	51.4010	0.2089	0.0281	1.3575	0.0055
Mean	1.1186	53.71171	0.24857	0.02836	1.35898	0.00635
SD	0.1934	9.54412	0.03176	0.00570	0.25646	0.00141
N	5	5	5	5	5	5
%Diff	15.4386	15.43997	-1.73859	10.43614	10.04863	-4.92102

DRAFT, NOT FOR SUBMISSION

Appendix 18

Appendix 1

Individual Organ Weights: Terminal Day 29±1

32025165

Sex: Male Day(s) Relative to Start Date

1.49x10^12 vg Group 4	Organ Weights					
	Heart (g)	Heart (%br)	Heart (%bw)	Kidney (g)	Kidney (%br)	Kidney (%bw)
	-	-	-	-	-	-
4001	1.490	72.3652	0.3039	3.300	160.2720	0.6730
4002	1.575	73.9784	0.3622	2.897	136.0733	0.6662
4003	1.393	66.7465	0.3520	2.930	140.3929	0.7404
4004	1.495	72.0829	0.3567	3.426	165.1880	0.8175
4005	1.703	82.2705	0.3343	3.215	155.3140	0.6311
Mean	1.5312	73.48872	0.34182	3.1536	151.44804	0.70564
SD	0.1157	5.61373	0.02366	0.2320	12.65103	0.07401
N	5	5	5	5	5	5
%Diff	9.5279	9.52376	-5.47514	5.3307	5.35832	-8.81711

DRAFT, NOT FOR SUBMISSION

Appendix 18

Appendix 1

Individual Organ Weights: Terminal Day 29±1

32025165

Sex: Male Day(s) Relative to Start Date

1.49x10^12 vg Group 4	Organ Weights					
	Liver (g)	Liver (%br)	Liver (%bw)	Spleen (g)	Spleen (%br)	Spleen (%bw)
	-	-	-	-	-	-
4001	19.683	955.9495	4.0139	0.715	34.7256	0.1458
4002	18.741	880.2724	4.3096	0.809	37.9991	0.1860
4003	16.463	788.8356	4.1603	0.702	33.6368	0.1774
4004	17.455	841.6104	4.1652	0.968	46.6731	0.2310
4005	18.675	902.1739	3.6658	0.999	48.2609	0.1961
Mean	18.2034	873.76838	4.06295	0.8386	40.25908	0.18727
SD	1.2540	62.94961	0.24539	0.1390	6.79614	0.03085
N	5	5	5	5	5	5
%Diff	16.8263	16.48970	1.24277	22.9258	22.45901	7.46358

DRAFT, NOT FOR SUBMISSION

Appendix 18
Appendix 1

Individual Organ Weights: Terminal Day 29±1

32025165
Sex: Male Day(s) Relative to Start Date

1.49x10^12 vg Group 4	Organ Weights					
	Testis (g)	Testis (%br)	Testis (%bw)	Thymus (g)	Thymus (%br)	Thymus (%bw)
	-	-	-	-	-	-
4001	4.146	201.3599	0.8455	0.407	19.7669	0.0830
4002	3.603	169.2344	0.8285	0.665	31.2353	0.1529
4003	3.581	171.5860	0.9049	0.373	17.8725	0.0943
4004	3.712	178.9778	0.8858	0.539	25.9884	0.1286
4005	3.839	185.4589	0.7536	0.581	28.0676	0.1140
Mean	3.7762	181.32341	0.84366	0.5130	24.58616	0.11457
SD	0.2307	12.89405	0.05889	0.1217	5.62576	0.02774
N	5	5	5	5	5	5
%Diff	13.8782	13.76218	-1.49065	-5.1756	-5.86659	-17.56722

DRAFT, NOT FOR SUBMISSION

Appendix 18

Appendix 1

Individual Organ Weights: Terminal Day 29±1

32025165

Sex: Female Day(s) Relative to Start Date

0 vg	Organ Weights					
Group 1	Terminal Body Wt (g)	Brain (g)	Brain (%bw)	Adrenal (g)	Adrenal (%br)	Adrenal (%bw)
	-	-	-	-	-	-
1501	229.258	1.785	0.7786	0.0750	4.2017	0.0327
1502	221.569	2.092	0.9442	0.0660	3.1549	0.0298
1503	251.241	1.888	0.7515	0.0550	2.9131	0.0219
1504	271.019	2.025	0.7472	0.0600	2.9630	0.0221
1505	210.616	1.812	0.8603	0.0630	3.4768	0.0299
Mean	236.7406	1.9204	0.81635	0.06380	3.34190	0.02729
SD	24.2587	0.1337	0.08469	0.00746	0.52911	0.00496
N	5	5	5	5	5	5

DRAFT, NOT FOR SUBMISSION

Appendix 18

Appendix 1

Individual Organ Weights: Terminal Day 29±1

32025165

Sex: Female Day(s) Relative to Start Date

0 vg Group 1	Organ Weights					
	Pituitary (g)	Pituitary (%br)	Pituitary (%bw)	Thyroid/ Parathyroid (g)	Thyroid/ Parathyroid (%br)	Thyroid/ Parathyroid (%bw)
	-	-	-	-	-	-
1501	0.0207	1.1597	0.0090	0.0228	1.2773	0.0099
1502	0.0179	0.8556	0.0081	0.0226	1.0803	0.0102
1503	0.0132	0.6992	0.0053	0.0222	1.1758	0.0088
1504	0.0201	0.9926	0.0074	0.0271	1.3383	0.0100
1505	0.0179	0.9879	0.0085	0.0226	1.2472	0.0107
Mean	0.01796	0.93898	0.00766	0.02346	1.22380	0.00994
SD	0.00295	0.17207	0.00147	0.00205	0.09926	0.00069
N	5	5	5	5	5	5

DRAFT, NOT FOR SUBMISSION

Appendix 18

Appendix 1

Individual Organ Weights: Terminal Day 29±1

32025165

Sex: Female   Day(s) Relative to Start Date

0 vg	Organ Weights					
	Heart	Heart	Heart	Kidney	Kidney	Kidney
Group 1	(g)	(%br)	(%bw)	(g)	(%br)	(%bw)
	-	-	-	-	-	-
1501	1.006	56.3585	0.4388	1.978	110.8123	0.8628
1502	0.983	46.9885	0.4437	1.711	81.7878	0.7722
1503	0.936	49.5763	0.3726	1.743	92.3199	0.6938
1504	1.007	49.7284	0.3716	2.133	105.3333	0.7870
1505	0.754	41.6115	0.3580	1.667	91.9978	0.7915
Mean	0.9372	48.85264	0.39691	1.8464	96.45023	0.78146
SD	0.1064	5.32772	0.04090	0.2004	11.58718	0.06025
N	5	5	5	5	5	5

DRAFT, NOT FOR SUBMISSION

Appendix 18

Appendix 1

Individual Organ Weights: Terminal Day 29±1

32025165

Sex: Female Day(s) Relative to Start Date

0 vg<br>Group 1	Organ Weights<br>Liver<br>(g)<br>-	 Liver<br>(%br)<br>-	 Liver<br>(%bw)<br>-	 Ovary Paired<br>(g)<br>-	 Ovary Paired<br>(%br)<br>-	 Ovary Paired<br>(%bw)<br>-
1501	8.426	472.0448	3.6753	0.102	5.7143	0.0445
1502	8.119	388.0975	3.6643	0.078	3.7285	0.0352
1503	8.945	473.7818	3.5603	0.104	5.5085	0.0414
1504	11.585	572.0988	4.2746	0.091	4.4938	0.0336
1505	8.300	458.0574	3.9408	0.095	5.2428	0.0451
Mean	9.0750	472.81605	3.82308	0.0940	4.93758	0.03995
SD	1.4364	65.67654	0.28888	0.0104	0.81872	0.00530
N	5	5	5	5	5	5

DRAFT, NOT FOR SUBMISSION

Appendix 18

Appendix 1

Individual Organ Weights: Terminal Day 29±1

32025165

Sex: Female   Day(s) Relative to Start Date

0 vg	Organ Weights					
	Spleen	Spleen	Spleen	Thymus	Thymus	Thymus
Group 1	(g)	(%br)	(%bw)	(g)	(%br)	(%bw)
	-	-	-	-	-	-
1501	0.503	28.1793	0.2194	0.403	22.5770	0.1758
1502	0.493	23.5660	0.2225	0.306	14.6272	0.1381
1503	0.493	26.1123	0.1962	0.411	21.7691	0.1636
1504	0.530	26.1728	0.1956	0.465	22.9630	0.1716
1505	0.452	24.9448	0.2146	0.369	20.3642	0.1752
Mean	0.4942	25.79504	0.20966	0.3908	20.46009	0.16485
SD	0.0280	1.70438	0.01288	0.0586	3.40924	0.01572
N	5	5	5	5	5	5

DRAFT, NOT FOR SUBMISSION

Appendix 18

Appendix 1

Individual Organ Weights: Terminal Day 29±1

32025165

Sex: Female   Day(s) Relative to Start Date

0 vg	Organ Weights		
Group 1	Uterus/ Cervix (g)	Uterus/ Cervix (%br)	Uterus/ Cervix (%bw)
	-	-	-
1501	0.751	42.0728	0.3276
1502	0.560	26.7686	0.2527
1503	0.532	28.1780	0.2117
1504	0.533	26.3210	0.1967
1505	1.115	61.5342	0.5294
Mean	0.6982	36.97493	0.30363
SD	0.2503	15.20040	0.13603
N	5	5	5

DRAFT, NOT FOR SUBMISSION

Appendix 18

Appendix 1

Individual Organ Weights: Terminal Day 29±1

32025165

Sex: Female Day(s) Relative to Start Date

1.49x10^12 vg Group 4	Organ Weights					
	Terminal Body Wt (g)	Brain (g)	Brain (%bw)	Adrenal (g)	Adrenal (%br)	Adrenal (%bw)
	-	-	-	-	-	-
4501	232.924	2.009	0.8625	0.0640	3.1857	0.0275
4502	265.268	2.161	0.8146	0.0600	2.7765	0.0226
4503	235.733	1.994	0.8459	0.0780	3.9117	0.0331
4504	227.041	1.917	0.8443	0.0770	4.0167	0.0339
4505	264.687	2.000	0.7556	0.0650	3.2500	0.0246
Mean	245.1306	2.0162	0.82460	0.06880	3.42812	0.02833
SD	18.3883	0.0889	0.04224	0.00817	0.52331	0.00504
N	5	5	5	5	5	5
%Diff	3.5440	4.9885	1.01001	7.83699	2.58003	3.81953

DRAFT, NOT FOR SUBMISSION

Appendix 18

Appendix 1

Individual Organ Weights: Terminal Day 29±1

32025165

Sex: Female Day(s) Relative to Start Date

1.49x10^12 vg Group 4	Organ Weights					
	Pituitary (g)	Pituitary (%br)	Pituitary (%bw)	Thyroid/ Parathyroid (g)	Thyroid/ Parathyroid (%br)	Thyroid/ Parathyroid (%bw)
	-	-	-	-	-	-
4501	0.0181	0.9009	0.0078	0.0249	1.2394	0.0107
4502	0.0188	0.8700	0.0071	0.0355	1.6428	0.0134
4503	0.0207	1.0381	0.0088	0.0268	1.3440	0.0114
4504	0.0219	1.1424	0.0096	0.0286	1.4919	0.0126
4505	0.0156	0.7800	0.0059	0.0255	1.2750	0.0096
Mean	0.01902	0.94629	0.00784	0.02826	1.39863	0.01153
SD	0.00243	0.14355	0.00146	0.00429	0.16727	0.00149
N	5	5	5	5	5	5
%Diff	5.90200	0.77807	2.35526	20.46036	14.28590	16.01569

DRAFT, NOT FOR SUBMISSION

Appendix 18

Appendix 1

Individual Organ Weights: Terminal Day 29±1

32025165

Sex: Female Day(s) Relative to Start Date

1.49x10^12 vg Group 4	Organ Weights					
	Heart (g)	Heart (%br)	Heart (%bw)	Kidney (g)	Kidney (%br)	Kidney (%bw)
	-	-	-	-	-	-
4501	0.907	45.1468	0.3894	1.954	97.2623	0.8389
4502	1.022	47.2929	0.3853	2.329	107.7742	0.8780
4503	0.944	47.3420	0.4005	2.223	111.4845	0.9430
4504	0.996	51.9562	0.4387	1.733	90.4017	0.7633
4505	0.961	48.0500	0.3631	1.918	95.9000	0.7246
Mean	0.9660	47.95759	0.39538	2.0314	100.56452	0.82956
SD	0.0448	2.48588	0.02776	0.2414	8.76313	0.08761
N	5	5	5	5	5	5
%Diff	3.0730	-1.83214	-0.38754	10.0195	4.26572	6.15638

DRAFT, NOT FOR SUBMISSION

Appendix 18

Appendix 1

Individual Organ Weights: Terminal Day 29±1

32025165

Sex: Female Day(s) Relative to Start Date

1.49x10^12 vg Group 4	Organ Weights					
	Liver (g)	Liver (%br)	Liver (%bw)	Ovary Paired (g)	Ovary Paired (%br)	Ovary Paired (%bw)
	-	-	-	-	-	-
4501	8.651	430.6122	3.7141	0.082	4.0816	0.0352
4502	10.043	464.7385	3.7860	0.070	3.2392	0.0264
4503	10.180	510.5316	4.3184	0.106	5.3159	0.0450
4504	8.301	433.0203	3.6562	0.095	4.9557	0.0418
4505	9.441	472.0500	3.5669	0.087	4.3500	0.0329
Mean	9.3232	462.19055	3.80831	0.0880	4.38850	0.03625
SD	0.8311	32.74388	0.29625	0.0135	0.80596	0.00737
N	5	5	5	5	5	5
%Diff	2.7350	-2.24728	-0.38647	-6.3830	-11.12051	-9.26120

DRAFT, NOT FOR SUBMISSION

Appendix 18

Appendix 1

Individual Organ Weights: Terminal Day 29±1

32025165

Sex: Female Day(s) Relative to Start Date

1.49x10^12 vg Group 4	Organ Weights					
	Spleen (g)	Spleen (%br)	Spleen (%bw)	Thymus (g)	Thymus (%br)	Thymus (%bw)
	-	-	-	-	-	-
4501	0.574	28.5714	0.2464	0.450	22.3992	0.1932
4502	0.582	26.9320	0.2194	0.451	20.8700	0.1700
4503	0.554	27.7834	0.2350	0.319	15.9980	0.1353
4504	0.556	29.0037	0.2449	0.436	22.7439	0.1920
4505	0.549	27.4500	0.2074	0.497	24.8500	0.1878
Mean	0.5630	27.94808	0.23063	0.4306	21.37221	0.17567
SD	0.0142	0.83822	0.01686	0.0665	3.32286	0.02440
N	5	5	5	5	5	5
%Diff	13.9215	8.34674	10.00180	10.1842	4.45803	6.56190

DRAFT, NOT FOR SUBMISSION

Appendix 18

Appendix 1

Individual Organ Weights: Terminal Day 29±1

32025165

Sex: Female Day(s) Relative to Start Date

1.49x10^12 vg Group 4	Organ Weights		
	Uterus/ Cervix (g)	Uterus/ Cervix (%br)	Uterus/ Cervix (%bw)
	-	-	-
4501	1.234	61.4236	0.5298
4502	0.539	24.9422	0.2032
4503	0.722	36.2086	0.3063
4504	0.623	32.4987	0.2744
4505	0.500	25.0000	0.1889
Mean	0.7236	36.01461	0.30051
SD	0.2978	15.01613	0.13712
N	5	5	5
%Diff	3.6379	-2.59720	-1.02605

Appendix 18

Appendix 2

Individual Organ Weights: Terminal Day 91±2

32025165

Sex: Male Day(s) Relative to Start Date

0 vg	Organ Weights					
Group 1	Terminal Body Wt (g)	Brain (g)	Brain (%bw)	Epididymis (g)	Epididymis (%br)	Epididymis (%bw)
	-	-	-	-	-	-
1006	687.034	2.371	0.3451	1.553	65.4998	0.2260
1007	495.259	2.163	0.4367	1.696	78.4096	0.3424
1008	570.074	2.206	0.3870	1.580	71.6228	0.2772
1009	481.171	2.296	0.4772	1.583	68.9460	0.3290
1010	585.859	2.270	0.3875	1.520	66.9604	0.2594
Mean	563.8794	2.2612	0.40669	1.5864	70.28772	0.28682
SD	82.5196	0.0807	0.05104	0.0663	5.08782	0.04850
N	5	5	5	5	5	5

DRAFT, NOT FOR SUBMISSION

Appendix 18

Appendix 2

Individual Organ Weights: Terminal Day 91±2

32025165

Sex: Male Day(s) Relative to Start Date

0 vg	Organ Weights					
	Adrenal	Adrenal	Adrenal	Pituitary	Pituitary	Pituitary
Group 1	(g)	(%br)	(%bw)	(g)	(%br)	(%bw)
	-	-	-	-	-	-
1006	0.0590	2.4884	0.0086	0.0212	0.8941	0.0031
1007	0.0900	4.1609	0.0182	0.0158	0.7305	0.0032
1008	0.0560	2.5385	0.0098	0.0164	0.7434	0.0029
1009	0.0700	3.0488	0.0145	0.0165	0.7186	0.0034
1010	0.0720	3.1718	0.0123	0.0170	0.7489	0.0029
Mean	0.06940	3.08168	0.01268	0.01738	0.76711	0.00310
SD	0.01341	0.67470	0.00383	0.00218	0.07197	0.00023
N	5	5	5	5	5	5

DRAFT, NOT FOR SUBMISSION

Appendix 18

Appendix 2

Individual Organ Weights: Terminal Day 91±2

32025165

Sex: Male Day(s) Relative to Start Date

0 vg	Organ Weights					
Group 1	Prostate (g)	Prostate (%br)	Prostate (%bw)	Thyroid/ Parathyroid (g)	Thyroid/ Parathyroid (%br)	Thyroid/ Parathyroid (%bw)
	-	-	-	-	-	-
1006	1.482	62.5053	0.2157	0.0273	1.1514	0.0040
1007	1.706	78.8719	0.3445	0.0282	1.3037	0.0057
1008	1.669	75.6573	0.2928	0.0264	1.1967	0.0046
1009	1.847	80.4443	0.3839	0.0246	1.0714	0.0051
1010	0.920	40.5286	0.1570	0.0371	1.6344	0.0063
Mean	1.5248	67.60148	0.27877	0.02872	1.27154	0.00515
SD	0.3623	16.70155	0.09267	0.00487	0.21951	0.00092
N	5	5	5	5	5	5

DRAFT, NOT FOR SUBMISSION

Appendix 18

Appendix 2

Individual Organ Weights: Terminal Day 91±2

32025165

Sex: Male Day(s) Relative to Start Date

0 vg	Organ Weights					
	Heart	Heart	Heart	Kidney	Kidney	Kidney
Group 1	(g)	(%br)	(%bw)	(g)	(%br)	(%bw)
	-	-	-	-	-	-
1006	1.885	79.5023	0.2744	4.478	188.8655	0.6518
1007	1.924	88.9505	0.3885	3.864	178.6408	0.7802
1008	1.758	79.6917	0.3084	3.987	180.7344	0.6994
1009	1.418	61.7596	0.2947	3.152	137.2822	0.6551
1010	1.506	66.3436	0.2571	3.169	139.6035	0.5409
Mean	1.6982	75.24956	0.30460	3.7300	165.02527	0.66547
SD	0.2263	11.03212	0.05080	0.5684	24.57872	0.08678
N	5	5	5	5	5	5

DRAFT, NOT FOR SUBMISSION

Appendix 18

Appendix 2

Individual Organ Weights: Terminal Day 91±2

32025165

Sex: Male Day(s) Relative to Start Date

0 vg	Organ Weights					
Group 1	Liver (g)	Liver (%br)	Liver (%bw)	Spleen (g)	Spleen (%br)	Spleen (%bw)
	-	-	-	-	-	-
1006	21.841	921.1725	3.1790	0.830	35.0063	0.1208
1007	22.957	1061.3500	4.6354	0.717	33.1484	0.1448
1008	19.019	862.1487	3.3362	0.748	33.9075	0.1312
1009	12.817	558.2317	2.6637	0.776	33.7979	0.1613
1010	16.366	720.9692	2.7935	0.589	25.9471	0.1005
Mean	18.6000	824.77441	3.32157	0.7320	32.36146	0.13172
SD	4.1260	192.66420	0.78393	0.0901	3.64732	0.02311
N	5	5	5	5	5	5

DRAFT, NOT FOR SUBMISSION

Appendix 18

Appendix 2

Individual Organ Weights: Terminal Day 91±2

32025165

Sex: Male   Day(s) Relative to Start Date

0 vg	Organ Weights					
	Testis	Testis	Testis	Thymus	Thymus	Thymus
Group 1	(g)	(%br)	(%bw)	(g)	(%br)	(%bw)
	-	-	-	-	-	-
1006	3.856	162.6318	0.5613	0.255	10.7550	0.0371
1007	3.579	165.4646	0.7227	0.275	12.7138	0.0555
1008	3.790	171.8042	0.6648	0.274	12.4207	0.0481
1009	4.071	177.3084	0.8461	0.151	6.5767	0.0314
1010	3.561	156.8722	0.6078	0.131	5.7709	0.0224
Mean	3.7714	166.81624	0.68052	0.2172	9.64741	0.03889
SD	0.2113	7.95587	0.11061	0.0704	3.27021	0.01317
N	5	5	5	5	5	5

DRAFT, NOT FOR SUBMISSION

Appendix 18

Appendix 2

Individual Organ Weights: Terminal Day 91±2

32025165

Sex: Male Day(s) Relative to Start Date

1.49x10^11 vg Group 2	Organ Weights					
	Terminal Body Wt (g)	Brain (g)	Brain (%bw)	Epididymis (g)	Epididymis (%br)	Epididymis (%bw)
	-	-	-	-	-	-
2001	726.278	2.349	0.3234	1.578	67.1775	0.2173
2002	491.006	2.041	0.4157	1.922	94.1695	0.3914
2003	574.907	2.159	0.3755	1.441	66.7439	0.2506
2004	461.719	2.119	0.4589	1.415	66.7768	0.3065
2005	578.270	2.116	0.3659	1.467	69.3289	0.2537
2006	537.539	2.349	0.4370	1.718	73.1375	0.3196
2007	736.870	2.214	0.3005	1.852	83.6495	0.2513
2008	694.919	2.205	0.3173	1.536	69.6599	0.2210
2009	510.844	2.415	0.4727	1.442	59.7101	0.2823
2010	689.628	2.293	0.3325	1.729	75.4034	0.2507
Mean	600.1980	2.2260	0.37995	1.6100	72.57570	0.27445
SD	103.1047	0.1217	0.06262	0.1836	9.88027	0.05274
N	10	10	10	10	10	10
%Diff	6.4408	-1.5567	-6.57496	1.4876	3.25517	-4.31261

DRAFT, NOT FOR SUBMISSION

Appendix 18

Appendix 2

Individual Organ Weights: Terminal Day 91±2

32025165

Sex: Male Day(s) Relative to Start Date

1.49x10^11 vg Group 2	Organ Weights					
	Adrenal (g)	Adrenal (%br)	Adrenal (%bw)	Pituitary (g)	Pituitary (%br)	Pituitary (%bw)
	-	-	-	-	-	-
2001	0.0670	2.8523	0.0092	0.0177	0.7535	0.0024
2002	0.0440	2.1558	0.0090	0.0162	0.7937	0.0033
2003	0.0460	2.1306	0.0080	0.0157	0.7272	0.0027
2004	0.0380	1.7933	0.0082	0.0125	0.5899	0.0027
2005	0.0420	1.9849	0.0073	0.0174	0.8223	0.0030
2006	0.0720	3.0651	0.0134	0.0145	0.6173	0.0027
2007	0.0550	2.4842	0.0075	0.0161	0.7272	0.0022
2008	0.0430	1.9501	0.0062	0.0157	0.7120	0.0023
2009	0.0500	2.0704	0.0098	0.0157	0.6501	0.0031
2010	0.0610	2.6603	0.0088	0.0178	0.7763	0.0026
Mean	0.05180	2.31470	0.00874	0.01593	0.71695	0.00270
SD	0.01153	0.42592	0.00195	0.00159	0.07647	0.00036
N	10	10	10	10	10	10
%Diff	-25.36023	-24.88848	-31.12662	-8.34292	-6.53924	-12.87697

DRAFT, NOT FOR SUBMISSION

Appendix 18

Appendix 2

Individual Organ Weights: Terminal Day 91±2

32025165

Sex: Male Day(s) Relative to Start Date

1.49x10^11 vg Group 2	Organ Weights					
	Prostate (g)	Prostate (%br)	Prostate (%bw)	Thyroid/ Parathyroid (g)	Thyroid/ Parathyroid (%br)	Thyroid/ Parathyroid (%bw)
	-	-	-	-	-	-
2001	1.422	60.5364	0.1958	0.0329	1.4006	0.0045
2002	1.471	72.0725	0.2996	0.0278	1.3621	0.0057
2003	1.198	55.4887	0.2084	0.0291	1.3478	0.0051
2004	1.314	62.0104	0.2846	0.0255	1.2034	0.0055
2005	1.647	77.8355	0.2848	0.0341	1.6115	0.0059
2006	1.341	57.0881	0.2495	0.0230	0.9791	0.0043
2007	1.984	89.6116	0.2692	0.0213	0.9621	0.0029
2008	1.700	77.0975	0.2446	0.0388	1.7596	0.0056
2009	1.559	64.5549	0.3052	0.0307	1.2712	0.0060
2010	1.575	68.6873	0.2284	0.0334	1.4566	0.0048
Mean	1.5211	68.49829	0.25701	0.02966	1.33541	0.00503
SD	0.2257	10.75066	0.03795	0.00542	0.25031	0.00095
N	10	10	10	10	10	10
%Diff	-0.2427	1.32661	-7.80534	3.27298	5.02342	-2.34725

DRAFT, NOT FOR SUBMISSION

Appendix 18

Appendix 2

Individual Organ Weights: Terminal Day 91±2

32025165

Sex: Male Day(s) Relative to Start Date

1.49x10^11 vg Group 2	Organ Weights					
	Heart (g)	Heart (%br)	Heart (%bw)	Kidney (g)	Kidney (%br)	Kidney (%bw)
	-	-	-	-	-	-
2001	2.103	89.5275	0.2896	2.235	95.1469	0.3077
2002	1.585	77.6580	0.3228	3.390	166.0951	0.6904
2003	1.639	75.9148	0.2851	3.211	148.7263	0.5585
2004	1.399	66.0217	0.3030	3.008	141.9538	0.6515
2005	1.773	83.7902	0.3066	3.436	162.3819	0.5942
2006	1.483	63.1332	0.2759	3.601	153.2993	0.6699
2007	2.328	105.1491	0.3159	4.396	198.5547	0.5966
2008	2.020	91.6100	0.2907	4.070	184.5805	0.5857
2009	1.689	69.9379	0.3306	3.527	146.0455	0.6904
2010	1.823	79.5028	0.2643	3.336	145.4863	0.4837
Mean	1.7842	80.22451	0.29845	3.4210	154.22700	0.58287
SD	0.2913	12.80385	0.02111	0.5817	27.70715	0.11636
N	10	10	10	10	10	10
%Diff	5.0642	6.61127	-2.01729	-8.2842	-6.54340	-12.41278

DRAFT, NOT FOR SUBMISSION

Appendix 18

Appendix 2

Individual Organ Weights: Terminal Day 91±2

32025165

Sex: Male Day(s) Relative to Start Date

1.49x10^11 vg Group 2	Organ Weights					
	Liver (g)	Liver (%br)	Liver (%bw)	Spleen (g)	Spleen (%br)	Spleen (%bw)
	-	-	-	-	-	-
2001	27.983	1191.2729	3.8529	0.992	42.2307	0.1366
2002	19.491	954.9731	3.9696	0.675	33.0720	0.1375
2003	17.785	823.7610	3.0935	0.685	31.7277	0.1191
2004	15.993	754.7428	3.4638	0.738	34.8277	0.1598
2005	20.624	974.6692	3.5665	0.461	21.7864	0.0797
2006	17.658	751.7241	3.2850	0.778	33.1205	0.1447
2007	23.695	1070.2349	3.2156	1.103	49.8193	0.1497
2008	27.599	1251.6553	3.9715	1.073	48.6621	0.1544
2009	15.988	662.0290	3.1297	0.898	37.1843	0.1758
2010	22.300	972.5251	3.2336	0.660	28.7833	0.0957
Mean	20.9116	940.75873	3.47819	0.8063	36.12140	0.13531
SD	4.4133	194.43763	0.34461	0.2055	8.70492	0.02949
N	10	10	10	10	10	10
%Diff	12.4280	14.06255	4.71528	10.1503	11.61857	2.72406

DRAFT, NOT FOR SUBMISSION

Appendix 18

Appendix 2

Individual Organ Weights: Terminal Day 91±2

32025165

Sex: Male Day(s) Relative to Start Date

1.49x10^11 vg Group 2	Organ Weights Testis (g)	Testis (%br)	Testis (%bw)	Thymus (g)	Thymus (%br)	Thymus (%bw)
	-	-	-	-	-	-
2001	3.626	154.3636	0.4993	0.666	28.3525	0.0917
2002	3.509	171.9255	0.7147	0.322	15.7766	0.0656
2003	3.348	155.0718	0.5824	0.481	22.2788	0.0837
2004	3.443	162.4823	0.7457	0.450	21.2364	0.0975
2005	3.834	181.1909	0.6630	0.341	16.1153	0.0590
2006	3.424	145.7642	0.6370	0.339	14.4317	0.0631
2007	4.918	222.1319	0.6674	0.311	14.0470	0.0422
2008	4.065	184.3537	0.5850	0.427	19.3651	0.0614
2009	3.892	161.1594	0.7619	0.238	9.8551	0.0466
2010	3.296	143.7418	0.4779	0.317	13.8247	0.0460
Mean	3.7355	168.21851	0.63341	0.3892	17.52831	0.06566
SD	0.4865	23.33300	0.09711	0.1219	5.33566	0.01939
N	10	10	10	10	10	10
%Diff	-0.9519	0.84061	-6.92251	79.1897	81.68939	68.84918

DRAFT, NOT FOR SUBMISSION

Appendix 18

Appendix 2

Individual Organ Weights: Terminal Day 91±2

32025165

Sex: Male Day(s) Relative to Start Date

4.47x10^11 vg Group 3	Organ Weights					
	Terminal Body Wt (g)	Brain (g)	Brain (%bw)	Epididymis (g)	Epididymis (%br)	Epididymis (%bw)
	-	-	-	-	-	-
3001	558.783	2.257	0.4039	1.420	62.9154	0.2541
3002	532.508	2.352	0.4417	1.437	61.0969	0.2699
3003	528.975	2.216	0.4189	1.536	69.3141	0.2904
3004	670.315	2.355	0.3513	1.644	69.8089	0.2453
3005	660.375	2.254	0.3413	1.753	77.7728	0.2655
3006	613.127	2.406	0.3924	1.984	82.4605	0.3236
3007	541.140	2.344	0.4332	1.572	67.0648	0.2905
3008	506.451	2.065	0.4077	1.542	74.6731	0.3045
3009	591.427	2.160	0.3652	1.621	75.0463	0.2741
3010	654.198	2.206	0.3372	1.653	74.9320	0.2527
Mean	585.7299	2.2615	0.38929	1.6162	71.50849	0.27704
SD	60.7826	0.1048	0.03822	0.1635	6.69272	0.02495
N	10	10	10	10	10	10
%Diff	3.8750	0.0133	-4.27824	1.8785	1.73683	-3.40951

DRAFT, NOT FOR SUBMISSION

Appendix 18

Appendix 2

Individual Organ Weights: Terminal Day 91±2

32025165

Sex: Male Day(s) Relative to Start Date

4.47x10^11 vg Group 3	Organ Weights					
	Adrenal (g)	Adrenal (%br)	Adrenal (%bw)	Pituitary (g)	Pituitary (%br)	Pituitary (%bw)
	-	-	-	-	-	-
3001	0.0560	2.4812	0.0100	0.0143	0.6336	0.0026
3002	0.0390	1.6582	0.0073	0.0135	0.5740	0.0025
3003	0.0580	2.6173	0.0110	-	-	-
3004	0.0590	2.5053	0.0088	0.0184	0.7813	0.0027
3005	0.0490	2.1739	0.0074	0.0164	0.7276	0.0025
3006	0.0520	2.1613	0.0085	0.0165	0.6858	0.0027
3007	0.0560	2.3891	0.0103	0.0162	0.6911	0.0030
3008	0.0400	1.9370	0.0079	0.0146	0.7070	0.0029
3009	0.0580	2.6852	0.0098	0.0149	0.6898	0.0025
3010	0.0450	2.0399	0.0069	0.0161	0.7298	0.0025
Mean	0.05120	2.26483	0.00879	0.01566	0.69112	0.00265
SD	0.00758	0.32763	0.00143	0.00148	0.05946	0.00019
N	10	10	10	9	9	9
%Diff	-26.22478	-26.50653	-30.66521	-9.92200	-9.90692	-14.35165

DRAFT, NOT FOR SUBMISSION

Appendix 18

Appendix 2

Individual Organ Weights: Terminal Day 91±2

32025165

Sex: Male Day(s) Relative to Start Date

4.47x10^11 vg Group 3	Organ Weights					
	Prostate (g)	Prostate (%br)	Prostate (%bw)	Thyroid/ Parathyroid (g)	Thyroid/ Parathyroid (%br)	Thyroid/ Parathyroid (%bw)
	-	-	-	-	-	-
3001	0.849	37.6163	0.1519	0.0433	1.9185	0.0077
3002	1.426	60.6293	0.2678	0.0272	1.1565	0.0051
3003	1.598	72.1119	0.3021	0.0242	1.0921	0.0046
3004	1.749	74.2675	0.2609	0.0274	1.1635	0.0041
3005	1.774	78.7045	0.2686	0.0218	0.9672	0.0033
3006	1.909	79.3433	0.3114	0.0246	1.0224	0.0040
3007	1.555	66.3396	0.2874	0.0218	0.9300	0.0040
3008	1.846	89.3947	0.3645	0.0185	0.8959	0.0037
3009	1.227	56.8056	0.2075	0.0369	1.7083	0.0062
3010	1.433	64.9592	0.2190	0.0345	1.5639	0.0053
Mean	1.5366	68.01718	0.26411	0.02802	1.24183	0.00480
SD	0.3217	14.42217	0.05978	0.00782	0.35832	0.00135
N	10	10	10	10	10	10
%Diff	0.7739	0.61494	-5.25787	-2.43733	-2.33661	-6.72087

DRAFT, NOT FOR SUBMISSION

Appendix 18

Appendix 2

Individual Organ Weights: Terminal Day 91±2

32025165

Sex: Male Day(s) Relative to Start Date

4.47x10^11 vg Group 3	Organ Weights					
	Heart (g)	Heart (%br)	Heart (%bw)	Kidney (g)	Kidney (%br)	Kidney (%bw)
	-	-	-	-	-	-
3001	1.709	75.7200	0.3058	3.525	156.1808	0.6308
3002	1.615	68.6650	0.3033	3.498	148.7245	0.6569
3003	1.519	68.5469	0.2872	3.496	157.7617	0.6609
3004	1.932	82.0382	0.2882	4.369	185.5202	0.6518
3005	1.833	81.3221	0.2776	3.817	169.3434	0.5780
3006	1.769	73.5245	0.2885	4.165	173.1089	0.6793
3007	1.703	72.6536	0.3147	3.371	143.8140	0.6229
3008	1.440	69.7337	0.2843	3.302	159.9031	0.6520
3009	1.847	85.5093	0.3123	3.183	147.3611	0.5382
3010	1.871	84.8141	0.2860	3.765	170.6709	0.5755
Mean	1.7238	76.25274	0.29479	3.6491	161.23886	0.62464
SD	0.1596	6.65235	0.01300	0.3805	13.23888	0.04586
N	10	10	10	10	10	10
%Diff	1.5075	1.33313	-3.21892	-2.1689	-2.29444	-6.13622

DRAFT, NOT FOR SUBMISSION

Appendix 18

Appendix 2

Individual Organ Weights: Terminal Day 91±2

32025165

Sex: Male Day(s) Relative to Start Date

4.47x10^11 vg Group 3	Organ Weights					
	Liver (g)	Liver (%br)	Liver (%bw)	Spleen (g)	Spleen (%br)	Spleen (%bw)
	-	-	-	-	-	-
3001	18.208	806.7346	3.2585	0.959	42.4900	0.1716
3002	17.432	741.1565	3.2736	0.725	30.8248	0.1361
3003	16.074	725.3610	3.0387	0.706	31.8592	0.1335
3004	24.158	1025.8174	3.6040	0.811	34.4374	0.1210
3005	26.009	1153.9042	3.9385	0.850	37.7107	0.1287
3006	18.455	767.0407	3.0100	0.995	41.3549	0.1623
3007	18.326	781.8259	3.3866	0.830	35.4096	0.1534
3008	18.459	893.8983	3.6448	0.808	39.1283	0.1595
3009	19.698	911.9444	3.3306	0.745	34.4907	0.1260
3010	23.023	1043.6537	3.5193	0.561	25.4306	0.0858
Mean	19.9842	885.13367	3.40044	0.7990	35.31364	0.13779
SD	3.2559	147.18669	0.28468	0.1254	5.15866	0.02515
N	10	10	10	10	10	10
%Diff	7.4419	7.31828	2.37476	9.1530	9.12251	4.60518

DRAFT, NOT FOR SUBMISSION

Appendix 18

Appendix 2

Individual Organ Weights: Terminal Day 91±2

32025165

Sex: Male Day(s) Relative to Start Date

4.47x10^11 vg Group 3	Organ Weights					
	Testis (g)	Testis (%br)	Testis (%bw)	Thymus (g)	Thymus (%br)	Thymus (%bw)
	-	-	-	-	-	-
3001	3.733	165.3965	0.6681	0.394	17.4568	0.0705
3002	4.057	172.4915	0.7619	0.431	18.3248	0.0809
3003	3.943	177.9332	0.7454	0.495	22.3375	0.0936
3004	3.740	158.8110	0.5579	0.484	20.5520	0.0722
3005	4.715	209.1837	0.7140	0.572	25.3771	0.0866
3006	3.919	162.8845	0.6392	0.462	19.2020	0.0754
3007	3.549	151.4078	0.6558	0.311	13.2679	0.0575
3008	3.708	179.5642	0.7322	0.182	8.8136	0.0359
3009	3.798	175.8333	0.6422	0.226	10.4630	0.0382
3010	3.819	173.1188	0.5838	0.243	11.0154	0.0371
Mean	3.8981	172.66245	0.67004	0.3800	16.68101	0.06480
SD	0.3200	15.67563	0.06819	0.1320	5.54348	0.02140
N	10	10	10	10	10	10
%Diff	3.3595	3.50458	-1.54078	74.9540	72.90674	66.61576

DRAFT, NOT FOR SUBMISSION

Appendix 18

Appendix 2

Individual Organ Weights: Terminal Day 91±2

32025165

Sex: Male Day(s) Relative to Start Date

1.49x10^12 vg Group 4	Organ Weights					
	Terminal Body Wt (g)	Brain (g)	Brain (%bw)	Epididymis (g)	Epididymis (%br)	Epididymis (%bw)
	-	-	-	-	-	-
4006	608.208	2.281	0.3750	1.823	79.9211	0.2997
4007	537.749	2.185	0.4063	1.703	77.9405	0.3167
4008	611.111	2.290	0.3747	1.677	73.2314	0.2744
4009	602.711	2.380	0.3949	1.820	76.4706	0.3020
4010	698.651	2.322	0.3324	1.803	77.6486	0.2581
4011	713.156	2.344	0.3287	1.621	69.1553	0.2273
4012	607.966	2.358	0.3879	1.603	67.9813	0.2637
4013	524.331	2.164	0.4127	1.121	51.8022	0.2138
4014	513.306	2.170	0.4227	1.500	69.1244	0.2922
4015	483.661	2.048	0.4234	1.386	67.6758	0.2866
Mean	590.0850	2.2542	0.38588	1.6057	71.09513	0.27344
SD	76.3519	0.1074	0.03395	0.2214	8.18138	0.03320
N	10	10	10	10	10	10
%Diff	4.6474	-0.3096	-5.11794	1.2166	1.14872	-4.66297

DRAFT, NOT FOR SUBMISSION

Appendix 18

Appendix 2

Individual Organ Weights: Terminal Day 91±2

32025165

Sex: Male Day(s) Relative to Start Date

1.49x10^12 vg Group 4	Organ Weights					
	Adrenal (g)	Adrenal (%br)	Adrenal (%bw)	Pituitary (g)	Pituitary (%br)	Pituitary (%bw)
	-	-	-	-	-	-
4006	0.0530	2.3235	0.0087	0.0171	0.7497	0.0028
4007	0.0370	1.6934	0.0069	0.0146	0.6682	0.0027
4008	0.0450	1.9651	0.0074	0.0174	0.7598	0.0028
4009	0.0580	2.4370	0.0096	0.0183	0.7689	0.0030
4010	0.0610	2.6270	0.0087	0.0155	0.6675	0.0022
4011	0.0740	3.1570	0.0104	0.0175	0.7466	0.0025
4012	0.0670	2.8414	0.0110	0.0168	0.7125	0.0028
4013	0.0600	2.7726	0.0114	0.0157	0.7255	0.0030
4014	0.0610	2.8111	0.0119	0.0139	0.6406	0.0027
4015	0.0330	1.6113	0.0068	0.0145	0.7080	0.0030
Mean	0.05490	2.42394	0.00929	0.01613	0.71472	0.00275
SD	0.01301	0.52060	0.00188	0.00150	0.04380	0.00026
N	10	10	10	10	10	10
%Diff	-20.89337	-21.34355	-26.79111	-7.19217	-6.82941	-11.04792

DRAFT, NOT FOR SUBMISSION

Appendix 18

Appendix 2

Individual Organ Weights: Terminal Day 91±2

32025165

Sex: Male Day(s) Relative to Start Date

1.49x10^12 vg Group 4	Organ Weights					
	Prostate (g)	Prostate (%br)	Prostate (%bw)	Thyroid/ Parathyroid (g)	Thyroid/ Parathyroid (%br)	Thyroid/ Parathyroid (%bw)
	-	-	-	-	-	-
4006	1.849	81.0609	0.3040	0.0357	1.5651	0.0059
4007	1.093	50.0229	0.2033	0.0286	1.3089	0.0053
4008	1.462	63.8428	0.2392	0.0304	1.3275	0.0050
4009	1.549	65.0840	0.2570	0.0273	1.1471	0.0045
4010	1.600	68.9061	0.2290	0.0274	1.1800	0.0039
4011	1.679	71.6297	0.2354	0.0309	1.3183	0.0043
4012	1.526	64.7159	0.2510	0.0337	1.4292	0.0055
4013	1.245	57.5323	0.2374	0.0236	1.0906	0.0045
4014	1.171	53.9631	0.2281	0.0316	1.4562	0.0062
4015	1.100	53.7109	0.2274	0.0294	1.4355	0.0061
Mean	1.4274	63.04687	0.24120	0.02986	1.32584	0.00512
SD	0.2613	9.48554	0.02644	0.00345	0.15125	0.00079
N	10	10	10	10	10	10
%Diff	-6.3877	-6.73743	-13.47766	3.96936	4.27062	-0.50800

DRAFT, NOT FOR SUBMISSION

Appendix 18

Appendix 2

Individual Organ Weights: Terminal Day 91±2

32025165

Sex: Male Day(s) Relative to Start Date

1.49x10^12 vg Group 4	Organ Weights					
	Heart (g)	Heart (%br)	Heart (%bw)	Kidney (g)	Kidney (%br)	Kidney (%bw)
	-	-	-	-	-	-
4006	2.265	99.2986	0.3724	4.082	178.9566	0.6712
4007	1.791	81.9680	0.3331	3.969	181.6476	0.7381
4008	1.955	85.3712	0.3199	4.249	185.5459	0.6953
4009	1.608	67.5630	0.2668	3.925	164.9160	0.6512
4010	1.643	70.7580	0.2352	3.697	159.2162	0.5292
4011	2.092	89.2491	0.2933	4.065	173.4215	0.5700
4012	1.985	84.1815	0.3265	3.771	159.9237	0.6203
4013	1.995	92.1904	0.3805	3.821	176.5712	0.7287
4014	1.299	59.8618	0.2531	2.792	128.6636	0.5439
4015	1.466	71.5820	0.3031	3.553	173.4863	0.7346
Mean	1.8099	80.20235	0.30838	3.7924	168.23485	0.64824
SD	0.3020	12.35084	0.04804	0.4061	16.48505	0.07939
N	10	10	10	10	10	10
%Diff	6.5776	6.58182	1.24269	1.6729	1.94490	-2.58857

DRAFT, NOT FOR SUBMISSION

Appendix 18

Appendix 2

Individual Organ Weights: Terminal Day 91±2

32025165

Sex: Male Day(s) Relative to Start Date

1.49x10^12 vg Group 4	Organ Weights					
	Liver (g)	Liver (%br)	Liver (%bw)	Spleen (g)	Spleen (%br)	Spleen (%bw)
	-	-	-	-	-	-
4006	25.521	1118.8514	4.1961	1.056	46.2955	0.1736
4007	20.419	934.5080	3.7971	0.702	32.1281	0.1305
4008	21.727	948.7773	3.5553	0.856	37.3799	0.1401
4009	42.156	1771.2605	6.9944	1.186	49.8319	0.1968
4010	22.616	973.9879	3.2371	0.863	37.1662	0.1235
4011	22.472	958.7031	3.1511	0.887	37.8413	0.1244
4012	21.582	915.2672	3.5499	1.014	43.0025	0.1668
4013	10.195	471.1183	1.9444	0.847	39.1405	0.1615
4014	16.666	768.0184	3.2468	0.907	41.7972	0.1767
4015	17.725	865.4785	3.6648	0.738	36.0352	0.1526
Mean	22.1079	972.59706	3.73369	0.9056	40.06184	0.15465
SD	8.2191	328.22100	1.28863	0.1455	5.23330	0.02472
N	10	10	10	10	10	10
%Diff	18.8597	17.92280	12.40757	23.7158	23.79492	17.40990

DRAFT, NOT FOR SUBMISSION

Appendix 18

Appendix 2

Individual Organ Weights: Terminal Day 91±2

32025165

Sex: Male Day(s) Relative to Start Date

1.49x10^12 vg Group 4	Organ Weights					
	Testis (g)	Testis (%br)	Testis (%bw)	Thymus (g)	Thymus (%br)	Thymus (%bw)
	-	-	-	-	-	-
4006	3.792	166.2429	0.6235	0.323	14.1605	0.0531
4007	3.697	169.1991	0.6875	0.305	13.9588	0.0567
4008	4.086	178.4279	0.6686	0.576	25.1528	0.0943
4009	4.433	186.2605	0.7355	0.294	12.3529	0.0488
4010	3.710	159.7761	0.5310	0.401	17.2696	0.0574
4011	3.611	154.0529	0.5063	0.674	28.7543	0.0945
4012	3.663	155.3435	0.6025	0.242	10.2629	0.0398
4013	2.980	137.7079	0.5683	0.375	17.3290	0.0715
4014	3.368	155.2074	0.6561	0.435	20.0461	0.0847
4015	3.065	149.6582	0.6337	0.242	11.8164	0.0500
Mean	3.6405	161.18764	0.62132	0.3867	17.11034	0.06509
SD	0.4347	14.23085	0.07115	0.1423	6.01049	0.01986
N	10	10	10	10	10	10
%Diff	-3.4709	-3.37413	-8.70044	78.0387	77.35685	67.36275

DRAFT, NOT FOR SUBMISSION

Appendix 18

Appendix 2

Individual Organ Weights: Terminal Day 91±2

32025165

Sex: Female Day(s) Relative to Start Date

0 vg<br>Group 1	Organ Weights					
	Terminal Body Wt (g)	Brain (g)	Brain (%bw)	Adrenal (g)	Adrenal (%br)	Adrenal (%bw)
	-	-	-	-	-	-
1506	270.546	1.987	0.7344	0.0510	2.5667	0.0189
1507	300.260	1.933	0.6438	0.0580	3.0005	0.0193
1508	233.683	1.951	0.8349	0.0640	3.2804	0.0274
1509	267.926	2.032	0.7584	0.0610	3.0020	0.0228
1510	278.701	1.969	0.7065	0.0450	2.2854	0.0161
Mean	270.2232	1.9744	0.73560	0.05580	2.82699	0.02089
SD	24.0554	0.0380	0.07011	0.00773	0.39602	0.00433
N	5	5	5	5	5	5

DRAFT, NOT FOR SUBMISSION

Appendix 18

Appendix 2

Individual Organ Weights: Terminal Day 91±2

32025165

Sex: Female Day(s) Relative to Start Date

0 vg	Organ Weights					
Group 1	Pituitary (g)	Pituitary (%br)	Pituitary (%bw)	Thyroid/ Parathyroid (g)	Thyroid/ Parathyroid (%br)	Thyroid/ Parathyroid (%bw)
	-	-	-	-	-	-
1506	0.0092	0.4630	0.0034	0.0256	1.2884	0.0095
1507	0.0173	0.8950	0.0058	0.0219	1.1330	0.0073
1508	0.0228	1.1686	0.0098	0.0250	1.2814	0.0107
1509	0.0222	1.0925	0.0083	0.0236	1.1614	0.0088
1510	0.0192	0.9751	0.0069	0.0157	0.7974	0.0056
Mean	0.01814	0.91885	0.00682	0.02236	1.13230	0.00838
SD	0.00548	0.27572	0.00243	0.00399	0.19976	0.00197
N	5	5	5	5	5	5

DRAFT, NOT FOR SUBMISSION

Appendix 18

Appendix 2

Individual Organ Weights: Terminal Day 91±2

32025165

Sex: Female Day(s) Relative to Start Date

0 vg	Organ Weights					
	Heart	Heart	Heart	Kidney	Kidney	Kidney
Group 1	(g)	(%br)	(%bw)	(g)	(%br)	(%bw)
	-	-	-	-	-	-
1506	1.020	51.3337	0.3770	1.981	99.6980	0.7322
1507	1.060	54.8370	0.3530	2.288	118.3652	0.7620
1508	1.065	54.5874	0.4557	1.893	97.0272	0.8101
1509	0.902	44.3898	0.3367	2.018	99.3110	0.7532
1510	1.051	53.3773	0.3771	2.164	109.9035	0.7765
Mean	1.0196	51.70504	0.37991	2.0688	104.86099	0.76679
SD	0.0680	4.31756	0.04572	0.1568	9.03630	0.02902
N	5	5	5	5	5	5

DRAFT, NOT FOR SUBMISSION

Appendix 18

Appendix 2

Individual Organ Weights: Terminal Day 91±2

32025165

Sex: Female Day(s) Relative to Start Date

0 vg	Organ Weights					
Group 1	Liver (g)	Liver (%br)	Liver (%bw)	Ovary Paired (g)	Ovary Paired (%br)	Ovary Paired (%bw)
	-	-	-	-	-	-
1506	11.370	572.2194	4.2026	0.085	4.2778	0.0314
1507	11.646	602.4832	3.8786	0.084	4.3456	0.0280
1508	7.671	393.1830	3.2827	0.096	4.9206	0.0411
1509	9.104	448.0315	3.3980	0.064	3.1496	0.0239
1510	9.116	462.9761	3.2709	0.083	4.2153	0.0298
Mean	9.7814	495.77864	3.60655	0.0824	4.18178	0.03083
SD	1.6849	88.19115	0.41546	0.0115	0.64194	0.00638
N	5	5	5	5	5	5

DRAFT, NOT FOR SUBMISSION

Appendix 18

Appendix 2

Individual Organ Weights: Terminal Day 91±2

32025165

Sex: Female Day(s) Relative to Start Date

0 vg	Organ Weights					
	Spleen	Spleen	Spleen	Thymus	Thymus	Thymus
Group 1	(g)	(%br)	(%bw)	(g)	(%br)	(%bw)
	-	-	-	-	-	-
1506	0.474	23.8551	0.1752	0.199	10.0151	0.0736
1507	0.596	30.8329	0.1985	0.361	18.6756	0.1202
1508	0.462	23.6802	0.1977	0.130	6.6632	0.0556
1509	0.422	20.7677	0.1575	0.274	13.4843	0.1023
1510	0.464	23.5653	0.1665	0.270	13.7125	0.0969
Mean	0.4836	24.54022	0.17908	0.2468	12.51016	0.08971
SD	0.0659	3.74130	0.01846	0.0870	4.49498	0.02531
N	5	5	5	5	5	5

DRAFT, NOT FOR SUBMISSION

Appendix 18

Appendix 2

Individual Organ Weights: Terminal Day 91±2

32025165

Sex: Female Day(s) Relative to Start Date

0 vg	Organ Weights		
Group 1	Uterus/ Cervix (g)	Uterus/ Cervix (%br)	Uterus/ Cervix (%bw)
	-	-	-
1506	1.064	53.5481	0.3933
1507	0.822	42.5246	0.2738
1508	0.798	40.9021	0.3415
1509	1.173	57.7264	0.4378
1510	0.621	31.5389	0.2228
Mean	0.8956	45.24799	0.33383
SD	0.2212	10.47432	0.08709
N	5	5	5

DRAFT, NOT FOR SUBMISSION

Appendix 18

Appendix 2

Individual Organ Weights: Terminal Day 91±2

32025165

Sex: Female Day(s) Relative to Start Date

1.49x10^11 vg Group 2	Organ Weights					
	Terminal Body Wt (g)	Brain (g)	Brain (%bw)	Adrenal (g)	Adrenal (%br)	Adrenal (%bw)
	-	-	-	-	-	-
2501	277.948	1.822	0.6555	0.0660	3.6224	0.0237
2502	282.576	1.746	0.6179	0.0620	3.5510	0.0219
2503	313.962	1.984	0.6319	0.0590	2.9738	0.0188
2504	271.908	2.064	0.7591	0.0640	3.1008	0.0235
2505	275.665	1.911	0.6932	0.0580	3.0351	0.0210
2506	366.103	2.266	0.6190	0.0610	2.6920	0.0167
2507	279.717	1.978	0.7071	0.0490	2.4772	0.0175
2508	300.371	1.986	0.6612	0.0570	2.8701	0.0190
2509	283.595	2.130	0.7511	0.0700	3.2864	0.0247
2510	290.003	2.009	0.6928	0.0730	3.6336	0.0252
Mean	294.1848	1.9896	0.67887	0.06190	3.12423	0.02121
SD	28.2415	0.1475	0.05079	0.00687	0.39683	0.00308
N	10	10	10	10	10	10
%Diff	8.8673	0.7699	-7.71196	10.93190	10.51439	1.49805

DRAFT, NOT FOR SUBMISSION

Appendix 18

Appendix 2

Individual Organ Weights: Terminal Day 91±2

32025165

Sex: Female Day(s) Relative to Start Date

1.49x10^11 vg Group 2	Organ Weights					
	Pituitary (g)	Pituitary (%br)	Pituitary (%bw)	Thyroid/ Parathyroid (g)	Thyroid/ Parathyroid (%br)	Thyroid/ Parathyroid (%bw)
	-	-	-	-	-	-
2501	0.0203	1.1142	0.0073	0.0187	1.0263	0.0067
2502	0.0167	0.9565	0.0059	0.0227	1.3001	0.0080
2503	0.0221	1.1139	0.0070	0.0240	1.2097	0.0076
2504	0.0200	0.9690	0.0074	0.0194	0.9399	0.0071
2505	0.0191	0.9995	0.0069	0.0226	1.1826	0.0082
2506	0.0305	1.3460	0.0083	0.0413	1.8226	0.0113
2507	0.0249	1.2588	0.0089	0.0183	0.9252	0.0065
2508	0.0209	1.0524	0.0070	0.0245	1.2336	0.0082
2509	0.0185	0.8685	0.0065	0.0219	1.0282	0.0077
2510	0.0196	0.9756	0.0068	0.0214	1.0652	0.0074
Mean	0.02126	1.06544	0.00720	0.02348	1.17335	0.00788
SD	0.00391	0.14658	0.00086	0.00661	0.26100	0.00132
N	10	10	10	10	10	10
%Diff	17.19956	15.95308	5.60438	5.00894	3.62511	-5.93380

DRAFT, NOT FOR SUBMISSION

Appendix 18

Appendix 2

Individual Organ Weights: Terminal Day 91±2

32025165

Sex: Female Day(s) Relative to Start Date

1.49x10^11 vg Group 2	Organ Weights					
	Heart (g)	Heart (%br)	Heart (%bw)	Kidney (g)	Kidney (%br)	Kidney (%bw)
	-	-	-	-	-	-
2501	0.991	54.3908	0.3565	2.139	117.3985	0.7696
2502	1.101	63.0584	0.3896	2.046	117.1821	0.7241
2503	1.216	61.2903	0.3873	2.293	115.5746	0.7303
2504	0.926	44.8643	0.3406	1.932	93.6047	0.7105
2505	0.911	47.6714	0.3305	2.210	115.6463	0.8017
2506	1.289	56.8844	0.3521	2.508	110.6796	0.6851
2507	1.051	53.1345	0.3757	1.963	99.2417	0.7018
2508	1.194	60.1208	0.3975	2.441	122.9104	0.8127
2509	0.974	45.7277	0.3434	1.955	91.7840	0.6894
2510	1.009	50.2240	0.3479	1.929	96.0179	0.6652
Mean	1.0662	53.73666	0.36212	2.1416	108.00397	0.72902
SD	0.1295	6.56080	0.02352	0.2151	11.58756	0.05013
N	10	10	10	10	10	10
%Diff	4.5704	3.92925	-4.68252	3.5189	2.99728	-4.92554

DRAFT, NOT FOR SUBMISSION

Appendix 18

Appendix 2

Individual Organ Weights: Terminal Day 91±2

32025165

Sex: Female Day(s) Relative to Start Date

1.49x10^11 vg Group 2	Organ Weights					
	Liver (g)	Liver (%br)	Liver (%bw)	Ovary Paired (g)	Ovary Paired (%br)	Ovary Paired (%bw)
	-	-	-	-	-	-
2501	11.014	604.5005	3.9626	0.084	4.6103	0.0302
2502	10.559	604.7537	3.7367	0.091	5.2119	0.0322
2503	10.729	540.7762	3.4173	0.090	4.5363	0.0287
2504	10.426	505.1357	3.8344	0.094	4.5543	0.0346
2505	10.259	536.8394	3.7215	0.091	4.7619	0.0330
2506	14.600	644.3071	3.9879	0.063	2.7802	0.0172
2507	9.353	472.8514	3.3437	0.038	1.9211	0.0136
2508	11.496	578.8520	3.8273	0.123	6.1934	0.0409
2509	10.145	476.2911	3.5773	0.080	3.7559	0.0282
2510	9.646	480.1394	3.3262	0.109	5.4256	0.0376
Mean	10.8227	544.44464	3.67349	0.0863	4.37509	0.02962
SD	1.4650	61.39349	0.24553	0.0233	1.26147	0.00849
N	10	10	10	10	10	10
%Diff	10.6457	9.81607	1.85620	4.7330	4.62267	-3.91709

DRAFT, NOT FOR SUBMISSION

Appendix 18

Appendix 2

Individual Organ Weights: Terminal Day 91±2

32025165

Sex: Female Day(s) Relative to Start Date

1.49x10^11 vg Group 2	Organ Weights					
	Spleen (g)	Spleen (%br)	Spleen (%bw)	Thymus (g)	Thymus (%br)	Thymus (%bw)
	-	-	-	-	-	-
2501	0.393	21.5697	0.1414	0.258	14.1603	0.0928
2502	0.464	26.5750	0.1642	0.335	19.1867	0.1186
2503	0.585	29.4859	0.1863	0.252	12.7016	0.0803
2504	0.627	30.3779	0.2306	0.279	13.5174	0.1026
2505	0.500	26.1643	0.1814	0.400	20.9314	0.1451
2506	0.548	24.1836	0.1497	0.291	12.8420	0.0795
2507	0.713	36.0465	0.2549	0.261	13.1951	0.0933
2508	0.572	28.8016	0.1904	0.345	17.3716	0.1149
2509	0.494	23.1925	0.1742	0.263	12.3474	0.0927
2510	0.380	18.9149	0.1310	0.304	15.1319	0.1048
Mean	0.5276	26.53119	0.18041	0.2988	15.13856	0.10246
SD	0.1032	4.92886	0.03860	0.0480	3.00549	0.01980
N	10	10	10	10	10	10
%Diff	9.0984	8.11309	0.74571	21.0697	21.01014	14.20638

DRAFT, NOT FOR SUBMISSION

Appendix 18

Appendix 2

Individual Organ Weights: Terminal Day 91±2

32025165

Sex: Female Day(s) Relative to Start Date

1.49x10^11 vg Group 2	Organ Weights		
	Uterus/ Cervix (g)	Uterus/ Cervix (%br)	Uterus/ Cervix (%bw)
	-	-	-
2501	0.815	44.7311	0.2932
2502	0.638	36.5407	0.2258
2503	0.753	37.9536	0.2398
2504	0.729	35.3198	0.2681
2505	1.283	67.1376	0.4654
2506	0.910	40.1589	0.2486
2507	0.790	39.9393	0.2824
2508	1.038	52.2659	0.3456
2509	0.764	35.8685	0.2694
2510	0.925	46.0428	0.3190
Mean	0.8645	43.59582	0.29573
SD	0.1859	9.84129	0.06968
N	10	10	10
%Diff	-3.4725	-3.65138	-11.41368

DRAFT, NOT FOR SUBMISSION

Appendix 18

Appendix 2

Individual Organ Weights: Terminal Day 91±2

32025165

Sex: Female Day(s) Relative to Start Date

4.47x10^11 vg Group 3	Organ Weights					
	Terminal Body Wt (g)	Brain (g)	Brain (%bw)	Adrenal (g)	Adrenal (%br)	Adrenal (%bw)
	-	-	-	-	-	-
3501	365.226	2.270	0.6215	0.0880	3.8767	0.0241
3502	285.309	1.866	0.6540	0.0660	3.5370	0.0231
3503	301.362	2.083	0.6912	0.0530	2.5444	0.0176
3504	270.674	2.054	0.7588	0.0670	3.2619	0.0248
3505	272.419	2.019	0.7411	0.0950	4.7053	0.0349
3506	270.652	1.899	0.7016	0.0900	4.7393	0.0333
3507	289.018	1.991	0.6889	0.0800	4.0181	0.0277
3508	293.553	1.970	0.6711	0.0660	3.3503	0.0225
3509	242.043	1.922	0.7941	0.0630	3.2778	0.0260
3510	253.701	2.022	0.7970	0.0760	3.7587	0.0300
Mean	284.3957	2.0096	0.71194	0.07440	3.70694	0.02638
SD	33.6386	0.1144	0.05897	0.01362	0.67335	0.00522
N	10	10	10	10	10	10
%Diff	5.2447	1.7828	-3.21653	33.33333	31.12672	26.27761

DRAFT, NOT FOR SUBMISSION

Appendix 18

Appendix 2

Individual Organ Weights: Terminal Day 91±2

32025165

Sex: Female Day(s) Relative to Start Date

4.47x10^11 vg Group 3	Organ Weights					
	Pituitary (g)	Pituitary (%br)	Pituitary (%bw)	Thyroid/ Parathyroid (g)	Thyroid/ Parathyroid (%br)	Thyroid/ Parathyroid (%bw)
	-	-	-	-	-	-
3501	0.0366	1.6123	0.0100	0.0261	1.1498	0.0071
3502	0.0220	1.1790	0.0077	0.0228	1.2219	0.0080
3503	0.0188	0.9025	0.0062	0.0251	1.2050	0.0083
3504	0.0214	1.0419	0.0079	0.0264	1.2853	0.0098
3505	0.0201	0.9955	0.0074	0.0216	1.0698	0.0079
3506	0.0256	1.3481	0.0095	0.0227	1.1954	0.0084
3507	0.0367	1.8433	0.0127	0.0215	1.0799	0.0074
3508	0.0207	1.0508	0.0071	0.0199	1.0102	0.0068
3509	0.0162	0.8429	0.0067	0.0165	0.8585	0.0068
3510	0.0187	0.9248	0.0074	0.0191	0.9446	0.0075
Mean	0.02368	1.17411	0.00825	0.02217	1.10202	0.00781
SD	0.00726	0.33004	0.00195	0.00316	0.13483	0.00089
N	10	10	10	10	10	10
%Diff	30.54024	27.78037	21.02906	-0.84973	-2.67384	-6.79350

DRAFT, NOT FOR SUBMISSION

Appendix 18

Appendix 2

Individual Organ Weights: Terminal Day 91±2

32025165

Sex: Female Day(s) Relative to Start Date

4.47x10^11 vg Group 3	Organ Weights					
	Heart (g)	Heart (%br)	Heart (%bw)	Kidney (g)	Kidney (%br)	Kidney (%bw)
	-	-	-	-	-	-
3501	1.420	62.5551	0.3888	2.706	119.2070	0.7409
3502	1.027	55.0375	0.3600	2.087	111.8435	0.7315
3503	1.272	61.0658	0.4221	2.201	105.6649	0.7304
3504	0.949	46.2025	0.3506	2.033	98.9776	0.7511
3505	1.099	54.4329	0.4034	1.798	89.0540	0.6600
3506	1.069	56.2928	0.3950	2.026	106.6877	0.7486
3507	1.110	55.7509	0.3841	2.179	109.4425	0.7539
3508	0.998	50.6599	0.3400	2.321	117.8173	0.7907
3509	0.908	47.2425	0.3751	1.628	84.7034	0.6726
3510	1.046	51.7310	0.4123	2.315	114.4906	0.9125
Mean	1.0898	54.09707	0.38313	2.1294	105.78886	0.74921
SD	0.1529	5.32683	0.02682	0.2971	11.65393	0.06910
N	10	10	10	10	10	10
%Diff	6.8851	4.62630	0.84766	2.9292	0.88485	-2.29273

DRAFT, NOT FOR SUBMISSION

Appendix 18
Appendix 2

Individual Organ Weights: Terminal Day 91±2

32025165
Sex: Female Day(s) Relative to Start Date

4.47x10^11 vg Group 3	Organ Weights					
	Liver (g)	Liver (%br)	Liver (%bw)	Ovary Paired (g)	Ovary Paired (%br)	Ovary Paired (%bw)
	-	-	-	-	-	-
3501	16.260	716.2996	4.4520	0.063	2.7753	0.0172
3502	11.193	599.8392	3.9231	0.094	5.0375	0.0329
3503	11.968	574.5559	3.9713	0.107	5.1368	0.0355
3504	9.852	479.6495	3.6398	0.083	4.0409	0.0307
3505	8.985	445.0223	3.2982	0.090	4.4577	0.0330
3506	10.024	527.8568	3.7036	0.060	3.1596	0.0222
3507	12.512	628.4279	4.3291	0.068	3.4154	0.0235
3508	10.646	540.4061	3.6266	0.116	5.8883	0.0395
3509	8.655	450.3122	3.5758	0.063	3.2778	0.0260
3510	10.643	526.3600	4.1951	0.097	4.7972	0.0382
Mean	11.0738	548.87295	3.87148	0.0841	4.19865	0.02989
SD	2.1847	84.28069	0.36844	0.0199	1.02503	0.00737
N	10	10	10	10	10	10
%Diff	13.2128	10.70928	7.34580	2.0631	0.40359	-3.05176

DRAFT, NOT FOR SUBMISSION

Appendix 18
Appendix 2

Individual Organ Weights: Terminal Day 91±2

32025165
Sex: Female Day(s) Relative to Start Date

4.47x10^11 vg Group 3	Organ Weights					
	Spleen (g)	Spleen (%br)	Spleen (%bw)	Thymus (g)	Thymus (%br)	Thymus (%bw)
	-	-	-	-	-	-
3501	0.591	26.0352	0.1618	0.278	12.2467	0.0761
3502	0.597	31.9936	0.2092	0.316	16.9346	0.1108
3503	0.610	29.2847	0.2024	0.249	11.9539	0.0826
3504	0.516	25.1217	0.1906	0.272	13.2425	0.1005
3505	0.438	21.6939	0.1608	0.330	16.3447	0.1211
3506	0.502	26.4350	0.1855	0.225	11.8483	0.0831
3507	0.515	25.8664	0.1782	0.284	14.2642	0.0983
3508	0.545	27.6650	0.1857	0.268	13.6041	0.0913
3509	0.440	22.8928	0.1818	0.278	14.4641	0.1149
3510	0.544	26.9041	0.2144	0.226	11.1771	0.0891
Mean	0.5298	26.38923	0.18704	0.2726	13.60802	0.09678
SD	0.0604	2.94322	0.01804	0.0340	1.92782	0.01503
N	10	10	10	10	10	10
%Diff	9.5533	7.53462	4.44757	10.4538	8.77575	7.87346

DRAFT, NOT FOR SUBMISSION

Appendix 18

Appendix 2

Individual Organ Weights: Terminal Day 91±2

32025165

Sex: Female Day(s) Relative to Start Date

4.47x10^11 vg Group 3	Organ Weights		
	Uterus/ Cervix (g)	Uterus/ Cervix (%br)	Uterus/ Cervix (%bw)
	-	-	-
3501	1.015	44.7137	0.2779
3502	0.880	47.1597	0.3084
3503	1.028	49.3519	0.3411
3504	0.753	36.6602	0.2782
3505	0.778	38.5339	0.2856
3506	0.801	42.1801	0.2960
3507	0.837	42.0392	0.2896
3508	0.680	34.5178	0.2316
3509	0.404	21.0198	0.1669
3510	0.466	23.0465	0.1837
Mean	0.7642	37.92227	0.26590
SD	0.2051	9.53187	0.05512
N	10	10	10
%Diff	-14.6717	-16.19017	-20.34777

DRAFT, NOT FOR SUBMISSION

Appendix 18

Appendix 2

Individual Organ Weights: Terminal Day 91±2

32025165

Sex: Female Day(s) Relative to Start Date

1.49x10^12 vg Group 4	Organ Weights					
	Terminal Body Wt (g)	Brain (g)	Brain (%bw)	Adrenal (g)	Adrenal (%br)	Adrenal (%bw)
	-	-	-	-	-	-
4506	305.863	1.920	0.6277	0.0760	3.9583	0.0248
4507	281.501	2.027	0.7201	0.0640	3.1574	0.0227
4508	278.957	2.000	0.7170	0.0660	3.3000	0.0237
4509	306.192	2.109	0.6888	0.0680	3.2243	0.0222
4510	258.243	1.895	0.7338	0.0640	3.3773	0.0248
4511	264.506	2.019	0.7633	0.0650	3.2194	0.0246
4512	320.302	1.950	0.6088	0.0880	4.5128	0.0275
4513	276.409	1.978	0.7156	0.0650	3.2861	0.0235
4514	296.358	1.891	0.6381	0.0650	3.4373	0.0219
4515	292.613	2.071	0.7078	0.0770	3.7180	0.0263
Mean	288.0944	1.9860	0.69209	0.06980	3.51910	0.02420
SD	19.6647	0.0733	0.05057	0.00800	0.42903	0.00177
N	10	10	10	10	10	10
%Diff	6.6135	0.5875	-5.91533	25.08961	24.48220	15.84585

DRAFT, NOT FOR SUBMISSION

Appendix 18

Appendix 2

Individual Organ Weights: Terminal Day 91±2

32025165

Sex: Female Day(s) Relative to Start Date

1.49x10^12 vg Group 4	Organ Weights					
	Pituitary (g)	Pituitary (%br)	Pituitary (%bw)	Thyroid/ Parathyroid (g)	Thyroid/ Parathyroid (%br)	Thyroid/ Parathyroid (%bw)
	-	-	-	-	-	-
4506	0.0211	1.0990	0.0069	0.0346	1.8021	0.0113
4507	0.0230	1.1347	0.0082	0.0232	1.1445	0.0082
4508	0.0265	1.3250	0.0095	0.0226	1.1300	0.0081
4509	0.0192	0.9104	0.0063	0.0237	1.1238	0.0077
4510	0.0258	1.3615	0.0100	0.0269	1.4195	0.0104
4511	0.0268	1.3274	0.0101	0.0249	1.2333	0.0094
4512	0.0249	1.2769	0.0078	0.0181	0.9282	0.0057
4513	0.0226	1.1426	0.0082	0.0169	0.8544	0.0061
4514	0.0166	0.8778	0.0056	0.0190	1.0048	0.0064
4515	0.0287	1.3858	0.0098	0.0175	0.8450	0.0060
Mean	0.02352	1.18410	0.00823	0.02274	1.14856	0.00794
SD	0.00376	0.18296	0.00162	0.00538	0.28994	0.00196
N	10	10	10	10	10	10
%Diff	29.65821	28.86773	20.72752	1.69946	1.43569	-5.26203

DRAFT, NOT FOR SUBMISSION

Appendix 18

Appendix 2

Individual Organ Weights: Terminal Day 91±2

32025165

Sex: Female Day(s) Relative to Start Date

1.49x10^12 vg Group 4	Organ Weights					
	Heart (g)	Heart (%br)	Heart (%bw)	Kidney (g)	Kidney (%br)	Kidney (%bw)
	-	-	-	-	-	-
4506	1.188	61.8750	0.3884	2.078	108.2292	0.6794
4507	0.980	48.3473	0.3481	2.102	103.7000	0.7467
4508	0.935	46.7500	0.3352	2.158	107.9000	0.7736
4509	1.116	52.9161	0.3645	2.552	121.0052	0.8335
4510	1.005	53.0343	0.3892	1.918	101.2137	0.7427
4511	0.935	46.3101	0.3535	2.260	111.9366	0.8544
4512	1.216	62.3590	0.3796	2.498	128.1026	0.7799
4513	0.955	48.2811	0.3455	2.040	103.1345	0.7380
4514	0.997	52.7234	0.3364	1.818	96.1396	0.6134
4515	0.993	47.9479	0.3394	2.202	106.3254	0.7525
Mean	1.0320	52.05441	0.35798	2.1626	108.76869	0.75142
SD	0.1034	5.87610	0.02112	0.2308	9.51367	0.06924
N	10	10	10	10	10	10
%Diff	1.2162	0.67569	-5.77340	4.5340	3.72654	-2.00458

DRAFT, NOT FOR SUBMISSION

Appendix 18

Appendix 2

Individual Organ Weights: Terminal Day 91±2

32025165

Sex: Female Day(s) Relative to Start Date

1.49x10^12 vg Group 4	Organ Weights					
	Liver (g)	Liver (%br)	Liver (%bw)	Ovary Paired (g)	Ovary Paired (%br)	Ovary Paired (%bw)
	-	-	-	-	-	-
4506	12.177	634.2188	3.9812	0.073	3.8021	0.0239
4507	9.848	485.8411	3.4984	0.071	3.5027	0.0252
4508	10.820	541.0000	3.8787	0.071	3.5500	0.0255
4509	10.316	489.1418	3.3691	0.116	5.5002	0.0379
4510	9.781	516.1478	3.7875	0.055	2.9024	0.0213
4511	11.100	549.7771	4.1965	0.063	3.1204	0.0238
4512	12.015	616.1538	3.7511	0.084	4.3077	0.0262
4513	9.477	479.1203	3.4286	0.078	3.9434	0.0282
4514	9.843	520.5182	3.3213	0.078	4.1248	0.0263
4515	11.140	537.9044	3.8071	0.097	4.6837	0.0331
Mean	10.6517	536.98234	3.70196	0.0786	3.94374	0.02715
SD	0.9547	52.55795	0.28798	0.0174	0.76504	0.00491
N	10	10	10	10	10	10
%Diff	8.8975	8.31090	2.64556	-4.6117	-5.69231	-11.94725

DRAFT, NOT FOR SUBMISSION

Appendix 18

Appendix 2

Individual Organ Weights: Terminal Day 91±2

32025165

Sex: Female Day(s) Relative to Start Date

1.49x10^12 vg Group 4	Organ Weights					
	Spleen (g)	Spleen (%br)	Spleen (%bw)	Thymus (g)	Thymus (%br)	Thymus (%bw)
	-	-	-	-	-	-
4506	0.520	27.0833	0.1700	0.355	18.4896	0.1161
4507	0.479	23.6310	0.1702	0.213	10.5081	0.0757
4508	0.486	24.3000	0.1742	0.302	15.1000	0.1083
4509	0.530	25.1304	0.1731	0.329	15.5998	0.1074
4510	0.482	25.4354	0.1866	0.283	14.9340	0.1096
4511	0.397	19.6632	0.1501	0.456	22.5854	0.1724
4512	0.633	32.4615	0.1976	0.372	19.0769	0.1161
4513	0.483	24.4186	0.1747	0.350	17.6946	0.1266
4514	0.470	24.8546	0.1586	0.279	14.7541	0.0941
4515	0.879	42.4433	0.3004	0.226	10.9126	0.0772
Mean	0.5359	26.94212	0.18556	0.3165	15.96553	0.11036
SD	0.1344	6.30674	0.04243	0.0722	3.67566	0.02740
N	10	10	10	10	10	10
%Diff	10.8147	9.78762	3.61809	28.2415	27.62053	23.01207

DRAFT, NOT FOR SUBMISSION

Appendix 18

Appendix 2

Individual Organ Weights: Terminal Day 91±2

32025165

Sex: Female Day(s) Relative to Start Date

1.49x10^12 vg Group 4	Organ Weights		
	Uterus/ Cervix (g)	Uterus/ Cervix (%br)	Uterus/ Cervix (%bw)
	-	-	-
4506	0.671	34.9479	0.2194
4507	0.642	31.6724	0.2281
4508	0.936	46.8000	0.3355
4509	0.739	35.0403	0.2414
4510	0.585	30.8707	0.2265
4511	0.838	41.5057	0.3168
4512	0.907	46.5128	0.2832
4513	1.084	54.8028	0.3922
4514	0.776	41.0365	0.2618
4515	0.553	26.7021	0.1890
Mean	0.7731	38.98913	0.26939
SD	0.1693	8.69580	0.06258
N	10	10	10
%Diff	-13.6780	-13.83236	-19.30498

DRAFT, NOT FOR SUBMISSION

Appendix 18

Appendix 3

Individual Macroscopic and Microscopic Pathology: Terminal Day 29±1

32025165

Animal: 1001	Group: 1	Sex: Male
Species: Rat	Strain: Sprague Dawley	
	Dose: 0 vg	
	Removal Reason: Terminal Euthanasia	
	Study Day (Week) of Death: 28 (4)	
		Gross Status: Complete
		Histo Status: Complete

Gross Pathology Animal Details:

No animal details found

Gross Pathology Observations [Correlation]:

No observations found

Any remaining protocol required tissues, which have been examined, have no visible lesions

Gross Pathology - The following Tissues were Not Examined:

None

Histopathology Animal Details:

No animal details found

Histopathology Observations [Correlation]:

GANGLION, DORSAL ROOT, LUMBAR : Infiltration, mononuclear cell; minimal

Histopathology - The following Tissues were Within Normal Limits:

BRAIN; GANGLION, DORSAL ROOT, CERVICAL; GANGLION, DORSAL ROOT, THORACIC; GLAND, ADRENAL; GLAND, MAMMARY; HEART; KIDNEY; LARGE INTESTINE, COLON; LIVER; LUNG; LYMPH NODE, ILIAC; NERVE, SCIATIC; PANCREAS; SPINAL CORD; SPLEEN; TESTIS, RIGHT

Histopathology - The following Tissues were Not Examined:

None

DRAFT, NOT FOR SUBMISSION

Appendix 18

Appendix 3

Individual Macroscopic and Microscopic Pathology: Terminal Day 29±1

32025165

Animal:	1002	Group:	1	Sex:	Male
Species:	Rat	Strain:	Sprague Dawley		
		Dose:	0 vg		
		Removal Reason:	Terminal Euthanasia		
		Study Day (Week) of Death:	28 (4)		
				Gross Status:	Complete
				Histo Status:	Complete

Gross Pathology Animal Details:

No animal details found

Gross Pathology Observations [Correlation]:

No observations found

Any remaining protocol required tissues, which have been examined, have no visible lesions

Gross Pathology - The following Tissues were Not Examined:

None

Histopathology Animal Details:

No animal details found

Histopathology Observations [Correlation]:

No observations found

Histopathology - The following Tissues were Within Normal Limits:

BRAIN; GANGLION, DORSAL ROOT, CERVICAL; GANGLION, DORSAL ROOT, LUMBAR; GANGLION, DORSAL ROOT, THORACIC; GLAND, ADRENAL; GLAND, MAMMARY; HEART; KIDNEY; LARGE INTESTINE, COLON; LIVER; LUNG; LYMPH NODE, ILIAC; NERVE, SCIATIC; PANCREAS; SPINAL CORD; SPLEEN; TESTIS, RIGHT

Histopathology - The following Tissues were Not Examined:

None

DRAFT, NOT FOR SUBMISSION

Appendix 18

Appendix 3

Individual Macroscopic and Microscopic Pathology: Terminal Day 29±1

32025165

Animal:	1003	Group:	1	Sex:	Male
Species:	Rat	Strain:	Sprague Dawley		
		Dose:	0 vg		
		Removal Reason:	Terminal Euthanasia		
		Study Day (Week) of Death:	28 (4)		
				Gross Status:	Complete
				Histo Status:	Complete

Gross Pathology Animal Details:

No animal details found

Gross Pathology Observations [Correlation]:

No observations found

Any remaining protocol required tissues, which have been examined, have no visible lesions

Gross Pathology - The following Tissues were Not Examined:

None

Histopathology Animal Details:

No animal details found

Histopathology Observations [Correlation]:

KIDNEY : Mineralization; focal, mild
KIDNEY : Necrosis; focal, mild

Histopathology - The following Tissues were Within Normal Limits:

BRAIN; GANGLION, DORSAL ROOT, CERVICAL; GANGLION, DORSAL ROOT, LUMBAR; GANGLION, DORSAL ROOT, THORACIC; GLAND, ADRENAL; GLAND, MAMMARY; HEART; LARGE INTESTINE, COLON; LIVER; LUNG; LYMPH NODE, ILIAC; NERVE, SCIATIC; PANCREAS; SPINAL CORD; SPLEEN; TESTIS, RIGHT

Histopathology - The following Tissues were Not Examined:

None

DRAFT, NOT FOR SUBMISSION

Appendix 18

Appendix 3

Individual Macroscopic and Microscopic Pathology: Terminal Day 29±1

32025165

Animal: 1004	Group: 1	Sex: Male
Species: Rat	Strain: Sprague Dawley	
	Dose: 0 vg	
	Removal Reason: Terminal Euthanasia	
	Study Day (Week) of Death: 28 (4)	
		Gross Status: Complete
		Histo Status: Complete

Gross Pathology Animal Details:

No animal details found

Gross Pathology Observations [Correlation]:

No observations found

Any remaining protocol required tissues, which have been examined, have no visible lesions

Gross Pathology - The following Tissues were Not Examined:

None

Histopathology Animal Details:

No animal details found

Histopathology Observations [Correlation]:

No observations found

Histopathology - The following Tissues were Within Normal Limits:

BRAIN; GANGLION, DORSAL ROOT, CERVICAL; GANGLION, DORSAL ROOT, LUMBAR; GANGLION, DORSAL ROOT, THORACIC; GLAND, ADRENAL; GLAND, MAMMARY; HEART; KIDNEY; LARGE INTESTINE, COLON; LIVER; LUNG; LYMPH NODE, ILIAC; NERVE, SCIATIC; PANCREAS; SPINAL CORD; SPLEEN; TESTIS, RIGHT

Histopathology - The following Tissues were Not Examined:

None

DRAFT, NOT FOR SUBMISSION

Appendix 18

Appendix 3

Individual Macroscopic and Microscopic Pathology: Terminal Day 29±1

32025165

Animal: 1005	Group: 1	Sex: Male
Species: Rat	Strain: Sprague Dawley	
	Dose: 0 vg	
	Removal Reason: Terminal Euthanasia	
	Study Day (Week) of Death: 28 (4)	
		Gross Status: Complete
		Histo Status: Complete

Gross Pathology Animal Details:

No animal details found

Gross Pathology Observations [Correlation]:

No observations found

Any remaining protocol required tissues, which have been examined, have no visible lesions

Gross Pathology - The following Tissues were Not Examined:

None

Histopathology Animal Details:

No animal details found

Histopathology Observations [Correlation]:

No observations found

Histopathology - The following Tissues were Within Normal Limits:

BRAIN; GANGLION, DORSAL ROOT, CERVICAL; GANGLION, DORSAL ROOT, LUMBAR; GANGLION, DORSAL ROOT, THORACIC; GLAND, ADRENAL; GLAND, MAMMARY; HEART; KIDNEY; LARGE INTESTINE, COLON; LIVER; LUNG; LYMPH NODE, ILIAC; NERVE, SCIATIC; PANCREAS; SPINAL CORD; SPLEEN; TESTIS, RIGHT

Histopathology - The following Tissues were Not Examined:

None

DRAFT, NOT FOR SUBMISSION

Appendix 18

Appendix 3

Individual Macroscopic and Microscopic Pathology: Terminal Day 29±1

32025165

Animal: 1501	Group: 1	Sex: Female
Species: Rat	Strain: Sprague Dawley	
	Dose: 0 vg	
	Removal Reason: Terminal Euthanasia	
	Study Day (Week) of Death: 29 (5)	
		Gross Status: Complete
		Histo Status: Complete

Gross Pathology Animal Details:

No animal details found

Gross Pathology Observations [Correlation]:

No observations found

Any remaining protocol required tissues, which have been examined, have no visible lesions

Gross Pathology - The following Tissues were Not Examined:

None

Histopathology Animal Details:

No animal details found

Histopathology Observations [Correlation]:

No observations found

Histopathology - The following Tissues were Within Normal Limits:

BRAIN; GANGLION, DORSAL ROOT, CERVICAL; GANGLION, DORSAL ROOT, LUMBAR; GANGLION, DORSAL ROOT, THORACIC; GLAND, ADRENAL; GLAND, MAMMARY; HEART; KIDNEY; LARGE INTESTINE, COLON; LIVER; LUNG; LYMPH NODE, ILIAC; NERVE, SCIATIC; OVARY; PANCREAS; SPINAL CORD; SPLEEN

Histopathology - The following Tissues were Not Examined:

None

DRAFT, NOT FOR SUBMISSION

Appendix 18

Appendix 3

Individual Macroscopic and Microscopic Pathology: Terminal Day 29±1

32025165

Animal: 1502	Group: 1	Sex: Female
Species: Rat	Strain: Sprague Dawley	
	Dose: 0 vg	
	Removal Reason: Terminal Euthanasia	
	Study Day (Week) of Death: 29 (5)	
		Gross Status: Complete
		Histo Status: Complete

Gross Pathology Animal Details:

No animal details found

Gross Pathology Observations [Correlation]:

No observations found

Any remaining protocol required tissues, which have been examined, have no visible lesions

Gross Pathology - The following Tissues were Not Examined:

None

Histopathology Animal Details:

No animal details found

Histopathology Observations [Correlation]:

KIDNEY : Dilatation; minimal, pelvis

Histopathology - The following Tissues were Within Normal Limits:

BRAIN; GANGLION, DORSAL ROOT, CERVICAL; GANGLION, DORSAL ROOT, LUMBAR; GANGLION, DORSAL ROOT, THORACIC; GLAND, ADRENAL; GLAND, MAMMARY; HEART; LARGE INTESTINE, COLON; LIVER; LUNG; LYMPH NODE, ILIAC; NERVE, SCIATIC; OVARY; PANCREAS; SPINAL CORD; SPLEEN

Histopathology - The following Tissues were Not Examined:

None

DRAFT, NOT FOR SUBMISSION

Appendix 18

Appendix 3

Individual Macroscopic and Microscopic Pathology: Terminal Day 29±1

32025165

Animal:	1503	Group:	1	Sex:	Female
Species:	Rat	Strain:	Sprague Dawley		
		Dose:	0 vg		
		Removal Reason:	Terminal Euthanasia		
		Study Day (Week) of Death:	29 (5)		
				Gross Status:	Complete
				Histo Status:	Complete

Gross Pathology Animal Details:

No animal details found

Gross Pathology Observations [Correlation]:

No observations found

Any remaining protocol required tissues, which have been examined, have no visible lesions

Gross Pathology - The following Tissues were Not Examined:

None

Histopathology Animal Details:

No animal details found

Histopathology Observations [Correlation]:

No observations found

Histopathology - The following Tissues were Within Normal Limits:

BRAIN; GANGLION, DORSAL ROOT, CERVICAL; GANGLION, DORSAL ROOT, LUMBAR; GANGLION, DORSAL ROOT, THORACIC; GLAND, ADRENAL; GLAND, MAMMARY; HEART; KIDNEY; LARGE INTESTINE, COLON; LIVER; LUNG; LYMPH NODE, ILIAC; NERVE, SCIATIC; OVARY; PANCREAS; SPINAL CORD; SPLEEN

Histopathology - The following Tissues were Not Examined:

None

DRAFT, NOT FOR SUBMISSION

Appendix 18

Appendix 3

Individual Macroscopic and Microscopic Pathology: Terminal Day 29±1

32025165

Animal: 1504	Group: 1	Sex: Female
Species: Rat	Strain: Sprague Dawley	
	Dose: 0 vg	
	Removal Reason: Terminal Euthanasia	
	Study Day (Week) of Death: 29 (5)	
		Gross Status: Complete
		Histo Status: Complete

Gross Pathology Animal Details:

No animal details found

Gross Pathology Observations [Correlation]:

No observations found

Any remaining protocol required tissues, which have been examined, have no visible lesions

Gross Pathology - The following Tissues were Not Examined:

None

Histopathology Animal Details:

No animal details found

Histopathology Observations [Correlation]:

No observations found

Histopathology - The following Tissues were Within Normal Limits:

BRAIN; GANGLION, DORSAL ROOT, CERVICAL; GANGLION, DORSAL ROOT, LUMBAR; GANGLION, DORSAL ROOT, THORACIC; GLAND, ADRENAL; GLAND, MAMMARY; HEART; KIDNEY; LARGE INTESTINE, COLON; LIVER; LUNG; LYMPH NODE, ILIAC; NERVE, SCIATIC; OVARY; PANCREAS; SPINAL CORD; SPLEEN

Histopathology - The following Tissues were Not Examined:

None

DRAFT, NOT FOR SUBMISSION

Appendix 18

Appendix 3

Individual Macroscopic and Microscopic Pathology: Terminal Day 29±1

32025165

Animal:	1505	Group:	1	Sex:	Female
Species:	Rat	Strain:	Sprague Dawley		
		Dose:	0 vg		
		Removal Reason:	Terminal Euthanasia		
		Study Day (Week) of Death:	29 (5)		
				Gross Status:	Complete
				Histo Status:	Complete

Gross Pathology Animal Details:

No animal details found

Gross Pathology Observations [Correlation]:

No observations found

Any remaining protocol required tissues, which have been examined, have no visible lesions

Gross Pathology - The following Tissues were Not Examined:

None

Histopathology Animal Details:

No animal details found

Histopathology Observations [Correlation]:

GANGLION, DORSAL ROOT, LUMBAR : Infiltration, mononuclear cell; minimal
LUNG : Alveolar macrophages, increased; minimal

Histopathology - The following Tissues were Within Normal Limits:

BRAIN; GANGLION, DORSAL ROOT, CERVICAL; GANGLION, DORSAL ROOT, THORACIC; GLAND, ADRENAL; GLAND, MAMMARY; HEART; KIDNEY; LARGE INTESTINE, COLON; LIVER; LYMPH NODE, ILIAC; NERVE, SCIATIC; OVARY; PANCREAS; SPINAL CORD; SPLEEN

Histopathology - The following Tissues were Not Examined:

None

DRAFT, NOT FOR SUBMISSION

Appendix 18

Appendix 3

Individual Macroscopic and Microscopic Pathology: Terminal Day 29±1

32025165

Animal: 4001	Group: 4	Sex: Male
Species: Rat	Strain: Sprague Dawley	
	Dose: 1.49x10^12 vg	
	Removal Reason: Terminal Euthanasia	
	Study Day (Week) of Death: 28 (4)	
		Gross Status: Complete
		Histo Status: Complete

Gross Pathology Animal Details:

No animal details found

Gross Pathology Observations [Correlation]:

No observations found

Any remaining protocol required tissues, which have been examined, have no visible lesions

Gross Pathology - The following Tissues were Not Examined:

None

Histopathology Animal Details:

No animal details found

Histopathology Observations [Correlation]:

No observations found

Histopathology - The following Tissues were Within Normal Limits:

BRAIN; GANGLION, DORSAL ROOT, CERVICAL; GANGLION, DORSAL ROOT, LUMBAR; GANGLION, DORSAL ROOT, THORACIC; GLAND, ADRENAL; GLAND, MAMMARY; HEART; KIDNEY; LARGE INTESTINE, COLON; LIVER; LUNG; LYMPH NODE, ILIAC; NERVE, SCIATIC; PANCREAS; SPINAL CORD; SPLEEN; TESTIS, RIGHT

Histopathology - The following Tissues were Not Examined:

None

Appendix 18

Appendix 3

Individual Macroscopic and Microscopic Pathology: Terminal Day 29±1

32025165

Animal: 4002	Group: 4	Sex: Male
Species: Rat	Strain: Sprague Dawley	
	Dose: 1.49x10^12 vg	
	Removal Reason: Terminal Euthanasia	
	Study Day (Week) of Death: 28 (4)	
		Gross Status: Complete
		Histo Status: Complete

Gross Pathology Animal Details:

No animal details found

Gross Pathology Observations [Correlation]:

No observations found

Any remaining protocol required tissues, which have been examined, have no visible lesions

Gross Pathology - The following Tissues were Not Examined:

None

Histopathology Animal Details:

No animal details found

Histopathology Observations [Correlation]:

GANGLION, DORSAL ROOT, LUMBAR : Infiltration, mononuclear cell; mild
GANGLION, DORSAL ROOT, LUMBAR : Degeneration; minimal, nerve fiber
LIVER : Vacuolation; hepatocellular, minimal
NERVE, SCIATIC : Degeneration; minimal, nerve fiber
SPINAL CORD : (Comment) Nerve root, lumbar
SPINAL CORD : Degeneration; minimal, nerve fiber

Histopathology - The following Tissues were Within Normal Limits:

BRAIN; GANGLION, DORSAL ROOT, CERVICAL; GANGLION, DORSAL ROOT, THORACIC; GLAND, ADRENAL; GLAND, MAMMARY; HEART; KIDNEY; LARGE INTESTINE, COLON; LUNG; LYMPH NODE, ILIAC; PANCREAS; SPLEEN; TESTIS, RIGHT

Histopathology - The following Tissues were Not Examined:

None

DRAFT, NOT FOR SUBMISSION

Appendix 18

Appendix 3

Individual Macroscopic and Microscopic Pathology: Terminal Day 29±1

32025165

Animal: 4003	Group: 4	Sex: Male
Species: Rat	Strain: Sprague Dawley	
	Dose: 1.49x10^12 vg	
	Removal Reason: Terminal Euthanasia	
	Study Day (Week) of Death: 28 (4)	
		Gross Status: Complete
		Histo Status: Complete

Gross Pathology Animal Details:

No animal details found

Gross Pathology Observations [Correlation]:

No observations found

Any remaining protocol required tissues, which have been examined, have no visible lesions

Gross Pathology - The following Tissues were Not Examined:

None

Histopathology Animal Details:

No animal details found

Histopathology Observations [Correlation]:

GANGLION, DORSAL ROOT, LUMBAR : Infiltration, mononuclear cell; minimal
GANGLION, DORSAL ROOT, LUMBAR : Degeneration; mild, nerve fiber
GANGLION, DORSAL ROOT, LUMBAR : Degeneration/necrosis; neuronal, minimal
GLAND, MAMMARY : Infiltration, mixed cell; minimal
GLAND, MAMMARY : Necrosis; minimal
NERVE, SCIATIC : Degeneration; minimal, nerve fiber
SPINAL CORD : (Comment) Nerve root lumbar
SPINAL CORD : Degeneration; mild, nerve fiber

Histopathology - The following Tissues were Within Normal Limits:

BRAIN; GANGLION, DORSAL ROOT, CERVICAL; GANGLION, DORSAL ROOT, THORACIC; GLAND, ADRENAL; HEART; KIDNEY; LARGE INTESTINE, COLON; LIVER; LUNG; LYMPH NODE, ILIAC; PANCREAS; SPLEEN; TESTIS, RIGHT

Histopathology - The following Tissues were Not Examined:

None

DRAFT, NOT FOR SUBMISSION

Appendix 18

Appendix 3

Individual Macroscopic and Microscopic Pathology: Terminal Day 29±1

32025165

Animal: 4004	Group: 4	Sex: Male
Species: Rat	Strain: Sprague Dawley	
	Dose: 1.49x10^12 vg	
	Removal Reason: Terminal Euthanasia	
	Study Day (Week) of Death: 28 (4)	
		Gross Status: Complete
		Histo Status: Complete

Gross Pathology Animal Details:

No animal details found

Gross Pathology Observations [Correlation]:

No observations found

Any remaining protocol required tissues, which have been examined, have no visible lesions

Gross Pathology - The following Tissues were Not Examined:

None

Histopathology Animal Details:

No animal details found

Histopathology Observations [Correlation]:

No observations found

Histopathology - The following Tissues were Within Normal Limits:

BRAIN; GANGLION, DORSAL ROOT, CERVICAL; GANGLION, DORSAL ROOT, LUMBAR; GANGLION, DORSAL ROOT, THORACIC; GLAND, ADRENAL; GLAND, MAMMARY; HEART; KIDNEY; LARGE INTESTINE, COLON; LIVER; LUNG; LYMPH NODE, ILIAC; NERVE, SCIATIC; PANCREAS; SPINAL CORD; SPLEEN; TESTIS, RIGHT

Histopathology - The following Tissues were Not Examined:

None

DRAFT, NOT FOR SUBMISSION

Appendix 18

Appendix 3

Individual Macroscopic and Microscopic Pathology: Terminal Day 29±1

32025165

Animal: 4005	Group: 4	Sex: Male
Species: Rat	Strain: Sprague Dawley	
	Dose: 1.49x10^12 vg	
	Removal Reason: Terminal Euthanasia	
	Study Day (Week) of Death: 28 (4)	
		Gross Status: Complete
		Histo Status: Complete

Gross Pathology Animal Details:

No animal details found

Gross Pathology Observations [Correlation]:

No observations found

Any remaining protocol required tissues, which have been examined, have no visible lesions

Gross Pathology - The following Tissues were Not Examined:

None

Histopathology Animal Details:

No animal details found

Histopathology Observations [Correlation]:

No observations found

Histopathology - The following Tissues were Within Normal Limits:

BRAIN; GANGLION, DORSAL ROOT, CERVICAL; GANGLION, DORSAL ROOT, LUMBAR; GANGLION, DORSAL ROOT, THORACIC; GLAND, ADRENAL; GLAND, MAMMARY; HEART; KIDNEY; LARGE INTESTINE, COLON; LIVER; LUNG; LYMPH NODE, ILIAC; NERVE, SCIATIC; PANCREAS; SPINAL CORD; SPLEEN; TESTIS, RIGHT

Histopathology - The following Tissues were Not Examined:

None

DRAFT, NOT FOR SUBMISSION

Appendix 18

Appendix 3

Individual Macroscopic and Microscopic Pathology: Terminal Day 29±1

32025165

Animal:	4501	Group:	4	Sex:	Female
Species:	Rat	Strain:	Sprague Dawley		
		Dose:	1.49x10^12 vg		
		Removal Reason:	Terminal Euthanasia		
		Study Day (Week) of Death:	29 (5)		
				Gross Status:	Complete
				Histo Status:	Complete

Gross Pathology Animal Details:

No animal details found

Gross Pathology Observations [Correlation]:

No observations found

Any remaining protocol required tissues, which have been examined, have no visible lesions

Gross Pathology - The following Tissues were Not Examined:

None

Histopathology Animal Details:

No animal details found

Histopathology Observations [Correlation]:

GANGLION, DORSAL ROOT, LUMBAR : Infiltration, mononuclear cell; minimal
GANGLION, DORSAL ROOT, LUMBAR : Degeneration; mild, nerve fiber
GANGLION, DORSAL ROOT, LUMBAR : Degeneration/necrosis; neuronal, minimal
NERVE, SCIATIC : Degeneration; minimal, nerve fiber
SPINAL CORD : (Comment) Dorsal funiculus
SPINAL CORD : Degeneration; minimal, nerve fiber

Histopathology - The following Tissues were Within Normal Limits:

BRAIN; GANGLION, DORSAL ROOT, CERVICAL; GANGLION, DORSAL ROOT, THORACIC; GLAND, ADRENAL; GLAND, MAMMARY; HEART; KIDNEY; LARGE INTESTINE, COLON; LIVER; LUNG; LYMPH NODE, ILIAC; OVARY; PANCREAS; SPLEEN

Histopathology - The following Tissues were Not Examined:

None

DRAFT, NOT FOR SUBMISSION

Appendix 18

Appendix 3

Individual Macroscopic and Microscopic Pathology: Terminal Day 29±1

32025165

Animal: 4502	Group: 4	Sex: Female
Species: Rat	Strain: Sprague Dawley	
	Dose: 1.49x10^12 vg	
	Removal Reason: Terminal Euthanasia	
	Study Day (Week) of Death: 29 (5)	
		Gross Status: Complete
		Histo Status: Complete

Gross Pathology Animal Details:

No animal details found

Gross Pathology Observations [Correlation]:

No observations found

Any remaining protocol required tissues, which have been examined, have no visible lesions

Gross Pathology - The following Tissues were Not Examined:

None

Histopathology Animal Details:

No animal details found

Histopathology Observations [Correlation]:

GANGLION, DORSAL ROOT, LUMBAR : Infiltration, mononuclear cell; minimal
KIDNEY : Mineralization; mild
KIDNEY : Dilatation; minimal, pelvis
NERVE, SCIATIC : Degeneration; minimal, nerve fiber
SPINAL CORD : (Comment) Nerve root lumbar and dorsal funiculus
SPINAL CORD : Degeneration; minimal, nerve fiber

Histopathology - The following Tissues were Within Normal Limits:

BRAIN; GANGLION, DORSAL ROOT, CERVICAL; GANGLION, DORSAL ROOT, THORACIC; GLAND, ADRENAL; GLAND, MAMMARY; HEART; LARGE INTESTINE, COLON; LIVER; LUNG; LYMPH NODE, ILIAC; OVARY; PANCREAS; SPLEEN

Histopathology - The following Tissues were Not Examined:

None

DRAFT, NOT FOR SUBMISSION

Appendix 18

Appendix 3

Individual Macroscopic and Microscopic Pathology: Terminal Day 29±1

32025165

Animal: 4503	Group: 4	Sex: Female
Species: Rat	Strain: Sprague Dawley	
	Dose: 1.49x10^12 vg	
	Removal Reason: Terminal Euthanasia	
	Study Day (Week) of Death: 29 (5)	
		Gross Status: Complete
		Histo Status: Complete

Gross Pathology Animal Details:

No animal details found

Gross Pathology Observations [Correlation]:

No observations found

Any remaining protocol required tissues, which have been examined, have no visible lesions

Gross Pathology - The following Tissues were Not Examined:

None

Histopathology Animal Details:

No animal details found

Histopathology Observations [Correlation]:

GANGLION, DORSAL ROOT, LUMBAR : Infiltration, mononuclear cell; mild
GANGLION, DORSAL ROOT, LUMBAR : Degeneration; mild, nerve fiber
GANGLION, DORSAL ROOT, LUMBAR : Degeneration/necrosis; neuronal, minimal
NERVE, SCIATIC : Degeneration; minimal, nerve fiber
NERVE, SCIATIC : Infiltration, mononuclear cell; minimal
SPINAL CORD : (Comment) Nerve root lumbar
SPINAL CORD : Degeneration; minimal, nerve fiber

Histopathology - The following Tissues were Within Normal Limits:

BRAIN; GANGLION, DORSAL ROOT, CERVICAL; GANGLION, DORSAL ROOT, THORACIC; GLAND, ADRENAL; GLAND, MAMMARY; HEART; KIDNEY; LARGE INTESTINE, COLON; LIVER; LUNG; LYMPH NODE, ILIAC; OVARY; PANCREAS; SPLEEN

Histopathology - The following Tissues were Not Examined:

None

DRAFT, NOT FOR SUBMISSION

Appendix 18
Appendix 3

Individual Macroscopic and Microscopic Pathology: Terminal Day 29±1

32025165

Animal:	4504	Group:	4	Sex:	Female
Species:	Rat	Strain:	Sprague Dawley		
		Dose:	1.49x10^12 vg		
		Removal Reason:	Terminal Euthanasia		
		Study Day (Week) of Death:	29 (5)		
				Gross Status:	Complete
				Histo Status:	Complete

Gross Pathology Animal Details:

No animal details found

Gross Pathology Observations [Correlation]:

No observations found

Any remaining protocol required tissues, which have been examined, have no visible lesions

Gross Pathology - The following Tissues were Not Examined:

None

Histopathology Animal Details:

No animal details found

Histopathology Observations [Correlation]:

GANGLION, DORSAL ROOT, LUMBAR : Infiltration, mononuclear cell; minimal
KIDNEY : Infiltration, mixed cell; minimal, adipose tissue

Histopathology - The following Tissues were Within Normal Limits:

BRAIN; GANGLION, DORSAL ROOT, CERVICAL; GANGLION, DORSAL ROOT, THORACIC; GLAND, ADRENAL; GLAND, MAMMARY; HEART; LARGE INTESTINE, COLON; LIVER; LUNG; LYMPH NODE, ILIAC; NERVE, SCIATIC; OVARY; PANCREAS; SPINAL CORD; SPLEEN

Histopathology - The following Tissues were Not Examined:

None

DRAFT, NOT FOR SUBMISSION

Appendix 18

Appendix 3

Individual Macroscopic and Microscopic Pathology: Terminal Day 29±1

32025165

Animal: 4505	Group: 4	Sex: Female
Species: Rat	Strain: Sprague Dawley<br>Dose: 1.49x10^12 vg<br>Removal Reason: Terminal Euthanasia<br>Study Day (Week) of Death: 29 (5)	
		Gross Status: Complete<br>Histo Status: Complete

Gross Pathology Animal Details:

No animal details found

Gross Pathology Observations [Correlation]:

No observations found

Any remaining protocol required tissues, which have been examined, have no visible lesions

Gross Pathology - The following Tissues were Not Examined:

None

Histopathology Animal Details:

No animal details found

Histopathology Observations [Correlation]:

GANGLION, DORSAL ROOT, LUMBAR : Infiltration, mononuclear cell; minimal
NERVE, SCIATIC : Degeneration; mild, nerve fiber

Histopathology - The following Tissues were Within Normal Limits:

BRAIN; GANGLION, DORSAL ROOT, CERVICAL; GANGLION, DORSAL ROOT, THORACIC; GLAND, ADRENAL; GLAND, MAMMARY; HEART; KIDNEY; LARGE INTESTINE, COLON; LIVER; LUNG; LYMPH NODE, ILIAC; OVARY; PANCREAS; SPINAL CORD; SPLEEN

Histopathology - The following Tissues were Not Examined:

None

DRAFT, NOT FOR SUBMISSION

Appendix 18

Appendix 3

Individual Macroscopic and Microscopic Pathology: Terminal Day 91±2

32025165

Animal:	1006	Group:	1	Sex:	Male
Species:	Rat	Strain:	Sprague Dawley		
		Dose:	0 vg		
		Removal Reason:	Terminal Euthanasia		
		Study Day (Week) of Death:	89 (13)		
				Gross Status:	Complete
				Histo Status:	Complete

Gross Pathology Animal Details:

No animal details found

Gross Pathology Observations [Correlation]:

No observations found

Any remaining protocol required tissues, which have been examined, have no visible lesions

Gross Pathology - The following Tissues were Not Examined:

None

Histopathology Animal Details:

No animal details found

Histopathology Observations [Correlation]:

PANCREAS : Atrophy; minimal

Histopathology - The following Tissues were Within Normal Limits:

BRAIN; GANGLION, DORSAL ROOT, CERVICAL; GANGLION, DORSAL ROOT, LUMBAR; GANGLION, DORSAL ROOT, THORACIC; GLAND, ADRENAL; GLAND, MAMMARY; HEART; KIDNEY; LARGE INTESTINE, COLON; LIVER; LUNG; LYMPH NODE, ILIAC; NERVE, SCIATIC; SPINAL CORD; SPLEEN; TESTIS, RIGHT

Histopathology - The following Tissues were Not Examined:

None

DRAFT, NOT FOR SUBMISSION

Appendix 18

Appendix 3

Individual Macroscopic and Microscopic Pathology: Terminal Day 91±2

32025165

Animal: 1007	Group: 1	Sex: Male
Species: Rat	Strain: Sprague Dawley	
	Dose: 0 vg	
	Removal Reason: Terminal Euthanasia	
	Study Day (Week) of Death: 90 (13)	
		Gross Status: Complete
		Histo Status: Complete

Gross Pathology Animal Details:

No animal details found

Gross Pathology Observations [Correlation]:

No observations found

Any remaining protocol required tissues, which have been examined, have no visible lesions

Gross Pathology - The following Tissues were Not Examined:

None

Histopathology Animal Details:

No animal details found

Histopathology Observations [Correlation]:

GANGLION, DORSAL ROOT, LUMBAR : Infiltration, mononuclear cell; minimal

Histopathology - The following Tissues were Within Normal Limits:

BRAIN; GANGLION, DORSAL ROOT, CERVICAL; GANGLION, DORSAL ROOT, THORACIC; GLAND, ADRENAL; GLAND, MAMMARY; HEART; KIDNEY; LARGE INTESTINE, COLON; LIVER; LUNG; LYMPH NODE, ILIAC; NERVE, SCIATIC; PANCREAS; SPINAL CORD; SPLEEN; TESTIS, RIGHT

Histopathology - The following Tissues were Not Examined:

None

DRAFT, NOT FOR SUBMISSION

Appendix 18

Appendix 3

Individual Macroscopic and Microscopic Pathology: Terminal Day 91±2

32025165

Animal: 1008	Group: 1	Sex: Male
Species: Rat	Strain: Sprague Dawley	
	Dose: 0 vg	
	Removal Reason: Terminal Euthanasia	
	Study Day (Week) of Death: 91 (13)	
		Gross Status: Complete
		Histo Status: Complete

Gross Pathology Animal Details:

No animal details found

Gross Pathology Observations [Correlation]:

No observations found

Any remaining protocol required tissues, which have been examined, have no visible lesions

Gross Pathology - The following Tissues were Not Examined:

None

Histopathology Animal Details:

No animal details found

Histopathology Observations [Correlation]:

PANCREAS : Fibrosis; minimal, islet of langerhans

Histopathology - The following Tissues were Within Normal Limits:

BRAIN; GANGLION, DORSAL ROOT, CERVICAL; GANGLION, DORSAL ROOT, LUMBAR; GANGLION, DORSAL ROOT, THORACIC; GLAND, ADRENAL; GLAND, MAMMARY; HEART; KIDNEY; LARGE INTESTINE, COLON; LIVER; LUNG; LYMPH NODE, ILIAC; NERVE, SCIATIC; SPINAL CORD; SPLEEN; TESTIS, RIGHT

Histopathology - The following Tissues were Not Examined:

None

DRAFT, NOT FOR SUBMISSION

Appendix 18

Appendix 3

Individual Macroscopic and Microscopic Pathology: Terminal Day 91±2

32025165

Animal: 1009	Group: 1	Sex: Male
Species: Rat	Strain: Sprague Dawley	
	Dose: 0 vg	
	Removal Reason: Terminal Euthanasia	
	Study Day (Week) of Death: 91 (13)	
		Gross Status: Complete
		Histo Status: Complete

Gross Pathology Animal Details:

No animal details found

Gross Pathology Observations [Correlation]:

No observations found

Any remaining protocol required tissues, which have been examined, have no visible lesions

Gross Pathology - The following Tissues were Not Examined:

None

Histopathology Animal Details:

No animal details found

Histopathology Observations [Correlation]:

GANGLION, DORSAL ROOT, LUMBAR : Infiltration, mononuclear cell; minimal
GLAND, ADRENAL : (Note) One Of A Pair Available For Evaluation.
PANCREAS : Atrophy; minimal
SPINAL CORD : (Comment) Lumbar, nerve root
SPINAL CORD : Degeneration; mild, nerve fiber

Histopathology - The following Tissues were Within Normal Limits:

BRAIN; GANGLION, DORSAL ROOT, CERVICAL; GANGLION, DORSAL ROOT, THORACIC; GLAND, ADRENAL; HEART; KIDNEY; LARGE INTESTINE, COLON; LIVER; LUNG; LYMPH NODE, ILIAC; NERVE, SCIATIC; SPLEEN; TESTIS, RIGHT

Histopathology - The following Tissues were Not Examined:

GLAND, MAMMARY - Not Present In Section.

DRAFT, NOT FOR SUBMISSION

Appendix 18

Appendix 3

Individual Macroscopic and Microscopic Pathology: Terminal Day 91±2

32025165

Animal:	1010	Group:	1	Sex:	Male
Species:	Rat	Strain:	Sprague Dawley		
		Dose:	0 vg		
		Removal Reason:	Terminal Euthanasia		
		Study Day (Week) of Death:	92 (14)		
				Gross Status:	Complete
				Histo Status:	Complete

Gross Pathology Animal Details:

No animal details found

Gross Pathology Observations [Correlation]:

No observations found

Any remaining protocol required tissues, which have been examined, have no visible lesions

Gross Pathology - The following Tissues were Not Examined:

None

Histopathology Animal Details:

No animal details found

Histopathology Observations [Correlation]:

GANGLION, DORSAL ROOT, LUMBAR : Degeneration; minimal, nerve fiber
GLAND, ADRENAL : Vacuolation; cortical, mild

Histopathology - The following Tissues were Within Normal Limits:

BRAIN; GANGLION, DORSAL ROOT, CERVICAL; GANGLION, DORSAL ROOT, THORACIC; GLAND, MAMMARY; HEART; KIDNEY; LARGE INTESTINE, COLON; LIVER; LUNG; LYMPH NODE, ILIAC; NERVE, SCIATIC; PANCREAS; SPINAL CORD; SPLEEN; TESTIS, RIGHT

Histopathology - The following Tissues were Not Examined:

None

DRAFT, NOT FOR SUBMISSION

Appendix 18

Appendix 3

Individual Macroscopic and Microscopic Pathology: Terminal Day 91±2

32025165

Animal: 1506	Group: 1	Sex: Female
Species: Rat	Strain: Sprague Dawley	
	Dose: 0 vg	
	Removal Reason: Terminal Euthanasia	
	Study Day (Week) of Death: 91 (13)	
		Gross Status: Complete
		Histo Status: Complete

Gross Pathology Animal Details:

No animal details found

Gross Pathology Observations [Correlation]:

No observations found

Any remaining protocol required tissues, which have been examined, have no visible lesions

Gross Pathology - The following Tissues were Not Examined:

None

Histopathology Animal Details:

No animal details found

Histopathology Observations [Correlation]:

No observations found

Histopathology - The following Tissues were Within Normal Limits:

BRAIN; GANGLION, DORSAL ROOT, CERVICAL; GANGLION, DORSAL ROOT, LUMBAR; GANGLION, DORSAL ROOT, THORACIC; GLAND, ADRENAL; GLAND, MAMMARY; HEART; KIDNEY; LARGE INTESTINE, COLON; LIVER; LUNG; LYMPH NODE, ILIAC; NERVE, SCIATIC; OVARY; PANCREAS; SPINAL CORD; SPLEEN

Histopathology - The following Tissues were Not Examined:

None

Appendix 18

Appendix 3

Individual Macroscopic and Microscopic Pathology: Terminal Day 91±2

32025165

Animal: 1507	Group: 1	Sex: Female
Species: Rat	Strain: Sprague Dawley	
	Dose: 0 vg	
	Removal Reason: Terminal Euthanasia	
	Study Day (Week) of Death: 91 (13)	
		Gross Status: Complete
		Histo Status: Complete

Gross Pathology Animal Details:

No animal details found

Gross Pathology Observations [Correlation]:

No observations found

Any remaining protocol required tissues, which have been examined, have no visible lesions

Gross Pathology - The following Tissues were Not Examined:

None

Histopathology Animal Details:

No animal details found

Histopathology Observations [Correlation]:

LIVER : Infiltration, mononuclear cell; minimal

Histopathology - The following Tissues were Within Normal Limits:

BRAIN; GANGLION, DORSAL ROOT, CERVICAL; GANGLION, DORSAL ROOT, LUMBAR; GANGLION, DORSAL ROOT, THORACIC; GLAND, ADRENAL; GLAND, MAMMARY; HEART; KIDNEY; LARGE INTESTINE, COLON; LUNG; LYMPH NODE, ILIAC; NERVE, SCIATIC; OVARY; PANCREAS; SPINAL CORD; SPLEEN

Histopathology - The following Tissues were Not Examined:

None

DRAFT, NOT FOR SUBMISSION

Appendix 18

Appendix 3

Individual Macroscopic and Microscopic Pathology: Terminal Day 91±2

32025165

Animal:	1508	Group:	1	Sex:	Female
Species:	Rat	Strain:	Sprague Dawley		
		Dose:	0 vg		
		Removal Reason:	Terminal Euthanasia		
		Study Day (Week) of Death:	91 (13)		
				Gross Status:	Complete
				Histo Status:	Complete

Gross Pathology Animal Details:

No animal details found

Gross Pathology Observations [Correlation]:

No observations found

Any remaining protocol required tissues, which have been examined, have no visible lesions

Gross Pathology - The following Tissues were Not Examined:

None

Histopathology Animal Details:

No animal details found

Histopathology Observations [Correlation]:

LIVER : Infiltration, mononuclear cell; minimal
OVARY : Atrophy; minimal

Histopathology - The following Tissues were Within Normal Limits:

BRAIN; GANGLION, DORSAL ROOT, CERVICAL; GANGLION, DORSAL ROOT, LUMBAR; GANGLION, DORSAL ROOT, THORACIC; GLAND, ADRENAL; GLAND, MAMMARY; HEART; KIDNEY; LARGE INTESTINE, COLON; LUNG; LYMPH NODE, ILIAC; NERVE, SCIATIC; PANCREAS; SPINAL CORD; SPLEEN

Histopathology - The following Tissues were Not Examined:

None

DRAFT, NOT FOR SUBMISSION

Appendix 18

Appendix 3

Individual Macroscopic and Microscopic Pathology: Terminal Day 91±2

32025165

Animal: 1509	Group: 1	Sex: Female
Species: Rat	Strain: Sprague Dawley	
	Dose: 0 vg	
	Removal Reason: Terminal Euthanasia	
	Study Day (Week) of Death: 92 (14)	
		Gross Status: Complete
		Histo Status: Complete

Gross Pathology Animal Details:

No animal details found

Gross Pathology Observations [Correlation]:

OVARY : Cyst, clear; left : (Comment) 0.3 cm in diameter. (TGL)

Any remaining protocol required tissues, which have been examined, have no visible lesions

Gross Pathology - The following Tissues were Not Examined:

None

Histopathology Animal Details:

No animal details found

Histopathology Observations [Correlation]:

OVARY : Cyst; mild [OVARY : Cyst, clear; left : (Comment) 0.3 cm in diameter. (G)]

Histopathology - The following Tissues were Within Normal Limits:

BRAIN; GANGLION, DORSAL ROOT, CERVICAL; GANGLION, DORSAL ROOT, LUMBAR; GANGLION, DORSAL ROOT, THORACIC; GLAND, ADRENAL; GLAND, MAMMARY; HEART; KIDNEY; LARGE INTESTINE, COLON; LIVER; LUNG; LYMPH NODE, ILIAC; NERVE, SCIATIC; PANCREAS; SPINAL CORD; SPLEEN

Histopathology - The following Tissues were Not Examined:

None

DRAFT, NOT FOR SUBMISSION

Appendix 18

Appendix 3

Individual Macroscopic and Microscopic Pathology: Terminal Day 91±2

32025165

Animal: 1510	Group: 1	Sex: Female
Species: Rat	Strain: Sprague Dawley	
	Dose: 0 vg	
	Removal Reason: Terminal Euthanasia	
	Study Day (Week) of Death: 93 (14)	
		Gross Status: Complete
		Histo Status: Complete

Gross Pathology Animal Details:

No animal details found

Gross Pathology Observations [Correlation]:

No observations found

Any remaining protocol required tissues, which have been examined, have no visible lesions

Gross Pathology - The following Tissues were Not Examined:

None

Histopathology Animal Details:

No animal details found

Histopathology Observations [Correlation]:

No observations found

Histopathology - The following Tissues were Within Normal Limits:

BRAIN; GANGLION, DORSAL ROOT, CERVICAL; GANGLION, DORSAL ROOT, LUMBAR; GANGLION, DORSAL ROOT, THORACIC; GLAND, ADRENAL; GLAND, MAMMARY; HEART; KIDNEY; LARGE INTESTINE, COLON; LIVER; LUNG; LYMPH NODE, ILIAC; NERVE, SCIATIC; OVARY; PANCREAS; SPINAL CORD; SPLEEN

Histopathology - The following Tissues were Not Examined:

None

DRAFT, NOT FOR SUBMISSION

Appendix 18

Appendix 3

Individual Macroscopic and Microscopic Pathology: Terminal Day 91±2

32025165

Animal: 2001	Group: 2		Sex: Male
Species: Rat	Strain: Sprague Dawley		
	Dose: 1.49x10^11 vg		
	Removal Reason: Terminal Euthanasia		
	Study Day (Week) of Death: 89 (13)		
		Gross Status: Complete	
		Histo Status: Complete	

Gross Pathology Animal Details:

No animal details found

Gross Pathology Observations [Correlation]:

No observations found

Any remaining protocol required tissues, which have been examined, have no visible lesions

Gross Pathology - The following Tissues were Not Examined:

None

Histopathology Animal Details:

No animal details found

Histopathology Observations [Correlation]:

GANGLION, DORSAL ROOT, THORACIC : Degeneration; minimal, nerve fiber
NERVE, SCIATIC : Degeneration; minimal, nerve fiber

Histopathology - The following Tissues were Within Normal Limits:

BRAIN; GANGLION, DORSAL ROOT, CERVICAL; GANGLION, DORSAL ROOT, LUMBAR; GLAND, ADRENAL; GLAND, MAMMARY; HEART; KIDNEY; LARGE INTESTINE, COLON; LIVER; LUNG; LYMPH NODE, ILIAC; PANCREAS; SPINAL CORD; SPLEEN; TESTIS, RIGHT

Histopathology - The following Tissues were Not Examined:

None

DRAFT, NOT FOR SUBMISSION

Appendix 18

Appendix 3

Individual Macroscopic and Microscopic Pathology: Terminal Day 91±2

32025165

Animal: 2002	Group: 2	Sex: Male
Species: Rat	Strain: Sprague Dawley	
	Dose: 1.49x10^11 vg	
	Removal Reason: Terminal Euthanasia	
	Study Day (Week) of Death: 90 (13)	
		Gross Status: Complete
		Histo Status: Complete

Gross Pathology Animal Details:

No animal details found

Gross Pathology Observations [Correlation]:

No observations found

Any remaining protocol required tissues, which have been examined, have no visible lesions

Gross Pathology - The following Tissues were Not Examined:

None

Histopathology Animal Details:

No animal details found

Histopathology Observations [Correlation]:

KIDNEY : Infarct; focal, moderate

Histopathology - The following Tissues were Within Normal Limits:

BRAIN; GANGLION, DORSAL ROOT, CERVICAL; GANGLION, DORSAL ROOT, LUMBAR; GANGLION, DORSAL ROOT, THORACIC; GLAND, ADRENAL; GLAND, MAMMARY; HEART; LARGE INTESTINE, COLON; LIVER; LUNG; LYMPH NODE, ILIAC; NERVE, SCIATIC; PANCREAS; SPINAL CORD; SPLEEN; TESTIS, RIGHT

Histopathology - The following Tissues were Not Examined:

None

DRAFT, NOT FOR SUBMISSION

Appendix 18

Appendix 3

Individual Macroscopic and Microscopic Pathology: Terminal Day 91±2

32025165

Animal: 2003	Group: 2	Sex: Male
Species: Rat	Strain: Sprague Dawley	
	Dose: 1.49x10^11 vg	
	Removal Reason: Terminal Euthanasia	
	Study Day (Week) of Death: 90 (13)	
		Gross Status: Complete
		Histo Status: Complete

Gross Pathology Animal Details:

No animal details found

Gross Pathology Observations [Correlation]:

No observations found

Any remaining protocol required tissues, which have been examined, have no visible lesions

Gross Pathology - The following Tissues were Not Examined:

None

Histopathology Animal Details:

No animal details found

Histopathology Observations [Correlation]:

NERVE, SCIATIC : Degeneration; minimal, nerve fiber

Histopathology - The following Tissues were Within Normal Limits:

BRAIN; GANGLION, DORSAL ROOT, CERVICAL; GANGLION, DORSAL ROOT, LUMBAR; GANGLION, DORSAL ROOT, THORACIC; GLAND, ADRENAL; GLAND, MAMMARY; HEART; KIDNEY; LARGE INTESTINE, COLON; LIVER; LUNG; LYMPH NODE, ILIAC; PANCREAS; SPINAL CORD; SPLEEN; TESTIS, RIGHT

Histopathology - The following Tissues were Not Examined:

None

DRAFT, NOT FOR SUBMISSION

Appendix 18

Appendix 3

Individual Macroscopic and Microscopic Pathology: Terminal Day 91±2

32025165

Animal: 2004	Group: 2	Sex: Male
Species: Rat	Strain: Sprague Dawley	
	Dose: 1.49x10^11 vg	
	Removal Reason: Terminal Euthanasia	
	Study Day (Week) of Death: 90 (13)	
		Gross Status: Complete
		Histo Status: Complete

Gross Pathology Animal Details:

No animal details found

Gross Pathology Observations [Correlation]:

No observations found

Any remaining protocol required tissues, which have been examined, have no visible lesions

Gross Pathology - The following Tissues were Not Examined:

None

Histopathology Animal Details:

No animal details found

Histopathology Observations [Correlation]:

SPINAL CORD : Degeneration; minimal, nerve fiber
TESTIS, RIGHT : Hypospermatogenesis; mild

Histopathology - The following Tissues were Within Normal Limits:

BRAIN; GANGLION, DORSAL ROOT, CERVICAL; GANGLION, DORSAL ROOT, LUMBAR; GANGLION, DORSAL ROOT, THORACIC; GLAND, ADRENAL; GLAND, MAMMARY; HEART; KIDNEY; LARGE INTESTINE, COLON; LIVER; LUNG; LYMPH NODE, ILIAC; NERVE, SCIATIC; PANCREAS; SPLEEN

Histopathology - The following Tissues were Not Examined:

None

DRAFT, NOT FOR SUBMISSION

Appendix 18

Appendix 3

Individual Macroscopic and Microscopic Pathology: Terminal Day 91±2

32025165

Animal: 2005	Group: 2	Sex: Male
Species: Rat	Strain: Sprague Dawley	
	Dose: 1.49x10^11 vg	
	Removal Reason: Terminal Euthanasia	
	Study Day (Week) of Death: 91 (13)	
		Gross Status: Complete
		Histo Status: Complete

Gross Pathology Animal Details:

No animal details found

Gross Pathology Observations [Correlation]:

No observations found

Any remaining protocol required tissues, which have been examined, have no visible lesions

Gross Pathology - The following Tissues were Not Examined:

None

Histopathology Animal Details:

No animal details found

Histopathology Observations [Correlation]:

GANGLION, DORSAL ROOT, LUMBAR : Infiltration, mononuclear cell; minimal
GANGLION, DORSAL ROOT, LUMBAR : Degeneration; minimal, nerve fiber
GLAND, ADRENAL : (Note) One Of A Pair Available For Evaluation.
NERVE, SCIATIC : Degeneration; minimal, nerve fiber

Histopathology - The following Tissues were Within Normal Limits:

BRAIN; GANGLION, DORSAL ROOT, CERVICAL; GANGLION, DORSAL ROOT, THORACIC; GLAND, ADRENAL; GLAND, MAMMARY; HEART; KIDNEY; LARGE INTESTINE, COLON; LIVER; LUNG; LYMPH NODE, ILIAC; PANCREAS; SPINAL CORD; SPLEEN; TESTIS, RIGHT

Histopathology - The following Tissues were Not Examined:

None

DRAFT, NOT FOR SUBMISSION

Appendix 18

Appendix 3

Individual Macroscopic and Microscopic Pathology: Terminal Day 91±2

32025165

Animal: 2006	Group: 2	Sex: Male
Species: Rat	Strain: Sprague Dawley	
	Dose: 1.49x10^11 vg	
	Removal Reason: Terminal Euthanasia	
	Study Day (Week) of Death: 91 (13)	
		Gross Status: Complete
		Histo Status: Complete

Gross Pathology Animal Details:

No animal details found

Gross Pathology Observations [Correlation]:

No observations found

Any remaining protocol required tissues, which have been examined, have no visible lesions

Gross Pathology - The following Tissues were Not Examined:

None

Histopathology Animal Details:

No animal details found

Histopathology Observations [Correlation]:

No observations found

Histopathology - The following Tissues were Within Normal Limits:

BRAIN; GANGLION, DORSAL ROOT, CERVICAL; GANGLION, DORSAL ROOT, LUMBAR; GANGLION, DORSAL ROOT, THORACIC; GLAND, ADRENAL; GLAND, MAMMARY; HEART; KIDNEY; LARGE INTESTINE, COLON; LIVER; LUNG; LYMPH NODE, ILIAC; NERVE, SCIATIC; PANCREAS; SPINAL CORD; SPLEEN; TESTIS, RIGHT

Histopathology - The following Tissues were Not Examined:

None

DRAFT, NOT FOR SUBMISSION

Appendix 18

Appendix 3

Individual Macroscopic and Microscopic Pathology: Terminal Day 91±2

32025165

Animal: 2007	Group: 2	Sex: Male
Species: Rat	Strain: Sprague Dawley	
	Dose: 1.49x10^11 vg	
	Removal Reason: Terminal Euthanasia	
	Study Day (Week) of Death: 91 (13)	
		Gross Status: Complete
		Histo Status: Complete

Gross Pathology Animal Details:

No animal details found

Gross Pathology Observations [Correlation]:

No observations found

Any remaining protocol required tissues, which have been examined, have no visible lesions

Gross Pathology - The following Tissues were Not Examined:

None

Histopathology Animal Details:

No animal details found

Histopathology Observations [Correlation]:

GLAND, ADRENAL : (Note) One Of A Pair Available For Evaluation.

Histopathology - The following Tissues were Within Normal Limits:

BRAIN; GANGLION, DORSAL ROOT, CERVICAL; GANGLION, DORSAL ROOT, LUMBAR; GANGLION, DORSAL ROOT, THORACIC; GLAND, ADRENAL; GLAND, MAMMARY; HEART; KIDNEY; LARGE INTESTINE, COLON; LIVER; LUNG; LYMPH NODE, ILIAC; NERVE, SCIATIC; PANCREAS; SPINAL CORD; SPLEEN; TESTIS, RIGHT

Histopathology - The following Tissues were Not Examined:

None

DRAFT, NOT FOR SUBMISSION

Appendix 18

Appendix 3

Individual Macroscopic and Microscopic Pathology: Terminal Day 91±2

32025165

Animal: 2008	Group: 2	Sex: Male
Species: Rat	Strain: Sprague Dawley	
	Dose: 1.49x10^11 vg	
	Removal Reason: Terminal Euthanasia	
	Study Day (Week) of Death: 91 (13)	
		Gross Status: Complete
		Histo Status: Complete

Gross Pathology Animal Details:

No animal details found

Gross Pathology Observations [Correlation]:

No observations found

Any remaining protocol required tissues, which have been examined, have no visible lesions

Gross Pathology - The following Tissues were Not Examined:

None

Histopathology Animal Details:

No animal details found

Histopathology Observations [Correlation]:

GLAND, ADRENAL : (Note) One Of A Pair Available For Evaluation.
PANCREAS : Fibrosis; mild, islet of langerhans

Histopathology - The following Tissues were Within Normal Limits:

BRAIN; GANGLION, DORSAL ROOT, CERVICAL; GANGLION, DORSAL ROOT, LUMBAR; GANGLION, DORSAL ROOT, THORACIC; GLAND, ADRENAL; GLAND, MAMMARY; HEART; KIDNEY; LARGE INTESTINE, COLON; LIVER; LUNG; LYMPH NODE, ILIAC; NERVE, SCIATIC; SPINAL CORD; SPLEEN; TESTIS, RIGHT

Histopathology - The following Tissues were Not Examined:

None

DRAFT, NOT FOR SUBMISSION

Appendix 18

Appendix 3

Individual Macroscopic and Microscopic Pathology: Terminal Day 91±2

32025165

Animal: 2009	Group: 2	Sex: Male
Species: Rat	Strain: Sprague Dawley	
	Dose: 1.49x10^11 vg	
	Removal Reason: Terminal Euthanasia	
	Study Day (Week) of Death: 92 (14)	
		Gross Status: Complete
		Histo Status: Complete

Gross Pathology Animal Details:

No animal details found

Gross Pathology Observations [Correlation]:

No observations found

Any remaining protocol required tissues, which have been examined, have no visible lesions

Gross Pathology - The following Tissues were Not Examined:

None

Histopathology Animal Details:

No animal details found

Histopathology Observations [Correlation]:

GANGLION, DORSAL ROOT, LUMBAR : Infiltration, mononuclear cell; minimal

Histopathology - The following Tissues were Within Normal Limits:

BRAIN; GANGLION, DORSAL ROOT, CERVICAL; GANGLION, DORSAL ROOT, THORACIC; GLAND, ADRENAL; GLAND, MAMMARY; HEART; KIDNEY; LARGE INTESTINE, COLON; LIVER; LUNG; LYMPH NODE, ILIAC; NERVE, SCIATIC; PANCREAS; SPINAL CORD; SPLEEN; TESTIS, RIGHT

Histopathology - The following Tissues were Not Examined:

None

DRAFT, NOT FOR SUBMISSION

Appendix 18

Appendix 3

Individual Macroscopic and Microscopic Pathology: Terminal Day 91±2

32025165

Animal: 2010	Group: 2	Sex: Male
Species: Rat	Strain: Sprague Dawley	
	Dose: 1.49x10^11 vg	
	Removal Reason: Terminal Euthanasia	
	Study Day (Week) of Death: 92 (14)	
		Gross Status: Complete
		Histo Status: Complete

Gross Pathology Animal Details:

No animal details found

Gross Pathology Observations [Correlation]:

No observations found

Any remaining protocol required tissues, which have been examined, have no visible lesions

Gross Pathology - The following Tissues were Not Examined:

None

Histopathology Animal Details:

No animal details found

Histopathology Observations [Correlation]:

NERVE, SCIATIC : Degeneration; minimal, nerve fiber
SPINAL CORD : (Comment) Lumbar, dorsal funiculus and nerve root
SPINAL CORD : Degeneration; minimal, nerve fiber

Histopathology - The following Tissues were Within Normal Limits:

BRAIN; GANGLION, DORSAL ROOT, CERVICAL; GANGLION, DORSAL ROOT, LUMBAR; GANGLION, DORSAL ROOT, THORACIC; GLAND, ADRENAL; GLAND, MAMMARY; HEART; KIDNEY; LARGE INTESTINE, COLON; LIVER; LUNG; LYMPH NODE, ILIAC; PANCREAS; SPLEEN; TESTIS, RIGHT

Histopathology - The following Tissues were Not Examined:

None

DRAFT, NOT FOR SUBMISSION

Appendix 18

Appendix 3

Individual Macroscopic and Microscopic Pathology: Terminal Day 91±2

32025165

Animal: 2501	Group: 2	Sex: Female
Species: Rat	Strain: Sprague Dawley	
	Dose: 1.49x10^11 vg	
	Removal Reason: Terminal Euthanasia	
	Study Day (Week) of Death: 91 (13)	
		Gross Status: Complete
		Histo Status: Complete

Gross Pathology Animal Details:

No animal details found

Gross Pathology Observations [Correlation]:

No observations found

Any remaining protocol required tissues, which have been examined, have no visible lesions

Gross Pathology - The following Tissues were Not Examined:

None

Histopathology Animal Details:

No animal details found

Histopathology Observations [Correlation]:

GANGLION, DORSAL ROOT, LUMBAR : Degeneration; minimal, nerve fiber

Histopathology - The following Tissues were Within Normal Limits:

BRAIN; GANGLION, DORSAL ROOT, CERVICAL; GANGLION, DORSAL ROOT, THORACIC; GLAND, ADRENAL; GLAND, MAMMARY; HEART; KIDNEY; LARGE INTESTINE, COLON; LIVER; LUNG; LYMPH NODE, ILIAC; NERVE, SCIATIC; OVARY; PANCREAS; SPINAL CORD; SPLEEN

Histopathology - The following Tissues were Not Examined:

None

DRAFT, NOT FOR SUBMISSION

Appendix 18

Appendix 3

Individual Macroscopic and Microscopic Pathology: Terminal Day 91±2

32025165

Animal: 2502	Group: 2	Sex: Female
Species: Rat	Strain: Sprague Dawley	
	Dose: 1.49x10^11 vg	
	Removal Reason: Terminal Euthanasia	
	Study Day (Week) of Death: 91 (13)	
		Gross Status: Complete
		Histo Status: Complete

Gross Pathology Animal Details:

No animal details found

Gross Pathology Observations [Correlation]:

No observations found

Any remaining protocol required tissues, which have been examined, have no visible lesions

Gross Pathology - The following Tissues were Not Examined:

None

Histopathology Animal Details:

No animal details found

Histopathology Observations [Correlation]:

GANGLION, DORSAL ROOT, LUMBAR : (Note) One Of A Pair Available For Evaluation.

Histopathology - The following Tissues were Within Normal Limits:

BRAIN; GANGLION, DORSAL ROOT, CERVICAL; GANGLION, DORSAL ROOT, LUMBAR; GANGLION, DORSAL ROOT, THORACIC; GLAND, ADRENAL; GLAND, MAMMARY; HEART; KIDNEY; LARGE INTESTINE, COLON; LIVER; LUNG; LYMPH NODE, ILIAC; NERVE, SCIATIC; OVARY; PANCREAS; SPINAL CORD; SPLEEN

Histopathology - The following Tissues were Not Examined:

None

DRAFT, NOT FOR SUBMISSION

Appendix 18

Appendix 3

Individual Macroscopic and Microscopic Pathology: Terminal Day 91±2

32025165

Animal: 2503	Group: 2	Sex: Female
Species: Rat	Strain: Sprague Dawley	
	Dose: 1.49x10^11 vg	
	Removal Reason: Terminal Euthanasia	
	Study Day (Week) of Death: 91 (13)	
		Gross Status: Complete
		Histo Status: Complete

Gross Pathology Animal Details:

No animal details found

Gross Pathology Observations [Correlation]:

No observations found

Any remaining protocol required tissues, which have been examined, have no visible lesions

Gross Pathology - The following Tissues were Not Examined:

None

Histopathology Animal Details:

No animal details found

Histopathology Observations [Correlation]:

GANGLION, DORSAL ROOT, LUMBAR : Infiltration, mononuclear cell; minimal

Histopathology - The following Tissues were Within Normal Limits:

BRAIN; GANGLION, DORSAL ROOT, CERVICAL; GANGLION, DORSAL ROOT, THORACIC; GLAND, ADRENAL; GLAND, MAMMARY; HEART; KIDNEY; LARGE INTESTINE, COLON; LIVER; LUNG; LYMPH NODE, ILIAC; NERVE, SCIATIC; OVARY; PANCREAS; SPINAL CORD; SPLEEN

Histopathology - The following Tissues were Not Examined:

None

DRAFT, NOT FOR SUBMISSION

Appendix 18
Appendix 3

Individual Macroscopic and Microscopic Pathology: Terminal Day 91±2

32025165

Animal: 2504	Group: 2			Sex: Female
Species: Rat	Strain: Sprague Dawley			
	Dose: 1.49x10^11 vg			
	Removal Reason: Terminal Euthanasia			
	Study Day (Week) of Death: 91 (13)			
			Gross Status:	Complete
			Histo Status:	Complete

Gross Pathology Animal Details:

No animal details found

Gross Pathology Observations [Correlation]:

No observations found

Any remaining protocol required tissues, which have been examined, have no visible lesions

Gross Pathology - The following Tissues were Not Examined:

None

Histopathology Animal Details:

No animal details found

Histopathology Observations [Correlation]:

GANGLION, DORSAL ROOT, LUMBAR : Infiltration, mononuclear cell; minimal
SPINAL CORD : (Comment) Lumbar, nerve root
SPINAL CORD : Degeneration; minimal, nerve fiber

Histopathology - The following Tissues were Within Normal Limits:

BRAIN; GANGLION, DORSAL ROOT, CERVICAL; GANGLION, DORSAL ROOT, THORACIC; GLAND, ADRENAL; GLAND, MAMMARY; HEART; KIDNEY; LARGE INTESTINE, COLON; LIVER; LUNG; LYMPH NODE, ILIAC; NERVE, SCIATIC; OVARY; PANCREAS; SPLEEN

Histopathology - The following Tissues were Not Examined:

None

DRAFT, NOT FOR SUBMISSION

Appendix 18

Appendix 3

Individual Macroscopic and Microscopic Pathology: Terminal Day 91±2

32025165

Animal: 2505	Group: 2	Sex: Female
Species: Rat	Strain: Sprague Dawley	
	Dose: 1.49x10^11 vg	
	Removal Reason: Terminal Euthanasia	
	Study Day (Week) of Death: 91 (13)	
		Gross Status: Complete
		Histo Status: Complete

Gross Pathology Animal Details:

No animal details found

Gross Pathology Observations [Correlation]:

UTERUS : Cyst, clear; horn : (Comment) 0.7 cm in diameter. (TGL)

Any remaining protocol required tissues, which have been examined, have no visible lesions

Gross Pathology - The following Tissues were Not Examined:

None

Histopathology Animal Details:

No animal details found

Histopathology Observations [Correlation]:

UTERUS : Cyst; mild [UTERUS : Cyst, clear; horn : (Comment) 0.7 cm in diameter. (G)]

Histopathology - The following Tissues were Within Normal Limits:

BRAIN; GANGLION, DORSAL ROOT, CERVICAL; GANGLION, DORSAL ROOT, LUMBAR; GANGLION, DORSAL ROOT, THORACIC; GLAND, ADRENAL; GLAND, MAMMARY; HEART; KIDNEY; LARGE INTESTINE, COLON; LIVER; LUNG; LYMPH NODE, ILIAC; NERVE, SCIATIC; OVARY; PANCREAS; SPINAL CORD; SPLEEN

Histopathology - The following Tissues were Not Examined:

None

DRAFT, NOT FOR SUBMISSION

Appendix 18

Appendix 3

Individual Macroscopic and Microscopic Pathology: Terminal Day 91±2

32025165

Animal: 2506	Group: 2	Sex: Female
Species: Rat	Strain: Sprague Dawley	
	Dose: 1.49x10^11 vg	
	Removal Reason: Terminal Euthanasia	
	Study Day (Week) of Death: 91 (13)	
		Gross Status: Complete
		Histo Status: Complete

Gross Pathology Animal Details:

No animal details found

Gross Pathology Observations [Correlation]:

No observations found

Any remaining protocol required tissues, which have been examined, have no visible lesions

Gross Pathology - The following Tissues were Not Examined:

None

Histopathology Animal Details:

No animal details found

Histopathology Observations [Correlation]:

NERVE, SCIATIC : Degeneration; minimal, nerve fiber
OVARY : Atrophy; minimal

Histopathology - The following Tissues were Within Normal Limits:

BRAIN; GANGLION, DORSAL ROOT, CERVICAL; GANGLION, DORSAL ROOT, LUMBAR; GANGLION, DORSAL ROOT, THORACIC; GLAND, ADRENAL; GLAND, MAMMARY; HEART; KIDNEY; LARGE INTESTINE, COLON; LIVER; LUNG; LYMPH NODE, ILIAC; PANCREAS; SPINAL CORD; SPLEEN

Histopathology - The following Tissues were Not Examined:

None

Appendix 18

Appendix 3

Individual Macroscopic and Microscopic Pathology: Terminal Day 91±2

32025165

Animal:	2507	Group:	2	Sex:	Female
Species:	Rat	Strain:	Sprague Dawley		
		Dose:	1.49x10^11 vg		
		Removal Reason:	Terminal Euthanasia		
		Study Day (Week) of Death:	92 (14)		
				Gross Status:	Complete
				Histo Status:	Complete

Gross Pathology Animal Details:

No animal details found

Gross Pathology Observations [Correlation]:

No observations found

Any remaining protocol required tissues, which have been examined, have no visible lesions

Gross Pathology - The following Tissues were Not Examined:

None

Histopathology Animal Details:

No animal details found

Histopathology Observations [Correlation]:

GANGLION, DORSAL ROOT, LUMBAR : Infiltration, mononuclear cell; minimal
GANGLION, DORSAL ROOT, LUMBAR : Degeneration; minimal, nerve fiber
OVARY : Atrophy; moderate
SPINAL CORD : (Comment) Lumbar, dorsal funiculus, nerve root
SPINAL CORD : Degeneration; minimal, nerve fiber

Histopathology - The following Tissues were Within Normal Limits:

BRAIN; GANGLION, DORSAL ROOT, CERVICAL; GANGLION, DORSAL ROOT, THORACIC; GLAND, ADRENAL; GLAND, MAMMARY; HEART; KIDNEY; LARGE INTESTINE, COLON; LIVER; LUNG; LYMPH NODE, ILIAC; NERVE, SCIATIC; PANCREAS; SPLEEN

Histopathology - The following Tissues were Not Examined:

None

DRAFT, NOT FOR SUBMISSION

Appendix 18

Appendix 3

Individual Macroscopic and Microscopic Pathology: Terminal Day 91±2

32025165

Animal: 2508	Group: 2	Sex: Female
Species: Rat	Strain: Sprague Dawley	
	Dose: 1.49x10^11 vg	
	Removal Reason: Terminal Euthanasia	
	Study Day (Week) of Death: 92 (14)	
		Gross Status: Complete
		Histo Status: Complete

Gross Pathology Animal Details:

No animal details found

Gross Pathology Observations [Correlation]:

No observations found

Any remaining protocol required tissues, which have been examined, have no visible lesions

Gross Pathology - The following Tissues were Not Examined:

None

Histopathology Animal Details:

No animal details found

Histopathology Observations [Correlation]:

OVARY : Atrophy; minimal

Histopathology - The following Tissues were Within Normal Limits:

BRAIN; GANGLION, DORSAL ROOT, CERVICAL; GANGLION, DORSAL ROOT, LUMBAR; GANGLION, DORSAL ROOT, THORACIC; GLAND, ADRENAL; GLAND, MAMMARY; HEART; KIDNEY; LARGE INTESTINE, COLON; LIVER; LUNG; LYMPH NODE, ILIAC; NERVE, SCIATIC; PANCREAS; SPINAL CORD; SPLEEN

Histopathology - The following Tissues were Not Examined:

None

DRAFT, NOT FOR SUBMISSION

Appendix 18

Appendix 3

Individual Macroscopic and Microscopic Pathology: Terminal Day 91±2

32025165

Animal: 2509	Group: 2	Sex: Female
Species: Rat	Strain: Sprague Dawley	
	Dose: 1.49x10^11 vg	
	Removal Reason: Terminal Euthanasia	
	Study Day (Week) of Death: 92 (14)	
		Gross Status: Complete
		Histo Status: Complete

Gross Pathology Animal Details:

No animal details found

Gross Pathology Observations [Correlation]:

No observations found

Any remaining protocol required tissues, which have been examined, have no visible lesions

Gross Pathology - The following Tissues were Not Examined:

None

Histopathology Animal Details:

No animal details found

Histopathology Observations [Correlation]:

GANGLION, DORSAL ROOT, LUMBAR : Infiltration, mononuclear cell; minimal
OVARY : Atrophy; moderate

Histopathology - The following Tissues were Within Normal Limits:

BRAIN; GANGLION, DORSAL ROOT, CERVICAL; GANGLION, DORSAL ROOT, THORACIC; GLAND, ADRENAL; GLAND, MAMMARY; HEART; KIDNEY; LARGE INTESTINE, COLON; LIVER; LUNG; LYMPH NODE, ILIAC; NERVE, SCIATIC; PANCREAS; SPINAL CORD; SPLEEN

Histopathology - The following Tissues were Not Examined:

None

DRAFT, NOT FOR SUBMISSION

Appendix 18

Appendix 3

Individual Macroscopic and Microscopic Pathology: Terminal Day 91±2

32025165

Animal: 2510	Group: 2	Sex: Female
Species: Rat	Strain: Sprague Dawley	
	Dose: 1.49x10^11 vg	
	Removal Reason: Terminal Euthanasia	
	Study Day (Week) of Death: 93 (14)	
		Gross Status: Complete
		Histo Status: Complete

Gross Pathology Animal Details:

No animal details found

Gross Pathology Observations [Correlation]:

No observations found

Any remaining protocol required tissues, which have been examined, have no visible lesions

Gross Pathology - The following Tissues were Not Examined:

None

Histopathology Animal Details:

No animal details found

Histopathology Observations [Correlation]:

GANGLION, DORSAL ROOT, LUMBAR : Infiltration, mononuclear cell; minimal
GANGLION, DORSAL ROOT, LUMBAR : Degeneration; minimal, nerve fiber
NERVE, SCIATIC : Degeneration; minimal, nerve fiber

Histopathology - The following Tissues were Within Normal Limits:

BRAIN; GANGLION, DORSAL ROOT, CERVICAL; GANGLION, DORSAL ROOT, THORACIC; GLAND, ADRENAL; GLAND, MAMMARY; HEART; KIDNEY; LARGE INTESTINE, COLON; LIVER; LUNG; LYMPH NODE, ILIAC; OVARY; PANCREAS; SPINAL CORD; SPLEEN

Histopathology - The following Tissues were Not Examined:

None

DRAFT, NOT FOR SUBMISSION

Appendix 18

Appendix 3

Individual Macroscopic and Microscopic Pathology: Terminal Day 91±2

32025165

Animal:	3001	Group:	3	Sex:	Male
Species:	Rat	Strain:	Sprague Dawley		
		Dose:	4.47x10^11 vg		
		Removal Reason:	Terminal Euthanasia		
		Study Day (Week) of Death:	89 (13)		
				Gross Status:	Complete
				Histo Status:	Complete

Gross Pathology Animal Details:

No animal details found

Gross Pathology Observations [Correlation]:

No observations found

Any remaining protocol required tissues, which have been examined, have no visible lesions

Gross Pathology - The following Tissues were Not Examined:

None

Histopathology Animal Details:

No animal details found

Histopathology Observations [Correlation]:

GANGLION, DORSAL ROOT, LUMBAR : Infiltration, mononuclear cell; minimal
GANGLION, DORSAL ROOT, LUMBAR : Degeneration; minimal, nerve fiber
NERVE, SCIATIC : Degeneration; minimal, nerve fiber

Histopathology - The following Tissues were Within Normal Limits:

BRAIN; GANGLION, DORSAL ROOT, CERVICAL; GANGLION, DORSAL ROOT, THORACIC; GLAND, ADRENAL; GLAND, MAMMARY; HEART; KIDNEY; LARGE INTESTINE, COLON; LIVER; LUNG; LYMPH NODE, ILIAC; PANCREAS; SPINAL CORD; SPLEEN; TESTIS, RIGHT

Histopathology - The following Tissues were Not Examined:

None

DRAFT, NOT FOR SUBMISSION

Appendix 18

Appendix 3

Individual Macroscopic and Microscopic Pathology: Terminal Day 91±2

32025165

Animal:	3002	Group:	3	Sex:	Male
Species:	Rat	Strain:	Sprague Dawley		
		Dose:	4.47x10^11 vg		
		Removal Reason:	Terminal Euthanasia		
		Study Day (Week) of Death:	89 (13)		
				Gross Status:	Complete
				Histo Status:	Complete

Gross Pathology Animal Details:

No animal details found

Gross Pathology Observations [Correlation]:

No observations found

Any remaining protocol required tissues, which have been examined, have no visible lesions

Gross Pathology - The following Tissues were Not Examined:

None

Histopathology Animal Details:

No animal details found

Histopathology Observations [Correlation]:

GANGLION, DORSAL ROOT, LUMBAR : Infiltration, mononuclear cell; minimal
GANGLION, DORSAL ROOT, LUMBAR : Degeneration; minimal, nerve fiber
GANGLION, DORSAL ROOT, LUMBAR : Degeneration/necrosis; neuronal, minimal
GANGLION, DORSAL ROOT, LUMBAR : Aggregate; minimal, glial cell
NERVE, SCIATIC : Degeneration; minimal, nerve fiber
SPINAL CORD : (Comment) Lumbar, dorsal funiculus
SPINAL CORD : Degeneration; minimal, nerve fiber

Histopathology - The following Tissues were Within Normal Limits:

BRAIN; GANGLION, DORSAL ROOT, CERVICAL; GANGLION, DORSAL ROOT, THORACIC; GLAND, ADRENAL; GLAND, MAMMARY; HEART; KIDNEY; LARGE INTESTINE, COLON; LIVER; LUNG; LYMPH NODE, ILIAC; PANCREAS; SPLEEN; TESTIS, RIGHT

Histopathology - The following Tissues were Not Examined:

None

DRAFT, NOT FOR SUBMISSION

Appendix 18

Appendix 3

Individual Macroscopic and Microscopic Pathology: Terminal Day 91±2

32025165

Animal:	3003	Group:	3	Sex:	Male
Species:	Rat	Strain:	Sprague Dawley		
		Dose:	4.47x10^11 vg		
		Removal Reason:	Terminal Euthanasia		
		Study Day (Week) of Death:	90 (13)		
				Gross Status:	Complete
				Histo Status:	Complete

Gross Pathology Animal Details:

No animal details found

Gross Pathology Observations [Correlation]:

No observations found

Any remaining protocol required tissues, which have been examined, have no visible lesions

Gross Pathology - The following Tissues were Not Examined:

None

Histopathology Animal Details:

No animal details found

Histopathology Observations [Correlation]:

GANGLION, DORSAL ROOT, LUMBAR : Infiltration, mononuclear cell; minimal
GANGLION, DORSAL ROOT, LUMBAR : Degeneration; minimal, nerve fiber
GANGLION, DORSAL ROOT, LUMBAR : Aggregate; minimal, glial cell
NERVE, SCIATIC : Degeneration; minimal, nerve fiber

Histopathology - The following Tissues were Within Normal Limits:

BRAIN; GANGLION, DORSAL ROOT, CERVICAL; GANGLION, DORSAL ROOT, THORACIC; GLAND, ADRENAL; GLAND, MAMMARY; HEART; KIDNEY; LARGE INTESTINE, COLON; LIVER; LUNG; LYMPH NODE, ILIAC; PANCREAS; SPINAL CORD; SPLEEN; TESTIS, RIGHT

Histopathology - The following Tissues were Not Examined:

None

DRAFT, NOT FOR SUBMISSION

Appendix 18

Appendix 3

Individual Macroscopic and Microscopic Pathology: Terminal Day 91±2

32025165

Animal: 3004	Group: 3	Sex: Male
Species: Rat	Strain: Sprague Dawley	
	Dose: 4.47x10^11 vg	
	Removal Reason: Terminal Euthanasia	
	Study Day (Week) of Death: 90 (13)	
		Gross Status: Complete
		Histo Status: Complete

Gross Pathology Animal Details:

No animal details found

Gross Pathology Observations [Correlation]:

No observations found

Any remaining protocol required tissues, which have been examined, have no visible lesions

Gross Pathology - The following Tissues were Not Examined:

None

Histopathology Animal Details:

No animal details found

Histopathology Observations [Correlation]:

GANGLION, DORSAL ROOT, LUMBAR : Infiltration, mononuclear cell; mild
GANGLION, DORSAL ROOT, LUMBAR : Degeneration; mild, nerve fiber
GANGLION, DORSAL ROOT, LUMBAR : Aggregate; minimal, glial cell
NERVE, SCIATIC : Degeneration; minimal, nerve fiber
SPINAL CORD : (Comment) Lumbar, dorsal funiculus
SPINAL CORD : Degeneration; minimal, nerve fiber

Histopathology - The following Tissues were Within Normal Limits:

BRAIN; GANGLION, DORSAL ROOT, CERVICAL; GANGLION, DORSAL ROOT, THORACIC; GLAND, ADRENAL; GLAND, MAMMARY; HEART; KIDNEY; LARGE INTESTINE, COLON; LIVER; LUNG; LYMPH NODE, ILIAC; PANCREAS; SPLEEN; TESTIS, RIGHT

Histopathology - The following Tissues were Not Examined:

None

DRAFT, NOT FOR SUBMISSION

Appendix 18

Appendix 3

Individual Macroscopic and Microscopic Pathology: Terminal Day 91±2

32025165

Animal: 3005	Group: 3	Sex: Male
Species: Rat	Strain: Sprague Dawley	
	Dose: 4.47x10^11 vg	
	Removal Reason: Terminal Euthanasia	
	Study Day (Week) of Death: 90 (13)	
		Gross Status: Complete
		Histo Status: Complete

Gross Pathology Animal Details:

No animal details found

Gross Pathology Observations [Correlation]:

No observations found

Any remaining protocol required tissues, which have been examined, have no visible lesions

Gross Pathology - The following Tissues were Not Examined:

None

Histopathology Animal Details:

No animal details found

Histopathology Observations [Correlation]:

GANGLION, DORSAL ROOT, LUMBAR : Infiltration, mononuclear cell; minimal
GANGLION, DORSAL ROOT, THORACIC : Autophagy; minimal
PANCREAS : Atrophy; minimal
PANCREAS : Fibrosis; minimal, islet of langerhans
PANCREAS : Infiltration, mononuclear cell; minimal

Histopathology - The following Tissues were Within Normal Limits:

BRAIN; GANGLION, DORSAL ROOT, CERVICAL; GLAND, ADRENAL; GLAND, MAMMARY; HEART; KIDNEY; LARGE INTESTINE, COLON; LIVER; LUNG; LYMPH NODE, ILIAC; NERVE, SCIATIC; SPINAL CORD; SPLEEN; TESTIS, RIGHT

Histopathology - The following Tissues were Not Examined:

None

DRAFT, NOT FOR SUBMISSION

Appendix 18

Appendix 3

Individual Macroscopic and Microscopic Pathology: Terminal Day 91±2

32025165

Animal:	3006	Group:	3	Sex:	Male
Species:	Rat	Strain:	Sprague Dawley		
		Dose:	4.47x10^11 vg		
		Removal Reason:	Terminal Euthanasia		
		Study Day (Week) of Death:	91 (13)		
				Gross Status:	Complete
				Histo Status:	Complete

Gross Pathology Animal Details:

No animal details found

Gross Pathology Observations [Correlation]:

No observations found

Any remaining protocol required tissues, which have been examined, have no visible lesions

Gross Pathology - The following Tissues were Not Examined:

None

Histopathology Animal Details:

No animal details found

Histopathology Observations [Correlation]:

LIVER : Infiltration, mononuclear cell; minimal

Histopathology - The following Tissues were Within Normal Limits:

BRAIN; GANGLION, DORSAL ROOT, CERVICAL; GANGLION, DORSAL ROOT, LUMBAR; GANGLION, DORSAL ROOT, THORACIC; GLAND, ADRENAL; GLAND, MAMMARY; HEART; KIDNEY; LARGE INTESTINE, COLON; LUNG; LYMPH NODE, ILIAC; NERVE, SCIATIC; PANCREAS; SPINAL CORD; SPLEEN; TESTIS, RIGHT

Histopathology - The following Tissues were Not Examined:

None

Appendix 18

Appendix 3

Individual Macroscopic and Microscopic Pathology: Terminal Day 91±2

32025165

Animal: 3007	Group: 3		Sex: Male
Species: Rat	Strain: Sprague Dawley		
	Dose: 4.47x10^11 vg		
	Removal Reason: Terminal Euthanasia		
	Study Day (Week) of Death: 91 (13)		
		Gross Status:	Complete
		Histo Status:	Complete

Gross Pathology Animal Details:

No animal details found

Gross Pathology Observations [Correlation]:

No observations found

Any remaining protocol required tissues, which have been examined, have no visible lesions

Gross Pathology - The following Tissues were Not Examined:

None

Histopathology Animal Details:

No animal details found

Histopathology Observations [Correlation]:

No observations found

Histopathology - The following Tissues were Within Normal Limits:

BRAIN; GANGLION, DORSAL ROOT, CERVICAL; GANGLION, DORSAL ROOT, LUMBAR; GANGLION, DORSAL ROOT, THORACIC; GLAND, ADRENAL; GLAND, MAMMARY; HEART; KIDNEY; LARGE INTESTINE, COLON; LIVER; LUNG; LYMPH NODE, ILIAC; NERVE, SCIATIC; PANCREAS; SPINAL CORD; SPLEEN; TESTIS, RIGHT

Histopathology - The following Tissues were Not Examined:

None

DRAFT, NOT FOR SUBMISSION

Appendix 18

Appendix 3

Individual Macroscopic and Microscopic Pathology: Terminal Day 91±2

32025165

Animal: 3008	Group: 3	Sex: Male
Species: Rat	Strain: Sprague Dawley	
	Dose: 4.47x10^11 vg	
	Removal Reason: Terminal Euthanasia	
	Study Day (Week) of Death: 91 (13)	
		Gross Status: Complete
		Histo Status: Complete

Gross Pathology Animal Details:

No animal details found

Gross Pathology Observations [Correlation]:

No observations found

Any remaining protocol required tissues, which have been examined, have no visible lesions

Gross Pathology - The following Tissues were Not Examined:

None

Histopathology Animal Details:

No animal details found

Histopathology Observations [Correlation]:

No observations found

Histopathology - The following Tissues were Within Normal Limits:

BRAIN; GANGLION, DORSAL ROOT, CERVICAL; GANGLION, DORSAL ROOT, LUMBAR; GANGLION, DORSAL ROOT, THORACIC; GLAND, ADRENAL; GLAND, MAMMARY; HEART; KIDNEY; LARGE INTESTINE, COLON; LIVER; LUNG; LYMPH NODE, ILIAC; NERVE, SCIATIC; PANCREAS; SPINAL CORD; SPLEEN; TESTIS, RIGHT

Histopathology - The following Tissues were Not Examined:

None

DRAFT, NOT FOR SUBMISSION

Appendix 18

Appendix 3

Individual Macroscopic and Microscopic Pathology: Terminal Day 91±2

32025165

Animal: 3009	Group: 3	Sex: Male
Species: Rat	Strain: Sprague Dawley	
	Dose: 4.47x10^11 vg	
	Removal Reason: Terminal Euthanasia	
	Study Day (Week) of Death: 92 (14)	
		Gross Status: Complete
		Histo Status: Complete

Gross Pathology Animal Details:

No animal details found

Gross Pathology Observations [Correlation]:

KIDNEY : Cyst, clear; left : (Comment) 0.3 cm in diameter. (TGL)

Any remaining protocol required tissues, which have been examined, have no visible lesions

Gross Pathology - The following Tissues were Not Examined:

None

Histopathology Animal Details:

No animal details found

Histopathology Observations [Correlation]:

GANGLION, DORSAL ROOT, LUMBAR : Infiltration, mononuclear cell; minimal
GANGLION, DORSAL ROOT, LUMBAR : Degeneration; minimal, nerve fiber
KIDNEY : Cyst; mild [KIDNEY : Cyst, clear; left : (Comment) 0.3 cm in diameter. (G)]
SPINAL CORD : (Comment) Lumbar, dorsal funiculus
SPINAL CORD : Degeneration; minimal, nerve fiber

Histopathology - The following Tissues were Within Normal Limits:

BRAIN; GANGLION, DORSAL ROOT, CERVICAL; GANGLION, DORSAL ROOT, THORACIC; GLAND, ADRENAL; GLAND, MAMMARY; HEART; LARGE INTESTINE, COLON; LIVER; LUNG; LYMPH NODE, ILIAC; NERVE, SCIATIC; PANCREAS; SPLEEN; TESTIS, RIGHT

Histopathology - The following Tissues were Not Examined:

None

DRAFT, NOT FOR SUBMISSION

Appendix 18

Appendix 3

Individual Macroscopic and Microscopic Pathology: Terminal Day 91±2

32025165

Animal: 3010	Group: 3	Sex: Male
Species: Rat	Strain: Sprague Dawley	
	Dose: 4.47x10^11 vg	
	Removal Reason: Terminal Euthanasia	
	Study Day (Week) of Death: 92 (14)	
		Gross Status: Complete
		Histo Status: Complete

Gross Pathology Animal Details:

No animal details found

Gross Pathology Observations [Correlation]:

No observations found

Any remaining protocol required tissues, which have been examined, have no visible lesions

Gross Pathology - The following Tissues were Not Examined:

None

Histopathology Animal Details:

No animal details found

Histopathology Observations [Correlation]:

GANGLION, DORSAL ROOT, LUMBAR : Degeneration; minimal, nerve fiber
NERVE, SCIATIC : Degeneration; minimal, nerve fiber
PANCREAS : Atrophy; mild
PANCREAS : Fibrosis; mild, islet of langerhans
PANCREAS : Infiltration, mononuclear cell; mild

Histopathology - The following Tissues were Within Normal Limits:

BRAIN; GANGLION, DORSAL ROOT, CERVICAL; GANGLION, DORSAL ROOT, THORACIC; GLAND, ADRENAL; GLAND, MAMMARY; HEART; KIDNEY; LARGE INTESTINE, COLON; LIVER; LUNG; LYMPH NODE, ILIAC; SPINAL CORD; SPLEEN; TESTIS, RIGHT

Histopathology - The following Tissues were Not Examined:

None

DRAFT, NOT FOR SUBMISSION

Appendix 18

Appendix 3

Individual Macroscopic and Microscopic Pathology: Terminal Day 91±2

32025165

Animal: 3501	Group: 3	Sex: Female
Species: Rat	Strain: Sprague Dawley	
	Dose: 4.47x10^11 vg	
	Removal Reason: Terminal Euthanasia	
	Study Day (Week) of Death: 91 (13)	
		Gross Status: Complete
		Histo Status: Complete

Gross Pathology Animal Details:

No animal details found

Gross Pathology Observations [Correlation]:

No observations found

Any remaining protocol required tissues, which have been examined, have no visible lesions

Gross Pathology - The following Tissues were Not Examined:

None

Histopathology Animal Details:

No animal details found

Histopathology Observations [Correlation]:

SPINAL CORD : (Comment) Lumbar and thoracic, dorsal funiculus
SPINAL CORD : Degeneration; minimal, nerve fiber

Histopathology - The following Tissues were Within Normal Limits:

BRAIN; GANGLION, DORSAL ROOT, CERVICAL; GANGLION, DORSAL ROOT, LUMBAR; GANGLION, DORSAL ROOT, THORACIC; GLAND, ADRENAL; GLAND, MAMMARY; HEART; KIDNEY; LARGE INTESTINE, COLON; LIVER; LUNG; LYMPH NODE, ILIAC; NERVE, SCIATIC; OVARY; PANCREAS; SPLEEN

Histopathology - The following Tissues were Not Examined:

None

Appendix 18

Appendix 3

Individual Macroscopic and Microscopic Pathology: Terminal Day 91±2

32025165

Animal: 3502	Group: 3	Sex: Female
Species: Rat	Strain: Sprague Dawley	
	Dose: 4.47x10^11 vg	
	Removal Reason: Terminal Euthanasia	
	Study Day (Week) of Death: 91 (13)	
		Gross Status: Complete
		Histo Status: Complete

Gross Pathology Animal Details:

No animal details found

Gross Pathology Observations [Correlation]:

No observations found

Any remaining protocol required tissues, which have been examined, have no visible lesions

Gross Pathology - The following Tissues were Not Examined:

None

Histopathology Animal Details:

No animal details found

Histopathology Observations [Correlation]:

GANGLION, DORSAL ROOT, LUMBAR : Infiltration, mononuclear cell; minimal

Histopathology - The following Tissues were Within Normal Limits:

BRAIN; GANGLION, DORSAL ROOT, CERVICAL; GANGLION, DORSAL ROOT, THORACIC; GLAND, ADRENAL; HEART; KIDNEY; LARGE INTESTINE, COLON; LIVER; LUNG; LYMPH NODE, ILIAC; NERVE, SCIATIC; OVARY; PANCREAS; SPINAL CORD; SPLEEN

Histopathology - The following Tissues were Not Examined:

GLAND, MAMMARY - Not Present In Section.

DRAFT, NOT FOR SUBMISSION

Appendix 18

Appendix 3

Individual Macroscopic and Microscopic Pathology: Terminal Day 91±2

32025165

Animal: 3503	Group: 3	Sex: Female
Species: Rat	Strain: Sprague Dawley	
	Dose: 4.47x10^11 vg	
	Removal Reason: Terminal Euthanasia	
	Study Day (Week) of Death: 91 (13)	
		Gross Status: Complete
		Histo Status: Complete

Gross Pathology Animal Details:

No animal details found

Gross Pathology Observations [Correlation]:

No observations found

Any remaining protocol required tissues, which have been examined, have no visible lesions

Gross Pathology - The following Tissues were Not Examined:

None

Histopathology Animal Details:

No animal details found

Histopathology Observations [Correlation]:

GANGLION, DORSAL ROOT, CERVICAL : Infiltration, mononuclear cell; minimal
GANGLION, DORSAL ROOT, CERVICAL : Degeneration; minimal, nerve fiber
GANGLION, DORSAL ROOT, LUMBAR : Infiltration, mononuclear cell; minimal
GANGLION, DORSAL ROOT, LUMBAR : Degeneration; mild, nerve fiber
NERVE, SCIATIC : Degeneration; minimal, nerve fiber
SPINAL CORD : (Comment) Lumbar dorsal funiculus
SPINAL CORD : Degeneration; minimal, nerve fiber

Histopathology - The following Tissues were Within Normal Limits:

BRAIN; GANGLION, DORSAL ROOT, THORACIC; GLAND, ADRENAL; GLAND, MAMMARY; HEART; KIDNEY; LARGE INTESTINE, COLON; LIVER; LUNG; LYMPH NODE, ILIAC; OVARY; PANCREAS; SPLEEN

Histopathology - The following Tissues were Not Examined:

None

DRAFT, NOT FOR SUBMISSION

Appendix 18

Appendix 3

Individual Macroscopic and Microscopic Pathology: Terminal Day 91±2

32025165

Animal: 3504	Group: 3	Sex: Female
Species: Rat	Strain: Sprague Dawley	
	Dose: 4.47x10^11 vg	
	Removal Reason: Terminal Euthanasia	
	Study Day (Week) of Death: 91 (13)	
		Gross Status: Complete
		Histo Status: Complete

Gross Pathology Animal Details:

No animal details found

Gross Pathology Observations [Correlation]:

No observations found

Any remaining protocol required tissues, which have been examined, have no visible lesions

Gross Pathology - The following Tissues were Not Examined:

None

Histopathology Animal Details:

No animal details found

Histopathology Observations [Correlation]:

No observations found

Histopathology - The following Tissues were Within Normal Limits:

BRAIN; GANGLION, DORSAL ROOT, CERVICAL; GANGLION, DORSAL ROOT, LUMBAR; GANGLION, DORSAL ROOT, THORACIC; GLAND, ADRENAL; GLAND, MAMMARY; HEART; KIDNEY; LARGE INTESTINE, COLON; LIVER; LUNG; LYMPH NODE, ILIAC; NERVE, SCIATIC; OVARY; PANCREAS; SPINAL CORD; SPLEEN

Histopathology - The following Tissues were Not Examined:

None

DRAFT, NOT FOR SUBMISSION

Appendix 18

Appendix 3

Individual Macroscopic and Microscopic Pathology: Terminal Day 91±2

32025165

Animal: 3505	Group: 3		Sex: Female
Species: Rat	Strain: Sprague Dawley		
	Dose: 4.47x10^11 vg		
	Removal Reason: Terminal Euthanasia		
	Study Day (Week) of Death: 91 (13)		
		Gross Status: Complete	
		Histo Status: Complete	

Gross Pathology Animal Details:

No animal details found

Gross Pathology Observations [Correlation]:

No observations found

Any remaining protocol required tissues, which have been examined, have no visible lesions

Gross Pathology - The following Tissues were Not Examined:

None

Histopathology Animal Details:

No animal details found

Histopathology Observations [Correlation]:

NERVE, SCIATIC : Degeneration; minimal, nerve fiber

Histopathology - The following Tissues were Within Normal Limits:

BRAIN; GANGLION, DORSAL ROOT, CERVICAL; GANGLION, DORSAL ROOT, LUMBAR; GANGLION, DORSAL ROOT, THORACIC; GLAND, ADRENAL; GLAND, MAMMARY; HEART; KIDNEY; LARGE INTESTINE, COLON; LIVER; LUNG; LYMPH NODE, ILIAC; OVARY; PANCREAS; SPINAL CORD; SPLEEN

Histopathology - The following Tissues were Not Examined:

None

Appendix 18

Appendix 3

Individual Macroscopic and Microscopic Pathology: Terminal Day 91±2

32025165

Animal: 3506	Group: 3	Sex: Female
Species: Rat	Strain: Sprague Dawley	
	Dose: 4.47x10^11 vg	
	Removal Reason: Terminal Euthanasia	
	Study Day (Week) of Death: 91 (13)	
		Gross Status: Complete
		Histo Status: Complete

Gross Pathology Animal Details:

No animal details found

Gross Pathology Observations [Correlation]:

No observations found

Any remaining protocol required tissues, which have been examined, have no visible lesions

Gross Pathology - The following Tissues were Not Examined:

None

Histopathology Animal Details:

No animal details found

Histopathology Observations [Correlation]:

GANGLION, DORSAL ROOT, LUMBAR : Infiltration, mononuclear cell; mild
GANGLION, DORSAL ROOT, LUMBAR : Degeneration; mild, nerve fiber
GANGLION, DORSAL ROOT, LUMBAR : Degeneration/necrosis; neuronal, minimal
GANGLION, DORSAL ROOT, LUMBAR : Aggregate; mild, glial cell
NERVE, SCIATIC : Degeneration; minimal, nerve fiber
OVARY : Atrophy; mild
SPINAL CORD : (Comment) Lumbar, thoracic, dorsal funiculus
SPINAL CORD : Degeneration; mild, nerve fiber

Histopathology - The following Tissues were Within Normal Limits:

BRAIN; GANGLION, DORSAL ROOT, CERVICAL; GANGLION, DORSAL ROOT, THORACIC; GLAND, ADRENAL; GLAND, MAMMARY; HEART; KIDNEY; LARGE INTESTINE, COLON; LIVER; LUNG; LYMPH NODE, ILIAC; PANCREAS; SPLEEN

Histopathology - The following Tissues were Not Examined:

None

DRAFT, NOT FOR SUBMISSION

Appendix 18

Appendix 3

Individual Macroscopic and Microscopic Pathology: Terminal Day 91±2

32025165

Animal: 3507	Group: 3	Sex: Female
Species: Rat	Strain: Sprague Dawley	
	Dose: 4.47x10^11 vg	
	Removal Reason: Terminal Euthanasia	
	Study Day (Week) of Death: 92 (14)	
		Gross Status: Complete
		Histo Status: Complete

Gross Pathology Animal Details:

No animal details found

Gross Pathology Observations [Correlation]:

No observations found

Any remaining protocol required tissues, which have been examined, have no visible lesions

Gross Pathology - The following Tissues were Not Examined:

None

Histopathology Animal Details:

No animal details found

Histopathology Observations [Correlation]:

GANGLION, DORSAL ROOT, LUMBAR : Infiltration, mononuclear cell; minimal
GANGLION, DORSAL ROOT, LUMBAR : Degeneration; minimal, nerve fiber
GANGLION, DORSAL ROOT, LUMBAR : Aggregate; minimal, glial cell
GLAND, ADRENAL : (Note) Only Cortex Available For Evaluation.
NERVE, SCIATIC : Degeneration; minimal, nerve fiber
OVARY : Atrophy; minimal
SPINAL CORD : (Comment) Lumbar, dorsal funiculus
SPINAL CORD : Degeneration; minimal, nerve fiber

Histopathology - The following Tissues were Within Normal Limits:

BRAIN; GANGLION, DORSAL ROOT, CERVICAL; GANGLION, DORSAL ROOT, THORACIC; GLAND, ADRENAL; GLAND, MAMMARY; HEART; KIDNEY; LARGE INTESTINE, COLON; LIVER; LUNG; LYMPH NODE, ILIAC; PANCREAS; SPLEEN

Histopathology - The following Tissues were Not Examined:

None

DRAFT, NOT FOR SUBMISSION

Appendix 18

Appendix 3

Individual Macroscopic and Microscopic Pathology: Terminal Day 91±2

32025165

Animal: 3508	Group: 3	Sex: Female
Species: Rat	Strain: Sprague Dawley	
	Dose: 4.47x10^11 vg	
	Removal Reason: Terminal Euthanasia	
	Study Day (Week) of Death: 92 (14)	
		Gross Status: Complete
		Histo Status: Complete

Gross Pathology Animal Details:

No animal details found

Gross Pathology Observations [Correlation]:

No observations found

Any remaining protocol required tissues, which have been examined, have no visible lesions

Gross Pathology - The following Tissues were Not Examined:

None

Histopathology Animal Details:

No animal details found

Histopathology Observations [Correlation]:

GANGLION, DORSAL ROOT, LUMBAR : Degeneration; minimal, nerve fiber
SPINAL CORD : (Comment) Lumbar, dorsal funiculus and nerve root
SPINAL CORD : Degeneration; mild, nerve fiber

Histopathology - The following Tissues were Within Normal Limits:

BRAIN; GANGLION, DORSAL ROOT, CERVICAL; GANGLION, DORSAL ROOT, THORACIC; GLAND, ADRENAL; GLAND, MAMMARY; HEART; KIDNEY; LARGE INTESTINE, COLON; LIVER; LUNG; LYMPH NODE, ILIAC; NERVE, SCIATIC; OVARY; PANCREAS; SPLEEN

Histopathology - The following Tissues were Not Examined:

None

DRAFT, NOT FOR SUBMISSION

Appendix 18

Appendix 3

Individual Macroscopic and Microscopic Pathology: Terminal Day 91±2

32025165

Animal: 3509	Group: 3	Sex: Female
Species: Rat	Strain: Sprague Dawley	
	Dose: 4.47x10^11 vg	
	Removal Reason: Terminal Euthanasia	
	Study Day (Week) of Death: 92 (14)	
		Gross Status: Complete
		Histo Status: Complete

Gross Pathology Animal Details:

No animal details found

Gross Pathology Observations [Correlation]:

THYMUS : Discoloration, dark; red (TGL)

Any remaining protocol required tissues, which have been examined, have no visible lesions

Gross Pathology - The following Tissues were Not Examined:

None

Histopathology Animal Details:

No animal details found

Histopathology Observations [Correlation]:

GLAND, ADRENAL : (Note) Only Cortex Available For Evaluation.
NERVE, SCIATIC : Degeneration; minimal, nerve fiber
SPINAL CORD : (Comment) Lumbar, thoracic, cervical, dorsal funiculus
SPINAL CORD : Degeneration; minimal, nerve fiber
THYMUS : Hemorrhage; mild [THYMUS : Discoloration, dark; red (G)]

Histopathology - The following Tissues were Within Normal Limits:

BRAIN; GANGLION, DORSAL ROOT, CERVICAL; GANGLION, DORSAL ROOT, LUMBAR; GANGLION, DORSAL ROOT, THORACIC; GLAND, ADRENAL; GLAND, MAMMARY; HEART; KIDNEY; LARGE INTESTINE, COLON; LIVER; LUNG; LYMPH NODE, ILIAC; OVARY; PANCREAS; SPLEEN

Histopathology - The following Tissues were Not Examined:

None

DRAFT, NOT FOR SUBMISSION

Appendix 18

Appendix 3

Individual Macroscopic and Microscopic Pathology: Terminal Day 91±2

32025165

Animal: 3510	Group: 3	Sex: Female
Species: Rat	Strain: Sprague Dawley	
	Dose: 4.47x10^11 vg	
	Removal Reason: Terminal Euthanasia	
	Study Day (Week) of Death: 93 (14)	
		Gross Status: Complete
		Histo Status: Complete

Gross Pathology Animal Details:

No animal details found

Gross Pathology Observations [Correlation]:

No observations found

Any remaining protocol required tissues, which have been examined, have no visible lesions

Gross Pathology - The following Tissues were Not Examined:

None

Histopathology Animal Details:

No animal details found

Histopathology Observations [Correlation]:

No observations found

Histopathology - The following Tissues were Within Normal Limits:

BRAIN; GANGLION, DORSAL ROOT, CERVICAL; GANGLION, DORSAL ROOT, LUMBAR; GANGLION, DORSAL ROOT, THORACIC; GLAND, ADRENAL; GLAND, MAMMARY; HEART; KIDNEY; LARGE INTESTINE, COLON; LIVER; LUNG; LYMPH NODE, ILIAC; NERVE, SCIATIC; OVARY; PANCREAS; SPINAL CORD; SPLEEN

Histopathology - The following Tissues were Not Examined:

None

DRAFT, NOT FOR SUBMISSION

Appendix 18

Appendix 3

Individual Macroscopic and Microscopic Pathology: Terminal Day 91±2

32025165

Animal: 4006	Group: 4	Sex: Male
Species: Rat	Strain: Sprague Dawley	
	Dose: 1.49x10^12 vg	
	Removal Reason: Terminal Euthanasia	
	Study Day (Week) of Death: 89 (13)	
		Gross Status: Complete
		Histo Status: Complete

Gross Pathology Animal Details:

No animal details found

Gross Pathology Observations [Correlation]:

No observations found

Any remaining protocol required tissues, which have been examined, have no visible lesions

Gross Pathology - The following Tissues were Not Examined:

None

Histopathology Animal Details:

No animal details found

Histopathology Observations [Correlation]:

GANGLION, DORSAL ROOT, LUMBAR : Infiltration, mononuclear cell; minimal
GANGLION, DORSAL ROOT, LUMBAR : Degeneration; mild, nerve fiber
GANGLION, DORSAL ROOT, LUMBAR : Aggregate; minimal, glial cell
NERVE, SCIATIC : Degeneration; minimal, nerve fiber
PANCREAS : Atrophy; minimal
SPINAL CORD : (Comment) Lumbar, nerve root
SPINAL CORD : Degeneration; minimal, nerve fiber

Histopathology - The following Tissues were Within Normal Limits:

BRAIN; GANGLION, DORSAL ROOT, CERVICAL; GANGLION, DORSAL ROOT, THORACIC; GLAND, ADRENAL; GLAND, MAMMARY; HEART; KIDNEY; LARGE INTESTINE, COLON; LIVER; LUNG; LYMPH NODE, ILIAC; SPLEEN; TESTIS, RIGHT

Histopathology - The following Tissues were Not Examined:

None

DRAFT, NOT FOR SUBMISSION

Appendix 18

Appendix 3

Individual Macroscopic and Microscopic Pathology: Terminal Day 91±2

32025165

Animal: 4007	Group: 4	Sex: Male
Species: Rat	Strain: Sprague Dawley	
	Dose: 1.49x10^12 vg	
	Removal Reason: Terminal Euthanasia	
	Study Day (Week) of Death: 89 (13)	
		Gross Status: Complete
		Histo Status: Complete

Gross Pathology Animal Details:

No animal details found

Gross Pathology Observations [Correlation]:

No observations found

Any remaining protocol required tissues, which have been examined, have no visible lesions

Gross Pathology - The following Tissues were Not Examined:

None

Histopathology Animal Details:

No animal details found

Histopathology Observations [Correlation]:

GANGLION, DORSAL ROOT, LUMBAR : Infiltration, mononuclear cell; minimal
GANGLION, DORSAL ROOT, LUMBAR : Degeneration; mild, nerve fiber
GANGLION, DORSAL ROOT, LUMBAR : Degeneration/necrosis; neuronal, minimal
GANGLION, DORSAL ROOT, LUMBAR : Aggregate; minimal, glial cell
NERVE, SCIATIC : Degeneration; minimal, nerve fiber
SPINAL CORD : (Comment) Lumbar, nerve root
SPINAL CORD : Degeneration; minimal, nerve fiber

Histopathology - The following Tissues were Within Normal Limits:

BRAIN; GANGLION, DORSAL ROOT, CERVICAL; GANGLION, DORSAL ROOT, THORACIC; GLAND, ADRENAL; GLAND, MAMMARY; HEART; KIDNEY; LARGE INTESTINE, COLON; LIVER; LUNG; LYMPH NODE, ILIAC; PANCREAS; SPLEEN; TESTIS, RIGHT

Histopathology - The following Tissues were Not Examined:

None

Appendix 18

Appendix 3

Individual Macroscopic and Microscopic Pathology: Terminal Day 91±2

32025165

Animal: 4008	Group: 4	Sex: Male
Species: Rat	Strain: Sprague Dawley	
	Dose: 1.49x10^12 vg	
	Removal Reason: Terminal Euthanasia	
	Study Day (Week) of Death: 90 (13)	
		Gross Status: Complete
		Histo Status: Complete

Gross Pathology Animal Details:

No animal details found

Gross Pathology Observations [Correlation]:

No observations found

Any remaining protocol required tissues, which have been examined, have no visible lesions

Gross Pathology - The following Tissues were Not Examined:

None

Histopathology Animal Details:

No animal details found

Histopathology Observations [Correlation]:

GANGLION, DORSAL ROOT, LUMBAR : Degeneration; minimal, nerve fiber
GANGLION, DORSAL ROOT, LUMBAR : Aggregate; minimal, glial cell
GANGLION, DORSAL ROOT, THORACIC : Degeneration; minimal, nerve fiber
NERVE, SCIATIC : Degeneration; minimal, nerve fiber
SPINAL CORD : (Comment) Lumbar, nerve root and dorsal funiculus
SPINAL CORD : Degeneration; mild, nerve fiber

Histopathology - The following Tissues were Within Normal Limits:

BRAIN; GANGLION, DORSAL ROOT, CERVICAL; GLAND, ADRENAL; GLAND, MAMMARY; HEART; KIDNEY; LARGE INTESTINE, COLON; LIVER; LUNG; LYMPH NODE, ILIAC; PANCREAS; SPLEEN; TESTIS, RIGHT

Histopathology - The following Tissues were Not Examined:

None

DRAFT, NOT FOR SUBMISSION

Appendix 18

Appendix 3

Individual Macroscopic and Microscopic Pathology: Terminal Day 91±2

32025165

Animal:	4009	Group:	4	Sex:	Male
Species:	Rat	Strain:	Sprague Dawley		
		Dose:	1.49x10^12 vg		
		Removal Reason:	Terminal Euthanasia		
		Study Day (Week) of Death:	90 (13)		
				Gross Status:	Complete
				Histo Status:	Complete

Gross Pathology Animal Details:

No animal details found

Gross Pathology Observations [Correlation]:

No observations found

Any remaining protocol required tissues, which have been examined, have no visible lesions

Gross Pathology - The following Tissues were Not Examined:

None

Histopathology Animal Details:

No animal details found

Histopathology Observations [Correlation]:

GANGLION, DORSAL ROOT, LUMBAR : Degeneration; minimal, nerve fiber
GANGLION, DORSAL ROOT, THORACIC : Degeneration; minimal, nerve fiber
LIVER : Vacuolation; hepatocellular, marked
LIVER : Eosinophilic globules; hepatocellular, marked
NERVE, SCIATIC : Degeneration; minimal, nerve fiber

Histopathology - The following Tissues were Within Normal Limits:

BRAIN; GANGLION, DORSAL ROOT, CERVICAL; GLAND, ADRENAL; GLAND, MAMMARY; HEART; KIDNEY; LARGE INTESTINE, COLON; LUNG; LYMPH NODE, ILIAC; PANCREAS; SPINAL CORD; SPLEEN; TESTIS, RIGHT

Histopathology - The following Tissues were Not Examined:

None

DRAFT, NOT FOR SUBMISSION

Appendix 18

Appendix 3

Individual Macroscopic and Microscopic Pathology: Terminal Day 91±2

32025165

Animal: 4010	Group: 4	Sex: Male
Species: Rat	Strain: Sprague Dawley	
	Dose: 1.49x10^12 vg	
	Removal Reason: Terminal Euthanasia	
	Study Day (Week) of Death: 91 (13)	
		Gross Status: Complete
		Histo Status: Complete

Gross Pathology Animal Details:

No animal details found

Gross Pathology Observations [Correlation]:

No observations found

Any remaining protocol required tissues, which have been examined, have no visible lesions

Gross Pathology - The following Tissues were Not Examined:

None

Histopathology Animal Details:

No animal details found

Histopathology Observations [Correlation]:

GANGLION, DORSAL ROOT, LUMBAR : Infiltration, mononuclear cell; mild
GANGLION, DORSAL ROOT, LUMBAR : Degeneration; minimal, nerve fiber
GANGLION, DORSAL ROOT, LUMBAR : Aggregate; minimal, glial cell
GANGLION, DORSAL ROOT, THORACIC : Degeneration; minimal, nerve fiber
PANCREAS : Atrophy; minimal
PANCREAS : Fibrosis; minimal, islet of langerhans
PANCREAS : Infiltration, mononuclear cell; minimal
SPINAL CORD : (Comment) Lumbar, nerve root
SPINAL CORD : Degeneration; mild, nerve fiber

Histopathology - The following Tissues were Within Normal Limits:

BRAIN; GANGLION, DORSAL ROOT, CERVICAL; GLAND, ADRENAL; GLAND, MAMMARY; HEART; KIDNEY; LARGE INTESTINE, COLON; LIVER; LUNG; LYMPH NODE, ILIAC; NERVE, SCIATIC; SPLEEN; TESTIS, RIGHT

Histopathology - The following Tissues were Not Examined:

None

DRAFT, NOT FOR SUBMISSION

Appendix 18

Appendix 3

Individual Macroscopic and Microscopic Pathology: Terminal Day 91±2

32025165

Animal: 4011	Group: 4	Sex: Male
Species: Rat	Strain: Sprague Dawley	
	Dose: 1.49x10^12 vg	
	Removal Reason: Terminal Euthanasia	
	Study Day (Week) of Death: 91 (13)	
		Gross Status: Complete
		Histo Status: Complete

Gross Pathology Animal Details:

No animal details found

Gross Pathology Observations [Correlation]:

No observations found

Any remaining protocol required tissues, which have been examined, have no visible lesions

Gross Pathology - The following Tissues were Not Examined:

None

Histopathology Animal Details:

No animal details found

Histopathology Observations [Correlation]:

GANGLION, DORSAL ROOT, LUMBAR : Infiltration, mononuclear cell; minimal
GANGLION, DORSAL ROOT, LUMBAR : Degeneration; minimal, nerve fiber
GANGLION, DORSAL ROOT, LUMBAR : Aggregate; minimal, glial cell
GLAND, ADRENAL : (Note) One Of A Pair Available For Evaluation.
HEART : Infiltration, mononuclear cell; minimal
KIDNEY : Infarct; multifocal, moderate
NERVE, SCIATIC : Degeneration; minimal, nerve fiber
PANCREAS : Fibrosis; minimal, islet of langerhans
SPINAL CORD : (Comment) Lumbar, nerve root
SPINAL CORD : Degeneration; minimal, nerve fiber

Histopathology - The following Tissues were Within Normal Limits:

BRAIN; GANGLION, DORSAL ROOT, CERVICAL; GANGLION, DORSAL ROOT, THORACIC; GLAND, ADRENAL; GLAND, MAMMARY; LARGE INTESTINE, COLON; LIVER; LUNG; LYMPH NODE, ILIAC; SPLEEN; TESTIS, RIGHT

Histopathology - The following Tissues were Not Examined:

None

DRAFT, NOT FOR SUBMISSION

Appendix 18

Appendix 3

Individual Macroscopic and Microscopic Pathology: Terminal Day 91±2

32025165

Animal: 4012	Group: 4	Sex: Male
Species: Rat	Strain: Sprague Dawley<br>Dose: 1.49x10^12 vg<br>Removal Reason: Terminal Euthanasia<br>Study Day (Week) of Death: 91 (13)	
		Gross Status: Complete<br>Histo Status: Complete

Gross Pathology Animal Details:

No animal details found

Gross Pathology Observations [Correlation]:

No observations found

Any remaining protocol required tissues, which have been examined, have no visible lesions

Gross Pathology - The following Tissues were Not Examined:

None

Histopathology Animal Details:

No animal details found

Histopathology Observations [Correlation]:

GANGLION, DORSAL ROOT, LUMBAR : Degeneration; mild, nerve fiber
GLAND, ADRENAL : (Note) One Of A Pair Available For Evaluation.
NERVE, SCIATIC : Degeneration; minimal, nerve fiber

Histopathology - The following Tissues were Within Normal Limits:

BRAIN; GANGLION, DORSAL ROOT, CERVICAL; GANGLION, DORSAL ROOT, THORACIC; GLAND, ADRENAL; GLAND, MAMMARY; HEART; KIDNEY; LARGE INTESTINE, COLON; LIVER; LUNG; LYMPH NODE, ILIAC; PANCREAS; SPINAL CORD; SPLEEN; TESTIS, RIGHT

Histopathology - The following Tissues were Not Examined:

None

DRAFT, NOT FOR SUBMISSION

Appendix 18

Appendix 3

Individual Macroscopic and Microscopic Pathology: Terminal Day 91±2

32025165

Animal:	4013	Group:	4	Sex:	Male
Species:	Rat	Strain:	Sprague Dawley		
		Dose:	1.49x10^12 vg		
		Removal Reason:	Terminal Euthanasia		
		Study Day (Week) of Death:	92 (14)		
				Gross Status:	Complete
				Histo Status:	Complete

Gross Pathology Animal Details:

No animal details found

Gross Pathology Observations [Correlation]:

No observations found

Any remaining protocol required tissues, which have been examined, have no visible lesions

Gross Pathology - The following Tissues were Not Examined:

None

Histopathology Animal Details:

No animal details found

Histopathology Observations [Correlation]:

GANGLION, DORSAL ROOT, LUMBAR : Infiltration, mononuclear cell; minimal
GANGLION, DORSAL ROOT, LUMBAR : Degeneration; minimal, nerve fiber
GANGLION, DORSAL ROOT, LUMBAR : Aggregate; minimal, glial cell
GLAND, ADRENAL : (Note) One Of A Pair Available For Evaluation.
NERVE, SCIATIC : Degeneration; minimal, nerve fiber
SPINAL CORD : (Comment) Lumbar, dorsal funiculus
SPINAL CORD : Degeneration; minimal, nerve fiber

Histopathology - The following Tissues were Within Normal Limits:

BRAIN; GANGLION, DORSAL ROOT, CERVICAL; GANGLION, DORSAL ROOT, THORACIC; GLAND, ADRENAL; GLAND, MAMMARY; HEART; KIDNEY; LARGE INTESTINE, COLON; LIVER; LUNG; LYMPH NODE, ILIAC; PANCREAS; SPLEEN; TESTIS, RIGHT

Histopathology - The following Tissues were Not Examined:

None

DRAFT, NOT FOR SUBMISSION

Appendix 18

Appendix 3

Individual Macroscopic and Microscopic Pathology: Terminal Day 91±2

32025165

Animal: 4014	Group: 4	Sex: Male
Species: Rat	Strain: Sprague Dawley	
	Dose: 1.49x10^12 vg	
	Removal Reason: Terminal Euthanasia	
	Study Day (Week) of Death: 92 (14)	
		Gross Status: Complete
		Histo Status: Complete

Gross Pathology Animal Details:

No animal details found

Gross Pathology Observations [Correlation]:

No observations found

Any remaining protocol required tissues, which have been examined, have no visible lesions

Gross Pathology - The following Tissues were Not Examined:

None

Histopathology Animal Details:

No animal details found

Histopathology Observations [Correlation]:

GANGLION, DORSAL ROOT, CERVICAL : Infiltration, mononuclear cell; minimal
GANGLION, DORSAL ROOT, LUMBAR : Infiltration, mononuclear cell; minimal
GANGLION, DORSAL ROOT, LUMBAR : Degeneration; moderate, nerve fiber
GANGLION, DORSAL ROOT, LUMBAR : Aggregate; minimal, glial cell
HEART : Infiltration, mononuclear cell; mild
NERVE, SCIATIC : Degeneration; minimal, nerve fiber
SPINAL CORD : (Comment) Lumbar, nerve root
SPINAL CORD : Degeneration; minimal, nerve fiber

Histopathology - The following Tissues were Within Normal Limits:

BRAIN; GANGLION, DORSAL ROOT, THORACIC; GLAND, ADRENAL; GLAND, MAMMARY; KIDNEY; LARGE INTESTINE, COLON; LIVER; LUNG; LYMPH NODE, ILIAC; PANCREAS; SPLEEN; TESTIS, RIGHT

Histopathology - The following Tissues were Not Examined:

None

DRAFT, NOT FOR SUBMISSION

Appendix 18
Appendix 3

Individual Macroscopic and Microscopic Pathology: Terminal Day 91±2

32025165

Animal: 4015	Group: 4	Sex: Male
Species: Rat	Strain: Sprague Dawley	
	Dose: 1.49x10^12 vg	
	Removal Reason: Terminal Euthanasia	
	Study Day (Week) of Death: 92 (14)	
		Gross Status: Complete
		Histo Status: Complete

Gross Pathology Animal Details:

No animal details found

Gross Pathology Observations [Correlation]:

LIVER : Nodule; red, medial lobe : (Comment) 0.6 cm in diameter. (TGL)
MUSCLE, DIAPHRAGM : Hernia : (Comment) Portion of median liver lobe found in thoracic cavity.

Any remaining protocol required tissues, which have been examined, have no visible lesions

Gross Pathology - The following Tissues were Not Examined:

None

Histopathology Animal Details:

No animal details found

Histopathology Observations [Correlation]:

GANGLION, DORSAL ROOT, LUMBAR : Infiltration, mononuclear cell; mild
GANGLION, DORSAL ROOT, LUMBAR : Degeneration; mild, nerve fiber
GANGLION, DORSAL ROOT, LUMBAR : Degeneration/necrosis; neuronal, minimal
GANGLION, DORSAL ROOT, LUMBAR : Aggregate; mild, glial cell
LIVER : Hepatodiaphragmatic nodule; mild [LIVER : Nodule; red, medial lobe : (Comment) 0.6 cm in diameter. (G)]
LIVER : Infiltration, mononuclear cell; minimal

Histopathology - The following Tissues were Within Normal Limits:

BRAIN; GANGLION, DORSAL ROOT, CERVICAL; GANGLION, DORSAL ROOT, THORACIC; GLAND, ADRENAL; GLAND, MAMMARY; HEART; KIDNEY; LARGE INTESTINE, COLON; LUNG; LYMPH NODE, ILIAC; NERVE, SCIATIC; PANCREAS; SPINAL CORD; SPLEEN; TESTIS, RIGHT

Histopathology - The following Tissues were Not Examined:

None

DRAFT, NOT FOR SUBMISSION

Appendix 18

Appendix 3

Individual Macroscopic and Microscopic Pathology: Terminal Day 91±2

32025165

Animal: 4506	Group: 4	Sex: Female
Species: Rat	Strain: Sprague Dawley	
	Dose: 1.49x10^12 vg	
	Removal Reason: Terminal Euthanasia	
	Study Day (Week) of Death: 91 (13)	
		Gross Status: Complete
		Histo Status: Complete

Gross Pathology Animal Details:

No animal details found

Gross Pathology Observations [Correlation]:

No observations found

Any remaining protocol required tissues, which have been examined, have no visible lesions

Gross Pathology - The following Tissues were Not Examined:

None

Histopathology Animal Details:

No animal details found

Histopathology Observations [Correlation]:

GANGLION, DORSAL ROOT, LUMBAR : Infiltration, mononuclear cell; minimal
GANGLION, DORSAL ROOT, LUMBAR : Degeneration; minimal, nerve fiber
GANGLION, DORSAL ROOT, LUMBAR : Aggregate; minimal, glial cell
NERVE, SCIATIC : Degeneration; minimal, nerve fiber
SPINAL CORD : (Comment) Lumbar, nerve root
SPINAL CORD : Degeneration; minimal, nerve fiber

Histopathology - The following Tissues were Within Normal Limits:

BRAIN; GANGLION, DORSAL ROOT, CERVICAL; GANGLION, DORSAL ROOT, THORACIC; GLAND, ADRENAL; GLAND, MAMMARY; HEART; KIDNEY; LARGE INTESTINE, COLON; LIVER; LUNG; LYMPH NODE, ILIAC; OVARY; PANCREAS; SPLEEN

Histopathology - The following Tissues were Not Examined:

None

DRAFT, NOT FOR SUBMISSION

Appendix 18

Appendix 3

Individual Macroscopic and Microscopic Pathology: Terminal Day 91±2

32025165

Animal: 4507	Group: 4	Sex: Female
Species: Rat	Strain: Sprague Dawley	
	Dose: 1.49x10^12 vg	
	Removal Reason: Terminal Euthanasia	
	Study Day (Week) of Death: 91 (13)	
		Gross Status: Complete
		Histo Status: Complete

Gross Pathology Animal Details:

No animal details found

Gross Pathology Observations [Correlation]:

No observations found

Any remaining protocol required tissues, which have been examined, have no visible lesions

Gross Pathology - The following Tissues were Not Examined:

None

Histopathology Animal Details:

No animal details found

Histopathology Observations [Correlation]:

GANGLION, DORSAL ROOT, LUMBAR : Infiltration, mononuclear cell; mild
GANGLION, DORSAL ROOT, LUMBAR : Degeneration; moderate, nerve fiber
GANGLION, DORSAL ROOT, LUMBAR : Degeneration/necrosis; neuronal, minimal
GANGLION, DORSAL ROOT, LUMBAR : Aggregate; mild, glial cell
NERVE, SCIATIC : Degeneration; minimal, nerve fiber
SPINAL CORD : (Comment) Lumbar, thoracic, dorsal funiculus and nerve root.
SPINAL CORD : Degeneration; mild, nerve fiber

Histopathology - The following Tissues were Within Normal Limits:

BRAIN; GANGLION, DORSAL ROOT, CERVICAL; GANGLION, DORSAL ROOT, THORACIC; GLAND, ADRENAL; GLAND, MAMMARY; HEART; KIDNEY; LARGE INTESTINE, COLON; LIVER; LUNG; LYMPH NODE, ILIAC; OVARY; PANCREAS; SPLEEN

Histopathology - The following Tissues were Not Examined:

None

DRAFT, NOT FOR SUBMISSION

Appendix 18

Appendix 3

Individual Macroscopic and Microscopic Pathology: Terminal Day 91±2

32025165

Animal: 4508	Group: 4	Sex: Female
Species: Rat	Strain: Sprague Dawley	
	Dose: 1.49x10^12 vg	
	Removal Reason: Terminal Euthanasia	
	Study Day (Week) of Death: 91 (13)	
		Gross Status: Complete
		Histo Status: Complete

Gross Pathology Animal Details:

No animal details found

Gross Pathology Observations [Correlation]:

No observations found

Any remaining protocol required tissues, which have been examined, have no visible lesions

Gross Pathology - The following Tissues were Not Examined:

None

Histopathology Animal Details:

No animal details found

Histopathology Observations [Correlation]:

GANGLION, DORSAL ROOT, LUMBAR : Infiltration, mononuclear cell; minimal
GANGLION, DORSAL ROOT, LUMBAR : Degeneration; minimal, nerve fiber
GANGLION, DORSAL ROOT, LUMBAR : Aggregate; minimal, glial cell
NERVE, SCIATIC : Degeneration; minimal, nerve fiber
OVARY : Atrophy; mild
SPINAL CORD : (Comment) Lumbar, dorsal funiculus, nerve root
SPINAL CORD : Degeneration; minimal, nerve fiber

Histopathology - The following Tissues were Within Normal Limits:

BRAIN; GANGLION, DORSAL ROOT, CERVICAL; GANGLION, DORSAL ROOT, THORACIC; GLAND, ADRENAL; GLAND, MAMMARY; HEART; KIDNEY; LARGE INTESTINE, COLON; LIVER; LUNG; LYMPH NODE, ILIAC; PANCREAS; SPLEEN

Histopathology - The following Tissues were Not Examined:

None

DRAFT, NOT FOR SUBMISSION

Appendix 18

Appendix 3

Individual Macroscopic and Microscopic Pathology: Terminal Day 91±2

32025165

Animal: 4509	Group: 4	Sex: Female
Species: Rat	Strain: Sprague Dawley	
	Dose: 1.49x10^12 vg	
	Removal Reason: Terminal Euthanasia	
	Study Day (Week) of Death: 91 (13)	
		Gross Status: Complete
		Histo Status: Complete

Gross Pathology Animal Details:

No animal details found

Gross Pathology Observations [Correlation]:

No observations found

Any remaining protocol required tissues, which have been examined, have no visible lesions

Gross Pathology - The following Tissues were Not Examined:

None

Histopathology Animal Details:

No animal details found

Histopathology Observations [Correlation]:

GANGLION, DORSAL ROOT, LUMBAR : Degeneration; minimal, nerve fiber
GANGLION, DORSAL ROOT, LUMBAR : Aggregate; minimal, glial cell
GLAND, ADRENAL : (Note) Only Cortex Available For Evaluation.
NERVE, SCIATIC : Degeneration; minimal, nerve fiber
SPINAL CORD : (Comment) Lumbar, thoracic, dorsal funiculus, nerve root
SPINAL CORD : Degeneration; mild, nerve fiber

Histopathology - The following Tissues were Within Normal Limits:

BRAIN; GANGLION, DORSAL ROOT, CERVICAL; GANGLION, DORSAL ROOT, THORACIC; GLAND, ADRENAL; GLAND, MAMMARY; HEART; KIDNEY; LARGE INTESTINE, COLON; LIVER; LUNG; LYMPH NODE, ILIAC; OVARY; SPLEEN

Histopathology - The following Tissues were Not Examined:

PANCREAS - Not Present In Section.

DRAFT, NOT FOR SUBMISSION

Appendix 18

Appendix 3

Individual Macroscopic and Microscopic Pathology: Terminal Day 91±2

32025165

Animal: 4510	Group: 4	Sex: Female
Species: Rat	Strain: Sprague Dawley	
	Dose: 1.49x10^12 vg	
	Removal Reason: Terminal Euthanasia	
	Study Day (Week) of Death: 91 (13)	
		Gross Status: Complete
		Histo Status: Complete

Gross Pathology Animal Details:

No animal details found

Gross Pathology Observations [Correlation]:

No observations found

Any remaining protocol required tissues, which have been examined, have no visible lesions

Gross Pathology - The following Tissues were Not Examined:

None

Histopathology Animal Details:

No animal details found

Histopathology Observations [Correlation]:

GANGLION, DORSAL ROOT, LUMBAR : Infiltration, mononuclear cell; minimal
GANGLION, DORSAL ROOT, LUMBAR : Degeneration; minimal, nerve fiber
GANGLION, DORSAL ROOT, LUMBAR : Aggregate; minimal, glial cell
NERVE, SCIATIC : Degeneration; minimal, nerve fiber
SPINAL CORD : (Comment) Lumbar, thoracic, dorsal funiculus
SPINAL CORD : Degeneration; mild, nerve fiber

Histopathology - The following Tissues were Within Normal Limits:

BRAIN; GANGLION, DORSAL ROOT, CERVICAL; GANGLION, DORSAL ROOT, THORACIC; GLAND, ADRENAL; GLAND, MAMMARY; HEART; KIDNEY; LARGE INTESTINE, COLON; LIVER; LUNG; LYMPH NODE, ILIAC; OVARY; PANCREAS; SPLEEN

Histopathology - The following Tissues were Not Examined:

None

DRAFT, NOT FOR SUBMISSION

Appendix 18

Appendix 3

Individual Macroscopic and Microscopic Pathology: Terminal Day 91±2

32025165

Animal: 4511	Group: 4	Sex: Female
Species: Rat	Strain: Sprague Dawley	
	Dose: 1.49x10^12 vg	
	Removal Reason: Terminal Euthanasia	
	Study Day (Week) of Death: 92 (14)	
		Gross Status: Complete
		Histo Status: Complete

Gross Pathology Animal Details:

No animal details found

Gross Pathology Observations [Correlation]:

No observations found

Any remaining protocol required tissues, which have been examined, have no visible lesions

Gross Pathology - The following Tissues were Not Examined:

None

Histopathology Animal Details:

No animal details found

Histopathology Observations [Correlation]:

GANGLION, DORSAL ROOT, LUMBAR : Infiltration, mononuclear cell; minimal
GANGLION, DORSAL ROOT, LUMBAR : Degeneration; minimal, nerve fiber
GANGLION, DORSAL ROOT, LUMBAR : Aggregate; minimal, glial cell
GANGLION, DORSAL ROOT, THORACIC : Degeneration; mild, nerve fiber
NERVE, SCIATIC : Degeneration; minimal, nerve fiber
OVARY : Atrophy; minimal
SPINAL CORD : (Comment) Lumbar, thoracic, dorsal funiculus
SPINAL CORD : Degeneration; mild, nerve fiber

Histopathology - The following Tissues were Within Normal Limits:

BRAIN; GANGLION, DORSAL ROOT, CERVICAL; GLAND, ADRENAL; GLAND, MAMMARY; HEART; KIDNEY; LARGE INTESTINE, COLON; LIVER; LUNG; LYMPH NODE, ILIAC; PANCREAS; SPLEEN

Histopathology - The following Tissues were Not Examined:

None

DRAFT, NOT FOR SUBMISSION

Appendix 18

Appendix 3

Individual Macroscopic and Microscopic Pathology: Terminal Day 91±2

32025165

Animal:	4512	Group:	4	Sex:	Female
Species:	Rat	Strain:	Sprague Dawley		
		Dose:	1.49x10^12 vg		
		Removal Reason:	Terminal Euthanasia		
		Study Day (Week) of Death:	92 (14)		
				Gross Status:	Complete
				Histo Status:	Complete

Gross Pathology Animal Details:

No animal details found

Gross Pathology Observations [Correlation]:

No observations found

Any remaining protocol required tissues, which have been examined, have no visible lesions

Gross Pathology - The following Tissues were Not Examined:

None

Histopathology Animal Details:

No animal details found

Histopathology Observations [Correlation]:

GANGLION, DORSAL ROOT, LUMBAR : Infiltration, mononuclear cell; minimal
GANGLION, DORSAL ROOT, LUMBAR : Degeneration; moderate, nerve fiber
GANGLION, DORSAL ROOT, LUMBAR : Aggregate; minimal, glial cell
NERVE, SCIATIC : Degeneration; minimal, nerve fiber
OVARY : Atrophy; minimal
SPINAL CORD : (Comment) Lumbar, thoracic, dorsal funiculus
SPINAL CORD : Degeneration; mild, nerve fiber

Histopathology - The following Tissues were Within Normal Limits:

BRAIN; GANGLION, DORSAL ROOT, CERVICAL; GANGLION, DORSAL ROOT, THORACIC; GLAND, ADRENAL; GLAND, MAMMARY; HEART; KIDNEY; LARGE INTESTINE, COLON; LIVER; LUNG; LYMPH NODE, ILIAC; PANCREAS; SPLEEN

Histopathology - The following Tissues were Not Examined:

None

DRAFT, NOT FOR SUBMISSION

Appendix 18

Appendix 3

Individual Macroscopic and Microscopic Pathology: Terminal Day 91±2

32025165

Animal: 4513	Group: 4	Sex: Female
Species: Rat	Strain: Sprague Dawley	
	Dose: 1.49x10^12 vg	
	Removal Reason: Terminal Euthanasia	
	Study Day (Week) of Death: 92 (14)	
		Gross Status: Complete
		Histo Status: Complete

Gross Pathology Animal Details:

No animal details found

Gross Pathology Observations [Correlation]:

No observations found

Any remaining protocol required tissues, which have been examined, have no visible lesions

Gross Pathology - The following Tissues were Not Examined:

None

Histopathology Animal Details:

No animal details found

Histopathology Observations [Correlation]:

GANGLION, DORSAL ROOT, LUMBAR : Infiltration, mononuclear cell; mild
GANGLION, DORSAL ROOT, LUMBAR : Degeneration; mild, nerve fiber
NERVE, SCIATIC : Degeneration; minimal, nerve fiber
SPINAL CORD : (Comment) Lumbar, thoracic, dorsal funiculus
SPINAL CORD : Degeneration; mild, nerve fiber

Histopathology - The following Tissues were Within Normal Limits:

BRAIN; GANGLION, DORSAL ROOT, CERVICAL; GANGLION, DORSAL ROOT, THORACIC; GLAND, ADRENAL; GLAND, MAMMARY; HEART; KIDNEY; LARGE INTESTINE, COLON; LIVER; LUNG; LYMPH NODE, ILIAC; OVARY; PANCREAS; SPLEEN

Histopathology - The following Tissues were Not Examined:

None

DRAFT, NOT FOR SUBMISSION

Appendix 18

Appendix 3

Individual Macroscopic and Microscopic Pathology: Terminal Day 91±2

32025165

Animal: 4514	Group: 4	Sex: Female
Species: Rat	Strain: Sprague Dawley	
	Dose: 1.49x10^12 vg	
	Removal Reason: Terminal Euthanasia	
	Study Day (Week) of Death: 93 (14)	
		Gross Status: Complete
		Histo Status: Complete

Gross Pathology Animal Details:

No animal details found

Gross Pathology Observations [Correlation]:

No observations found

Any remaining protocol required tissues, which have been examined, have no visible lesions

Gross Pathology - The following Tissues were Not Examined:

None

Histopathology Animal Details:

No animal details found

Histopathology Observations [Correlation]:

GANGLION, DORSAL ROOT, LUMBAR : Infiltration, mononuclear cell; mild
GANGLION, DORSAL ROOT, LUMBAR : Degeneration; mild, nerve fiber
GANGLION, DORSAL ROOT, LUMBAR : Degeneration/necrosis; neuronal, minimal
GANGLION, DORSAL ROOT, LUMBAR : Aggregate; mild, glial cell
NERVE, SCIATIC : Degeneration; minimal, nerve fiber
SPINAL CORD : (Comment) Lumbar, thoracic, dorsal funiculus, nerve root
SPINAL CORD : Degeneration; mild, nerve fiber

Histopathology - The following Tissues were Within Normal Limits:

BRAIN; GANGLION, DORSAL ROOT, CERVICAL; GANGLION, DORSAL ROOT, THORACIC; GLAND, ADRENAL; GLAND, MAMMARY; HEART; KIDNEY; LARGE INTESTINE, COLON; LIVER; LUNG; LYMPH NODE, ILIAC; OVARY; PANCREAS; SPLEEN

Histopathology - The following Tissues were Not Examined:

None

DRAFT, NOT FOR SUBMISSION

Appendix 18

Appendix 3

Individual Macroscopic and Microscopic Pathology: Terminal Day 91±2

32025165

Animal: 4515	Group: 4	Sex: Female
Species: Rat	Strain: Sprague Dawley	
	Dose: 1.49x10^12 vg	
	Removal Reason: Terminal Euthanasia	
	Study Day (Week) of Death: 93 (14)	
		Gross Status: Complete
		Histo Status: Complete

Gross Pathology Animal Details:

No animal details found

Gross Pathology Observations [Correlation]:

No observations found

Any remaining protocol required tissues, which have been examined, have no visible lesions

Gross Pathology - The following Tissues were Not Examined:

None

Histopathology Animal Details:

No animal details found

Histopathology Observations [Correlation]:

GANGLION, DORSAL ROOT, LUMBAR : (Note) One Of A Pair Available For Evaluation.
GANGLION, DORSAL ROOT, LUMBAR : Degeneration; minimal, nerve fiber

Histopathology - The following Tissues were Within Normal Limits:

BRAIN; GANGLION, DORSAL ROOT, CERVICAL; GANGLION, DORSAL ROOT, THORACIC; GLAND, ADRENAL; GLAND, MAMMARY; HEART; KIDNEY; LARGE INTESTINE, COLON; LIVER; LUNG; LYMPH NODE, ILIAC; NERVE, SCIATIC; OVARY; PANCREAS; SPINAL CORD; SPLEEN

Histopathology - The following Tissues were Not Examined:

None

DRAFT, NOT FOR SUBMISSION

Appendix 18

Appendix 4

32025165

Image Document of AAV Associated
Histopathological Changes

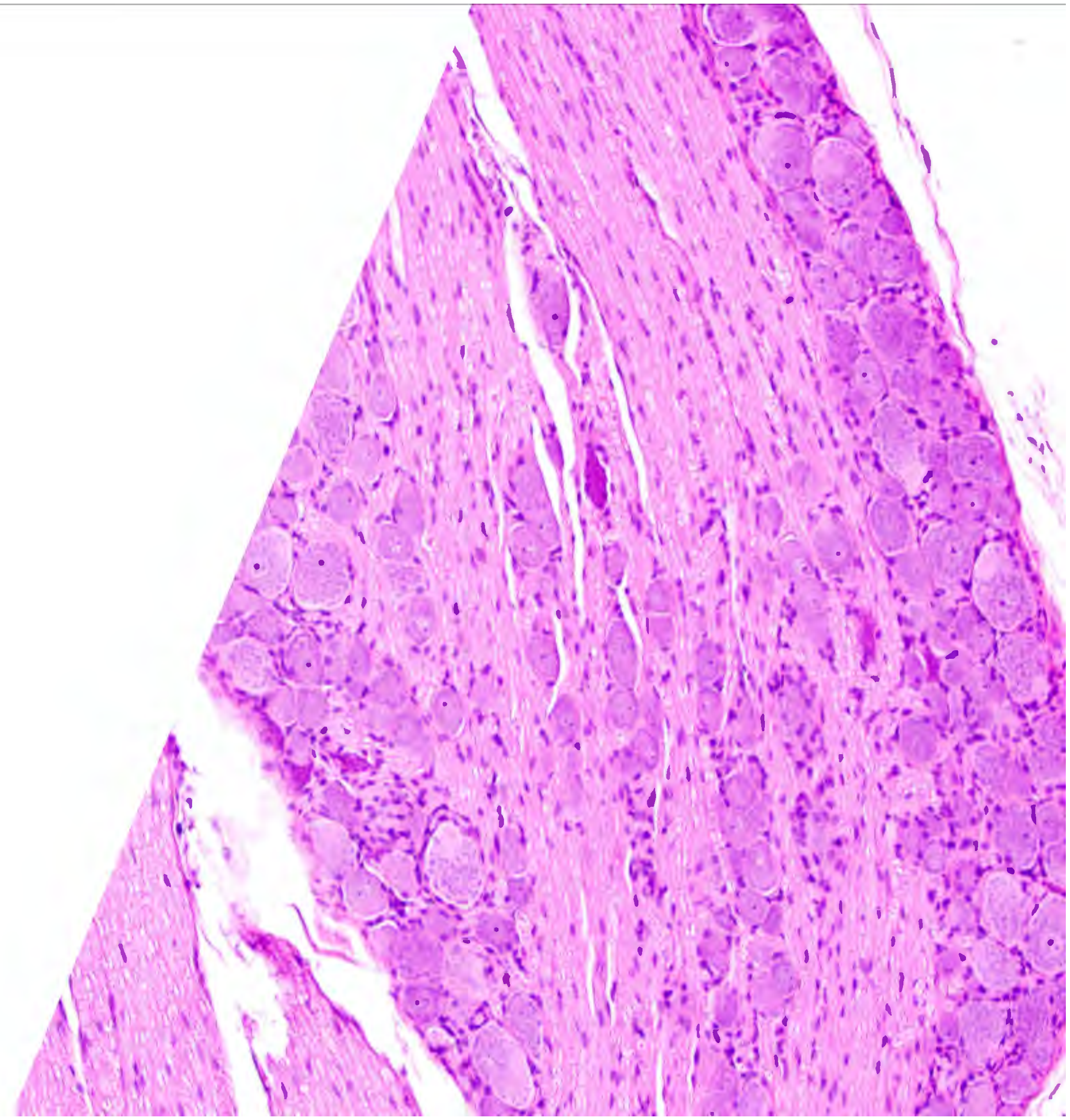

DRAFT, NOT FOR SUBMISSION

Appendix 18
Appendix 4

Images from Control Animals

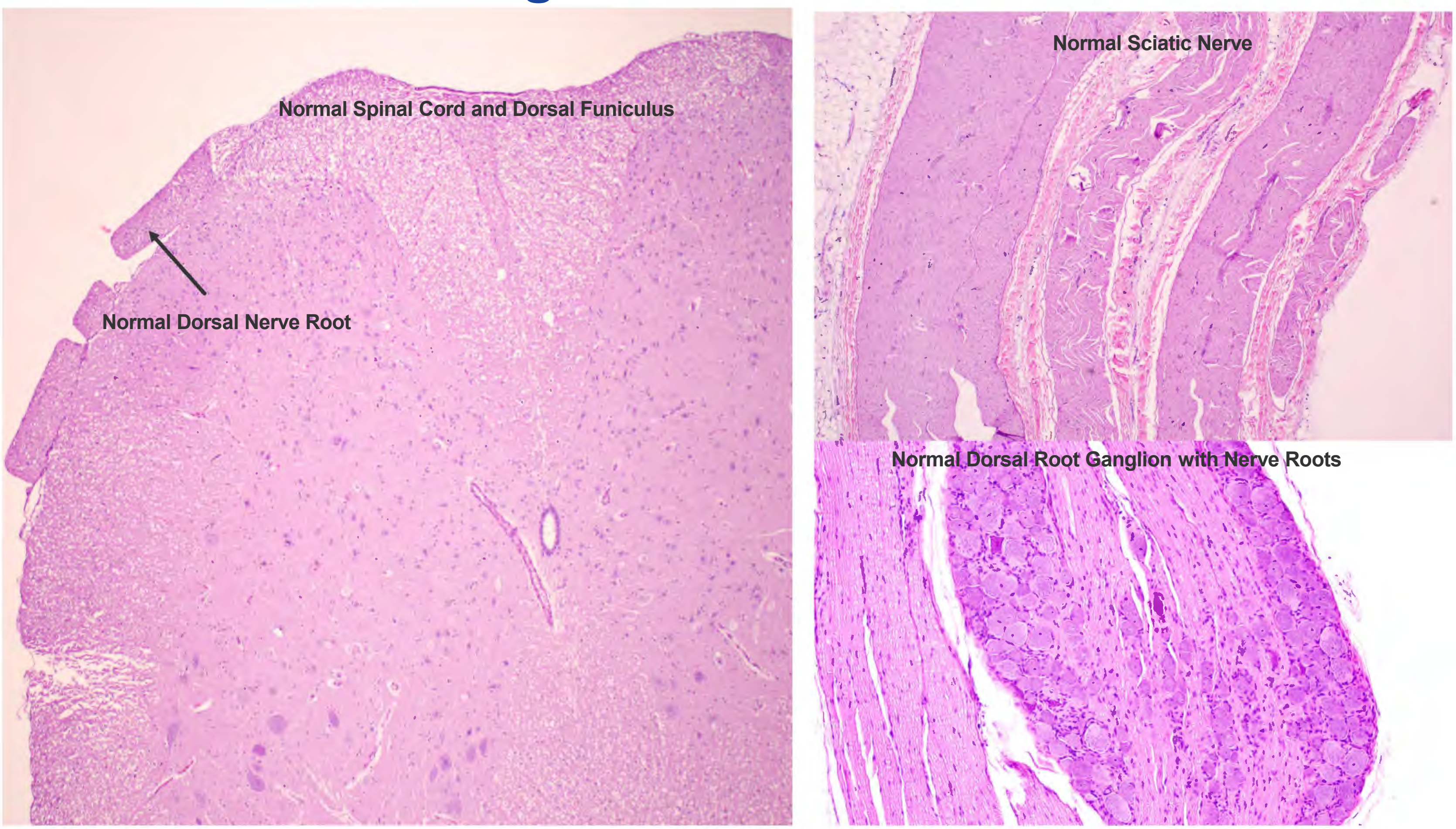

DRAFT, NOT FOR SUBMISSION

Appendix 18
Appendix 4

Animal No. 4002 male 1.49 x 10^{12} vg

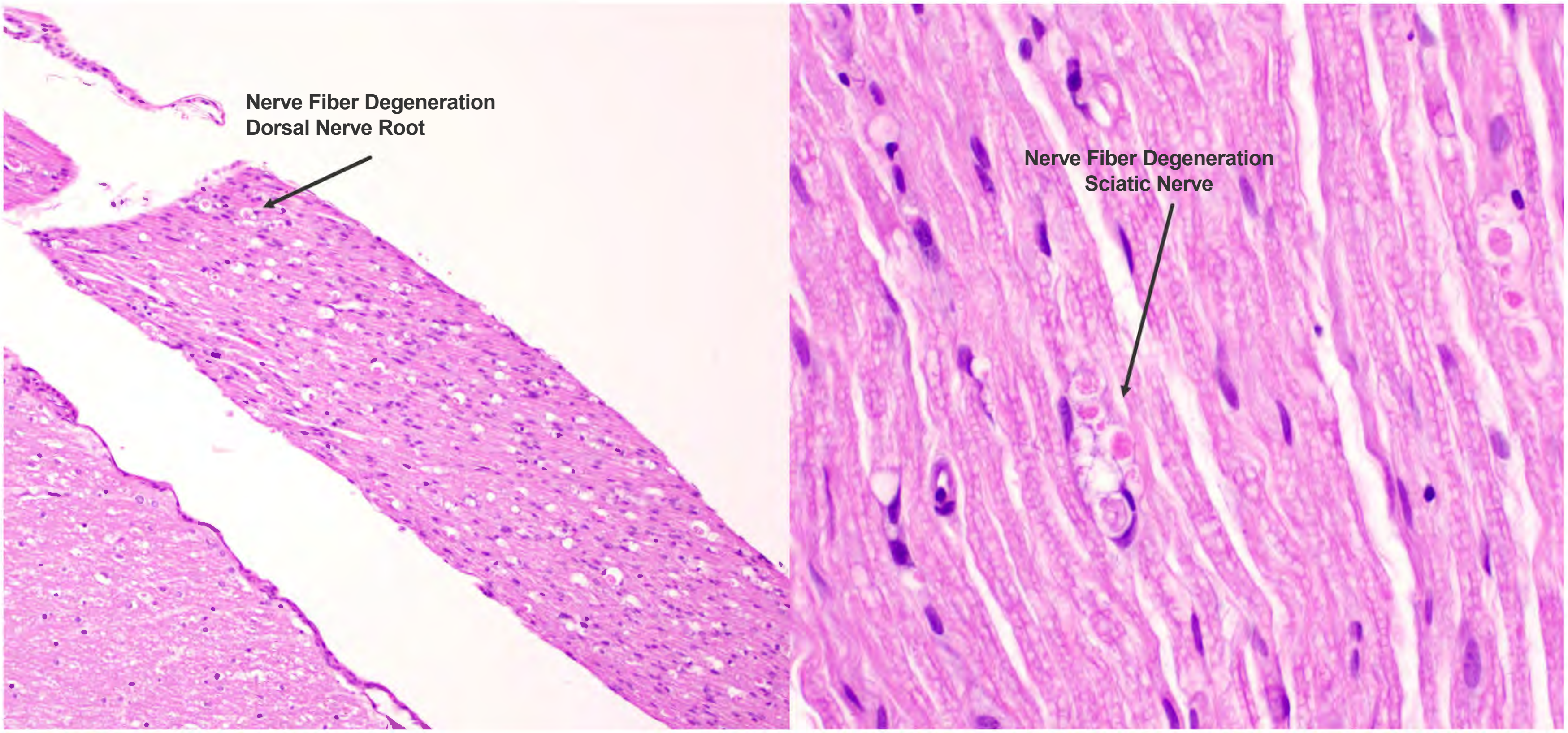

DRAFT, NOT FOR SUBMISSION

Appendix 18

Appendix 4

Animal No. 4002 male 1.49 x 10^{12} vg

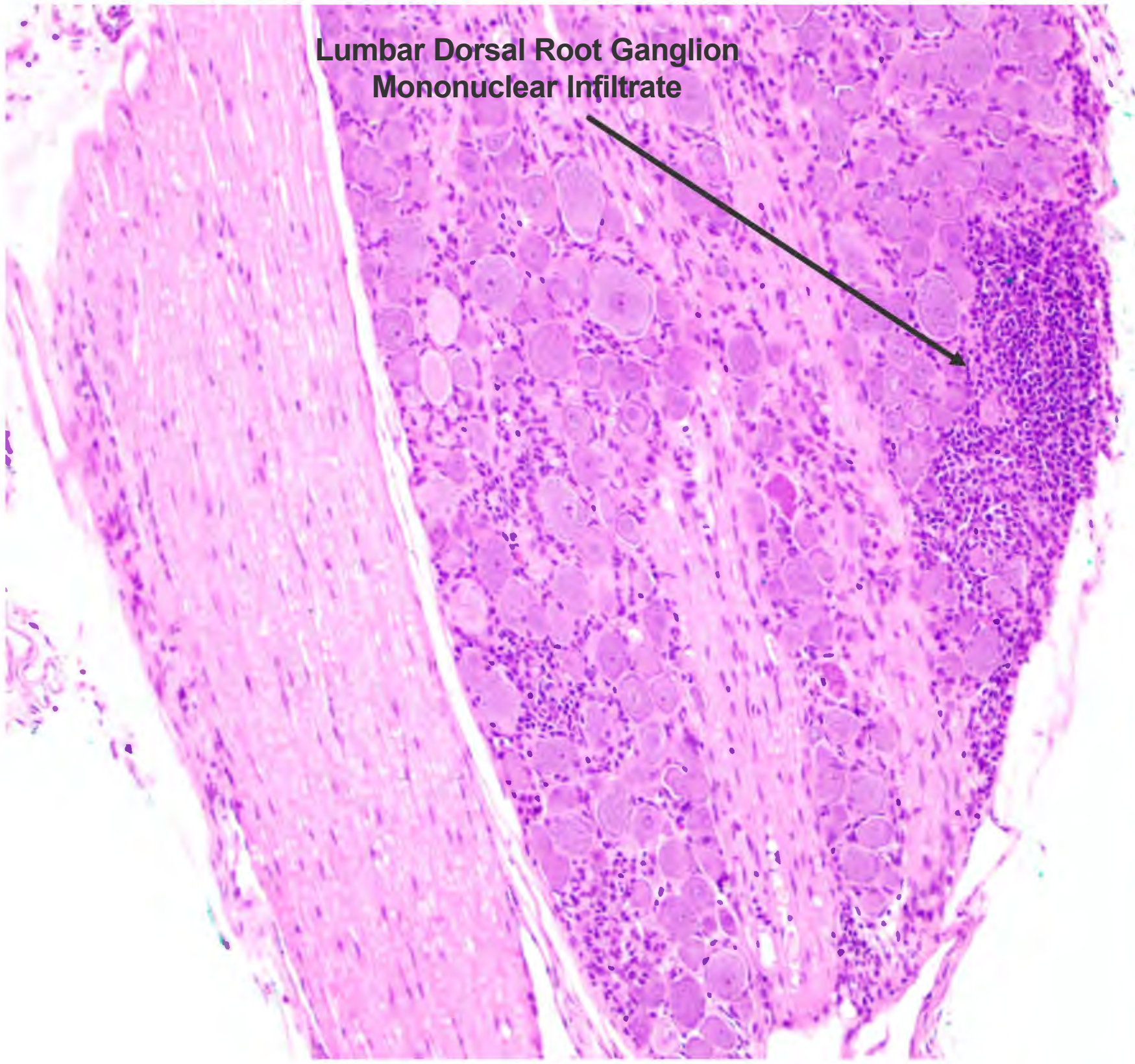

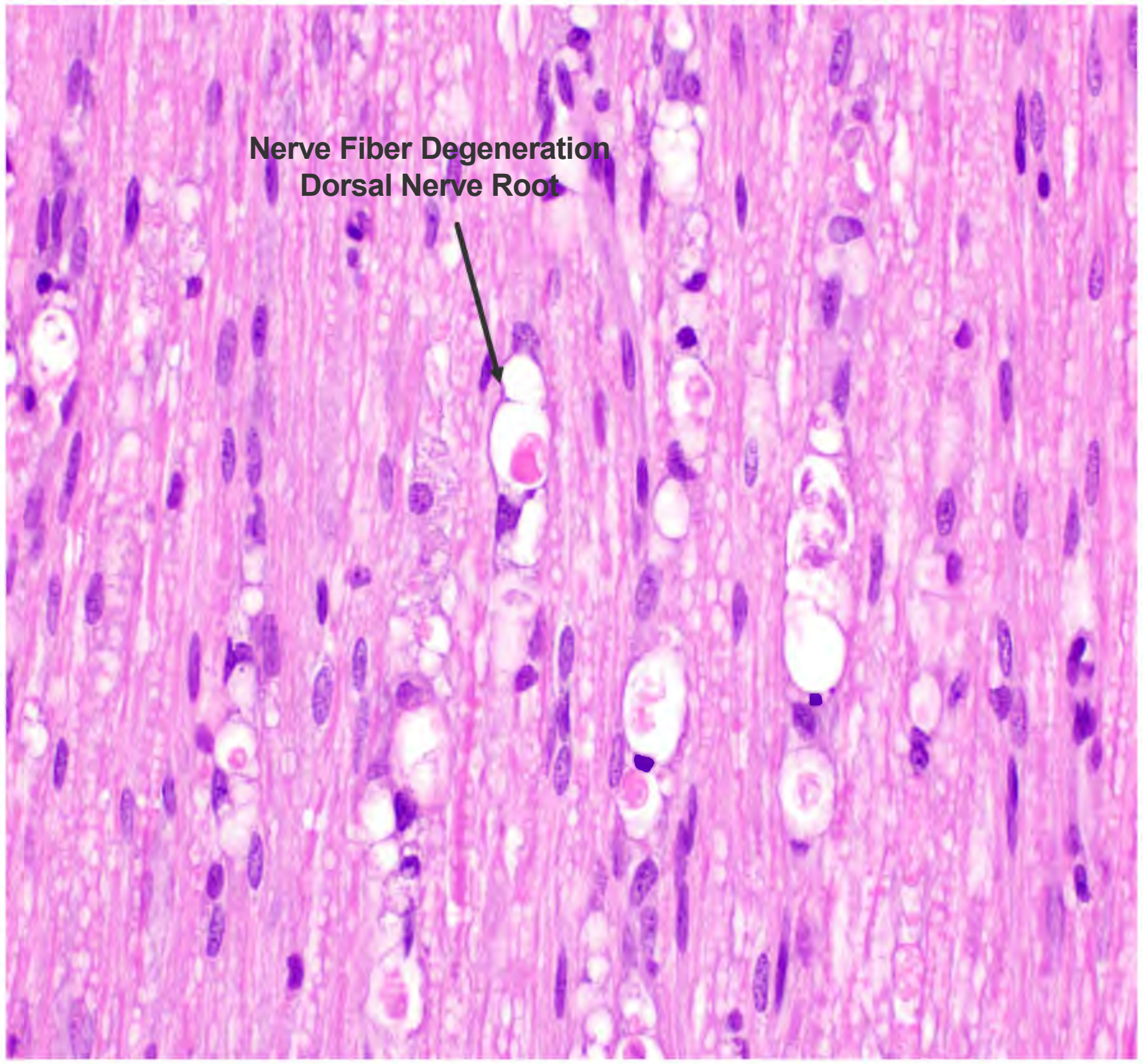

DRAFT, NOT FOR SUBMISSION

Appendix 18

Appendix 4

Animal No. 4003 male 1.49 x 10^{12} vg

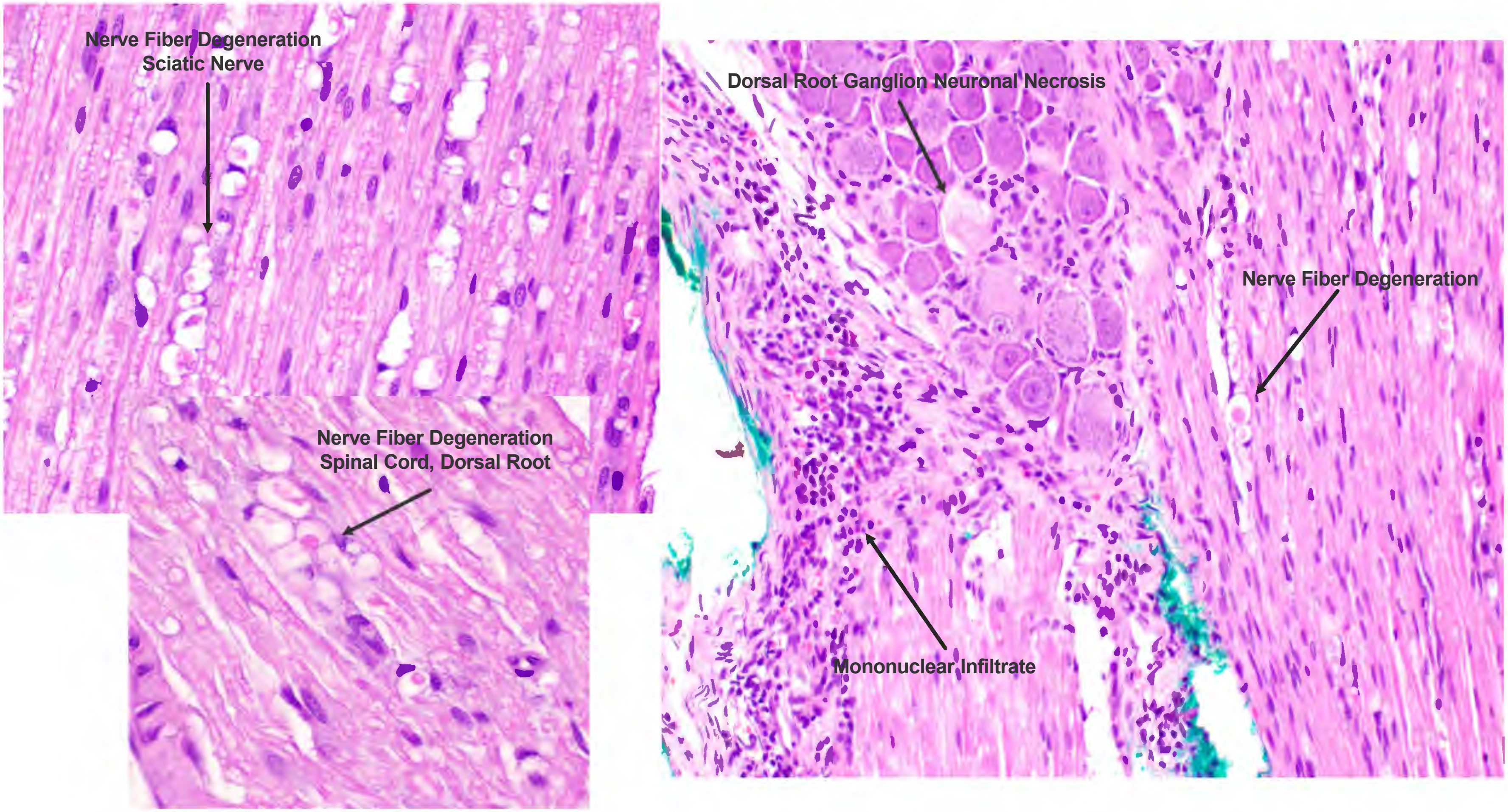

DRAFT, NOT FOR SUBMISSION

Appendix 18

Appendix 4

Animal No. 4501 female 1.49 x 10^{12} vg

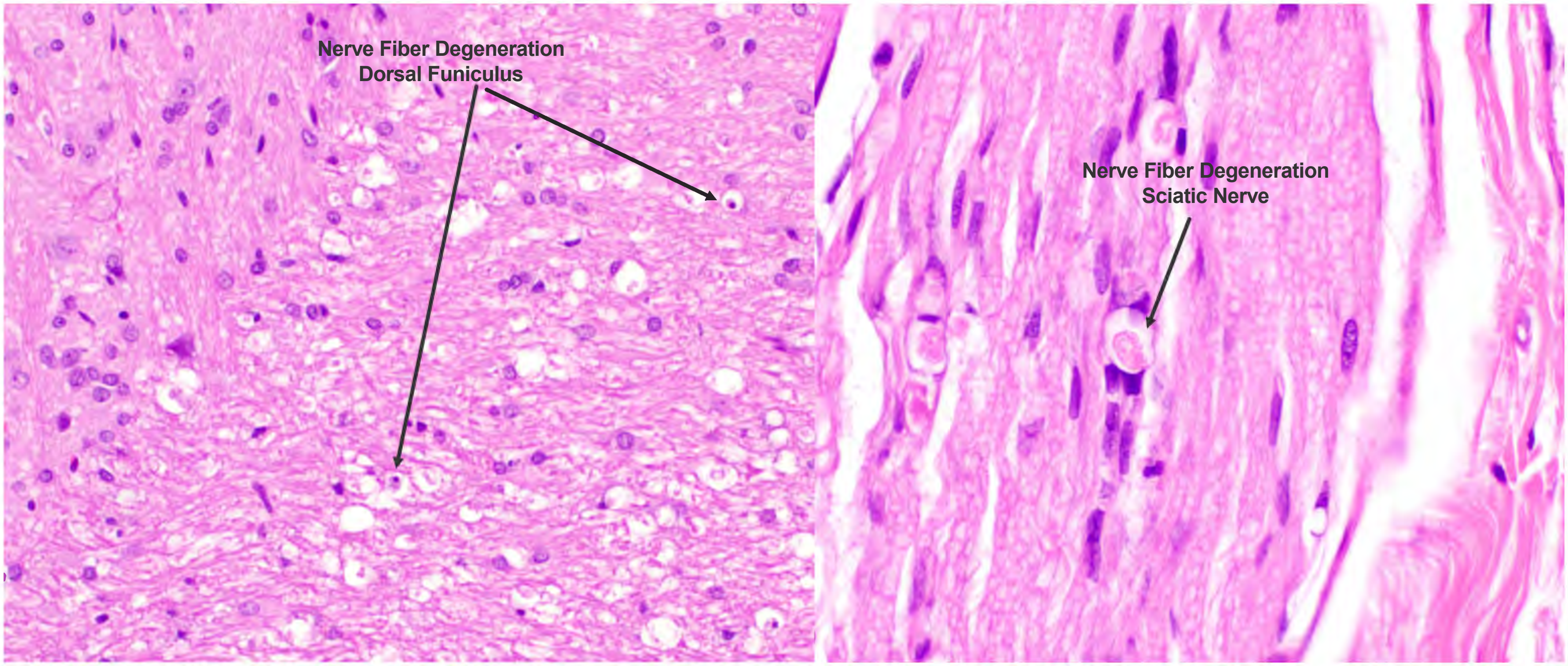

DRAFT, NOT FOR SUBMISSION

Appendix 18

Appendix 4

Animal No. 4501 female 1.49 x 10^{12} vg

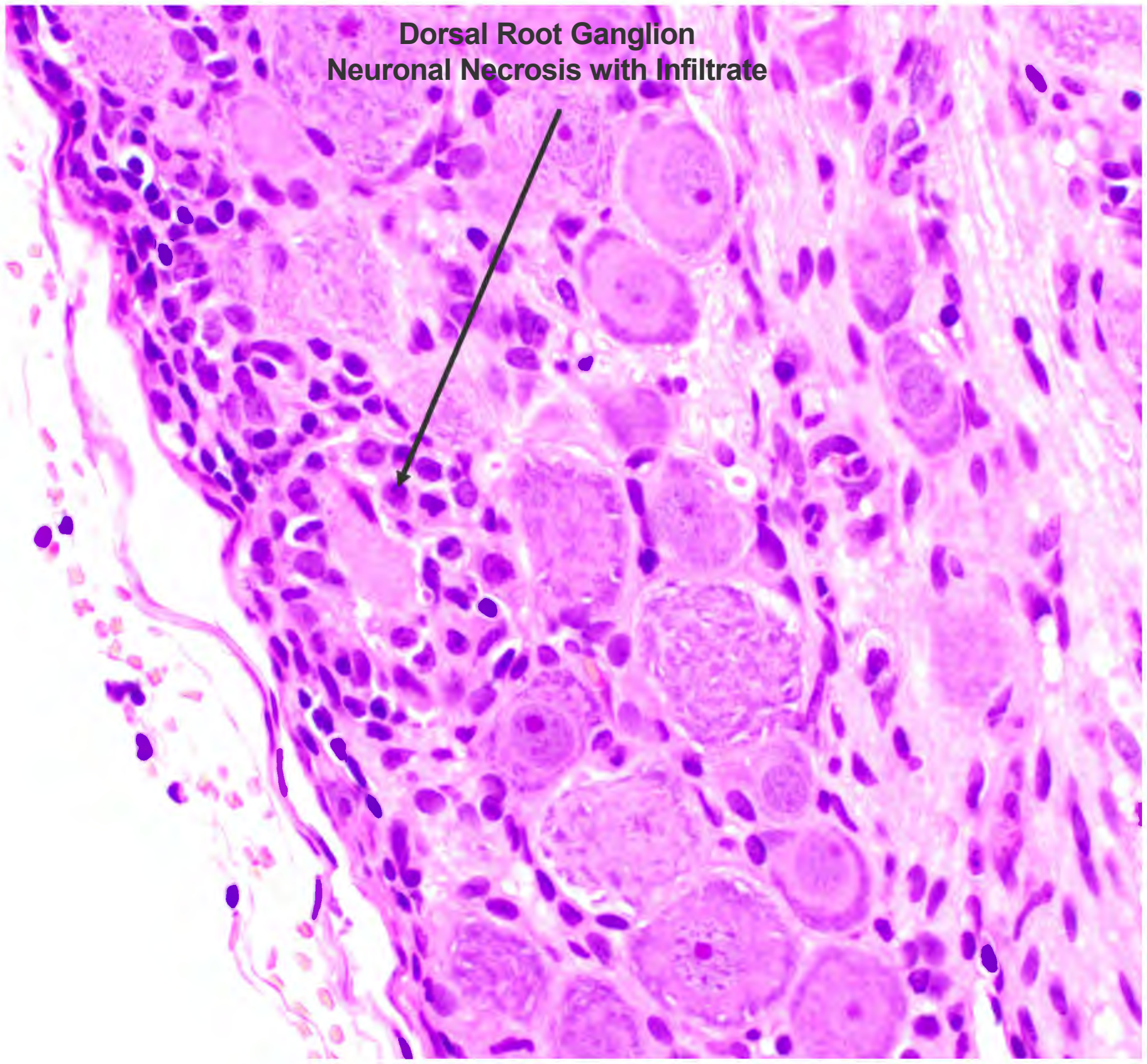

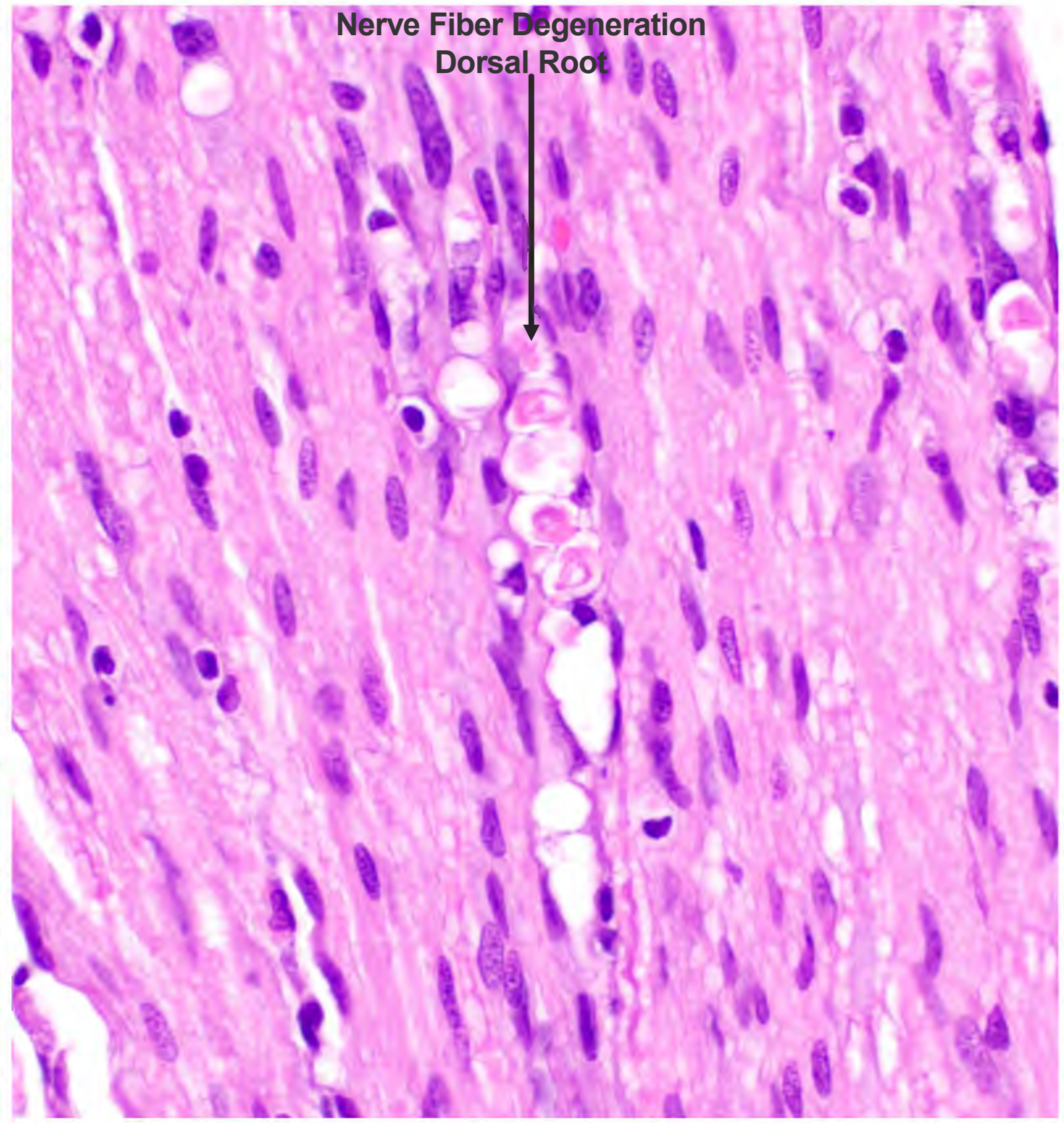

Appendix 18

Appendix 4

Animal No. 4502 female 1.49 x 10^{12} vg

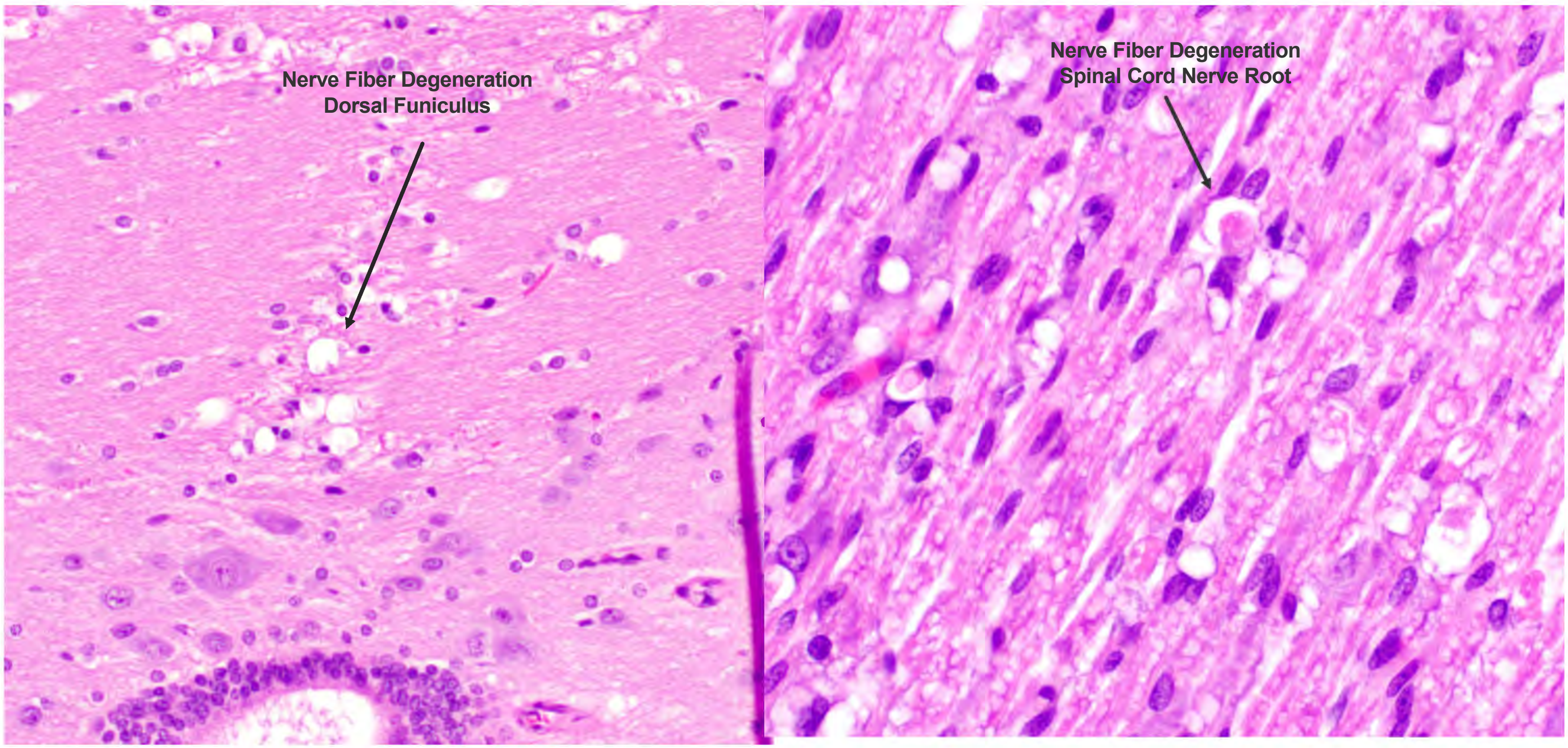

DRAFT, NOT FOR SUBMISSION

Appendix 18

Appendix 4

Animal No. 4502 female 1.49 x 10^{12} vg

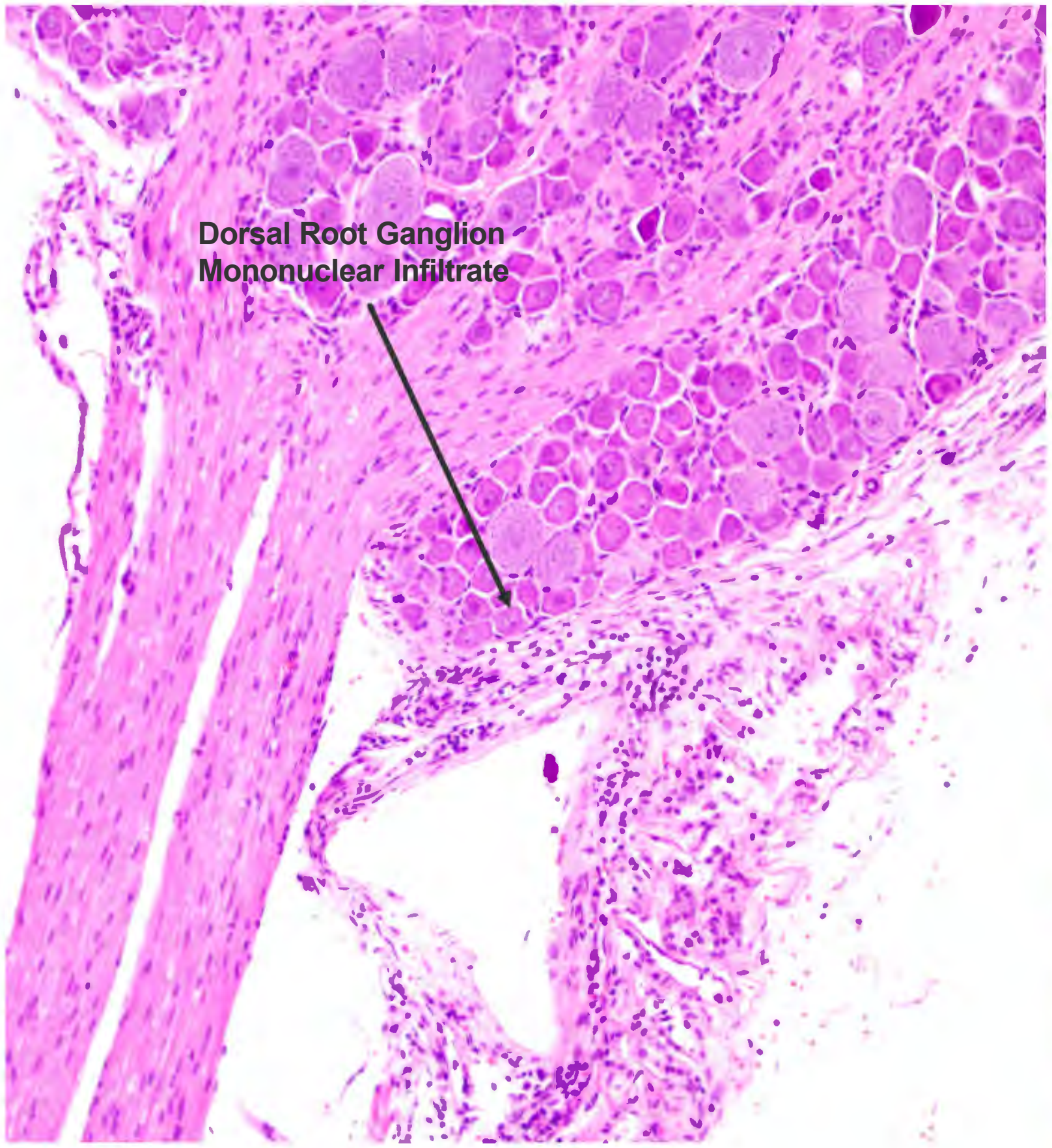

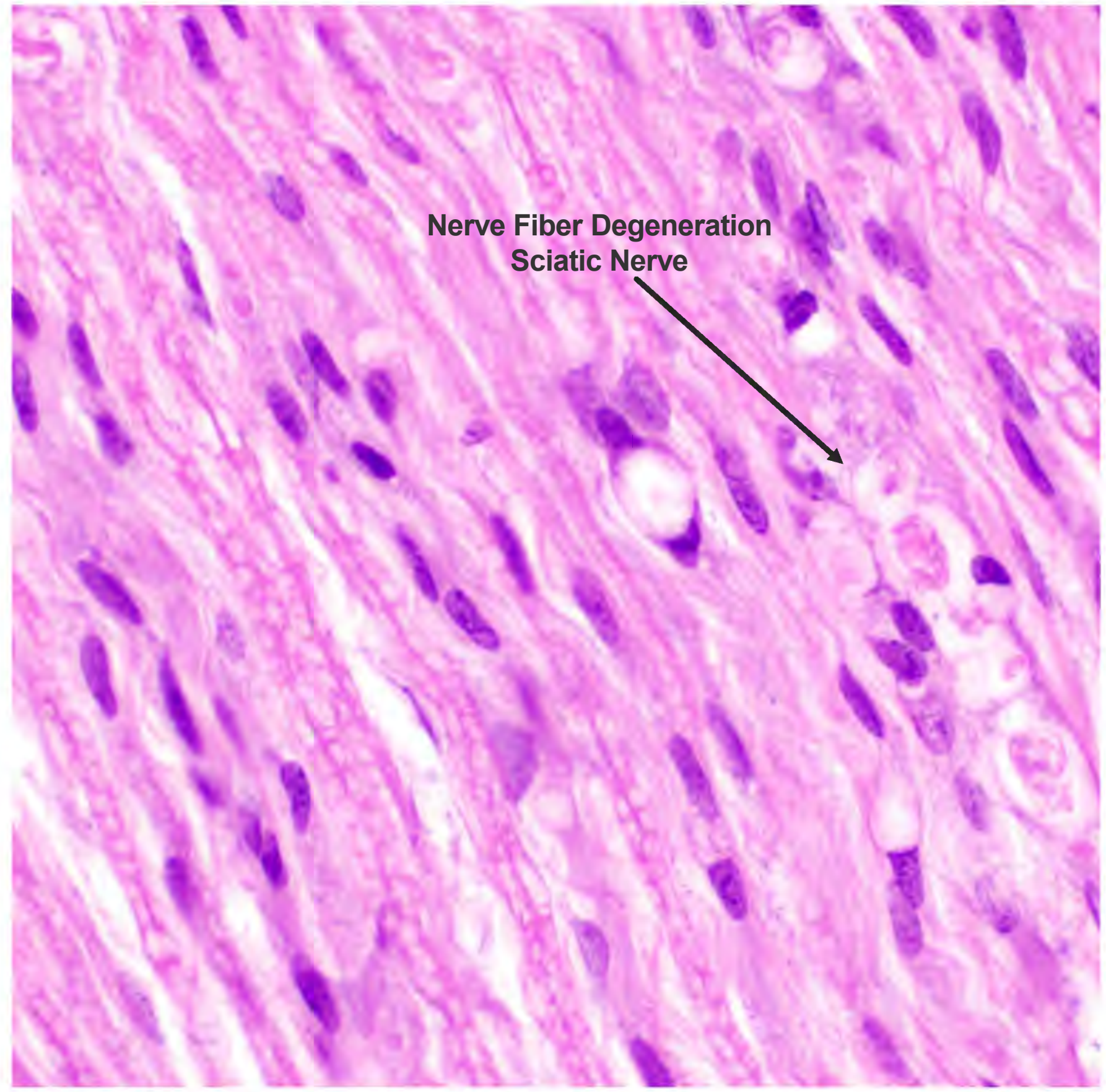

DRAFT, NOT FOR SUBMISSION

Appendix 18

Appendix 4

Animal No. 4503 female 1.49 x 10^{12} vg

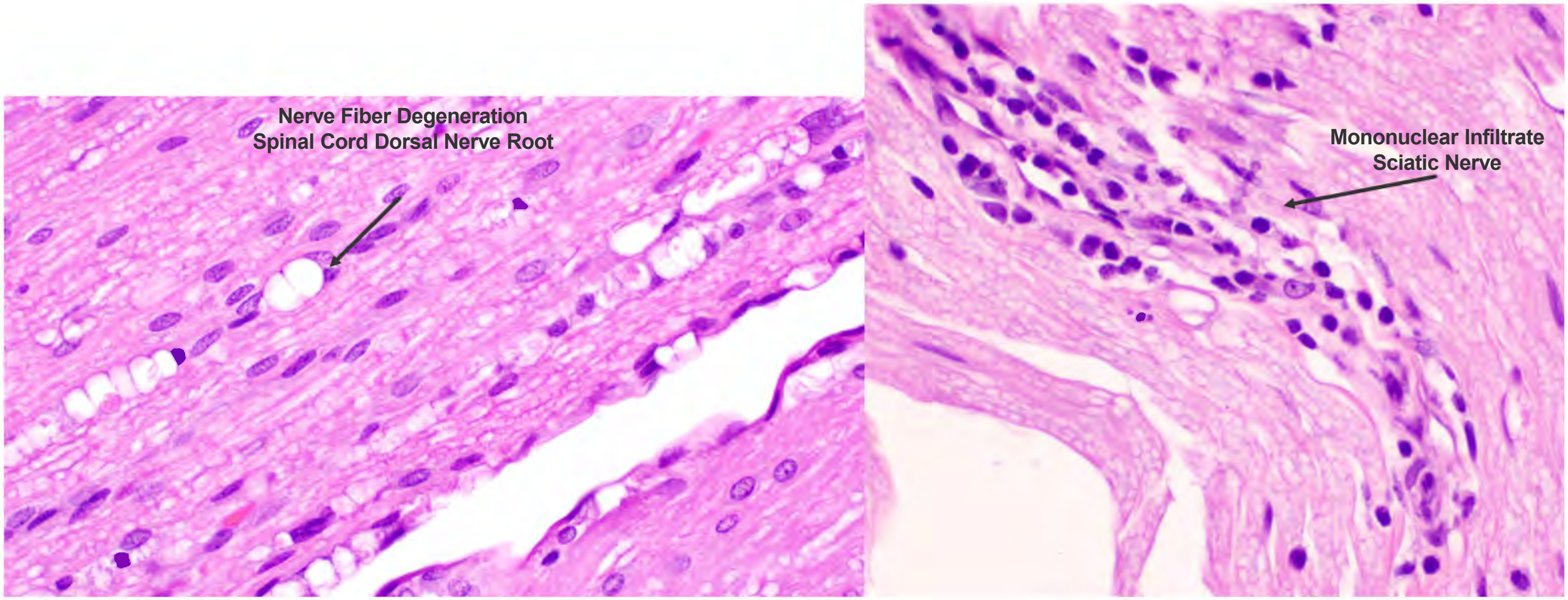

Appendix 18
Appendix 4

Animal No. 4503 female 1.49 x 10^{12} vg

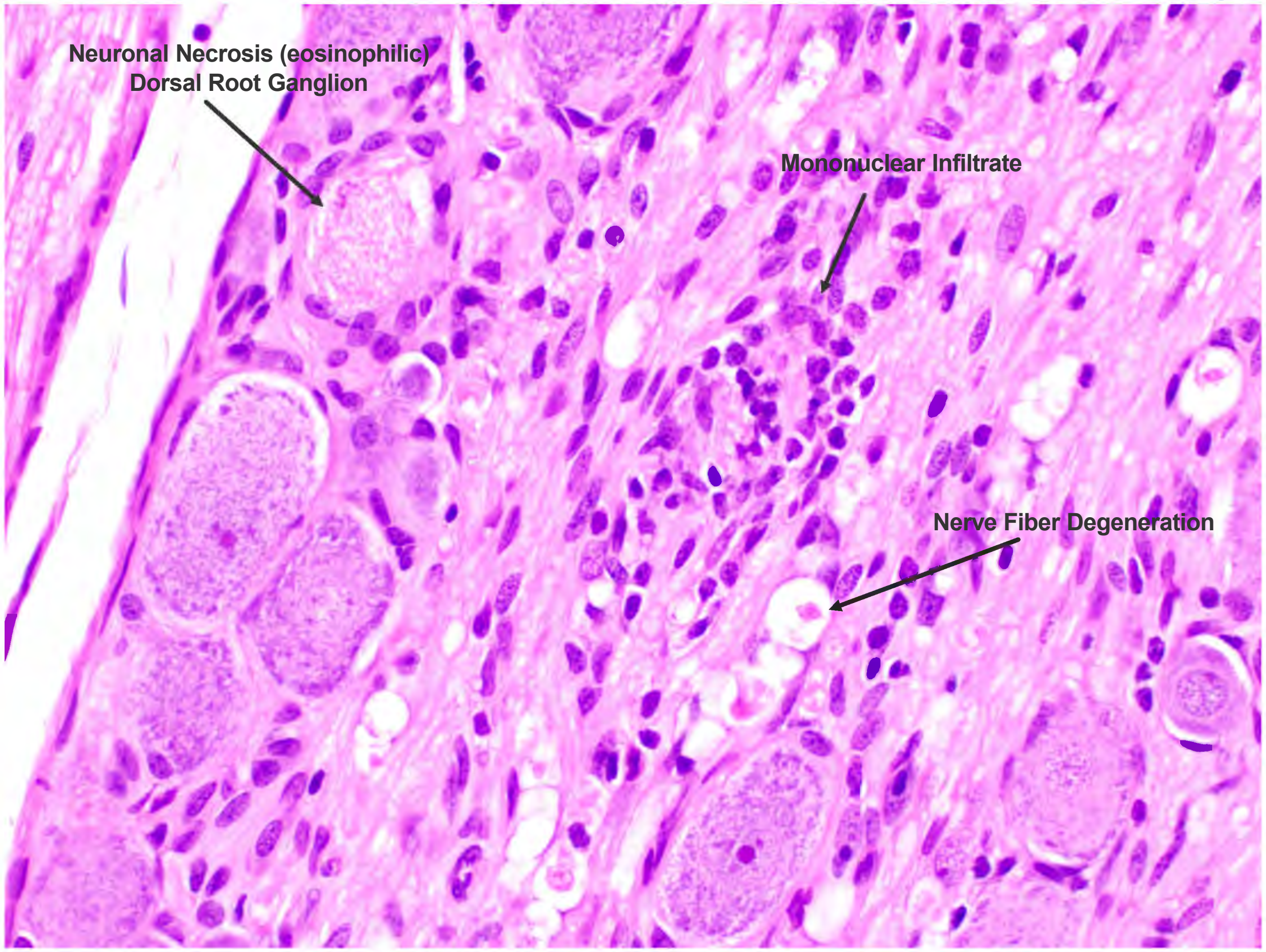

DRAFT, NOT FOR SUBMISSION

Appendix 18

Appendix 4

Animal No. 4504 female 1.49 x 10^{12} vg

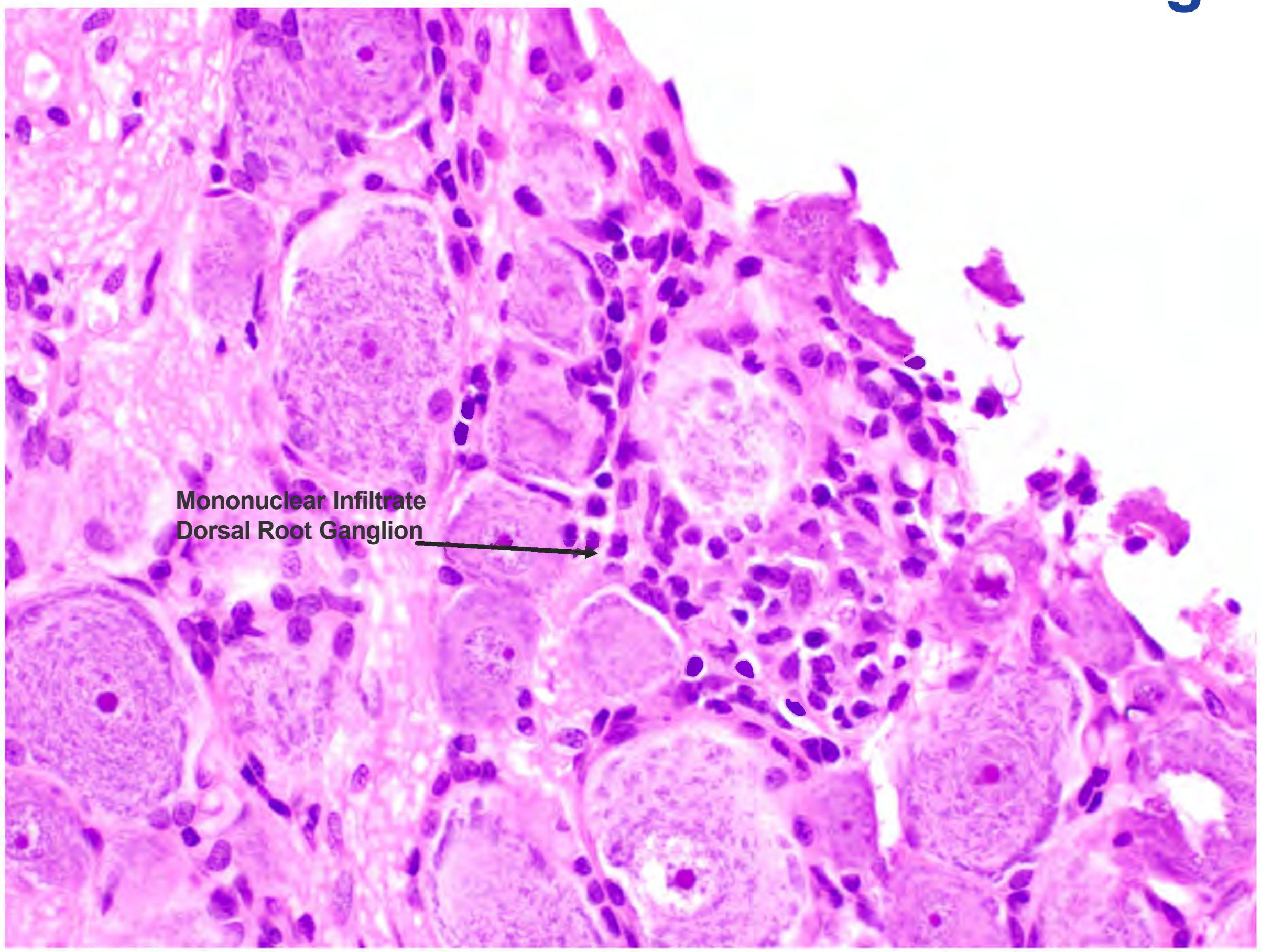

Appendix 18
Appendix 4

Animal No. 4505 female 1.49 x 10^{12} vg

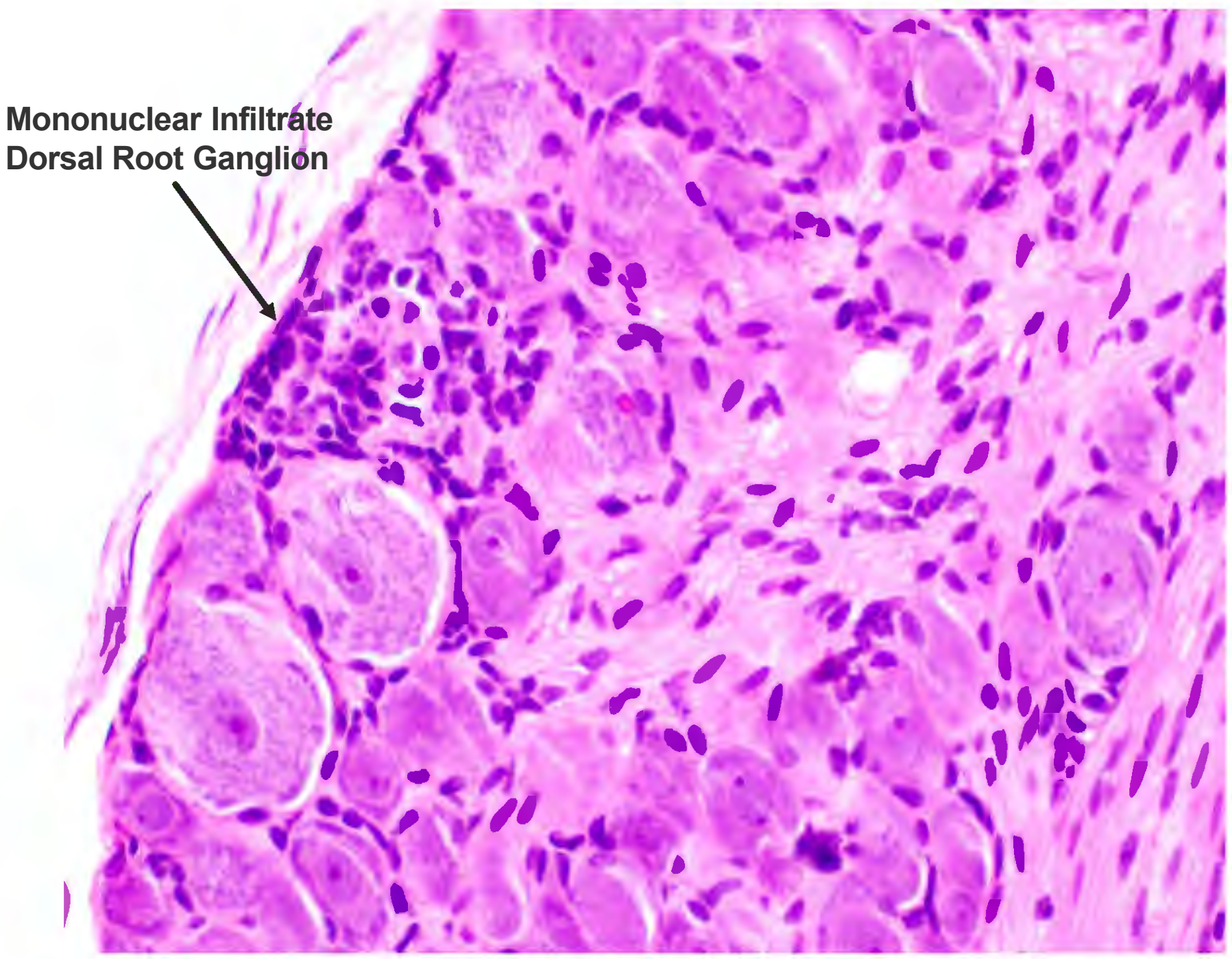

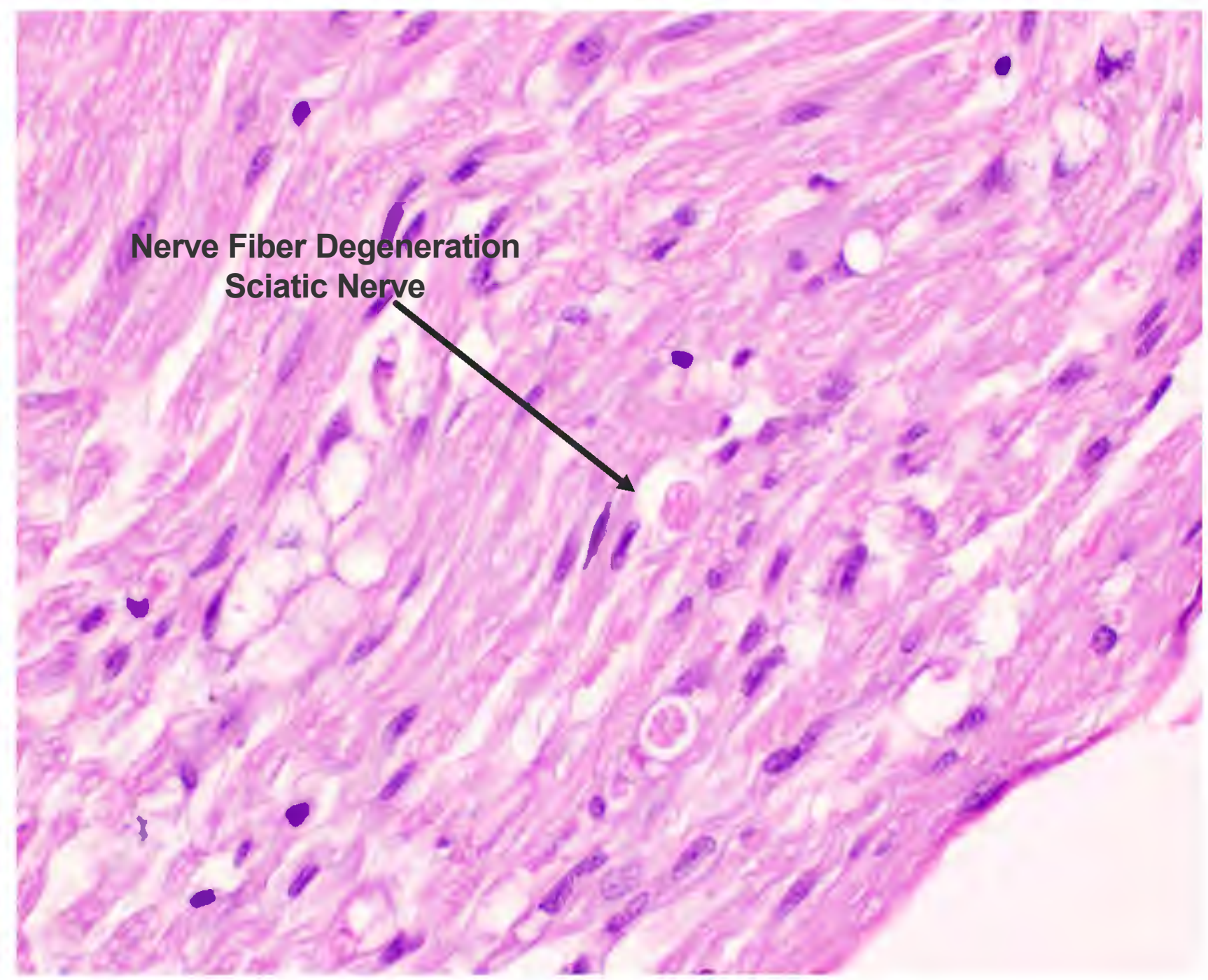

DRAFT, NOT FOR SUBMISSION
