# Supplementary material for: AAV-mediated gene therapy demonstrates phenotypic rescue in a mouse model of Cockayne syndrome
Source: J Clin Invest. 2026 Jul 30;136(18):e196689. doi: 10.1172/JCI196689 (PMC13574149; doi:10.1172/JCI196689)

Fig. 1A

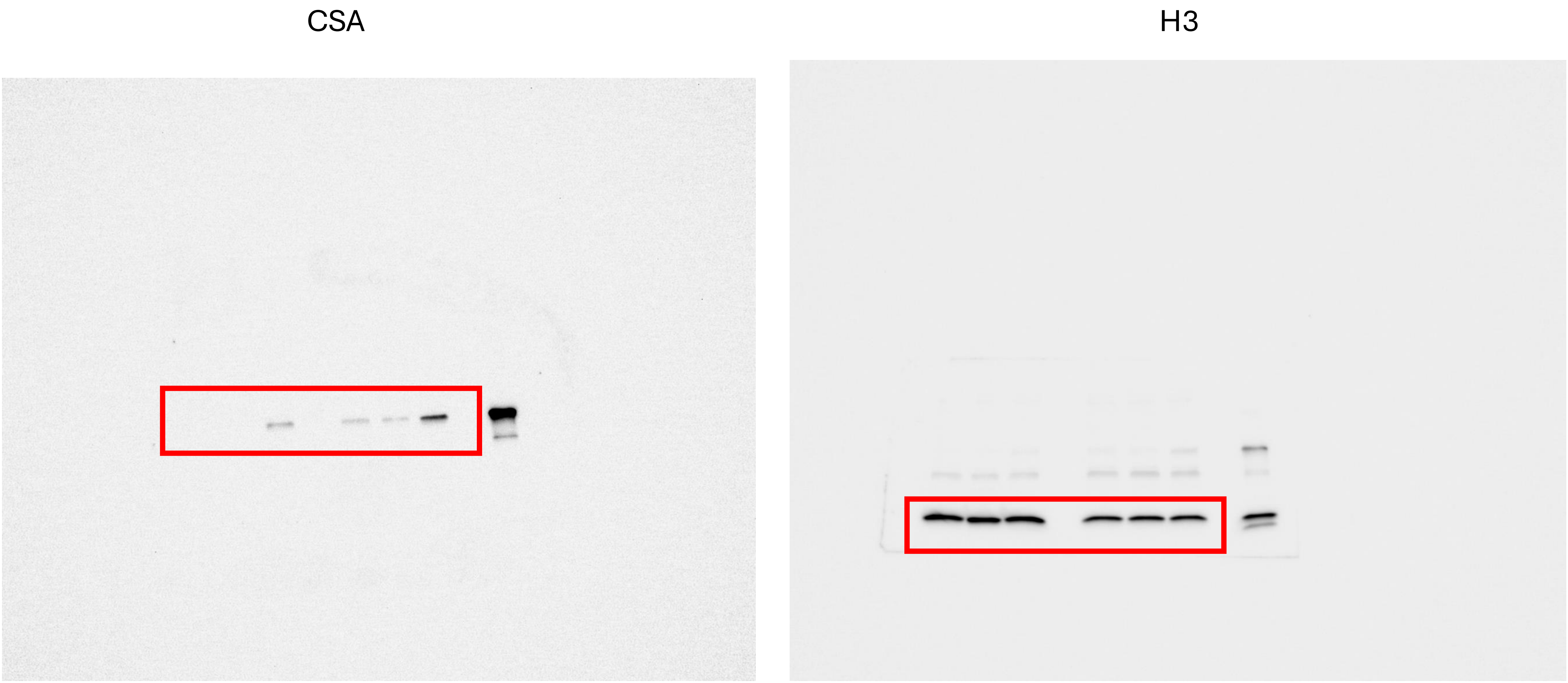

Fig. 1B

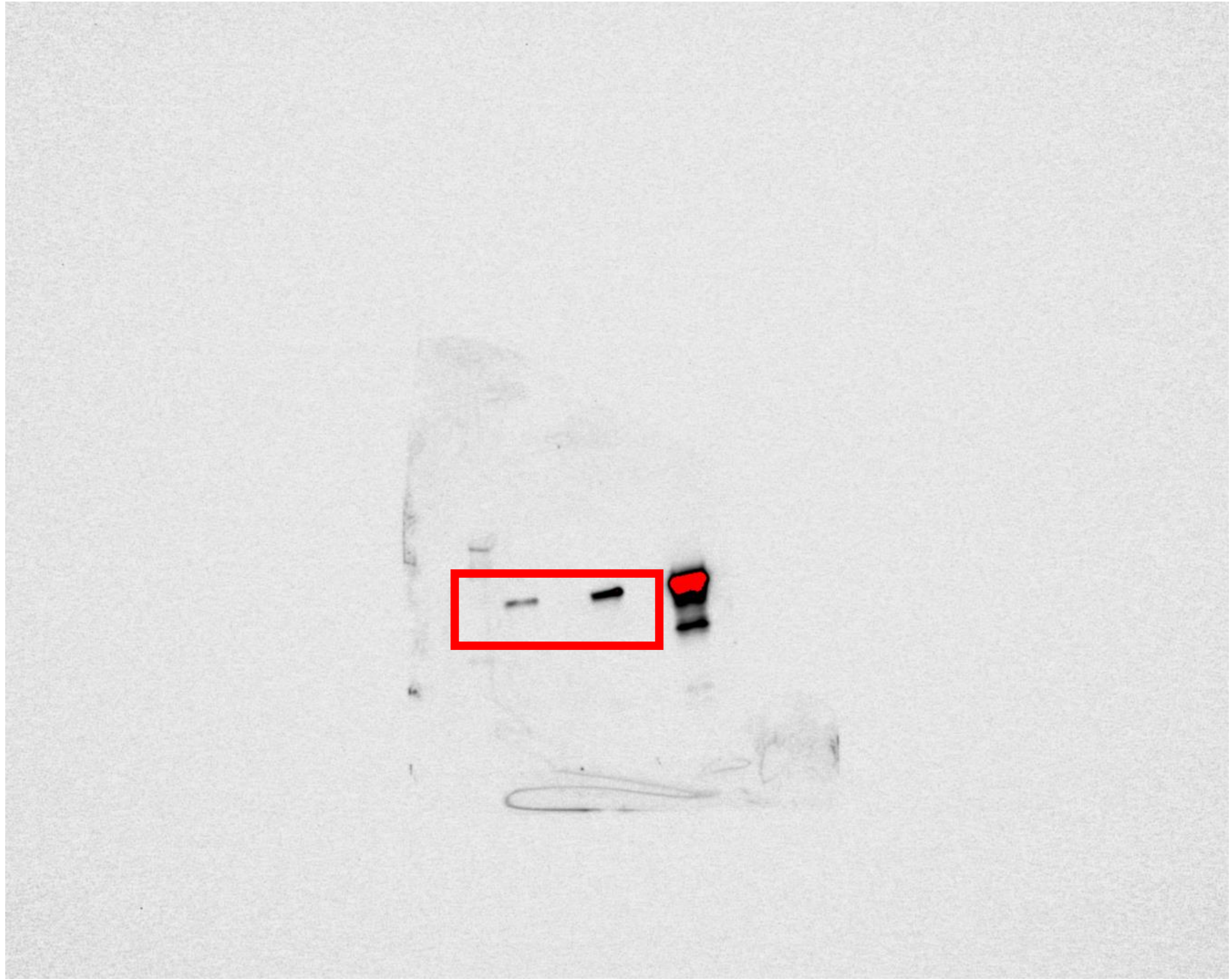

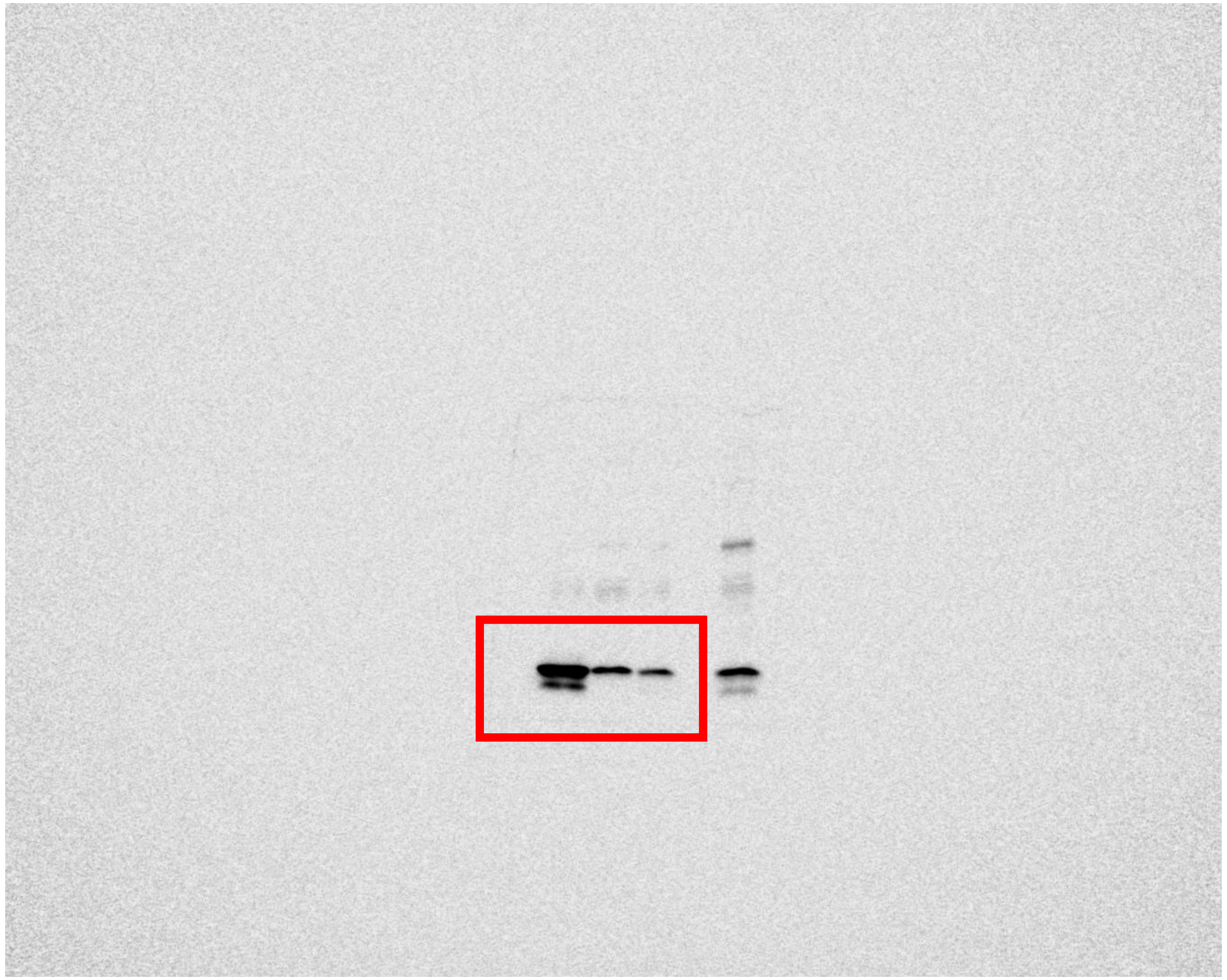

Fig.4A

Brain - CSA

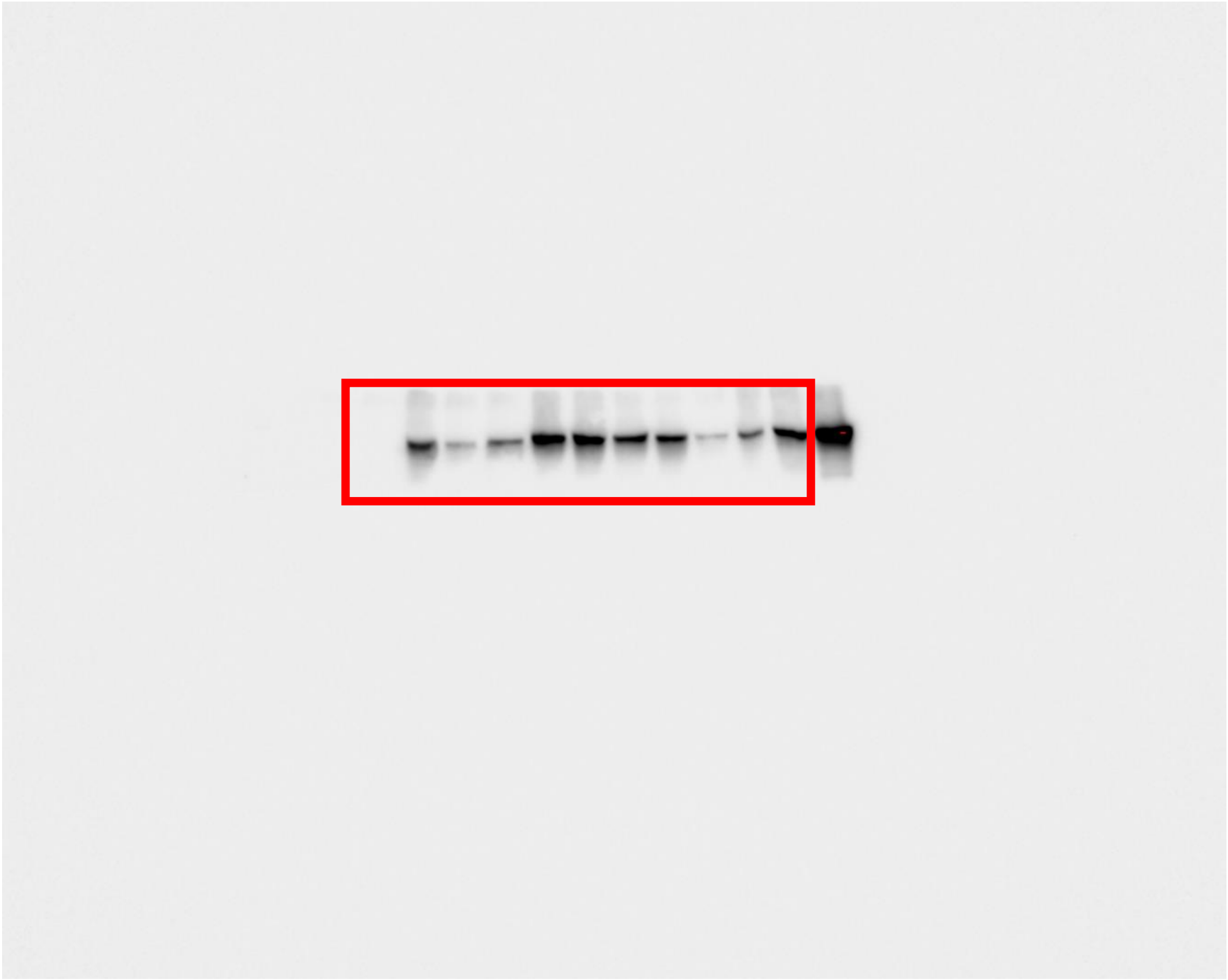

Brain - tubulin

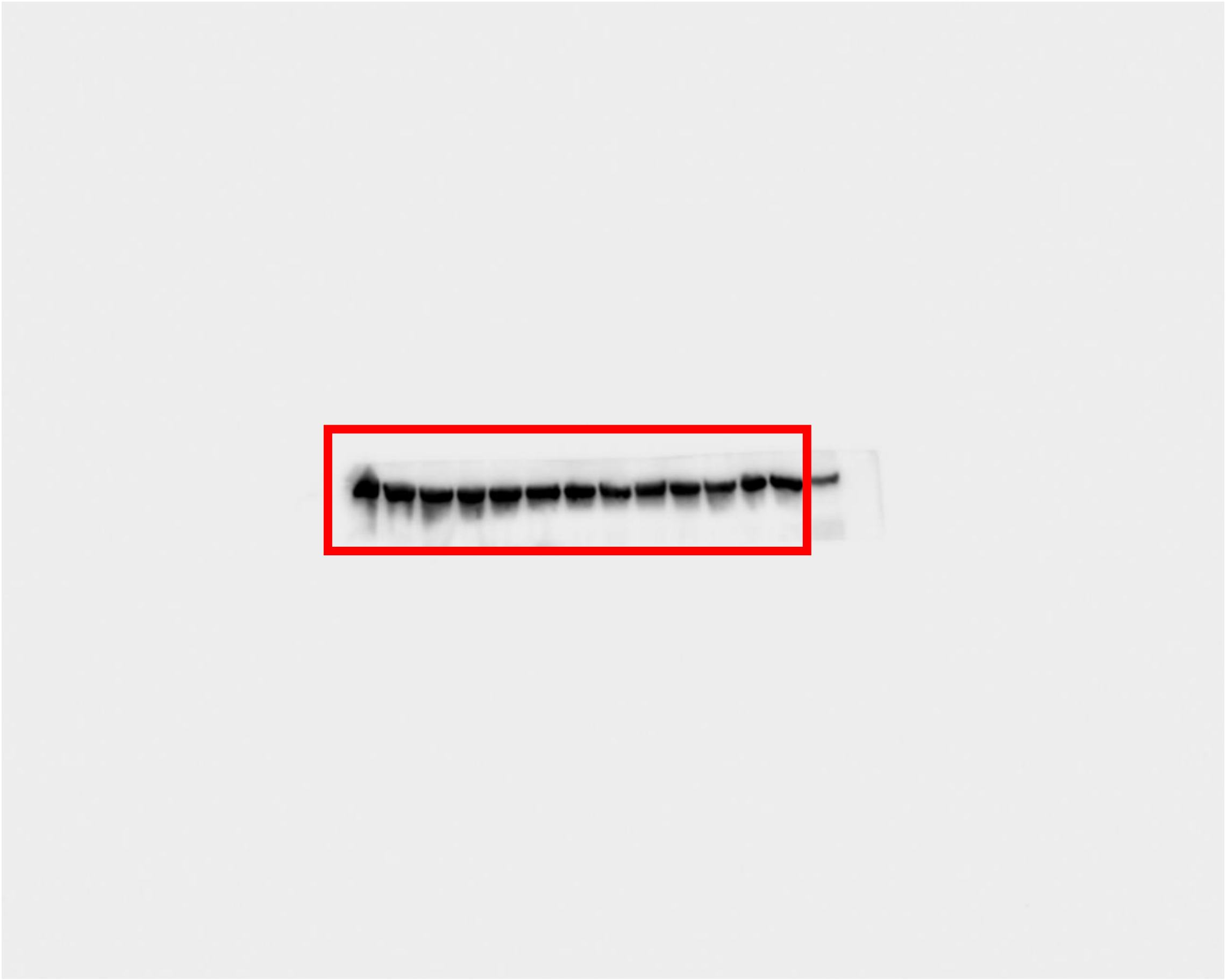

Fig. 4B

Heart - CSA

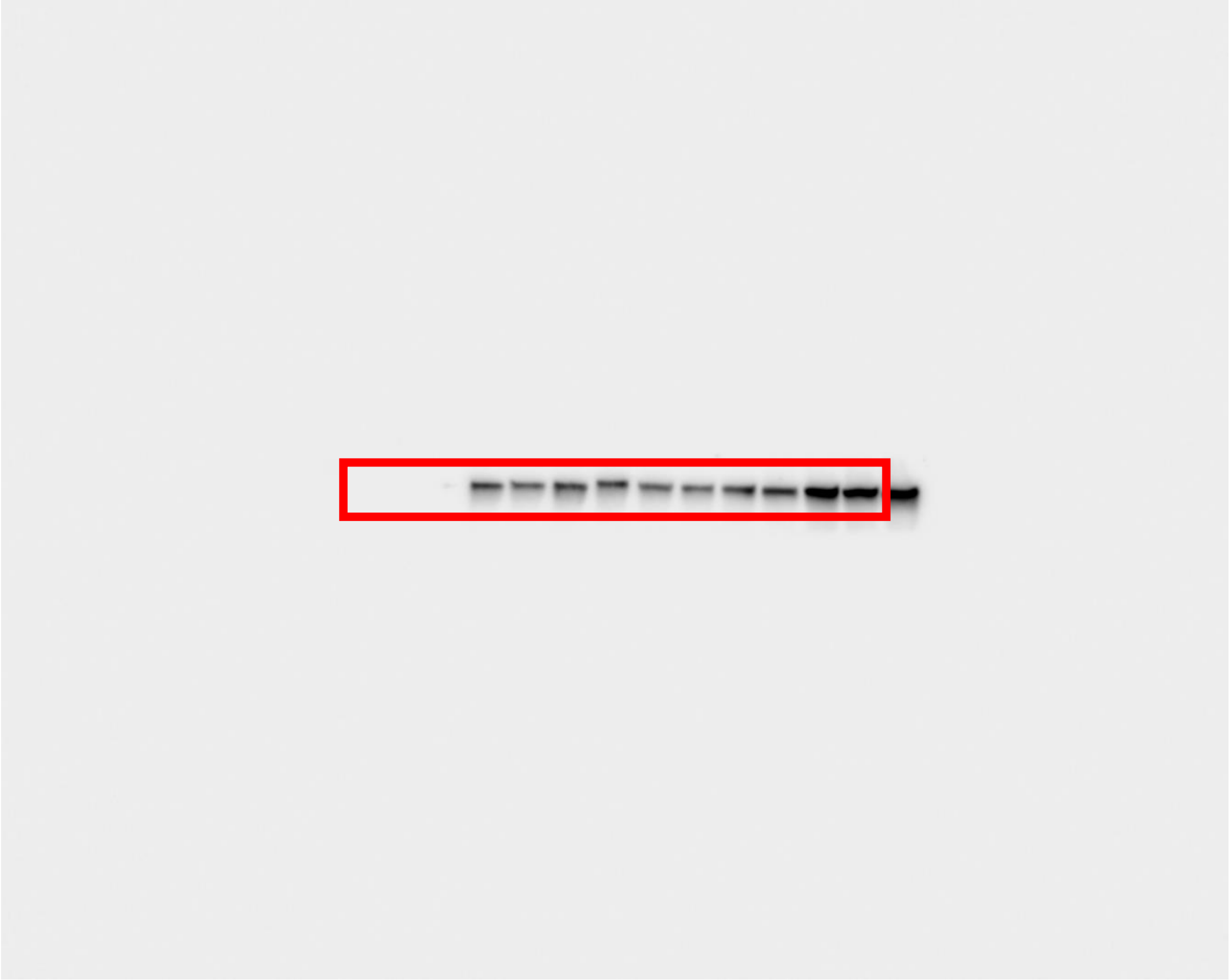

Heart - Vinculin

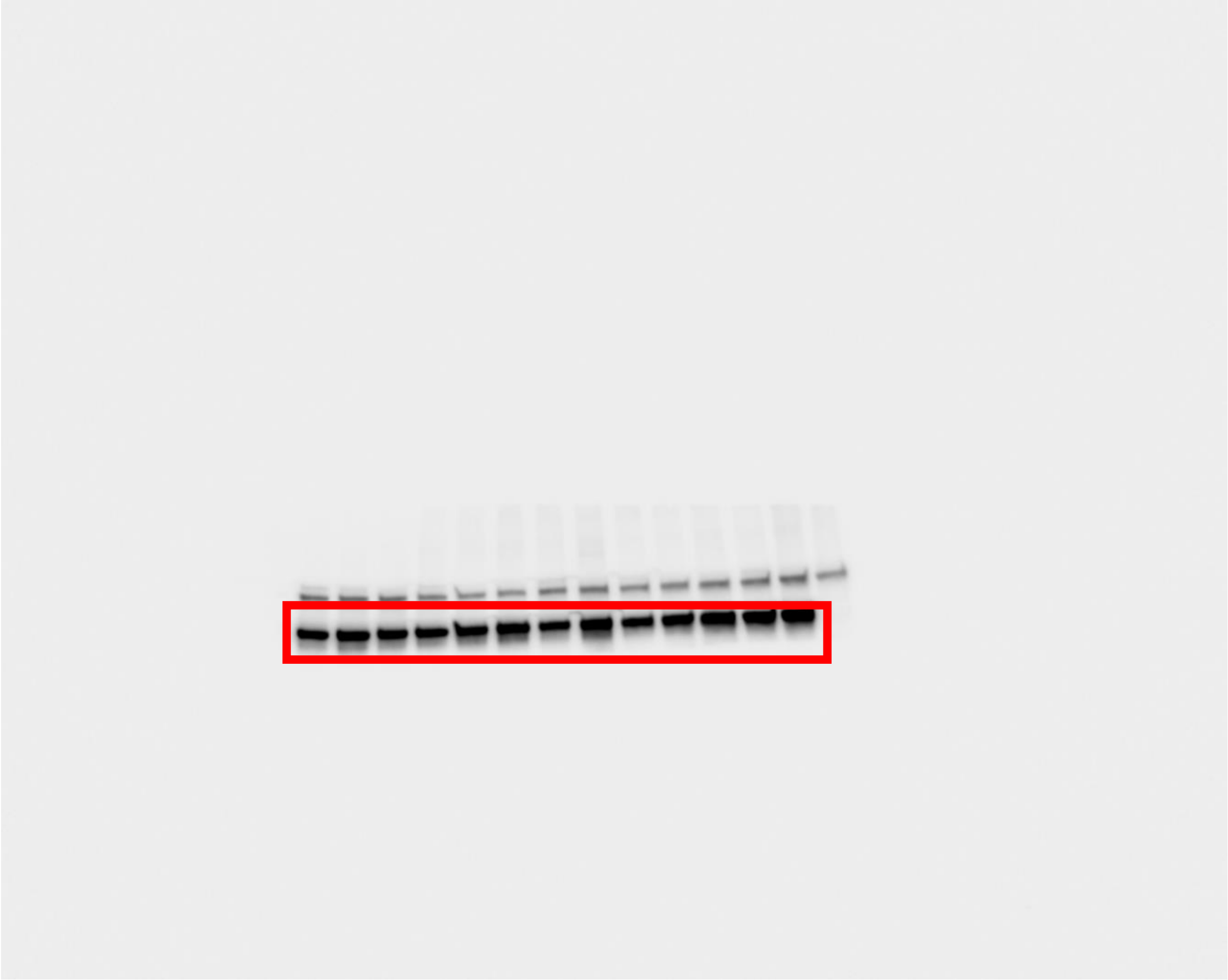

Sup. Fig. 1

CSA

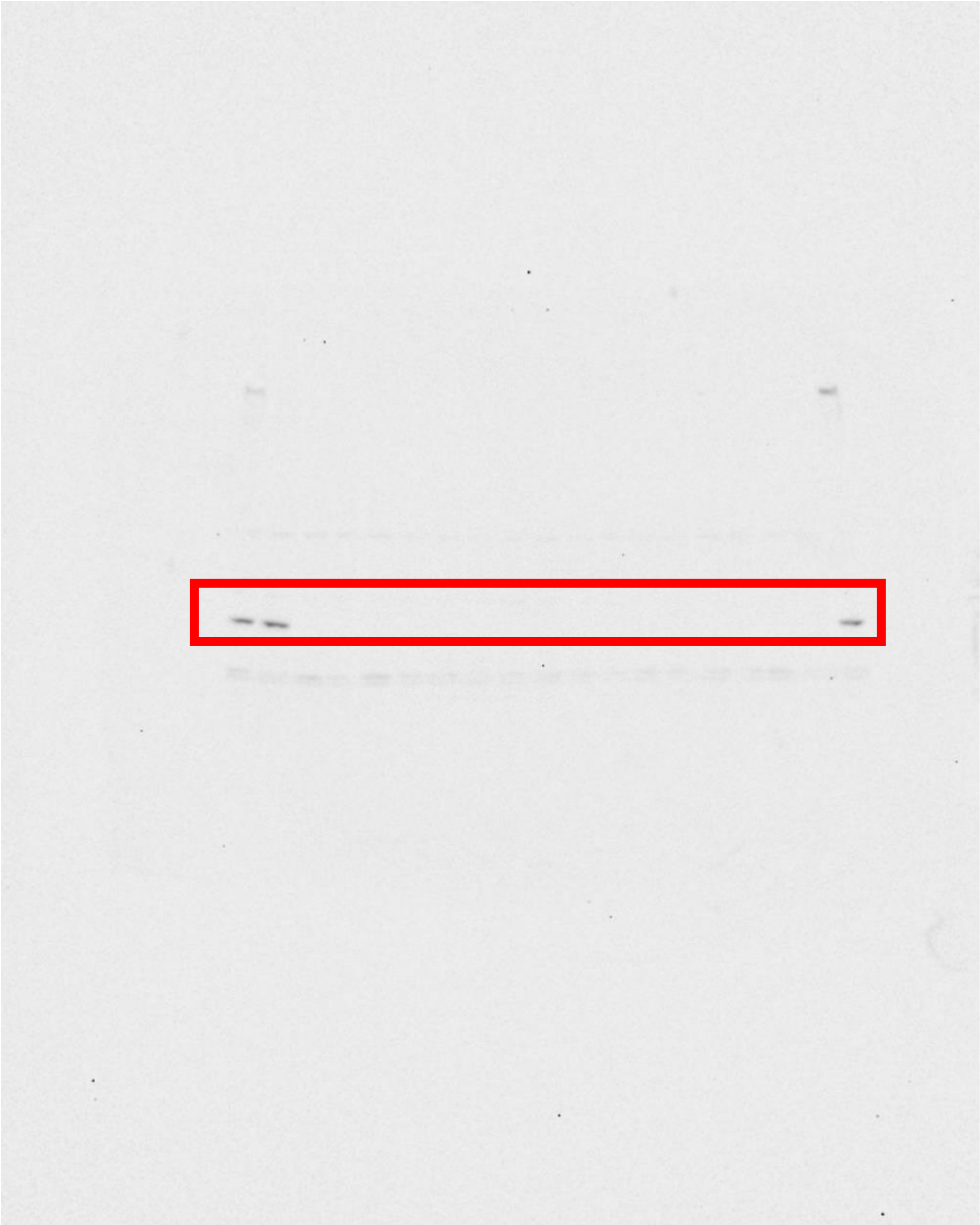

Alpha tubulin

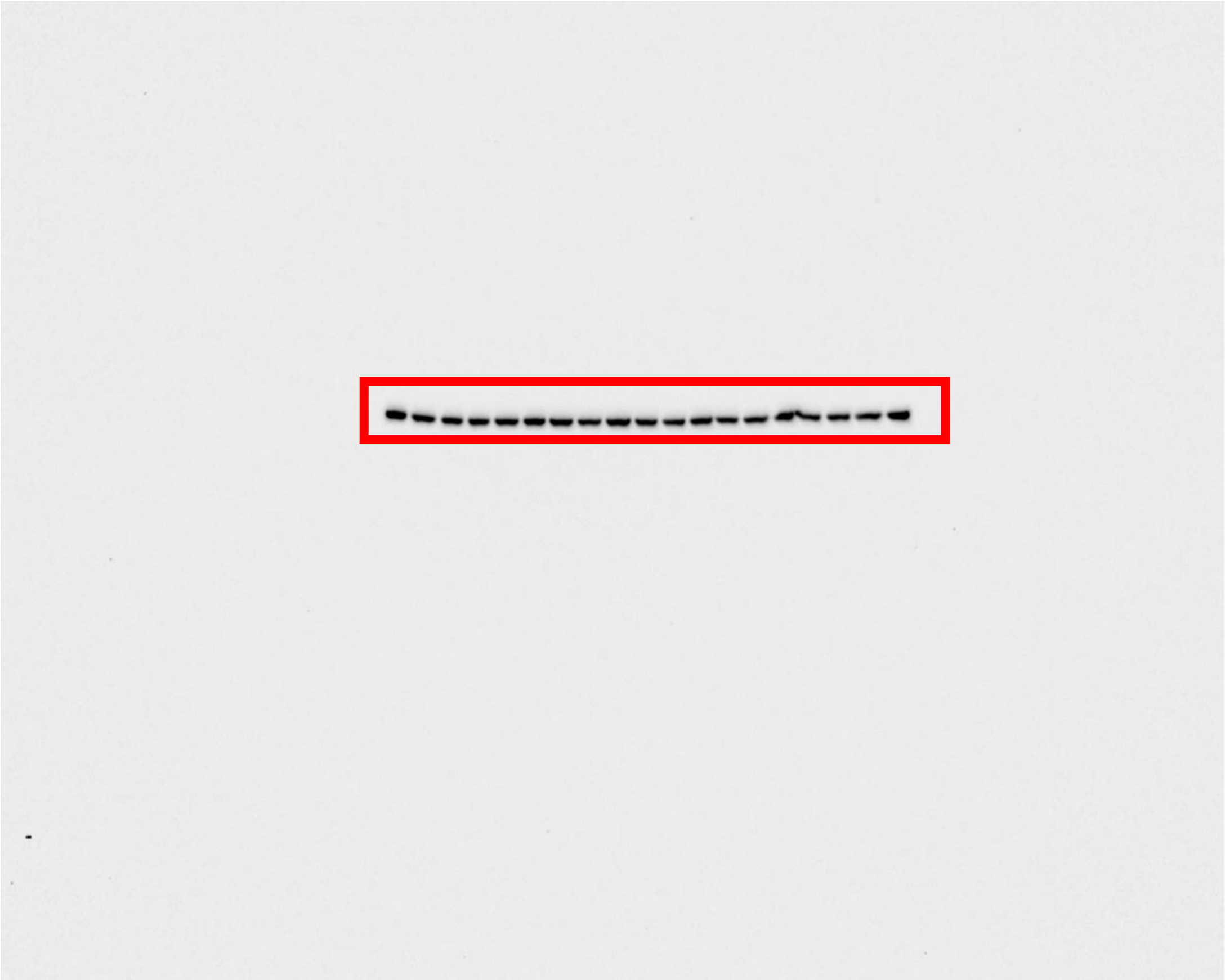

Sup. Fig. 2A

CSA

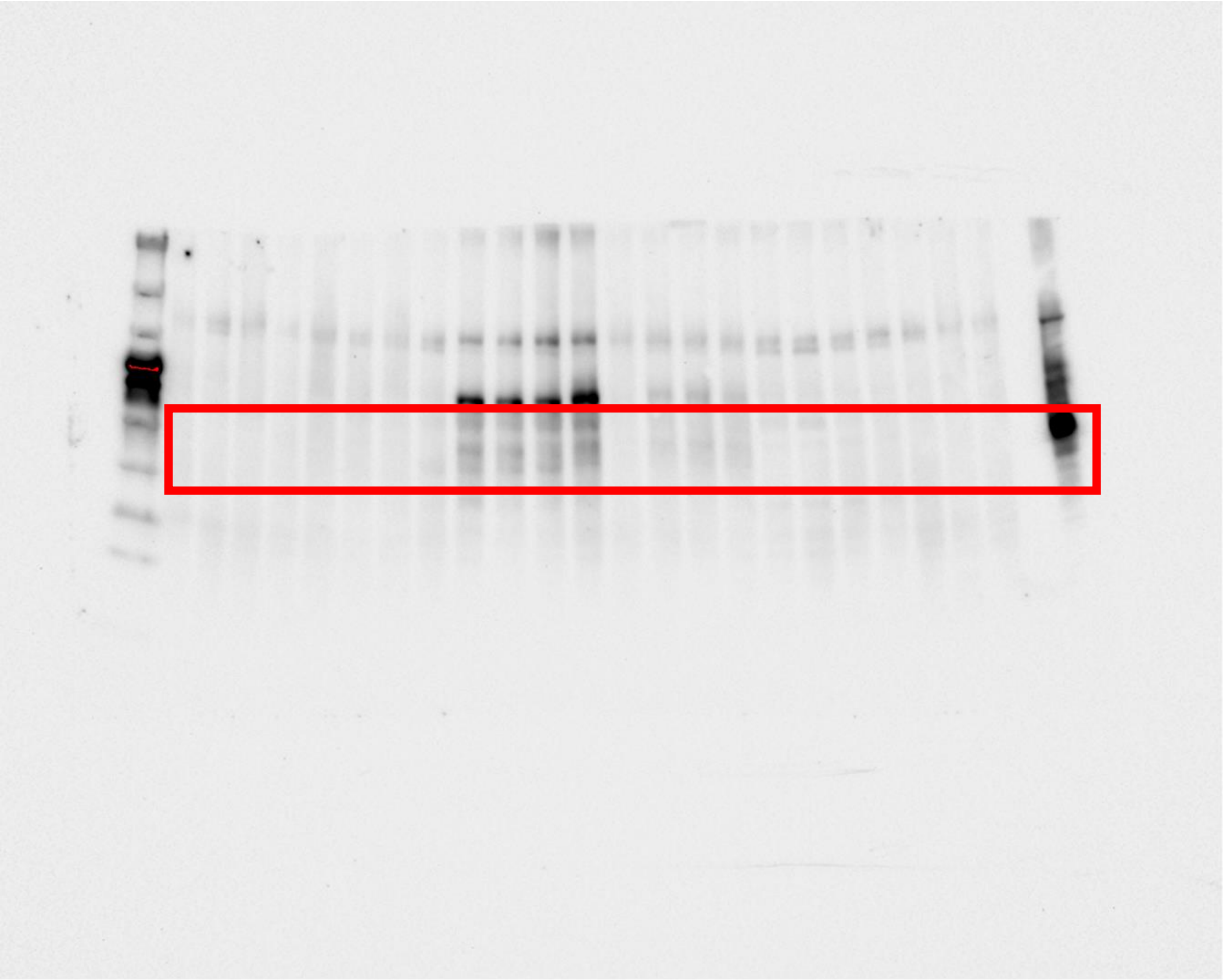

Vinculin

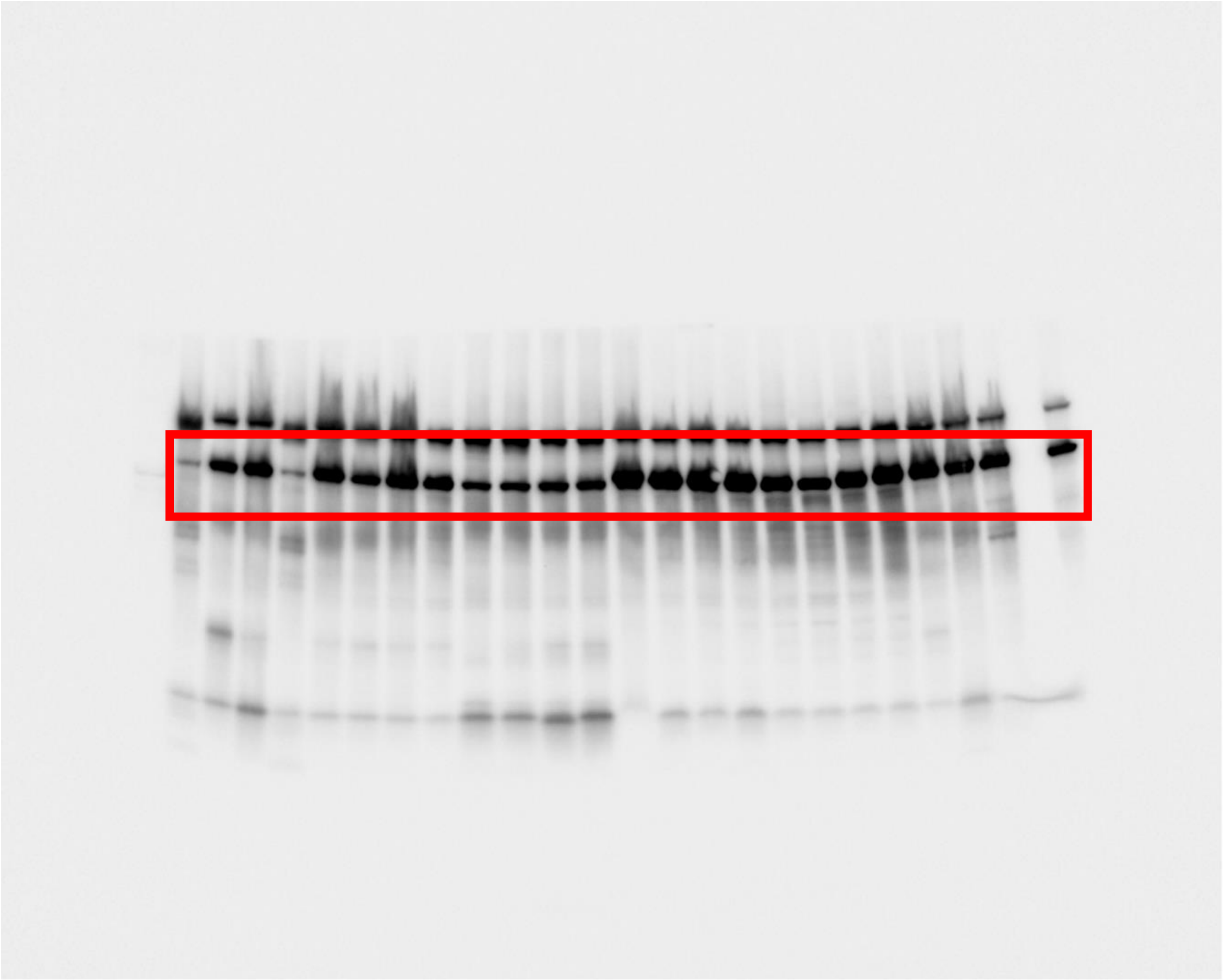

Sup. Fig. 2B

Atf3

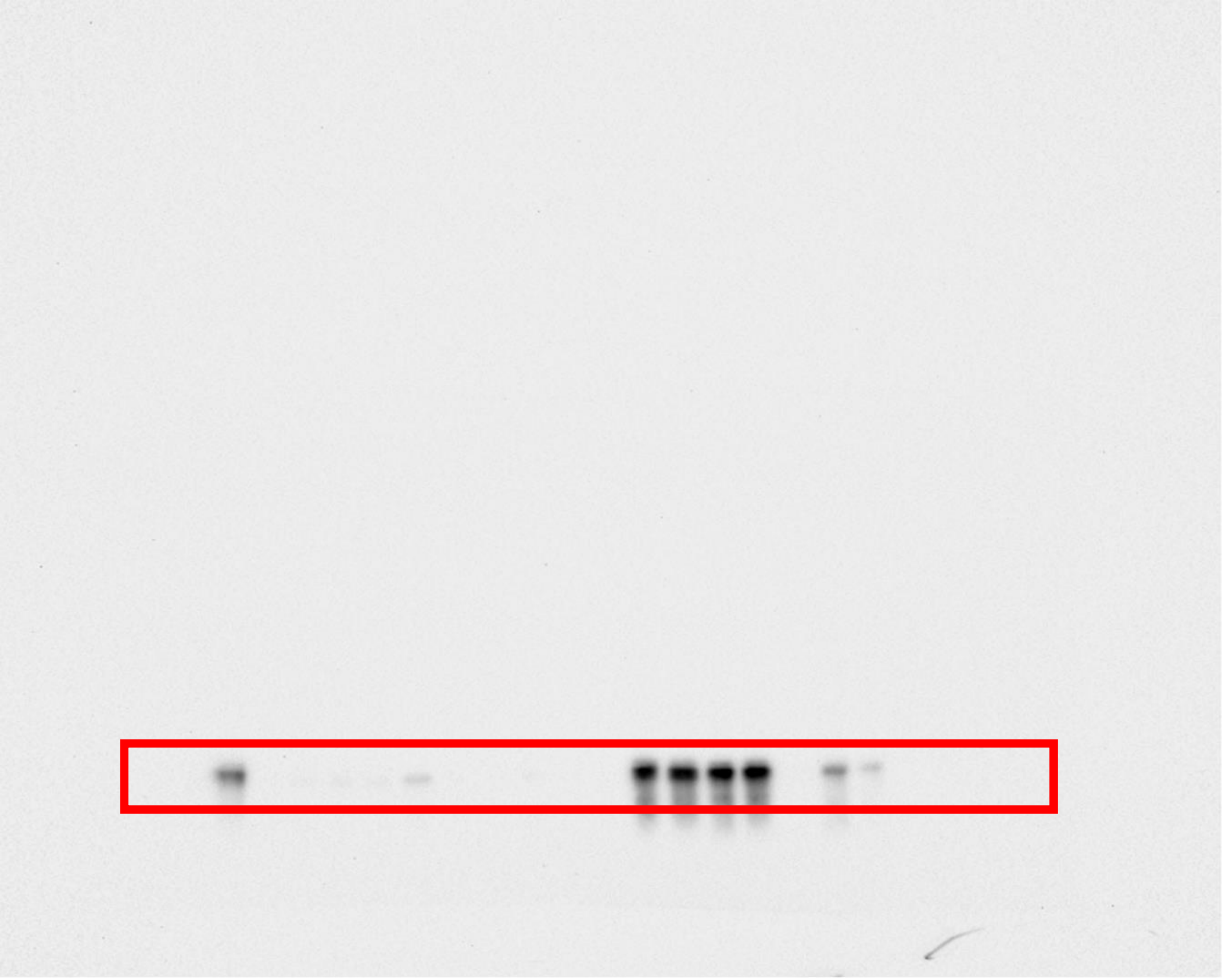

Vinculin

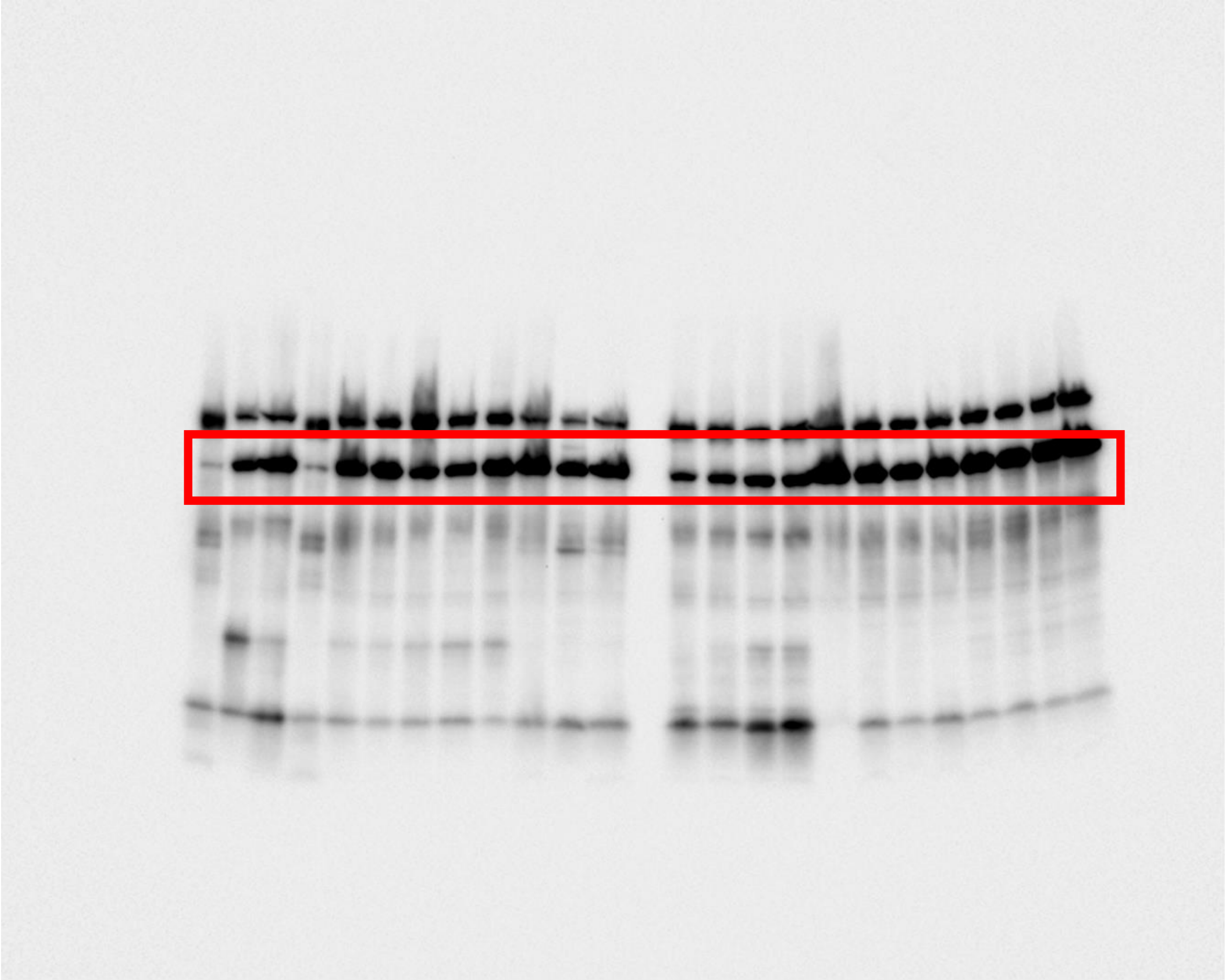

Sup. Fig. 2C

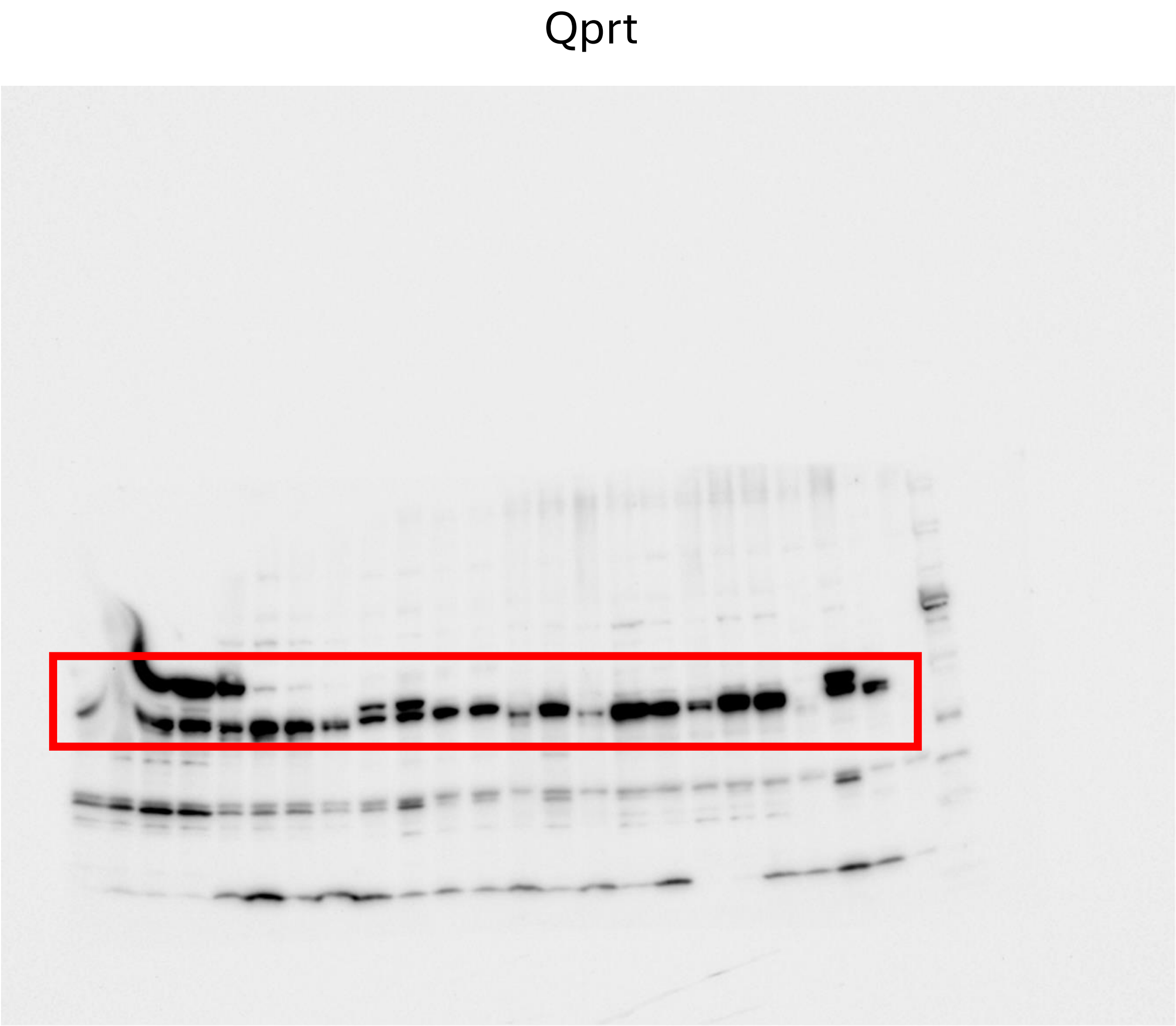

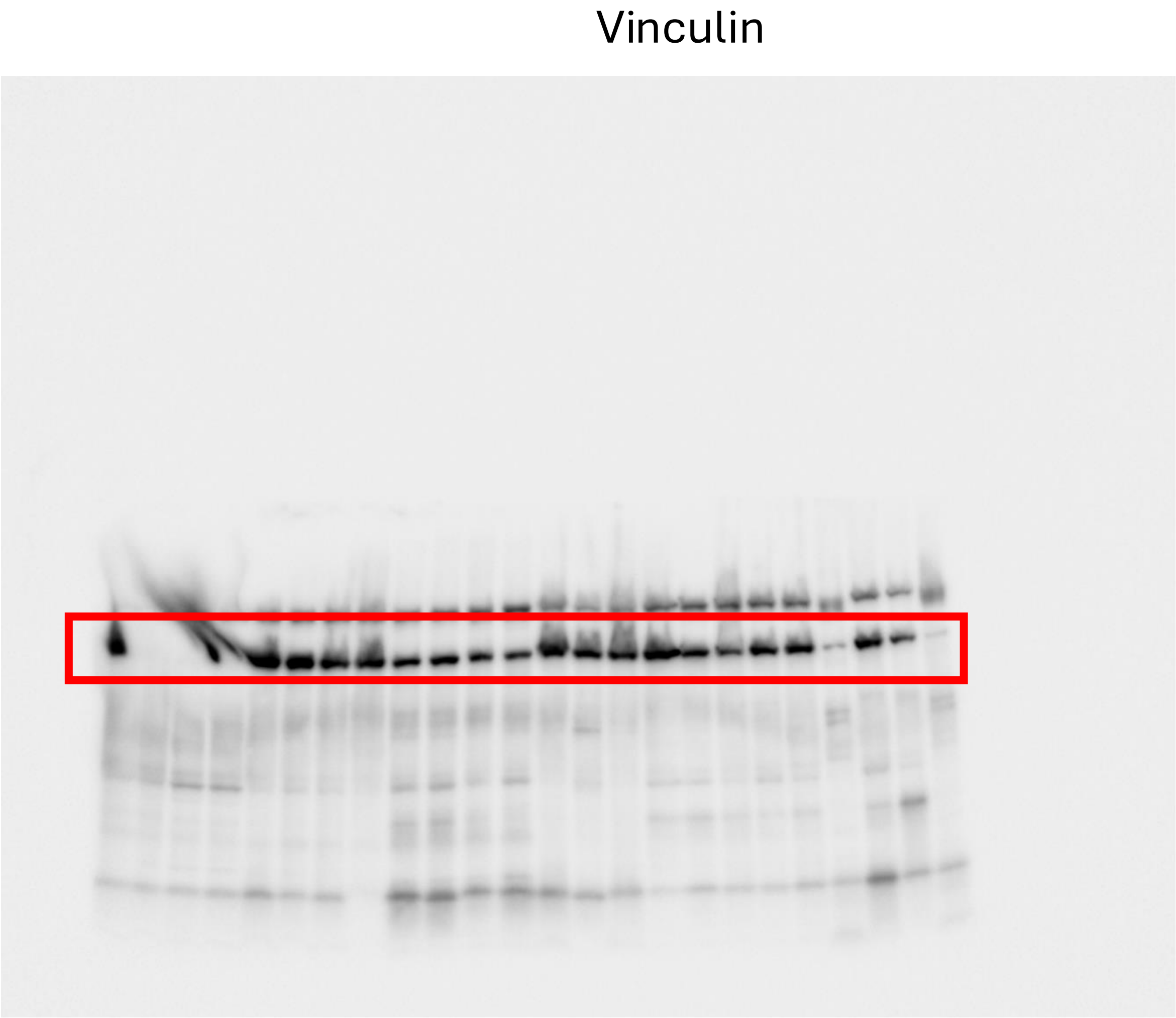

Supplement: Supplementary file 2 [file jci-136-196689-s025.pdf]
